# The 2020 report of the *Lancet* Countdown on health and climate change: responding to converging crises

**DOI:** 10.1016/S0140-6736(20)32290-X

**Published:** 2020-12-02

**Authors:** Nick Watts, Markus Amann, Nigel Arnell, Sonja Ayeb-Karlsson, Jessica Beagley, Kristine Belesova, Maxwell Boykoff, Peter Byass, Wenjia Cai, Diarmid Campbell-Lendrum, Stuart Capstick, Jonathan Chambers, Samantha Coleman, Carole Dalin, Meaghan Daly, Niheer Dasandi, Shouro Dasgupta, Michael Davies, Claudia Di Napoli, Paula Dominguez-Salas, Paul Drummond, Robert Dubrow, Kristie L Ebi, Matthew Eckelman, Paul Ekins, Luis E Escobar, Lucien Georgeson, Su Golder, Delia Grace, Hilary Graham, Paul Haggar, Ian Hamilton, Stella Hartinger, Jeremy Hess, Shih-Che Hsu, Nick Hughes, Slava Jankin Mikhaylov, Marcia P Jimenez, Ilan Kelman, Harry Kennard, Gregor Kiesewetter, Patrick L Kinney, Tord Kjellstrom, Dominic Kniveton, Pete Lampard, Bruno Lemke, Yang Liu, Zhao Liu, Melissa Lott, Rachel Lowe, Jaime Martinez-Urtaza, Mark Maslin, Lucy McAllister, Alice McGushin, Celia McMichael, James Milner, Maziar Moradi-Lakeh, Karyn Morrissey, Simon Munzert, Kris A Murray, Tara Neville, Maria Nilsson, Maquins Odhiambo Sewe, Tadj Oreszczyn, Matthias Otto, Fereidoon Owfi, Olivia Pearman, David Pencheon, Ruth Quinn, Mahnaz Rabbaniha, Elizabeth Robinson, Joacim Rocklöv, Marina Romanello, Jan C Semenza, Jodi Sherman, Liuhua Shi, Marco Springmann, Meisam Tabatabaei, Jonathon Taylor, Joaquin Triñanes, Joy Shumake-Guillemot, Bryan Vu, Paul Wilkinson, Matthew Winning, Peng Gong, Hugh Montgomery, Anthony Costello

**Affiliations:** Institute for Global Health, University College London, London, UK; Air Quality and Greenhouse Gases Program, International Institute for Applied Systems Analysis, Laxenburg, Austria; Department of Meteorology, University of Reading, Reading, UK; Institute for Environment and Human Security, United Nations University, Bonn, Germany; Institute for Global Health, University College London, London, UK; Centre on Climate Change and Planetary Health, London School of Hygiene & Tropical Medicine, London, UK; Environmental Studies Program, University of Colorado Boulder, Boulder, CO, USA; Department of Epidemiology and Global Health, Umeå University, Umeå, Sweden; Department of Earth System Science, Tsinghua University, Beijing, China; Environment, Climate Change and Health Department, World Health Organization, Geneva, Switzerland; School of Psychology, Cardiff University, Cardiff, UK; Institute for Environmental Sciences, University of Geneva, Geneva, Switzerland; Institute for Global Health, University College London, London, UK; Institute for Sustainable Resources, University College London, London, UK; Department of Environmental Studies, University of New England, Biddeford, ME, USA; School of Government, University of Birmingham, Birmingham, UK; Centro Euro-Mediterraneo sui Cambiamenti Climatici, Venice, Italy; Institute for Environmental Design and Engineering, University College London, London, UK; School of Agriculture, Policy, and Development, University of Reading, Reading, UK; Department of Population Health, London School of Hygiene & Tropical Medicine, London, UK; Institute for Sustainable Resources, University College London, London, UK; Yale Center on Climate Change and Health, Yale University, New Haven, CT, USA; Department of Global Health, University of Washington, Seattle, WA, USA; Department of Civil & Environmental Engineering, Northeastern University, Boston, MA, USA; Institute for Sustainable Resources, University College London, London, UK; Department of Fish and Wildlife Conservation, Virginia Polytechnic Institute and State University, Blacksburg, VA, USA; Oxford Martin School, University of Oxford, Oxford, UK; Department of Health Sciences, University of York, York, UK; CGIAR Research Program on Agriculture for Human Nutrition and Health, International Livestock Research Institute, Nairobi, Kenya; Department of Environmental Studies, University of New England, Biddeford, ME, USA; School of Psychology, Cardiff University, Cardiff, UK; Energy Institute, University College London, London, UK; School of Public Health and Administration, Universidad Peruana Cayetano Heredia, Lima, Peru; Center for Health and the Global Environment, University of Washington, Seattle, WA, USA; Energy Institute, University College London, London, UK; Institute for Sustainable Resources, University College London, London, UK; Data Science Lab, Hertie School, Berlin, Germany; Department of Epidemiology, Harvard TH Chan School of Public Health, Harvard University, Boston, MA, USA; Institute for Global Health, University College London, London, UK; Energy Institute, University College London, London, UK; Air Quality and Greenhouse Gases Program, International Institute for Applied Systems Analysis, Laxenburg, Austria; Department of Environmental Health, Boston University, Boston, MA, USA; Health and Environment International Trust, Nelson, New Zealand; School of Global Studies, University of Sussex, Falmer, UK; Department of Health Sciences, University of York, York, UK; School of Health, Nelson Marlborough Institute of Technology, Nelson, New Zealand; Rollins School of Public Health, Emory University, Atlanta, GA, USA; Department of Earth System Science, Tsinghua University, Beijing, China; Center on Global Energy Policy, Columbia University, New York, NY, USA; Centre for Mathematical Modelling of Infectious Diseases, London School of Hygiene & Tropical Medicine, London, UK; Department of Genetics and Microbiology, Universitat Autònoma de Barcelona, Barcelona, Spain; Department of Geography, University College London, London, UK; Center for Energy Markets, Technical University of Munich, Munich, Germany; Institute for Global Health, University College London, London, UK; School of Geography, University of Melbourne, Melbourne, VIC, Australia; Department of Public Health, Environments, and Society, London School of Hygiene & Tropical Medicine, London, UK; Preventive Medicine and Public Health Research Center, Psychosocial Health Research Institute, Iran University of Medical Sciences, Tehran, Iran; European Centre for Environment and Human Health, University of Exeter, Exeter, UK; Data Science Lab, Hertie School, Berlin, Germany; Medical Research Council Centre for Global Infectious Disease Analysis, Department of Infectious Disease Epidemiology, Imperial College London, London, UK; Medical Research Council Unit The Gambia at London School of Hygiene & Tropical Medicine, Bakau, The Gambia, Bakau, The Gambia; Environment, Climate Change and Health Department, World Health Organization, Geneva, Switzerland; Department of Epidemiology and Global Health, Umeå University, Umeå, Sweden; Department of Public Health and Clinical Medicine, Umeå University, Umeå, Sweden; Energy Institute, University College London, London, UK; Department of Arts, Media and Digital Technologies, Nelson Marlborough Institute of Technology, Nelson, New Zealand; Iranian Fisheries Science Research Institute, Agricultural Research, Education, and Extension Organisation, Tehran, Iran; Environmental Studies Program, University of Colorado Boulder, Boulder, CO, USA; Medical and Health School, University of Exeter, Exeter, UK; Department of Civil and Structural Engineering, University of Sheffield, Sheffield, UK; Iranian Fisheries Science Research Institute, Agricultural Research, Education, and Extension Organisation, Tehran, Iran; School of Agriculture, Policy, and Development, University of Reading, Reading, UK; Department of Public Health and Clinical Medicine, Umeå University, Umeå, Sweden; Institute for Global Health, University College London, London, UK; Scientific Assessment Section, European Centre for Disease Prevention and Control, Solna, Sweden; Department of Anesthesiology, Yale University, New Haven, CT, USA; Gangarosa Department of Environmental Health, Atlanta, GA, USA; Oxford Martin School, University of Oxford, Oxford, UK; Institute of Tropical Aquaculture and Fisheries, Universiti Malaysia Terengganu, Kuala Terengganu, Malaysia; Department of Civil Engineering, Tampere University, Tampere, Finland; Department of Electronics and Computer Science, CRETUS Institute, Universidade de Santiago de Compostela, Santiago, Spain; WHO-WMO Joint Climate and Health Office, Geneva, Switzerland; Rollins School of Public Health, Emory University, Atlanta, GA, USA; Department of Public Health, Environments, and Society, London School of Hygiene & Tropical Medicine, London, UK; Institute for Sustainable Resources, University College London, London, UK; Department of Earth System Science, Tsinghua University, Beijing, China; Institute for Human Health and Performance, University College London, London, UK; Office of the Vice Provost for Research, University College London, London, UK

## Abstract

The *Lancet* Countdown is an international collaboration established to provide an independent, global monitoring system dedicated to tracking the emerging health profile of the changing climate.

The 2020 report presents 43 indicators across five sections: climate change impacts, exposures, and vulnerabilities; adaptation, planning, and resilience for health; mitigation actions and health co-benefits; economics and finance; and public and political engagement. This report represents the findings and consensus of the 35 leading academic institutions and UN agencies that make up the *Lancet* Countdown, and draws on the expertise of climate scientists, geographers, engineers, experts in energy, food, and transport, economists, social, and political scientists, data scientists, public health professionals, and doctors.

**The emerging health profile of the changing climate:**

5 years ago, countries committed to limit global warming to “well below 2°C” as part of the landmark Paris Agreement. 5 years on, global carbon dioxide (CO_2_) emissions continue to rise steadily, with no convincing or sustained abatement, resulting in a rise in the global average temperature of 1·2°C. Indeed, the five hottest years on record have occurred since 2015.

The changing climate has already produced considerable shifts in the underlying social and environmental determinants of health at the global level. Indicators in all domains of section 1 (climate change impacts, exposures, and vulnerabilities) are worsening. Concerning, and often accelerating, trends were seen for each of the human symptoms of climate change monitored, with the 2020 indicators presenting the most worrying outlook reported since the *Lancet* Countdown was first established.

These effects are often unequal, disproportionately impacting populations who have contributed the least to the problem. This fact reveals a deeper question of justice, whereby climate change interacts with existing social and economic inequalities and exacerbates longstanding trends within and between countries. An examination of the causes of climate change revealed similar issues, and many carbon-intensive practices and policies lead to poor air quality, poor food quality, and poor housing quality, which disproportionately harm the health of disadvantaged populations.

Vulnerable populations were exposed to an additional 475 million heatwave events globally in 2019, which was, in turn, reflected in excess morbidity and mortality (indicator 1.1.2). During the past 20 years, there has been a 53·7% increase in heat-related mortality in people older than 65 years, reaching a total of 296 000 deaths in 2018 (indicator 1.1.3). The high cost in terms of human lives and suffering is associated with effects on economic output, with 302 billion h of potential labour capacity lost in 2019 (indicator 1.1.4). India and Indonesia were among the worst affected countries, seeing losses of potential labour capacity equivalent to 4–6% of their annual gross domestic product (indicator 4.1.3). In Europe in 2018, the monetised cost of heat-related mortality was equivalent to 1·2% of regional gross national income, or the average income of 11 million European citizens (indicator 4.1.2).

Turning to extremes of weather, advancements in climate science allow for greater accuracy and certainty in attribution; studies from 2015 to 2020 have shown the fingerprints of climate change in 76 floods, droughts, storms, and temperature anomalies (indicator 1.2.3). Furthermore, there was an increase in the number of days people were exposed to a very high or extremely high risk of wildfire between 2001–04 and 2016–19 in 114 countries (indicator 1.2.1). Correspondingly, 67% of global cities surveyed expected climate change to seriously compromise their public health assets and infrastructure (indicator 2.1.3).

The changing climate has downstream effects, impacting broader environmental systems, which in turn harm human health. Global food security is threatened by rising temperatures and increases in the frequency of extreme events; global yield potential for major crops declined by 1·8–5·6% between 1981 and 2019 (indicator 1.4.1). The climate suitability for infectious disease transmission has been growing rapidly since the 1950s, with a 15·0% increase for dengue caused by *Aedes albopictus* in 2018, and regional increases for malaria and *Vibrio* bacteria (indicator 1.3.1). Projecting forward, based on current populations, between 145 million people and 565 million people face potential inundation from rising sea levels (indicator 1.5).

Despite these clear and escalating signs, the global response to climate change has been muted and national efforts continue to fall short of the commitments made in the Paris Agreement. The carbon intensity of the global energy system has remained almost flat for 30 years, with global coal use increasing by 74% during this time (indicators 3.1.1 and 3.1.2). The reduction in global coal use that had been observed since 2013 has now reversed for the past 2 consecutive years: coal use rose by 1·7% from 2016 to 2018. The health burden is substantial—more than 1 million deaths occur every year as a result of air pollution from coal-fired power, and some 390 000 of these deaths were a result of particulate pollution in 2018 (indicator 3.3). The response in the food and agricultural sector has been similarly concerning. Emissions from livestock grew by 16% from 2000 to 2017, with 93% of emissions coming from ruminant animals (indicator 3.5.1). Likewise, increasingly unhealthy diets are becoming more common worldwide, with excess red meat consumption contributing to some 990 000 deaths in 2017 (indicator 3.5.2). 5 years on from when countries reached an agreement in Paris, a concerning number of indicators are showing an early, but sustained, reversal of previously positive trends identified in past reports (indicators 1.3.2, 3.1.2, and 4.2.3).

**A growing response from health professionals:**

Despite little economy-wide improvement, relative gains have been made in several key sectors: from 2010 to 2017, the average annual growth rate in renewable energy capacity was 21%, and low-carbon electricity was responsible for 28% of capacity in China in 2017 (indicator 3.1.3). However, the indicators presented in the 2020 report of the *Lancet* Countdown suggest that some of the most considerable progress was seen in the growing momentum of the health profession’s engagement with climate change globally. Doctors, nurses, and the broader profession have a central role in health system adaptation and mitigation, in understanding and maximising the health benefits of any intervention, and in communicating the need for an accelerated response.

In the case of adaptation in national health systems, this change is underway. Impressively, health services in 86 countries are now connected with their equivalent meteorological services to assist in health adaptation planning (indicator 2.2). At least 51 countries have developed plans for national health adaptation, and global spending in health adaptation rose to 5·3% of all adaptation spending in 2018–19, reaching US$18·4 billion in 2019 (indicators 2.1.1 and 2.4).

The health-care sector, which was responsible for 4·6% of global greenhouse gas emissions in 2017, is taking early but important steps to reduce its own emissions (indicator 3.6). In the UK, the National Health Service has declared an ambition to deliver a net-zero health service as soon as possible, building on a decade of impressive progress in reducing delivery of care emissions by 57% since 1990, and by 22% when considering the service’s supply chain and broader responsibilities. Elsewhere, the Western Australian Department of Health used its 2016 *Public Health Act* to conduct Australia’s first climate and health inquiry, and the German Federal Ministry of Health has established a dedicated department on health protection and sustainability responsible for climate-related matters. This progress is becoming more evenly distributed around the world, with 73% of countries making explicit references to health and wellbeing in their Nationally Determined Contributions under the Paris Agreement, and 100% of countries in the South-East Asia and Eastern Mediterranean regions doing so (indicator 5.4). Similarly, least-developed countries and small island developing states are providing increasing global leadership within the UN General Debate on the connections between health and climate change (indicator 5.4).

Individual health professionals and their associations are also responding well, with health institutions committing to divest more than $42 billion worth of assets from fossil fuels (indicator 4.2.4). In academia, the publication of original research on health and climate changed has increased by a factor of eight from 2007 to 2019 (indicator 5.3).

These shifts are being translated into the broader public discourse. From 2018 to 2019, the coverage of health and climate change in the media increased by 96% world-wide, outpacing the increased coverage of climate change overall, and reaching the highest observed point to date (indicator 5.1). Just as it did with advancements in sanitation and hygiene and with tobacco control, growing and sustained engagement from the health profession during the past 5 years is now beginning to fill a crucial gap in the global response to climate change.

**The next 5 years: a joint response to two public health crises:**

Dec 12, 2020, will mark the anniversary of the 2015 Paris Agreement, with countries set to update their national commitments and review these commitments every 5 years. These next 5 years will be pivotal. To reach the 1·5°C target and limit temperature rise to “well below 2°C”, the 56 gigatonnes of CO_2_ equivalent (GtCO_2_e) currently emitted annually will need to drop to 25 GtCO_2_e within only 10 years (by 2030). In effect, this decrease will require a 7·6% reduction every year, representing an increase in current levels of national government ambition of a factor of five. Without further intervention during the next 5 years, the reductions required to achieve this target increase to 15·4% every year, moving the 1·5°C target out of reach.

The need for accelerated efforts to tackle climate change during the next 5 years will be contextualised by the impacts of, and the global response to, the COVID-19 pandemic. With the loss of life from the pandemic and from climate change measured in the hundreds of thousands, the potential economic costs measured in the trillions, and the broader consequences expected to continue for years to come, the measures taken to address both of these public health crises must be carefully examined and closely linked. Health professionals are well placed to act as a bridge between the two issues, and analogically considering the clinical approach to managing a patient with COVID-19 might be useful in understanding the ways in which these two public health crises should be jointly addressed.

First, in an acute setting, a high priority is placed on rapidly diagnosing and comprehensively assessing the situation. Likewise, further work is required to understand the problem, including: which populations are vulnerable to both the pandemic and to climate change; how global and national economies have reacted and adapted, and the health and environmental consequences of these actions; and which aspects of these shifts should be retained to support longer term, sustainable development. Second, appropriate resuscitation and treatment options are reviewed and administered, with careful consideration of any potential side-effects, the goals of care, and the life-long health of the patient. Economic recovery packages that prioritise outdated forms of energy and transport that are fossil fuel intensive will have unintended side-effects, unnecessarily adding to the 7 million people that die every year from air pollution. Instead, investments in health imperatives, such as renewable energy and clean air, active travel infrastructure and physical activity, and resilient and climate-smart health care, will ultimately be more effective than these outdated methods.

Finally, attention turns to secondary prevention and long-term recovery, seeking to minimise the permanent effects of the disease and prevent recurrence. Many of the steps taken to prepare for unexpected shocks, such as a pandemic, are similar to those required to adapt to the extremes of weather and new threats expected from climate change. These steps include the need to identify vulnerable populations, assess the capacity of public health systems, develop and invest in preparedness measures, and emphasise community resilience and equity. Indeed, without considering the current and future impacts of climate change, efforts to prepare for future pandemics are likely to be undermined.

At every step and in both cases, acting with a level of urgency proportionate to the scale of the threat, adhering to the best available science, and practising clear and consistent communications, are paramount. The consequences of the pandemic will contextualise the economic, social, and environmental policies of governments during the next 5 years, a period that is crucial in determining whether temperatures will remain “well below 2°C”. Unless the global COVID-19 recovery is aligned with the response to climate change, the world will fail to meet the target laid out in the Paris Agreement, damaging public health in the short term and long term.

## Introduction

The world has already warmed by more than 1·2°C compared with preindustrial levels, resulting in profound, immediate, and rapidly worsening health effects, and moving dangerously close to the agreed limit of maintaining temperatures “well below 2°C”.^1–4^ These health impacts are seen on every continent, with the ongoing spread of dengue virus across South America, the cardiovascular and respiratory effects of record heatwaves and wildfires in Australia, western North America, and western Europe, and the undernutrition and mental health effects of floods and droughts in China, Bangladesh, Ethiopia, and South Africa.^5–8^ In the long term, climate change threatens the very foundations of human health and wellbeing, with the *Global Risks Report*^9^ registering climate change as one of the five most damaging or probable global risks every year for the past decade.

It is clear that human and environmental systems are inextricably linked, and that any response to climate change must harness, rather than damage, these connections.^10^ Indeed, a response commensurate to the size of the challenge, which prioritises strengthening healthcare systems, invests in local communities, and ensures clean air, safe drinking water, and nourishing food, will provide the foundations for future generations to not only survive, but to thrive.^11^ Evidence suggests that being more ambitious than current climate policies by limiting warming to 1·5°C by 2100 would generate a net global benefit of US$264–610 trillion.^12^ The economic case of expanding ambition is further strengthened when the benefits of a healthier workforce and reduced health-care costs are considered.^13–15^

The present day effects of climate change will continue to worsen without meaningful intervention. These tangible, if less visible, impacts on public health have so far resulted in a delayed and inadequate policy response. By contrast, and on a considerably shorter timescale, COVID-19, the disease caused by severe acute respiratory syndrome coronavirus 2, has rapidly developed into a global public health emergency. Since COVID-19 was first detected in December, 2019, the loss of life and livelihoods has occurred with staggering speed. However, as for climate change, much of the impact is expected to unfold over the coming months and years, and is likely to disproportionately affect vulnerable populations as both the direct effects of the virus, and the indirect effects of the response to the virus, are felt throughout the world. Several lessons and parallels between climate change and COVID-19 are discussed in panel 1, focusing on the response to, and the recovery from, the two health crises.

The *Lancet* Countdown exists as an independent, multidisciplinary collaboration dedicated to tracking the links between public health and climate change. It brings together 35 academic institutions and UN agencies from every continent, and structures its work across five key sections: climate change impacts, exposures, and vulnerabilities; adaptation, planning, and resilience for health; mitigation actions and health co-benefits; economics and finance; and public and political engagement (panel 2). The 43 indicators and conclusions presented in this report are the cumulative result of the past 8 years of collaboration, and represent the consensus of climate scientists, geographers, engineers, experts in energy, food, and transport, economists, social and political scientists, public health professionals, and doctors.

Where the COVID-19 pandemic has direct implications for an indicator being reported (and where accurate data exists to allow meaningful commentary), these implications are discussed in-text. Beyond this deviation, the 2020 report of the *Lancet* Countdown maintains focus on the connections between public health and climate change, and the collaboration worked hard to ensure the continued high quality of its indicators, with only minor amendments and omissions resulting from the ongoing disruptions.

### Expanding and strengthening a global monitoring system for health and climate change

the *Lancet* Countdown’s work draws on decades of underlying scientific progress and data, with the initial indicator set selected as part of an open, global consultation that sought to identify which of the connections between health and climate change could be meaningfully tracked.^27^ Proposals for indicators were considered and adopted on the basis of numerous criteria, including the existence of a credible underlying link between climate change and health that was well described in the scientific literature; the availability of reliable and regularly updated data across expanded geographical and temporal scales; the presence of acceptable methods for monitoring; and the relevance to policy and availability of actionable interventions.

An iterative and adaptive approach has substantively improved most of these initial indicators and resulted in the development of several additional indicators. Given this approach, and the rapidly evolving nature of the scientific and data landscape, each annual update replaces the analysis from previous years. The methods, sources of data, and improvements for each indicator are described in full in the appendix, which is an essential companion to the main report.

The 2020 report of the *Lancet* Countdown reflects an enormous amount of work done during the past 12 months to refine and improve these indicators, including the annual update of the data. Several key developments have occurred.

Methods and datasets have been strengthened and standardised for indicators that capture heat and heatwaves, floods and droughts, wildfires, the climate suitability for infectious disease transmission, food security and undernutrition, health adaptation spending, food and agriculture, low-carbon health care, the economics of air pollution, and engagement in health and climate change from the media, the scientific community, and individuals.

Geographical or temporal coverage have been improved or expanded for indicators that track heat and heatwaves, labour capacity loss, floods and droughts, the climate suitability for infectious disease transmission, climate change risk assessments in cities, the use of clean household energy, and household air pollution.

New indicators have been developed to explore heat-related mortality, migration and population displacement, access to urban green space, the health benefits of low-carbon diets, the economic costs of extremes of heat and of labour capacity loss, net carbon pricing, and the extent to which the UN Framework Convention on Climate Change’s (UNFCCC) Nationally Determined Contributions (NDCs) engage with public health.

This continued progress has been supported by the *Lancet* Countdown’s scientific advisory group and the creation of a new, independent, quality improvement process, which provided independent expert input on the indicators before the formal peer review process, adding rigour and transparency to the collaboration’s research. In every case, the most up-to-date data available are presented, with the precise nature and timing of these updates varying depending on the data source. This presentation of data has occurred despite the impact of COVID-19, which has only affected the production of a small subset of indicators for this report.

The *Lancet* Countdown has also taken several steps to ensure that it has the expertise, data, and representation required to build a global monitoring system. Partnering with Tsinghua University, Beijing, China, and Universidad Peruana Cayetano Heredia, Lima, Peru, the collaboration launched two new regional offices for South America (in Lima), and for Asia (in Beijing), and developed a new partnership to build capacity in west Africa. This expansion is coupled with ongoing work to develop national and regional *Lancet* Countdown reports in Australia (in partnership with the *Medical Journal of Australia*), the EU (in partnership with the European Environment Agency), China, and the USA. At the same time, a new data visualisation platform has been launched, allowing health professionals and policy makers to investigate the indicators in this report.

Future work will concentrate on supporting these regional and national efforts, building capacity for communications and engagement, developing new indicators (with a particular interest in developing indicators related to mental health and gender), and further improving existing indicators. To this end, the continued growth of the *Lancet* Countdown depends on the dedication of each of its composite experts and partners, continued support from the Wellcome Trust, and ongoing input and offers of support from new academic institutions willing to build on the analysis published in this report.

## Section 1: climate change impacts, exposures, and vulnerabilities

A changing climate threatens to undermine the past 50 years of gains in public health, disrupting the well-being of communities and the foundations on which health systems are built.^28^ The effects of climate change are pervasive and impact the food, air, water, and shelter that society depend on, extending across every region of the world and every income group. These effects act to exacerbate existing inequities, with vulnerable populations within and between countries affected more frequently and with a more lasting impact.^3^

Section 1 of the 2020 report tracks the links between climate change and human health along several exposure pathways, from the climate signal through to the resulting health outcome. This section begins by examining several dimensions of the effects of heat and heatwaves, ranging from exposure and vulnerability through to labour capacity and mortality (indicators 1.1.1–1.1.4). The indicator on heat-related mortality has been developed for the 2020 report, and, although ongoing work will strengthen these findings in subsequent years, this indicator complements existing indicators on exposure and vulnerability to heat and represents an important step forward.

Indicators 1.2.1–1.2.3 navigate the effects of extreme weather events, tracking wildfires, floods and droughts, and the lethality of extreme weather events. The wildfire indicator now tracks the risk of, and the exposure to, wildfires, the classification of drought has been updated to better align with climate change trends, and the attribution of the health effects of extreme weather events to climate change is presented. The climate suitability for the transmission of infectious diseases and the vulnerability of populations to infectious diseases were monitored, and so too were the evolving impacts of climate change on terrestrial and marine food security (indicators 1.3.1–1.4.2). The consideration of regional variation provided robust estimates of the effects of rising temperatures on crop yield potential. Indicator 1.5, which tracks exposure to rising sea levels in the context of migration and displacement, the resulting health effects, and policy responses, closes this section.

### Indicator 1.1: health and heat

Exposure to high temperatures and heatwaves results in a range of negative health impacts, from morbidity and mortality due to heat stress and heatstroke to exacerbations of cardiovascular and respiratory disease.^29,30^ The worst affected are those older than 65 years, those with disabilities or pre-existing medical conditions, those working outdoors or in non-cooled environments, and those living in regions already at the limits for human habitation.^31^ The following indicators track the vulnerabilities, exposures, and impacts of heat and heatwaves in every region of the world.

#### Indicator 1.1.1: vulnerability to the extremes of heat—headline finding: vulnerability to the extremes of heat continues to increase in every region of the world, led by populations in Europe, with the Western Pacific region, South-East Asia region, and the African region all seeing an increase of more than 10% since 1990

This indicator re-examines the index results presented in the 2019 report,^28^ which combines data on the proportion of the population older than 65 years; the prevalence of chronic respiratory disease, cardiovascular disease, and diabetes in this population, and the proportion of the total population living in urban areas. It also introduces a more comprehensive index of heat vulnerability, combining these aforementioned factors with heatwave exposure data and the International Health Regulations capacity score.

Since 1990, as a result of ageing populations, the high prevalence of chronic disease, and rising levels of urbanisation, populations in the European and Eastern Mediterranean regions have been the most vulnerable to the extremes of heat of all the WHO regions. In 2017, vulnerability was 40·6% in the European region and 38·7% in the Eastern Mediterranean region. However, no WHO region is immune and vulnerability has worsened everywhere. From 1990 to 2017, vulnerability increased in the African region (28·4% to 31·3%), the South-East Asia region (28·3% to 31·3%), and the Western Pacific region (33·2% to 36·6%). By taking into account health system strengthening and heatwave exposure across these regions, this vulnerability indicator can be usefully built into one that captures population risk, which has been done for the 2020 report (appendix pp 4–5). This new indicator shows trends similar to those aforementioned, with risk rising in every region. This index will be further developed during the course of 2020, and presented in full, alongside a broader suite of risk indicators, in future reports.

#### Indicator 1.1.2: exposure of vulnerable populations to heatwaves—headline finding: a record 475 million additional exposures to heatwaves affecting vulnerable populations were observed in 2019, representing some 2·9 billion additional days of heatwaves experienced

**Figure 1:**
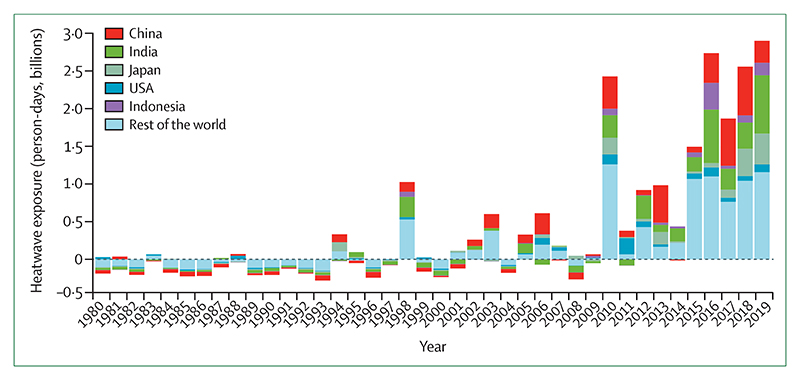
Change in days of heatwave exposure relative to the 1986–2005 baseline in people older than 65 years The dotted line at 0 represents baseline.

Since 2010, there has been an increase in the number of days of heatwave exposure, relative to a 1986–2005 base-line, in the population older than 65 years (figure 1). This rise has been driven by the combination of increasing heatwave occurrences and ageing populations. In 2019, there were 475 million additional exposure events. Expressed as the number of days in which a heatwave was experienced, this number breaks the previous 2016 record by an additional 160 million person-days.

Indicator 1.1.2 tracks the exposure of vulnerable populations to heatwaves and has now been updated to make use of the latest climate data and a hybrid population dataset.^32–34^ This indicator has undergone several additional improvements to best capture heatwave exposure in every region of the world, including an improved definition of heatwave, the quantification of exposure days to capture changing frequency and duration, and improved estimates of demographic breakdown (appendix pp 6–11).

#### Indicator 1.1.3: heat-related mortality—headline finding: from 2000 to 2018, heat-related mortality in people older than 65 years increased by 53·7% and, in 2018, reached 296 000 deaths, the majority of which occurred in Japan, eastern China, northern India, and central Europe

This metric, newly created for the 2020 report, tracks global heat-related mortality in populations older than 65 years. By use of methods originally described by WHO, this indicator applies the exposure-response function and optimum temperature described by Honda and colleagues^35^ to the daily maximum temperature exposure of the population older than 65 years to estimate the attributable fraction and thus the heat-related excess mortality.^36^ As with indicator 1.1.2, data on daily maximum temperature were taken from the European Centre for Medium-Range Weather Forecasts’ fifth reanalysis (ERA5) and gridded population data were taken from a hybrid of the National Aeronautics and Space Administration’s gridded population of the world (version four) and the Inter-Sectoral Impact Model Intercomparison Project, with full methodology described in the appendix (pp 12–13).^32–34^

**Figure 2:**
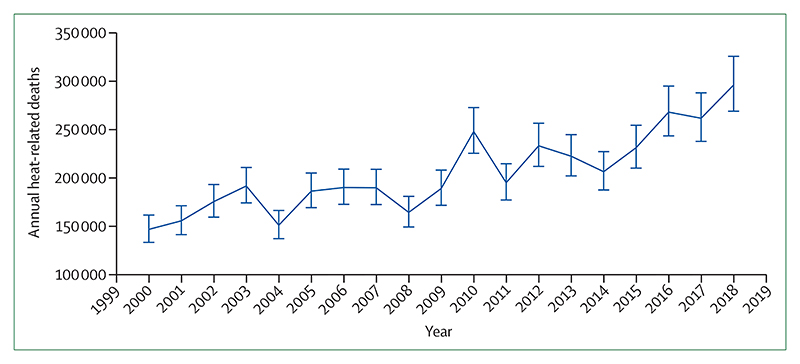
Global heat-related mortality for populations older than 65 years The error bars were calculated on the basis of the uncertainty range of the exposure-response function, as described by Honda and colleagues.^35^

**Figure 3:**
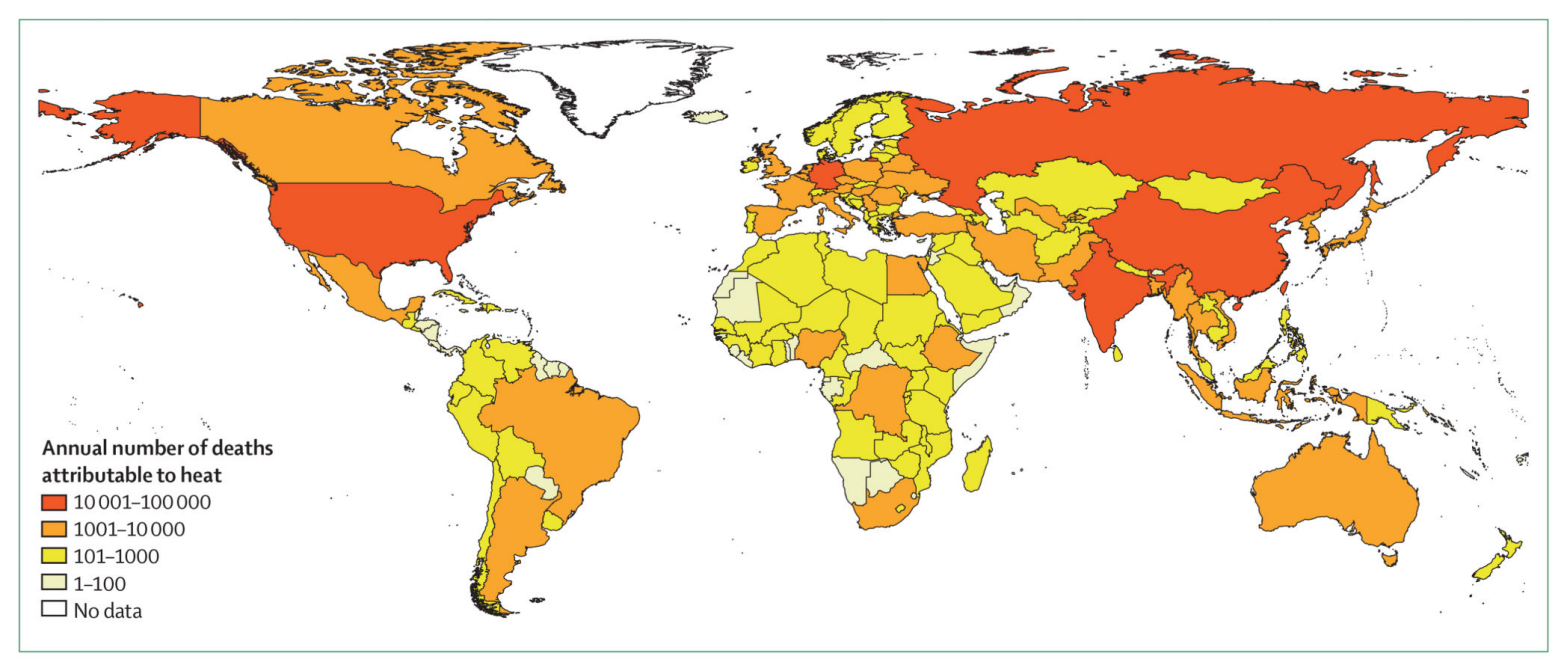
Annual heat-related mortality in the population older than 65 years averaged from 2014 to 2018

This indicator estimates that the global average heat-related mortality per year in people older than 65 years has increased by 53·7% from 2000–04 to 2014–18, with a total of 296 000 deaths in 2018 (figures 2, 3). With the largest populations, China (62 000 deaths) and India (31 000 deaths) had the most deaths in 2018, followed by Germany (around 20 200 deaths), the USA (almost 19 000 deaths), Russia (18 600 deaths), and Japan (around 14 200 deaths). At more than 104 000 deaths, the European region was the most affected of the WHO regions. Importantly, the effects of temperature on mortality vary by region and are modified by local factors, including population urban green space and inequality, both within and between countries.^37,38^ Work has begun to develop a future form of this indicator, which builds in more localised exposure-response functions as these functions become available.

#### Indicator 1.1.4: change in labour capacity—headline finding: rising temperatures were responsible for an excess of 100 billion potential work h lost globally in 2019 compared with those lost in 2000, with India’s agricultural sector among the worst affected

Indicator 1.1.4 tracks the effects of heat exposure on working people, with impact expressed as potential work hours lost.^39^ This indicator has been updated to capture construction, service, manufacturing, and agricultural sectors, and used climate data from the ERA5 models, with methods and data described in full previously and in the appendix (pp 13–16).^33,40–43^

Across the globe, a potential 302 billion work h were lost in 2019, which is 103 billion h more than that lost in 2000. 13 countries represented 244·1 billion (80·7%) of the 302·4 billion global work h lost in 2019 (table 1), with India having the greatest total loss and Cambodia having the highest per-capita loss of any country. In many countries in the world, agricultural workers see the worst of these effects, whereas, in high-income countries, such as the USA, the burden is often on those in the construction sector.

### Indicator 1.2: health and extreme weather events

Extreme weather events, including wildfires, floods, storms, and droughts, affect human health in various ways, with the frequency and intensity of such events shifting as a result of climate change. Death and injury as a direct consequence of an extreme event are often compounded by effects that are mediated through the environment—eg, the exacerbation of respiratory symptoms from wildfire smoke and the spread of vector-borne and water-borne diseases following a flood or drought. Impacts are also mediated through social systems—eg, the disruption to health services and the mental ill health that can be caused by storms and fires.^3,44^ The following indicators track the risk and exposure of the population to wildfires, changes in meteorological flood and drought, and the lethality of extreme weather events.

#### Indicator 1.2.1: wildfires—headline finding: in 114 countries, there was an increase in the number of days people were exposed to very high or extremely high risk of danger from fire in 2016–19 compared with 2001–04. This increased risk translated into an increase in population exposure to wildfires in 128 countries

**Figure 4:**
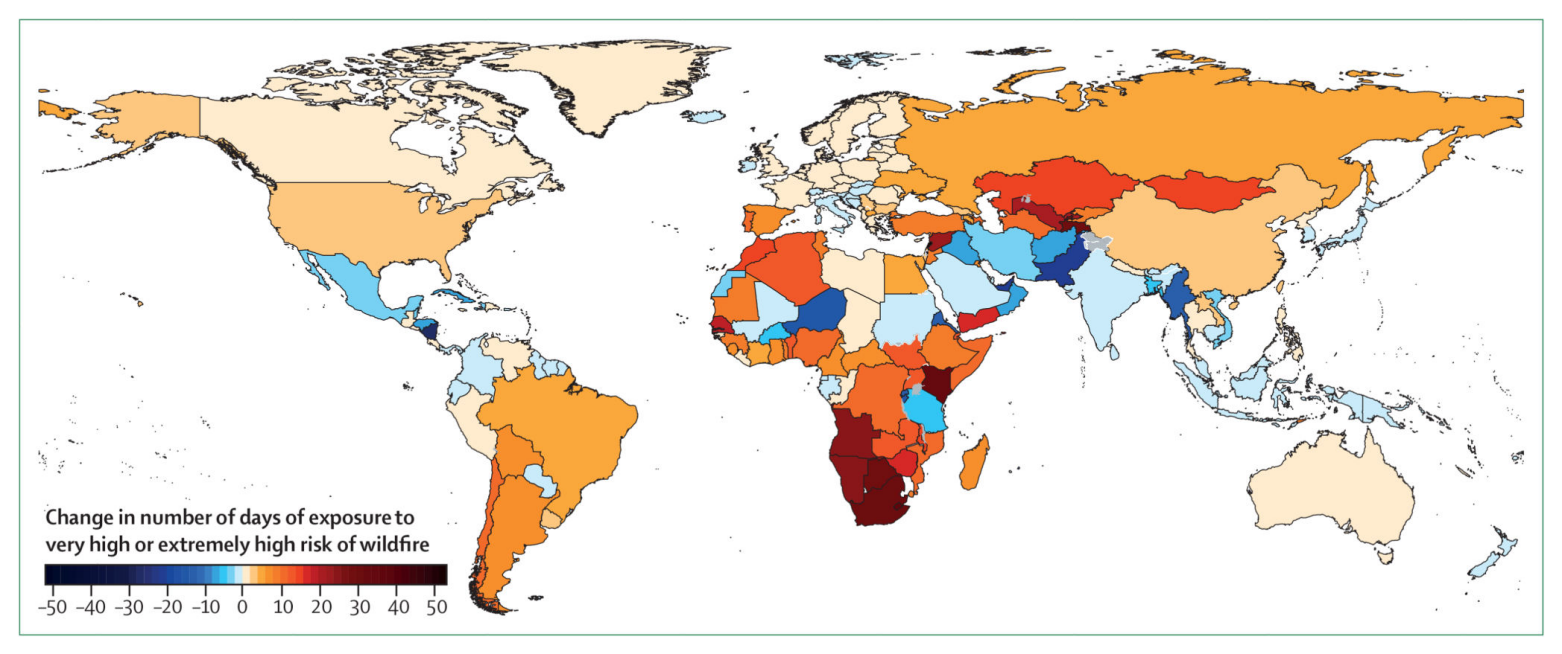
Population-weighted average changes in the number of days of exposure to very high or extremely high risk of wildfire in 2016–19 compared with 2001–04 Large urban areas with a population density of 400 people per km^2^ or more are excluded. Wildfire risk is based on the Fire Danger Index, which rates risk on a scale from 1 to 6 (1 is very low; 2 is low; 3 is medium; 4 is high; 5 is very high; and 6 is extremely high). The higher the number, the more favourable the meteorological conditions are to trigger a wildfire.

For the 2020 report, analysis on the effects of wildfires has been developed to track the average number of days people are exposed to very high or extremely high risk (figure 4) of wildfire annually and the change in actual population exposure to wildfires across the globe. The indicator uses both model-based risk to wildfires and satellite-observed exposure. Climatological wildfire risk was estimated by combining daily very high or extremely high wildfire risk (a fire danger index score of 5 or 6) with climate and population data for every 0·25° × 0·25° global grid cell.^32,45^ For wildfire exposure, satellite-observed active fire spots were detected by use of the Moderate Resolution Imaging Spectroradiometer, and then aggregated and spatially joined with gridded population data on a global grid with a resolution of 10 km, with urban areas excluded.^32,46^ A full description of the methodology can be found in the appendix (pp 17–18).

Compared with the period 2001–04, there was an increase in the risk of wildfire in 114 (58%) of 196 countries in 2016–19, with the largest increases occurring in Lebanon, Kenya, and South Africa (figure 4). Considering area-weighted, rather than population-weighted change, Australia, devastated by the 2019–20 fire season, had one of the largest increases in wildfire risk. During 2016–19, this increased risk translated into an additional 194 000 daily exposures to wildfires per year around the world, and an increase in population exposure to wild-fires in 128 countries, compared with 2001–04. Driven by the record breaking fires in 2017 and 2018, the USA saw one of the largest increases globally, with more than 470 000 additional daily exposures to wildfires per year occurring in 2016–19 compared with 2001–04.

#### Indicator 1.2.2: flood and drought—headline finding: in 2018, the global land surface area affected by excess drought was more than twice that of a historical baseline

Climate change alters hydrological cycles, tending to make dry areas drier and wet areas wetter.^3^ By altering rainfall patterns and increasing temperatures, climate change affects the intensity, duration, and frequency of drought events.^3,47^ Drought poses multiple risks for health, threatening drinking water supplies and sanitation, and crop and livestock productivity, enhancing the risk of wildfires, and potentially leading to forced migration.^48^ Additionally, altered precipitation patterns increase the risk of localised flood events, resulting in direct injury, the spread of infectious diseases, and impacts on mental health.^49^

In the 2020 report, meteorological drought is tracked by use of the standardised precipitation evapotranspiration index, which considers both precipitation and temperature, and the effect of temperature on the loss of soil moisture. This index measures significant increases in the number of months of drought compared with an extended historical baseline (1950–2005) to account for periodic variations such as those generated by the El Niño Southern Oscillation.^50^ A full explanation of the methodology and additional analysis are in the appendix (pp 19–21).

In 2018, there was a larger number of exceptional drought events affecting all populated continents and the global land surface area affected by an excess number of months in drought was more than twice that of the historical base-line. Areas that saw unusually high numbers of months with excess drought in 2018 included Europe, the Eastern Mediterranean region, and, specifically, Mongolia.

#### Indicator 1.2.3: lethality of extreme weather events—headline finding: from 1990 to 2019, the long-term, increasing trends in the number of weather-related disasters were accompanied by an increase in the number of people affected by these disasters in countries where health-care expenditure had reduced or had minimally increased during 2000–17

The links between climate change and the health effects of extreme weather events are presented in two ways for this indicator. The first part studies long-term trends in the occurrence of such events, along with changes in the number of people affected, and the resultant mortality. The methods and data for this analysis are similar to those used in previous reports and are described in full in the appendix (pp 22–24).^51^ Recognising that an increase in the variability and intensity of these events is also expected, the second part considers the attribution of individual extreme weather events to climate change, and the effects that a selection of events have had on the health of populations (table 2, panel 3).

From 1990 to 2019, there were clear, significant, increasing trends in the number of occurrences of weather-related disasters, but no significant difference in the number of people affected per event or the number of deaths per event. Within the subset of countries that had a reduction, or a minimal increase in, health-care expenditure from 2000 to 2017, a significant increase in the number of people affected by extreme weather events was identified. By contrast, in countries with the greatest increase in health-care expenditure in 2000–17, the number of people affected by extreme weather events decreased between 1990 and 2019, despite an increasing frequency of events. One possible explanation for this finding could be the adaptive effects of health system strengthening. This relationship will be further explored in future reports from the *Lancet* Countdown by considering variables, such as expenditure for specific health-care functions and excess deaths, in addition to the immediate event-related deaths.

### Indicator 1.3: climate-sensitive infectious diseases

#### *Indicator 1.3.1: climate suitability for infectious disease transmission—headline finding: changing climatic conditions are increasingly suitable for the transmission of numerous infectious diseases. From 1950 to 2018, the global climate suitability for the transmission of dengue increased by 8·9% for* Aedes aegypti *and 15·0% for* Aedes albopictus. *In 2015–19, suitability for malaria transmission in highland areas was 38·7% higher in the African region and 149·7% higher in the Western Pacific region compared with a 1950s baseline*

Climate change is affecting the risk to humans and the distribution of many infectious diseases, including vector-borne, food-borne, and water-borne diseases.^3^ By use of three different models, this indicator tracks the change in climate suitability for the transmission of infectious diseases of particular global importance: dengue, malaria, and pathogenic *Vibrio* bacteria (ie, *Vibrio parahaemolyticus, Vibrio vulnificus*, and non-toxigenic *Vibrio cholerae*). Temperature-driven, process-based mathematical models were used to capture the change in vectorial capacity of *A aegypti* and *A albopictus* for the transmission of dengue compared with a 1950s baseline.^94^ Change in the climate suitability for *Plasmodium falciparum* malaria was modelled on the basis of empirically derived thresholds of precipitation, temperature, and relative humidity and compared with a 1950s baseline.^94^ Highland areas (ie, those ≥1500 m above sea level) are highlighted in the model because increasing temperatures are eroding the effect altitude has as a barrier to malaria transmission, which has resulted in more favourable conditions in densely populated highland areas, as seen in Ethiopia.^95^ In the case of pathogenic *Vibrio* spp, which cause a range of human infections, including gastroenteritis, wound infections, sepsis, and cholera, 2019 and 2016–19 average climate suitability were compared with a 1980s global baseline and between one region each in Europe (the Baltics), the Atlantic Northeast coast of the USA, and the Pacific Northwest coast of North America.^96–98^ Full descriptions of the context of these diseases, the methodology of the models, and additional analysis can be found in the appendix (pp 25–33).

**Figure 5:**
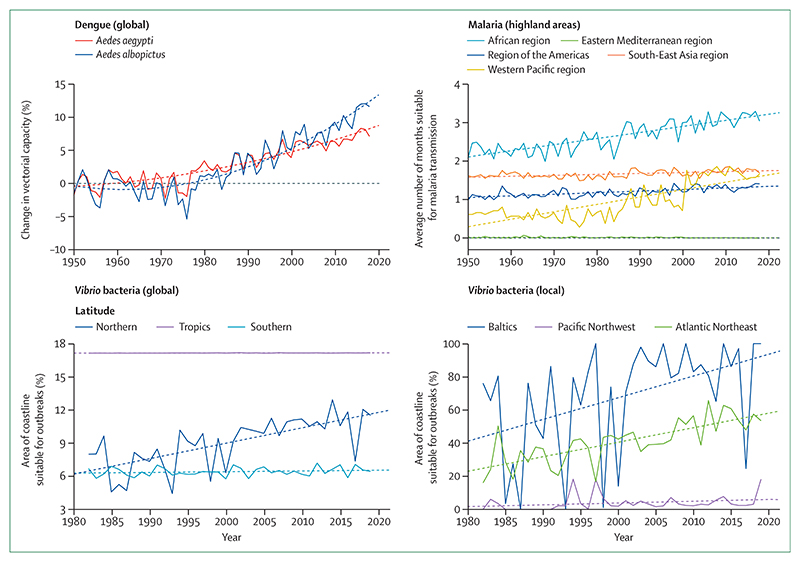
Change in climate suitability for infectious diseases Solid lines represent the annual change. Dashed lines represent the trend since 1950 (for dengue and malaria) and 1982 (for *Vibrio* bacteria).

Climate suitability for disease transmission increased globally for all diseases tracked. 2018 was particularly favourable for the transmission of dengue, with a global rise in vectorial capacity of 8·9% for *A aegypti* and 15·0% for *A albopictus* compared with a 1950s baseline (figure 5). Although average suitability for dengue remained low in Europe, 2018 was the most suitable year yet recorded for both vector species in this region, with a change from the 1950s baseline of 25·8% for *A aegypti* and 40·7% for *A albopictus*. There have been significant increases in the environmental suitability for the transmission of falciparum malaria in highland areas of four of the five malaria endemic regions, with an increase of 38·7% in the African region and 149·7% in the Western Pacific region in 2015–19 compared with the 1950s baseline (figure 5). The coastal area suitable for *Vibrio* infections in the past 5 years has increased at northern latitudes (40–70° N) by 50·6% compared with a 1980s baseline. Regionally, the area of coastline suitable for *Vibrio* spp has increased by 61·2% for the Baltics and 98·9% for the Atlantic Northeast. In 2019, for the second consecutive year, the entirety of the Baltic coastline was suitable for the transmission of *Vibrio* bacteria.

#### Indicator 1.3.2: vulnerability to mosquito-borne diseases—headline finding: following a sharp decline from 2010 to 2016, 2016–18 saw small up-ticks in national vulnerability to dengue outbreaks in four of six WHO regions; further data are required to establish a trend

As discussed, climate change is expected to facilitate the expansion of *Aedes* mosquito vectors that transmit dengue. Improvements in public health services might counteract these threats in the short-to-medium term; however, climate change will continue to make such efforts increasingly difficult and costly.^99^ This indicator tracks vulnerability to mosquito-borne disease by combining data from indicator 1.3.1 on vectorial capacity for the transmission of dengue with the core capacities of countries’ health-care systems, as outlined by WHO’s International Health Regulations, which have been shown to be effective predictors of protection against disease outbreak.^100^ The methods used here remain unchanged from previous reports and are described in full in the appendix (pp 33–35).^94,101^

From 2010 to 2016, vulnerability to mosquito-borne diseases declined substantially for the four most vulnerable WHO regions (the Western Pacific region, the African region, the South-East Asia region, and the region of the Americas), reflecting considerable improvements in their core health capacities. However, from 2016 to 2018, this trend began to halt, and then reversed, with further data required to confirm any long-term shift.

### Indicator 1.4: food security and undernutrition

Although the global food system still produces enough to feed a growing world population, poor management and distribution has resulted in a paucity of progress on the second sustainable development goal on hunger. The global number of undernourished people is projected to increase to more than 840 million in 2030.^102^

Climate change threatens to exacerbate this crisis further, with rising temperatures, climatic shocks, and ground level ozone affecting crop yields, and sea surface temperature and coral bleaching affecting marine food security.^3^ These effects will be experienced unequally, disproportionately impacting countries and populations already facing poverty and malnutrition, and exacerbating existing inequalities. The following two indicators monitor these changes, tracking the change in crop yield potential and sea surface temperature.

#### Indicator 1.4.1: terrestrial food security and undernutrition—headline finding: from 1981 to 2019, crop yield potential for maize, winter wheat, soybean, and rice has followed a consistently downward trend, with reductions relative to baseline of 5·6% for maize, 2·1% for winter wheat, 4·8% for soybean, and 1·8% for rice

For this indicator, crop yield potential was characterised by crop growth duration (the time taken to reach a target sum of accumulated temperatures) during the crop’s growing season. If this sum is reached early, then the crop matures too quickly, and yields are lower than average. Therefore, a reduction in crop growth duration represents a reduction in crop yield potential.^103^ This indicator tracks the change in crop growth duration for four key staple crops: maize, wheat, soybean, and rice at the individual country level and globally by use of a similar approach to previous reports, which has been improved to provide more accurate local estimates and now uses ERA5 data.^34^

**Figure 6:**
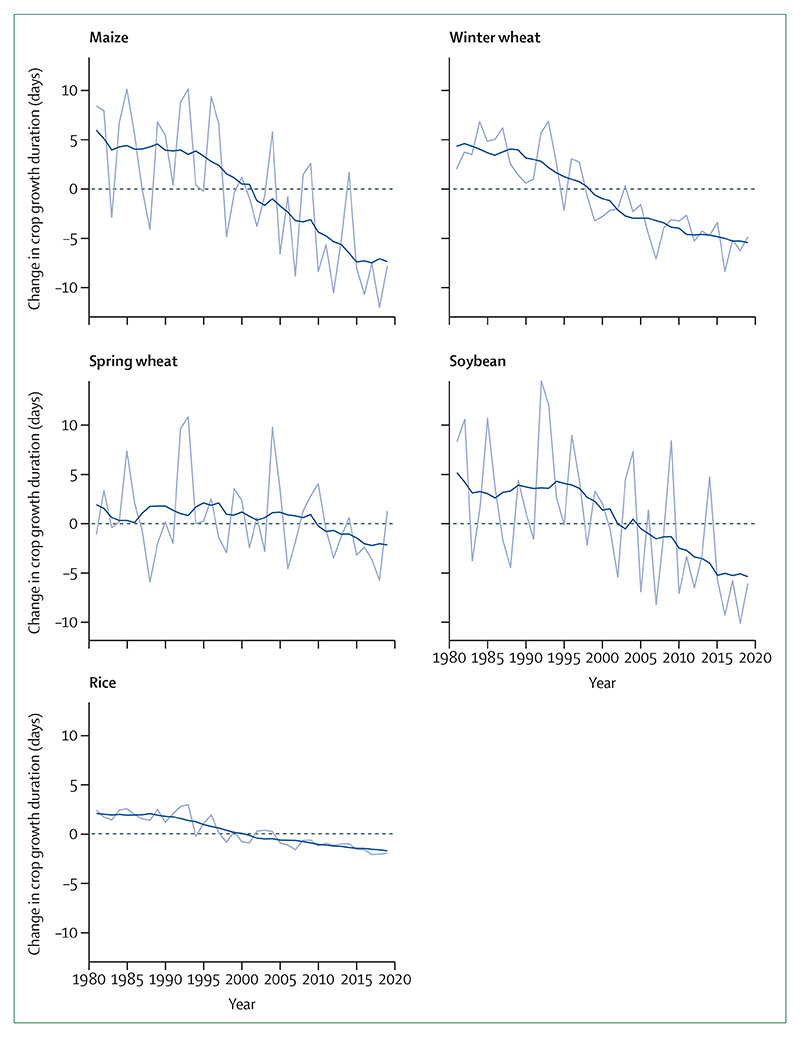
Change in crop growth duration relative to the 1981–2010 global average The grey line represents the annual global area-weighted change. The blue line represents the running mean over 11 years (5 years forward and 5 years backward). The dashed line represents the 1981–2010 baseline.

The yield potential of maize, winter wheat, soybean, and rice continues to decline globally and for most individual countries. This indicator shows that continuing to increase or even maintain global production is increasingly difficult because of the changing climate. In 2019, the reduction in crop growth duration relative to baseline was 5·6% (7·9 days) for maize, 2·1% (4·9 days) for winter wheat, 4·8% (6·1 days) for soybean, and 1·8% (2·0 days) for rice (figure 6). For maize, most countries in the world saw a decline in crop growth duration, with large areas of South Africa, the USA, and Europe having reductions in their crop growing seasons of more than 20 days—a reduction of more than 14% of the 1981–2010 global average crop duration. This reduction compounds the current negative impacts of weather and climate shocks, made more frequent and more extreme by climate change, that are hampering localised efforts to reduce undernutrition.

#### Indicator 1.4.2: marine food security and undernutrition—headline finding: average sea surface temperature rose in 46 of 64 investigated territorial waters between 2003–07 and 2015–19, presenting a risk to marine food security

A large proportion of the global population, especially in low-income and middle-income countries, is highly dependent on fish sources of protein.^104^ Additionally, omega-3 is important in the prevention of cardiovascular disease; worldwide, 1·4 million deaths due to cardiovascular disease in 2017 were attributed to diets low in seafood omega-3 fatty acids.^105^ Sea surface temperatures, rising as a consequence of climate change, impair marine fish capacity and capture through numerous mechanisms, including the bleaching of coral reefs and reduced oxygen content, putting populations at risk.^106^ This indicator tracks sea surface temperatures in the territorial waters of 64 countries located in 16 fishing areas of the Food and Agriculture Organization of the UN.^107–109^

Comparing the time periods 2003–07 and 2015–19, average sea surface temperatures increased in 46 of the 64 investigated areas, with a maximum increase of 0·87°C observed in the territorial waters of Ecuador. Farm-based fish consumption has increased consistently during the past four decades, with a corresponding decline in capture-based fish consumption, exacerbated in part by these evolving temperature trends.^106^ Between 1990 and 2017, diets low in seafood omega-3 increased by 4·7% at a global level, with more than 70% of countries seeing a rise in exposure to this risk factor, increasing the risk of mortality from cardiovascular disease.

### Indicator 1.5: migration, displacement, and rising sea levels

#### Headline finding: without intervention, between 145 million people and 565 million people living in coastal areas today will be exposed to, and affected by, rising sea levels in the future

Through its impacts on extreme weather events, land degradation, food and water security, and rising sea levels, climate change is influencing human migration, displacement, and relocation with consequences to human health.^110,111^ Left unabated, estimates for the average global sea level rise by the end of the century range from 1·0 –2·5 m, with projections rising as high as 5 m when taking into account regional and local coastal variation.^112,113^ This indicator, newly introduced for the 2020 report, tracks current population exposure to future rising sea levels and provides a measure of the extent to which health or wellbeing are considered in national policies that connect climate change and human mobility.

**Figure 7:**
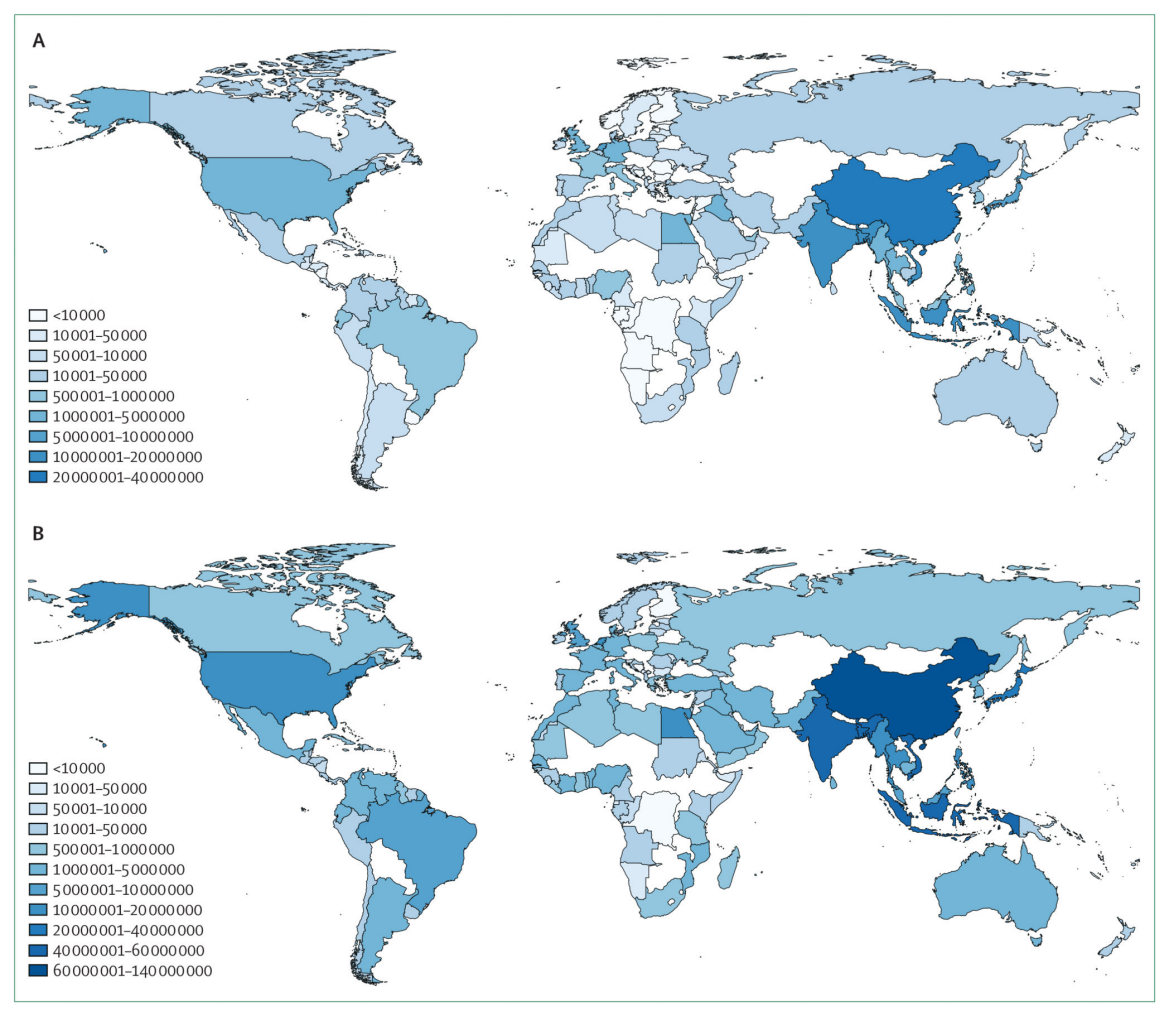
Number of people exposed to 1 m and 5 m of global average sea level rise by country (A) 1 m. (B) 5 m.

The exposure of populations to average global sea level rises of 1 m and 5 m was measured by use of a coastal digital elevation model and current population distribution data, with a full description of this new indicator outlined in the appendix (pp 51–57).^114,115^ Based on the population distributions of 2017, 145 million of the world’s population could be exposed to an average global sea level rise of 1 m, a value rising to 565 million people with an average sea level rise of 5 m (figure 7). A range of health impacts related to rising sea levels are likely to occur, with changes in water and soil quality and supply, livelihood security, disease vector ecology, flooding, and saltwater intrusion.^116,117^ The health consequences of these effects will depend on various factors, including the options of both in situ and migration adaptation.^118–120^ These effects could be moderated if countries begin to prepare. Considering preparation for climate change-related migration, national policies that connect climate change and migration were also assessed as part of this indicator. Up to Dec 31, 2019, there were 43 national policies across 37 countries that connected climate change and migration, and 40 of these policies across 35 countries explicitly referenced health or wellbeing. The policies commonly accepted that mobility could be domestic and international, although mention of immobility was sparse.

### Conclusion

The indicators that comprise section 1 of the 2020 report describe a warming world that is affecting human health both directly and indirectly and putting already vulnerable populations at a high risk. Metrics of exposure and vulnerability to extreme weather are complemented by trends of worsening global crop yield potential and increasing climate suitability for the transmission of infectious disease. Subsequent reports will continue to develop the methods and data underlying these indicators, with a particular focus on the creation of a new indicator on mental health, and the exploration of the gender dimensions of existing indicators.

Correlating climate change and mental health is challenging for several reasons, including local and global stigma and under-reporting, differences in health systems, and variations in cultural understandings of wellbeing. Partly because of this difficulty, the literature has focused on extremes of heat, with investigations reporting correlations between higher temperatures and heatwaves and the risk of violence or suicide. Proposed reasons for this association vary from the effects of disrupted sleep to short-term agitation.^121,122^ Stronger evidence outlines the links between extreme weather events and mental ill health, with emerging research describing the effect of a loss of access to the environment and ecosystem services.^123^

Taken as a whole, the data described in section 1 provide a compelling justification for an accelerated response to climate change. There are clear limits to adaptation, necessitating increasingly urgent interventions to reduce greenhouse gas emissions. How communities, governments, and health systems will be able to moderate the impacts of a changing climate is discussed in section 2 and section 3.

## Section 2: adaptation, planning, and resilience for health

With a growing understanding of the human costs of a warming climate, the need for adaptation measures to protect health is now more important than ever. The COVID-19 pandemic makes clear the challenges faced by health systems around the world resulting from large unexpected shifts in demand without sufficient adaptation or integration of health services across other sectors.^124^ As this public health crisis continues, and is compounded by climate-attributable risks, rapid and proactive interventions are crucial to prepare for, and build resilience to, both the health threats of climate change and of pandemics.^125^

Heavily determined by regional hazards and the underlying health needs of populations, the implementation of adaptation and resiliency measures requires localised planning and intervention. National adaptation priorities must take into account subnational capacities, inequalities, and the local distribution of vulnerable populations. As health adaptation interventions are being increasingly introduced, evidence of their success often remains mixed.^126^ Measuring the impact of these long-term interventions at the global scale presents particular challenges, and the indicators in this section aim to monitor the progress of health adaptation through the lens of the WHO Operational Framework for Building Climate Resilient Health Systems.^23^ The adaptation indicators look beyond the health system to focus on the following domains: planning and assessment (indicators 2.1.1–2.1.3), information systems (indicator 2.2), delivery and implementation (indicators 2.3.1–2.3.3), and spending (indicator 2.4). As is often the case in adaptation, several of these indicators rely on self-reported data on adaptation plans, assessments, and services, which also presents challenges. Where possible, efforts have been made to validate these data.

Numerous indicators in this section have been further developed for the 2020 report and one new indicator is presented. The data on national health adaptation planning and assessments (indicators 2.1.1 and 2.1.2) has been presented in greater detail and calculations of the effectiveness of air conditioning as an intervention (indicator 2.3.2) have been improved by use of more recent evidence. The definition of health-related adaptation spending (indicator 2.4) has been expanded to capture activities that are closely related to health in various non-health sectors. Importantly, a new indicator, focusing on the use of urban green spaces as an adaptive measure with numerous health benefits, has been introduced in this year’s report (indicator 2.3.3).

### Indicator 2.1: adaptation planning and assessment

Adaptation planning and risk management is essential across all levels of government, with national strategy and coordination linked to subnational and local implementation and delivery.^3^ In every case, risk assessments are an important first step of this process.

The following three indicators track adaptation plans and assessments at the national and city level by use of data from the WHO Health and Climate Change Survey and the CDP Annual Cities Survey.^127,128^ Information on the data and methods for each are presented in the appendix (pp 58–61). Data from the WHO survey have not been updated for this year, and hence further qualitative analysis has been done to investigate the barriers to adaptation.

#### Indicator 2.1.1: national adaptation plans for health—headline finding: 50% of countries surveyed have developed national health and climate change strategies or plans. However, funding remains a key barrier to implementation of these strategies, with 9% of countries reporting to have the funds to fully implement their plans

51 (50%) of 101 countries surveyed have developed national health and climate change strategies or plans. National governments have identified financing as one of the main barriers to the implementation of these plans.^28,128^ Of the 45 countries with plans and who reported on funding, only four (9%) reported having adequate national funding available to fully implement such strategies. This low proportion highlights the importance of access to international climate finance for governments from low-resource settings. Despite this importance, only 17 (49%) of 35 national health authorities from low-income and lower-middle-income countries reported having access to climate funds from bodies such as the Global Environment Facility, the Adaptation Fund, the Green Climate Fund, or other donors. The Green Climate Fund, which currently has not funded a single health sector project for the tenth year running, is now looking to align its programming to incorporate health and wellbeing co-benefits in light of, and in response to, COVID-19. Although not yet accredited to submit and implement projects, WHO became a Green Climate Fund readiness partner in 2020, giving WHO the ability to support countries in their efforts to develop health components of national adaptation plans and to strengthen health considerations related to climate change.

Another key barrier to the implementation of national health and climate strategies is a paucity of multisectoral collaboration within government. Progress on cooperation across sectors remains uneven, with 45 (45%) of 101 countries surveyed reporting the existence of a memorandum of understanding that outlines roles and responsibilities with respect to climate policy between the health sector and the water and sanitation sector. However, less than a third of the 101 countries had a similar cooperative agreement between the health sector and the agricultural (31 [31%]) or social service sectors (26 [26%]). Furthermore, only about a quarter of countries reported agreements between the health sector and the sectors for transport (25 [25%]), household energy (19 [19%]), or electricity generation (22 [22%]). These omissions represent an important missed opportunity to recognise the health implications of national climate policies and to promote activities that maximise health benefits, avoid negative health effects, and evaluate the associated health savings that might result.

#### Indicator 2.1.2: national assessments of climate change impacts, vulnerability, and adaptation for health—headline finding: 48 (48%) of 101 countries surveyed have assessed national vulnerability and adaptation for health, with further investment required to adequately fund these crucial components of health system resilience

Strengthening all aspects of a health system allows it to protect and promote the health of a population in the face of known and unexpected stressors and pressures. In the case of climate change, this strengthening requires a comprehensive assessment of current and projected risks and population vulnerability. This indicator focuses on vulnerability assessments at the national level and the barriers faced by national health-care systems.^128^

Similar to the scarcity of funding for health and climate change plans, vulnerability assessments for health are also under-resourced. Indeed, assessing vulnerability was among the top three adaptation priorities identified as being underfunded by national health authorities, alongside the strengthening of surveillance and early warning systems and broader research on health and climate change. This underfunding was reported to be particularly true for subnational assessments and for those designed to be particularly sensitive to the needs of vulnerable population groups.

#### Indicator 2.1.3: city-level climate change risk assessments—headline finding: in 2019, 605 (77%) of 789 global cities surveyed had either already completed or were currently undertaking climate change risk assessments, with 545 (67%) of 814 cities expecting climate change to seriously compromise their public health assets and services, a substantial increase from 2018

Cities are home to more than half of the world’s population, produce 80% of global gross domestic product (GDP), consume two thirds of the world’s energy, and represent a crucial component of the local adaptation response to climate change.^129^ As such, this indicator captures cities that have undertaken a climate change risk or vulnerability assessment and expectations on the vulnerability of their public health assets. First presented in the 2017 report of the *Lancet* Countdown and since improved to include further questions specific to public health, data for this indicator are sourced from the Carbon Disclosure Project’s 2019 survey of 789 global cities (a 33% increase in survey respondents from 2018).^127,130^

In 2019, 491 (62%) of 789 cities had completed an assessment of climate change risk or vulnerability, and a further 114 (28%) cities were either in the process of an assessment or will have completed one within the next 2 years. Although some selection bias probably exists, a growing number of risk assessments are being completed by cities in low-income countries (14 [64%] of 22 in 2019), highlighting the beginning of adaptation where adaptation is arguably most needed. The survey also revealed a core driving factor in these assessments—545 (67%) of 814 cities reported that their public health infrastructure would be seriously compromised by climate change.

### Indicator 2.2: climate information services for health

#### Headline finding: the number of countries reporting that their meteorological services provide climate information to the health sector has continued to grow, increasing from 70 to 86 countries during the past 12 months

The use of meteorological services in the health sector is an essential component of adaptation. This indicator tracks the collaboration between these two parts of government by use of data reported by national meteorological and hydrological services to the World Meteorological Organization. Further detail is provided in the appendix (pp 62–64).

A total of 86 national meteorological and hydrological services of member states of the World Meteorological Organization reported providing climate services to the health sector, an increase of 16 from the 2019 report of the *Lancet* Countdown.^28^ By WHO region, 19 of the countries reporting these climate services were from the African region, 16 were from the region of the Americas, seven were from the Eastern Mediterranean region, 23 were from the European region, eight were from the South-East Asia region, and 13 were from the Western Pacific region. Of the 86 positive respondents, 66 (77%) reported being highly engaged with their corresponding health service, alongside other sectors such as agriculture, water, and electricity generation. As detailed in indicator 2.1.1, multisector collaborations present governments with the opportunity to support an adaptation approach to the risks of climate change that is fully integrated.

### Indicator 2.3: adaptation delivery and implementation

#### Indicator 2.3.1: detection, preparedness, and response to health emergencies—headline finding: in preparation for a multi-hazard public health emergency, 109 countries have reported medium-to-high implementation of a national health emergency framework

The International Health Regulations are an instrument of international law designed to aid the global community in preventing and responding to potential public health emergencies.^101^ This indicator focuses on core capacity eight, which evaluates the degree to which countries have implemented a national health emergency framework by assessing levels of planning, management, and resource allocation.^101^ The national health emergency framework applies to all public health events and emergencies, air pollution, extreme temperatures, droughts, floods, and storms. The core capacities of the International Health Regulations are also important components of the response to infectious disease threats, with similar capacities and functions considered when assessing preparedness to a pandemic such as the COVID-19 pandemic.^131^ The results of this survey are provided in full in the appendix (pp 64–65).

In 2019, 166 (86%) of 194 WHO member states completed the assessment portion related to core capacity eight, 16 fewer than in 2018. Of these 166, 109 (66%) countries reported having medium-to-high degrees of implementation of multi-hazard preparedness and capacity, a 10% increase compared with 2018 data. The level of implementation varied by region. Medium-to-high levels were reported in 26 (90%) of 29 countries in the region of the Americas, 41 (87%) of 47 in the European region, 11 (85%) of 13 in the Western Pacific region, seven (64%) of 11 in the South-East Asia region, 12 (63%) of 19 in the Eastern Mediterranean region, and in only 12 (26%) of 47 countries in the African region. Despite these disparities, capacities have increased across all regions, and the global average increased from 59% in 2018 to 62% in 2019.

#### Indicator 2.3.2: air conditioning: benefits and harms—headline finding: between 2016 and 2018, the world’s air conditioning stock continued to rise, further contributing to climate change, air pollution, peak electricity demand, and urban heat islands, while also conferring protection against heat-related illness

Air conditioning represents one of numerous effective indoor cooling mechanisms for preventing heat-related illness and mortality.^132^ However, in 2018, air conditioning accounted for an enormous 8·5% of total global electricity consumption, contributing to, if sourced from fossil fuels, emissions of carbon dioxide (CO_2_) and fine particulate matter (PM_2·5_), and ground level ozone formation, with the potential to leak hydrofluorocarbons that act as powerful greenhouse gases. On hot days, air conditioning can be responsible for more than half of peak electricity demand locally, and emits waste heat that contributes to the urban heat island effect.^133,134^ Further research is needed to establish whether the overall harms of air conditioning outweigh the benefits. However, increased use of air conditioning in response to the warming climate could result in around 1000 additional deaths related to air pollution every summer in the eastern USA by 2050.^135^

International programmes and organisations, including Sustainable Energy for All, the Kigali Cooling Efficiency Program, and the International Energy Agency (IEA), are working to develop solutions to provide efficient indoor cooling that protect vulnerable populations against heat-related illness while minimising the health-associated harms. Such initiatives include designing buildings with improved insulation, energy efficiency measures, and improved ventilation, and increasing urban green space (detailed in indicator 2.3.3). Evidence suggests that simple electric fans with light water spraying could also be an effective stay-at-home measure against heatwaves in hot and humid regions during the COVID-19 pandemic.^136^

This indicator draws on data provided by the IEA and includes an improved calculation of the prevented fraction of deaths from air conditioning, making use of an updated meta-analysis that built on the previously available 2007 assessment of prognostic factors in heat-wave-related deaths, with full detail described in the appendix (pp 66–69).^132,137^

**Figure 8:**
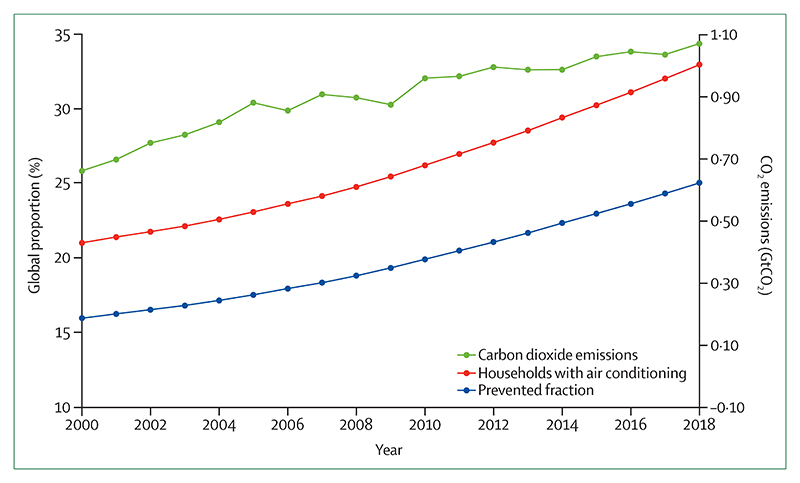
Frequency and effects of air conditioning Global proportion of households with air conditioning (red line), prevented fraction of heatwave-related mortality because of air conditioning (blue line), and CO_2_ emissions from air conditioning (green line), from 2000 to 2018. CO_2_=carbon dioxide. GtCO_2_=gigatonnes of carbon dioxide.

Between 2016 and 2018, the world’s air conditioning stock (residential and commercial) increased from 1·74 billion units to 1·90 billion units and the proportion of households with air conditioning increased from 31·1% to 33·0% (a 56·7% rise since 2000; figure 8). Correspondingly, the global prevented fraction of mortality related to heatwaves increased from 23·6% in 2016 to 25·0% in 2018. Global CO_2_ emissions from electricity consumption due to air conditioning increased from 1·04 GtCO_2_ in 2016 to 1·07 GtCO_2_ in 2018 (2% of total global emissions), highlighting the need for sustainable cooling methods in the face of a warming climate.

### Indicator 2.3.3: urban green space

#### Headline finding: urban green space is an important measure to reduce population exposure to heat; 9% of global urban centres had a very high or exceptionally high degree of greenness in 2019, and more than 156 million people were living in urban centres with concerningly low levels of urban green space

Access to urban green space provides benefits to human health by reducing exposure to air and noise pollution, relieving stress, providing a setting for social interaction and physical activity, and reducing all-cause mortality.^138,139^ In addition, green space sequesters carbon and provides local cooling that disrupts urban heat islands, benefiting both climate change mitigation and heat adaptation. As access to green space can often disproportionately benefit the most privileged in society, it is important to consider how green spaces are designed and distributed to ensure safety and equitable access.^140,141^

This indicator, new in the 2020 report, quantifies exposure to urban green space for 2019 in the 468 urban centres of more than 1 million inhabitants, as defined by the Global Human Settlement programme of the European Commission.^142,143^ Indicator 2.3.3 uses remote sensing of green vegetation through the satellite-based normalised difference vegetation index, which measures the reflectance signature of green plants in the visible red and near-infrared parts of the spectrum, providing an indication of the level of green coverage on the earth surface. The maximum normalised difference vegetation index for all seasons was used to define the average level of greenness of each urban area. A full description of the methodology can be found in the appendix (pp 70–72).

**Figure 9:**
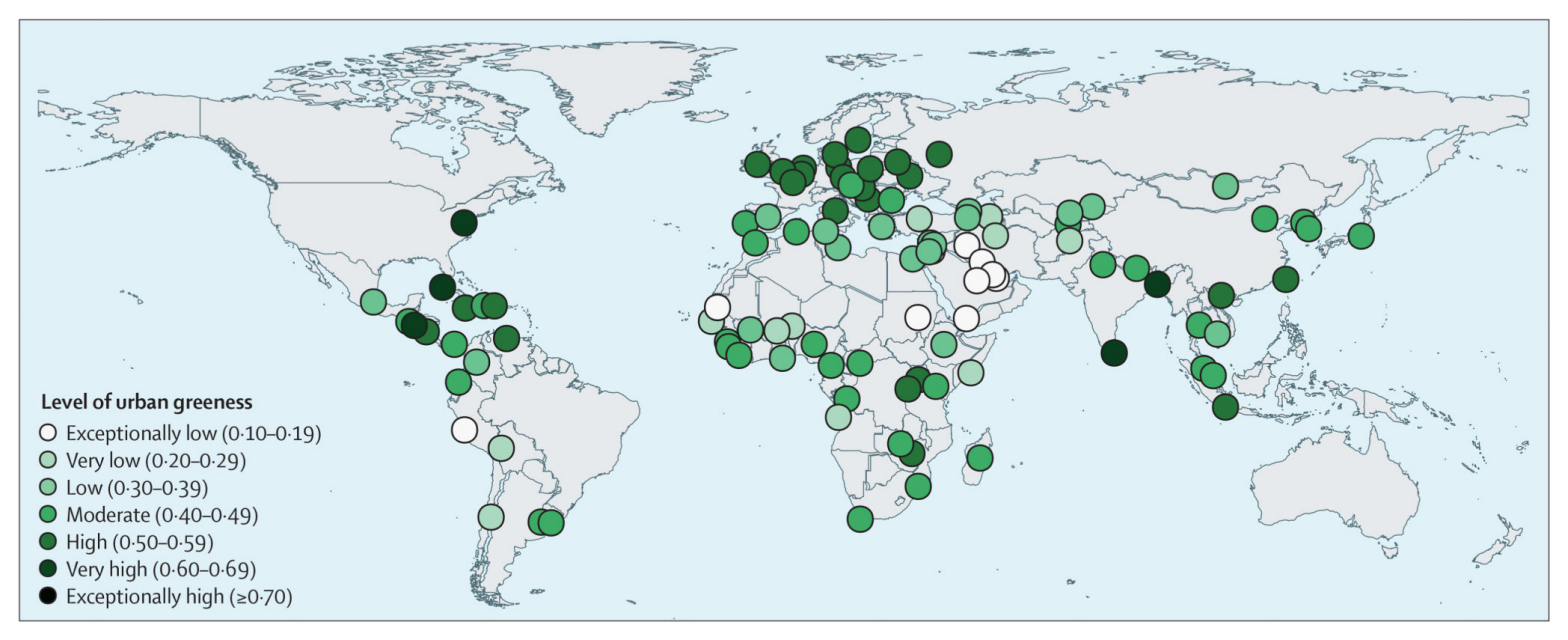
Urban greenness in capital cities with more than 1 million inhabitants in 2019 Levels of urban greenness were quantified on the basis of the mean, population-weighted normalised difference vegetation index, which is a standard, satellite-based measurment to estimate vegetation and is on a scale of –1·0 to 1·0.

In 2019, only 42 (9%) of 468 global urban centres had very high to exceptionally high levels of greenness, notably including five capital cities—Colombo (Sri Lanka), Washington, DC (USA), Dhaka (Bangladesh), San Salvador (El Salvador), and Havana (Cuba; figure 9). Concerningly, 49 (10%) urban centres, home to more than 156 million people and including 21 capital cities, were at the opposite end of the spectrum, with very low levels of urban green space.^38^

### Indicator 2.4: spending on adaptation for health and health-related activities

#### Headline finding: at $18·4 billion in 2018–19, global spending on health adaptation has increased to 5·3% of total spending on adaptation, while health-related spending has remained flat at approximately 28·4% of global adaptation spending from 2015 to 2019

As noted in the evaluation of national adaptation plans (indicator 2.1.1), inadequate financial resources pose the largest barrier to the implementation of adaptation measures. This indicator tracks spending on health and health-related adaptation within the Adaptation and Resilience to Climate Change dataset from the data research firm, kMatrix, which includes spend data from 191 countries.^144^ Health-specific spending is that which occurs within the formal health-care sector. For the 2020 report, an enhanced definition of health-related spending was developed through an expert review workshop to more accurately categorise spending. The definition captures adaptation spending within other sectors (ie, agriculture and forestry, the built environment, disaster preparedness, energy, transportation, waste, and water) that have a direct impact on one or more of the basic determinants of health (ie, food, water, air, or shelter) and have been linked to health outcomes in the published literature. A full description of the methodology can be found in the appendix (pp 73–75).

**Figure 10:**
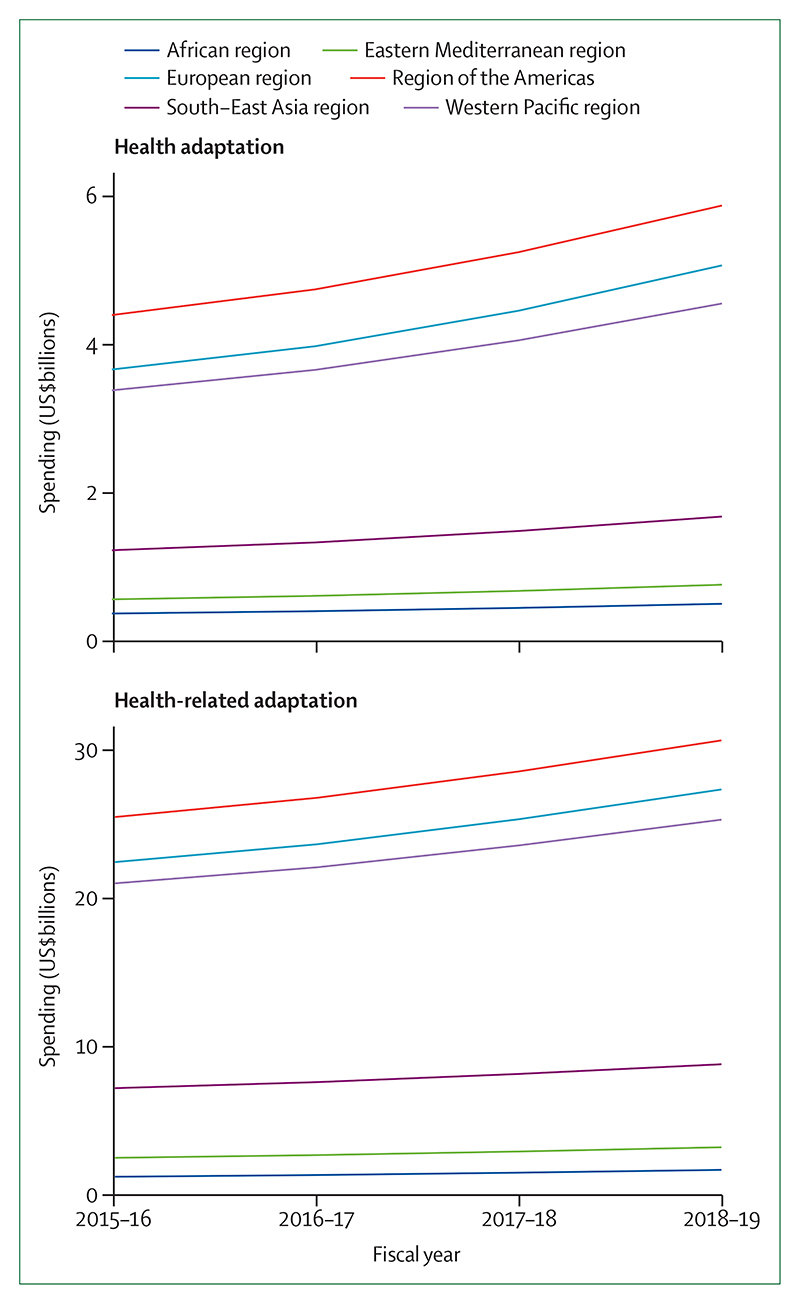
Adaptation and resilience to climate change spending by WHO Region

Spending on climate change adaptation within the health-care sector increased by 12·7% to $18·4 billion in 2018–19 compared with data from 2017–18 (figure 10). Spending on health adaptation made up 5·3% of all adaptation spending globally in 2018–19, a share higher than 5% for the first time. The wider measure of spending on health-related adaptation increased by 7·2% to $99·9 billion from 2017–18 to 2018–19; however, as a share of global adaptation spending, spending on health-related adaptation has remained more or less constant (28·4% in 2015–16 and 28·5% in 2018–19).

Grouped by WHO region, spending for health adaptation in 2018–19 varied from $0·48 per capita in the African region to $5·92 per capita in the region of the Americas, remaining less than $1·00 per capita in the South-East Asia region. Again, looking more broadly at spending on health-related adaptation, a wider variation, ranging from $2·63 per capita in the African region to $30·82 per capita for the region of the Americas, was evident.

### Conclusion

The indicators presented in this section continue to move in a positive direction, with growing recognition of the impacts of climate change within the health community. However, there is much more work to do, with a need to move from planning to implementation, and to better engage with other sectors of society in adaptation interventions (indicators 2.1.2, 2.1.2, and 2.2). The core capacity scores of the International Health Regulations show a need for support across many African and Eastern Mediterranean countries (indicator 2.3.1), requiring additional engagement and resources.

Global spending trends have shown promise in recent years for health and health-related adaptation (indicator 2.4); however, governments remain unable to fully implement their plans for national health adaptation (indicator 2.1.1). The findings here reiterate the need to strengthen underlying health systems and create multisectoral alignment to protect human health, particularly for the most vulnerable populations. COVID-19 has dramatically altered the pattern of healthcare demand, with health systems restructuring services overnight.^145^ Although the full impact of these changes is unclear, the rapid introduction of new online and telemedicine services brings many synergies with efforts to reduce the emissions of the health-care sector, and with those to increase the resilience of service delivery. As governments continue to respond to the public health and economic effects of the COVID-19 pandemic, it will be important to align these priorities and ensure that enhanced preparedness for future pandemics also confers an increased capacity to respond to climate change.

## Section 3: mitigation actions and health co-benefits

In 2018, greenhouse gas emissions rose to an unprecedented 51·8 gigatonnes of CO_2_ equivalent (GtCO_2_e; 55·3 GtCO_2_e including land use change), with fossil fuel emissions from transport, power generation, and industry accounting for 37·5 GtCO_2_e (72%).^146^ The vast majority of the growth in emissions, the economy, and the demand for energy occurred in low-income and middle-income countries, despite global economic headwinds.^147^

COVID-19 has had a profound effect on the global economy and on greenhouse gas emissions. Ongoing volatility makes the projections of any long-term effects challenging, although daily CO_2_ emissions were 17% lower in April, 2020, than they were in April, 2019, with some countries having reductions in emissions of up to 26%.^148^ Current estimates suggest that global emissions will fall by 8% in 2020 as a result of both the economic downturn and the restrictions to local and international travel.^21,148^ As efforts to revitalise the economy take effect, aligning such interventions with those necessary to mitigate climate change will allow governments to generate a synergistic response, improving public health in the short term and in the long term.

If carefully planned and implemented, these interventions will yield major health benefits, underlining the importance of a “health in all policies” approach.^149,150^ Highlighting this practice, the following section tracks efforts to mitigate climate change in the sectors most relevant to public health: power generation and air pollution (indicators 3.1.1–3.1.3 and 3.3); household energy and buildings (indicator 3.2); transport (indicator 3.4); diets and agriculture (indicators 3.5.1 and 3.5.2); and health care (indicator 3.6). New in the 2020 report are indicators of the national emissions from agricultural consumption (indicator 3.5.1) and the associated premature mortality from unhealthy and emissions-intensive diets (indicator 3.5.2). The methodologies of each of the existing indicators have also improved, particularly indicator 3.6, which, on the basis of feedback, has been revised to better estimate emissions from the health-care sector.

Importantly, this section must be interpreted with the understanding that enhanced ambition is urgently required, and that countries will need to increase the strength of their mitigation commitments within the Paris Agreement’s NDCs by a factor of three to limit warming to 2°C, and by a factor of five to limit warming to 1·5°C.^146^

### Indicator 3.1: energy system and health

#### Indicator 3.1.1: carbon intensity of the energy system—headline finding: the carbon intensity of the global primary energy supply has remained flat for the past three decades. Although in 2017 carbon intensity was at its lowest since 2006, it was still 0·4% higher than the levels in 1990

Because fossil fuel combustion in the energy system continues to be the biggest source of greenhouse gas emissions, mitigation in this area is key to meeting the commitments of the Paris Agreement. This indicator tracks the carbon intensity of the global energy system, expressed as the CO_2_ emitted per terajoule of the total primary energy supply, with methods and data described in the appendix (p 76).^151,152^

**Figure 11:**
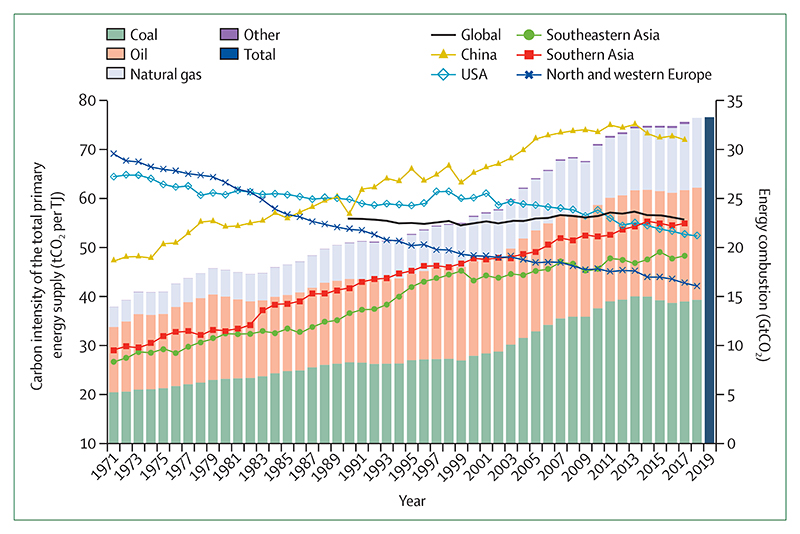
Carbon intensity of the total primary energy supply for selected regions and countries and global CO_2_ emissions by fuel type, 1971–2019 Carbon intensity trends are shown by a trend line (primary axis) and global CO_2_ emissions by stacked bars (secondary axis). This carbon intensity metric estimates the tCO_2_ for each unit of total primary energy supplied (tCO_2_ per TJ). For reference, the carbon intensity of fuels are as follows: coal, 95–100 tCO_2_ per TJ; oil, 70–75 tCO_2_ per TJ; and natural gas, 56 tCO_2_ per TJ. CO_2_=carbon dioxide. tCO_2_=tonnes of carbon dioxide.

The carbon intensity of the global energy system has barely altered in almost 30 years: in 2017, carbon intensity was 0·4% higher than that in 1990 (figure 11). Nevertheless, regional values have changed substantially. In 2018, carbon intensity was 12% lower in the USA and 20% lower in north and western Europe than the levels in 1990. China’s carbon intensity remained high at 72 tonnes of CO_2_ (tCO_2_) per TJ in 2017; however, China’s carbon intensity is decreasing, and in 2017 was 4% lower than its peak in 2013. Early statistics for 2020 suggest that global demand for all fossil fuels reduced in the first quarter because of COVID-19, and will continue to decline across the year, with resulting reductions in emissions.^21^ However, without targeted intervention, emissions could rebound, as they did following the global financial crisis of 2008–09, in which a 1·4% decrease in CO_2_ emissions in 2009 was offset by a 5·9% rise in 2010.^153^

#### Indicator 3.1.2: coal phase-out—headline finding: in 2018, global energy supply from coal was 1·2% higher than in 2017 and 74% higher than in 1990

Coal combustion continues to be the largest contributor to emissions from the energy sector and is a major contributor to premature mortality due to air pollution (indicator 3.3). The phase-out of coal-fired power is therefore an important first step in the mitigation of climate change. This indicator reports on progress towards a global phase-out, tracking the total primary energy supply from coal and coal’s share of total electricity generation, with methods provided in full in the appendix (pp 77–78).^154^

**Figure 12:**
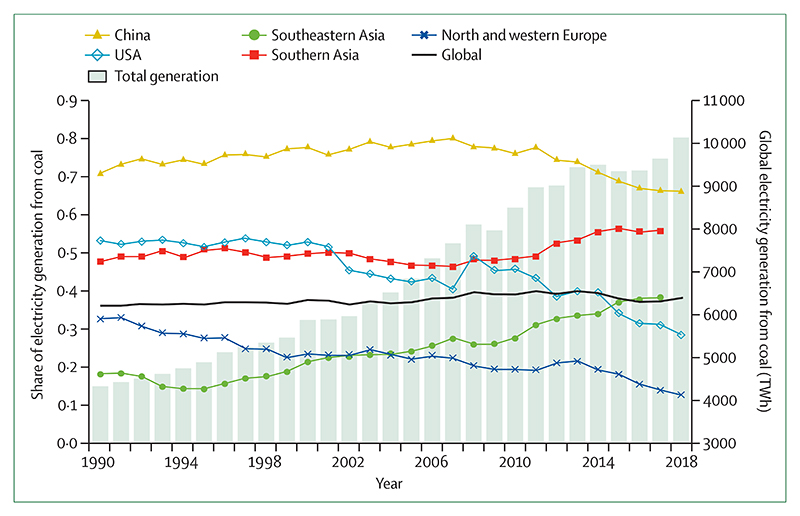
Share of electricity generation from coal in selected countries and regions, and global electricity generation from coal Regional shares of electricity generation from coal are shown by the trend lines (primary axis) and total electricity generation from coal by the bars (secondary axis). The global share of electricity generation from coal is shown with the thick black line. Data series are shown to at least 2017 and are extended to 2018 when data allow.

Global coal use for energy increased by 1·2% from 2017 to 2018, and, although remaining below the 2014 peak, use of coal for energy has risen by 74% overall since 1990. China, responsible for 52% of global coal consumption, has driven the rise, counteracting a 2017–18 reduction in coal use from other major economies such as Germany (–6·0%), the USA (–4·2%), Australia (–3·3%), and Japan (–1·2%). However, the share of electricity generation from coal in China is falling rapidly, decreasing from 80% in 2007 to 66% in 2018, as China moves to other power sources to meet the rising demand for electricity (figure 12). Likewise, northern and western Europe have seen falls in their share of electricity generation from coal, decreasing from 21% in 2013 to 13% in 2018.

As a result of the COVID-19 pandemic, cheap oil, and continued growth in renewables, global demand for coal fell by almost 8% in the first quarter of 2020 and is expected to remain at this level throughout the year.^21^ Additionally, Austria and Sweden closed their last coal-fired power plants in April, 2020, with other countries soon to follow.^155^

#### Indicator 3.1.3: zero-carbon emission electricity—headline finding: the average annual growth rate in power generation from wind and solar sources was 21% globally and 38% in China between 2010 and 2017, with all forms of low-carbon energy responsible for 33% of total electricity generation worldwide in 2017

Continued growth in renewable energy, particularly wind and solar sources, is key to replacing fossil fuels. This indicator tracks electricity generation and the share of total electricity generation from all low-carbon sources (nuclear and all renewables, including hydro) and renewables (wind and solar, excluding hydro and biomass). A full description of the methods and data can be found in the appendix (pp 79–80).^154^

Electricity generation from low-carbon sources continues to rise, growing by 10% from 2015 to 2017 to then account for 33% of total generation. In China during the same period, there was a 21% increase in low-carbon electricity generation, reaching 1800 TWh and 28% of all electricity produced.

Focusing on wind and solar energy reveals a similar picture, with global electricity generation from these sources increasing annually by 21% between 2010 and 2017. During the same period, China saw an even higher growth rate in power generation from wind and solar sources of approximately 38% per year due to a rapid increase in the use of solar energy, reaching 425 TWh in 2017. Despite this rise, China’s share of electricity generation from renewables remained relatively small at 6·5%, similar to India’s 5·0%. Contrary to the decline in demand for fossil fuels, the IEA expect the demand for renewable energy to increase in 2020 because of the lower operational costs of renewable sources compared with fossil fuel sources, but further policy support is necessary to continue this growth.^21,156^

### Indicator 3.2: clean household energy

#### Headline finding: primary reliance on healthy fuels and technology for household cooking has continued to rise, reaching 63% of the global population in 2018. However, total consumption of zero-emission energy for all household needs remained low at 26%

The use of unhealthy and unsustainable fuels and technologies for cooking, heating, and lighting in the home contributes both to greenhouse gas emissions and to dangerous concentrations of household air pollution.^157^ Primary reliance on such fuels and technologies for cooking is particularly problematic, resulting in recurrent direct exposure to high concentrations of poor quality air and causing more than 3·8 million premature deaths every year.^158^ This issue disproportionately affects women and children, who, in many cultural contexts, spend more time in the home than do men, are in charge of food preparation, and face threats to their safety associated with the gathering of cooking fuels.^157^

This indicator draws on national surveys collected by WHO across 194 countries and tracks the proportion of the population who use clean fuels and technologies for cooking, defined as those that have emission rate targets meeting WHO guidelines for air quality. This indicator also tracks the usage of zero-emission energy in the residential sector, measured as fuels with both zero greenhouse gas and zero particulate emissions at the point of use (mainly electricity and renewable heating) with data from the IEA.^154^

**Figure 13:**
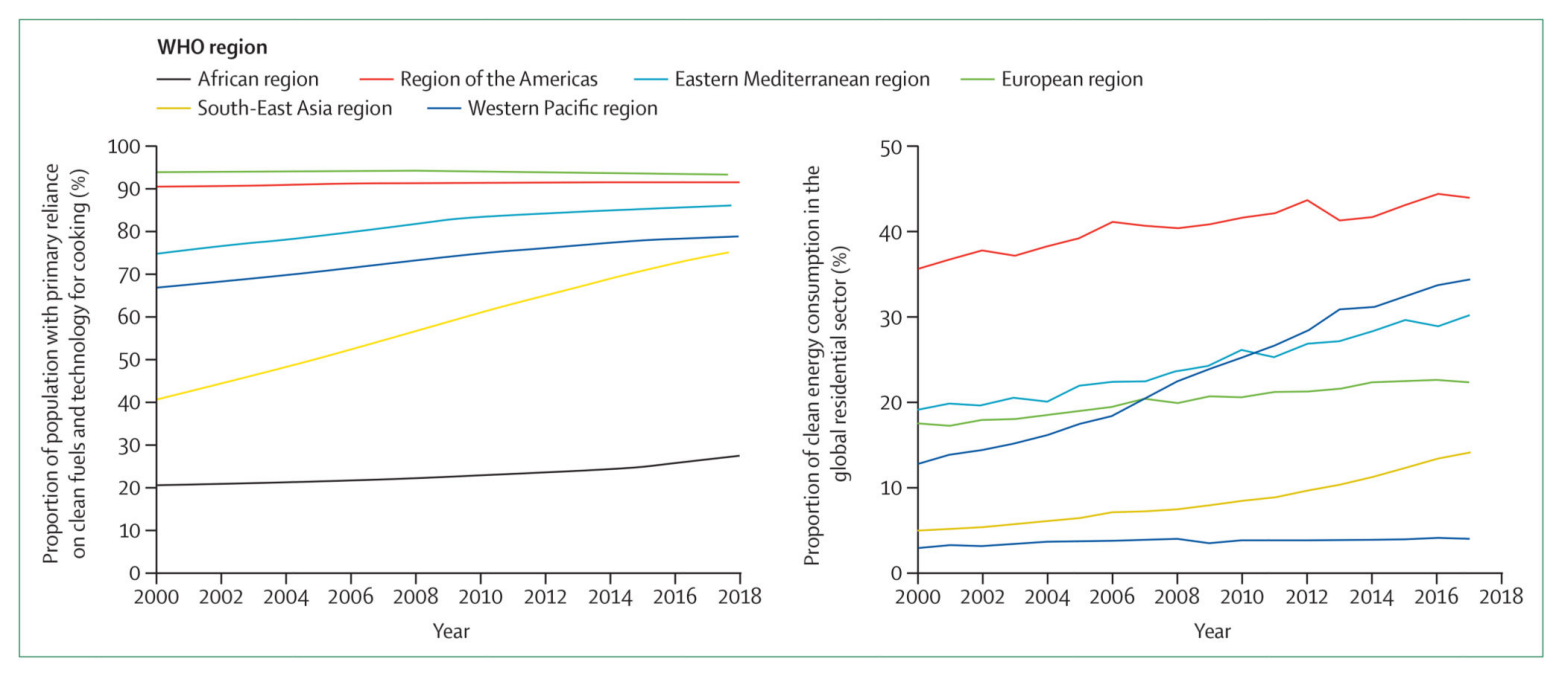
Household energy usage (A) Proportion of population with a primary reliance on clean fuels and technology for cooking by WHO region, 2000–18. (B) Proportion of clean energy at the point of consumption in the global residential sector, 2000–16. Proportion is measured as the zero-emission energy consumed (fuels with no emissions at the point of use) over the total energy consumed in the residential sector. Electricity comprised 75% of total clean energy use in 2016.

In 2018, 63% of the global population relied primarily on clean fuels and technologies for cooking, an increase of 26% since 2000. In China, this proportion increased from 43% in 2000 to 64% in 2018; in Vietnam, this proportion increased from 13% to 64% during the same period. However, little progress has been made in sub-Saharan Africa, where only 15% of households rely on clean fuels and technology for cooking. Importantly, overall use of zero-emission energy in the home (for all sources, including heating and lighting) remains low (26% globally in 2017) and has increased by only 2% per year since 2010 (figure 13).

**Figure 14:**
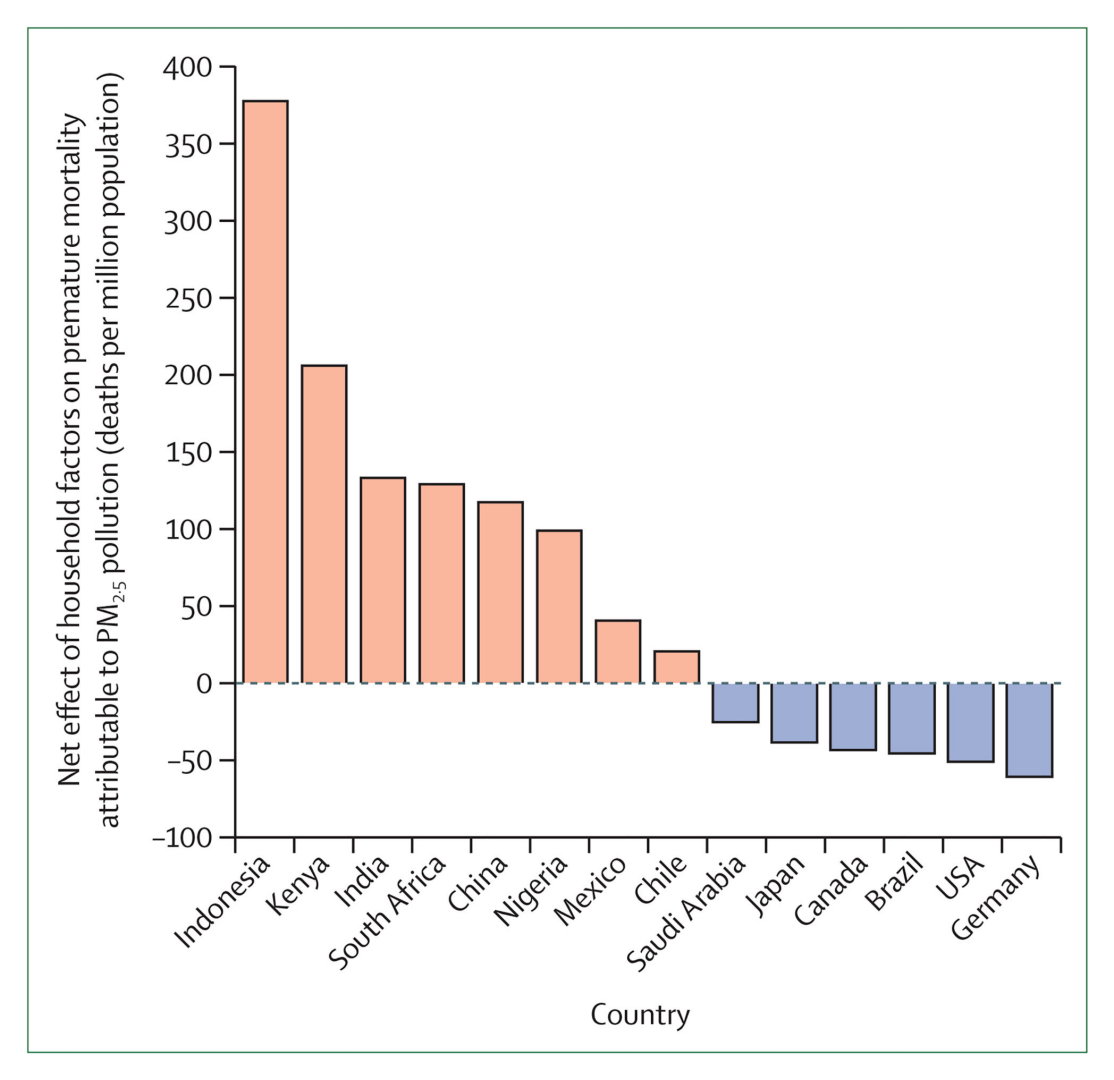
Estimated net effect of housing design and indoor fuel burning on premature mortality due to air pollution in 2018 PM_2·5_=fine particulate matter.

This section of the report is continuously evolving to understand the health co-benefits of mitigation efforts, and is now able to present findings from a new indicator under development that tracks mortality from household air pollution. Taking data on fuel and stove types used for cooking and the typical characteristics of housing ventilation, this indicator calculates household exposure to PM_2·5_, both from cooking and from air pollution infiltrating from outside. A full explanation of the methods is described in the appendix (pp 81–82). Here, the estimated effect of household factors on deaths attributable to PM_2·5_ pollution in 2018 are presented for selected countries (figure 14). In the middle-income countries assessed, the use of solid fuels for cooking, combined with poor housing ventilation, increased mortality from PM_2·5_ exposure. For other mostly high-income countries, housing design and extract ventilation prevented ambient air pollution from entering the home. Combined with the use of healthy cooking fuels, this prevention resulted in a net negative effect in total (both household and ambient) mortality attributable to PM_2·5_, showing a clear co-benefit of mitigation.

### Indicator 3.3: premature mortality from ambient air pollution by sector

#### Headline finding: premature deaths from ambient PM_2·5_ attributed to coal use are rapidly declining, falling from 440 000 deaths in 2015 to 390 000 deaths in 2018. However, total deaths from ambient PM_2·5_ have increased slightly during this time period, from 2·95 million deaths in 2015 to 3·01 million deaths in 2018, highlighting the need for accelerated intervention

Many of the leading contributors to global greenhouse gas emissions also contribute to ambient air pollution, disproportionately impacting on the health of communities with a low socioeconomic status.^159^ Indeed, some 91% of deaths from ambient air pollution occur in low-income and middle-income countries.^160^ This indicator tracks the source-attributable premature mortality from outdoor ambient air pollution. The methods remain unchanged and are described in the appendix (pp 83–84).^161,162^

**Figure 15:**
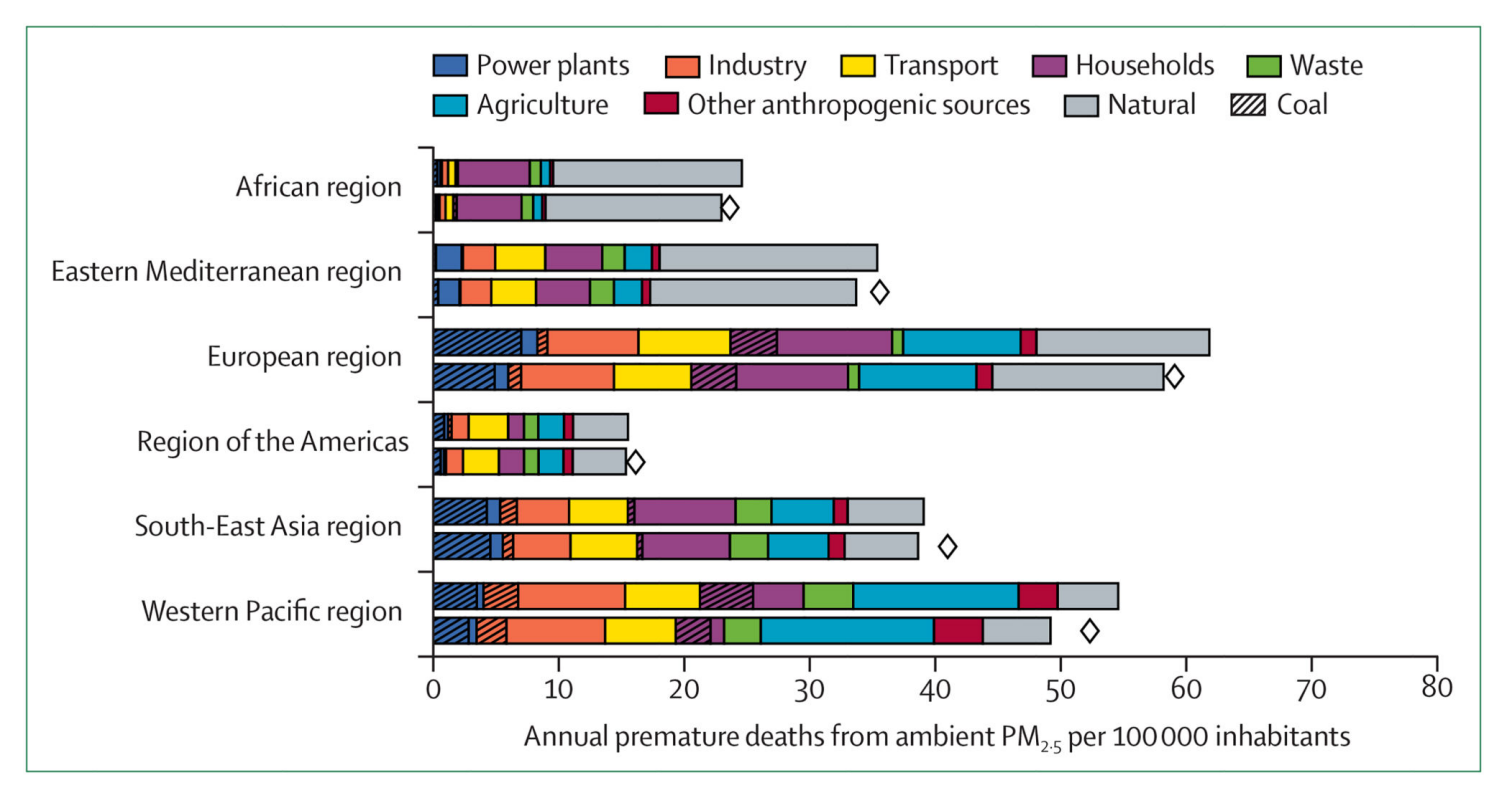
Premature deaths attributable to exposure to PM_2·5_ in 2015 and 2018 by key sources of pollution in WHO regions The coloured bars represent the attributable deaths if there were a constant 2015 population structure. The diamonds represent the total attributable deaths for 2018 when considering demographic changes. PM_2·5_=fine particulate matter.

Trends in mortality due to air pollution vary by world region. In Europe and China, mortality from air pollution decreased from 2015 to 2018 as a result of the implementation of technologies to control emissions and reductions in the use of raw coal in the power and residential sectors.^163^ The overall number of deaths attributable to ambient PM_2·5_ in 2018 was estimated at 3·01 million, a slight increase from the 2·95 million deaths in 2015. Nonetheless, the total and per-capita deaths attributable to coal combustion have decreased from roughly 440 000 deaths in 2015 to less than 390 000 death in 2018 (figure 15). Decreases were also seen in the contribution from biomass burning to ambient PM_2·5_ deaths (about 410 000 deaths in 2015, decreasing to 360 000 deaths in 2018) and were mostly due to the increasing access to cleaner household fuels (although, 2·6 billion people still rely on fuelwood combustion in the home).^164^

If measures to respond to the economic fallout from COVID-19 are aligned with the priorities of the Paris Agreement, transient reductions in air pollution following the sudden halt in economic activities and road transport could become more permanent, resulting in further improvements in health and air quality in 2020 and into the future.

### Indicator 3.4: sustainable and healthy transport

#### Headline finding: although fossil fuels continue to dominate the transport sector, the use of electricity for road transport rose by 18·1% from 2016 to 2017, and the global electric vehicle fleet increased to more than 5·1 million vehicles in 2018 (a rise of 2 million vehicles in only 12 months)

The transition to ultra-low emission vehicles is another essential component of mitigating climate change. In addition, policies that reduce overall vehicle use and increase walking and cycling will yield the greatest benefits in terms of reductions in greenhouse gas emissions and air pollution and the health advantages of increased physical activity.^165^ Well designed public transport and active travel infrastructure can also help to reduce inequality and improve mobility for those who otherwise have sparse travel options.^166^ For the 2020 report, global trends in fuel use for road transport were monitored, with methods and data available in the appendix (p 85).^167^

**Figure 16:**
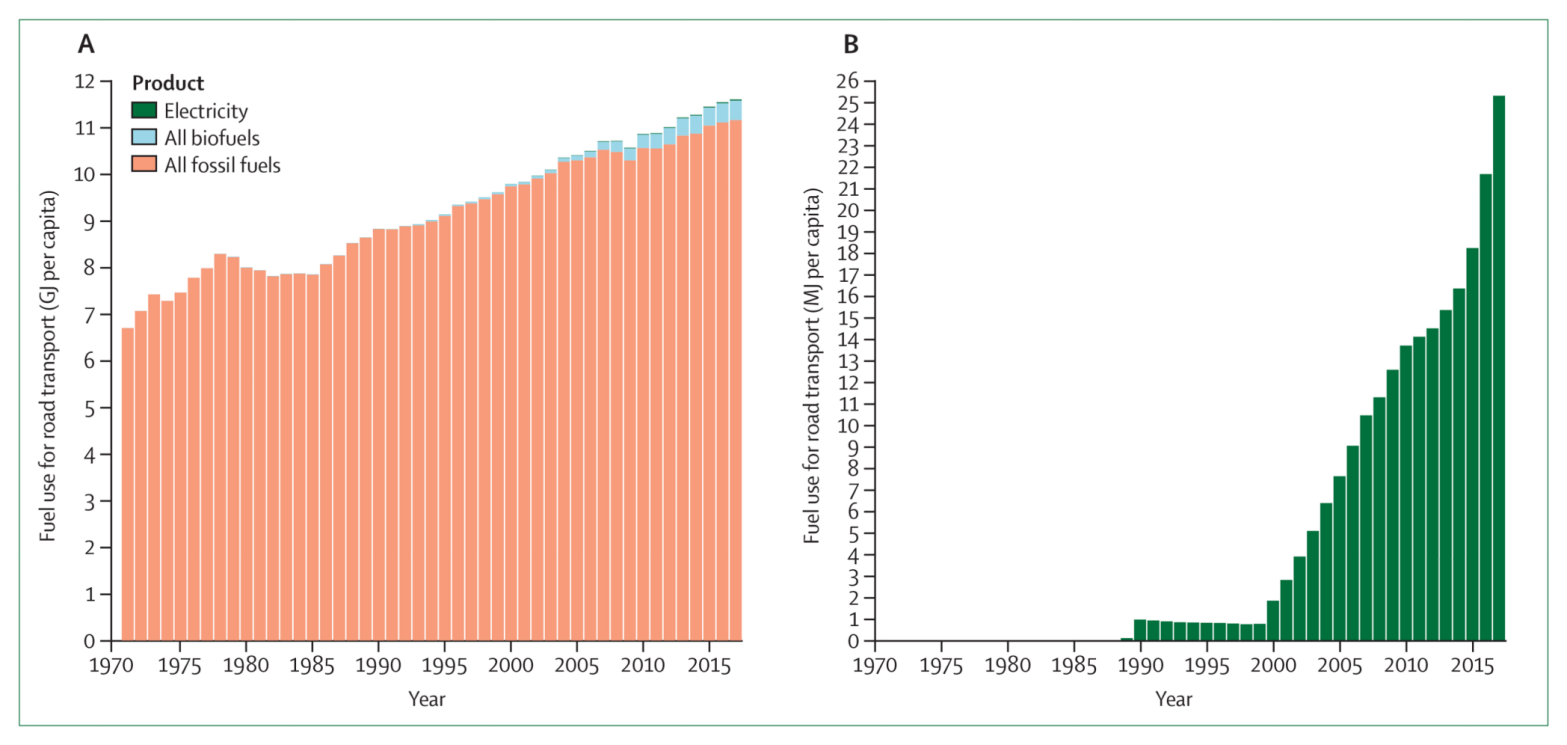
Per-capita fuel use for road transport (A) All fossil fuels, biofuels, and electricity. (B) Electricity only. Please note the varying scales in the y-axes.

Global per-capita use of fuel for road transport increased by 0·5% from 2016 to 2017, with the rate of growth slowing slightly compared with previous years (figure 16). Although fossil fuels continue to contribute to most total fuel use, the use of clean fuels is growing at a much faster pace. Between 2016 and 2017, total use of fossil fuels for transport increased by only 1·7%, whereas the use of electricity for road transport increased by 18·1%. From 2017 to 2018, the global electric vehicle fleet grew by an enormous 64·5%, rising to more than 5·1 million vehicles in 2018. In line with this rapid growth, there are now more than 5·2 million charging stations available for passenger vehicles and another 157 000 fast chargers available for buses worldwide.

### Indicator 3.5: food, agriculture, and health

#### Indicator 3.5.1: emissions from agricultural production and consumption—headline finding: ruminant livestock continue to dominate agriculture’s contribution to climate change and are responsible for 56% of total agricultural emissions and 93% of all livestock emissions globally. This proportion represents a 5·5% increase in the per-capita emissions from beef consumption between 2000 and 2017, which is particularly concerning given the sharp rise in population during this time period and the health impacts of excess red meat consumption

The food system is responsible for 20–30% of global greenhouse gas emissions, most of which originate from meat and dairy livestock.^168^ Improved for the 2020 report, agricultural emissions from countries’ production and consumption (adjusting for international trade) were tracked by use of data from the Food and Agriculture Organization of the United Nations, with a full description of methods and data provided in the appendix (pp 86–91).^169,170^ Although countries’ emissions are typically measured on a production basis, it is their consumption that generates the demand and results in diet-related health outcomes.

**Figure 17:**
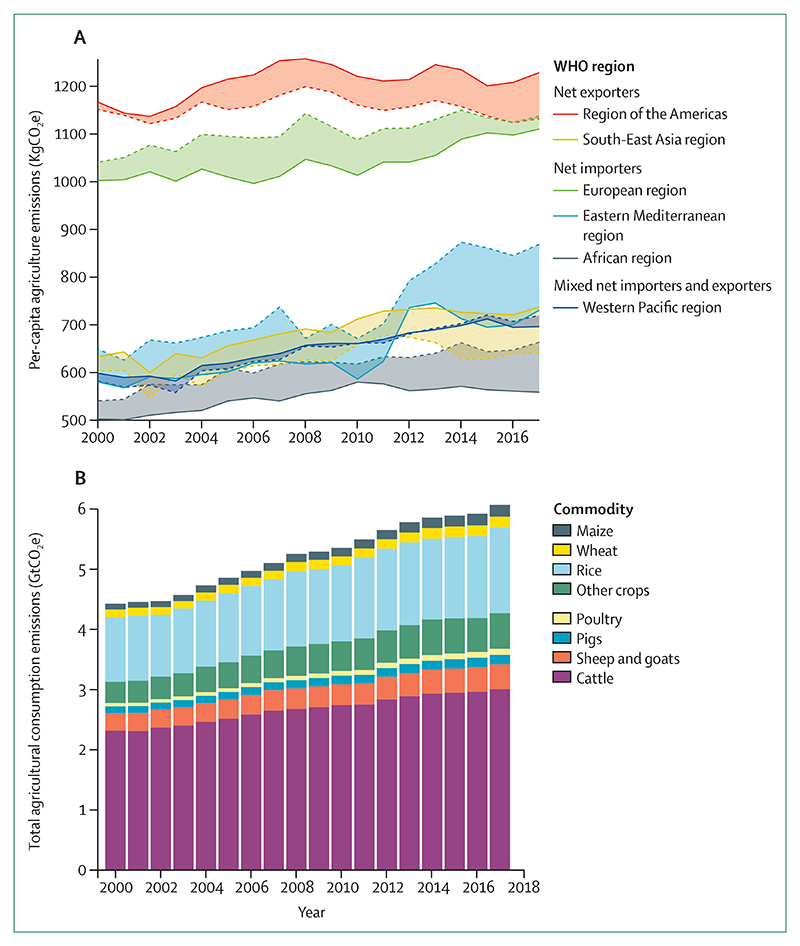
Agricultural production and consumption emissions, 2000–17 (A) Emissions by WHO region. (B) Global agricultural consumption emissions by commodity. Trade data from the Food and Agriculture Organization of the United Nations were used to calculate these numbers. Per-capita production is shown by the solid lines and per-capita consumption by the dotted lines. GtCO_2_e=gigatonnes of carbon dioxide equivalent. kgCO_2_e=kilograms of carbon dioxide equivalent.

Overall emissions from livestock production have increased by 16% since 2000 to more than 3·2 GtCO_2_e in 2017. Ruminants contribute to 93% of total livestock emissions, of which non-dairy cattle contribute 67%. Regarding emissions from consumption, products from the beef industry dominate, both in absolute and percapita terms (figure 17). Average emissions from beef consumption were 402 kgCO_2_e per person in 2017, compared with 380 kgCO_2_e per person in 2000.

Ultimately, effective mitigation will maximise human health while reducing food and agricultural emissions; however, no one diet is applicable everywhere and there are important nuances and variations to be considered across regions and countries. Excessive consumption of red meat brings considerable health consequences, and plant-based sources that are less emissions-intensive are important alternatives, particularly in Europe and the Americas where per-capita emissions are high. In other parts of the world, sustainable farming and agricultural practices are being implemented to meet the nutritional requirements of rapidly growing populations while also keeping emissions low.^171^

#### Indicator 3.5.2: diet and health co-benefits—headline finding: the global number of deaths due to excess red meat consumption rose to 990 000 deaths in 2017, a 72% increase since 1990

An unhealthy diet is one of the leading risk factors for premature death, both globally and in most regions.^105^ Combined with a range of food system-wide interventions, achieving dietary change consistent with the Paris Agreement and the sustainable development goals is possible by reducing reliance on red meat consumption and prioritising healthier alternatives, with various diets and choices available depending on the region, individual, and cultural context.^172,173^ New to the 2020 report, this indicator presents the change in deaths attributable to dietary risks by focusing on one particular area—the consumption of excess red meat. Here, this indicator links food consumption from the food balance sheets of the Food and Agriculture Organization of the United Nations with dietary and weight-related risk factors, with a full description of methods and data presented in the appendix (pp 91–97).^107,174^

**Figure 18:**
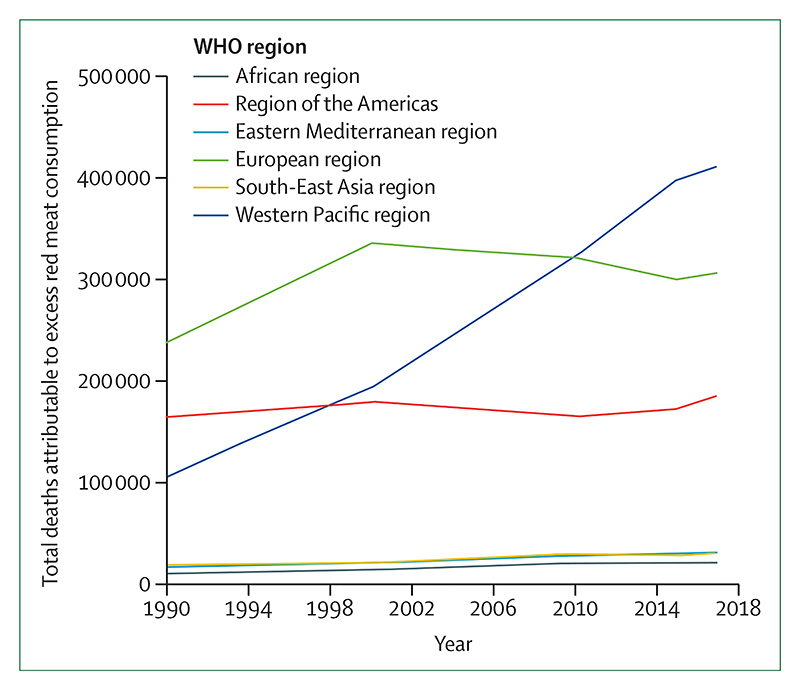
Deaths attributable to excess red meat consumption in 1990–2017 by WHO region

Globally, diet and weight-related risk factors have barely changed since 1990, accounting for 8·8 million deaths in 2017, representing 19% of total mortality. The regions with the largest proportion of diet-related deaths included the Eastern Mediterranean region (28%), the European region (25%), and the region of the Americas (22%). High red meat consumption was responsible for 990 000 deaths globally in 2017 (figure 18). The greatest contribution to this total came from the Western Pacific region, where red meat consumption was responsible for an estimated 411 500 deaths (3·3% of all deaths in this region). Although there has been an overall improvement in dietary risk factors in Europe, deaths attributable to red meat consumption still accounted for 3·4% of all deaths (306 800 deaths).

### Indicator 3.6: mitigation in the health-care sector

#### Headline finding: the health-care sector was responsible for approximately 4·6% of global greenhouse gas emissions in 2017, with substantial variations in per-capita emissions and health-care access and quality

Health care is among the most important sectors in managing the effects of climate change and, simultaneously, this sector has an important role in reducing its own carbon emissions (panel 4). Emissions from the global health-care sector were modelled by use of environmentally extended multiregion input-output (EE MRIO) models combined with data on health-care expenditure from WHO.^177–181^ Based on external review and feedback, the improvements in methodology included adjustments in the EE MRIO satellite accounts that reflect recent shifts in emissions intensities, particularly in the energy sector, with a full description of methods and additional analysis in the appendix (pp 98–99).

**Figure 19:**
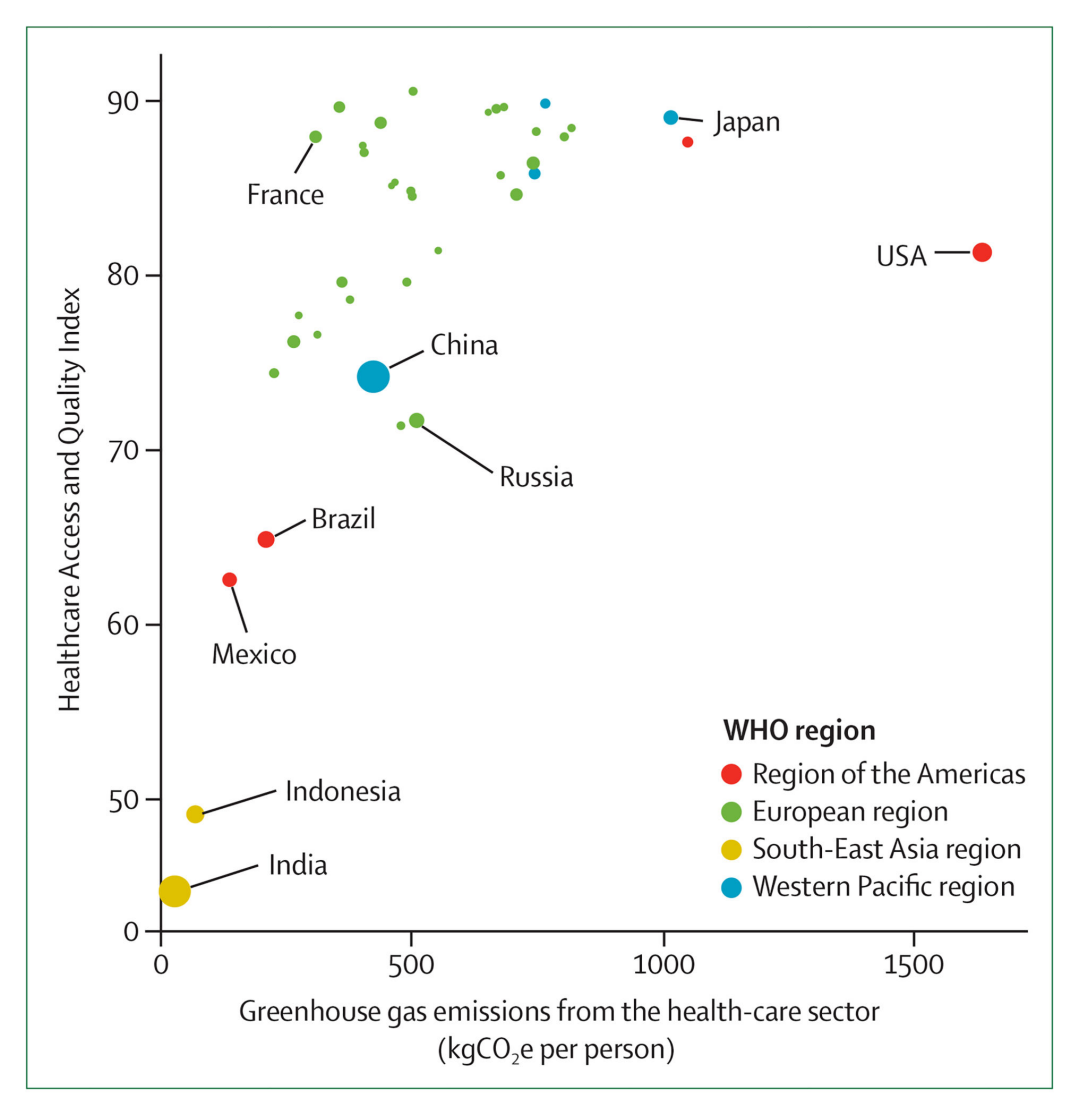
National per-capita greenhouse gas emissions from the healthcare sector against the Healthcare Access and Quality Index for 2015 kgCO_2_e=kilograms of carbon dioxide equivalent.

In 2017, the health-care sector contributed to approximately 4·6% of global greenhouse gas emissions, a rise of 6·1% from 2016. On a per-capita level, comparing emissions alone does not capture crucial differences in health outcomes among countries, including in access to care. Similarly, increases in emissions in a single country over time might reflect additional health-care spending that improves population health. Therefore, the 2015 per-capita greenhouse gas emissions from the health-care sector were plotted against the 2015 Healthcare Access and Quality (HAQ) Index (figure 19).^178^ There was a clear positive relationship between the two variables until emissions reached 400 kgCO_2_e per person. After this point, countries achieved very similar HAQ levels with vastly different emissions profiles. For example, France, Japan, and the USA had very high HAQ scores, and had per-capita emissions ranging from 350 kgCO_2_e for France, through to 1220 kgCO_2_e for Japan, and to 1720 kgCO_2_e for the USA, suggesting that much of health care can achieve high-quality patient outcomes with considerably reduced emissions.

### Conclusion

The trends during the past year show a concerning paucity of progress in numerous sectors, including a continued failure to reduce the carbon intensity of the global energy system, an increase in the use of coal-fired power, and a rise in agricultural emissions and premature deaths from excess red meat consumption. These issues are in part counteracted by the growth of renewable energy and improvements in low-carbon transport. Although the use of these greener options continues to rise at a pace, it is important to consider that they are starting from a low baseline.

In many cases, 2020 will probably be an inflection point for several of the indicators presented during the coming decade, with the direction of future trends yet to be seen. Ensuring that the recovery from the pandemic is synergistic with the long-term public health imperative of responding to climate change will be crucial in the coming months, years, and decades.

## Section 4: economics and finance

Section 1 described the emerging human symptoms of climate change, and sections 2 and 3 detailed efforts to adapt and mitigate against the worst of these effects. In turn, section 4 examines the financial and economic dimensions of the impacts of climate change and the efforts to respond.

The Intergovernmental Panel on Climate Change estimate that limiting warming to 1·5°C would require an annual investment in the energy system equivalent to around 2·5% of global GDP until 2035.^82^ Such investment would limit the cost of the damage from climate change (up to $4 trillion per year by 2100 if warming is limited to 2°C rather than to 3°C) and generate a range of other economic benefits (eg, the creation of new technologies and industries) and health benefits from avoiding the effects of climate change and current carbon-intensive activities. Once such factors are considered, the overall economic implications of limiting warming to 1·5°C are likely to be positive, particularly if responses in policy are accelerated as soon as possible to a level commensurate with the scale of the challenge. Estimates suggest that investment to “bend the curve” from the world’s current path and limit warming to a rise of 1·5°C by 2100 would generate a net global benefit of $264–610 trillion (3·1–7·2 times the size of the global economy in 2018).^12^

The global economy will look substantially different following the recovery from the COVID-19 pandemic. As governments around the world grapple with the challenge of restarting their economies, ensuring that these efforts are aligned with the response to climate change is important. If the enormous fiscal stimulus that will be required is directed away from high-carbon, and towards low-carbon, infrastructure and activities, an opportunity to permanently bend the curve presents itself. Metrics examining these core concepts are tracked in this report, allowing future data to reveal the long-term effect of COVID-19 on the low-carbon economy.

The nine indicators in this section fall into two broad domains. The first is the health and economic costs of climate change and its mitigation (indicators 4.1.1–4.1.4). This domain includes two new indicators for the 2020 report: the economics of heat-related mortality (indicator 4.1.2) and the potential reduction in earnings from heat-related loss of labour capacity (indicator 4.1.3). The second domain examines the economics of the transition to zero-carbon economies (indicators 4.2.1–4.2.5), which is fundamental to the improvement of human health and wellbeing. This domain also includes a new indicator (indicator 4.2.5) that merges three indicators presented in previous reports (ie, on fossil fuel subsidies, the strength and coverage of carbon prices, and carbon pricing revenues) to examine the net carbon prices in place around the world.

### Indicator 4.1: the health and economic costs of climate change and benefits from mitigation

#### Indicator 4.1.1: economic losses due to climate-related extreme events—headline finding: in 2019, economic losses from climate-related extreme events were nearly five times greater in low-income economies than in high-income economies. Just 4% of these losses were insured in low-income economies compared with 60% in high-income economies

Section 1 presented the evidence linking the impacts of climate change to human health and wellbeing. The loss of physical infrastructure (eg, agricultural land, homes, and health infrastructure) because of such events will further exacerbate these health effects. This indicator tracks the total annual economic losses (insured and uninsured) that result from climate-related extreme events. The methodology has changed from previous reports and is described in full in the appendix (pp 101–103).^182^

In 2019, 236 climate-related extreme events were recorded, with absolute economic losses totalling $132 billion. Although most of these losses occurred in high-income economies, when normalised by GDP, the value of total economic losses in low-income countries was nearly five times greater. In addition, although 60% of losses in high-income economies were insured, this proportion reduced to 3–5% for other income groups. When normalised by GDP, relative economic losses have been decreasing as the number of total extreme events has been increasing, suggesting that adaptation and prevention are reducing the impacts of these events.^183^

#### Indicator 4.1.2: costs of heat-related mortality—headline finding: the monetised value of global heat-related mortality increased from 0·23% of gross world product in 2000 to 0·37% in 2018. Europe was the worst affected in 2018, with costs equal to the average income of 11 million of its citizens and 1·2% of regional gross national income

As indicator 1.1.3 highlights, rising temperatures and extremes of heat are resulting in worsening morbidity and mortality for populations around the world. The 2020 report introduces a new indicator that considers the economic impact of this problem by tracking the monetised value of global heat-related mortality. To do so, this indicator uses the value of a statistical life estimated for the member countries of the Organisation for Economic Co-operation and Development (OECD) and the fixed ratio of the value of a statistical life to gross national income for non-OECD countries, applying these values to the heat-related mortality data from indicator 1.1.3.^184,185^ To address any distributional effects, and to more accurately capture the economic harm that climate change presents to low-income and middle-income countries, two indices have been calculated. The value of mortality is presented as a proportion of total gross national income (and gross world product) and as the average income per person this loss would be equivalent to in a given country and region. A full description of the methods, data, caveats, and further analysis are described in the appendix (pp 103–106).

**Figure 20:**
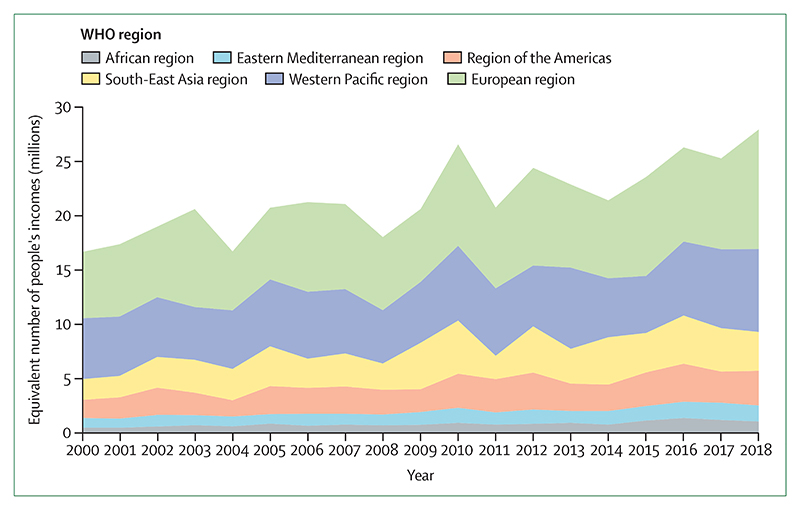
Cost of heat-related mortality represented as the number of people to whose income this value is equivalent, on average, for each WHO region

As global heat-related mortality increased from 2000 to 2018, so too did the monetised cost of these deaths. At a global level and represented as a proportion of gross world product, the cost increased from 0·23% in 2000 to 0·37% in 2018. Because of the high number of heat-related deaths, Europe was the worst affected WHO region, reaching a cost equivalent to the income of 11 million of its citizens in 2018 (led by Germany at 1·9 million; figure 20) and 1·2% of regional gross national income. Although in terms of the proportion of gross national income the value of mortality for the Western Pacific region (0·43%) and the South-East Asia region (0·19%) was comparatively low, the impact is more substantial when considered against the average income in these regions.

#### Indicator 4.1.3: loss of earnings from heat-related reduction in labour capacity—headline finding: rising temperatures make outdoor labour increasingly difficult, often resulting in public health and economic consequences for a wide range of occupations. By 2015, heat-related reduction in labour capacity resulted in earnings losses equivalent to an estimated 3·9–5·9% of GDP in the lower-middle-income countries tracked

Higher temperatures, driven by climate change, are affecting people’s ability to work (indicator 1.1.4). This new indicator considers the loss of earnings that could result from such reduced capacity, compounding the initial cause of ill health and impacting on wellbeing. The indicator adopts the outputs of indicator 1.1.4 for 25 countries, selected by the impact their workers experience and for geographical coverage, and combines these outputs with data on average earnings by country and sector held in the International Labor Organization databases.^40^ These estimates will be modified by various factors, ranging from whether or not sick leave was taken, the presence of workers’ sick pay rights, and the availability of shade. A full description of the methods and additional analysis is provided in the appendix (pp 107–120).

When taken as a share of GDP, low-income and lower-middle-income countries are the worst affected by heat-related reductions in labour capacity, with economic losses predominantly seen in agriculture, despite this sector being on average the lowest paid of the sectors considered. By 2015, averaged estimated losses in earnings reached the equivalent of 3·9–5·9% of GDP for the lower-middle-income countries tracked, including Indonesia, India, and Cambodia, and between 0·6–1·0% for the upper-middle-income countries tracked, including China, Brazil, and Mexico.

#### Indicator 4.1.4: costs of the health impacts of air pollution—headline finding: across Europe, ambient PM_2·5_ pollution from human activity reduced between 2015 and 2018. If held constant, this improvement alone would lead to an annual average reduction in years of life lost to the current population worth $8·8 billion

As described in indicator 3.3, global mortality due to ambient PM_2·5_ pollution has risen from around 2·95 million deaths in 2015 to 3·01 million deaths in 2018. However, because of improvements in air quality, including the closure of coal power stations, premature mortality due to air pollution in Europe has decreased during the same period. This indicator captures the cost of that change in the EU by placing an economic value on the years of life lost that result from exposure to PM_2·5_ from anthropogenic sources, with the methods and data described in full in the appendix (pp 121–122).^186^

**Figure 21:**
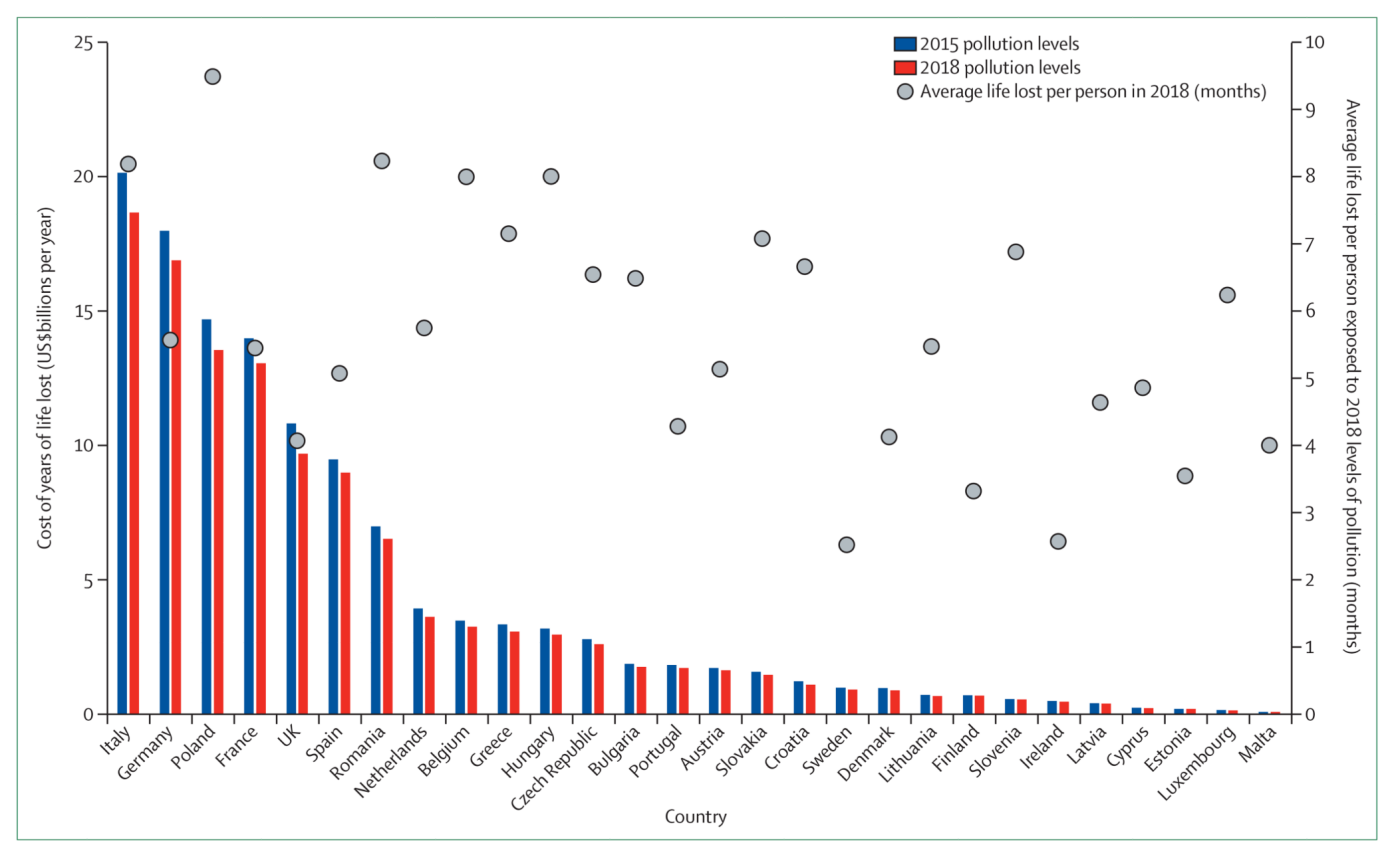
Annual cost of years of life lost and average months of life lost per person due to anthropogenic PM_2·5_ exposure PM_2·5_=fine particulate matter.

If the population of the EU in 2015 were to be exposed to anthropogenic PM_2·5_ emissions at 2018 levels instead of those present in 2015 consistently during the course of their lives, the total average economic value of the reduction in years of life lost would be around $8·8 billion (€9·85 billion) every year. Despite this, 2018 PM_2·5_ levels are still damaging to the cardiovascular and respiratory systems, and the total average cost to the current population would still be $116 billion (€129 billion) per year. Based on the levels of air pollution in 2018, the average life lost per person in the EU is 5·7 months, but this loss of life is estimated at more than 8 months per person for individuals in Poland, Romania, Hungary, Italy, and Belgium (figure 21).

### Indicator 4.2: the economics of the transition to zero-carbon economies

#### Indicator 4.2.1: investment in new coal capacity—headline finding: largely driven by China, investment in new coal capacity has been declining since 2011 and decreased by 6% between 2018 and 2019. Despite this reduction, global coal capacity continues to increase, with fewer retirements than there were additions of coal plants for every year tracked

As identified in section 3, phasing out coal is essential, not only for the mitigation of climate change, but also for the reduction of premature mortality due to air pollution. Taking data from the IEA, this indicator looks at future coal use, tracking investment in new coal-fired power generation. The data represent ongoing capital spending, with investment in a new coal plant spread evenly from the year construction begins to the year the plant becomes operational.^187^ For the 2020 report, data are presented for key countries and regions alongside the global trend. Further details on the methods and data can be found in the appendix (p 123).

**Figure 22:**
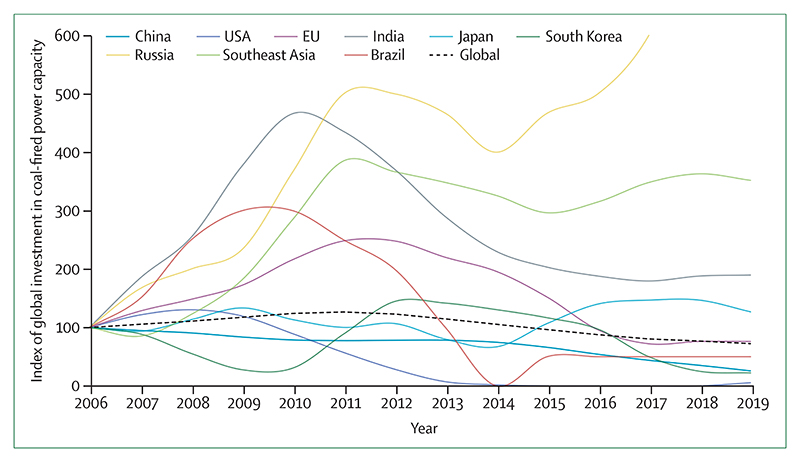
Annual investment in coal-fired capacity, 2006–19 An index score of 100 corresponds to 2006 levels of capacity.

Following the trend since 2011, global investment in coal-fired power decreased by a further 6% between 2018 and 2019 (figure 22). With a 27% reduction in investments during these 2 years, China has been driving this decline. Final investment decisions (the point at which the project’s future development is approved) have reached their lowest point in 40 years and, driven by declining investment in Asia, in part as a result of COVID-19, a further 11% reduction in investment is forecast for 2020. However, despite a substantial decline in actual investment, there were more final investment decisions in China in 2019 than in 2018, and, with the approval of 8 GW of new capacity, the number of final investment decisions had reached 2019 levels by March, 2020. Additionally, with fewer retirements than there were additions of coal plants in 2019 (and in every year presented), there was an overall increase in global coal capacity.

#### Indicator 4.2.2: investments in zero-carbon energy and energy efficiency—headline finding: progress towards zero-carbon energy has stalled; investments in zero-carbon energy and energy efficiency have not increased since 2016 and are a long way from doubling by 2030, which is required to be consistent with the Paris Agreement

This indicator monitors annual global investment in zero-carbon energy, energy efficiency, electricity networks, and in all fossil fuels, complementing and providing a wider context to indicator 4.2.1. Data are sourced from the IEA and the methodology remains the same as that in the 2019 report of the *Lancet* Countdown, with hydropower now considered separately and all values presented in US$2019.^187^

**Figure 23:**
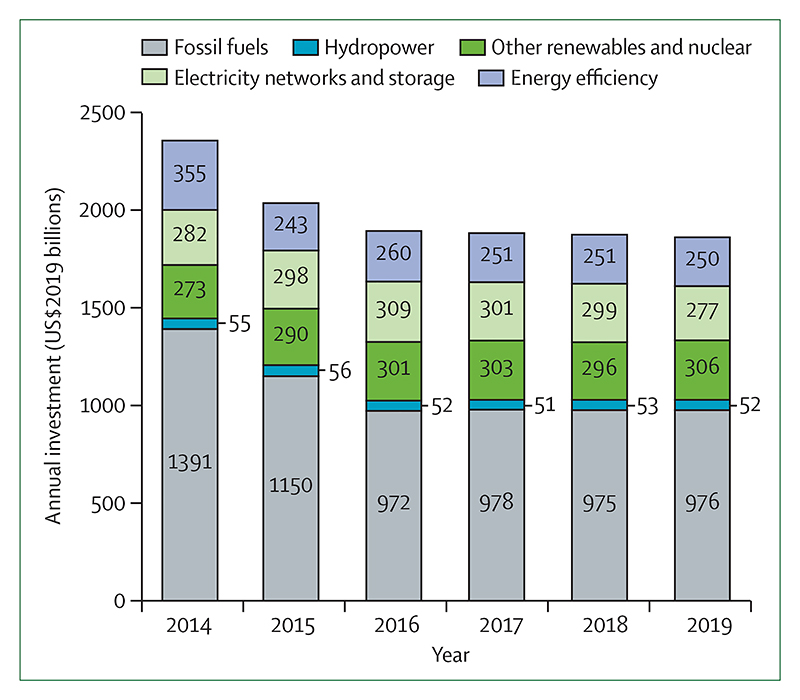
Annual investment in energy supply and efficiency

Since 2016, investment in global energy supply and efficiency has remained stable at just less than $1·9 trillion, with fossil fuel supply consistently accounting for around half this value and all renewables and energy efficiency combined maintaining a share of 32% (figure 23). For a pathway consistent with 1·5°C of warming this century, annual investments must increase to $4·3 trillion by 2030, with investment in renewable electricity, electricity networks and storage, and energy efficiency accounting for at least half this value.^188^

As a result of the COVID-19 pandemic, short-term disruption and long-term reassessments of probable returns mean that total energy investment is estimated to decrease by 20% in 2020 (the largest fall ever recorded), with investment in oil and gas supply to be reduced by a third. Investment in renewables is likely to fare better than is investment in fossil fuel capacity, with investment in zero-carbon energy (ie, nuclear, hydropower, and other renewables) and energy efficiency projected to increase from 32% to 37% in 2020 because of falling investments in fossil fuels.^187^ Stimulus plans focused on boosting energy efficiency and renewable energy will be essential to ensure that the power generation system is on track to meet the sustainable development goals and the goals of the Paris Agreement.^156^

#### *Indicator 4.2.3:* employment in low-carbon and high-carbon industries*—headline finding: renewable energy provided 11·5 million jobs in 2019, a 4·5% rise from 2018. Although still employing more people overall than the renewable energy industry, employment in fossil fuel extraction declined by 3% from 2018 to 2019*

There is mounting evidence that employees in some fossil fuel extractive industries, particularly those in coal mining, and populations living in close proximity to these industries, have a high incidence of certain illnesses, such as chronic respiratory diseases, cancers, and congenital anomalies.^189,190^ Combined with increased job certainty, a managed transition of employment opportunities away from fossil fuel-related industries and towards low-carbon industries will result in the improved occupational health of employees within the energy sector. This indicator tracks global direct employment in fossil fuel extraction industries (ie, coal mining, and oil and gas exploration and production) and direct and indirect (supply chain) employment in renewable energy for the most recent year available, with a full description of the methods and data available in the appendix (pp 125–126).^191–193^

Globally, around 11·5 million people were employed directly or indirectly by the renewable energy industry in 2019, representing an increase of 4·5% from 2018. The solar photovoltaic sector provided over a third of these jobs, with employment also rising in wind, bioenergy, and other technologies. Fossil fuel extraction industries continue to employ more people globally than do all renewable energy industries, although the number of jobs in 2019 (12·7 million) was slightly lower than the number in 2018 (13·1 million).

As the demand for fossil fuels declines, planned efforts, including retraining and job placements, are important to ensure the ongoing employment of those currently working in fossil fuel extraction industries. The same will be true as part of the response to COVID-19, with structured retraining and deployment programmes for renewable energy potentially forming an important component of a recovery plan. Indeed, the IEA estimates that such a strategy, which accelerates the deployment of low-carbon electricity sources, expands access to electricity grids and energy efficiency, and delivers cleaner transport, would create an additional 9 million jobs per year globally during the next 3 years.^156^

#### Indicator 4.2.4: funds divested from fossil fuels—headline finding: the global value of new funds committed to fossil fuel divestment in 2019 was $4·01 trillion, of which health institutions accounted for around $19 million. From 2008 to 2019, there was a cumulative sum of $11·51 trillion divested from fossil fuels, with health institutions accounting for $42 billion

By encouraging investors to reduce their financial interests in the fossil fuel industry, divestment efforts both remove the social licence to operate and guard against the risk of losses due to stranded assets in a world in which demand for fossil fuels rapidly decreases.^194,195^ This indicator tracks the total global value of funds divested from fossil fuels and the value of divested funds coming from health institutions by use of data provided by 350.org, with annual data and full methodology described in the appendix (pp 126–127).^196^

**Figure 24:**
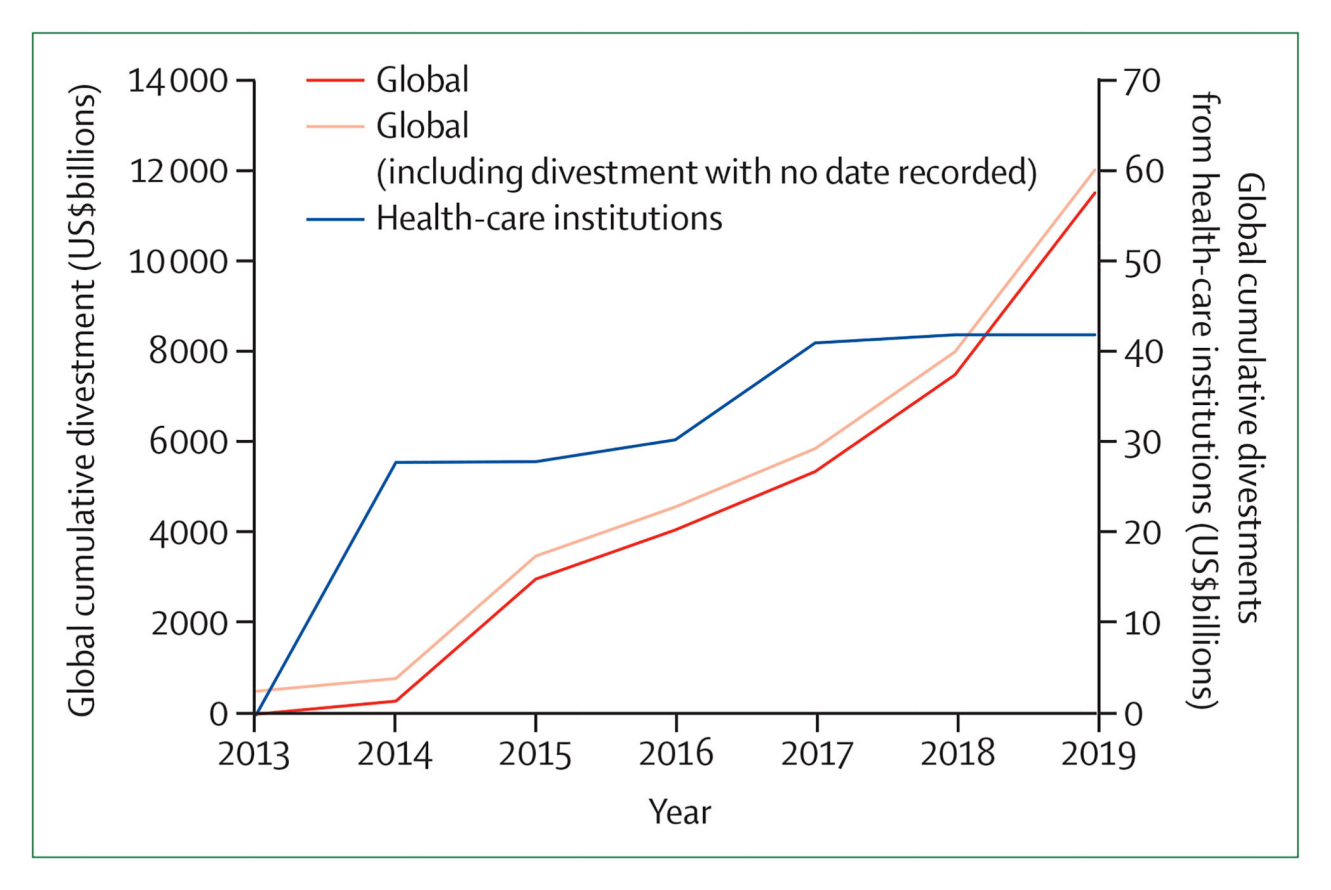
Cumulative divestment globally and in health-care institutions

From 2008 to the end of 2019, 1157 organisations, with cumulative assets worth at least $11·51 trillion, have committed to fossil fuel divestment (figure 24). Of these organisations, only 23 are health institutions, including the World Medical Association, the British Medical Association, the Canadian Medical Association, the UK Faculty of Public Health, the Royal College of General Practitioners, the Royal Australasian College of Physicians, Gundersen Health System, the Berlin Doctors Pension Fund, and the Royal College of Emergency Medicine, with total assets of approximately $42 billion. The annual value of new funds committed to divesting increased from $2·14 trillion in 2018 to $4·01 trillion in 2019. However, divestment from health institutions has decreased from $867 million in 2018 to $19 million in 2019, owed mainly to divestment from particularly large institutions in previous years.

#### Indicator 4.2.5: net value of fossil fuel subsidies and carbon prices—headline finding: 58 of the 75 countries reviewed were operating with a net negative carbon price in 2017. The resulting net loss of revenue was, in many cases, equivalent to substantial proportions of the national health budget

Placing a price on greenhouse gas emissions provides an incentive to drive the transition towards a low-carbon economy.^197,198^ This strategy also allows for a closer reflection of the true cost of emissions-intensive practices, particularly fossil fuel use, capturing some of the negative externalities resulting from their impact on health. However, not all countries explicitly set carbon prices, and, in some cases, the strength of any carbon price might be undermined by the opposing influence of subsidies on fossil fuel production and consumption.^199,200^

Indicator 4.2.5 has been created for the 2020 report by combining previous indicators on fossil fuel subsidies and carbon pricing. This indicator calculates net, economy-wide average carbon prices and associated net carbon revenue to government. The calculations are based on the value of overall fossil fuel subsidies, the revenue from carbon pricing mechanisms, and the total CO_2_ emissions of the economy. Data on fossil fuel subsidies are calculated on the basis of analysis from the IEA and OECD.^201,202^ Together, these sources cover 75 countries and account for around 92% of global CO_2_ emissions. Carbon prices and revenues are derived from data in the World Bank Carbon Pricing Dashboard and include international, national, and subnational mechanisms within countries, 38 of which overlap with those covered by subsidy data and thus form part of this analysis. A full description of the methodology, other data sources, and the methods for integrating these sources, can be found in the appendix (pp 129–137).

**Figure 25:**
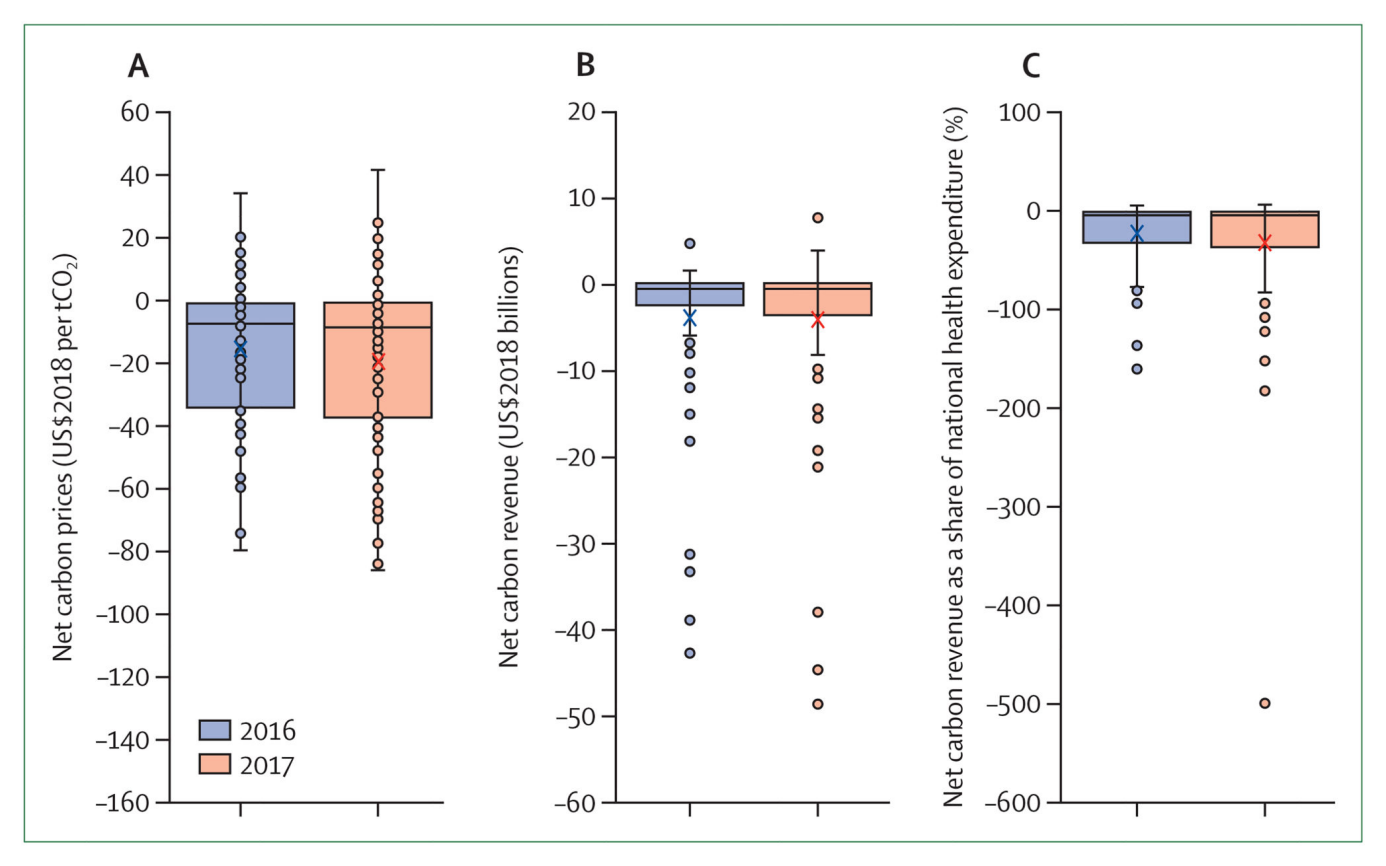
Net carbon prices, net carbon revenues, and net carbon revenue as a share of current national health expenditure across 75 countries in 2016 and 2017 (A) Net carbon prices. (B) Net carbon revenues. (C) Net carbon revenue as a share of current national health expenditure. The boxes represent the IQRs, the horizontal lines inside the boxes represent the medians, and the crosses represent the means. The brackets represent the range from minimum to maximum; however, points are represented as outliers beyond this range if their values are 1·5 times the IQR less than the first quartile or more than the third quartile. tC0_2_=tonnes of carbon dioxide.

Of the 75 countries, 61 (81%) countries in 2016 and 58 (77%) countries in 2017 had net negative carbon prices, and only 14 (19%) countries in 2016 and 17 (23%) countries in 2017 had a price higher than zero, a result of substantial subsidies for fossil fuel production and consumption (figure 25). The median net carbon revenue was negative, a pay-out of $0·66 billion (IQR –0·04 to –3·48), with some countries providing net fossil fuel subsidies in the tens of billions of dollars each year. In many cases, these subsidies were equivalent to substantial proportions of the national health budget—more than 100% in eight of the 75 countries in 2017. Of the 38 countries that had formal carbon pricing mechanisms in place in 2017, 21 still had net negative carbon prices.

### Conclusion

The economic and financial dimensions of public health and climate change are central to any comprehensive mitigation and adaptation effort. This section has covered the health and economic costs of climate change and the indicators of progress underlying a transition to a low-carbon economy. We have developed several new metrics to inform this section and will continue to expand the geographical coverage and reach of these indicators in subsequent reports.

The outlook presented here is mixed. On the one hand, investment in new coal capacity continues to decrease and employment in renewable energy continues to rise. On the other hand, composite indicators of net carbon pricing reveal that government policies are often miscoordinated, resulting in inefficiencies and disrupted price signals. The full economic effects of COVID-19 will continue to develop during the course of several years, leaving a lasting impact on the world. Indeed, the nature and extent of the economic impact and response to this pandemic will have a defining role in determining whether the world meets the commitments of the Paris Agreement. For this reason, strong investment in mitigation and adaptation technologies and interventions is more important now than ever before, and shall lead to healthier and more prepared hospitals, economies, and populations.

## Section 5: public and political engagement

As previous sections made clear, the health impacts of climate change are multiplying, disproportionately affecting those who have contributed least to rising global temperatures. The public are voicing concern as individuals, and as members of communities and new social movements, urging for greater ambition from those with the power to curb carbon emissions.^203–210^

This section tracks engagement in health and climate change across multiple parts of society, including the media, by individuals, scientists, governments, and the corporate sector. For each group, the methods used in previous reports have been enhanced, increasing the sensitivity and specificity of the metrics of health and climate change engagement.

The media, and national newspapers in particular, are central to shaping public perceptions of climate change.^211–214^ The media indicator (indicator 5.1) tracks newspaper coverage of health and climate change in 36 countries, with additional analysis provided for China’s *People’s Daily* (the official voice of the government and China’s most influential newspaper), and content analysis of newspaper coverage in India and the USA.^215,216^

Individual engagement (indicator 5.2) is tracked through the use of Wikipedia, an online information source that has outpaced traditional encyclopaedias in terms of reach, coverage, and comprehensiveness.^217–221^

Reintroduced in the 2020 report with a revised methodology, the scientific indicator (indicator 5.3) tracks academic engagement with health and climate change in peer-reviewed journals, the premier source of high-quality research that provides evidence used by the media, the government, and the public.^218,222,223^

The fourth indicator (indicator 5.4) focuses on the governmental domain, a key arena for driving the global response to climate change. This indicator tracks government engagement in health and climate change at the UN General Assembly, where the UN General Debate provides a platform for national leaders to address the global community.^224,225^ New to the 2020 report, this indicator also examines engagement with health in the NDCs that underpin the UNFCCC 2015 Paris Agreement.^4,226,227^

The final indicator (indicator 5.5) focuses on the corporate sector, which, through the sector’s behaviour and wider political influence, is central to the transition to a low-carbon economy.^228–230^ This indicator tracks engagement with health and climate change in healthcare companies within the UN Global Compact, the world’s biggest corporate sustainability framework.

### Indicator 5.1: media coverage of health and climate change

#### Headline finding: although total coverage of climate change increased substantially from 2018 to 2019, the rise was even greater for coverage of health and climate change, which increased by 96% during this period and has considerably increased from 2007 to 2019

This indicator tracks coverage of health and climate change from 2007 to 2019 in 36 countries, together with separate analyses of China’s *People’s Daily* and the content of coverage in leading newspapers in India and the USA. The analysis of coverage was based on keyword searches (in English, German, Portuguese, and Spanish) for health and climate change in 61 newspapers selected to provide a global spread of high circulation papers. The search strategy was revised for the 2020 report to exclude false positives while retaining true positive articles. Additionally, coverage of health and climate change in *Renmin Ribao*, the Chinese language edition of *People’s Daily*, was tracked by use of keyword searches, algorithm-based natural language processing, and manual screening. The content of coverage of health and climate change was analysed in India (in *The Times of India* and *The Hindustan Times*) and the USA (in *The New York Times* and *The Washington Post*) from July 1, 2019, to Sept 30, 2019, and from Nov 1, 2019, to Dec 31, 2019. These periods were chosen to include extreme weather (monsoons and drought) and the 25th Conference of the Parties (COP; COP25).^28^ The newspapers form part of the elite press that, via their influence on the country’s political and economic elites, have an influence on the policy agenda.^231–236^ Articles were searched by health and climate change keywords and manually screened; the final sample of 209 articles was independently coded by use of the template developed for the 2018 analysis.^28,237^ Full descriptions of the methods, data sources, and further analyses are presented in the appendix (pp 136–168).

**Figure 26:**
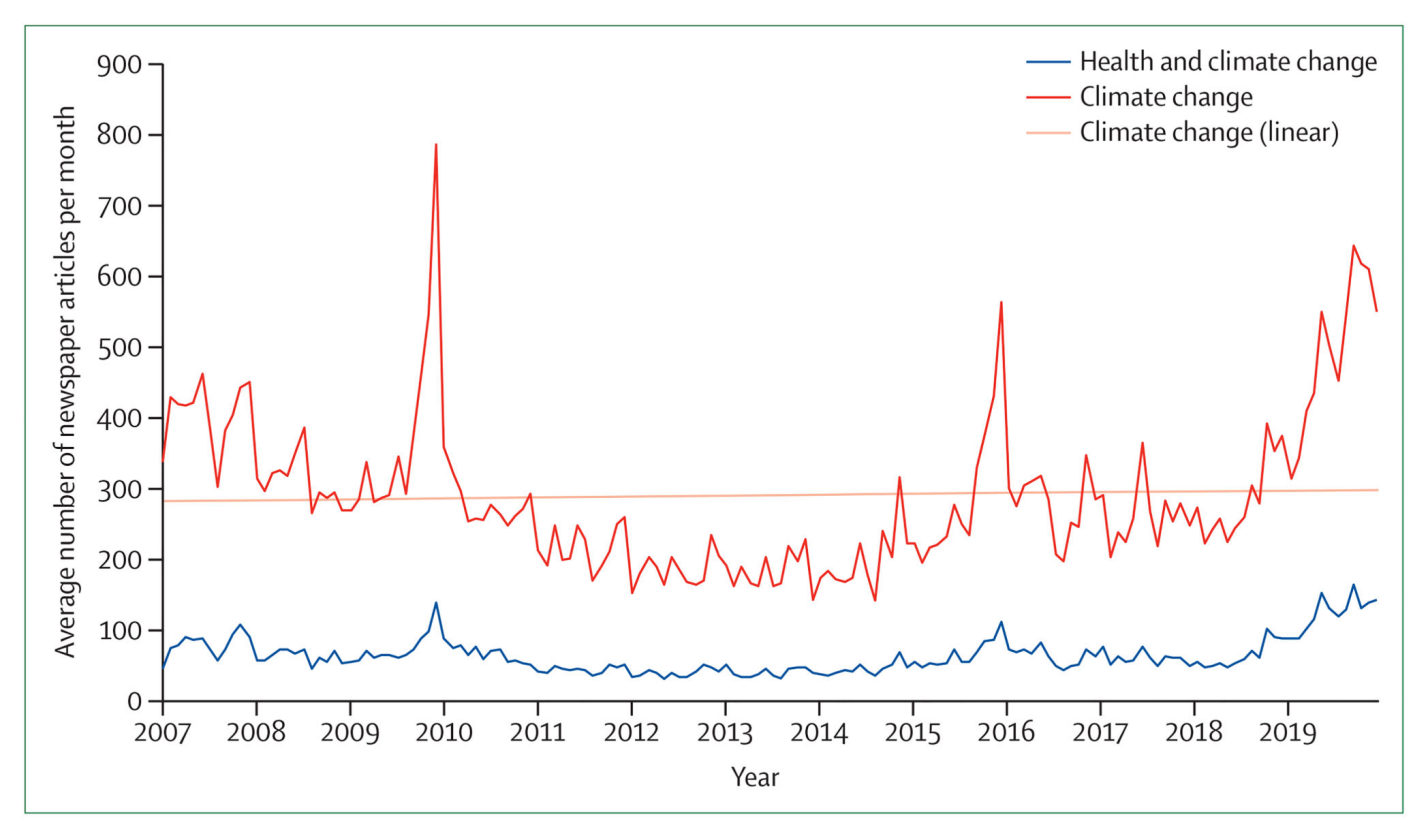
Average monthly coverage of climate change, and health and climate change combined, in 61 newspapers from 36 countries, 2007–19 The non-linear lines represent the average monthly coverage of climate change and health and climate change only across the 61 newspapers. The linear line represents the linear trend of the average number of climate change articles per month between 2007 and 2019.

Across the 36 countries, an increasing proportion of newspaper articles on climate change refer to human health. From 2018 to 2019, health and climate change coverage increased by 96%, outpacing the increase in overall coverage of climate change (74%). From 2007 to 2019, the average monthly number of newspaper articles on health and climate change increased by 57% and the average monthly number of articles on climate change increased by 23%. Overall, the coverage for health and climate change only made up 16% of all climate change coverage in the 2007–19 period (figure 26).

Coverage of health and climate change peaked in months that coincided with the 15th COP (COP15) in 2009 (Copenhagen, Denmark) and the 21st COP (COP21) in 2015 (Paris, France). Coverage rose again in late 2018 and remained high across 2019, corresponding with the rise of the school climate strikes and a series of extreme weather events, including the Californian and southern Australian wildfires.

Between 2008 and 2019, 275 (1·8%) of 15 001 articles on climate change in *People’s Daily* were related to health. Health-related coverage spiked in 2013 because of coverage of the health threats of air pollution and heatwaves.^238^

Regarding the content of coverage in newspapers in India and the USA, three broad themes were identified in articles linking health and climate change. The dominant theme was the health impacts of climate change, discussed in 142 (68%) of 209 articles. References were often to the broad health impacts of climate change (eg, the *Hindustan Times* wrote, on Nov 14, 2019, that “few countries are likely to suffer from the health effects of climate change as much as India”).^239^ More specific connections were also made to climate-related stressors (eg, extreme weather events, wildfires, and population displacement) and health sequelae (eg, vector-borne disease and mental ill health).

The second theme related to the common causes and co-benefits of addressing climate change and health, discussed in 81 (39%) of 209 articles. Air pollution was the most frequently highlighted topic in this theme. The co-benefits of lifestyle changes to protect health and reduce emissions were also noted. The third theme focused on adaptation, discussed in 25 (12%) of 209 articles. For example, the *Times of India*, on Dec 10, 2019, noted that “all levels of government need to prioritize building health system resilience to climate change”.^240^ In addition, a small group of articles (six across the corpus) made a link between health and climate change with respect to activism and protests.

The relative prominence of the three main themes in the 2019 analysis matched that of the 2018 analysis, and the *Times of India* again gave more emphasis to the common causes and co-benefits of addressing climate change and health than did the other newspapers.^28^

### Indicator 5.2: individual engagement in health and climate change

#### Headline finding: individual information seeking about health and climate change increased by 24% from 2018 to 2019, driven mainly by initial interest in health

Wikipedia usage provides a digital footprint of individual information seeking.^241,242^ This indicator tracks individual engagement in health and climate change by capturing visits to pairs of articles (eg, an individual clicking from a page on human health to one on climate change). By use of data from the Wikimedia Foundation on the English version of Wikipedia (representing around 50% of global traffic to all Wikipedia language editions), this indicator is based on 6902 articles related to health and 1837 articles related to climate change.^243,244^ Methods, data sources, and further analyses are described in the appendix (pp 169–182).

In both 2018 and 2019, individuals typically visited articles on either health or climate change, with little co-click activity between these pages. When these articles were linked, the majority (75%) of co-visits started from a health-related page. Although the overall number of health and climate change co-views was low, the value did increase by 24% from 2018 to 2019, pointing to a rising individual engagement in the links between these two topics. In both years, co-clicks increased in months coinciding with key events in climate politics. Co-clicks from articles on climate change to health in 2019 spiked during the COP and in September at the time of Greta Thunberg’s speech at the UN’s Climate Action Summit.^245^

### Indicator 5.3: coverage of health and climate change in scientific journals

#### Headline finding: between 2007 and 2019, original research on health and climate change increased by a factor of eight, a trend driven by research led by scientists in high-income countries

This indicator is based on keyword searches for health and climate change in OVID MEDLINE and OVID Embase and used the comprehensive indexing systems and thesaurus of Medical Subject Headings for MEDLINE and Emtree for Embase. Methods, data sources, and further analyses are described in the appendix (pp 183–193).

**Figure 27:**
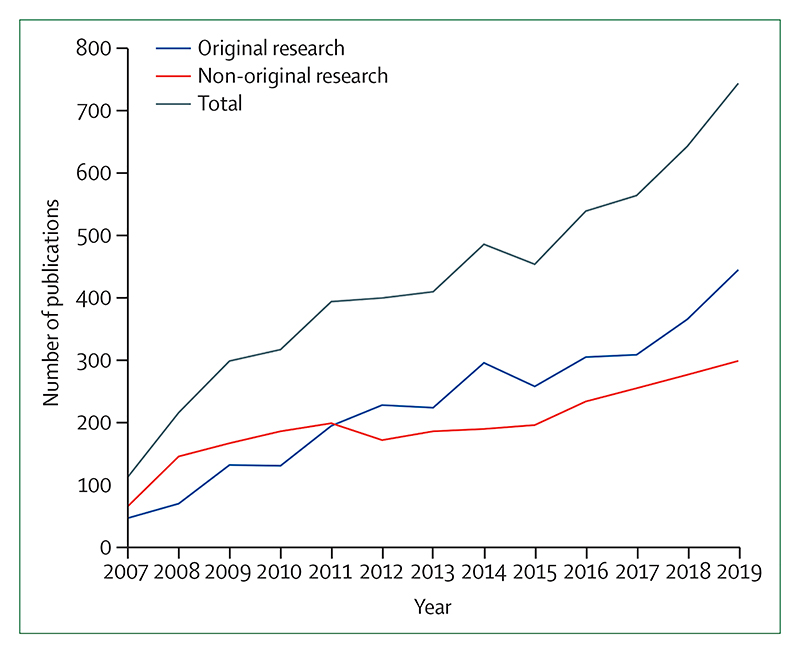
Scientific journal articles relating to health and climate change, 2007–19

Between 2007 and 2019, 5579 published academic articles referred to links between climate change and health. The period saw an increase in original research (ie, primary studies and evidence reviews) by a factor of eight and an increase in research-related articles (ie, editorials, reviews, comments, and letters) by a factor of three. In 2011, the number of original research articles surpassed the number of research-related articles, with new research representing 60% of total scientific output on health and climate change in 2019 (445 of 744 articles; figure 27).

Consistent with observations in section 1 (panel 3), the overall increase in research on health and climate change was mainly led by scientists based in high-income countries. USA-led research made up 1507 (27·0%) of 5579 articles in 2007–19 and 194 (26·1%) of 744 articles in 2019. UK-led research produced 826 (14·8%) articles in 2007–19 and 114 (15·3%) in 2019. Major contributions to the 2019 output also come from the Netherlands (63 [8·5%] of 744) and Switzerland (50 [6·7%] of 744). Increases were also evident for China, South Africa, and India.

Across the same period, articles on health and climate change represented only a small proportion (5579 [9·2%]) of a total of 60 883 articles on climate change. However, the increase in articles relating to health and climate change was greater than the increase in overall climate change output.

### Indicator 5.4: government engagement in health and climate change

#### Headline finding: national governments are increasingly paying attention to health and climate change. Small island developing states are leading this trend at the UN General Debate, and poorer and more climate-vulnerable countries were more likely to reference health in their NDCs, with 95% of least-developed countries making these references

This indicator examines engagement with health and climate change in the UN General Debate and engagement with health in NDCs committed to as part of the 2015 Paris Agreement.^4,224^ The indicator uses keyword searches of the UN General Debate corpus, with algorithm-based, natural language processing applied to the official English versions of the statements.^246,247^ References to health-related terms (eg, “health”, “illness”, “disease”, and “malnutrition”) and climate-related health exposures were examined in the 185 countries who registered their NDCs in the UNFCCC repository by March, 2020, with a total of 2159 pages of text analysed. Building on previous analyses, this indicator analyses references and their prominence in the text.^227,248^ Methods, data sources, and further analyses are described in the appendix (pp 194–218).

As part of the annual UN General Assembly, the UN General Debate provides a global forum for national leaders to discuss issues they consider important. Health has been a long-standing issue, but engagement with climate change was infrequent until the late 1980s. From the mid-2000s, national leaders began to focus on the connections between health and climate change, with the proportion of leaders making these connections rising rapidly from 2007 and peaking in 2014 at 24%.

Engagement in health and climate change continued to be led by the small island developing states, particularly in the Western Pacific region. By contrast, engagement remained low among the more powerful global actors, and particularly among those with the highest CO_2_ emissions (eg, the USA, China, and the EU). For the third consecutive year, President Donald Trump’s statement on behalf of the USA failed to make a single reference to climate change, let alone to the link between climate change and health. However, 2019 did see growing engagement with climate change and health by other high-income countries (eg, Australia, Canada, Germany, and Spain) and by low-income countries, particularly in the African region (eg, Burkina Faso, Botswana, Côte d’Ivoire, Niger, and Togo).

At the 2019 UN General Debate, the majority of health and climate change references focused on the health impacts of climate change. For example, Dominica broached the effects of climate change on small island developing states, highlighting “rising sea levels, violent tropical storms and hurricanes, periods of severe drought alternating with floods and forest fires, new plant diseases, and vector-borne disease such as chikungunya and Zika present an existential threat”.^249^ Similarly, Tonga’s UN General Debate statement discussed how extreme weather events linked to climate change “are increasingly more intense, inflicting damage and destruction on our communities and ecosystems and putting the health of our peoples at risk”.^250^

The 2019 UN General Debate also saw discussion of adaptation and resilience to “upgrade and climate-proof our health-care facilities” (Nauru),^251^ improve “the quality of health care and the durability of health-care systems in the face of the climate crisis” (Palau),^252^ and build “climate change resilience in our sectoral policies and strategies for health, transport, agriculture and pastoral production” (Niger).^253^

**Figure 28:**
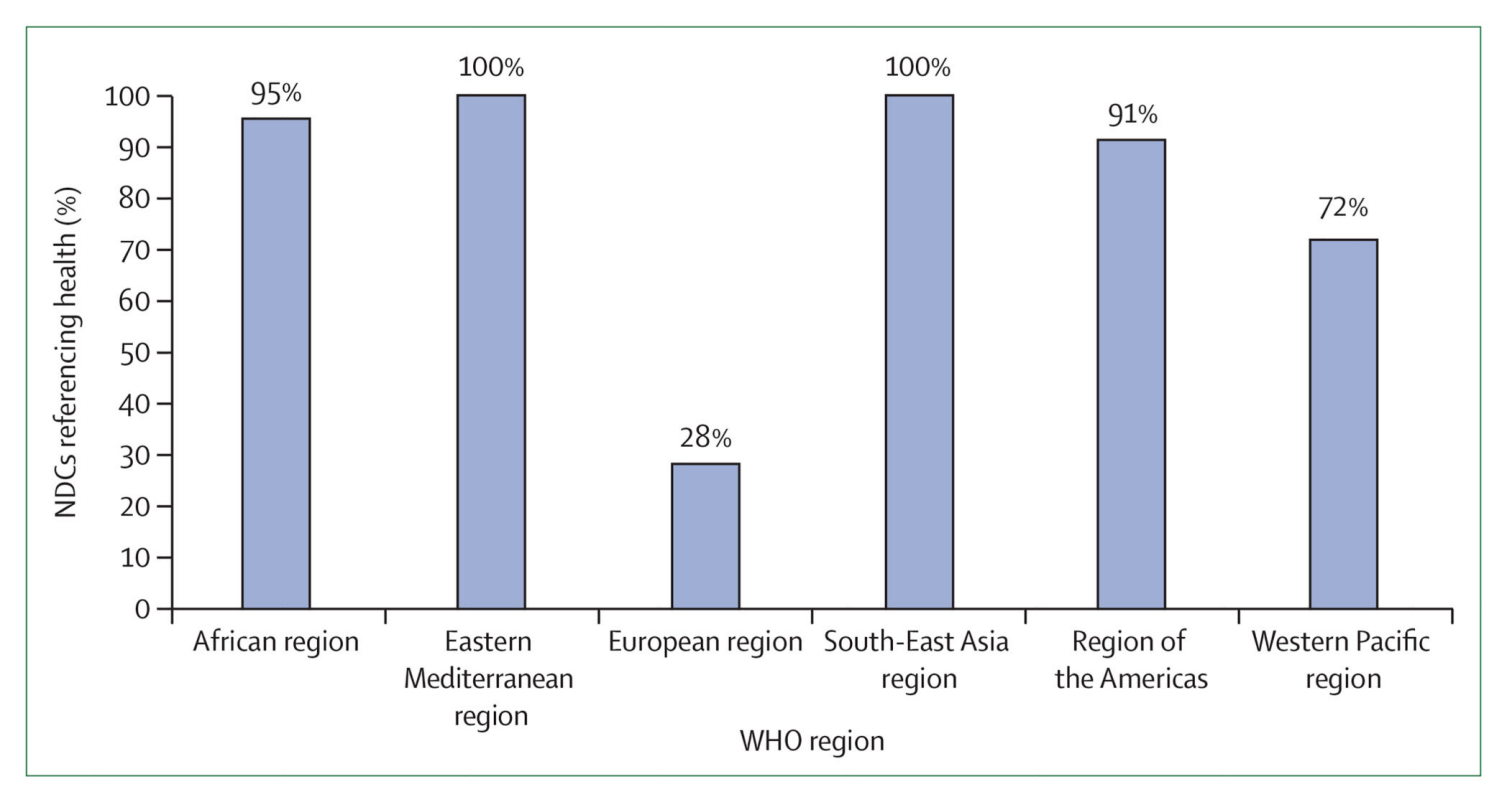
References to health in NDCs by WHO region The European region, which consists of 53 countries, is adjusted for the single NDC representing 28 EU countries; treating the EU as one country would increase the regional proportion of NCDs referencing health to 60%. NDCs=Nationally Determined Contributions.

The second part of this indicator focuses on health within the NDCs, assessing both the references and their prominence within the text. Here, 135 (73%) of 185 NDCs included considerations of public health. At the WHO regional level, all countries in the South-East Asia and Eastern Mediterranean regions discussed these links (figure 28). At the country level, references to health were particularly common among the UNFCCC-defined least-developed countries (40 [95%] of 42). By contrast, the NDCs of the EU (representing the contributions of 28 countries) and the USA did not have any references.

A range of health dimensions were highlighted in the NDCs, including the direct impacts of climate change on health and health-related infrastructure. For example, in their respective NDCs, Morocco noted that climate change would increase deaths “by 250 000 annually between 2030 and 2050 due to malnutrition, malaria, diarrhea and heat-related stress”^254^ and Cambodia discussed the effects of climate change on “death, injury, psychological disorders and damage to public health infrastructure”.^255^ There were also references to the co-benefits of interventions; for instance, Saint Lucia referred to “human health benefits” among “co-benefits associated with its [climate change] mitigation efforts”.^256^

Among the 135 NDCs considering health and climate change, extreme weather events (eg, floods and droughts) and food security were the most commonly cited topics, with 70 (52%) discussing these links. The proportion of NDCs discussing an exposure term in relation to health was highest in the NDCs from countries in the South-East Asia region and was lowest in Europe. Examples included Sri Lanka’s NDC that warned of “water borne diseases” that “can increase due to extreme heat and drought”^257^ and Nepal’s NDC that described “an increased frequency of extreme weather events such as landslides, floods and droughts resulting to the loss of human lives”.^258^

### Indicator 5.5: corporate sector engagement in health and climate change

#### Headline finding: in 2019, engagement in health and climate change increased to 24% among health-care companies in the UN Global Compact, although this engagement continues to lag behind that of other sectors

**Figure 29:**
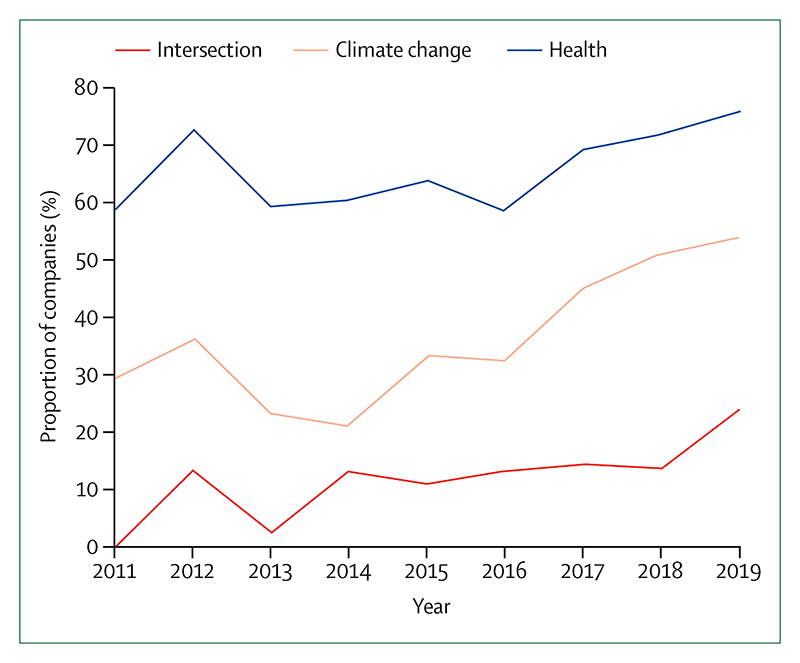
Proportion of health-care sector companies referring to climate change, health, and the intersection of health and climate change in *Communication on Progress* reports, 2011–19

The UN Global Compact is a platform supported by the UN and created to promote environmental and social responsibility in the business sector.^259^ This platform represents more than 10 000 companies from more than 160 countries. Focusing on the health-care sector, this indicator tracks engagement in health and climate change in the *Communication on Progress* reports that companies in the UN Global Compact submit each year (figure 29).

Analysis was based on keyword searches of terms related to health and climate change in 20 775 annual reports in the database of the UN Global Compact, and engagement in health and climate change was identified by use of natural language processing. Methods, data sources, and further analyses are described in the appendix (pp 219–228).

This indicator points to an increase in engagement by the health-care sector in 2019, with 12 (24%) of 50 companies referring to the links between climate change and health (figure 29). However, other sectors had higher levels of engagement than did the health-care sector, including the energy sector and the real estate investment sector.

### Conclusion

Public and political engagement is essential to curb fossil fuel consumption and limit the global temperature rise to less than 1·5°C.^260^ Section 5 has examined indicators of engagement relating to the media, the public, the scientific community, national governments, and the corporate sector. Taken together, the analyses point to two broad trends.

First, engagement with health and climate change continues to increase. Between 2007 and 2019, newspaper coverage increased by more than 50% and scientific journal output increased by more than 500%. Across 2018 and 2019, the proportion of Wikipedia users searching for articles that linked health and climate change also increased. There is evidence of dynamic and reinforcing relationships between these domains. Media coverage increased at times of heightened political and public engagement. As captured by Wikipedia use, there was a spike in individual engagement in health and climate change in September, 2019, coinciding with Greta Thunberg’s speech at the UN Climate Action Summit.

However, beneath these trends are persisting inequalities in wealth and political influence. In both the UN General Debate and the NDCs, engagement in health and climate change is led by countries and regions that are affected most by the changing climate to which they have contributed the least. At the same time, the science of health and climate change continues to be led by high-income, high-emitting countries, which are mainly responsible for climate change.^208,261^

Second, in absolute terms, climate change continues to be framed in ways that pay little attention to its health dimensions. One-sixth of newspaper articles on climate change discuss its health dimensions; less than one-tenth of scientific articles do so, as do less than a quarter of health-care companies signed up to sustainable business practices. In the political domain, health and climate change are rarely connected by government leaders in their speeches at the UN’s major global forum and, although most NDCs refer to health, the NDCs of countries with high per-capita carbon emissions, including EU countries and the USA, do not. Nonetheless, in key domains of engagement, the health dimensions of climate change are increasingly recognised, with media and scientific coverage rising more rapidly for health and climate change than for climate change as a whole.

Despite the fact that underlying inequalities in the drivers and effects of climate change remain, there is evidence that health is becoming increasingly central to public and political engagement.

## Conclusion: the 2020 report of the *Lancet* Countdown

With the global average temperature having risen to 1·2°C more than that in preindustrial times, the indicators contained in the 2020 report provide insights into the health impacts of climate change today and in the future. Extremes of heat affect vulnerable populations the most, with some 296 000 deaths occurring as a result of high temperatures in 2018 (indicator 1.1.3).

The climate suitability for the transmission of a range of infectious diseases—dengue fever, malaria, and those caused by *Vibrio* bacteria—has risen across the world (indicator 1.3.1). At the same time, crop yield potential has fallen for each of the major crops tracked, with dire consequences anticipated for food-insecure populations (indicator 1.4.1).

And yet, the global response has remained muted. The carbon intensity of the global energy system has been stable during the past three decades, and global coal use for energy increased by 74% during the same period (indicators 3.1.1 and 3.1.2). This rise has resulted in approximately 390 000 deaths from PM_2·5_ generated by coal-fired power, with total global mortality for all ambient sources exceeding 3·01 million deaths, in 2018 (indicator 3.3). In the agricultural sector, emissions from livestock grew by 16% from 2000 to 2017, with some 990 000 deaths occurring globally from excess red meat consumption in 2017 (indicators 3.5.1 and 3.5.2).

In the face of these problems, the response from the health profession continues to gain momentum. Spending on health system adaptation continued to increase, rising by 12·7% in 2019 to $18·4 billion (indicator 2.4). In just more than 10 years, original research on health and climate change has increased by a factor of eight, and, in half that time, health institutions with total assets of $42 billion have divested their holdings from fossil fuel industries (indicators 5.3 and 4.2.3). Led by low-income countries, more governments are linking health and climate change in their annual speeches at the UN General Debate and their NDCs under the Paris Agreement.

The public health and financial effects of COVID-19 will be felt for years to come, and efforts to protect and rebuild local communities and national economies will need to be robust and sustained. Despite concerning indicators across each section of this report, the 2021 UN Climate Change Conference presents an opportunity for course correction and revitalised NDCs. The window of opportunity is narrow, and, if the response to COVID-19 is not fully and directly aligned with national climate change strategies, the world will be unable to meet its commitments under the Paris Agreement, damaging health and health systems today, and in the future.

## Supplementary Material

Chinese translation of the abstract

French translation of the abstract

German translation of the abstract

Spanish translation of the abstract

Supplementary appendix

## Figures and Tables

**Table 1:** Potential heat-related work hours lost

	Billions of work hours lost in 2000 (n=199·0)	Billions of work hours lost in 2019 (n=302·4)	Work hours lost per person in 2019
Global	199·0	302·4 (100·0%)	52·7
India	75·0	118·3 (39·1%)	111·2
China	33·4	28·3 (9·4%)	24·5
Bangladesh	13·3	18·2 (6·0%)	148·0
Pakistan	9·5	17·0 (5·6%)	116·2
Indonesia	10·7	15·0 (5·0%)	71·8
Vietnam	7·7	12·5 (4·1%)	160·3
Thailand	6·3	9·7 (3·2%)	164·4
Nigeria	4·3	9·4 (3·1%)	66·7
Philippines	3·5	5·8 (1·9%)	71·4
Brazil	2·8	4·0 (1·3%)	23·3
Cambodia	1·7	2·2 (0·7%)	202·2
USA	1·2	2·0 (0·7%)	7·1
Mexico	0·9	1·7 (0·6%)	17·4
Rest of the world	28·7	58·3 (19·3%)	27·5

Data are n or n (%). For these estimates, all agricultural and construction work was assumed to be in the shade or indoors—the lower bounds of potential work hours lost. Work hours lost per person were estimated for the population older than 15 years.

**Table 2:** Detection and attribution studies linking extreme weather events to climate change from 2015 to 2020

	Anthropogenic influence increased event likelihood or strength	Anthropogenic influence decreased event likelihood or strength	Anthropogenic influence not identified or uncertain
Heat (36 studies; 32 events)	Events ending in 2015 in India, Pakistan, China, Indonesia, Europe,^8,52^ Egypt, Japan, southern India and Sri Lanka, Australia, and worldwide;^8,53^ in 2016 in southern Africa, Thailand, Asia, and worldwide; in 2017 in Australia,^54^ the USA, South Korea, western Europe,^55^ China, and the Euro-Mediterranean region; in 2018 in northeast Asia, the Iberian Peninsula, and Europe; in 2019 in France^56^ and western Europe;^57^ and in 2020 in Australia^58^	··	Events ending in 2015–16 in India^59^
Cold and frost (nine studies; eight events)	Events ending in 2016 in Australia	Events ending in 2015 in the USA; in 2016 in China; and in 2018 in North America^60^ and the UK	··
Drought and reduced precipitation (26 studies; 24 events)	Events ending in 2015 in the USA, Canada, Ethiopia, Indonesia, and Australia; in 2016 in southern Africa and Thailand; in 2017 in east Africa, the USA, and China; and in 2018 in South Africa,^61^ China, and the USA	··	Events ending in 2015 in Brazil,^62^ Nigeria, and Ethiopia;^63^ in 2016 in Brazil, the USA, Somalia,^64^ and western Europe; in 2017 in Kenya^65^ and the USA; and in 2019 in Australia^58^
Wildfire (five studies; six events)	Events ending in 2015 in the USA; in 2016 in Australia and western North America; in 2018 in Australia; and in 2020 in Australia^58^	··	Events ending in 2017 in Australia
Heavy precipitation and flood (23 studies; 19 events)	Events ending in 2015 in China and the USA; in 2016 in France,^66^ China, and Louisiana (USA);^67^ in 2017 in Bangladesh, Peru, Uruguay, and China; and in 2018 in the USA and Japan^6,68^	Events ending in 2018 in China	Events ending in 2015 in India; in 2016 in Germany^66^ and Australia; in 2017 in Bangladesh;^69^ and in 2018 in Mozambique, Zimbabwe and Zambia, Australia, India,^70^ and China*
Storms (eight studies; eight events)	Events ending in 2015 in the UK^71^ and the western north Pacific;^72^ in 2017 in the USA;^73^ in 2018 in the USA;^74^ and in 2019 in the USA^75^	··	Events ending in 2016 in the USA and in 2018 in western Europe^76^
Marine heat and melting sea ice (13 studies; ten events)	Events ending in 2015 in the northern hemisphere; in 2016 in the USA, Australia, the Coral Sea,^7,77^ the North Pole,^7,78^ the Gulf of Alaska and the Bering Sea, and the central equatorial Pacific; and in 2018 in the Tasman Sea and the Bering Sea	··	Events ending in 2015 in the central equatorial Pacific and in 2016 in the eastern equatorial Pacific
Total studies	81	6	27
Total events	76	5	28

Events have been listed according to the year in which they ended. In some countries and regions, multiple events in the same year were studied. References were gained from papers published in the *Bulletin of the American Meteorological Society*,^5–8^ or otherwise are listed separately.

* Anthropogenic influence had varied effects.

## Panel 1 Health, climate change, and COVID-19

As of Nov 9, 2020, the COVID-19 pandemic has spread to 190 countries, with more than 50 493 000 cases confirmed and more than 1 257 700 deaths recorded.^16^ The scale and extent of the suffering, and the social and economic toll, will continue to evolve over the coming months, with the effects of the pandemic likely to be felt for years to come.^17^ The relationship between the spread of existing and novel infectious diseases, worsening environmental degradation, deforestation, and change in land use, and animal ill health has long been analysed and described. Equally, both climate change and COVID-19 act to exacerbate existing inequalities within and between countries.^18–20^

As a direct consequence of the pandemic, an 8% reduction in greenhouse gas emissions is projected for 2020, which would be the most rapid 1-year decline on record.^21^ Crucially, these reductions do not represent the decarbonisation of the economy required to respond to climate change, but simply the freezing of economic activity. Equally, the 1·4% reduction in greenhouse gas emissions that followed the 2008 global financial crisis was proceeded by a rebound, with emissions rising by 5·9% in 2010. Likewise, it is unlikely that the current fall in emissions will be sustained, with any reductions being potentially outweighed by a shift away from otherwise ambitious policies for climate change mitigation. However, this route need not be taken.^21^ Over the next 5 years, considerable financial, social, and political investment will be required to continue to protect populations and health systems from the worst effects of COVID-19, to safely restart and restructure national and local economies, and to rebuild in a way that prepares for future economic and public health shocks. Harnessing the health co-benefits of climate change mitigation and adaptation will ensure the economic, social, and environmental sustainability of these efforts, while providing a framework that encourages investment in local communities and health systems and synergises with existing health challenges.^22^

Multiple, ready-to-go examples of such alignment are available, such as commonalities between future pandemic preparedness and effective health adaptation to climate-related impacts.^23^ In climate-related health adaptation, decision making under deep uncertainty necessitates the use of the principles of flexibility, robustness, economic low regrets, and equity to guide decisions.^24^ At the broader level, reducing poverty and strengthening health systems will both stimulate and restructure economies, and are among the most effective measures to enhance community resilience to climate change.^3^

Turning to mitigation, at a time when more and more countries are closing down the last of their coal-fired power plants and oil prices are reaching record lows, the fossil fuel sector is expected to be more affected than is the renewable energy sector.^21^ If done with care and adequate protection for workers, government stimulus packages are well placed to prioritise investment in healthier, cleaner forms of energy. The response to COVID-19 has encouraged a rethinking of the scale and pace of ambition. Health systems have restructured services practically overnight to conduct millions of primary care and specialist appointments online, and a sudden switch to online work and virtual conferencing has shifted investment towards communications infrastructure instead of aviation and road transport.^25,26^ A number of these changes should be reviewed, improved on, and retained over the coming years.

It is clear that a growing body of literature and rhetoric will be inadequate to respond to climate change, and this work must take advantage of the moment to combine public health and climate change policies in a way that addresses inequality directly. The UN Framework Convention on Climate Change’s 26th Conference of the Parties, which is postponed to 2021 and is set to be in Glasgow, UK, presents an immediate opportunity to ensure the long-term effectiveness of the response to COVID-19 by linking the recovery to countries’ revised commitments (Nationally Determined Contributions) under the Paris Agreement. The solution to one economic and public health crisis must not exacerbate another, and, in the long term, the response to COVID-19 and climate change will be the most successful when they are closely aligned.

## Panel 2 The indicators of the 2020 report of The *Lancet* Countdown

### Climate change impacts, exposures, and vulnerabilities

1.1: health and heat
  1.1.1: vulnerability to the extremes of heat
  1.1.2: exposure of vulnerable populations to heatwaves
  1.1.3: heat-related mortality
  1.1.4: change in labour capacity
1.2: health and extreme weather events
  1.2.1: wildfires
  1.2.2: flood and drought
  1.2.3: lethality of extreme weather events
1.3: climate-sensitive infectious diseases
  1.3.1: climate suitability for infectious disease transmission
  1.3.2: vulnerability to mosquito-borne diseases
1.4: food security and undernutrition
  1.4.1: terrestrial food security and undernutrition
  1.4.2: marine food security and undernutrition
1.5: migration, displacement, and rising sea levels

### Adaptation, planning, and resilience for health

2.1: adaptation planning and assessment
  2.1.1: national adaptation plans for health
  2.1.2: national assessments of climate change impacts, vulnerability, and adaptation for health
  2.1.3: city-level climate change risk assessments
2.2: climate information services for health
2.3: adaptation delivery and implementation
  2.3.1: detection, preparedness, and response to health emergencies
  2.3.2: air conditioning: benefits and harms
  2.3.3: urban green space
2.4: spending on adaptation for health and health-related activities

### Mitigation actions and health co-benefits

3.1: energy system and health
  3.1.1: carbon intensity of the energy system
  3.1.2: coal phase-out
  3.1.3: zero-carbon emission electricity
3.2: clean household energy
3.3: premature mortality from ambient air pollution by sector
3.4: sustainable and healthy transport
3.5: food, agriculture, and health
  3.5.1: emissions from agricultural production and consumption
  3.5.2: diet and health co-benefits
3.6: mitigation in the health-care sector

### Economics and finance

4.1: the health and economic costs of climate change and benefits from mitigation
  4.1.1: economic losses due to climate-related extreme events
  4.1.2: costs of heat-related mortality
  4.1.3: loss of earnings from heat-related reduction in labour capacity
  4.1.4: costs of the health impacts of air pollution
4.2: the economics of the transition to zero-carbon economies
  4.2.1: investment in new coal capacity
  4.2.2: investments in zero-carbon energy and energy efficiency
  4.2.3: employment in low-carbon and high-carbon industries
  4.2.4: funds divested from fossil fuels
  4.2.5: net value of fossil fuel subsidies and carbon prices

### Public and political engagement

5.1: media coverage of health and climate change
5.2: individual engagement in health and climate change
5.3: coverage of health and climate change in scientific journals
5.4: government engagement in health and climate change
5.5: corporate sector engagement in health and climate change

## Panel 3 Quantifying the links between climate change, human health, and extreme events

Formal statistical methods, grouped as detection and attribution studies, are already used widely in other sectors, and are increasingly deployed to quantify the extent to which climate change has had observed impacts on population health and health systems.^79–81^ However, detection and attribution studies focusing on the changing likelihood and intensity of extreme events are generally limited to meteorological events in high-income and upper-middle-income countries. Further development of this body of literature offers an essential and unique way of improving understanding of current impacts and future risks of climate change on lives and livelihoods, guiding evidence-based management and adaptation. The following three case studies illustrate the linkage of detection and attribution studies of meteorological events to the resulting health impacts.

### 1 Reduced sea ice in the Arctic region

The Arctic region is warming two to three times faster than the global annual average, with observable impacts for Arctic communities, but limited data on the health consequences.^82^ Extreme weather events, shifting migration patterns, and warmer and shorter winters now threaten food security and vital infrastructure.

The winter of 2017–18 heralded warm temperatures and an extreme low ice year in the Bering Sea.^83^ The extent of sea ice was the lowest in recorded and reconstructed history: an estimated two in 1800 year event considering preindustrial climate forcing according to one study.^84^ This study also suggested that climate change was responsible for 90% of the attributable risk, and that this extent of sea ice might become the mean within 20 years.^84^

This low ice year had multiple detrimental effects on communities in western Alaska, USA, although the health impacts have rarely been measured. These communities generally depend on sea ice for transportation, hunting and fishing, coastal buffering from storms, and a host of other ecosystem services. During this period of record low sea ice, a range of events occurred, including a loss of power, and damage to the water treatment plant, in Little Diomede (an Alaskan island) and a fatal accident that resulted from open waterholes along a previously frozen travel corridor on the Kuskokwim River.^85–87^

### 2 Northern European heatwaves in 2018 and 2019

During the summer of 2018, parts of northern Scandinavia experienced record breaking daily temperatures that were more than 5°C warmer than those in 1981–2010, an occurrence that evidence suggests was made five times more probable as a result of climate change.^88^ In Sweden, the Public Health Agency estimated an excess mortality of 750 deaths between July and August, 2018, with more than 600 of these attributed to higher temperatures, when compared with the same weeks in 2017.^89^

Countries across western Europe and Scandinavia again experienced record breaking temperatures in 2019, with the temperatures in several countries exceeding 40°C for 3–4 days during June and July. Attribution studies suggest climate change was responsible for a ten times increase in the likelihood of the event occurring, and a 1·2–3·0°C increase in the temperature of these events, with almost 1500 deaths in France and 400 deaths in the Netherlands occurring because of these events.^57,90,91^

### 3 Japan heatwave of 2018

The summer of 2018 in Japan saw a combination of a national emergency resulting from extreme precipitation followed closely by record breaking temperatures. The event had roughly a 20% probability of occurring in today’s world compared with a probability of 0% in a world without climate change.^92,93^ Another attribution study compared modest and extreme heatwave days with a 1941–79 baseline, concluding that the probability of the defined heatwave event was 1·5 times higher for 1980–2018 and 7·0–8·0 times higher for 2019–50. This hot summer had large health implications. In 2018, there were an estimated 14 200 heat-related deaths in the population in Japan aged more than 65 years—more than 3000 more deaths than the previous record set in 2010, and 8100 more than the 2000–04 average (indicator 1.1.3).

## Panel 4 For a greener National Health Service

With more than 1·5 million employees, England’s National Health Service (NHS England) is the largest single employer in Europe and the largest single-payer health-care system in the world, with an annual budget of £134 billion. Although providing high-quality health care to a population of almost 56 million people, NHS England contributes to 4–5% of the country’s total greenhouse gas emissions. Accountable to both NHS England and Public Health England, the Sustainable Development Unit was founded in 2008 to ensure the health service met its commitments under the UK Climate Change Act. Since then, the NHS has achieved impressive reductions in greenhouse gas emissions while maintaining high standards of care and decreasing costs, reducing delivery of care emissions by 57% and emissions from its supply chain and broader responsibilities by 22% compared with 1990 levels.^175^ In January, 2020, NHS England announced its commitment to become the world’s first net zero health system, alongside a new campaign for a greener NHS.^176^ A new baseline of NHS England’s carbon footprint was quantified and different sources of emissions were identified by use of a hybrid model of bottom-up measurements of direct emissions (ie, onsite fossil fuel use, fleet and transport, and anaesthetic gases) and energy use, and top-down measurements based on multiregional input–output models to estimate other indirect emissions (eg, from the upstream energy system, pharmaceutical procurement, and patient use of metred dose inhalers). NHS England is now working to develop a strategy for how and when net zero emissions can be achieved.
