# Supplementary material for: The 2020 report of the Lancet Countdown on health and climate change: responding to converging crises
Source: Lancet. Author manuscript; Available in PMC 2024 Nov 15. (PMC7616803; doi:10.1016/S0140-6736(20)32290-X)
Supplement: Chinese translation of the abstract [file EMS200002-supplement-Chinese_translation_of_the_abstract.pdf]

THE LANCET

Supplementary appendix

This translation in Chinese was submitted by the authors and we reproduce it as supplied. It has not been peer reviewed. *The Lancet*'s editorial processes have only been applied to the original in English, which should serve as reference for this manuscript.

此简体中文译文由作者提交，我方按照提供的版本刊登。此译文并未经过同行审阅。医学期刊《柳叶刀》的编辑流程仅适用于英文原稿，英文原稿应作为此手稿的参考。

Supplement to: Watts N, Amann M, Arnell N, et al. The 2020 report of The *Lancet* Countdown on health and climate change: responding to converging crises. *Lancet* 2020; published online Dec 2. http://dx.doi.org/10.1016/S0140-6736(20)32290-X.

“柳叶刀健康与气候变化倒计时”2020年报告：协同应对交织的危机

Nick Watts, Markus Amann, Nigel Arnell, Sonja Ayeb-Karlsson, Jessica Beagley, Kristine Belesova, Maxwell Boykoff, Peter Byass, Wenjia Cai, Diarmid Campbell-Lendrum, Stuart Capstick, Jonathan Chambers, Samantha Coleman, Carole Dalin, Meaghan Daly, Niheer Dasandi, Shouro Dasgupta, Michael Davies, Claudia Di Napoli, Paula Dominguez-Salas, Paul Drummond, Robert Dubrow, Kristie L Ebi, Matthew Eckelman, Paul Ekins, Luis E Escobar, Lucien Georgeson, Su Golder, Delia Grace, Hilary Graham, Paul Haggar, Ian Hamilton, Stella Hartinger, Jeremy Hess, Shih-Che Hsu, Nick Hughes, Slava Jankin Mikhaylov, Marcia P Jimenez, Ilan Kelman, Harry Kennard, Gregor Kiesewetter, Patrick L Kinney, Tord Kjellstrom, Dominic Kniveton, Pete Lampard, Bruno Lemke, Yang Liu, Zhao Liu, Melissa Lott, Rachel Lowe, Jaime Martinez-Urtaza, Mark Maslin, Lucy McAllister, Alice McGushin, Celia McMichael, James Milner, Maziar Moradi-Lakeh, Karyn Morrissey, Simon Munzert, Kris A Murray, Tara Neville, Maria Nilsson, Maquins Odhiambo Sewe, Tadj Oreszczyn, Matthias Otto, Fereidoon Owfi, Olivia Pearman, David Pencheon, Ruth Quinn, Mahnaz Rabbaniha, Elizabeth Robinson, Joacim Rocklöv, Marina Romanello, Jan C Semenza, Jodi Sherman, Liuhua Shi, Marco Springmann, Meisam Tabatabaei, Jonathon Taylor, Joaquin Triñanes, Joy Shumake-Guillemot, Bryan Vu, Paul Wilkinson, Matthew Winning, Peng Gong*, Hugh Montgomery*, Anthony Costello*

执行摘要

“柳叶刀倒计时”是一项国际合作项目，旨在提供一个独立的全球监测系统，专门跟踪不断变化的气候中新出现的健康状况。

2020年报告提出了5大领域43项指标，包括：气候变化影响、暴露程度和脆弱性；针对健康的适应措施、规划和恢复力；减缓措施及健康协同效益；经济和投资；以及公众和政治参与。该报告代表了“柳叶刀倒计时”系统里的35家顶尖学术机构和联合国机构的发现和共识，同时还汇聚了专业人士的见解，包括气候学家、地理学家、工程师、能源专家、粮食专家、交通专家、经济学家、社会学家、政治学家、数据科学家、公共卫生专业人员和医生等。

气候变化引发的健康影响日益严重

五年前，各国政府把将全球温升控制在“远低于2℃”的目标写入了具有里程碑意义的《巴黎协定》。五年过去了，全球CO_2排放量仍然稳步增长，并没有令人信服或持续的减排，因此全球平均气温已经上升了1.2℃。事实上，2015-2019年是有记录以来最热的五年。

气候变化已在全球范围内对影响健康的社会和环境决定因素产生重大影响。在气候变化影响、暴露程度和脆弱性领域，本项目跟踪的所有指标都在恶化。在所监测的气候变化相关的每种人类健康症状都呈现出令人担忧且往往加速恶化的趋势，而2020年的指标则呈现了自“柳叶刀倒计时”成立以来最令人担忧的前景。

这些健康影响通常不是公平分布的，造成问题越少的人群往往受到更大的影响。这揭示了一个更深层次的公平问题，即气候变化会与现有的社会和经济不平等相互作用，加剧国家内部和国家之间不平等的长期趋势。对气候变化成因的研究也有类似发现，许多碳密集型的实践和政策导致了空气质量、食品质量和住房质量的下降，严重损害了脆弱人群的健康。

相比历史平均水平，脆弱人群在全球范围内多遭受了4.75亿次热浪事件，也因此导致了发病和死亡的增加。在过去20年里，65岁以上老年人的热相关死亡数增加了53.7%，这一数值在2018年达到了29.6万人（指标1.1.2和1.1.3）。在人的生命和痛苦方面的高昂成本将显著影响经济产

Published Online
December 2, 2020
https://doi.org/10.1016/S0140-6736(20)32290-X

*Co-chairs

Institute for Global Health (N Watts MA, J Beagley BA, S Coleman MSE, Prof I Kelman PhD, A McGushin MSc, M Romanello PhD), **Office of the Vice Provost for Research** (Prof A Costello FmedSci), **Energy Institute** (S-C Hsu MSc, I Hamilton PhD, H Kennard PhD, Prof T Oreszczyn PhD), **Institute for Sustainable Resources** (C Dalin PhD, P Drummond MSc, Prof P Ekins PhD, N Hughes PhD, M Winning PhD), **Institute for Environmental Design and Engineering** (Prof M Davies PhD), **Department of Geography** (Prof M Maslin PhD), **and Institute for Human Health and Performance** (Prof H Montgomery MD), **University College London, London, UK; Air Quality and Greenhouse Gases Program, International Institute for Applied Systems Analysis, Laxenburg, Austria** (M Amann PhD, G Kiesewetter PhD); **Department of Meteorology** (Prof N W Arnell PhD) **and School of Agriculture, Policy, and Development** (C Di Napoli PhD, Prof E Robinson PhD), **University of Reading, Reading, UK; Institute for Environment and Human Security, United Nations University, Bonn, Germany** (S Ayeb-Karlsson PhD); **Centre on Climate Change and Planetary Health** (K Belesova PhD), **Department**

of Population Health (P Dominguez-Salas PhD), Centre for Mathematical Modelling of Infectious Diseases (R Lowe PhD), and Department of Public Health, Environments, and Society (J Milner PhD, Prof P Wilkinson FRCP), London School of Hygiene & Tropical Medicine, London, UK; Environmental Studies Program, University of Colorado Boulder, Boulder, CO, USA (Prof M Boykoff PhD, O Pearman MEM); Department of Epidemiology and Global Health (Prof P Byass PhD, Prof M Nilsson PhD) and Department of Public Health and Clinical Medicine (M O Sewe PhD, Prof J Rocklöv PhD), Umeå University, Umeå, Sweden; Department of Earth System Science, Tsinghua University, Beijing, China (W Cai PhD, Prof P Gong PhD, Z Liu PhD); Environment, Climate Change and Health Department, World Health Organization, Geneva, Switzerland (D Campbell-Lendrum DPhil, T Neville MSc); School of Psychology, Cardiff University, Cardiff, UK (S Capstick PhD, P Haggar PhD); Institute for Environmental Sciences, University of Geneva, Geneva, Switzerland (J Chambers PhD); Department of Environmental Studies, University of New England, Biddeford, ME, USA (M Daly PhD); School of Government, University of Birmingham, Birmingham, UK (N Dasandi PhD); Centro Euro-Mediterraneo sui Cambiamenti Climatici, Venice, Italy (S Dasgupta PhD); Yale Center on Climate Change and Health (Prof R Dubrow PhD) and Department of Anesthesiology (J Sherman MD), Yale University, New Haven, CT, USA; Department of Global Health (Prof K L Ebi PhD) and Center for Health and the Global Environment (J Hess MD), University of Washington, Seattle, WA, USA; Department of Civil & Environmental Engineering, Northeastern University, Boston, MA, USA (M Eckelman PhD); Department of Fish and Wildlife Conservation, Virginia Polytechnic Institute and State University, Blacksburg, VA, USA (L E Escobar PhD); Oxford

出，2019年损失的潜在劳动小时数超过3020亿小时（指标1.1.4）。印度和印度尼西亚是受影响最严重的国家，其损失的潜在劳动小时所带来的经济影响相当于其年度国内生产总值的4-6%（指标4.1.3）。2018年，欧洲热相关死亡的货币化成本相当于该地区国民总收入的1.2%，或者相当于1100万欧洲公民的平均年收入（指标4.1.2）。

至于极端天气，气候科学的进步使得极端天气的归因越来越准确和稳健。2015年至2020年的研究表明，76次洪水、干旱、风暴和气温异常都有气候变化的痕迹（指标1.2.3）。此外，2001-2004年至2016-2019年期间，全球有114个国家中的人们面临的野火风险非常高或极高的天数有所增加（指标1.2.1）。相应地，被调查的全球城市中有67%都预期气候变化将严重损害其公共卫生资产和基础设施（指标2.1.3）。

气候变化会产生下游效应，影响更广泛的环境系统，进而危害人类健康。全球粮食安全因气温上升和极端天气事件发生频率增加而备受威胁；1981年至2019年间，全球主要农作物的产量潜力下降了1.8-5.6%（指标1.4.1）。自1950年代以来，传染病传播的气候适宜性一直在迅速增长，2018年由白纹伊蚊引起的登革热的气候适宜性增加了15%，同时疟疾和弧菌传播的气候适宜性也呈区域性增长趋势（指标1.3.1）。基于目前的人口，预计未来约有1.45亿至5.65亿人将面临海平面上升而被淹没的危险（指标1.5）。

尽管出现了这些明显且逐步恶化的趋势，但全球应对气候变化的行动仍然不足，各国所作努力仍然远未达到《巴黎协定》中作出的承诺。过去的30年来，全球能源系统的碳强度几乎保持不变，在此期间全球煤炭使用量增长了74%（指标3.1.1和3.1.2）。2013年以来全球煤炭使用量减少的状况在近两年已连续逆转；从2016年到2018年，煤炭使用量增长了1.7%。健康负担沉重——燃煤发电带来的空气污染每年造成超过100万人死亡，其中2018年约有39万人死于颗粒物污染（指标3.3）。粮食和农业部门的反应同样令人担忧。从2000年到2017年，牲畜的温室气体排放量增长了16%，其中93%的排放量来自反刍动物（指标3.5.1）。同样，越来越不健康的饮食在世界范围内流行，过量食用红肉在2017年导致约99万例相关的死亡（指标3.5.2）。在各国达成《巴黎协定》的五年后，不少指标与以往报告中展现的积极性趋势相比，已开始出现了早期但持续性的逆转局面（指标1.3.2、3.1.2和4.2.3）

卫生专业人员的回应日益增加

尽管缺乏全经济体的系统性改善，但本报告在几个关键部门发现了相对的进展：可再生能源装机量在2010到2017年间以每年21%的速度迅速增长，低碳电力现已占到中国2017年发电装机的28%（指标3.1.3）。“柳叶刀倒计时”2020年报告中呈现的指标表明，在全球范围内，卫生专业人员参与应对气候变化问题日趋明显，取得了最为可观的进展。在卫生医疗系统的适应和减缓、理解和最大限度发挥应对气候变化措施的健康效益，以及传达加快应对行动的必要性等多个方

 www.thelancet.com Published online December 2, 2020 https://doi.org/10.1016/S0140-6736(20)32290-X

面，医生、护士和更广泛的卫生专业人员都发挥着至关重要的作用。

就国家卫生系统的适应而言，这种变化正在发生。令人印象深刻的是，现在有86个国家的卫生服务部门和相应的气象服务部门建立了联系，以协助制定国家健康适应计划（指标2.2）。目前至少有51个国家制定了国家健康适应计划，全球在健康适应方面的支出在2018-19年度增加到所有适应支出的5.3%，2019年达到184亿美元（指标2.1.1和2.4）。

2017年，医疗部门的温室气体排放占到全球总排放的4.6%，医疗部门正采取重要举措来减少自身排放，尽管这些措施还处于早期阶段（指标3.6）。在英国，国民医疗服务体系（National Health Service，简称NHS）已经宣布要尽快实现“净零排放的医疗服务”的目标，NHS在过去已经取得了令人瞩目的进展：自1990年以来，NHS的医疗服务直接排放量减少了57%，而考虑供应链的排放则减少了22%。在其他地方，西澳大利亚州立卫生部（Western Australian Department of Health）利用其2016年《公共卫生法》进行了澳大利亚首次气候与健康咨询，德国联邦卫生部（German Federal Ministry of Health）成立了专门的健康保护和可持续性部门，负责气候相关事务。这一进展在世界各地越来越普遍，73%的国家在《巴黎协定》下做出的国家承诺中明确提及健康和福祉，这一比例在东南亚和东地中海地区国家高达100%（指标5.4）。同样，在涉及健康和气候变化相互联系的联合国一般性辩论中，最不发达国家和小岛屿发展中国家正发挥着越来越大的全球领导作用（指标5.4）。

卫生专业人员及其行业协会也在积极行动，医疗卫生机构已承诺从化石燃料项目中撤出价值超过420亿美元的投资（指标4.2.4）。在学术界，从2007年到2019年，有关健康和气候变化的原创性研究出版物增长了9倍（指标5.3）。

这些转变也催生了更广泛的公众讨论。从2018年到2019年，全球媒体对健康和气候变化的报道增加了96%，超过了对气候变化报道的总体增长幅度，达到了迄今为止的最高值（指标5.1）。正如在环境卫生、个人卫生以及烟草控制方面取得的进展一样，得益于过去五年来越来越多卫生专业人员的持续参与，全球应对气候变化的关键缺口正在缩小。

未来五年：对两个公共卫生危机的协同应对

2020年12月12日将是2015年《巴黎协定》的周年纪念日，各国将更新本国承诺并每五年进行一次审评。接下来的五年将是关键时期。为了实现1.5度温升控制目标并将温升幅度保持在“远低于2℃”的水平，需要在2030年前的短短10年时间里，把温室气体排放量从目前的每年56 $GtCO_2e$降至25 $GtCO_2e$。实际上，这就要求每年减排7.6%，相当于目前各国政府减排目标力度的五倍。如果今后五年不采取进一步的干预措施，每年所需的减排量将增加到15.4%，1.5度温升控制目标将难以实现。

未来五年，新冠肺炎疫情及其应对将成为加速

Martin School, University of Oxford, Oxford, UK (L Georgeson PhD, M Springmann PhD); Department of Health Sciences, University of York, York, UK (S Golder PhD, Prof H Graham PhD, P Lampard PhD); CGIAR Research Program on Agriculture for Human Nutrition and Health, International Livestock Research Institute, Nairobi, Kenya (D Grace PhD); School of Public Health and Administration, Universidad Peruana Cayetano Heredia, Lima, Peru (S Hartinger PhD); Department of Epidemiology, Harvard TH Chan School of Public Health, Harvard University, Boston, MA, USA (M P Jimenez PhD); Department of Environmental Health, Boston University, Boston, MA, USA (Prof P L Kinney ScD); Health and Environment International Trust, Nelson, New Zealand (Prof T Kjellstrom PhD); School of Global Studies, University of Sussex, Falmer, UK (Prof D Kniveton PhD); School of Health (B Lemke PhD) and Department of Arts, Media and Digital Technologies (M Otto MEng), Nelson Marlborough Institute of Technology, Nelson, New Zealand; Gangarosa Department of Environmental Health (L Shi ScD), Rollins School of Public Health, Emory University, Atlanta, GA, USA (Prof Y Liu PhD, B Vu MSPH); Center on Global Energy Policy, Columbia University, New York, NY, USA (M Lott PhD); Department of Genetics and Microbiology, Universitat Autònoma de Barcelona, Barcelona, Spain (Prof J Martinez-Urtaza PhD); Center for Energy Markets, Technical University of Munich, Munich, Germany (L McAllister PhD); Data Science Lab, Hertie School, Berlin, Germany (Prof S Jankin Mikhaylov PhD, Prof S Munzert PhD); School of Geography, University of Melbourne, Melbourne, VIC, Australia (C McMichael PhD); Preventive Medicine and Public Health Research Center, Psychosocial Health Research Institute, Iran University of Medical Sciences, Tehran, Iran (Prof M Moradi-Lakeh MD);

应对气候变化的新背景。由于疫情大流行和气候变化造成的生命损失都达到了数十万的级别，潜在的经济成本均高达万亿级别，并且预计在未来几年内还将继续产生更深远的影响，因此必须认真制定这两种公共卫生危机的应对措施，并将其紧密联系起来。在2020年5月，超过4000万卫生专业人员致函全球领导人，强调了上述观点。这些卫生专业人员非常适合在这两个问题之间发挥桥梁作用。应对新冠肺炎的具体方法可能有助于了解应如何协同应对这两种公共卫生危机。

首先，在紧急情况下，应高度重视快速诊断和全面评估情况。同样，需要开展进一步工作来理解这个问题，包括：哪些人群既容易受到疫情影响又容易受到气候变化影响；全球和各国做出了何种反应和调整，由此带来了怎样的健康和环境影响；以及应保留这些转变的哪些方面以支持长期可持续发展。其次，回顾和管理康复和治疗方案，并仔细考虑治疗方案的潜在副作用、护理目标和患者的终生健康。如果经济复苏方案中优先考虑了过时的依赖化石燃料的能源和交通方式，将会产生计划之外的副作用，无谓地增加每年因空气污染而死亡的人数。与之相反，增加有利于健康的投资，例如投资可再生能源和清洁空气、主动交通基础设施和体育锻炼设施，以及建设具有恢复力和气候智能型的医疗保健体系，最终将更加有效。

最后，应将关注点转向次生灾害预防和长期复苏，力求使疾病的长远影响降至最低，并防止其复发。许多预防突发事件（如疫情）所采取的措施，与应对极端天气和气候变化所需的措施是相似的。这些措施包括：识别脆弱人群、评估公共卫生系统的能力、制定和投资防备措施以及强调社区恢复力和公平性。实际上，如果忽略当前和未来气候变化的影响，当前为应对未来疫情所做的努力很有可能会被削弱。

对于疫情大流行和气候变化这两种公共卫生危机，都必须按照与威胁程度相称的紧迫性采取行动，遵照最佳的科学发现来制定应对策略，同时要确保信息沟通的清晰和一致。这场疫情将会影响各国政府未来五年的经济、社会和环境政策，而这五年对于能否实现“远低于2℃”的温升控制目标至关重要。除非全球新冠肺炎疫情的复苏措施与应对气候变化的措施保持一致，否则全球将无法实现《巴黎协定》的目标，在短期和长期内都会损害公众的健康。

European Centre for Environment and Human Health (K Morrissey PhD) **and Medical and Health School** (Prof D Pencheon MSc), **University of Exeter, Exeter, UK; Medical Research Council Centre for Global Infectious Disease Analysis, Department of Infectious Disease Epidemiology, Imperial College London, London, UK** (K A Murray PhD); **Medical Research Council Unit The Gambia at London School of Hygiene & Tropical Medicine, Bakau, The Gambia** (K A Murray); **Iranian Fisheries Science Research Institute, Agricultural Research, Education, and Extension Organisation, Tehran, Iran** (F Owfi PhD, M Rabbaniha PhD); **Department of Civil and Structural Engineering, University of Sheffield, Sheffield, UK** (R Quinn PhD); **Scientific Assessment Section, European Centre for Disease Prevention and Control, Solna, Sweden** (Prof J C Semenza PhD); **WHO-WMO Joint Climate and Health Office, Geneva, Switzerland** (J Shumake-Guillemot DrPH); **Institute of Tropical Aquaculture and Fisheries, Universiti Malaysia Terengganu, Kuala Terengganu, Malaysia** (Prof M Tabatabaei PhD); **Department of Civil Engineering, Tampere University, Tampere, Finland** (J Taylor PhD); **and Department of Electronics and Computer Science, CRETUS Institute, Universidade de Santiago de Compostela, Santiago, Spain** (J Triñanes PhD)

Correspondence to: Dr Nick Watts, Institute for Global Health, University College London, London W1T 4TJ, UK **nicholas.watts@ucl.ac.uk**

For **Peter Byass' obituary** see **Obituary** *Lancet* 2020; **396:** 752

www.thelancet.com **Published online December 2, 2020 https://doi.org/10.1016/S0140-6736(20)32290-X**
