# Supplementary material for: The 2020 report of the Lancet Countdown on health and climate change: responding to converging crises
Source: Lancet. Author manuscript; Available in PMC 2024 Nov 15. (PMC7616803; doi:10.1016/S0140-6736(20)32290-X)
Supplement: French translation of the abstract [file EMS200002-supplement-French_translation_of_the_abstract.pdf]

THE LANCET

Supplementary appendix

This translation in French was submitted by the authors and we reproduce it as supplied. It has not been peer reviewed. *The Lancet*'s editorial processes have only been applied to the original in English, which should serve as reference for this manuscript.

Cette traduction en français a été proposée par les auteurs et nous l'avons reproduite telle quelle. Elle n'a pas été examinée par des pairs. Les processus éditoriaux du *Lancet* n'ont été appliqués qu'à l'original en anglais et c'est cette version qui doit servir de référence pour ce manuscrit.

Supplement to: Watts N, Amann M, Arnell N, et al. The 2020 report of The *Lancet* Countdown on health and climate change: responding to converging crises. *Lancet* 2020; published online Dec 2. http://dx.doi.org/10.1016/S0140-6736(20)32290-X.

Le Rapport 2020 du Lancet Countdown sur la santé et le changement climatique : Répondre aux crises convergentes

Nick Watts, Markus Amann, Nigel Arnell, Sonja Ayeb-Karlsson, Jessica Beagley, Kristine Belesova, Maxwell Boykoff, Peter Byass, Wenjia Cai, Diarmid Campbell-Lendrum, Stuart Capstick, Jonathan Chambers, Samantha Coleman, Carole Dalin, Meaghan Daly, Niheer Dasandi, Shouro Dasgupta, Michael Davies, Claudia Di Napoli, Paula Dominguez-Salas, Paul Drummond, Robert Dubrow, Kristie L Ebi, Matthew Eckelman, Paul Ekins, Luis E Escobar, Lucien Georgeson, Su Golder, Delia Grace, Hilary Graham, Paul Haggar, Ian Hamilton, Stella Hartinger, Jeremy Hess, Shih-Che Hsu, Nick Hughes, Slava Jankin Mikhaylov, Marcia P Jimenez, Ilan Kelman, Harry Kennard, Gregor Kiesewetter, Patrick L Kinney, Tord Kjellstrom, Dominic Kniveton, Pete Lampard, Bruno Lemke, Yang Liu, Zhao Liu, Melissa Lott, Rachel Lowe, Jaime Martinez-Urtaza, Mark Maslin, Lucy McAllister, Alice McGushin, Celia McMichael, James Milner, Maziar Moradi-Lakeh, Karyn Morrissey, Simon Munzert, Kris A Murray, Tara Neville, Maria Nilsson, Maquins Odhiambo Sewe, Tadj Oreszczyn, Matthias Otto, Fereidoon Owfi, Olivia Pearman, David Pencheon, Ruth Quinn, Mahnaz Rabbaniha, Elizabeth Robinson, Joacim Rocklöv, Marina Romanello, Jan C Semenza, Jodi Sherman, Liuhua Shi, Marco Springmann, Meisam Tabatabaei, Jonathon Taylor, Joaquin Triñanes, Joy Shumake-Guillemot, Bryan Vu, Paul Wilkinson, Matthew Winning, Peng Gong*, Hugh Montgomery*, Anthony Costello*

Résumé

Le Lancet Countdown est une collaboration internationale, qui a été établie pour fournir un système de surveillance mondial indépendant dédié au suivi du profil de santé émergent du changement climatique.

Le Rapport 2020 présente 43 indicateurs répartis en cinq sections : les impacts du changement climatique et l'exposition et la vulnérabilité au changement climatique; l'adaptation, la planification et la résilience pour la santé ; les mesures d'atténuation et les avantages connexes pour la santé ; l'économie et les finances ; et, l'engagement public et politique. Ce rapport représente les conclusions et le consensus des 35 principales institutions universitaires et agences des Nations Unies qui composent le Lancet Countdown, et s'appuie sur l'expertise de climatologues, de géographes et d'ingénieurs ; d'experts en énergie, en alimentation et en transports ; et, d'économistes, de spécialistes des sciences sociales et politiques, de spécialistes des données, de professionnels de la santé publique et de médecins.

Le profil de santé émergent du changement climatique

Il y a cinq ans, les pays se sont engagés à limiter le réchauffement à « bien en dessous de 2 °C », dans le cadre de l'historique Accord de Paris. Cinq ans plus tard, les émissions mondiales de CO_2 continuent d'augmenter de façon régulière, sans réduction convaincante ou durable, et en conséquence la température moyenne mondiale montre un réchauffement de 1,2 °C. En effet, depuis 2015, le monde a vécu les cinq années les plus chaudes jamais enregistrées.

Le changement climatique a déjà significativement altéré les déterminants sociaux et environnementaux sous-jacents de la santé au niveau mondial. Les différents indicateurs suivis par la collaboration dans tous les domaines *d'impacts, d'expositions et de vulnérabilités* se détériorent. Des tendances préoccupantes et souvent en accélération sont observées pour chacun des symptômes humains du changement climatique surveillés ; avec les indicateurs 2020 présentant les perspectives les plus inquiétantes rapportées depuis la création du Lancet Countdown.

Ces effets sont souvent inégaux, affectant de manière disproportionnée les populations qui ont le moins contribué au problème. Cela met en évidence une question plus profonde de justice, selon laquelle le changement climatique interagit avec les inégalités sociales et économiques existantes et exacerbe les tendances de longue date à l'intérieur et entre les pays. Un examen des causes du changement climatique révèle des problèmes similaires, et de nombreuses pratiques et politiques générant de hauts niveaux d'émissions de carbone entraînent une mauvaise qualité de l'air, une mauvaise qualité des aliments et une mauvaise qualité des logements, ce qui nuit de manière disproportionnée à la santé des populations défavorisées.

Les populations vulnérables ont été exposées à 475 millions d'épisodes caniculaires supplémentaires au niveau mondial, ce qui, à son tour, a été reflété dans des excès de morbidité et de mortalité. Au cours des 20 dernières années, il y a eu une augmentation de 53,7 % de la mortalité liée à la chaleur chez les personnes de plus de 65 ans, atteignant un total de 296 000 décès en 2018 (Indicateurs 1.1.2 et 1.1.3). Le coût élevé en termes de vies humaines et de souffrances est associé à des impacts sur la production économique, avec en 2019, plus de 302 milliards d'heures de capacité potentielle de main-d'œuvre perdues (Indicateur 1.1.4). L'Inde et l'Indonésie sont parmi les pays les plus touchés, enregistrant des pertes potentielles de capacité de main-d'œuvre équivalant à 4 à 6 % de leur produit intérieur brut annuel (Indicateur 4.1.3). En Europe, en 2018, le coût monétisé de la mortalité liée à la chaleur équivalait à 1,2 % du revenu national brut régional, soit le revenu moyen de 11 millions de citoyens européens (Indicateur 4.1.2).

En ce qui concerne les conditions météorologiques extrêmes, les progrès de la science du climat permettent de plus en plus une meilleure précision et certitude dans l'attribution. Des études de 2015 à 2020 ont montré les

Published Online
December 2, 2020
https://doi.org/10.1016/S0140-6736(20)32290-X

*Co-chairs

Institute for Global Health (N Watts MA, J Beagley BA, S Coleman MSE, Prof I Kelman PhD, A McGushin MSc, M Romanello PhD), **Office of the Vice Provost for Research** (Prof A Costello FmedSci), **Energy Institute** (S-C Hsu MSc, I Hamilton PhD, H Kennard PhD, Prof T Oreszczyn PhD), **Institute for Sustainable Resources** (C Dalin PhD, P Drummond MSc, Prof P Ekins PhD, N Hughes PhD, M Winning PhD), **Institute for Environmental Design and Engineering** (Prof M Davies PhD), **Department of Geography** (Prof M Maslin PhD), **and Institute for Human Health and Performance** (Prof H Montgomery MD), **University College London, London, UK; Air Quality and Greenhouse Gases Program, International Institute for Applied Systems Analysis, Laxenburg, Austria** (M Amann PhD, G Kiesewetter PhD); **Department of Meteorology** (Prof N W Arnell PhD) **and School of Agriculture, Policy, and Development** (C Di Napoli PhD, Prof E Robinson PhD), **University of Reading, Reading, UK; Institute for Environment and Human Security, United Nations University, Bonn, Germany** (S Ayeb-Karlsson PhD); **Centre on Climate Change and Planetary Health** (K Belesova PhD), **Department**

of Population Health (P Dominguez-Salas PhD), Centre for Mathematical Modelling of Infectious Diseases (R Lowe PhD), and Department of Public Health, Environments, and Society (J Milner PhD, Prof P Wilkinson FRCP), London School of Hygiene & Tropical Medicine, London, UK; Environmental Studies Program, University of Colorado Boulder, Boulder, CO, USA (Prof M Boykoff PhD, O Pearman MEM); Department of Epidemiology and Global Health (Prof P Byass PhD, Prof M Nilsson PhD) and Department of Public Health and Clinical Medicine (M O Sewe PhD, Prof J Rocklöv PhD), Umeå University, Umeå, Sweden; Department of Earth System Science, Tsinghua University, Beijing, China (W Cai PhD, Prof P Gong PhD, Z Liu PhD); Environment, Climate Change and Health Department, World Health Organization, Geneva, Switzerland (D Campbell-Lendrum DPhil, T Neville MSc); School of Psychology, Cardiff University, Cardiff, UK (S Capstick PhD, P Haggar PhD); Institute for Environmental Sciences, University of Geneva, Geneva, Switzerland (J Chambers PhD); Department of Environmental Studies, University of New England, Biddeford, ME, USA (M Daly PhD); School of Government, University of Birmingham, Birmingham, UK (N Dasandi PhD); Centro Euro-Mediterraneo sui Cambiamenti Climatici, Venice, Italy (S Dasgupta PhD); Yale Center on Climate Change and Health (Prof R Dubrow PhD) and Department of Anesthesiology (J Sherman MD), Yale University, New Haven, CT, USA; Department of Global Health (Prof K L Ebi PhD) and Center for Health and the Global Environment (J Hess MD), University of Washington, Seattle, WA, USA; Department of Civil & Environmental Engineering, Northeastern University, Boston, MA, USA (M Eckelman PhD); Department of Fish and Wildlife Conservation, Virginia Polytechnic Institute and State University, Blacksburg, VA, USA (L E Escobar PhD); Oxford

empreintes du changement climatique dans 76 inondations, sécheresses, tempêtes et anomalies de température (Indicateur 1.2.3). En outre, il y a eu une augmentation du nombre de jours où les personnes ont été exposées à un risque très élevé ou extrêmement élevé d'incendies de forêt entre 2001 et 2004 et 2016 et 2019 dans 114 pays (Indicateur 1.2.1). De même, 67 % des villes du monde interrogées s'attendent à ce que le changement climatique compromette gravement leurs actifs et leurs infrastructures en matière de santé publique (Indicateur 2.1.3).

Le changement climatique a des effets en aval, affectant des systèmes environnementaux plus larges, qui à leur tour nuisent à la santé humaine. La sécurité alimentaire mondiale est menacée par la hausse des températures et l'augmentation de la fréquence des épisodes extrêmes. Le rendement potentiel mondial des principales cultures a diminué de 1,8 à 5,6 % entre 1981 et 2019 (Indicateur 1.4.1). La propicité du climat à la transmission de maladies infectieuses a augmenté rapidement depuis les années 1950, avec une augmentation de 15 % de la propicité du climat à la dengue causée par *Aedes albopictus* en 2018 et des augmentations régionales pour le paludisme et les bactéries *Vibrio* (Indicateur 1.3.1). Selon les prévisions, sur la base des populations actuelles, entre 145 millions et 565 millions de personnes risquent de subir des innondations en raison de l'élévation du niveau de la mer (Indicateur 1.5).

Malgré ces signes clairs et croissants, la réponse mondiale au changement climatique a été faible et les efforts nationaux continuent à être bien en deçà des engagements pris dans l'Accord de Paris. L'intensité en carbone du système énergétique mondial est restée pratiquement inchangée pendant 30 ans, avec une consommation mondiale de charbon ayant augmentée de 74 % pendant cette période (Indicateurs 3.1.1 et 3.1.2). La réduction de l'utilisation mondiale de charbon qui avait été observée depuis 2013 s'est maintenant inversée au cours des deux dernières années consécutives. L'utilisation de charbon a augmenté de 1,7 % de 2016 à 2018. Le fardeau sanitaire qui en résulte est considérable : plus d'un million de décès surviennent chaque année en raison de la pollution de l'air par les centrales au charbon, et en 2018, environ 390 000 de ces décès résultaient de la pollution particulaire (Indicateur 3.3). La réponse dans le secteur alimentaire et agricole a été tout aussi préoccupante. Les émissions provenant du bétail ont augmenté de 16 % entre 2000 et 2017, avec 93 % des émissions provenant des ruminants (Indicateur 3.5.1). De même, les régimes alimentaires de plus en plus malsains observés sont de plus en plus courants dans le monde, avec une consommation excessive de viande rouge contribuant à quelque 990 000 décès en 2017 (Indicateur 3.5.2). Cinq ans après la date à laquelle les pays sont parvenus à un accord à Paris, un nombre inquiétant d'indicateurs montre un renversement précoce mais soutenu des tendances jusqu'alors positives identifiées dans les rapports précédents (Indicateurs 1.3.2, 3.1.2 et 4.2.3).

Une réponse croissante des professionnels de la santé

Malgré le peu d'amélioration à l'échelle de l'économie, des avancées relatives ont été réalisées dans plusieurs secteurs clés. La capacité d'énergie renouvelable a augmenté de 21 % par an entre 2010 et 2017 et la production d'électricité à faible émission de carbone était responsable de 28 % des capacités en Chine en 2017 (Indicateur 3.1.3). Cependant, les indicateurs présentés dans le rapport 2020 du Lancet Countdown suggèrent que certains des progrès les plus considérables ont été observés dans l'élan croissant de l'engagement des professionnels de la santé dans le changement climatique, et ce *à l›échelle mondiale. Les médecins, les infirmi*ers et la profession au sens large ont un rôle central dans l'adaptation et l'atténuation du système de santé, dans la compréhension et la maximisation des avantages pour la santé de toute intervention et dans la communication de la nécessité d'une réponse plus rapide.

Dans le cas de l'adaptation des systèmes de santé nationaux, ce changement est en cours. De manière impressionnante, les services de santé de 86 pays sont désormais connectés à leurs homologues des services météorologiques pour aider à la planification de l'adaptation sanitaire (Indicateur 2.2). Au moins 51 pays ont élaboré des plans nationaux d'adaptation sanitaire, et les dépenses mondiales en matière d'adaptation sanitaire ont atteint 5,3 % de toutes les dépenses d'adaptation en 2018 et 2019, atteignant 18,4 milliards de dollars américains en 2019 (Indicateurs 2.1.1 et 2.4).

Le secteur de la santé, qui était responsable de 4,6 % des émissions mondiales de gaz à effet de serre en 2017, est en train d'adopter des mesures précoces mais importantes pour réduire ses propres émissions (Indicateur 3.6). Au Royaume-Uni, le Service National de Santé (National Health Service) a déclaré son ambition de fournir un « service de santé zéro émission nette » dès que possible, en s'appuyant sur une décennie de progrès impressionnants dans la réduction des émissions dans la réalisation des soins de 57 % depuis 1990 et de 22 % en prenant en compte la chaîne d'approvisionnement et les responsabilités plus larges du service. Ailleurs, le Département de la santé de l'Australie-Occidentale a utilisé sa *Loi de 2016 sur la santé publique* pour mener la première enquête sur le climat et la santé en Australie, et le ministère fédéral allemand de la Santé a créé un département dédié à la protection de la santé et à la durabilité, responsable des questions liées au climat. Ces progrès sont de mieux en mieux répartis dans le monde, 73 % des pays font explicitement référence à la santé et au bien-être dans leurs engagements nationaux au titre de l'Accord de Paris, dont 100 % des pays d'Asie du Sud-Est et de la

www.thelancet.com Published online December 2, 2020 https://doi.org/10.1016/S0140-6736(20)32290-X

Méditerranée orientale (Indicateur 5.4). De façon similaire, les pays les moins avancés et les petits États insulaires en développement assurent un leadership mondial croissant dans le cadre du débat général des Nations Unies sur les liens entre la santé et le changement climatique (Indicateur 5.4).

Les professionnels de santé et leurs associations réagissent également : les établissements de santé s'engageant à céder plus de 42 milliards de dollars américains d'actifs provenant de combustibles fossiles (Indicateur 4.2.4). Dans le milieu universitaire, le nombre de publications de recherches originales sur la santé et le changement climatique a été multiplié par neuf entre 2007 et 2019 (Indicateur 5.3).

Ces changements se retrouvent dans le discours public plus large. De 2018 à 2019, la couverture en matière de santé et de changement climatique dans les médias a augmenté de 96 % dans le monde, dépassant la couverture accrue du changement climatique dans son ensemble et atteignant le niveau le plus élevé observé à ce jour (Indicateur 5.1). Tout comme cela a été le cas pour les progrès en matière d'assainissement et d'hygiène et de lutte antitabac, l'engagement croissant et soutenu des professions de la santé au cours des cinq dernières années commence maintenant à combler une lacune cruciale dans la réponse mondiale au changement climatique.

Les cinq prochaines années : Une réponse conjointe à deux crises de santé publique

Le 12 décembre 2020 marquera l'anniversaire de l'Accord de Paris de 2015, les pays ayant prévu une mise à jour et une révision de leurs engagements nationaux tous les cinq ans. Ces cinq prochaines années seront cruciales. Pour atteindre l'objectif de 1,5 °C et maintenir l'élévation de température « bien en dessous de 2 °C », les 56 gigatonnes de CO_2e (Gt CO_2e) actuellement émis annuellement devront chuter à 25 Gt CO_2e en seulement 10 ans (d'ici 2030). De façon concrète, cela nécessite une réduction de 7,6 % chaque année, ce qui représente une multiplication par cinq des ambitions actuelles des gouvernements nationaux. Sans intervention supplémentaire au cours des cinq prochaines années, les réductions requises passeront à 15,4 % chaque année, ce qui rendra l'objectif de 1,5 °C hors d'atteinte.

La nécessité d'accélérer les efforts pour lutter contre le changement climatique au cours des cinq prochaines années sera contextualisée par les impacts et la réponse globale à la pandémie de Covid-19. Avec les pertes en vies humaines dues à la pandémie et au changement climatique mesurées en centaines de milliers, les coûts économiques potentiels mesurés en milliers de milliards et les conséquences plus larges qui devraient se poursuivre pendant des années à venir, les mesures prises pour faire face à ces deux crises de santé publique doivent être soigneusement examinées et étroitement liées. En mai 2020, plus de 40 millions de professionnels de la santé ont écrit aux dirigeants mondiaux pour souligner ce point. Ces professionnels de la santé sont bien placés pour servir de pont entre les deux problèmes, et l'examen de l'approche clinique de la prise en charge d'un patient atteint de la COVID-19 peut être utile pour comprendre les façons dont ces deux crises de santé publique devraient être traitées conjointement.

Premièrement, dans un contexte grave, une priorité élevée doit être accordée au diagnostic rapide et à l'évaluation complète de la situation. De même, des travaux supplémentaires sont nécessaires pour comprendre le problème, notamment : quelles populations sont vulnérables à la fois à la pandémie et au changement climatique ; comment les économies mondiales et nationales ont réagi et se sont adaptées, et les conséquences sur la santé et l'environnement qui en résultent ; et quels aspects de ces changements devraient être conservés pour soutenir le développement durable à plus long terme. Deuxièmement, les options de réanimation et de traitement appropriées doivent être examinées et administrées, en tenant soigneusement compte de tout effet secondaire potentiel, des objectifs de soins et de la santé à vie du patient. Les programmes de relance économique qui accordent la priorité aux formes désuètes d'énergie et de transport à forte consommation de combustibles fossiles auront des effets secondaires involontaires, ajoutant inutilement aux sept millions de personnes qui meurent chaque année à cause de la pollution atmosphérique. Au lieu de cela, les investissements dans les impératifs de santé, tels que les énergies renouvelables et l'air propre, les infrastructures de voyage actives et l'activité physique, et les soins de santé résilients et climato-intelligents seront, au final, plus efficaces.

Enfin, l'attention se porte sur la prévention secondaire et le rétablissement à long terme, cherchant à minimiser les effets permanents de la maladie et à prévenir sa récurrence. Bon nombre des étapes de la préparation à des chocs imprévus tels qu'une pandémie sont similaires à celles nécessaires pour s'adapter aux conditions météorologiques extrêmes et aux nouvelles menaces attendues du changement climatique. Ces étapes comprennent la nécessité d'identifier les populations vulnérables, d'évaluer la capacité des systèmes de santé publique, de développer et d'investir dans des mesures de préparation et de mettre l'accent sur la résilience et l'équité des communautés. En effet, si nous ne tenons pas compte des impacts actuels et futurs du changement climatique, les efforts de préparation aux futures pandémies seront probablement sapés.

À chaque étape et dans les deux cas, agir avec un niveau d›urgence proportionné à l›ampleur de la menace, adhérer aux meilleures connaissances scientifiques disponibles et assurer des communications claires et cohérentes sont des éléments primordiaux. Les conséquences de la pandémie contextualiseront les

Martin School, University of Oxford, Oxford, UK (L Georgeson PhD, M Springmann PhD); **Department of Health Sciences, University of York, York, UK** (S Golder PhD, Prof H Graham PhD, P Lampard PhD); **CGIAR Research Program on Agriculture for Human Nutrition and Health, International Livestock Research Institute, Nairobi, Kenya** (D Grace PhD); **School of Public Health and Administration, Universidad Peruana Cayetano Heredia, Lima, Peru** (S Hartinger PhD); **Department of Epidemiology, Harvard TH Chan School of Public Health, Harvard University, Boston, MA, USA** (M P Jimenez PhD); **Department of Environmental Health, Boston University, Boston, MA, USA** (Prof P L Kinney ScD); **Health and Environment International Trust, Nelson, New Zealand** (Prof T Kjellstrom PhD); **School of Global Studies, University of Sussex, Falmer, UK** (Prof D Kniveton PhD); **School of Health** (B Lemke PhD) **and Department of Arts, Media and Digital Technologies** (M Otto MEng), **Nelson Marlborough Institute of Technology, Nelson, New Zealand; Gangarosa Department of Environmental Health** (L Shi ScD), **Rollins School of Public Health, Emory University, Atlanta, GA, USA** (Prof Y Liu PhD, B Vu MSPH); **Center on Global Energy Policy, Columbia University, New York, NY, USA** (M Lott PhD); **Department of Genetics and Microbiology, Universitat Autònoma de Barcelona, Barcelona, Spain** (Prof J Martinez-Urtaza PhD); **Center for Energy Markets, Technical University of Munich, Munich, Germany** (L McAllister PhD); **Data Science Lab, Hertie School, Berlin, Germany** (Prof S Jankin Mikhaylov PhD, Prof S Munzert PhD); **School of Geography, University of Melbourne, Melbourne, VIC, Australia** (C McMichael PhD); **Preventive Medicine and Public Health Research Center, Psychosocial Health Research Institute, Iran University of Medical Sciences, Tehran, Iran** (Prof M Moradi-Lakeh MD);

politiques économiques, sociales et environnementales des gouvernements au cours des cinq prochaines années, une période cruciale pour déterminer si les températures resteront « bien en dessous de 2 °C ». À moins que la reprise mondiale post- COVID-19 ne soit alignée sur la réponse au changement climatique, le monde ne parviendra pas à atteindre l'objectif fixé dans l'Accord de Paris, ce qui nuira à la santé publique à la fois à court et à long terme.

European Centre for Environment and Human Health (K Morrissey PhD) **and Medical and Health School** (Prof D Pencheon MSc), **University of Exeter, Exeter, UK; Medical Research Council Centre for Global Infectious Disease Analysis, Department of Infectious Disease Epidemiology, Imperial College London, London, UK** (K A Murray PhD)**; Medical Research Council Unit The Gambia at London School of Hygiene & Tropical Medicine, Bakau, The Gambia** (K A Murray)**; Iranian Fisheries Science Research Institute, Agricultural Research, Education, and Extension Organisation, Tehran, Iran** (F Owfi PhD, M Rabbaniha PhD)**; Department of Civil and Structural Engineering, University of Sheffield, Sheffield, UK** (R Quinn PhD)**; Scientific Assessment Section, European Centre for Disease Prevention and Control, Solna, Sweden** (Prof J C Semenza PhD)**; WHO-WMO Joint Climate and Health Office, Geneva, Switzerland** (J Shumake-Guillemot DrPH)**; Institute of Tropical Aquaculture and Fisheries, Universiti Malaysia Terengganu, Kuala Terengganu, Malaysia** (Prof M Tabatabaei PhD)**; Department of Civil Engineering, Tampere University, Tampere, Finland** (J Taylor PhD)**; and Department of Electronics and Computer Science, CRETUS Institute, Universidade de Santiago de Compostela, Santiago, Spain** (J Triñanes PhD)

Correspondence to: Dr Nick Watts, Institute for Global Health, University College London, London W1T 4TJ, UK **nicholas.watts@ucl.ac.uk**

For **Peter Byass' obituary** see **Obituary** *Lancet* 2020; **396:** 752

www.thelancet.com **Published online December 2, 2020 https://doi.org/10.1016/S0140-6736(20)32290-X**
