# Supplementary material for: The 2020 report of the Lancet Countdown on health and climate change: responding to converging crises
Source: Lancet. Author manuscript; Available in PMC 2024 Nov 15. (PMC7616803; doi:10.1016/S0140-6736(20)32290-X)
Supplement: German translation of the abstract [file EMS200002-supplement-German_translation_of_the_abstract.pdf]

THE LANCET

Supplementary appendix

This translation in German was submitted by the authors and we reproduce it as supplied. It has not been peer reviewed. *The Lancet*'s editorial processes have only been applied to the original in English, which should serve as reference for this manuscript.

Diese Übersetzung in deutscher Sprache wurde von den Autoren eingereicht und wir reproduzieren sie wie vorgelegt. Die Übersetzung wurde nicht von Experten begutachtet. Die redaktionellen Prozesse von The Lancet wurden nur auf das Original in englischer Sprache angewendet, das als Referenz für dieses Manuskript dienen soll.

Supplement to: Watts N, Amann M, Arnell N, et al. The 2020 report of The *Lancet* Countdown on health and climate change: responding to converging crises. *Lancet* 2020; published online Dec 2. http://dx.doi.org/10.1016/S0140-6736(20)32290-X.

Der Lancet Countdown 2020 zu Gesundheit und Klimawandel: Antworten auf sich überlagernde Krisen

Nick Watts, Markus Amann, Nigel Arnell, Sonja Ayeb-Karlsson, Jessica Beagley, Kristine Belesova, Maxwell Boykoff, Peter Byass, Wenjia Cai, Diarmid Campbell-Lendrum, Stuart Capstick, Jonathan Chambers, Samantha Coleman, Carole Dalin, Meaghan Daly, Niheer Dasandi, Shouro Dasgupta, Michael Davies, Claudia Di Napoli, Paula Dominguez-Salas, Paul Drummond, Robert Dubrow, Kristie L Ebi, Matthew Eckelman, Paul Ekins, Luis E Escobar, Lucien Georgeson, Su Golder, Delia Grace, Hilary Graham, Paul Haggar, Ian Hamilton, Stella Hartinger, Jeremy Hess, Shih-Che Hsu, Nick Hughes, Slava Jankin Mikhaylov, Marcia P Jimenez, Ilan Kelman, Harry Kennard, Gregor Kiesewetter, Patrick L Kinney, Tord Kjellstrom, Dominic Kniveton, Pete Lampard, Bruno Lemke, Yang Liu, Zhao Liu, Melissa Lott, Rachel Lowe, Jaime Martinez-Urtaza, Mark Maslin, Lucy McAllister, Alice McGushin, Celia McMichael, James Milner, Maziar Moradi-Lakeh, Karyn Morrissey, Simon Munzert, Kris A Murray, Tara Neville, Maria Nilsson, Maquins Odhiambo Sewe, Tadj Oreszczyn, Matthias Otto, Fereidoon Owfi, Olivia Pearman, David Pencheon, Ruth Quinn, Mahnaz Rabbaniha, Elizabeth Robinson, Joacim Rocklöv, Marina Romanello, Jan C Semenza, Jodi Sherman, Liuhua Shi, Marco Springmann, Meisam Tabatabaei, Jonathon Taylor, Joaquin Triñanes, Joy Shumake-Guillemot, Bryan Vu, Paul Wilkinson, Matthew Winning, Peng Gong, Hugh Montgomery*, Anthony Costello**

- Zusammenfassung -

Der Lancet Countdown ist eine internationale Kooperation; sie wurde gegründet, um ein unabhängiges, globales Monitoringsystem zur Verfügung zu stellen, das die deutlicher werdenden gesundheitlichen Folgen des Klimawandels dokumentiert.

Der Bericht von 2020 legt Daten zu 43 Indikatoren in fünf Themenbereichen vor: die Auswirkungen des Klimawandels, Exposition und Anfälligkeit; Anpassung, Planung und Resilienz bezogen auf die Gesundheit; Klimaschutzmaßnahmen und gesundheitliche Co-Benefits; Wirtschaft und Finanzen sowie öffentliches und politisches Engagement. Dieser Bericht gibt die Ergebnisse und den Konsens der 35 führenden akademischen Institutionen und UN-Organisationen wieder, die gemeinsam den Lancet Countdown bilden. Er stützt sich auf das Fachwissen von Klimaforschern, Geographen und Ingenieuren, von Energie-, Lebensmittel- und Verkehrsexperten, sowie von Ökonomen, Sozial- und Politikwissenschaftlern, Datenwissenschaftlern, Fachleuten des öffentlichen Gesundheitswesens und Ärzten.

Welche Auswirkungen auf die Gesundheit hat der Klimawandel?

Vor fünf Jahren haben sich viele Länder im Rahmen des wegweisenden Pariser Abkommens verpflichtet, die Erwärmung auf „deutlich unter 2° C“ zu begrenzen. Fünf Jahre später steigen die globalen CO2-Emissionen weiter stetig an, ohne dass sich eine überzeugende oder nachhaltige Verringerung abzeichnen würde. Die Folge ist ein Anstieg der globalen Durchschnittstemperatur um 1,2° C. Tatsächlich sind seit 2015 die fünf heißesten Jahre seit Beginn der Aufzeichnungen zu verzeichnen.

Die Klimaveränderungen haben weltweit bereits zu erheblichen Verschiebungen der sozialen und ökologischen Determinanten von Gesundheit geführt. Sämtliche Indikatoren, welche der Lancet Countdown zu den Auswirkungen des Klimawandels, seinen Gefahren und Vulnerabilitäten verfolgt, haben sich verschlechtert. Für jedes der beobachteten Symptome des Klimawandels am Menschen zeichnen sich besorgniserregende und sich häufig beschleunigende Trends ab. Dabei spiegeln die Indikatoren von 2020 die bisher besorgniserregendste Entwicklung seit Beginn des Lancet Countdown wider.

Diese Effekte sind oft ungleich verteilt und treffen überproportional jene Bevölkerungsgruppen, die am wenigsten zum Problem beigetragen haben. Das stellt die grundlegende Frage nach Gerechtigkeit, da der Klimawandel mit bestehenden sozialen und wirtschaftlichen Ungleichheiten in Wechselwirkung tritt und langfristige Trends in und zwischen Ländern verschärft. Wer nach den Ursachen des Klimawandels forscht, entdeckt ähnliche Zusammenhänge: Viele CO2-intensive Praktiken und Vorgaben führen zu einer schlechten Luftqualität, einer schlechten Lebensmittelqualität und einer schlechten Wohnqualität, was die Gesundheit benachteiligter Bevölkerungsgruppen überproportional beeinträchtigt.

So waren vulnerable Bevölkerungsgruppen weltweit zusätzlichen 475 Millionen Hitzewellenereignissen ausgesetzt, was sich wiederum in einer höheren Morbidität und Mortalität niederschlug. In den vergangenen 20 Jahren ist die Mortalität, die mit Hitze verbunden ist, bei Menschen über 65 Jahren um 53,7 Prozent gestiegen, was 2018 zu 296.000 Todesfälle führte (Indikatoren 1.1.2 und 1.1.3). Das ist ein hoher Preis an menschlichen Leben und Leiden, der auch Auswirkungen auf die Wirtschaftsleistung hat. So wurden 2019 mehr als 302 Milliarden Stunden potenzieller Arbeitskraft eingebüßt (Indikator 1.1.4). Indien und Indonesien zählen zu den am stärksten betroffenen Ländern. Ihre potenziellen Verluste an Arbeitskraft machten 4 bis 6 Prozent ihres jährlichen Bruttoinlandsprodukts aus (Indikator 4.1.3). In Europa entsprachen 2018 die monetären Kosten der hitzebedingten Sterblichkeit 1,2 Prozent des regionalen Bruttoinlandprodukts oder anders ausgedrückt dem Durchschnittseinkommen von 11 Millionen europäischen Bürgern (Indikator 4.1.2).

Was Extremwetterereignisse angeht, so ermöglichen Fortschritte in der Klimawissenschaft zunehmend eine größere Genauigkeit und Sicherheit bei der Zuordnung.

Published Online
December 2, 2020
https://doi.org/10.1016/S0140-6736(20)32290-X

*Co-chairs

Institute for Global Health (N Watts MA, J Beagley BA, S Coleman MSE, Prof I Kelman PhD, A McGushin MSc, M Romanello PhD), **Office of the Vice Provost for Research** (Prof A Costello FmedSci), **Energy Institute** (S-C Hsu MSc, I Hamilton PhD, H Kennard PhD, Prof T Oreszczyn PhD), **Institute for Sustainable Resources** (C Dalin PhD, P Drummond MSc, Prof P Ekins PhD, N Hughes PhD, M Winning PhD), **Institute for Environmental Design and Engineering** (Prof M Davies PhD), **Department of Geography** (Prof M Maslin PhD), **and Institute for Human Health and Performance** (Prof H Montgomery MD), **University College London, London, UK; Air Quality and Greenhouse Gases Program, International Institute for Applied Systems Analysis, Laxenburg, Austria** (M Amann PhD, G Kiesewetter PhD); **Department of Meteorology** (Prof N W Arnell PhD) **and School of Agriculture, Policy, and Development** (C Di Napoli PhD, Prof E Robinson PhD), **University of Reading, Reading, UK; Institute for Environment and Human Security, United Nations University, Bonn, Germany** (S Ayeb-Karlsson PhD); **Centre on Climate Change and Planetary Health** (K Belesova PhD), **Department**

of Population Health (P Dominguez-Salas PhD), Centre for Mathematical Modelling of Infectious Diseases (R Lowe PhD), and Department of Public Health, Environments, and Society (J Milner PhD, Prof P Wilkinson FRCP), London School of Hygiene & Tropical Medicine, London, UK; Environmental Studies Program, University of Colorado Boulder, Boulder, CO, USA (Prof M Boykoff PhD, O Pearman MEM); Department of Epidemiology and Global Health (Prof P Byass PhD, Prof M Nilsson PhD) and Department of Public Health and Clinical Medicine (M O Sewe PhD, Prof J Rocklöv PhD), Umeå University, Umeå, Sweden; Department of Earth System Science, Tsinghua University, Beijing, China (W Cai PhD, Prof P Gong PhD, Z Liu PhD); Environment, Climate Change and Health Department, World Health Organization, Geneva, Switzerland (D Campbell-Lendrum DPhil, T Neville MSc); School of Psychology, Cardiff University, Cardiff, UK (S Capstick PhD, P Haggar PhD); Institute for Environmental Sciences, University of Geneva, Geneva, Switzerland (J Chambers PhD); Department of Environmental Studies, University of New England, Biddeford, ME, USA (M Daly PhD); School of Government, University of Birmingham, Birmingham, UK (N Dasandi PhD); Centro Euro-Mediterraneo sui Cambiamenti Climatici, Venice, Italy (S Dasgupta PhD); Yale Center on Climate Change and Health (Prof R Dubrow PhD) and Department of Anesthesiology (J Sherman MD), Yale University, New Haven, CT, USA; Department of Global Health (Prof K L Ebi PhD) and Center for Health and the Global Environment (J Hess MD), University of Washington, Seattle, WA, USA; Department of Civil & Environmental Engineering, Northeastern University, Boston, MA, USA (M Eckelman PhD); Department of Fish and Wildlife Conservation, Virginia Polytechnic Institute and State University, Blacksburg, VA, USA (L E Escobar PhD); Oxford

Studien zwischen 2015 bis 2020 konnten den Einfluss des Klimawandels bei 76 Überschwemmungen, Dürreperioden, Stürmen und Temperaturanomalien nachweisen (Indikator 1.2.3). Zudem zeigte sich in 114 Ländern bei einem Vergleich der Zeiträume von 2001-2004 und 2016-2019 die Zunahme derjenigen Tage, an denen Menschen einem sehr hohen oder extrem hohen Waldbrandrisiko ausgesetzt waren (Indikator 1.2.1). In 67 Prozent der untersuchten Weltstädte geht man davon aus, dass der Klimawandel ihre Vermögenswerte und Infrastrukturen im Bereich der öffentlichen Gesundheit ernsthaft gefährdet (Indikator 2.1.3).

Der Klimawandel wirkt sich umfassend auf Ökosysteme aus, was im Gegenzug der menschlichen Gesundheit zusätzlich schadet. Die globale Ernährungssicherheit ist durch ansteigende Temperaturen und die Zunahme extremer Wetterereignisse bedroht. Der mögliche Ertrag der wichtigsten Feldfrüchte sank zwischen 1981 und 2019 um 1,8 bis 5,6% (Indikator 1.4.1). Für die Übertragung von Infektionskrankheiten haben sich die klimatischen Bedingungen seit den 1950er Jahren rapide verbessert, im Jahr 2018 allein für das Dengue Fiebers, das von der Tigermücke *(Aedes albopictus)* übertragen wird, um 15 Prozent. Außerdem gibt es regionale Zunahmen von Malaria und Vibrio-Bakterien (Indikator 1.3.1). Zwischen 145 und 565 Millionen Menschen sind außerdem - basierend auf den aktuellen Bevölkerungszahlen - in Zukunft von Überschwemmungen durch den Anstieg des Meeresspiegels bedroht (Indikator 1.5).

Trotz dieser klaren und eskalierenden Anzeichen ist die globale Antwort auf den Klimawandel verhalten, und die nationalen Bemühungen bleiben weit hinter den im Pariser Abkommen eingegangenen Verpflichtungen zurück. Die Kohleabhängigkeit der globalen Energieerzeugung und -nutzung ist seit 30 Jahren nahezu unverändert; der Einsatz von Kohle ist in dieser Zeit um 74 Prozent angestiegen (Indikatoren 3.1.1 und 3.1.2). Seit 2013 war ein Rückgang der weltweiten Kohlenutzung verzeichnet worden, doch dieser Trend hat sich in den vergangenen zwei Jahren umgekehrt. Der Kohleverbrauch stieg von 2016 bis 2018 um 1,7 Prozent. Die Gesundheitsbelastung dadurch ist erheblich - jedes Jahr sterben mehr als eine Million Menschen infolge der Luftverschmutzung durch Kohlekraftwerke, und rund 390.000 dieser Todesfälle waren 2018 auf Feinstaub zurückzuführen (Indikator 3.3). Die Entwicklungen im Nahrungsmittel- und Agrarsektor waren ähnlich besorgniserregend. Die Emissionen durch Nutztiere stiegen von 2000 bis 2017 um 16 Prozent, wobei 93 Prozent davon von Wiederkäuern stammten (Indikator 3.5.1). Auch wird weltweit ungesundes Essverhalten immer häufiger. Der übermäßige Verzehr von rotem Fleisch trug 2017 zu rund 990.000 Todesfällen bei (Indikator 3.5.2). Fünf Jahre nach dem Pariser Klima-Abkommen weisen beunruhigend viele Indikatoren nach anfänglich positiver Entwicklung laut früherer Berichte jetzt wieder eine negative Tendenz auf (Indikatoren 1.3.2, 3.1.2 und 4.2.3).

Die Gesundheitsberufe werden zunehmend aktiv

Trotz geringer Verbesserungen in der Gesamtwirtschaft wurden in mehreren Schlüsselsektoren relative Zuwächse erzielt: In den Jahren 2010 bis 2017 nahm die Leistung erneuerbarer Energien jährlich um 21 Prozent zu; Stromproduktion mit geringem CO2-Ausstoß nahm 2017 in China für 28% der Kapazität ein (Indikator 3.1.3).

Die im Lancet Countdown 2020 vorgestellten Indikatoren deuten jedoch darauf hin, dass einige der bedeutendsten Fortschritte sich in der weltweit wachsenden Dynamik des Engagements der Gesundheitsberufe für den Klimawandel zeigen. Ärzte, Krankenschwestern und andere Gesundheitsberufe spielen eine zentrale Rolle bei der Anpassung des Gesundheitssystems und beim Klimaschutz im Gesundheitssektor, beim Verständnis und beim Maximieren des gesundheitlichen Nutzens jeglicher Klimaschutzmaßnahmen und dabei, die Notwendigkeit eines beschleunigten Vorgehens zu vermitteln.

Die Anpassung der nationalen Gesundheitssysteme ist im Gange. Die beeindruckende Zahl von 86 Ländern hat ihre Gesundheitsdienste mit den entsprechenden Wetterdiensten verbunden, um die Planung zu verbessern (Indikator 2.2). Mindestens 51 Länder haben nationale Anpassungspläne entwickelt, und die weltweiten Ausgaben für die Adaptation im Gesundheitswesen stiegen im Zeitraum 2018-19 auf 5,3 Prozent der gesamten Anpassungskosten; 2019 erreichten sie 18,4 Mrd. USD (Indikatoren 2.1.1 und 2.4).

Der Gesundheitssektor, der 2017 selbst für 4,6 Prozent des weltweiten Ausstoßes von Klimagasen verantwortlich war, unternimmt erste aber wichtige Schritte, um seine eigenen Emissionen zu reduzieren (Indikator 3.6). Im Vereinigten Königreich hat der National Health Service (NHS) erklärt, er wolle so bald wie möglich als „Netto-Null-Gesundheitsdienst“ operieren, nachdem er auf zehn Jahre beeindruckender Fortschritte bei der Emissions-Reduzierung verweisen kann und seit 1990 die Emissionen für Behandlung und Pflege selbst um 57 Prozent gesenkt hat; bzw. um 22 Prozent, wenn man die Emissionen der Lieferketten und andere Verantwortlichkeiten des NHS mit berücksichtigt.

Anderenorts hat zum Beispiel das westaustralische Gesundheitsministerium sein Public-Health-Gesetz von 2016 dazu genutzt, die erste Erhebung Australiens zu Klima und Gesundheit durchzuführen, und das deutsche Bundesgesundheitsministerium hat eine eigene Abteilung für Gesundheitsschutz und Nachhaltigkeit eingerichtet, die für klimabezogene Angelegenheiten zuständig ist. Fortschritte dieser Art erfassen immer mehr Länder auf der ganzen Welt, wobei 73 Prozent sich in ihren nationalen Verpflichtungen im Rahmen des Pariser Abkommens ausdrücklich auf Gesundheit und Wohlbefinden beziehen. In Südostasien und im östlichen

 www.thelancet.com Published online December 2, 2020 https://doi.org/10.1016/S0140-6736(20)32290-X

Mittelmeerraum sind es sogar alle Länder (Indikator 5.4). In ähnlicher Weise übernehmen die am wenigsten entwickelten Länder sowie die kleinen Inselentwicklungsstaaten eine zunehmende globale Führungsrolle innerhalb der UN-Generaldebatte über die Zusammenhänge zwischen Gesundheit und Klimawandel (Indikator 5.4).

Angehörige der Gesundheitsberufe und ihre Verbände werden ebenfalls aktiv. Gesundheitseinrichtungen haben sich verpflichtet, Vermögenswerte im Wert von über 42 Milliarden US-Dollar von Investitionen in fossile Brennstoffe abzuziehen (Indikator 4.2.4). In der Wissenschaft hat die Veröffentlichung von Original-Studien zu Gesundheit und Klimawandel zwischen 2007 bis 2019 um das Neunfache zugenommen (Indikator 5.3).

Diese Entwicklungen übersetzen sich in den breiteren öffentlichen Diskurs. Von 2018 bis 2019 nahm die Berichterstattung über Gesundheit und Klimawandel in den Medien weltweit um 96 Prozent zu. Sie übertraf damit die Zunahme der Berichterstattung über den Klimawandel an sich und erreichte damit bislang den Höchststand (Indikator 5.1). Genau wie bei den Fortschritten in den Bereichen Sanitation und Hygiene sowie bei der Eindämmung des Tabakkonsums beginnt das zunehmende und anhaltende Engagement der Gesundheitsberufe über die letzten fünf Jahre nun eine entscheidende Lücke in der weltweiten Antwort auf den Klimawandel zu schließen.

Die nächsten fünf Jahre: Eine gemeinsame Antwort auf zwei Krisen im Bereich der öffentlichen Gesundheit

Am 12. Dezember 2020 jährt sich das Pariser Abkommen von 2015 zum fünften Mal. Die beteiligten Länder sind dazu angehalten, ihre nationalen Verpflichtungen zu aktualisieren und sie alle fünf Jahre zu überprüfen. Die kommenden fünf Jahre werden entscheidend sein. Um das Ziel von 1,5° C zu erreichen und den Temperaturanstieg „deutlich unter 2° C“ zu begrenzen, müssen die derzeit jährlich ausgestoßenen 56 Gigatonnen CO_2-Äquivalente (Gt CO2e) innerhalb von nur zehn Jahren (bis 2030) auf 25 Gt CO2e fallen. Praktisch erfordert dies eine jährliche Emissionsreduzierung um 7,6 Prozent, was einer Verfünffachung der derzeitigen Ambitionen der nationalen Regierungen entspricht. Erfolgen keine weiteren Maßnahmen in den nächsten fünf Jahren, erhöhen sich die erforderlichen Reduktionen auf 15,4 Prozent pro Jahr, wodurch das 1,5° C-Ziel außer Reichweite gerät.

Die Notwendigkeit forcierter Anstrengungen zur Bekämpfung des Klimawandels in den nächsten fünf Jahren muss im Zusammenhang mit den weltweiten Auswirkungen und der Antwort auf die COVID-19-Pandemie gesehen werden. Angesichts der hunderttausenden Todesopfer durch die Pandemie und den Klimawandel, der potenziellen wirtschaftlichen Kosten, die in die Billionen gehen , und der weiterreichenden Auswirkungen, die noch über Jahre zu spüren sein werden, müssen die ergriffenen Maßnahmen für beide Krisen der öffentlichen Gesundheit sorgfältig überprüft und eng miteinander verschränkt werden. Im Mai 2020 schrieben über 40 Millionen Angehörige der Gesundheitsberufe an internationale Regierungschefs, um das hervorzuheben. Diese Gesundheitsexperten sind hervorragend geeignet, um zwischen den beiden Themen zu vermitteln. Auch kann der klinische Ansatz zur Behandlung eines Patienten mit COVID-19 hilfreich sein, um verständlich zu machen, wie diese beiden Krisen im Bereich der öffentlichen Gesundheit gemeinsam angegangen werden sollten.

Erstens haben in einem akutmedizinischen Setting die schnelle Diagnose und umfassende Bewertung der Situation eine hohe Priorität. Das Problem muss auch darüber hinaus noch besser verstanden werden, einschließlich der Antworten auf die Fragen: Welche Bevölkerungsgruppen sind sowohl für die Pandemie als auch für den Klimawandel besonders anfällig? Wie haben die globalen und nationalen Volkswirtschaften reagiert und sich angepasst, und welche gesundheitlichen und ökologischen Folgen hat das? Welche Aspekte dieser Veränderungen sollten beibehalten werden, um eine längerfristig nachhaltige Entwicklung zu unterstützen?

Danach werden geeignete Reanimations- und Behandlungsoptionen geprüft und angewendet, wobei mögliche Nebenwirkungen, die Therapieziele und die lebenslange Gesundheit des Patienten sorgfältig in Betracht gezogen werden. Konjunkturpakete, bei denen veraltete Energie- und Transportformen mit hohem Verbrauch an fossilen Brennstoffen dominieren, werden unbeabsichtigte Nebenwirkungen haben und unnötigerweise zu den sieben Millionen Menschen beitragen, die jedes Jahr an Luftverschmutzung sterben. Hingegen werden Investitionen in die notwendigen Voraussetzungen für Gesundheit, wie erneuerbare Energien und saubere Luft, Infrastruktur für aktiven nicht-motorisierten Transport und körperliche Bewegung sowie eine resiliente und klimafreundliche Gesundheitsversorgung letztendlich effektiver sein.

Schließlich richtet sich die Aufmerksamkeit auf die Sekundärprävention und die langfristige Genesung, um die dauerhaften Auswirkungen der Krankheit zu minimieren und ihr Wiederauftreten zu verhindern. Viele der Schritte zur Vorbereitung auf unerwartete Krisen wie eine Pandemie ähneln denen, die erforderlich sind, um sich an die extremen Wetterbedingungen und neuen Bedrohungen anzupassen, die mit dem Klimawandel einhergehen. Dazu gehört die Notwendigkeit, vulnerable Bevölkerungsgruppen zu identifizieren, die Kapazität der öffentlichen Gesundheitssysteme zu bewerten, Vorsorgemaßnahmen zu entwickeln und in diese zu investieren sowie die Notwendigkeit einer widerstandsfähigen und gerechten

Martin School, University of Oxford, Oxford, UK (L Georgeson PhD, M Springmann PhD); **Department of Health Sciences, University of York, York, UK** (S Golder PhD, Prof H Graham PhD, P Lampard PhD); **CGIAR Research Program on Agriculture for Human Nutrition and Health, International Livestock Research Institute, Nairobi, Kenya** (D Grace PhD); **School of Public Health and Administration, Universidad Peruana Cayetano Heredia, Lima, Peru** (S Hartinger PhD); **Department of Epidemiology, Harvard TH Chan School of Public Health, Harvard University, Boston, MA, USA** (M P Jimenez PhD); **Department of Environmental Health, Boston University, Boston, MA, USA** (Prof P L Kinney ScD); **Health and Environment International Trust, Nelson, New Zealand** (Prof T Kjellstrom PhD); **School of Global Studies, University of Sussex, Falmer, UK** (Prof D Kniveton PhD); **School of Health** (B Lemke PhD) **and Department of Arts, Media and Digital Technologies** (M Otto MEng), **Nelson Marlborough Institute of Technology, Nelson, New Zealand; Gangarosa Department of Environmental Health** (L Shi ScD), **Rollins School of Public Health, Emory University, Atlanta, GA, USA** (Prof Y Liu PhD, B Vu MSPH); **Center on Global Energy Policy, Columbia University, New York, NY, USA** (M Lott PhD); **Department of Genetics and Microbiology, Universitat Autònoma de Barcelona, Barcelona, Spain** (Prof J Martinez-Urtaza PhD); **Center for Energy Markets, Technical University of Munich, Munich, Germany** (L McAllister PhD); **Data Science Lab, Hertie School, Berlin, Germany** (Prof S Jankin Mikhaylov PhD, Prof S Munzert PhD); **School of Geography, University of Melbourne, Melbourne, VIC, Australia** (C McMichael PhD); **Preventive Medicine and Public Health Research Center, Psychosocial Health Research Institute, Iran University of Medical Sciences, Tehran, Iran** (Prof M Moradi-Lakeh MD);

European Centre for Environment and Human Health (K Morrissey PhD) and Medical and Health School (Prof D Pencheon MSc), University of Exeter, Exeter, UK; Medical Research Council Centre for Global Infectious Disease Analysis, Department of Infectious Disease Epidemiology, Imperial College London, London, UK (K A Murray PhD); Medical Research Council Unit The Gambia at London School of Hygiene & Tropical Medicine, Bakau, The Gambia (K A Murray); Iranian Fisheries Science Research Institute, Agricultural Research, Education, and Extension Organisation, Tehran, Iran (F Owfi PhD, M Rabbaniha PhD); Department of Civil and Structural Engineering, University of Sheffield, Sheffield, UK (R Quinn PhD); Scientific Assessment Section, European Centre for Disease Prevention and Control, Solna, Sweden (Prof J C Semenza PhD); WHO-WMO Joint Climate and Health Office, Geneva, Switzerland (J Shumake-Guillemot DrPH); Institute of Tropical Aquaculture and Fisheries, Universiti Malaysia Terengganu, Kuala Terengganu, Malaysia (Prof M Tabatabaei PhD); Department of Civil Engineering, Tampere University, Tampere, Finland (J Taylor PhD); and Department of Electronics and Computer Science, CRETUS Institute, Universidade de Santiago de Compostela, Santiago, Spain (J Triñanes PhD)

Correspondence to: Dr Nick Watts, Institute for Global Health, University College London, London W1T 4TJ, UK nicholas.watts@ucl.ac.uk

For **Peter Byass' obituary** see **Obituary** *Lancet* 2020; **396:** 752

Gemeinschaft zu betonen. Werden die gegenwärtigen und zukünftigen Auswirkungen des Klimawandels nicht berücksichtigt, wird das die Bemühungen zur Vorbereitung auf künftige Pandemien vermutlich untergraben.

Bei jedem Schritt ist es in beiden Fällen von größter Bedeutung, die Dringlichkeit des Handelns dem Ausmaß der Bedrohung anzupassen, sich an die beste verfügbare Wissenschaft zu halten und klar und konsistent zu kommunizieren. Die Folgen der Pandemie werden die Wirtschafts-, Sozial- und Umweltpolitik der Regierungen in den nächsten fünf Jahren prägen. Dieser Zeitraum ist ausschlaggebend für die Entscheidung, ob die Temperaturen „weit unter 2° C" bleiben werden. Wenn die globale Erholung von COVID-19 nicht mit der Antwort auf den Klimawandel in Einklang steht, wird die Welt das im Pariser Abkommen festgelegte Ziel nicht erreichen und die öffentliche Gesundheit kurz- und langfristig schädigen.

www.thelancet.com **Published online December 2, 2020 https://doi.org/10.1016/S0140-6736(20)32290-X**
