# Supplementary material for: The 2020 report of the Lancet Countdown on health and climate change: responding to converging crises
Source: Lancet. Author manuscript; Available in PMC 2024 Nov 15. (PMC7616803; doi:10.1016/S0140-6736(20)32290-X)
Supplement: Spanish translation of the abstract [file EMS200002-supplement-Spanish_translation_of_the_abstract.pdf]

THE LANCET

Supplementary appendix

This translation in Spanish was submitted by the authors and we reproduce it as supplied. It has not been peer reviewed. *The Lancet*'s editorial processes have only been applied to the original in English, which should serve as reference for this manuscript.

Los autores nos proporcionaron esta traducción al español y la reproducimos tal como nos fue entregada. No la hemos revisado. Los procesos editoriales de *The Lancet* se han aplicado únicamente al original en inglés, que debe servir de referencia para este manuscrito.

Supplement to: Watts N, Amann M, Arnell N, et al. The 2020 report of The *Lancet* Countdown on health and climate change: responding to converging crises. *Lancet* 2020; published online Dec 2. http://dx.doi.org/10.1016/S0140-6736(20)32290-X.

El Informe 2020 del Lancet Countdown sobre la salud y el cambio climático: respondiendo a dos crisis convergentes

Nick Watts, Markus Amann, Nigel Arnell, Sonja Ayeb-Karlsson, Jessica Beagley, Kristine Belesova, Maxwell Boykoff, Peter Byass, Wenjia Cai, Diarmid Campbell-Lendrum, Stuart Capstick, Jonathan Chambers, Samantha Coleman, Carole Dalin, Meaghan Daly, Niheer Dasandi, Shouro Dasgupta, Michael Davies, Claudia Di Napoli, Paula Dominguez-Salas, Paul Drummond, Robert Dubrow, Kristie L Ebi, Matthew Eckelman, Paul Ekins, Luis E Escobar, Lucien Georgeson, Su Golder, Delia Grace, Hilary Graham, Paul Haggar, Ian Hamilton, Stella Hartinger, Jeremy Hess, Shih-Che Hsu, Nick Hughes, Slava Jankin Mikhaylov, Marcia P Jimenez, Ilan Kelman, Harry Kennard, Gregor Kiesewetter, Patrick L Kinney, Tord Kjellstrom, Dominic Kniveton, Pete Lampard, Bruno Lemke, Yang Liu, Zhao Liu, Melissa Lott, Rachel Lowe, Jaime Martinez-Urtaza, Mark Maslin, Lucy McAllister, Alice McGushin, Celia McMichael, James Milner, Maziar Moradi-Lakeh, Karyn Morrissey, Simon Munzert, Kris A Murray, Tara Neville, Maria Nilsson, Maquins Odhiambo Sewe, Tadj Oreszczyn, Matthias Otto, Fereidoon Owfi, Olivia Pearman, David Pencheon, Ruth Quinn, Mahnaz Rabbaniha, Elizabeth Robinson, Joacim Rocklöv, Marina Romanello, Jan C Semenza, Jodi Sherman, Liuhua Shi, Marco Springmann, Meisam Tabatabaei, Jonathon Taylor, Joaquin Triñanes, Joy Shumake-Guillemot, Bryan Vu, Paul Wilkinson, Matthew Winning, Peng Gong, Hugh Montgomery*, Anthony Costello**

Resumen ejecutivo

Lancet Countdown es una colaboración internacional, establecida para proporcionar un sistema de vigilancia independiente y global dedicado a seguir el perfil de salud emergente del cambio climático.

El informe del 2020 presenta 43 indicadores en cinco áreas: impactos del cambio climático, exposición y vulnerabilidad; adaptación, planificación y resiliencia en la salud; acciones de mitigación y co-beneficios para la salud; economía y finanzas; y compromiso público y político. Este informe representa las conclusiones y el consenso de las 35 principales instituciones académicas y organismos de las Naciones Unidas que componen Lancet Countdown, y recurre a la experiencia de climatólogos, geógrafos e ingenieros; de especialistas en energía, alimentación y transporte; y de economistas, científicos sociales y políticos, científicos de datos, profesionales de la salud pública y médicos.

El perfil de salud que emerge del cambio climático

Hace cinco años, los países se comprometieron a limitar el calentamiento a "bien por debajo de 2 °C", como parte del histórico Acuerdo de París. Cinco años después, las emisiones globales de CO_2 continúan aumentando constantemente, sin una disminución convincente ni sostenida, habiendo resultado en un aumento de 1,2 °C de la temperatura media global. De hecho, los cinco años más calurosos de los que se tiene constancia se han producido desde el año 2015.

Los cambios en el clima ya han producido alteraciones importantes en los determinantes sociales y ambientales subyacentes de la salud a nivel mundial. Los indicadores en todos los ámbitos de *impactos, exposiciones y vulnerabilidades* que la colaboración sigue están empeorando. Se observan tendencias preocupantes, y a menudo aceleradas, para cada uno de los síntomas humanos del cambio climático que se vigilan, siendo los indicadores de 2020 los que presentan las perspectivas más preocupantes que se hayan informado desde que se estableció por primera vez Lancet Countdown.

Esos efectos suelen ser desiguales y repercuten de manera desproporcionada en las poblaciones que menos han contribuido al problema. Esto revela una cuestión de justicia más profunda, en la que el cambio climático interactúa con las desigualdades sociales y económicas existentes y exacerba tendencias arraigadas dentro de cada país y entre países. Un examen de las causas del cambio climático revela cuestiones similares, y muchas prácticas y políticas con altas emisiones de carbono dan lugar a la mala calidad del aire, la mala calidad de los alimentos y la mala calidad de la vivienda, lo que perjudica desproporcionadamente la salud de las poblaciones más desfavorecidas.

Las poblaciones vulnerables estuvieron expuestas a 475 millones de sucesos de olas de calor adicionales en todo el mundo, lo que a su vez se reflejó en un exceso de morbilidad y mortalidad. En los últimos 20 años, se ha producido un aumento del 53,7 % de la mortalidad relacionada con el calor en personas mayores de 65 años, alcanzando un total de 296.000 muertes en 2018 (indicadores 1.1.2 y 1.1.3). El coste elevado en términos de vidas humanas y sufrimiento se asocia también a impactos en el rendimiento económico, con más de 302.000 millones de horas de capacidad laboral potencial perdidas en 2019 (indicador 1.1.4). La India e Indonesia se encuentran entre los países más afectados, y sufren pérdidas potenciales de capacidad laboral equivalentes a un 4 y un 6 % de su producto bruto interno anual (indicador 4.1.3). En Europa, en 2018, el coste monetizado de la mortalidad relacionada con el calor equivalía al 1,2 % del ingreso nacional bruto regional, es decir, el ingreso promedio de 11 millones de ciudadanos europeos (indicador 4.1.2).

En lo que respecta a los fenómenos meteorológicos extremos, los avances de la ciencia climática permiten cada vez más una mayor precisión y certeza en su atribución; estudios realizados sobre eventos ocurridos entre 2015 y 2020 han mostrado las huellas del cambio climático en 76 inundaciones, sequías, tormentas y anomalías en la temperatura (indicador 1.2.3). Además, entre 2001-04 y 2016-19 se registró un aumento del

Published Online
December 2, 2020
https://doi.org/10.1016/S0140-6736(20)32290-X

*Co-chairs

Institute for Global Health (N Watts MA, J Beagley BA, S Coleman MSE, Prof I Kelman PhD, A McGushin MSc, M Romanello PhD), **Office of the Vice Provost for Research** (Prof A Costello FmedSci), **Energy Institute** (S-C Hsu MSc, I Hamilton PhD, H Kennard PhD, Prof T Oreszczyn PhD), **Institute for Sustainable Resources** (C Dalin PhD, P Drummond MSc, Prof P Ekins PhD, N Hughes PhD, M Winning PhD), **Institute for Environmental Design and Engineering** (Prof M Davies PhD), **Department of Geography** (Prof M Maslin PhD), **and Institute for Human Health and Performance** (Prof H Montgomery MD), **University College London, London, UK; Air Quality and Greenhouse Gases Program, International Institute for Applied Systems Analysis, Laxenburg, Austria** (M Amann PhD, G Kiesewetter PhD); **Department of Meteorology** (Prof N W Arnell PhD) **and School of Agriculture, Policy, and Development** (C Di Napoli PhD, Prof E Robinson PhD), **University of Reading, Reading, UK; Institute for Environment and Human Security, United Nations University, Bonn, Germany** (S Ayeb-Karlsson PhD); **Centre on Climate Change and Planetary Health** (K Belesova PhD), **Department**

of Population Health (P Dominguez-Salas PhD), Centre for Mathematical Modelling of Infectious Diseases (R Lowe PhD), and Department of Public Health, Environments, and Society (J Milner PhD, Prof P Wilkinson FRCP), London School of Hygiene & Tropical Medicine, London, UK; Environmental Studies Program, University of Colorado Boulder, Boulder, CO, USA (Prof M Boykoff PhD, O Pearman MEM); Department of Epidemiology and Global Health (Prof P Byass PhD, Prof M Nilsson PhD) and Department of Public Health and Clinical Medicine (M O Sewe PhD, Prof J Rocklöv PhD), Umeå University, Umeå, Sweden; Department of Earth System Science, Tsinghua University, Beijing, China (W Cai PhD, Prof P Gong PhD, Z Liu PhD); Environment, Climate Change and Health Department, World Health Organization, Geneva, Switzerland (D Campbell-Lendrum DPhil, T Neville MSc); School of Psychology, Cardiff University, Cardiff, UK (S Capstick PhD, P Haggar PhD); Institute for Environmental Sciences, University of Geneva, Geneva, Switzerland (J Chambers PhD); Department of Environmental Studies, University of New England, Biddeford, ME, USA (M Daly PhD); School of Government, University of Birmingham, Birmingham, UK (N Dasandi PhD); Centro Euro-Mediterraneo sui Cambiamenti Climatici, Venice, Italy (S Dasgupta PhD); Yale Center on Climate Change and Health (Prof R Dubrow PhD) and Department of Anesthesiology (J Sherman MD), Yale University, New Haven, CT, USA; Department of Global Health (Prof K L Ebi PhD) and Center for Health and the Global Environment (J Hess MD), University of Washington, Seattle, WA, USA; Department of Civil & Environmental Engineering, Northeastern University, Boston, MA, USA (M Eckelman PhD); Department of Fish and Wildlife Conservation, Virginia Polytechnic Institute and State University, Blacksburg, VA, USA (L E Escobar PhD); Oxford

número de días en que las personas estuvieron expuestas a un riesgo muy alto o extremadamente alto de incendio forestal en 114 países (indicador 1.2.1). Conforme con ello, el 67 % de las ciudades mundiales encuestadas esperan que el cambio climático comprometa gravemente sus bienes e infraestructuras de salud pública (indicador 2.1.3).

El clima cambiante tiene efectos posteriores, que afectan a sistemas ambientales más amplios, perjudicando a su vez la salud humana. La seguridad alimentaria mundial se ve amenazada por el aumento de las temperaturas y el incremento de la frecuencia de los fenómenos extremos; el potencial de rendimiento mundial de los principales cultivos disminuyó entre un 1,8 y un 5,6 % entre 1981 y 2019 (indicador 1.4.1). La idoneidad climática para la transmisión de enfermedades infecciosas ha venido aumentando rápidamente desde la década de 1950, con un aumento del 15 % en la idoneidad climática para el dengue provocado por el *Aedes albopictus* en 2018 y aumentos regionales en la idoneidad climática para malaria y bacterias del género *Vibrio* (indicador 1.3.1). La proyección hacia el futuro, sobre la base de las poblaciones actuales, indica que entre 145 y 565 millones de personas se enfrentan a posibles inundaciones debido a la elevación del nivel del mar (indicador 1.5).

A pesar de estas señales claras y en aumento, la respuesta mundial al cambio climático ha sido endeble, y los esfuerzos nacionales siguen estando muy por debajo de los compromisos asumidos en el Acuerdo de París. La intensidad de carbono del sistema energético mundial se ha mantenido casi sin cambios durante 30 años, y el uso de carbón a nivel mundial ha aumentado en un 74 % durante este tiempo (indicadores 3.1.1 y 3.1.2). La reducción del uso mundial de carbón que se había observado desde 2013 se ha invertido durante los dos últimos años: el uso de carbón aumentó un 1,7 % de 2016 a 2018. La carga para la salud es considerable, cada año se producen más de un millón de muertes como consecuencia de la contaminación atmosférica provocada por la energía del carbón, y unas 390.000 de esas muertes fueron el resultado de la contaminación por partículas en 2018 (indicador 3.3). La respuesta del sector alimentario y agrícola ha sido igualmente preocupante. Las emisiones procedentes del ganado aumentaron en un 16 % entre 2000 y 2017, con 93 % de las emisiones proviniendo de los rumiantes (indicador 3.5.1). Asimismo, se están volviendo más comunes en todo el mundo las dietas crecientemente insalubres, con el consumo excesivo carnes rojas contribuyendo a unas 990.000 muertes en 2017 (indicador 3.5.2). Cinco años después de que los países llegaran a un acuerdo en París, un numero preocupante de indicadores muestran una reversión incipiente, pero sostenida, de las tendencias positivas previamente identificadas en informes anteriores (indicadores 1.3.2, 3.1.2 y 4.2.3).

Una respuesta en aumento de los profesionales de la salud

A pesar de las escasas mejoras de la economía en su conjunto, se han logrado avances en varios sectores clave: de 2010 a 2017, la tasa de crecimiento anual en la capacidad de energía renovable fue del 21 %, y la electricidad con bajas emisiones de carbono fue responsable del 28 % de la capacidad eléctrica de China en 2017 (indicador 3.1.3). Sin embargo, los indicadores presentados en el informe de 2020 de Lancet Countdown sugieren que algunos de los progresos más considerables se observaron en el creciente compromiso de la profesión sanitaria con el cambio climático, a nivel mundial. Los médicos, el personal de enfermería y la profesión *médica* en general, desempeñan un papel fundamental en la adaptación de los sistemas de salud y la mitigación de su impacto ambiental, en la comprensión y la maximización de los beneficios para la salud de las intervenciones implementadas, y en la comunicación de la necesidad de una respuesta acelerada al cambio climático.

En el caso de la adaptación en los sistemas nacionales de salud, este cambio está en marcha. Los servicios de salud de 86 países están ahora conectados con sus servicios meteorológicos nacionales para ayudar en la planificación de la adaptación sanitaria (indicador 2.2). Por lo menos 51 países han desarrollado planes nacionales de adaptación sanitaria, y el gasto mundial en adaptación sanitaria aumentó al 5,3 % de todo el gasto de adaptación en 2018-19, llegando a los 18.400 millones de dólares en 2019 (indicadores 2.1.1 y 2.4).

El sector de la salud, responsable del 4,6 % de las emisiones mundiales de gases de efecto invernadero en 2017, está tomando medidas tempranas pero importantes para reducir sus propias emisiones (indicador 3.6). En el Reino Unido, el Servicio Nacional de Salud ha declarado su ambición de ofrecer un “servicio de salud con huella de carbono cero” lo antes posible, basándose en una década de avances impresionantes en la reducción de las emisiones de carbono de la atención sanitaria, correspondientes a un 57 % desde 1990, y un 22 % si se considera la cadena de suministro y las responsabilidades más amplias. Por otra parte, el Departamento de Salud de Australia Occidental utilizó su *Ley de Salud Pública de* 2016 para llevar a cabo la primera investigación sobre el clima y la salud de Australia, y el Ministerio Federal de Salud de Alemania ha establecido un departamento dedicado a la protección de la salud y la sostenibilidad responsable de las cuestiones relacionadas con el clima. Este progreso se está distribuyendo de manera más uniforme en todo el mundo, ya que el 73 % de los países hacen referencia explícita a la salud y el bienestar en sus Contribuciones Determinadas a Nivel Nacional en el marco del Acuerdo de París, y el 100 % de los países del Asia sudoriental y el Mediterráneo oriental lo hacen (indicador 5.4). Del mismo modo, los países menos desarrollados y los

 www.thelancet.com Published online December 2, 2020 https://doi.org/10.1016/S0140-6736(20)32290-X

pequeños estados insulares en desarrollo asumen un liderazgo mundial cada vez mayor en el marco del debate general de las Naciones Unidas con respecto a las conexiones entre la salud y el cambio climático (indicador 5.4).

Los profesionales de la salud y sus asociaciones están también respondiendo, y las instituciones de salud se han comprometido a desinvertir sus activos de los combustibles fósiles, por valor de más de 42.000 millones de dólares estadounidenses (indicador 4.2.4). En el ámbito académico, la publicación de investigaciones originales sobre la salud y el cambio climático se ha multiplicado por nueve entre 2007 y 2019 (indicador 5.3).

Estos cambios se traducen en un discurso público más amplio. Entre 2018 y 2019, la cobertura de la salud y el cambio climático en los medios de comunicación del mundo aumentó en un 96 %, superando el aumento de la cobertura del cambio climático en general, y alcanzando el punto más alto observado hasta la fecha (indicador 5.1). Igual que ocurrió con los avances en materia de saneamiento e higiene y con el control del tabaco, el compromiso creciente y sostenido de la profesión sanitaria durante los últimos cinco años ahora empieza a llenar un vacío crucial en la respuesta mundial ante el cambio climático.

Los próximos cinco años: Una respuesta conjunta a dos crisis de salud pública

El 12 de diciembre de 2020 se celebrará el aniversario del Acuerdo de París de 2015, y se prevé que los países actualicen sus Contribuciones Determinadas a Nivel Nacional, y las revisen cada cinco años. Estos próximos cinco años serán cruciales. Para alcanzar el objetivo de 1,5°C y mantener el aumento de la temperatura "bien por debajo de 2 °C", las 56 gigatoneladas de CO_2e (Gt de CO_2e) que se emiten actualmente de forma anual tendrán que disminuir a 25 Gt de CO_2e en solamente 10 años (para 2030). En efecto, esto requiere una reducción del 7,6 % cada año, lo que representa un aumento de cinco veces en los niveles actuales de ambición de los gobiernos nacionales. Sin más intervención en los próximos cinco años, las reducciones necesarias aumentarán al 15,4 % cada año, lo que hace que el objetivo de 1,5 °C esté fuera de alcance.

La necesidad de esfuerzos acelerados para hacer frente al cambio climático durante los próximos cinco años se contextualizará en los efectos de la pandemia COVID-19 y la respuesta mundial a la misma. Con la pérdida de vidas a causa de la pandemia y del cambio climático en el orden de cientos de miles, los posibles costes económicos en el orden de billones, y la expectativa de que las consecuencias se extiendan muchos años por venir, las medidas que se adopten para hacer frente a ambas crisis de salud pública deben examinarse cuidadosamente y vincularse estrechamente. En mayo de 2020, más de 40 millones de profesionales de la salud escribieron a los líderes mundiales, resaltando este punto. Estos profesionales de la salud están bien posicionados para actuar como puente entre las dos cuestiones, y considerar el enfoque clínico del tratamiento de un paciente con COVID-19 puede ser útil para comprender las formas en que deben abordarse conjuntamente estas dos crisis de salud pública.

En primer lugar, en un contexto agudo, se da una gran prioridad al diagnóstico rápido y a la evaluación exhaustiva de la situación. Asimismo, es necesario trabajar para comprender el problema, incluyendo: qué poblaciones son vulnerables tanto a la pandemia como al cambio climático; cómo han reaccionado y se han adaptado las economías mundiales y nacionales, y cuáles han sido las consecuencias sanitarias y ambientales; y qué aspectos de esos cambios deben conservarse para apoyar el desarrollo sostenible a largo plazo.

En segundo lugar, se examinan y administran opciones apropiadas de resucitación y tratamiento, con una consideración cuidadosa de los posibles efectos secundarios, los objetivos de la atención, y la salud del paciente a lo largo de toda su vida. Los paquetes de recuperación económica que den prioridad a formas anticuadas de energía y transporte con uso intensivo de combustibles fósiles tendrán efectos secundarios no deseados, que se sumarán innecesariamente a los siete millones de personas que mueren cada año a causa de la contaminación atmosférica. En su lugar, las inversiones en imperativos de salud, tales como la energía renovable y el aire limpio, la infraestructura de viajes activos y la actividad física, y la atención de salud adaptable e inteligente con respecto al clima, serán en última instancia más eficaces.

Por último, la atención se centra en la prevención secundaria y la recuperación a largo plazo, tratando de reducir al mínimo los efectos permanentes de la enfermedad y evitar su reaparición. Muchas de las medidas adoptadas para prepararse para crisis inesperadas, como una pandemia, son similares a las que se requieren para adaptarse a los extremos del clima y a las nuevas amenazas que se esperan del cambio climático. Estas medidas incluyen la necesidad de identificar a las poblaciones vulnerables, evaluar la capacidad de los sistemas de salud pública, desarrollar medidas de preparación e invertir en ellas, y hacer hincapié en la capacidad de recuperación y la equidad de las comunidades. De hecho, si no se tienen en cuenta los efectos actuales y futuros del cambio climático, es probable que se debiliten los esfuerzos de preparación para futuras pandemias.

En cada etapa y en ambos casos, es primordial actuar con un nivel de urgencia proporcional a la magnitud de la amenaza, adherirse a la mejor ciencia disponible, y comunicar de forma clara y coherente. Las consecuencias de la pandemia contextualizarán las políticas económicas, sociales y ambientales de los gobiernos durante los próximos cinco años, un periodo que es crucial para

Martin School, University of Oxford, Oxford, UK (L Georgeson PhD, M Springmann PhD); **Department of Health Sciences, University of York, York, UK** (S Golder PhD, Prof H Graham PhD, P Lampard PhD); **CGIAR Research Program on Agriculture for Human Nutrition and Health, International Livestock Research Institute, Nairobi, Kenya** (D Grace PhD); **School of Public Health and Administration, Universidad Peruana Cayetano Heredia, Lima, Peru** (S Hartinger PhD); **Department of Epidemiology, Harvard TH Chan School of Public Health, Harvard University, Boston, MA, USA** (M P Jimenez PhD); **Department of Environmental Health, Boston University, Boston, MA, USA** (Prof P L Kinney ScD); **Health and Environment International Trust, Nelson, New Zealand** (Prof T Kjellstrom PhD); **School of Global Studies, University of Sussex, Falmer, UK** (Prof D Kniveton PhD); **School of Health** (B Lemke PhD) **and Department of Arts, Media and Digital Technologies** (M Otto MEng), **Nelson Marlborough Institute of Technology, Nelson, New Zealand; Gangarosa Department of Environmental Health** (L Shi ScD), **Rollins School of Public Health, Emory University, Atlanta, GA, USA** (Prof Y Liu PhD, B Vu MSPH); **Center on Global Energy Policy, Columbia University, New York, NY, USA** (M Lott PhD); **Department of Genetics and Microbiology, Universitat Autònoma de Barcelona, Barcelona, Spain** (Prof J Martinez-Urtaza PhD); **Center for Energy Markets, Technical University of Munich, Munich, Germany** (L McAllister PhD); **Data Science Lab, Hertie School, Berlin, Germany** (Prof S Jankin Mikhaylov PhD, Prof S Munzert PhD); **School of Geography, University of Melbourne, Melbourne, VIC, Australia** (C McMichael PhD); **Preventive Medicine and Public Health Research Center, Psychosocial Health Research Institute, Iran University of Medical Sciences, Tehran, Iran** (Prof M Moradi-Lakeh MD);

determinar si las temperaturas se mantendrán "bien por debajo de los 2 °C". A menos que la recuperación mundial de COVID-19 se ajuste a la respuesta al cambio climático, el mundo no podrá cumplir el objetivo establecido en el Acuerdo de París, lo cual perjudicará la salud pública tanto a corto como a largo plazo.

European Centre for Environment and Human Health (K Morrissey PhD) **and Medical and Health School** (Prof D Pencheon MSc), **University of Exeter, Exeter, UK; Medical Research Council Centre for Global Infectious Disease Analysis, Department of Infectious Disease Epidemiology, Imperial College London, London, UK** (K A Murray PhD); **Medical Research Council Unit The Gambia at London School of Hygiene & Tropical Medicine, Bakau, The Gambia** (K A Murray); **Iranian Fisheries Science Research Institute, Agricultural Research, Education, and Extension Organisation, Tehran, Iran** (F Owfi PhD, M Rabbaniha PhD); **Department of Civil and Structural Engineering, University of Sheffield, Sheffield, UK** (R Quinn PhD); **Scientific Assessment Section, European Centre for Disease Prevention and Control, Solna, Sweden** (Prof J C Semenza PhD); **WHO-WMO Joint Climate and Health Office, Geneva, Switzerland** (J Shumake-Guillemot DrPH); **Institute of Tropical Aquaculture and Fisheries, Universiti Malaysia Terengganu, Kuala Terengganu, Malaysia** (Prof M Tabatabaei PhD); **Department of Civil Engineering, Tampere University, Tampere, Finland** (J Taylor PhD); **and Department of Electronics and Computer Science, CRETUS Institute, Universidade de Santiago de Compostela, Santiago, Spain** (J Triñanes PhD)

Correspondence to: Dr Nick Watts, Institute for Global Health, University College London, London W1T 4TJ, UK **nicholas.watts@ucl.ac.uk**

For **Peter Byass' obituary** see **Obituary** *Lancet* 2020; **396:** 752

 www.thelancet.com **Published online December 2, 2020 https://doi.org/10.1016/S0140-6736(20)32290-X**
