# Supplementary material for: The 2020 report of the Lancet Countdown on health and climate change: responding to converging crises
Source: Lancet. Author manuscript; Available in PMC 2024 Nov 15. (PMC7616803; doi:10.1016/S0140-6736(20)32290-X)
Supplement: Supplementary appendix [file EMS200002-supplement-Supplementary_appendix.pdf]

THE LANCET

Supplementary appendix

This appendix formed part of the original submission and has been peer reviewed. We post it as supplied by the authors.

Supplement to: Watts N, Amann M, Arnell N, et al. The 2020 report of The *Lancet* Countdown on health and climate change: responding to converging crises. *Lancet* 2020; published online Dec 2. http://dx.doi.org/10.1016/S0140-6736(20)32290-X.

The 2020 Report of the Lancet Countdown on Health and Climate Change

Appendix

Section 1: Climate Change Impacts, Exposures, and Vulnerability

1.1: Heat and Health

Health and Exposure to Warming

Methods

The methodology for this indicator remains similar to that described in the 2019 report of the Lancet Countdown appendix.[1] It however uses as input data the monthly temperature historical records from ECMWF ERA5 climate reanalysis dataset,[2] which supersedes the ERA-Interim used in previous years.[3] Change in summer temperature was calculated on a global grid at 0.5° by 0.5°. A baseline temperature grid was calculated as the average of summer temperatures (June, July, August for the northern hemisphere, December, January, February for the southern hemisphere) from 1986-2005 using a global grid of temperatures from the ERA5. Using this same dataset, temperature changes relative to the 1986-2005 average were calculated for every grid point for every year. The 'population-weighted' average was calculated by weighting each grid cell by the fraction of the total world population contained within that grid cell. The population data has been extended to the pre-2000 period by merging the NASA GPWv4 dataset (valid from 2000 to present) with the ISIMIP Histsoc dataset (valid for 1980-2000).[4,5]

This makes it possible to calculate exposure trends from 1980 to the present day, giving more context to these trends. Note however that as the population data is discontinuous there can be some inconsistencies between the pre and post 2000 values. Manual inspection of the datasets indicates that for total population the discrepancies at the global scale are small in percentage terms but still are up to 60 million in absolute terms. It is not clear if there is any spatial bias in these discrepancies, therefore per-countries analysis should be avoided when covering time spans which cross the year 2000.

Data

1. Climate data from the European Centre for Medium-Range Weather Forecasts (ECMWF) ERA5 reanalysis.[2]
2. Population data from the NASA Socioeconomic Data and Applications Center (SEDAC) Gridded Population of the World (GPWv4) and The Inter-Sectoral Impact Model Intercomparison Project (ISIMIP) Histsoc dataset.[4,5]

Caveats

As two distinct sources were used for population data some inconsistencies between pre and post 2000 values may be present. Manual inspection of the datasets indicates that discrepancies at the global scale are small in percentage terms but still are up to 60 million in absolute terms. As it is not clear if there is any spatial bias in these discrepancies, per-country analysis should be avoided when covering time spans which cross the year 2000.

Future form of the indicator

Future development of this indicator will require new population data sources, as the NASA GPWv4 project covers only until 2020. Additionally, the anticipated extension of the ECMWF ERA5 dataset back to 1950 will allow a longer time series to be analysed.

Additional analysis

The change in summer temperature relative to the 1986–2005 average is presented (Figure 1). In 2019 the upward trend in both the global mean summer temperature anomaly relative (+0.36˚C) and population weighted summer temperature anomaly (+0.83˚C) continued.

Figure 2 maps the mean summer temperature anomaly for 2019 and highlights the large increases across Australia, for which 2019 was its warmest year on record. This is also consistent with the range of other heat-related climate hazard, namely droughts and wildfires, that have occurred in the country.[6]

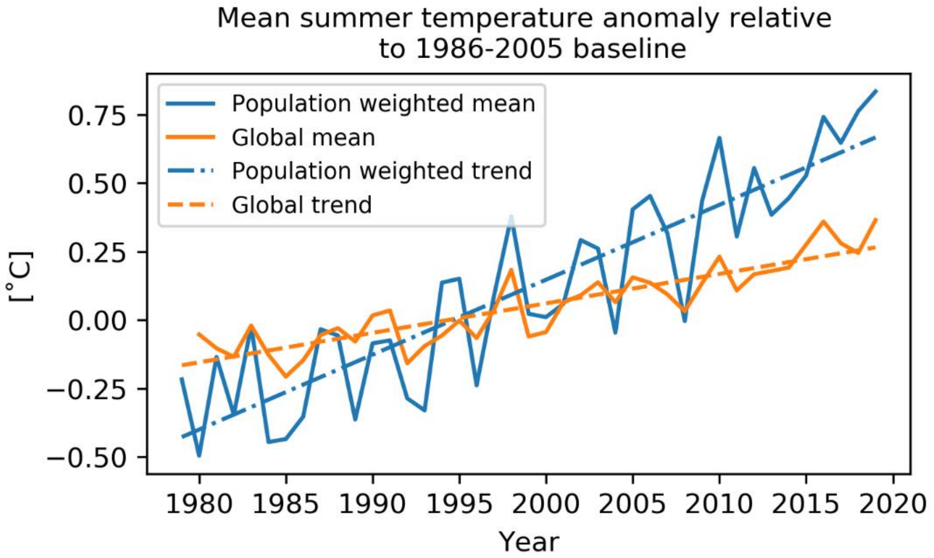

Figure 1: Global mean trends of summer temperature anomaly compared to the population weighted trend (relative to the 1986-2005 baseline).

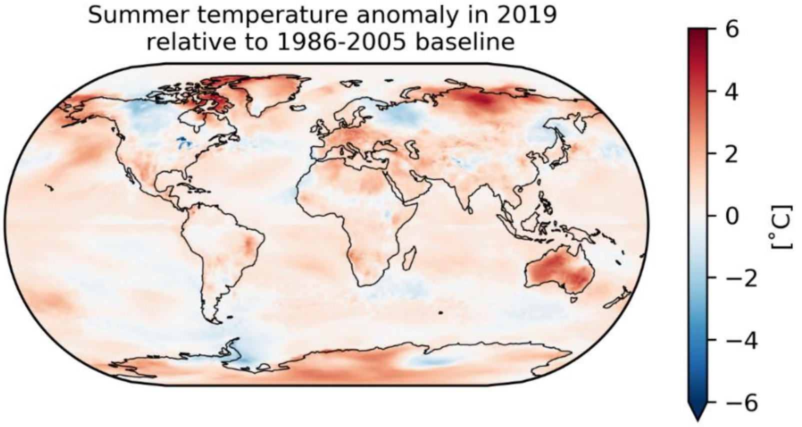

Figure 2: Map of summer temperature anomaly for 2019 relative to the 1986-2005 baseline.

Indicator 1.1.1: Vulnerability to Extremes of Heat

Methods

The methodology for this indicator has been updated from previous reports.[7] This year it presents the indicator presented in previous reports, as well as a first attempt at estimating risk of health impacts from heat.

The first part of the indicator displays an index derived by taking mean of proportion of the population over 65 years;[8] the prevalence of cardiovascular, diabetes and chronic respiratory diseases among population over 65 years using GBD study 2017 estimates; and the proportion of the population living in urban areas as a measure of exposure to urban heat island. The index ranges between 0 and 100 and is a measure of potential vulnerability to heat exposure of the population over 65 years by country. Aggregated trends are displayed by WHO regional classifications for the period 1990 to 2017.

The second part of the indicator combines exposure data as described in indicator 1.1.1, along with further data on population vulnerability and concepts of adaptation and resilience measures as detailed in Section 2 of the report.

The calculation for this index is as follows:

$$\frac{He}{Pop} \times \left(\frac{Dp + Ur}{2}\right) \times \frac{1}{IHR}$$

Where *He* is the five year moving average of the annual increase in the number of heatwave exposure days for the over 65 population compared with the 1986-2005 baseline. *Pop* is the total population. *Dp* is the chronic disease prevalence in the over 65 population: the arithmetic mean of the prevalence of chronic respiratory disease, cardiovascular disease and diabetes. *Ur* is the proportion of population living in urban areas. *IHR* is the average International Health Regulations core capacity score.

Variables in each of categories were limited to datasets that were readily available at the time of publishing and have sufficient global coverage (i.e. a majority reporting of WHO countries). Following collection of the variable data, any missing data points were interpolated or considered 'missing'.

Data

1. Data on heatwave exposure in the over 65 population is taken from Indicator 1.1.1.
2. Population data is from the NASA Socioeconomic Data and Applications Center (SEDAC) Gridded Population of the World (GPWv4) and The Inter-Sectoral Impact Model Intercomparison Project (ISIMIP) Histsoc dataset as described in Indicator 1.1.1.[4,5]
3. Global Burden of Disease Collaborative Network. Global Burden of Disease Study 2017 (GBD 2017) Population Estimates 1950-2017. Seattle, United States: Institute for Health Metrics and Evaluation (IHME), 2018.[8]
4. Global Burden of Disease Collaborative Network. Global Burden of Disease Study 2017 (GBD 2017) Results. Seattle, United States: Institute for Health Metrics and Evaluation (IHME), 2018. Available from http://ghdx.healthdata.org/gbd-results-tool.[9]
5. Urban population (% of total) The United Nations Population Division's World Urbanization Prospects.[10]
6. IHR core capacities data, 2010-2017.[11]

Caveats

There is no consistent and universally accepted standard for distinguishing urban from rural areas, in part because of the wide variety of situations across countries. Most countries use an urban classification related to the size or characteristics of settlements.[12] This indicator does not include the existence of heat early warning systems, or prevalence of cooling devices. Neither does it include the prevalence of green areas in cities.

Future Form of Indicator

Future iterations of this indicator will consider the incorporation of adaptation measures more specific to heat and heatwaves, such as the use of heat early warning systems and heat adaptation plans and urban greenness. For future Lancet Countdown reports, a full suite of indices will be built for the following climate stressors: floods, wildfires, mosquito-borne diseases, drought, and sea-level rise. As with many of the Lancet Countdown's indicators, the indices would be a part of the process of annual improvement. The improvement process will be started by assembling a panel of climate and health experts in order to discuss the best set of variables to be used in each of the different indices, their comparative baselines, and the proper modes of data validation. Additionally, methodology around developing a single global index will be explored.

Indicator 1.1.2: Exposure of Vulnerable Populations to Heatwaves

Methods

The data source and the methodology for this indicator have been extended and improved for the 2020 report. The data source has been changed from ERA-Interim to ERA5.[2] ERA5 provides temperature reanalysis data at high time resolution (hourly steps) which allows daily minima/maxima to be better captured.

From this dataset, heatwaves were defined as the periods when the daily minimum temperature exceeds the 99th percentile of minimum daily temperature over the whole baseline period (1986-2005) for 3 days or more. This relaxes the restriction applied in previous reports whereby only summer months were considered (December, January, and February in the southern hemisphere; June, July, and August in the northern hemisphere). This makes it possible to capture hot seasons in regions where these do not coincide with those months (notably parts of India).

The gridded 99th percentile of daily minimum temperature, taken from the European Centre for Medium-Range Weather Forecasts (ECMWF) ERA5 dataset, was calculated on a 0.5° x 0.5° global grid for 1986-2005. For each year from 1980 to present, the number of heatwave events and total days of heatwaves per year was calculated according to the definition above.

Inspection of the data has shown that increasing heatwave length can result in fewer discrete heatwave events as they merge into single long events – this is therefore better captured by the person-days metric.

The exposure of vulnerable populations to heatwaves is expressed in person-days, i.e. as number of days of heatwave times the number of people affected. This captures the changes in length as well as in frequency of heatwaves.

Population data has been extended in the 2020 Lancet Countdown report by merging the NASA GPWv4 dataset (valid from 2000 to present) with the ISIMIP Histsoc dataset (valid for 1980-2000).[4,5] To capture the demographic distribution of vulnerable populations the spatial distribution of demographics from NASA GPWv4 (valid for 2010) was merged with the temporal trends from the United Nation World Population Prospects (UN WPP; valid for the whole considered period). This makes it possible to calculate exposure trends from 1980 to the present day.

To combine the UN WPP demographics with the GPWv4 demographics, for each country the proportional change in fraction of demographic in each age band relative to 2010 was computed as:

$$\delta^{wpp}_{year,country,age} = f^{wpp}_{year,country,age} / f^{wpp}_{2010,country,age}$$

where:

- $\delta^{wpp}_{year,country,age}$ is the ratio of change in demographic for a given age and and country from the UN WPP dataset
- $f^{wpp}_{year,country,age}$ is the fraction of population in the UN WPP dataset for a given age band, country, and year
- $f^{wpp}_{2010,country,age}$ is the fraction of population in the UN WPP dataset for a given age band, country for the year 2020

The gridded demographic fraction was then calculated relative to the 2010 demographic data given by GPWv4.

For each subset of cells corresponding to a given country *c*, the fraction of population in a given age band is calculated as:

$$f^{gpw}_{year,c,age} = \delta^{wpp}_{year,country,age} * f^{gpw}_{2010,c,age}$$

where:

- $f^{gpw}_{year,c,age}$ is the fraction of the population in a given age band for given year, for the grid cell c.
- $f^{gpw}_{2010,c,age}$ is the fraction of the population in a given age band for 2010, for the grid cell c.

The matching between grid cells and country codes was performed using the GPWv4 gridded country code lookup data and country name lookup table. The final dataset is assembled by combining the cells from all countries into a single gridded time series. The trends in change in number of persons over 65 are shown in Figure 3 and Figure 4. As the population data is discontinuous there can be some inconsistencies between the pre and post 2000 values. Therefore, the indicator is presented as exposure to change rather than change in exposure, as this avoids calculating changes in population across the data discontinuity. Furthermore, the demographic data are continuous across the time period as they are standardised on the UN WPP model.

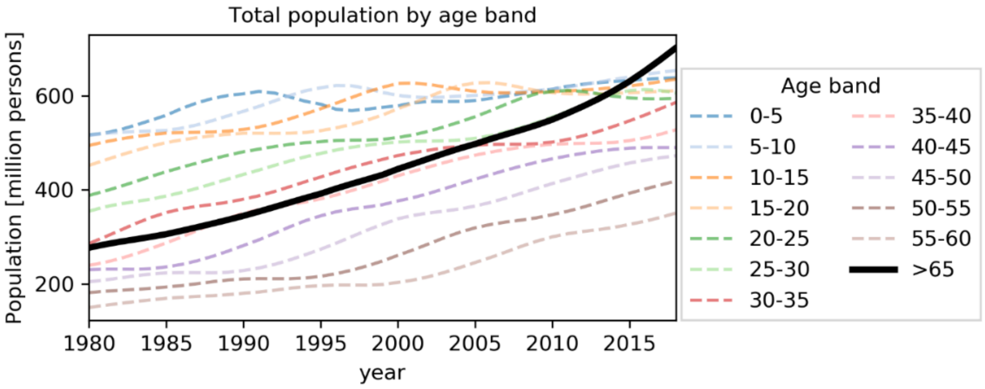

Figure 3: Total population by age band, age band for people over 65 highlighted (solid black line)

.

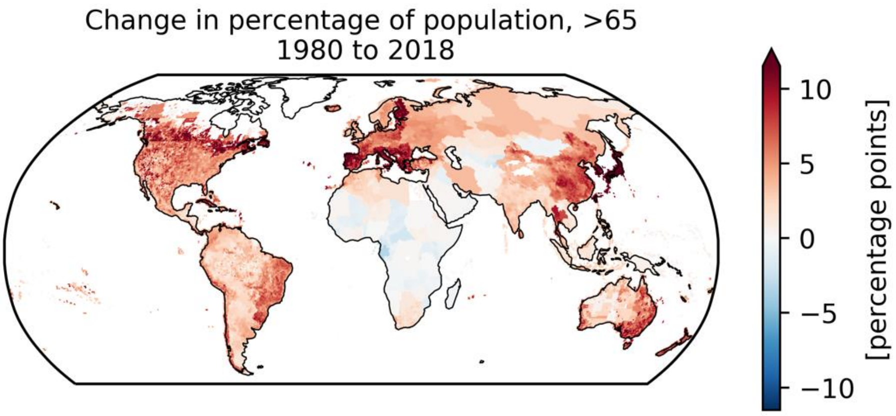

Figure 4: Change in percentage points of the percentage of population over 65 between 1980 and 2018 (start and end of the period studied).

Data

1. Climate data from the European Centre for Medium-Range Weather Forecasts (ECMWF) ERA5 reanalysis.[2]
2. Population data from the NASA Socioeconomic Data and Applications Center (SEDAC) Gridded Population of the World (GPWv4) and The Inter-Sectoral Impact Model Intercomparison Project (ISIMIP) Histsoc dataset.[4,5]
3. Demographic data from the United Nation World Population Prospects (UN WPP).[13]

Caveats

As two distinct sources where used for population data there may be some inconsistencies between the pre and post 2000 values. Therefore, the indicator is presented as exposure to change rather than change in exposure, as this avoids calculating changes in population across the data discontinuity. Furthermore, the demographic data are continuous across the time period as they are standardised on the UN WPP model.

Future form of the indicator

Future iterations of this indicator could take advantage of the upcoming extension of the ERA5 dataset back to 1950. Combined with suitable population and demographic data (yet to be determined) this would give a more complete picture of the changing exposures. Further work may also consider the differences in health risks resulting from heatwave exposure related to the differences between the readiness of country health system and the prevalence of health conditions putting people at risk from heatwave (such as existing cardiovascular and respiratory conditions). Future developments could also increase the resolution to 0.25x0.25°.

Additional analysis

Improvements to the input data and method for the 2020 indicator mean that numbers are not directly comparable with the results of the previous Lancet Countdown publications, all years have therefore been re-calculated for the current publication.

Mapping the change in number of heatwaves in 2019 (Figure 5) highlights the particularly large anomalies (over 20 days more heatwave days relative to the baseline) experienced in the Middle East and the west coast of India in 2019, as well as many significant events across the globe.

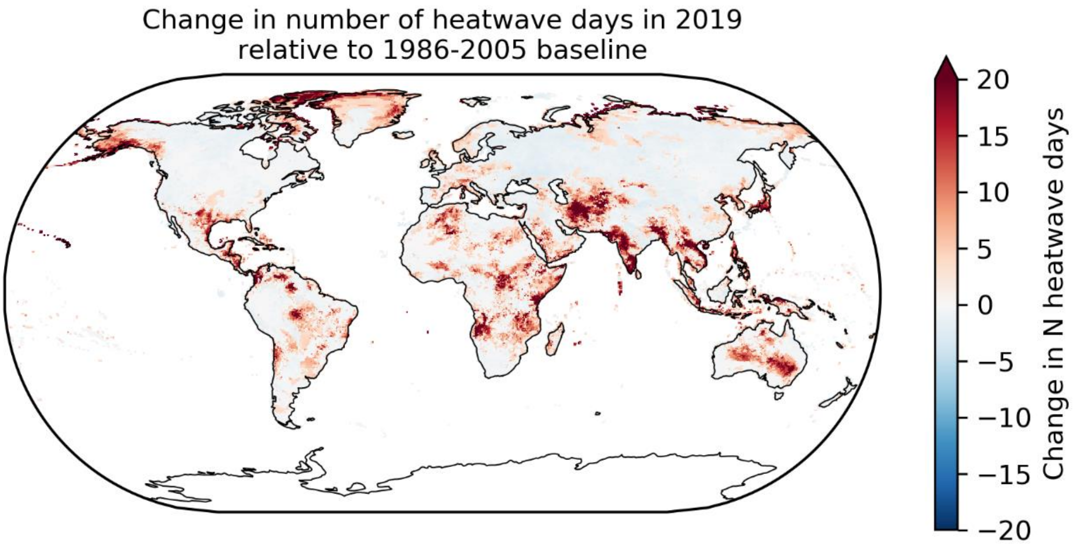

Figure 5: Map of the change in number of heatwave days over land in 2019 relative to the 1986-2005 baseline.

As expected, the use of the full year of data increases the absolute number of heatwave days counted but does not globally affect the trends in heatwave occurrence (Figure 6). The same is broadly true of the exposure weighted trends in heatwave occurrence (Figure 7).

There is however an exception for the year 2016, where the new method indicated a significantly higher number of heatwave days than previously. As can be seen in Figure 8 the new method reveals a number of areas with significantly higher heatwave counts when all months are taking into account. A large part of the new events are heatwave events in April and May 2016 in India and south east Asia (notably Thailand)[14,15] (Figure 9), where as many as 50 additional heatwave days were counted relative to the previous method.

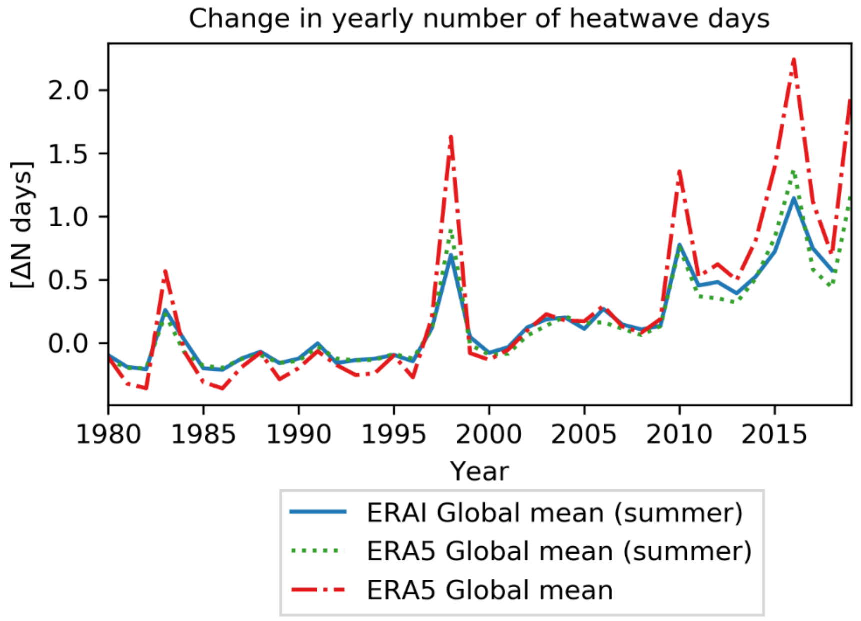

Figure 6: Comparison of global mean of change in number of heatwave days per year relative to baseline between the method of using summer months with the ERA Interim dataset (ERAI), summer months using ERA5, and all months using ERA5.

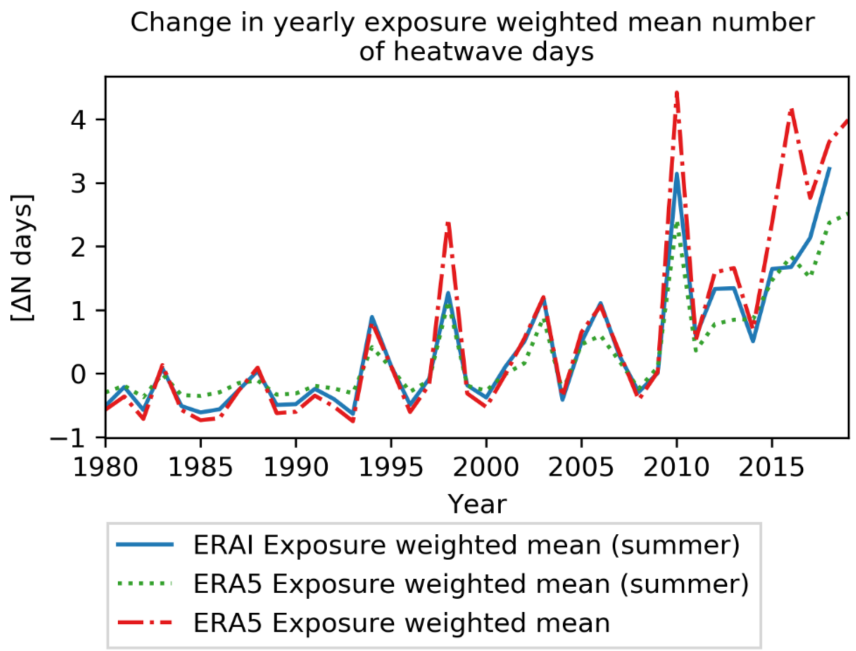

Figure 7: Comparison of exposure weighted mean of change in number of heatwave days per year relative to baseline between the method of using summer months with the ERA Interim dataset (ERAI), summer months using ERA5, and all months using ERA5.

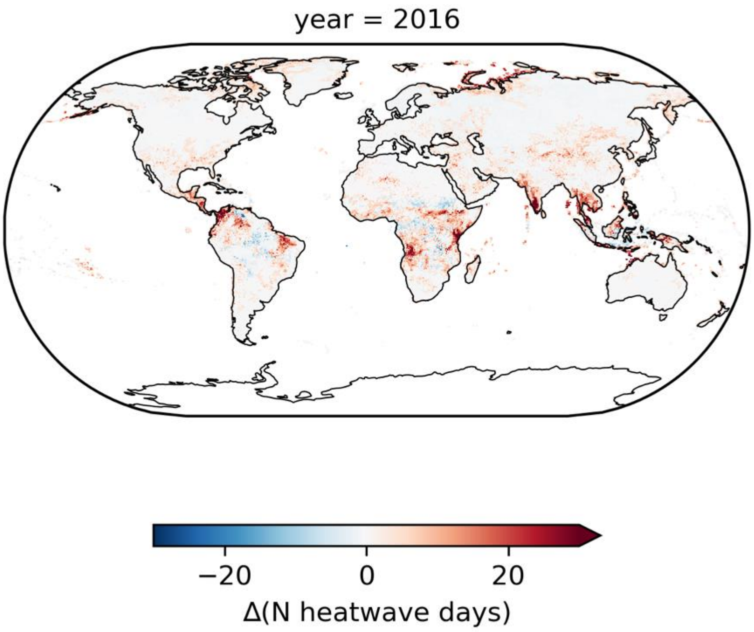

Figure 8: Comparison map for 2016 of number of heatwave days counted using the all months compared to the summer months, calculated as number of days for all months method minus number of days for summer months method.

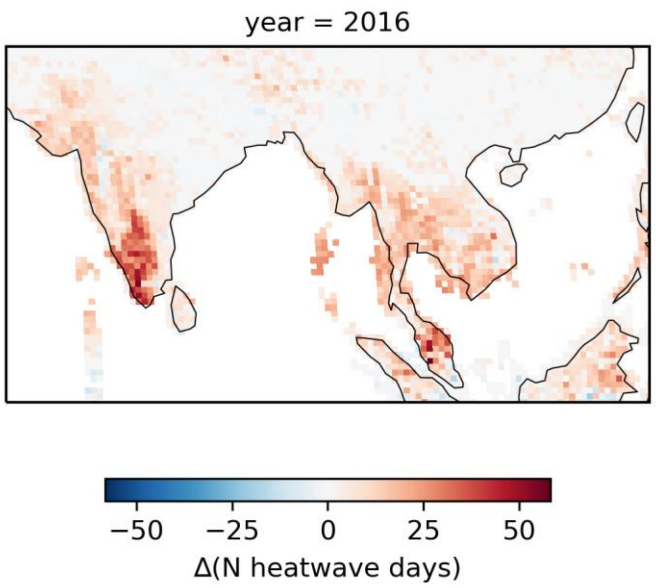

Figure 9: Zoomed map of the 2016 comparison between old and new methods highlighting the newly captured heatwave events over India and Thailand.

In previous Lancet Countdown reports, the exposures were reported as person-events. These are shown in Figure 10, as calculated using the new ERA 5 data and heatwave definition (i.e. using all months of the year). There was a change of 475 million heatwave exposure person-events in 2019 and 377 million exposure person-events in 2018 relative to the baseline.

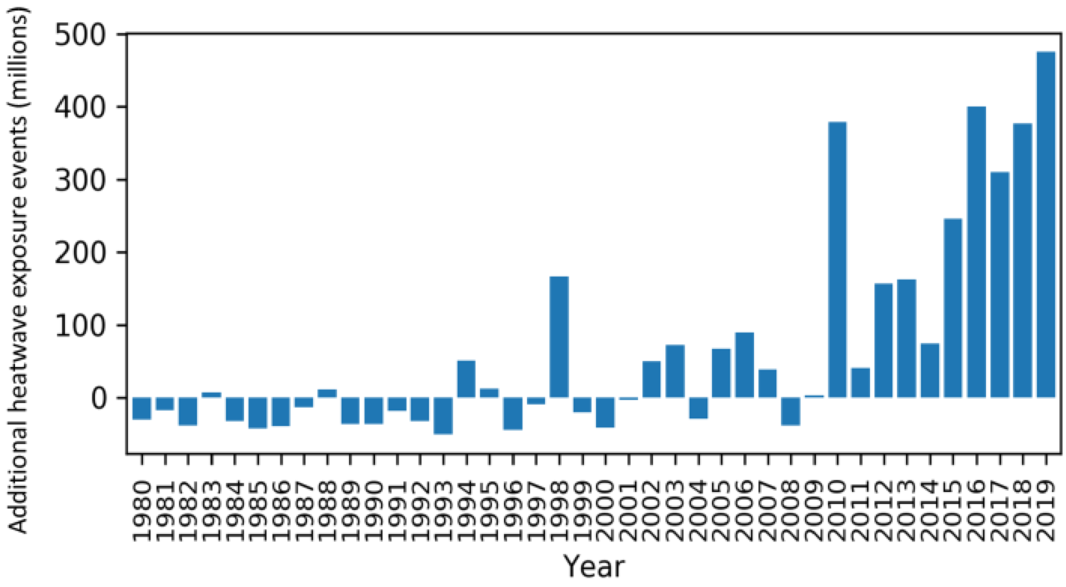

Figure 10: Change in exposure to number of heatwave events.

Indicator 1.1.3: Heat-Related Mortality

Methods

This is the first year this indicator has been included in a Lancet Countdown report.

The indicator tracks the global total number and spatial pattern of heat-related mortality from 2000 to 2018. The method is as follows.

Heat-related mortality in one day *E* is expressed as

$$\mathrm{E} = \mathrm{y}_0 \times \mathrm{Pop} \times \mathrm{AF} \qquad (1)$$

Where y_0 is the baseline daily mortality rate *Pop* is the population size and *AF* is the attributable fraction. y_0 is computed as the yearly non-injury mortality rate from mortality rate from the Global Burden of Disease (GBD) data, divided by 365, and then multiplied by 0.88, as the minimum mortality rate at optimum temperature is approximately 0.88 average daily mortality, as described by Honda et al. (2014).[16] As yearly mortality rate from the GBD is only available to 2017, it was assumed that the mortality rate in 2018 remained the same as 2017.

AF is calculated via the relative risk (*RR*) which represents the increase in the risk of mortality resulting from the temperature increase. *RR* is regressed as $\mathrm{RR} = \exp^{\beta(t-OT)}$, so *AF* is calculated as

$$\mathrm{AF} = \frac{\mathrm{RR} - 1}{\mathrm{RR}} = 1 - \exp^{-\beta(t-OT)}$$

where *t* is the daily maximum temperature, *β* is the exposure-response function and OT is optimum temperature, and both parameters were adopted from Honda et al. (2014).[16] The method was applied to gridded daily temperature data from the ECMWF ERA5 dataset and gridded population data the hybrid of NASA GPWv4 and ISIMIP Histsoc datasets as described in Indicator 1.1.2. As the indicator focuses on population that is 65 years old or older, age-structure data from the United Nations World Population Prospects was also used.

The heat-related mortality was first calculated at grid level at 0.5° spatial resolution. Then it was accumulated to global level to produce a time-series analysis.

Data

1. Climate data from the European Centre for Medium-Range Weather Forecasts (ECMWF) ERA5 reanalysis.[2]
2. Population data from the NASA Socioeconomic Data and Applications Center (SEDAC) Gridded Population of the World (GPWv4) and The Inter-Sectoral Impact Model Intercomparison Project (ISIMIP) Histsoc dataset.[4,5]
3. Demographic data from the United Nation World Population Prospects (UN WPP).[13]
4. Mortality rate data is from the Global Burden of Disease.[17]

Caveats

First, only on exposure-response relation parameter from one Japanese study was used for the whole world. It may not be suitable for other countries and regions worldwide. Second, only the heat-related mortality of the over 65 population was calculated this time. Third, this analysis assumes a constant While there is emerging evidence for declining exposure-response functions over time, data remain insufficient to take this into account quantitatively. Further work is required to include other population groups at risk of heat-related mortality.

Future form of the indicator

One possible improvement of the indicator would be to use more localised exposure-response functions in more places worldwide when these functions become available.

Another improvement could be to calculate the mortality for all ages, not only for people over 65 years old.

Indicator 1.1.4: Change in Labour Capacity

Methods

The analysis is based on 68,940 grid cell data (0.5 x 0.5 degrees with boundaries exactly on the degree line and half degree line) for climate and population. The focus is on trends since the end of the 20th century and on a method that can calculate labour capacity loss at country level. The model data chosen for the calculations was the ERA5 reanalysis hourly data on single levels (3A edition downloaded July 2020),, and the analysis method is described in detail in the paper by Kjellstrom et al., 2018.[18]

Analysis starts from hourly estimates of temperature (t2m) and dew point (d2m). These inputs are used to derive hourly estimates of the heat stress index Wet Bulb Globe Temperature (WBGT) and work loss factor (WLF) at three different metabolic rates under the assumptions that workplace heat exposure is in the shade and air movement is 1 m/s (approximately the speed at which arms and legs move during work). The data was aggregated to provide estimates of mean annual WBGT and mean annual WLF between the hours of 7 am - 7 pm local solar time for each grid-cell.

Exposure was assumed to be atmospheric heat in the shade or indoors (i.e. incoming heat radiation from the sun is absent) without effective air conditioning. The impact of heat on labour capacity depends on clothing (assuming light clothing for all) and metabolic rate based on physical work activity. Labour is divided into four sectors: service (metabolic rate at 200W), manufacturing (300W), agriculture (400W) and construction (400W).

The function relating WLF (the fraction of work hours lost) to an hourly WBGT level is given by the cumulative normal distribution (ERF) function:

$$Loss\ fraction = \frac{1}{2}\left(1 + \mathrm{ERF}\left(\frac{\mathrm{WBGThourly} - \mathrm{WBGTaver}}{\mathrm{WBGT}_{\mathrm{SD}} * \sqrt{2}}\right)\right)$$

Where WBGTaver and $\mathrm{WBGT}_{\mathrm{SD}}$ are the parameters (Table 1) in the function for a given activity level.

Table 1: Input values for labour loss fraction calculation

**Metabolic rate**	**WBGTaver**	**WBGT SD**
200 Watts	35.533	3.948
300 Watts	33.492	3.948
400 Watts	32.465	4.1607

For each grid cell, the working age population (15+ years old, from UN demographic data) for each time period is used as input data as well as the percentages of people in this age range working in the 4 sectors (based on ILOSTAT data for agriculture, construction, manufacturing and services). Populations in grid cells that overlap country borders have been apportioned to the countries involved based on population distribution within the cell.

The total yearly work hours lost for each sector and country are calculated by first, for each grid cell, multiplying each employment sector population by the relevant work loss factor and then, second, summing the resulting sector work hours lost over all grid locations in each country (and in all countries together).

Data

1. Climate data from the European Centre for Medium-Range Weather Forecasts (ECMWF) ERA5 reanalysis.[2]
2. Grid cell based population data was provided from the Gridded Population of the World (GPWv4) database from CIESIN at Columbia University, New York.[19]
3. Sector employment data was provided from ILO, ILOSTAT, (ILO, 2020).[20]

Caveats

The distribution of agricultural, construction, manufacturing and service sector workers is only reported at country level, hence this proportion is distributed evenly to all grid cells within each country.

Future form of the indicator

This indicator will be updated in future to show the number of workers affected globally and in more countries.

Additional analysis

The global trends of work hours lost (WHL) in the four sectors is shown in Figure 11. Agriculture dominates, but is flattening due to the reductions of the agricultural workforce in many low and middle income countries. The impact of rising heat is increasing the fastest in construction.

Because of its definition this indicator is influenced by the changes in population numbers and the distribution of the workforce within countries as well as climate change. The sectors most likely to be affected by increasing heat are agriculture and construction. WHL was calculated as a percent of potential work hours and found for India that the impact on manufacturing workers went from 3.5% in 2000 to 4.5% in 2019, and for agriculture and construction work in the shade the WHL went from 6.5 to 8.2%. Another way of interpreting the trends is to calculate the impacts in 2000 and 2019 using the same population and workforce distribution for both years. For India the trend of increased WHL if populations and sector distribution changes was 1.4 times as in Table 1, while analysis with fixed population and sectors produces a 1.3 times increase. Each country will have different trends. In the next report this will be explored this further. In addition, data will be included the ClimateCHIP website: www.ClimateCHIP.org

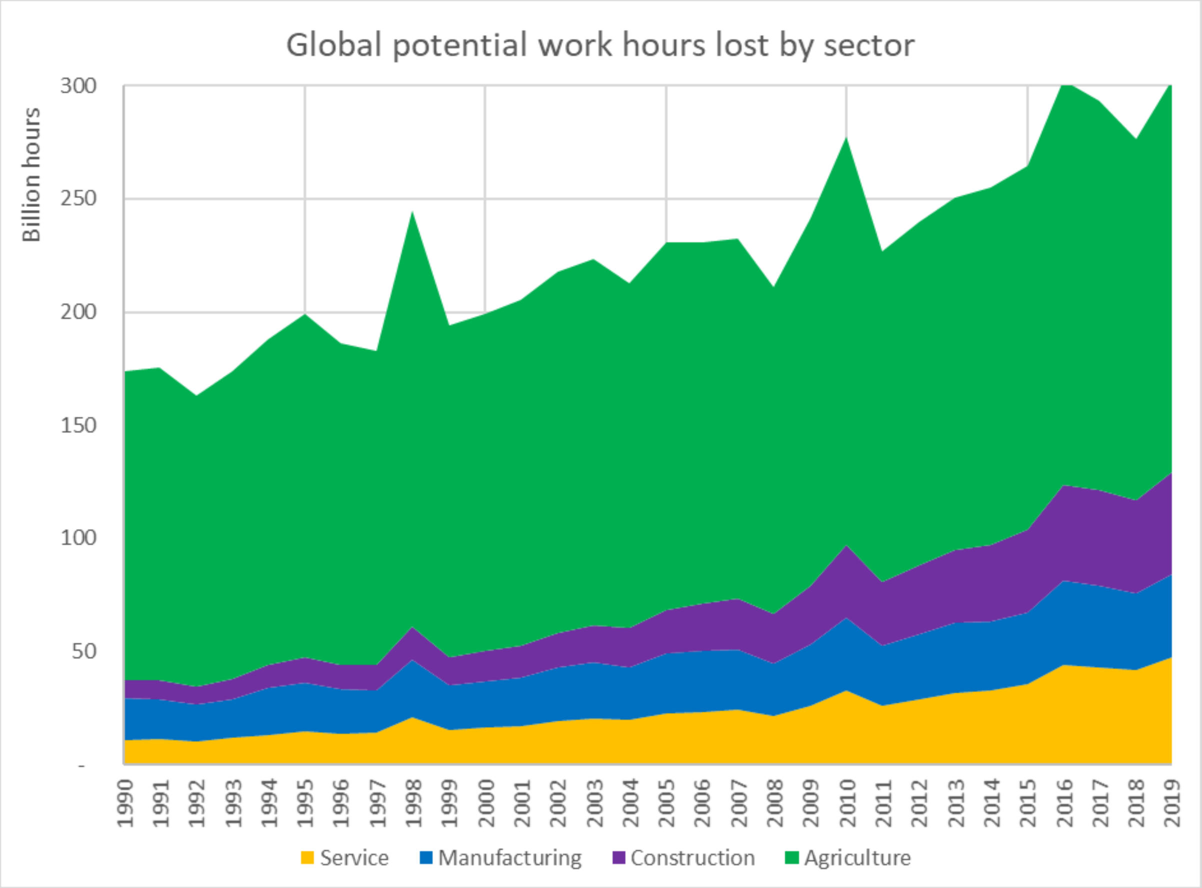

Figure 11: Global potential work hours lost (millions) due to heat 1995-2019.

Due to its definition this indicator is influenced by the changes in population numbers and the distribution of the workforce within countries as well as climate change. WLF (work loss fraction) is defined as the fraction of workhours lost for one worker at a specific metabolic rate, and thus describes work capacity loss due to heat independently from population and employment statistics. Figure 12: Global work loss fraction by sector. shows global WLF trends attributable to climate alone.

Country-specific WHL trends can vary greatly depending on whether population trends are included. For example, between 1990 and 2019 the WHL in **India** (where the most losses occur) without population or sector changes increase from 57 to 92 billion hours (+53%), rather than to 118 billion hours (+106%) if population and sector changes are included.

Countries have different WHL responses to climate change with and without population influences. The next report will explore this further.

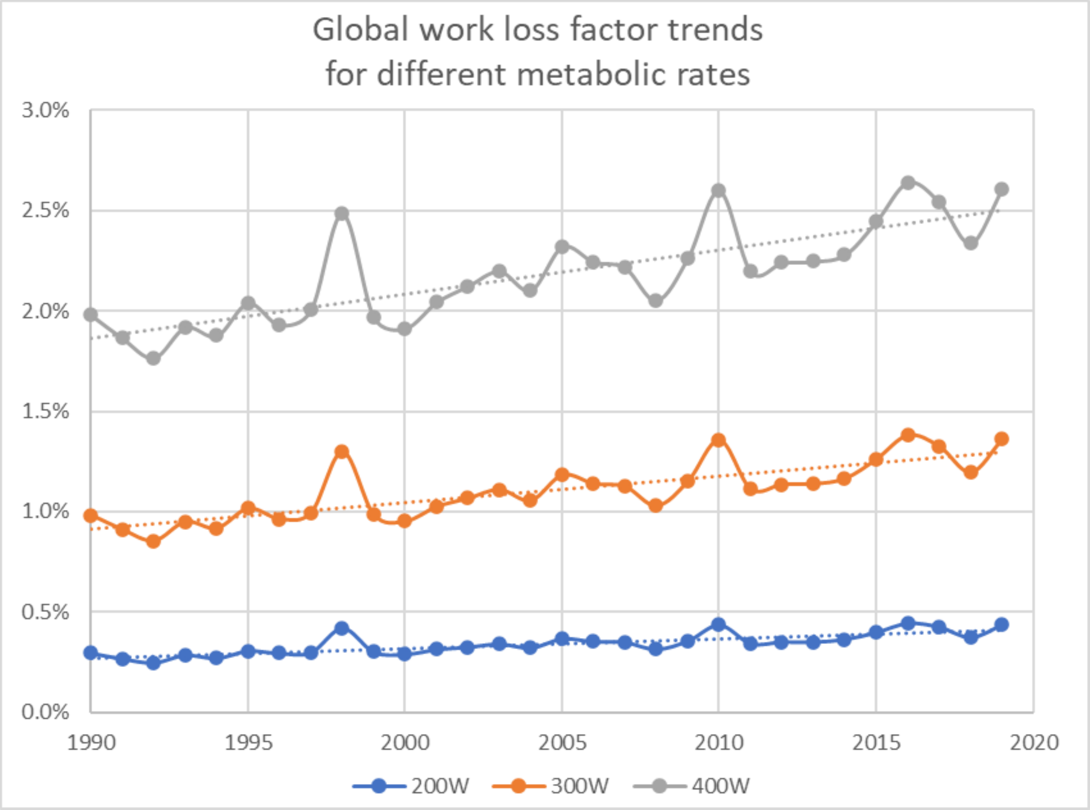

Figure 12: Global work loss fraction by sector.

1.2: Health and Extreme Weather Events

Indicator 1.2.1: Wildfires

Methods

This indicator has been improved from the 2019 Lancet Countdown report to account for both population exposure to wildfire and fire danger risk at the global scale.[1]

The change in population exposure to wildfire is represented as the change in the average annual number of days people were exposed to wildfire in each country. Satellite-observed active fire spots were aggregated and spatially joined with gridded global population data on a global 10 km x 10km resolution grid. Grid cells with a population density ≥ 400 persons/km^2 were excluded to remove urban heat sources unrelated to wildfires. The mean annual number of person-days exposed to wildfire during the most recent four years was compared with the baseline period of 2001-04.

The fire danger risk is represented in terms of the Fire danger index (FDI). Provided by ECMWF ERA5 atmospheric reanalysis, FDI is a numeric rating with values 1-6 representing very low, low, medium, high, very high and extreme fire danger risk, respectively. Daily FDI data, available from 3rd January 1979 through 26th December 2019 worldwide, was aggregated so as to obtain the yearly number of days of each fire danger risk level at every 0.25° x 0.25° grid cell. The changes in mean number of days exposed to very high or extremely high fire danger risk (defined as FDI ≥ 5) were collected for the most recent available period, 2016 to 2019, and compared with a baseline from 2001 to 2004.

Gridded population density data (i.e., population count per square kilometre) from NASA SEDAC GPW v4.11 dataset, was retrieved for the years 2000, 2005, 2010, 2015, and 2020. The data set with a spatial resolution of 2.5' × 2.5' (around 5km x 5km) was used. Population density data was re-gridded to the spatial resolution of the fire danger data using a conservative method (i.e., the total population is conserved) and further linearly interpolated for each year from 2000-2019. The re-gridded population data was used to calculate population-weighted mean days of fire risk. Similar to wildfire exposure, grid cells with a population density ≥ 400 persons/km^2 were excluded in the calculation of changes in mean number of days exposed to very high or extremely high fire danger risk.

Data

1. Collection 6 active fire product from the Moderate Resolution Imaging Spectroradiometer (MODIS).[21] This contains both Terra (from November 2000) and Aqua (from July 2002) pixels in the same annual file.
2. Fire danger indices historical data produced by the Copernicus Emergency Management Service for the European Forest Fire Information System (EFFIS).[22]
3. Population data from the NASA Socioeconomic Data and Applications Center (SEDAC) Gridded Population of the World (GPW v4.11).[4]

Caveats

Cloud cover may introduce spatial biases into fire exposure estimates. While observing the same fire, Terra and Aqua may report slightly different coordinates of the fire centroid, therefore introducing a double counting issue. This indicator does not quantify the populations exposed to wildfire smoke, which increases morbidity and mortality of cardiovascular and respiratory diseases.[23] Estimating the distribution and population exposure to wildfire remains challenging.

The fire danger index represents a potential fire risk calculated on meteorological parameters. It does not represent actual fire events. The actual fire events can be also influenced by anthropogenic factors, such as human-induced land use and land cover changes, industrial-scale fire suppression, and human induced ignition.

The fire danger index does not account for the potential fertiliser effect of CO_2 and the associated changes in vegetation and thus the fuel load of fire.

The fire danger index does not consider potential changes in lightning ignitions, which can be affected by climate change, but the effect is highly uncertain.

Future form of the indicator

This indicator will be extended to analyse longer term averages. Subnational estimates will be reported to better represent the populations at risk.

Additional analysis

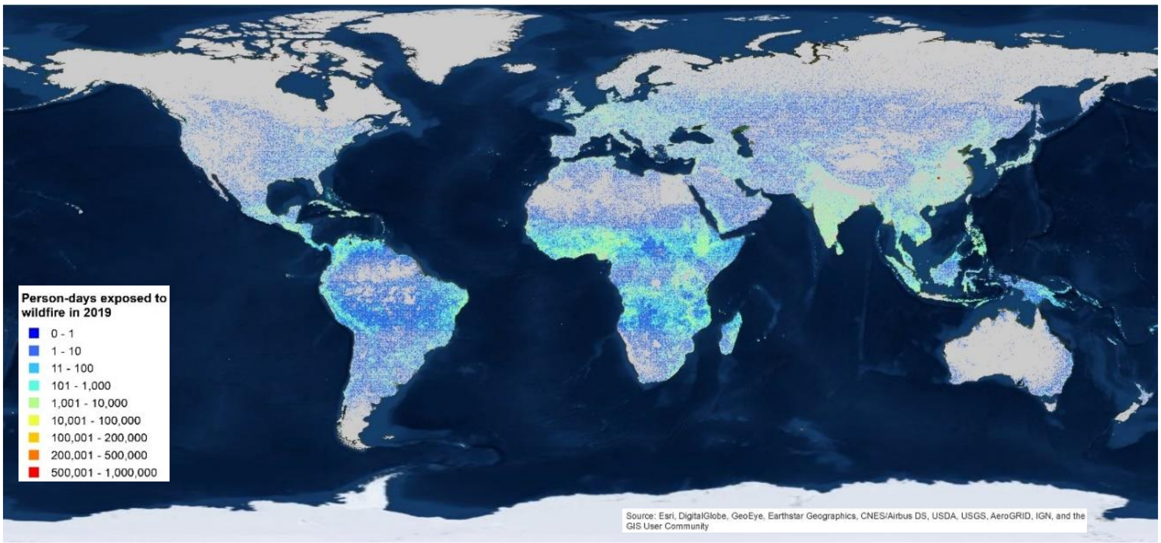

Figure 13: Map showing the average annual number of days people were exposed to wildfires in 2019. Large urban areas with population density ≥ 400 persons/km² are excluded.

Indicator 1.2.2: Flood and Drought

Methods

The data source and the methodology for this indicator have been updated for the 2020 report.

Drought

The drought indicator was improved in the 2020 report, using the Standard Precipitation Evapotranspiration Index (SPEI), to measure meteorological drought. This index was calculated by Beguería et al. using CRU 4.03 precipitation and temperature datasets, on 1 month timescale.[24] It is a multiscalar index, which takes into account both precipitation (using the basis of the more commonly used SPI index) and temperature, to estimate potential evapotranspiration, using the FAO-56 Penman-Monteith method. As such, it captures the impact of increased temperatures on atmospheric water demand, resulting in a more appropriate index to capture the impact of a warming world on drought events than the previously used SPI index. More information on this index and its calculation can be found here: https://spei.csic.es/home.html.

Droughts were defined according to three severity levels using the SPEI thresholds indicated in Table 2, as defined by the Federal Office of Meteorology and Climatology MeteoSwiss.[25] In order to detect excess (unusual) drought events, "excess severe drought events" were defined as yearly counts of months in drought for each grid cell which exceed 2 standard deviations above the mean of the yearly counts of months in drought for the baseline period. Due to the high variability of drought events across the world, and especially due to the impacts of periodic variations such as those generated by the El Niño Southern Oscillation, an extended baseline period of 1950-2005 was used to increase the robustness of this indicator. The excess events were defined for each SPEI severity level of drought independently, and the percentage of land area exposed to excess drought events at the different severity levels were calculated.

Table 2 Summary of drought severity thresholds

SPEI value	description	frequency of event in respective month
< -1.3	severe drought	1-2 x in 20 years
< -1.6	extreme drought	1-2 x in 40 years
< - 2	Exceptional drought	1 x in 50 years or less

An important note. For total drought events, the more areas affected severe droughts are strict subsets of the areas affected by milder droughts in that year. However, for excess droughts, the excess area defined *with respect to that drought's severity level*. As the baseline distributions of drought events are independent for each severity level, the resulting trends are also independent, and the areas affected by excess severe droughts are no longer strict subsets of areas affected by excess mild droughts.

Data

1. Climate data from the European Centre for Medium-Range Weather Forecasts (ECMWF) ERA5 reanalysis.[26]
2. The SPEI index was taken from the The Global SPEI database, SPEIbase (Consejo Superior de Investigaciones Cientificas)[24,27]

Caveats

A limitation of this indicator is that it only captures the impacts of climate change on meteorological drought, but does not capture the impacts of climate change on hydrological or agricultural drought, which can have major health impacts too. Moreover, it does not measure the direct relation between a drought and the population living in drought-affected areas. It is not possible to do a population-based weighting because many people affected by a drought may not live in the area affected – e.g. in the case of droughts affecting agricultural areas (which are generally sparsely populated) with impacts on the food supply. It is therefore difficult to determine trends in persons exposed to drought impact from the trends of severe drought area.

Further work is required to link reported drought damages in societies to climatic indicators. This would require a better understanding of the exposure factors of populations.

Future form of the indicator

Further development of the indicator will focus on using a combination of indexes that capture agricultural hydrological drought, and meteorological drought, and better capture the health implication of drought events.

As for floods, a forthcoming extension of the ERA5 dataset time coverage back to 1950 will allow to assess any trend on excess of heavy rainfall events over a longer period thus potentially confirming upward trend since 2005.

Further developments may also consider a potential integration between flood and drought, as sudden and increasingly unpredictable changes between unusually dry and unusually wet spells have been observed.[28]

Additional analysis

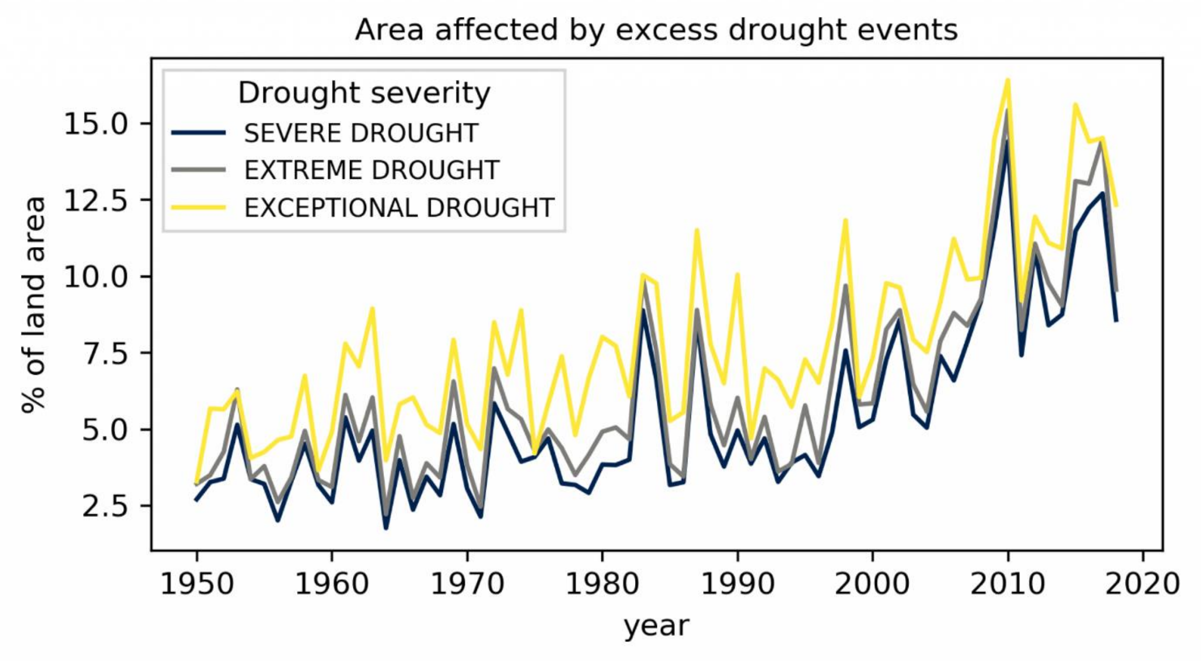

Figure 14: Percentage of land area affected by excess drought events.

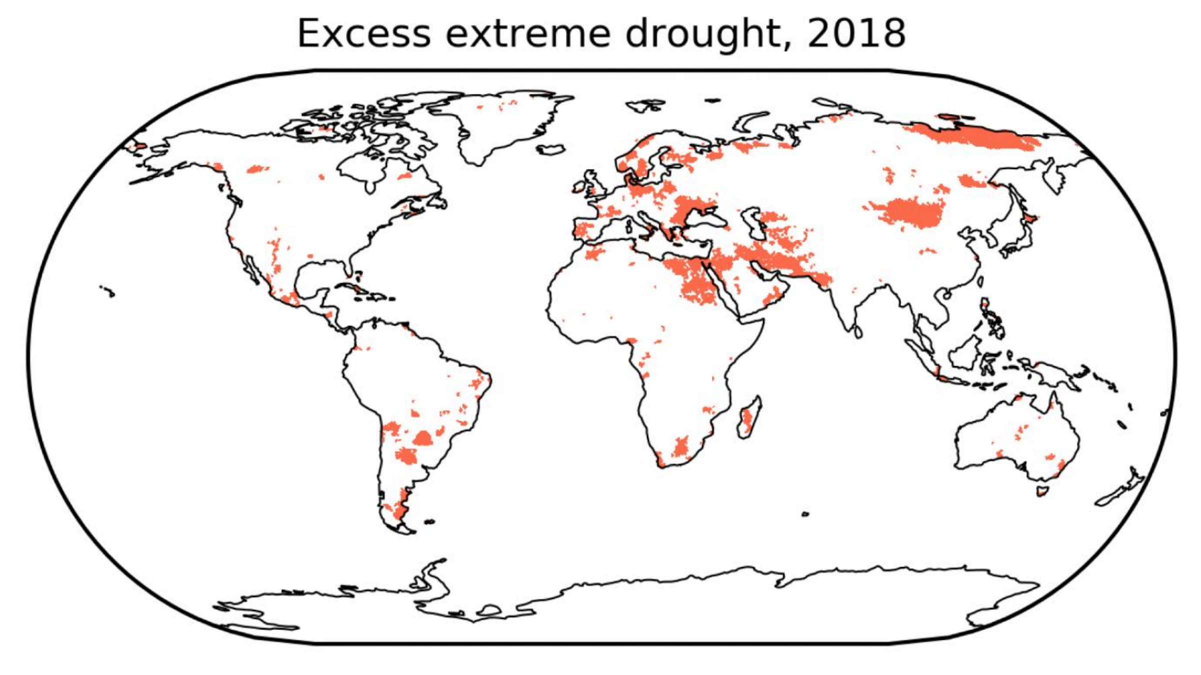

Figure 15: Land area affected by excess drought events.

Indicator 1.2.3: Lethality of Extreme Weather Events

Methods

The methodology for this indicator has been updated from previous reports.[1] The number of occurrences of weather-related disasters (drought, storms, wildfires, floods and extreme temperatures), the number of people affected in each disaster, and the lethality of these events have been grouped according to the average annual health expenditure per capita (in PPP) and health expenditure as % Gross Domestic Product (GDP) for each country over the period from 2000 to 2017. This data was obtained from the World Health Organization's Global Health Observatory. The choice of grouping according to these metrics was based on the data presented in the supplementary information of the 2018 Lancet Countdown report.[7] This indicated that grouping countries according to which quartile of health expenditure per capita (in PPP) and as % Gross Domestic Product (GDP) individual countries fell into showed distinct differences in lethality.

The methodology uses data from the Centre for Research on the Epidemiology of Disasters (EM-DAT).[29] Here, deaths, as proxy of the lethality of weather-related disasters, are defined as the number of people who lost their life because the disaster happened. People affected are defined as those requiring immediate assistance during a period of emergency; hence requiring basic survival needs such as food, water, shelter, sanitation and immediate medical assistance.

Detection and attribution studies of events which ended between January 2015 and January 2020 and were published in the annual collections of the Bulletin of the American Meteorological Society (BAMS) and/or by the World Weather Attribution (WWA) were reviewed. Where the WWA noted the occurrence of an extreme event, but did not publish an attribution study, a wider review of the literature was conducted and studies on the same event but published by other sources were considered. Where WWA entries linked to external / peer reviewed studies with full details (compared to the rapid analyses available on the WWA website), or when authors of an older study refined methods and republished analyses, the external / peer reviewed / updated sources were used in place of the analyses published on the WWA website / older sources. Studies of events deemed to present a particular threat to human health were included (i.e. extreme heat during summer months, extreme cold during winter months, drought, wildfire, flood, storm, and marine heat), while other temperature anomalies (such as studies of mild winters without known impact to human health) were excluded. Studies that did not quantitatively assess the attribution of climate change to an event were also excluded. Where studies examined multiple events or multiple independent factors contributing to an event, each event or factor is reflected. Where the attribution of an event to climate change was calculated based on the attribution of a sole risk factor for the event, the study is listed only once. Where climate change was found to have various effect on a single event, as for heavy rainfall in Northwest China in 2018, for which climate change decreased the probability of summer decreased heavy rainfall but increased that of daily extremes, then the event is listed under "varied effects".

Data

1. EM-DAT at the Centre for Research on the Epidemiology of Disasters (CRED) at the Université Catholique de Louvain, Belgium.[29]
2. World Health Organization's Global Health Observatory.[30]
3. Detection and Attribution studies were collected from the "Explaining Extreme Events from a Climate Perspective" special supplements to the Bulletin of the American Meteorological Society and the World Weather Attribution website.[31-35]

Caveats

The EM-DAT database contains a number of possible biases. Firstly, there is a possible bias in missing some disaster events because of under-reporting. EM-DAT classifies an event as a disaster if 10 or more people die; 100 or more people are affected; there is a declaration of a state of emergency; or a call for international assistance. Similarly, there are likely biases in how countries report both the number of deaths and people affected. Numbers of deaths for example may not include mortality from the cascading risks of natural hazards or those that occur as a result of longer causal chains from the hazard. Secondly, estimates of the numbers of

people affected have different biases for different country because of how the concept of "affected people" is defined. This must be considered when comparing countries.

Future form of the Indicator

Future efforts will include a comparison of estimates of those exposed with those affected. Additionally, the impact of replacing the number of people killed with the number requiring assistance also explored.

Countries have begun reporting against the Sendai Framework indicators and the disaster risk reduction indicators of the Sustainable Development Goals on the online Sendai Framework Monitor (https://sendaimonitor.undrr.org/). This indicator aims to expand to include country specific progress in vulnerability levels of health service systems to climate risks in relation to this monitoring data.

Additional analysis

The progression of the number of weather-related disasters for each quartile of countries is shown in the figure below. Data is presented as standardised anomalies (Z scores), representing the difference between the variable that year and average of the variable from 1990-2019, normalised by the standard deviation of the variable over the same period. Only statistically significant (at 0.05 significance level) linear trends over time are shown.

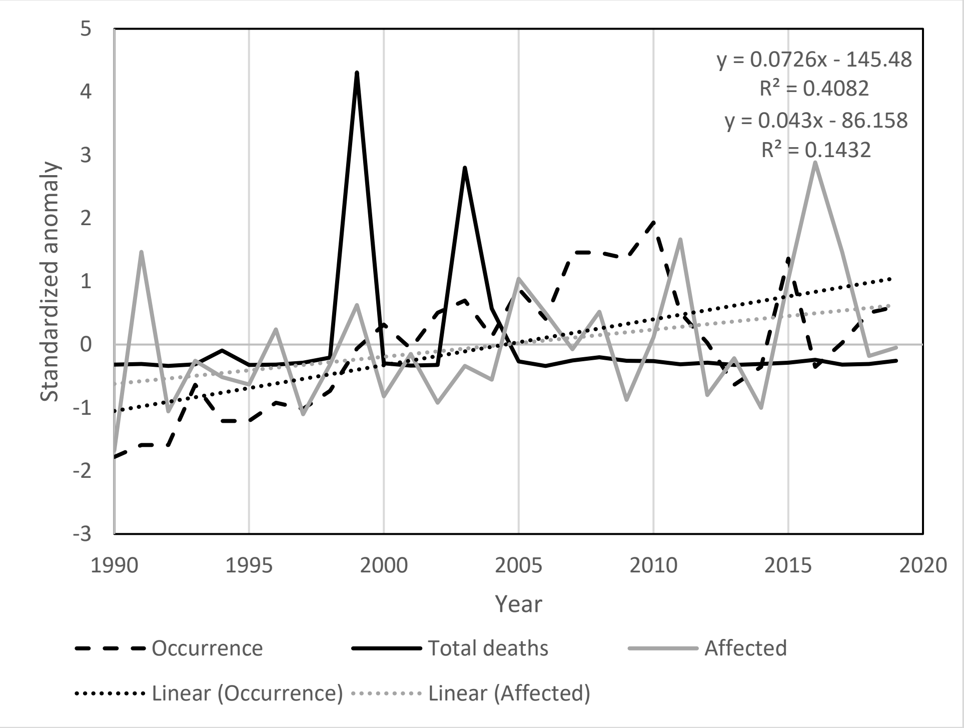

Figure 16: Time series of standardised anomalies of the deaths, occurrences and number of people affected by weather-related disasters for those countries in the first quartile of countries ranked according to the percentage change in annual health expenditure (CHE) as % Gross Domestic Product, from 2000-2017. The standardizing period for anomalies is from 1990-2019.

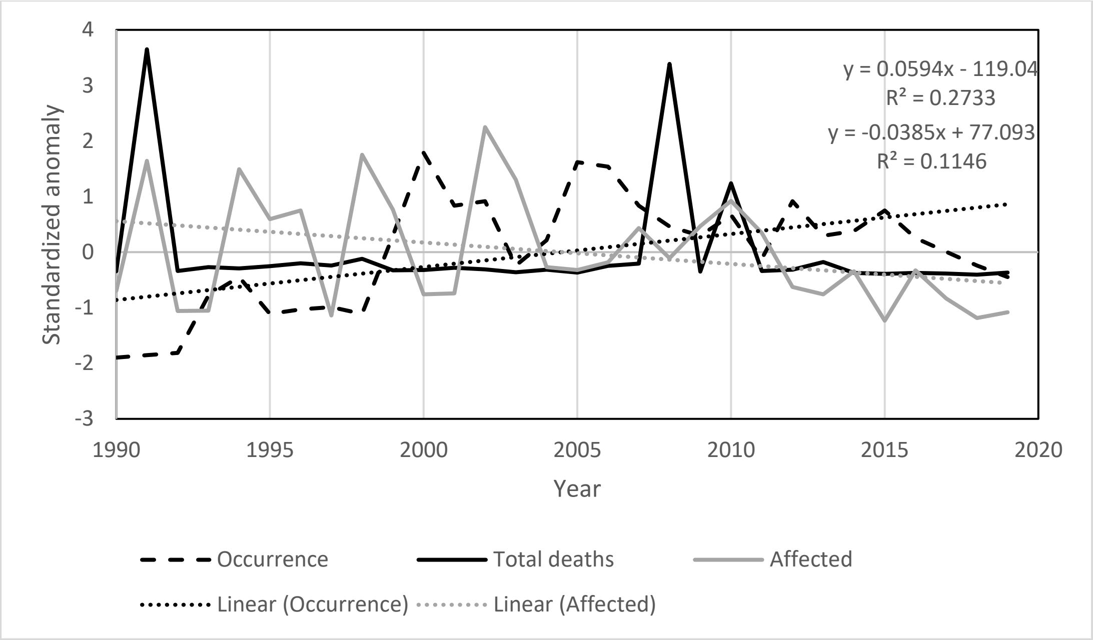

Figure 17: Time series of standardised anomalies of the deaths, occurrences and number of people affected by weather-related disasters for those countries in the fourth quartile of countries ranked according to the percentage change in annual health expenditure as % Gross Domestic Product, from 2000-2017. The standardising period for anomalies is from 1990-2019.

1.3: Climate-Sensitive Infectious Diseases

Indicator 1.3.1: Climate Suitability for Infectious Disease Transmission

The disease indicators will be reported upon annually and assessed against the baseline data and trends presented here. Other climate-sensitive infectious diseases in addition to malaria, Vibrio, and dengue will be added through time and the current indicators refined. In future, it is intended to expand efforts to project trends (as for dengue) using available models (e.g., RCPs from AR5). In addition, efforts will expand to link environmental suitability information to disease outcomes e.g., via disease case or surveillance data. Numerous jurisdictions currently already undertake indicator (e.g., annual country- or regional-level reporting of confirmed human cases), event-based (e.g., outbreak investigation and 'epidemic intelligence'), and biosecurity (e.g., sentinel site) surveillance for infectious diseases, vectors, or key zoonotic hosts. Many of these datasets and methods of analysis could be made available and leveraged in future for the Lancet Countdown. For example, EU member states already report cases of notifiable diseases, zoonotic diseases, and outbreaks of food-borne and zoonotic disease, while vector surveillance remains voluntary.[36]

Dengue

Methods

Cases of dengue have doubled every decade since 1990, with 58·4 million (23.6 million–121.9 million) apparent cases in 2013, accounting for over 10,000 deaths and 1.14 million (0.73 million–1.98 million) disability-adjusted life-years.[37] Beside global mobility, climate change has been suggested as one potential contributor to this increase in burden.[38] *Aedes aegypti* and *A. albopictus*, the principal vectors of dengue, also carry other important emerging or re-emerging arboviruses, including Yellow Fever, Chikungunya, Mayaro, and Zika viruses, and are likely to be similarly responsive to climate change.

Methods for calculating vectorial capacity (V) follow Rocklöv et al. (2019).[39] V refers to a vector's ability to transmit disease to humans. It incorporates interactions between host, virus, and vector, assuming that all three of these elements are present. Specifically, V represents the average daily number of secondary cases generated by one primary case introduced into a fully susceptible population, and is expressed as:

$$V = ma^2 b_m p^n / -lnp$$

where a is the average vector biting rate, b_m is probability of vector infection and transmission of virus to its saliva, p is the daily survival probability, n is the duration of the extrinsic incubation period (EIP), and m is the female vector-to-human population ratio. Here m is set to 1 assuming the female vector and human population are constant. Detailed model description and explanation can be found in Rocklöv et al. (2019).[39] In this application, the time unit is 1 day and each of the vector parameters depends on temperature, with parameter values derived from the literature, typically from experimental data, as described in Liu-Helmersson et al. (2016).[40] Diurnal temperature range (DTR) was reconstructed using a representative daily temperature through a piecewise sinusoidal function based on the monthly average of daily minimum, maximum, and mean observations.

Historical trends were derived by backcasting the models on data from the Climate Research Unit (CRU) online database, time series (CRU-TS 4.03) of gridded (0.5° x 0.5°) monthly averages of daily temperature observations (minimums, maximum, and mean) for the time period 1950-2018.[41]

The annual average V were extracted values per grid cell to *Aedes aegypti* and *Aedes albopictus* presence locations provided in Kraemer et al.(2015).[42] and averaged these values by country to get country-specific trends in V at monthly (seasonality analysis) or yearly time steps from 1950-2018 for each species. 'Global vectorial capacity' indicates globally averaged values across all countries

Data

1. Climate Research Unit (CRU) climate dataset (University of East Anglia).[41]

Caveats

Key caveats and limitations of the V model and its parameterisation are fully described in Liu-Helmersson et al.[40,43] and Rocklöv et al. V should not be confused with actual dengue cases, although it is an indicator of the risk of infection.[39]

Future form of the indicator

This indicator may be updated to include vectorial capacity for the transmission of Yellow Fever, Chikungunya, and Zika. It will also strive to include more model estimates of vector to human densities (m), going beyond the presence / absence approach of vectors used here.

Additional analysis

Suitability across other regions is increasing steadily, with average V over the past 5 years 5-10% higher for A. aegypti in African, Western Pacific and the Americas WHO regions, and >5% for A. albopictus in all WHO regions. For all WHO regions and for both vector species, gains in V have predominantly occurred since the late 1970s (Figure 18, bottom row).

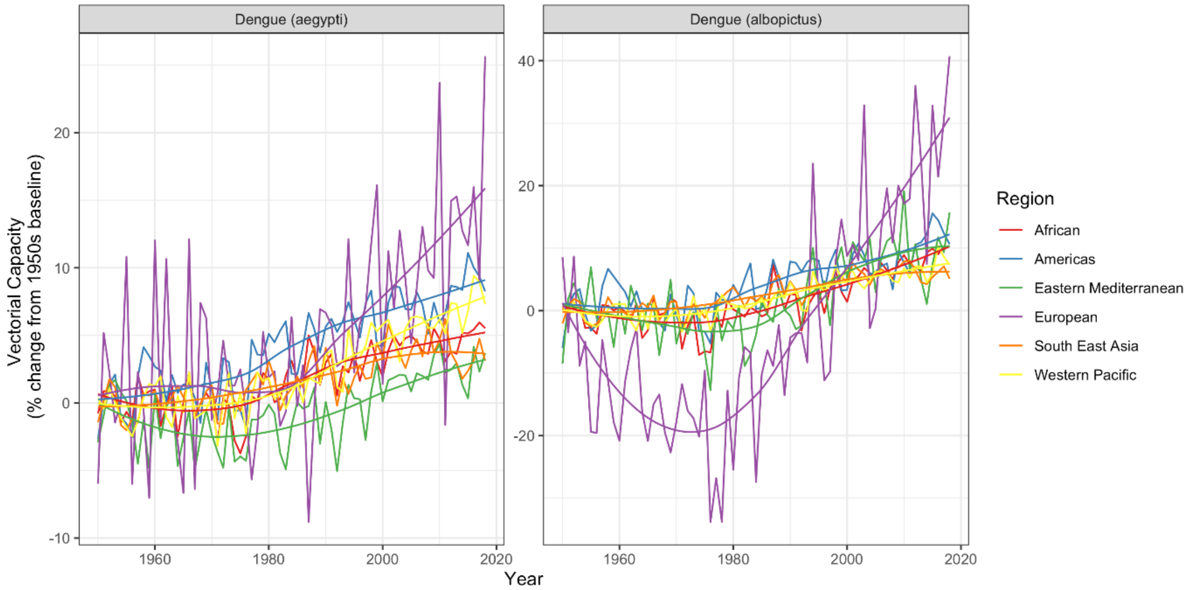

Figure 18: Percentage change in vectorial capacity for dengue transmitted by Aedes mosquitoes from the 1950-1954 baseline, grouped by WHO region.

Malaria

Methods

Temperature, precipitation and relative humidity are climatic factors that impact the abundance and feeding cycle rate of *Anopheles* mosquitoes, which transmit the *Plasmodium* parasites that cause malaria. Temperature also drives the development rate of *Plasmodium* parasites within the mosquito vectors.[44] Temperatures within the range 18°C to 32°C are considered most suitable for *P. falciparum* parasites, which cause the most severe forms of malaria (including cerebral malaria). Below this lower limit the development of the parasite ceases while above 32°C the survival of the mosquito is compromised. Relative humidity greater than 60% is also considered as a requirement for the mosquito to survive long enough for the parasite to develop sufficiently to be transmitted to the human host stage. Rainfall and surface water are needed for the egg laying and larval stages of the mosquito life cycle, with monthly rainfall accumulation of at least 80mm considered more suitable for transmission.[44]

A recent study has found a significant increase in elevation of the lower temperature limits for the development of malaria parasites in Ethiopia.[45] Increasing temperatures in the region are eroding the perceived barrier to malaria transmission, allowing more favourable conditions to begin climbing into densely populated highland areas. The malaria indicator focuses on determining global changes in climate suitability over time between highland and lowland areas in regions that have not yet achieved malaria elimination.

The number of months suitable for malaria transmission per year from 1950-2018 was calculated globally. Suitability is based on empirically derived thresholds of precipitation, temperature and relative humidity for *Plasmodium falciparum*.

Monthly observations of temperature, precipitation and vapour pressure data from the Climate Research Unit (CRU TS4.03) were downloaded using the Koninklijk Nederlands Meteorologisch Instituut (KNMI) Climate Explorer.[41,46] The variables were extracted at a 0.5° spatial resolution over land. Elevation data at a 0.5° spatial resolution was obtained from the University of Washington Joint Institute for the Study of the Atmosphere and Ocean (JISAO).[47]

Relative humidity (RH) was estimated using the formula:[48]

$$RH = \frac{e}{e_{sat}} \times 100,$$

where e is vapour pressure and e_{sat} is saturated vapour pressure (in hPa) at mean air temperature T in °C, given by:

$$e_{sat} = 6.108 \exp[17.27\,T\,/(237.3 + T)]\,.$$

Climatic suitability was defined as the coincidence of precipitation accumulation greater than 80 mm, average temperature between 18°C and 32°C, and relative humidity greater than 60%.[44] The combined values are an indication of the lower limit for potential transmission of *Plasmodium falciparum* (Figure 19).

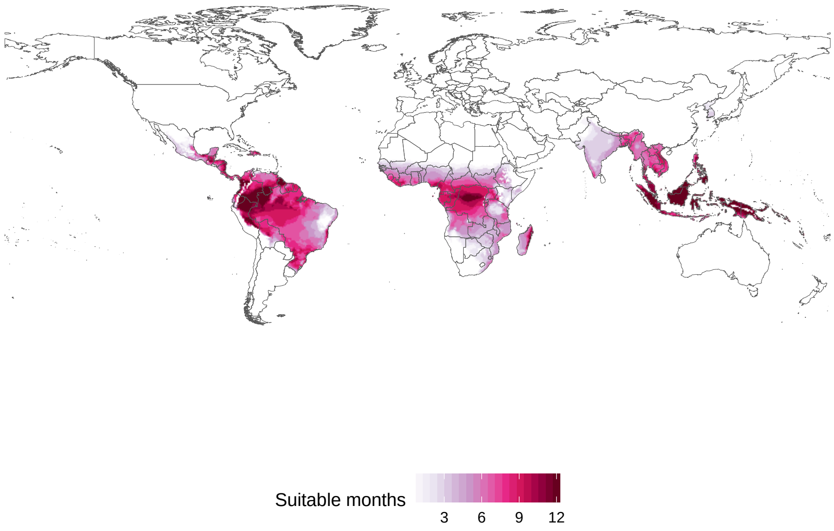

Figure 19: Environmental suitability for the transmission of Plasmodium falciparum in 2017. Number of months with precipitation accumulation greater than 80 mm, average temperature between 18°C and 32°C and relative humidity greater than 60% in 2017.

The mean number of months per year with suitable climate conditions was then calculated for WHO regions with annual malaria incidence rates greater than zero (Figure 20).[49]

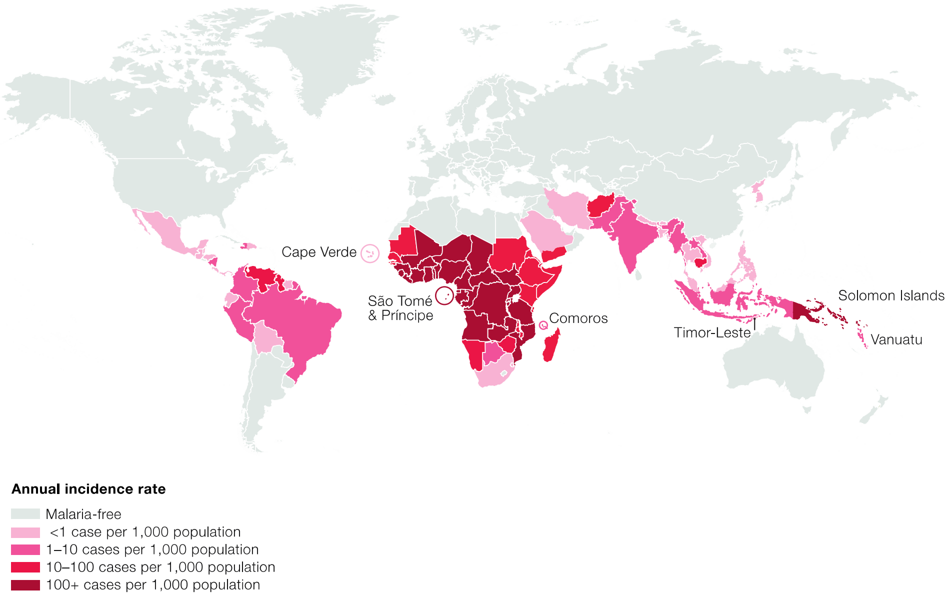

Figure 20: Malaria cases per 1000 total population in 2017 by country. The annual malaria incidence is defined as the number of cases caused by the four human malaria species (Plasmodium falciparum, Plasmodium vivax, Plasmodium malariae, and Plasmodium ovale) per 1000 total population in the year (Source: Feachem et al., Lancet 2019).[49]

Environmental suitability was stratified by elevation to contrast trends in highland areas (≥1500m) and lowland areas (<1500m). Excluding 'malaria free' countries, time series were included for WHO Regions: African Region, Region of the Americas, East Mediterranean Region, South-East Asia Region and Western Pacific region in areas greater than 1,500 metres above mean sea level (see main text).

Data

1. Monthly observations of temperature, precipitation and vapour pressure data from the Climate Research Unit (CRU TS4.03) were downloaded using the Koninklijk Nederlands Meteorologisch Instituut (KNMI) Climate Explorer.[41,46]
2. Elevation data was obtained from the University of Washington Joint Institute for the Study of the Atmosphere and Ocean (JISAO).[47]

Caveats

These results are based on climatic data, not malaria case data. The malaria suitability climate thresholds used are based on a consensus of the literature. In practice, the optimal and limiting conditions for transmission are dependent on the particular species of the parasite and vector.[50] Control efforts might limit the impact of these climate changes on malaria or conversely, the climate suitability may either enhance or hamper control efforts.[51]

Additional analysis

The percentage change figures reported in the main text were calculated relative to a 1950s baseline (5-year average, 1950-1954 compared to 5-year average, 2014-2018) to illustrate the overall trend accounting for interannual variability (Figure 21).

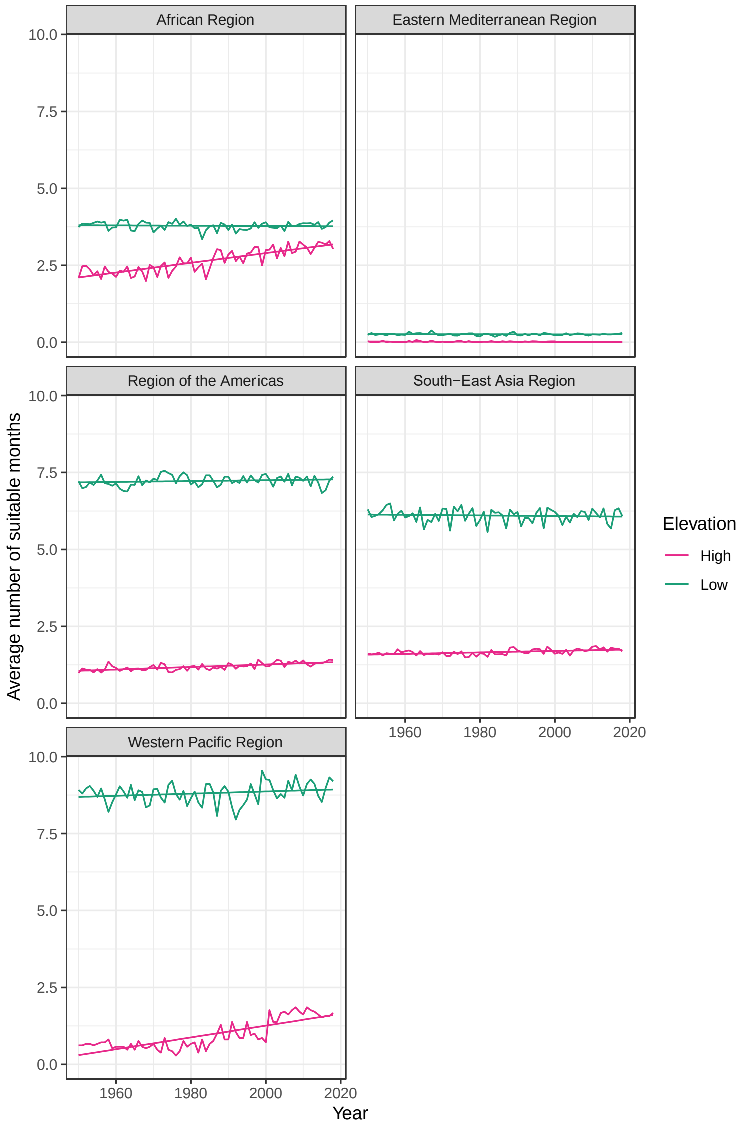

Figure 21: Environmental suitability for malaria 1950 to 2018, grouped by WHO region and elevation (high ≥1500m, low <1500m). Countries considered 'malaria free' are excluded from the analysis.

Vibrio

Methods

Vibrio spp. are globally distributed aquatic bacteria that are ubiquitous in warm estuarine and coastal waters with low to moderate salinity. *V. parahaemolyticus, V. vulnificus*, and non-toxigenic *V. cholerae* (non-O1/non-O139) are pathogenic in humans. These *Vibrio* species are associated with sporadic cases of gastroenteritis, wound infections, ear infections, or septicaemia in circumscribed localities.
Vibrio ecology, abundances, distributions, and patterns of infection are often strongly mediated by environmental conditions. Water temperature, salinity, and turbidity predict the distribution and abundance of *V. vulnificus* in Chesapeake Bay, with the number of infections increasing as a result of recent local warming and changes in rainfall.[52] Increased water temperatures also explain outbreaks of *Vibrio* infections in countries bordering the Baltic Sea,[52] and range expansions the Atlantic Northeast, Pacific Northwest, and Alaska.[53]

This indicator focuses on mapping environmental suitability for pathogenic *Vibrio* spp. in coastal zones globally (<30km from coast).

The indicator uses thresholds of >18°C for Sea Surface Temperature (SST) and <30 PSU for Sea Surface Salinity (SSS). These values were derived on the basis of a consensus in the literature.[54-56] Estimates for SST were obtained from NOAA Optimum Interpolation 1/4 Degree Daily Sea Surface Temperature (OISST) Analysis version 2 for the period 1982-2019. This dataset is provided by the NOAA/OAR/ESRL PSD.[57] The salinity fields were created from daily data obtained from Mercator Ocean Reanalysis.[58]

Here suitability is reported at two levels. First, it was calculated the percentage of coastline globally that experienced suitable conditions for *Vibrio* infections and summarised the results across three latitudinal bands (northern latitudes = 40-70°N; tropical latitudes = 25°S-40°N; and southern latitudes = 25-40°S). Second, suitability in three focal regions in which human *Vibrio* infection is frequently observed, the Baltic Sea, the Pacific northwest and the northeastern coast of the United States (36-50°N) were calculated. For the Baltic (main text), Pacific NW and northeastern coast of the United States, the percentage of coastline suitable for *Vibrio* infections are presented. In addition, the number of days per year suitable for outbreaks is presented for the Baltic (Figure 22). The percentage change figures reported in the main text were calculated relative to a 1980s baseline (5 year average, 1982-86), either an average for the 2010s (5 year average, 2015-2019) to illustrate the overall trend accounting for interannual variability or for the most recent year for which data were available (2019).

Data

1. Estimates for SST were obtained from NOAA Optimum Interpolation 1/4 Degree Daily Sea Surface Temperature (OISST) Analysis version 2 for the period 1982-2019. This dataset is provided by the NOAA/OAR/ESRL PSD.[57]
2. The salinity fields were created from daily data obtained from Mercator Ocean Reanalysis.[58]

Caveats

The results are derived on the basis of suitable SST and SSS conditions only, and do not include other potentially important drivers (e.g. globalisation), environmental predictors of pathogenic *Vibrio* infections (e.g., cholorphyll-*a*, turbidity) nor disease case data. Nevertheless, these associations have been explored and are reported in the supporting references included above.

In the global analysis, the slope of the trendlines over the time series is mostly flat for the tropical/subtropical region and the southern Hemisphere. However, the SST-only suitability shows a strong upward trend in the southern hemisphere, indicating that on average temperature conditions are also improving growth conditions for *Vibrio* in these areas, while SSS is generally limiting. However, locally suitable SSS conditions will also

occur in these regions on the basis of, for example, variation in local rainfall and river runoff, which can make these regions sporadically suitable for *Vibrio* infections.

Additional analysis

In addition to the area suitable for *Vibrio* outbreaks, the number of days suitable per year has doubled in the Baltic region, extending the highest risk season by around 6.5 weeks (from 40.8 days 1982-1986 to 86.4 days in 2015-2019).

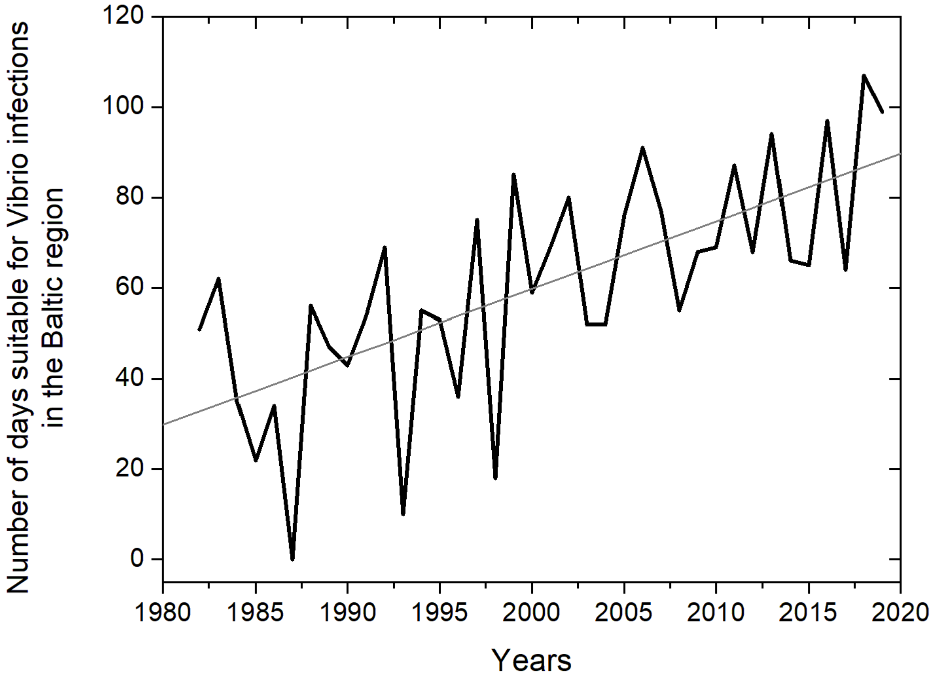

Figure 22: Annual number of days suitable for Vibrio infections in the Baltic Region.

This Latitude-time plot (Hovmoller diagram, Figure 23) indicates poleward expansion of suitable environments for *Vibrio* spp. in this region. For latitudes >39 and similarly to the Baltic Sea, there is a general widening of the *Vibrio* spp. season as well as an increase in the amount of shoreline affected.

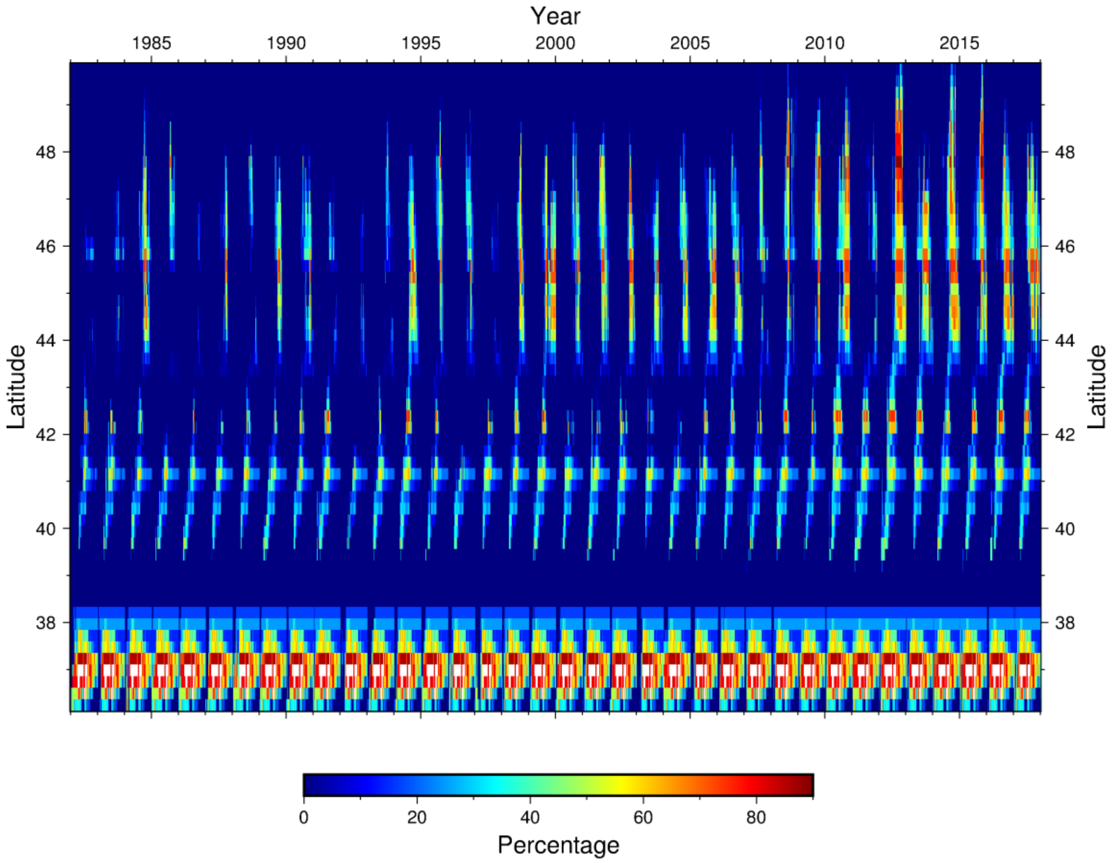

Figure 23: Percentage coastline suitable for Vibrio spp., V. parahaemolyticus, V. vulnificus, and non-toxigenic V. cholerae (non-O1/non-O139), by latitude along the United States northeast coastal region (36N-50N).

Indicator 1.3.2: Vulnerability to Mosquito-Borne Diseases

Methods

This indicator computes the vulnerability of a given country to threats posed by infectious diseases, taking into account core competency in key areas. The key areas are in surveillance, legislation, food safety, human resources, laboratory, point of entry, response, preparedness, risk communication and zoonosis which form part of International Health Regulations (IHR) Core Capacity Monitoring Framework.[11,59] A composite index was computed by taking average of the 11 core competencies. The average core capacities outlined in the monitoring framework has shown protective to outbreak risk.[60]

Specifically, this indicator displays how the core capacities influence the abundance and vectoral capacity of *Aedes aegypti* (Dengue) and of *Aedes albopictus* (Chikungunya and Zika) for each country and aggregated by WHO and World bank regions. The IHR core capacities data covers the period 2010-2018, so trends are presented for this period. Vulnerability is computed by taking the product of vectoral capacity (V) for Dengue, Chikungunya and Zika (including vector abundance), is normalised to range between 0 and 1, and dividing by the average of the 11 IHR core capacities. The formula below is used for the computation of vulnerability.

Vulnerability = Vectorial capacity / average IHR core capacity

The V, which express the average daily rate of subsequent cases in a susceptible population resulting from one infected case, was computed using the formula:[39]

$$V = ma^2 b_m p^n / -lnp$$

Here, a is the average vector biting rate, b_m is the probability of vector infection and transmission of virus to its saliva, n is the extrinsic incubation period while p is the daily survival probability. All these parameters are temperature dependent and are further described in Rocklöv et al. (2019).[39] m denotes the female mosquito to human population ratio and is estimated here as potential vector abundance for *Aedes aegypti* according to Liu-Helmersson et al. (2016)[40] and a modified *Aedes albopictus* abundance model based on Jia et al. (2016).[61] The V is estimated assuming human population are constant.

V depends only on vector biology and is intrinsically related to the basic reproduction number for vector-borne diseases, R_0, which is the expected number of hosts to be infected by a single infected host in a susceptible population and is formulated as:[39]

$$R_0 = Vb_h / r_h$$

Computation of V and abundance estimates was done at 0.5°x0.5° spatial resolution based on CRU TS v 4.0.3.[41] V (aegypti,chikungunya and zika) and vector abundance was run for both *aedes* aegypti and *aedes* albopictus vectors. The gridded outputs were subsequently aggregated to country level using shapefiles.

Data

1. Climate research unit (CRU) TS vs 4.0.3 precipitation and temperature data.[41]
2. IHR core capacities data, 2010-2018.[11]

Caveats

The abundance models generate predictions and not observed frequencies in relation to climate conditions, and by so should be considered a potential abundance estimate. The IHR data is self-reported by countries and may therefore include reporting bias which would affect this indicator. A reduction of this indicator while keeping the vector hazard constant does not correspond to full protection but indicates rather that the situation has improved by important improvements in core capacities.

Future form of the indicator

This indicator will be updated to capture a more comprehensive risk index, including further measurements of population vulnerability alongside adaptive capacity. It may also be updated to include vectorial capacity for the transmission of Yellow Fever, Chikungunya, and Zika. It will also strive to include more model estimates of vector to human densities (m), going beyond the presence / absence approach of vectors used here.

1.4: Food Security and Undernutrition

Indicator 1.4.1: Terrestrial Food Security and Undernutrition

Methods

Actual crop yields vary from year to year, not only with variations in weather, but also with changes in variety, farming practices and the occurrence of pest and disease. Crop yields, as estimated by crop models, are sensitive to the precise form of the crop model, and many models do not account for the short-term extremes that can significantly affect yields. The effect of year-to-year climatic variability on crop yields is therefore here represented by an agri-climatological proxy indicator, calculated from observed climate data and characterising potential variability in yield. Maize, rice, soybean and winter and spring wheat were selected as important traded and subsistence crops.

There are several potential proxies for variability from year to year in crop yield, including the number of hot days during critical periods in the growing season,[62-64] and the accumulated temperature between lower and upper thresholds over the growing season.[65] The proxy used here is based on crop duration, defined as the time taken in a year to accumulate the reference period (1981-2010) average growing season accumulated temperature total (ATT).[64] If the ATT is reached early, then the crop matures too quickly and yields are lower than average.[64,66] Here, the crop duration loss was defined as the difference in the time taken (in days) to accumulate the average growing season accumulated temperature.

The index is calculated across the area of land under cultivation[67] at 0.25° x 0.25°, and then area-weighted averaged. For the 2020 Lancet Countdown report, the index has been improved to capture different dates for crop planting and growing seasons ("harvest length") that varies by location.[68] Climate data is taken from the monthly historical records from ECMWF ERA5 climate reanalysis dataset between January 1980 and December 2019, and synthetic daily data is estimated for each grid cell by applying a regional average daily anomaly to the monthly value. The plot in the paper show the global average annual change in crop growth duration. The horizontal dashed line shows the average difference in crop growth duration over the reference period 1981-2010. Note that this is not zero because of the non-linear relationship between ATT and the time taken to accumulate a specific value of ATT.

Data

1. Climate data from the European Centre for Medium-Range Weather Forecasts (ECMWF) ERA5 reanalysis.[2]
2. Data for crop areas is taken from MIRCA2000.[67]
3. SAGE crop calendar is taken from Sacks et al.[68]

Caveats

Different ways of calculating the agri-climate index using different data sets would produce slightly different time series, as would the use of different agri-climate proxies. However, the broad patterns of variability over space and time are likely to be consistent across proxies and data sources.

The indicator does not take into account water shortage, and therefore characterises long-term change in yield potential rather than year to year variability.

Additional analysis

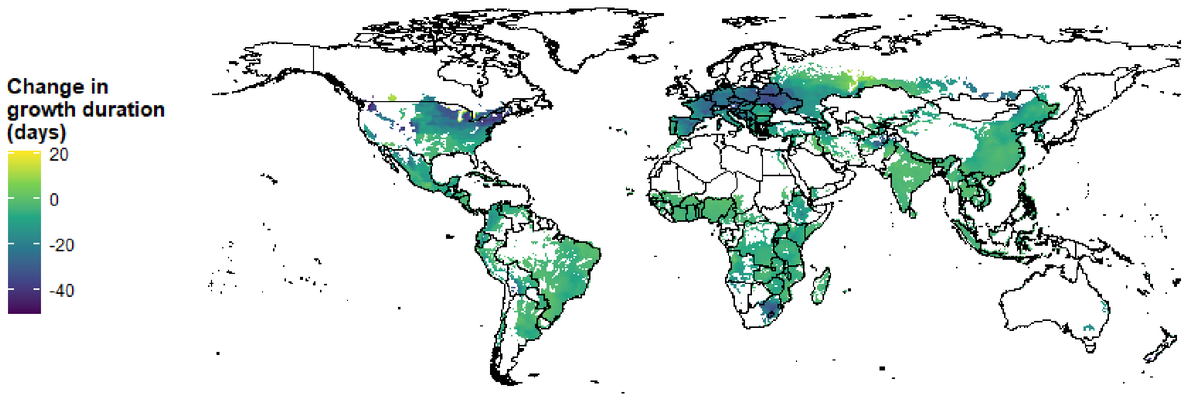

Figure 24: Change in maize growth duration in 2015-2019 compared with the 1981-2010 baseline.

Indicator 1.4.2: Marine Food Security and Undernutrition

Methods

A large proportion of the global population, especially in low-income and middle-income countries, are largely dependent on fish sources of protein.[69] Sea surface temperature rise is among the well accepted consequences of climate change.[70] The resultant thermal stress can sequentially impair marine fish capacity and capture including through bleaching of coral reefs. To compensate for the reduced marine fish production, countries have geared up farm-based fish production.

The methodology for this indicator applies to the same marine basins and countries as described in the 2019 Lancet Countdown report.[1] Sixteen FAO (Food and Agriculture Organization of the United Nations) fishing areas (3 areas located in Antarctica were excluded) which are important in terms of projected impacts and vulnerabilities associated with climate change were selected. Sixty-four countries located in these basins were chosen in order to attribute the impacts of climate change (more specifically Sea Surface Temperature SST) to the deterioration of major coral reef sites and the consequent decreased consumption of capture-based fish.

The indicator also compares the exposure to diet low in seafood ω3 in 195 countries between 1990 and 2017 and provides the relative risk of mortality from ischemic heart diseases by exposure to diet low in seafood ω3 (Figure 25).

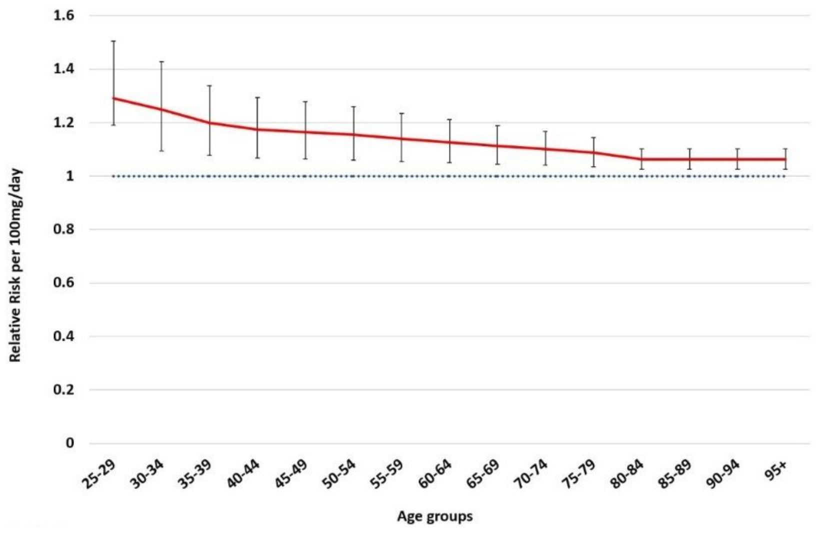

Figure 25: Relative risk (RR) of mortality for ischemic heart diseases by exposure to diet low in seafood ω3. Data taken from the 2017 Global Burden of Disease Study.[71]

Data

1. Data for SST was obtained from NASA Earth Observations (NEO) and covers the period from 2003 to 2019.[72]
2. The location of coral reef sites and data on annual maximum bleaching alert area caused by thermal stress was obtained from NOAA Coral Reef Watch Zones. Data is provided in five-year intervals from 1985 to 2019.[73]
3. Data on capture-based and farmed-based fish consumption per capita from 1980 to 2017 was collected from FAO.[74]

Caveats

There is a lack of information and data in the available databases such as FAO on fish species composition of the captured and farmed fish products. This could in turn lead to some concerns about the methodological approach used to calculate ω3 intake. More specifically, most of the approaches are based on fish intake, which usually ignore or underestimate variations in ω3 contents of different types of fishes, and especially capture-based compared with farmed-based fish.

The territorial waters of a number of countries investigated namely Canada, Nicaragua, Spain, Australia, and Indonesia, are located in more than one marine basin. Since fish capture data is reported based on countries and not marine basins, this could potentially introduce a level of uncertainty in the association between SST and fish capture.

Future form of the indicator

Future form of the indicator may combine spatiotemporal data on SST, capture, and types of captured and consumed fish species. This will allow to better estimate the exposure to a diet low in ω3 contents and its attributable health burden. Since the geographical coordinates for some of the data are not available, i.e., fish capture and health data, in the next step, the level of details on location will need to be defined. For instance, marine basin will be included in fish capture analysis as a variable.

Additional analysis

Figure 26 presents changes in sea surface temperature between the 2003-07 and the 2015-19 time periods for the 64 countries investigated from different basins.

Figure 27 reflects the increasing deterioration of annual maximum Bleaching Alert Area globally and threats to marine primary productivity being expected to follow.

Figure 28 shows that, between 1990 and 2017, diets low in seafood ω3 increased by 4.7% at global level with more than 70% of the countries experiencing an increase in exposure to this risk factor. This pattern may have increased the risk of mortality from ischemic heart diseases. Any factor that decreases quantity of seafoods or their ω3 contents, could increase the risk of ischemic heart diseases and other cardiovascular disorders which are already imposing a high burden to human populations.[37]

Figure 29 presents the trend of capture-based per capita fish consumption, a key source of ω3 fatty acids, as well as the corresponding trend for farm-based per capita fish consumption for the 64 countries considered in the indicator. The two trends are different: farm-based fish consumption has increased constantly during the last 4 decades whereas capture-based fish consumption has been decreasing since the mid-90s. It is worth noting, however, that the consumption trends are heterogeneous among different countries as shown in Figure 30.

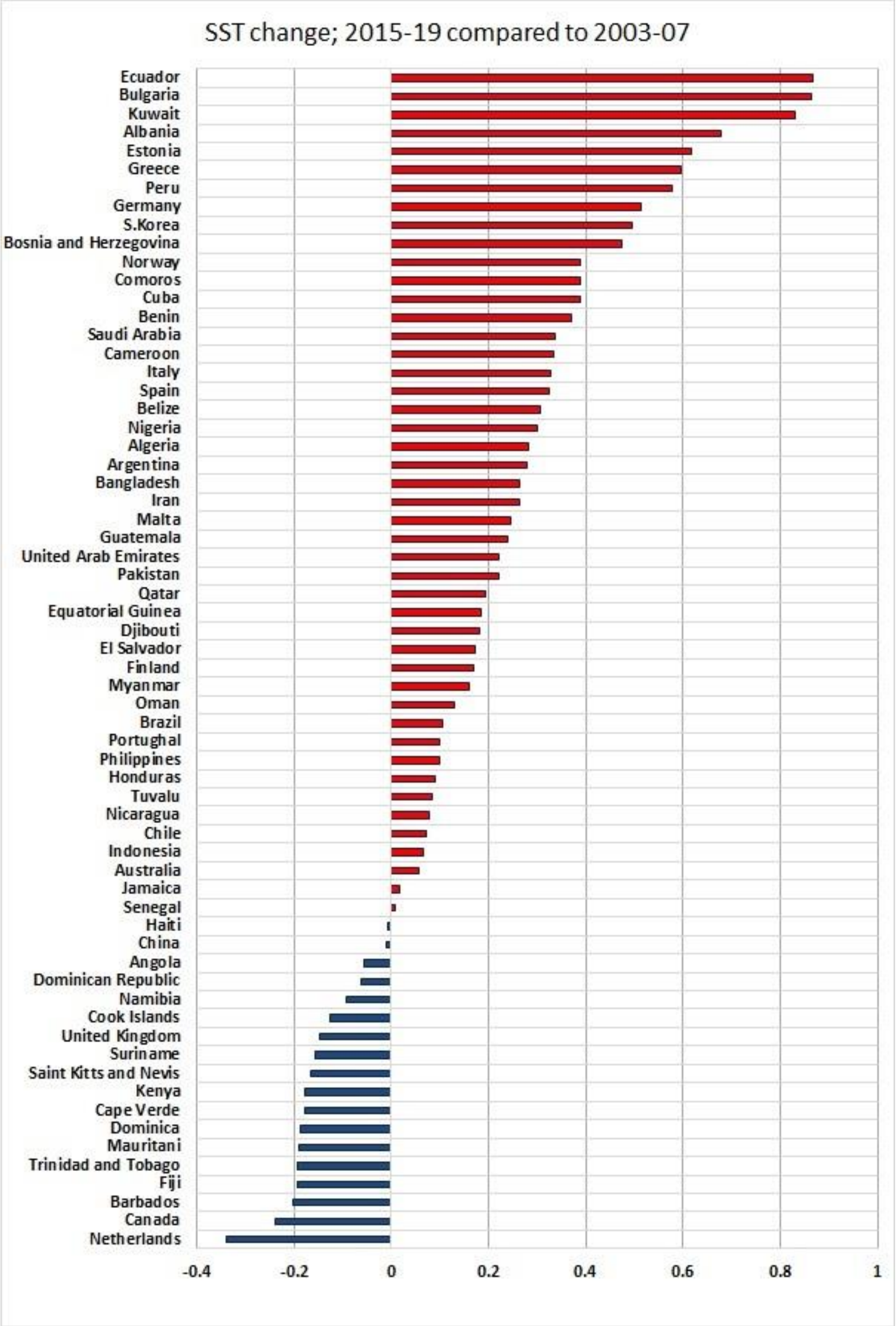

Figure 26: Changes in sea surface temperature (SST, °C) for the territorial waters of the 64 countries investigated located in different basins (FAO fishing areas): 2015-19 compared to 2003-07. Source: Sea Surface Temperature (MODIS), NASA Earth Observations (NEO); available at https://neo.sci.gsfc.nasa.gov/

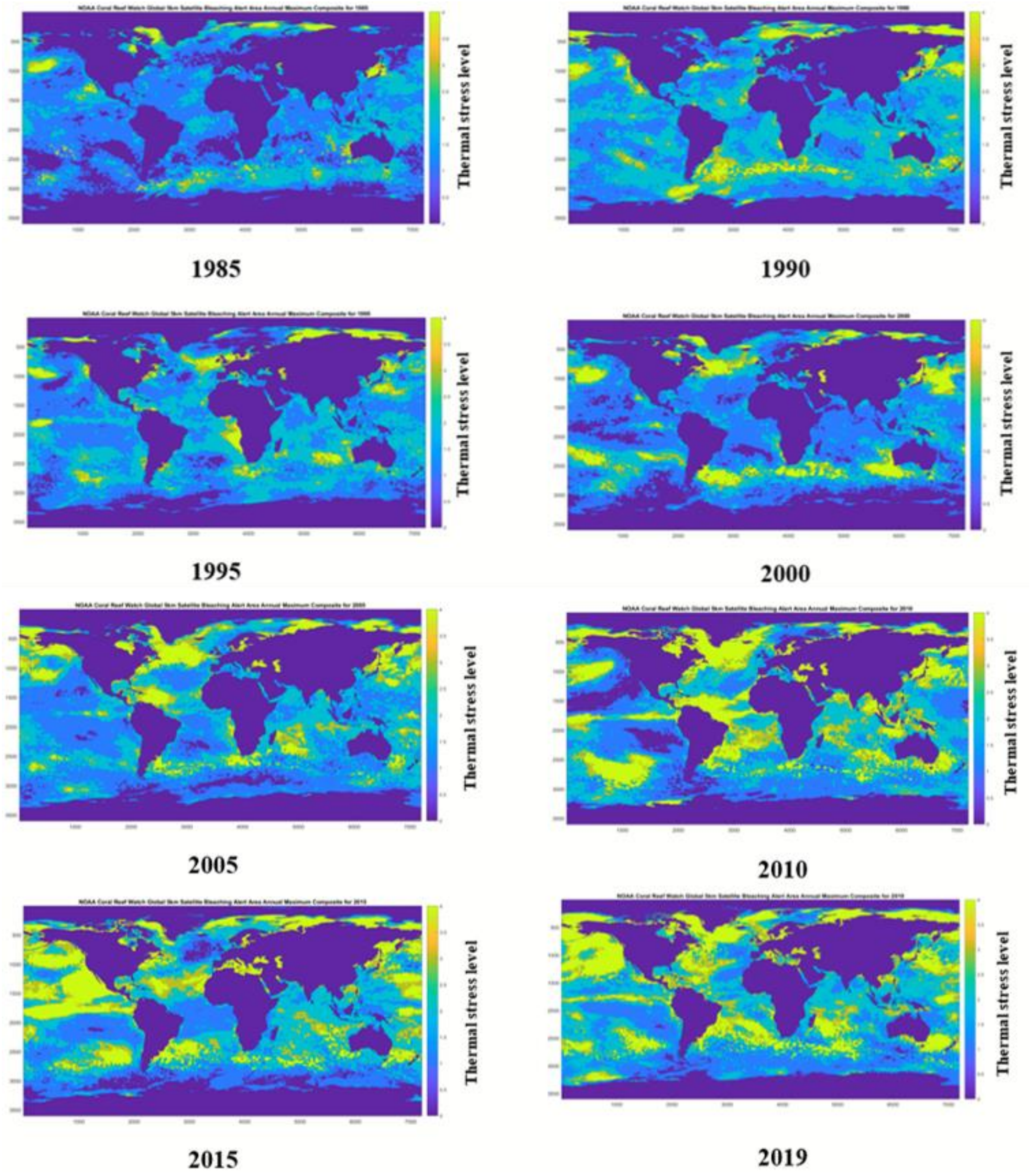

Figure 27: Comparing annual maximum Bleaching Alert Area caused by thermal stress in five-year intervals (1985-2019). Source: NOAA Coral Reef Watch. 2019, updated daily. NOAA Coral Reef Watch Version 3.1 Daily Global 5-km Satellite Coral Bleaching Degree Heating Week Product, Jun. 3, 2013-Jun. 2, 2014. College Park, Maryland, USA: NOAA Coral Reef Watch. Dataset accessed 2020-05-07 at https://coralreefwatch.noaa.gov/satellite/hdf/index.php.

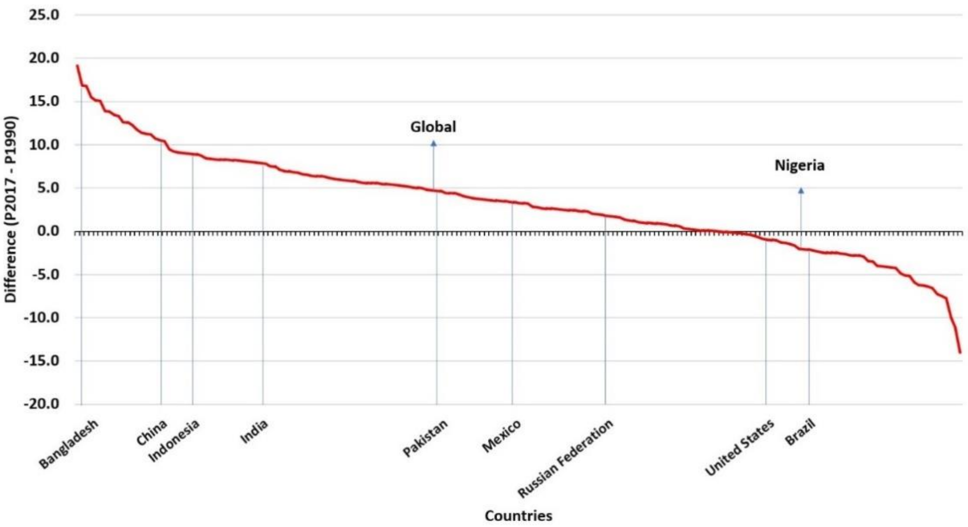

Figure 28: Difference in percentage of exposure to diet low in seafood ω3 in 2017 compared to the 1990 baseline in 195 countries (Countries have been sorted by the measure of difference on the horizontal axis and their names have been hidden except for the top 10 most populous countries).

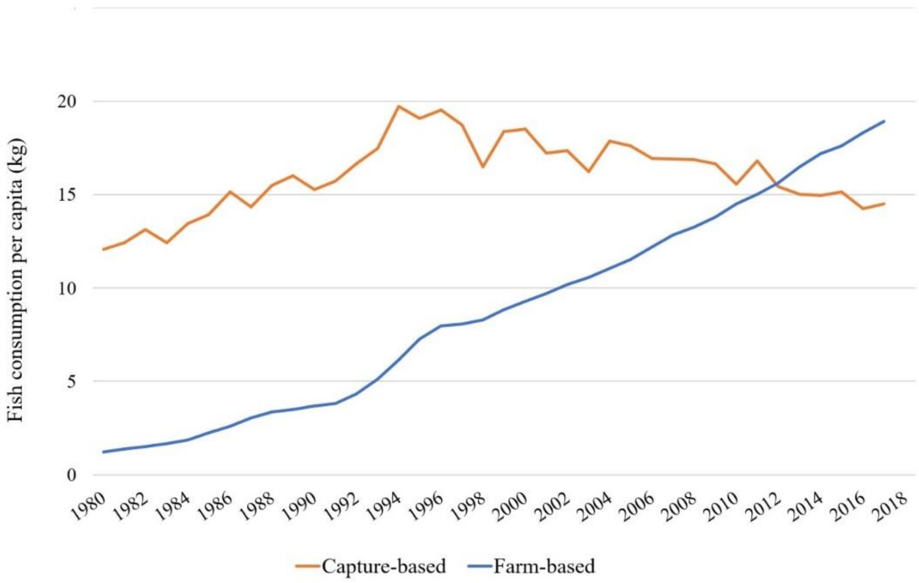

Figure 29: Population weighted average fish consumption per capita in 64 selected countries, separated by origin of fish (capture-based vs. farm-based).

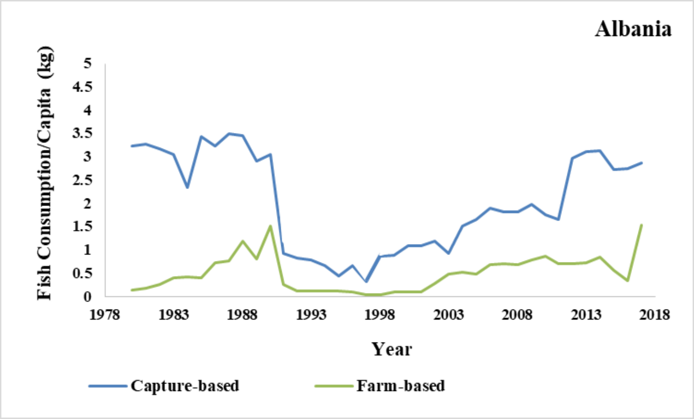
Albania
Fish Consumption/Capita (kg)
0 0.5 1 1.5 2 2.5 3 3.5 4 4.5 5
1978 1983 1988 1993 1998 2003 2008 2013 2018
Year
Capture-based
Farm-based

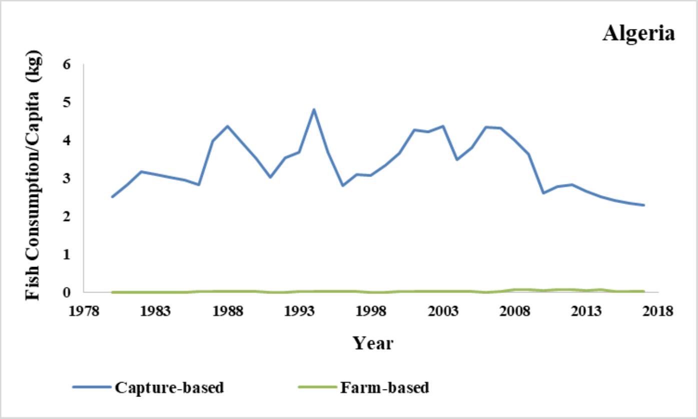
Algeria
Fish Consumption/Capita (kg)
0 1 2 3 4 5 6
1978 1983 1988 1993 1998 2003 2008 2013 2018
Year
Capture-based
Farm-based

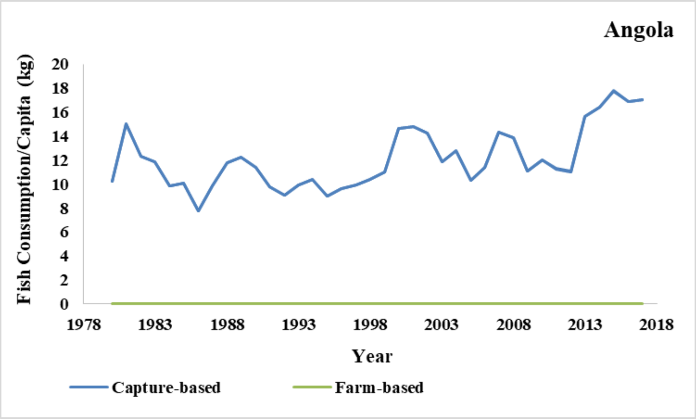
Angola
Fish Consumption/Capita (kg)
0 2 4 6 8 10 12 14 16 18 20
1978 1983 1988 1993 1998 2003 2008 2013 2018
Year
Capture-based
Farm-based

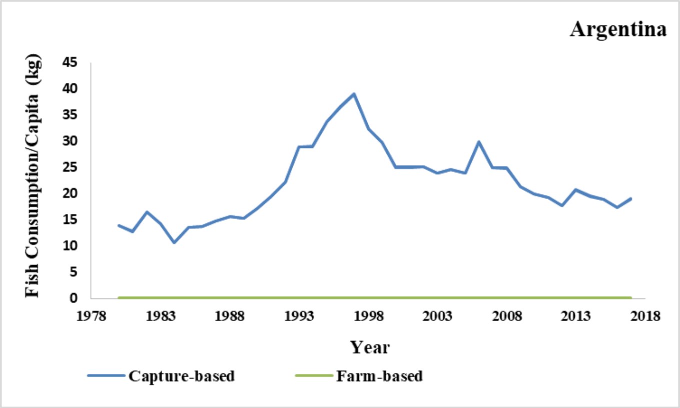
Argentina
Fish Consumption/Capita (kg)
0 5 10 15 20 25 30 35 40 45
1978 1983 1988 1993 1998 2003 2008 2013 2018
Year
Capture-based
Farm-based

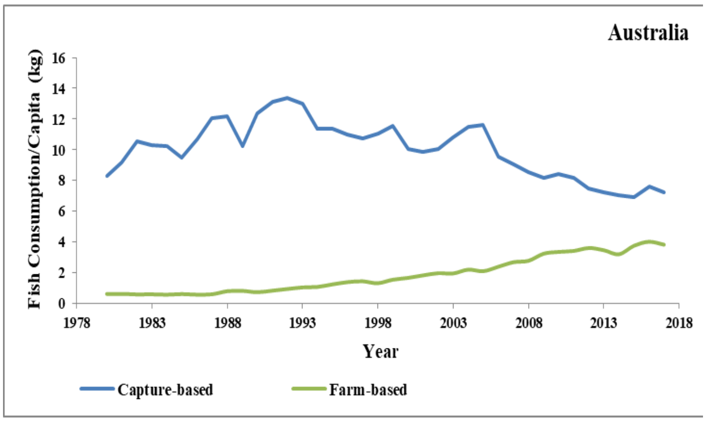
Australia
Fish Consumption/Capita (kg)
0 2 4 6 8 10 12 14 16
1978 1983 1988 1993 1998 2003 2008 2013 2018
Year
Capture-based
Farm-based

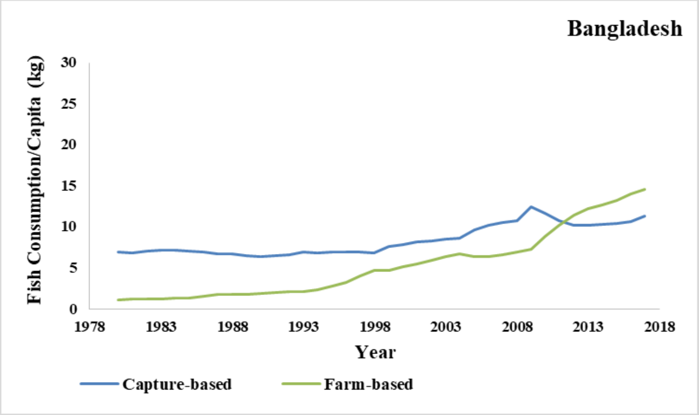
Bangladesh
Fish Consumption/Capita (kg)
0 5 10 15 20 25 30
1978 1983 1988 1993 1998 2003 2008 2013 2018
Year
Capture-based
Farm-based

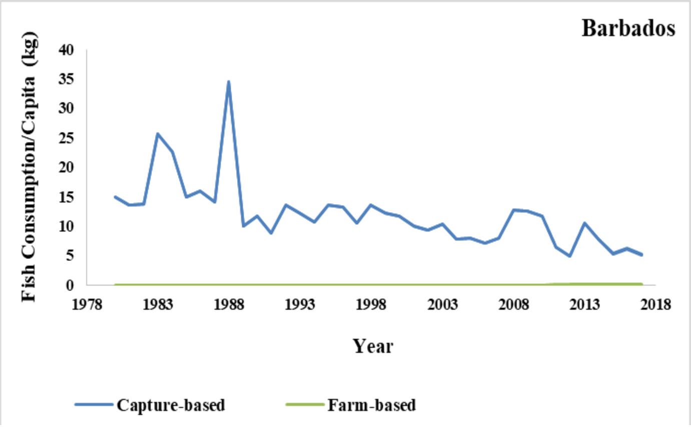
Barbados
Fish Consumption/Capita (kg)
0 5 10 15 20 25 30 35 40
1978 1983 1988 1993 1998 2003 2008 2013 2018
Year
Capture-based
Farm-based

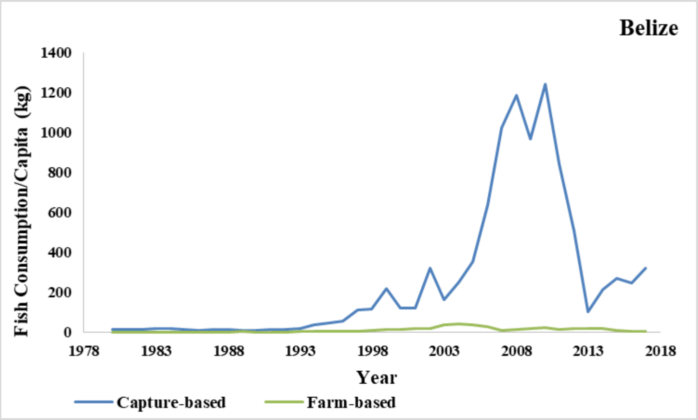
Belize
Fish Consumption/Capita (kg)
0 200 400 600 800 1000 1200 1400
1978 1983 1988 1993 1998 2003 2008 2013 2018
Year
Capture-based
Farm-based

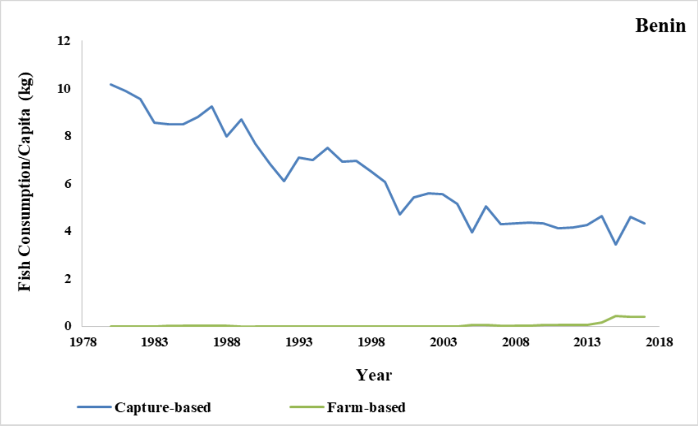
Benin
Fish Consumption/Capita (kg)
Year
Capture-based
Farm-based

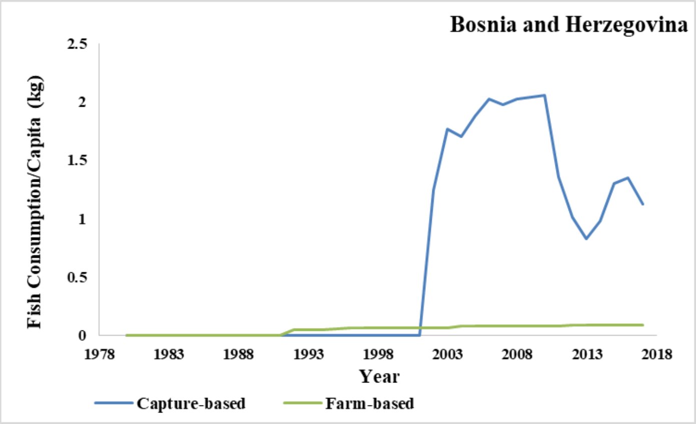
Bosnia and Herzegovina
Fish Consumption/Capita (kg)
Year
Capture-based
Farm-based

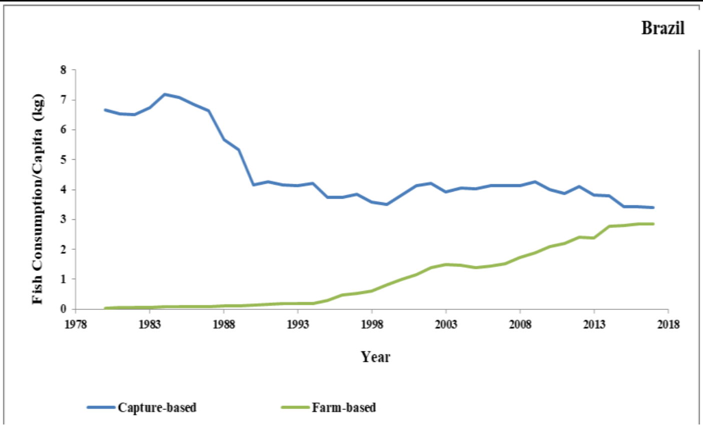
Brazil
Fish Consumption/Capita (kg)
Year
Capture-based
Farm-based

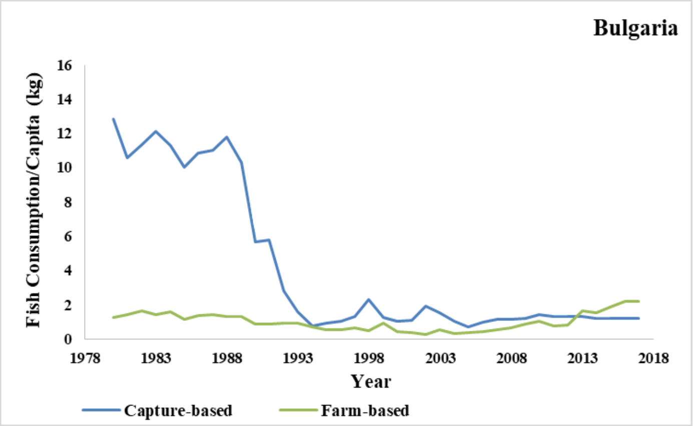
Bulgaria
Fish Consumption/Capita (kg)
Year
Capture-based
Farm-based

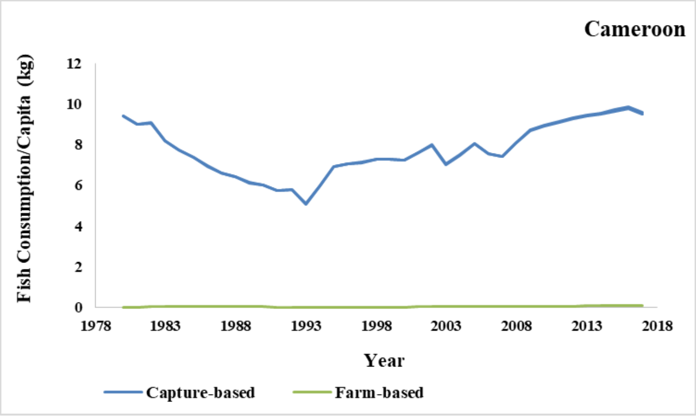
Cameroon
Fish Consumption/Capita (kg)
Year
Capture-based
Farm-based

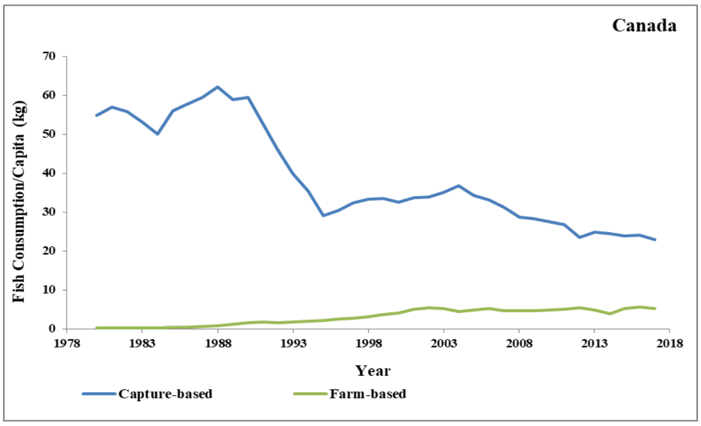
Canada
Fish Consumption/Capita (kg)
Year
Capture-based
Farm-based

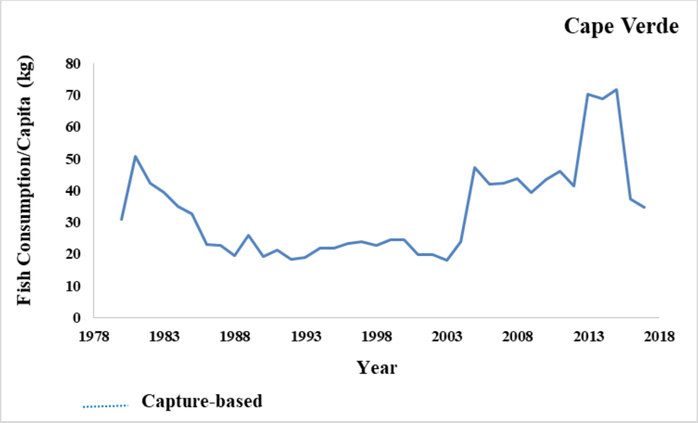
Cape Verde
Fish Consumption/Capita (kg)
Year
Capture-based

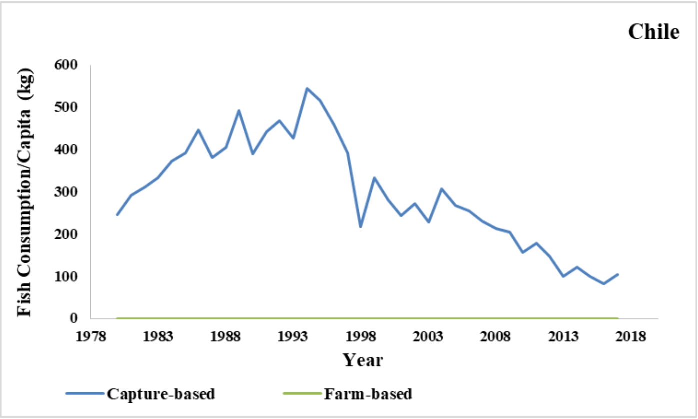
Chile
Fish Consumption/Capita (kg)
Year
Capture-based
Farm-based

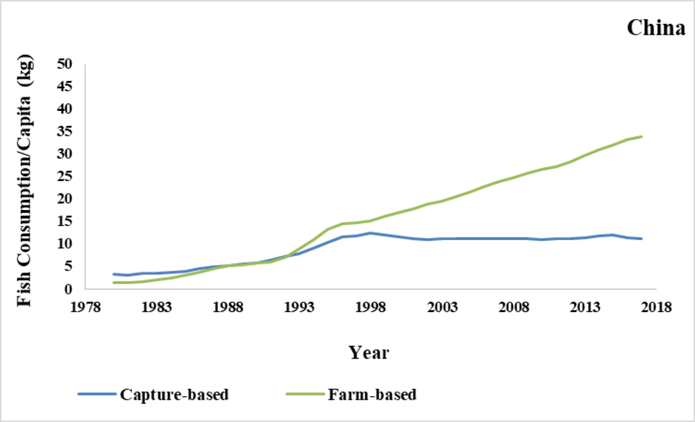
China
Fish Consumption/Capita (kg)
50
45
40
35
30
25
20
15
10
5
0
1978
1983
1988
1993
1998
2003
2008
2013
2018
Year
Capture-based
Farm-based

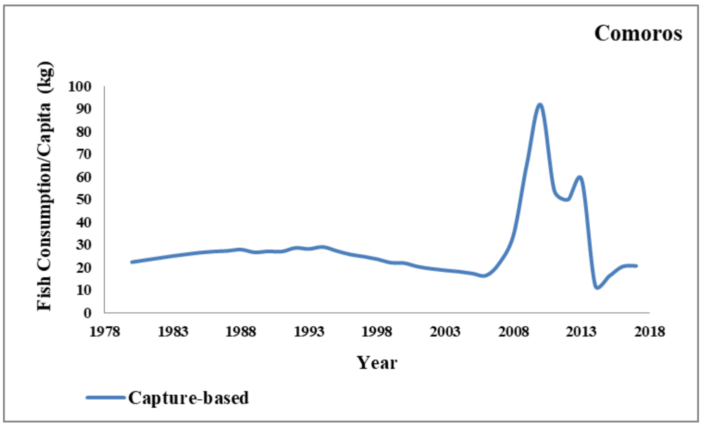
Comoros
Fish Consumption/Capita (kg)
100
90
80
70
60
50
40
30
20
10
0
1978
1983
1988
1993
1998
2003
2008
2013
2018
Year
Capture-based

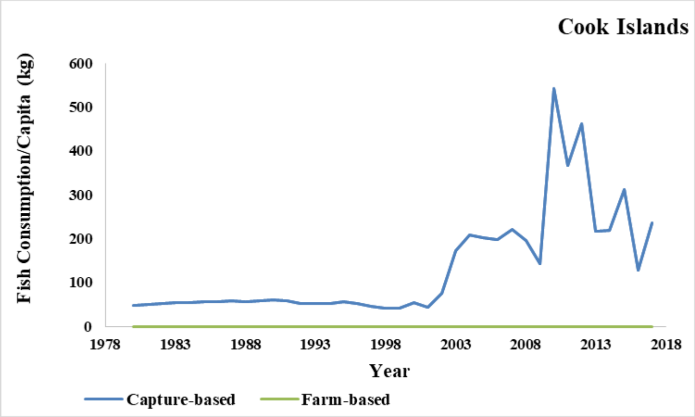
Cook Islands
Fish Consumption/Capita (kg)
600
500
400
300
200
100
0
1978
1983
1988
1993
1998
2003
2008
2013
2018
Year
Capture-based
Farm-based

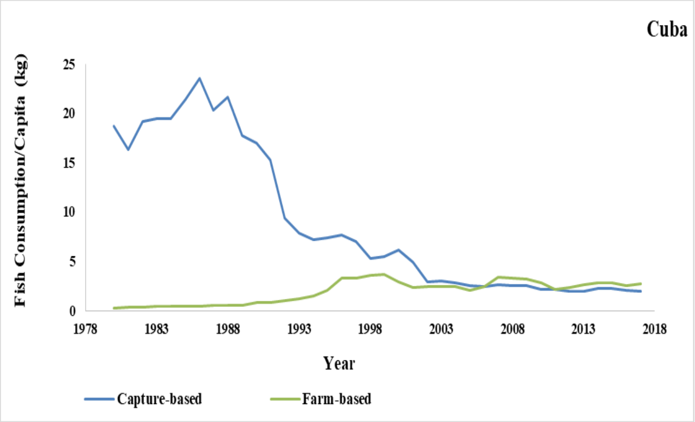
Cuba
Fish Consumption/Capita (kg)
25
20
15
10
5
0
1978
1983
1988
1993
1998
2003
2008
2013
2018
Year
Capture-based
Farm-based

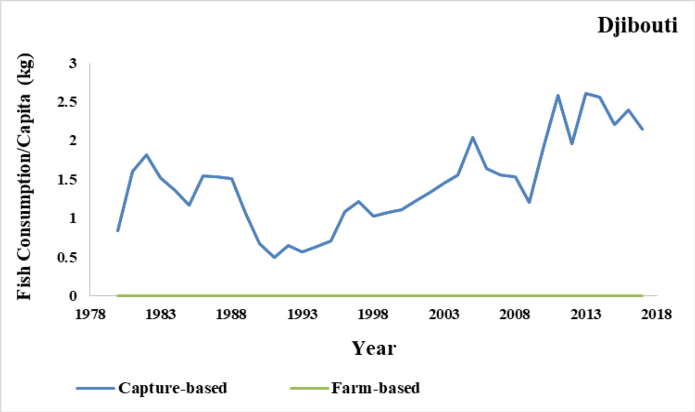
Djibouti
Fish Consumption/Capita (kg)
3
2.5
2
1.5
1
0.5
0
1978
1983
1988
1993
1998
2003
2008
2013
2018
Year
Capture-based
Farm-based

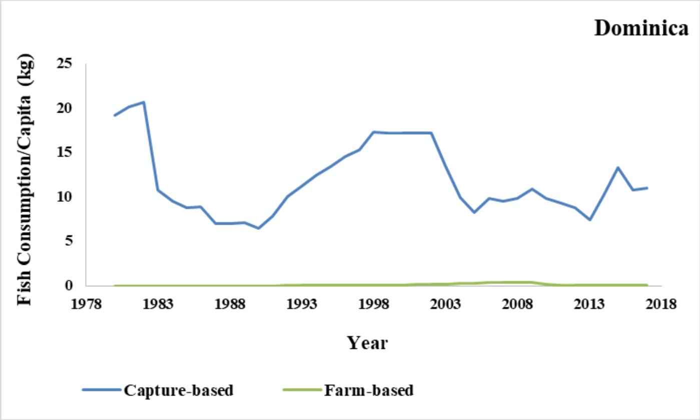
Dominica
Fish Consumption/Capita (kg)
25
20
15
10
5
0
1978
1983
1988
1993
1998
2003
2008
2013
2018
Year
Capture-based
Farm-based

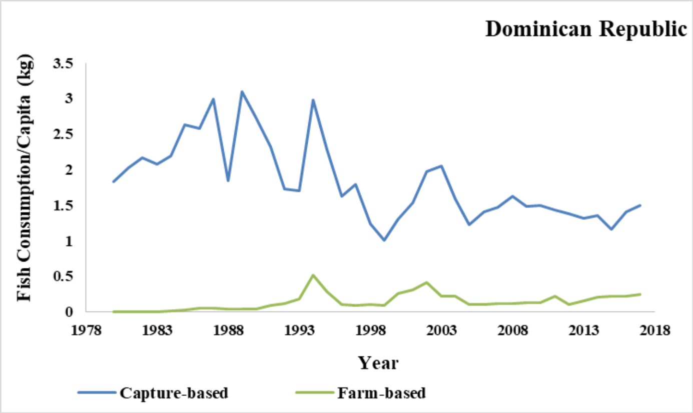
Dominican Republic
Fish Consumption/Capita (kg)
3.5
3
2.5
2
1.5
1
0.5
0
1978
1983
1988
1993
1998
2003
2008
2013
2018
Year
Capture-based
Farm-based

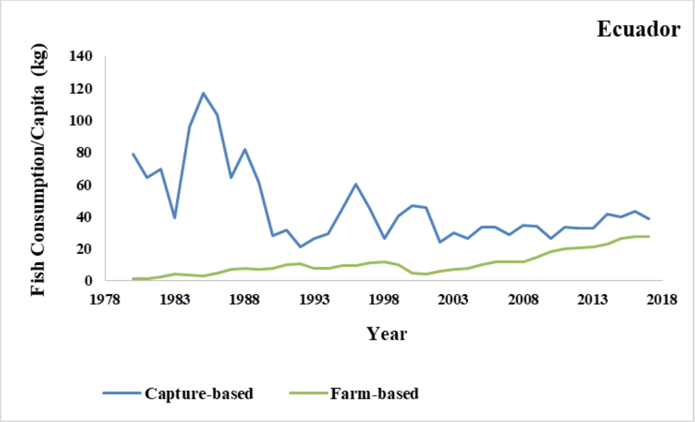
Ecuador
Fish Consumption/Capita (kg)
140
120
100
80
60
40
20
0
1978
1983
1988
1993
1998
2003
2008
2013
2018
Year
Capture-based
Farm-based

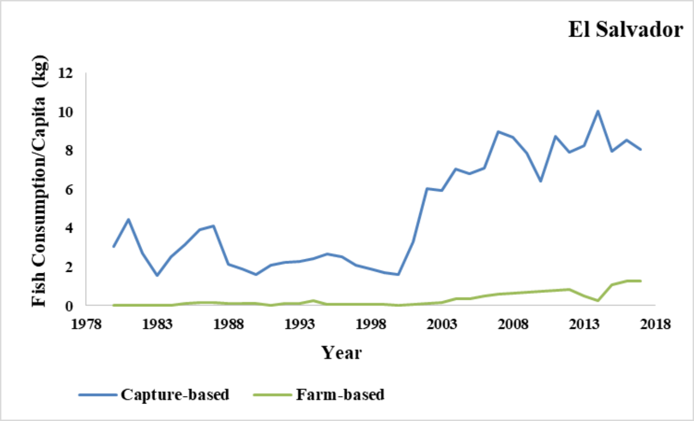
El Salvador
Fish Consumption/Capita (kg)
0
2
4
6
8
10
12
1978
1983
1988
1993
1998
2003
2008
2013
2018
Year
Capture-based
Farm-based

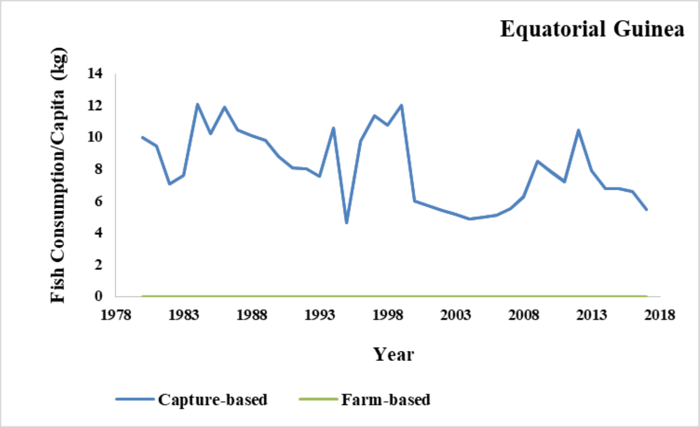
Equatorial Guinea
Fish Consumption/Capita (kg)
0
2
4
6
8
10
12
14
1978
1983
1988
1993
1998
2003
2008
2013
2018
Year
Capture-based
Farm-based

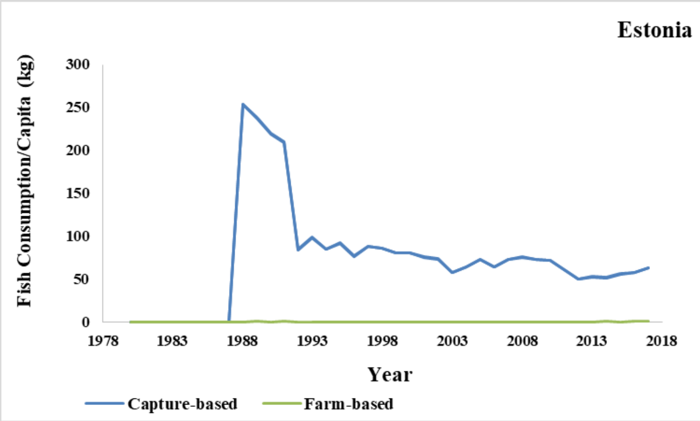
Estonia
Fish Consumption/Capita (kg)
0
50
100
150
200
250
300
1978
1983
1988
1993
1998
2003
2008
2013
2018
Year
Capture-based
Farm-based

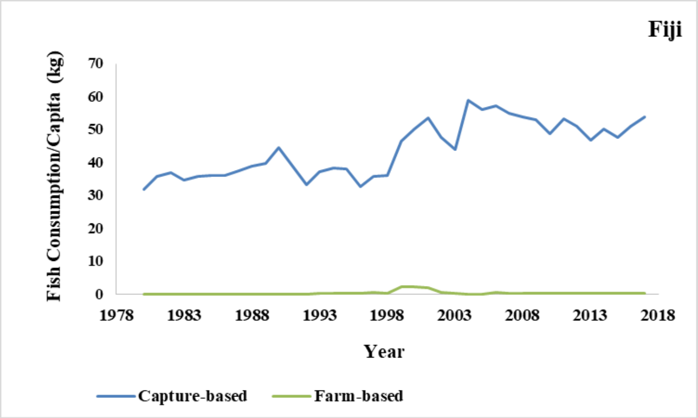
Fiji
Fish Consumption/Capita (kg)
0
10
20
30
40
50
60
70
1978
1983
1988
1993
1998
2003
2008
2013
2018
Year
Capture-based
Farm-based

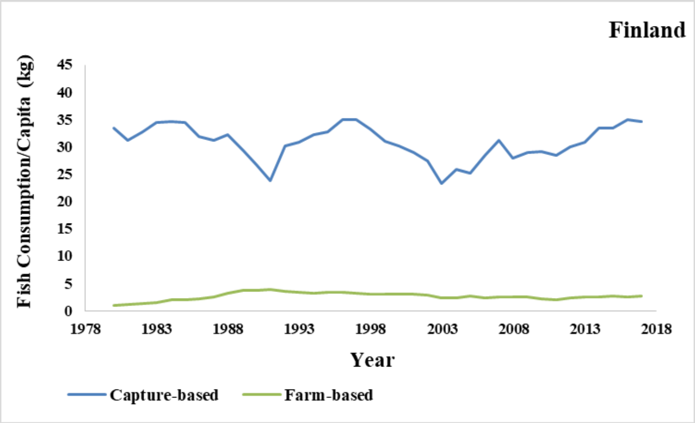
Finland
Fish Consumption/Capita (kg)
0
5
10
15
20
25
30
35
40
45
1978
1983
1988
1993
1998
2003
2008
2013
2018
Year
Capture-based
Farm-based

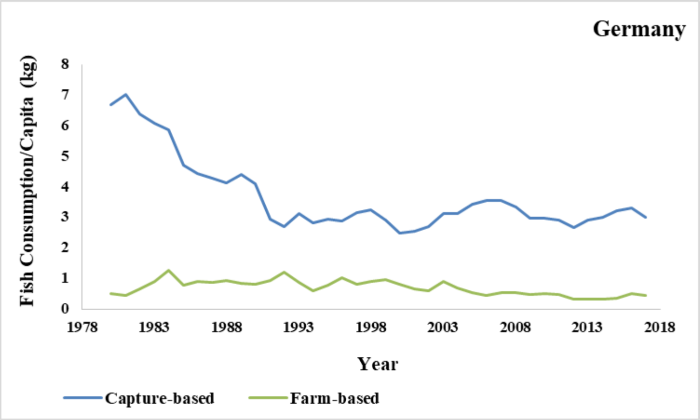
Germany
Fish Consumption/Capita (kg)
0
1
2
3
4
5
6
7
8
1978
1983
1988
1993
1998
2003
2008
2013
2018
Year
Capture-based
Farm-based

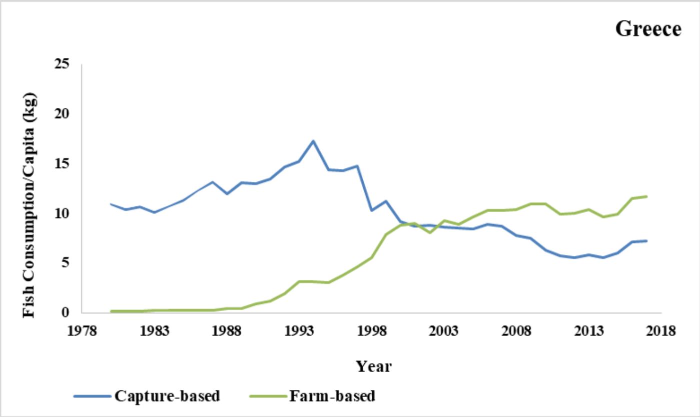
Greece
Fish Consumption/Capita (kg)
0
5
10
15
20
25
1978
1983
1988
1993
1998
2003
2008
2013
2018
Year
Capture-based
Farm-based

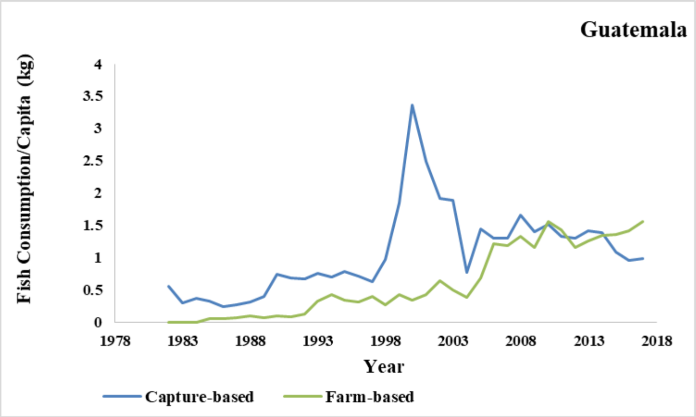
Guatemala
Fish Consumption/Capita (kg)
0
0.5
1
1.5
2
2.5
3
3.5
4
1978
1983
1988
1993
1998
2003
2008
2013
2018
Year
Capture-based
Farm-based

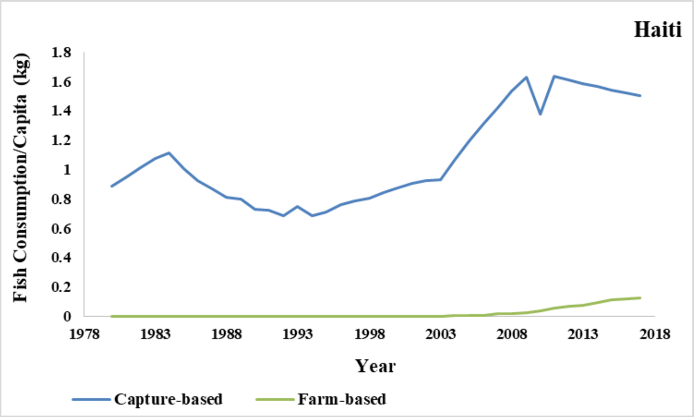
Haiti
Fish Consumption/Capita (kg)
1.8
1.6
1.4
1.2
1
0.8
0.6
0.4
0.2
0
1978
1983
1988
1993
1998
2003
2008
2013
2018
Year
Capture-based
Farm-based

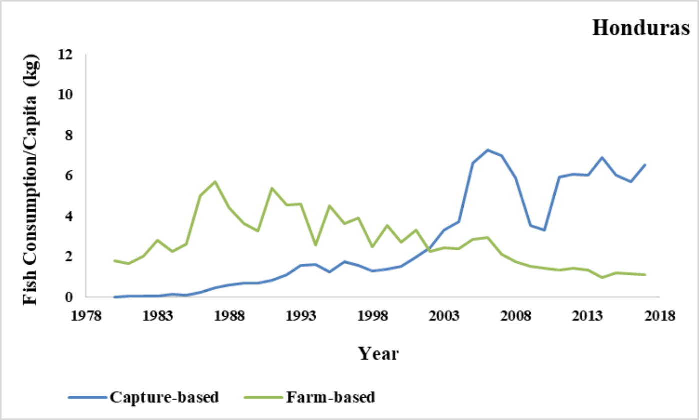
Honduras
Fish Consumption/Capita (kg)
12
10
8
6
4
2
0
1978
1983
1988
1993
1998
2003
2008
2013
2018
Year
Capture-based
Farm-based

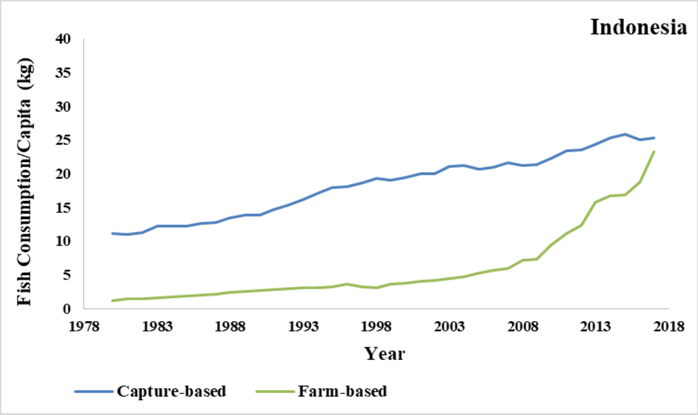
Indonesia
Fish Consumption/Capita (kg)
40
35
30
25
20
15
10
5
0
1978
1983
1988
1993
1998
2003
2008
2013
2018
Year
Capture-based
Farm-based

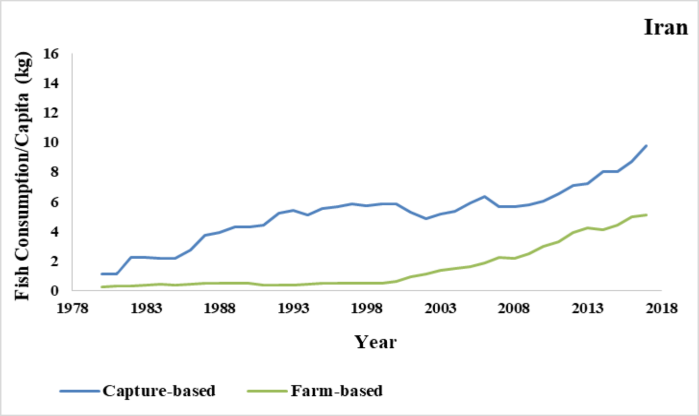
Iran
Fish Consumption/Capita (kg)
16
14
12
10
8
6
4
2
0
1978
1983
1988
1993
1998
2003
2008
2013
2018
Year
Capture-based
Farm-based

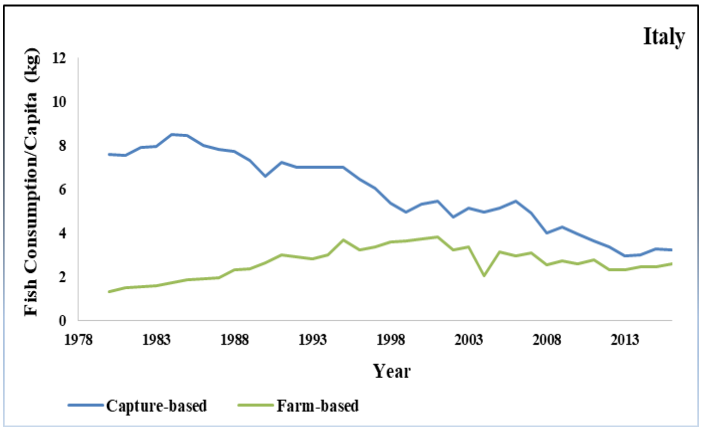
Italy
Fish Consumption/Capita (kg)
12
10
8
6
4
2
0
1978
1983
1988
1993
1998
2003
2008
2013
Year
Capture-based
Farm-based

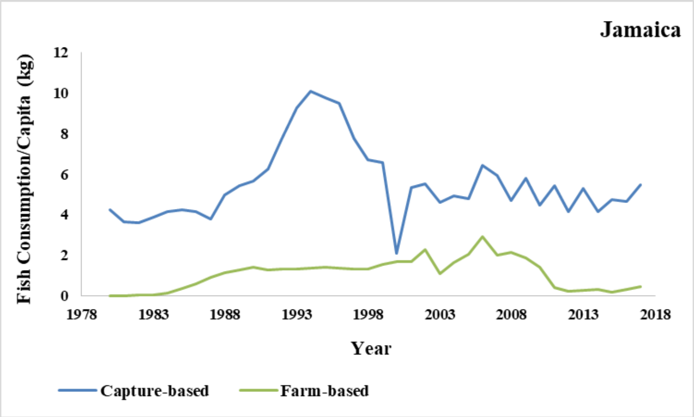
Jamaica
Fish Consumption/Capita (kg)
12
10
8
6
4
2
0
1978
1983
1988
1993
1998
2003
2008
2013
2018
Year
Capture-based
Farm-based

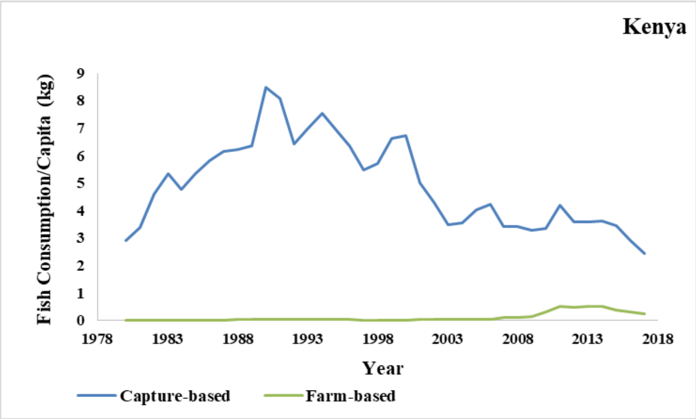
Kenya
Fish Consumption/Capita (kg)
9
8
7
6
5
4
3
2
1
0
1978
1983
1988
1993
1998
2003
2008
2013
2018
Year
Capture-based
Farm-based

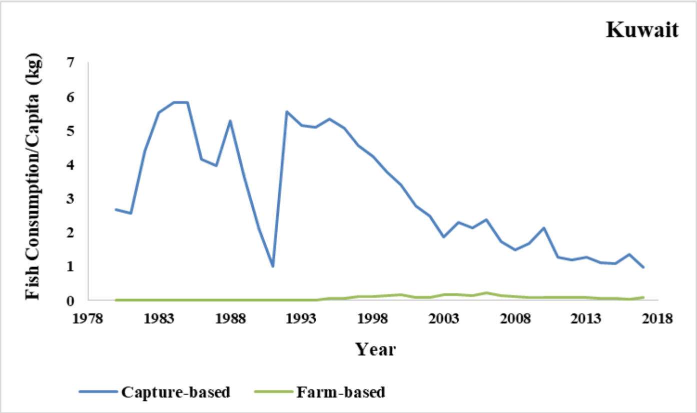
Kuwait
Fish Consumption/Capita (kg)
7
6
5
4
3
2
1
0
1978
1983
1988
1993
1998
2003
2008
2013
2018
Year
Capture-based
Farm-based

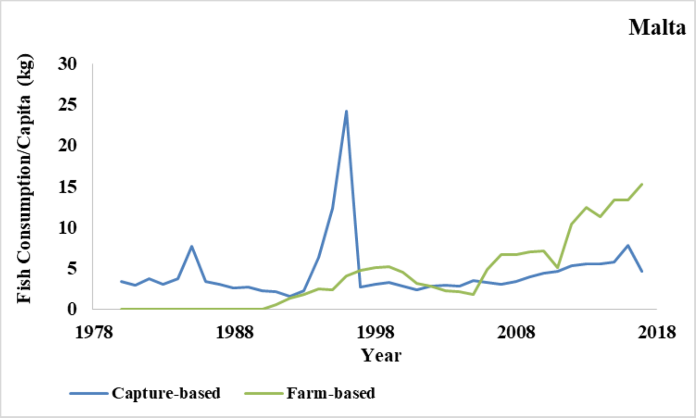
Malta
Fish Consumption/Capita (kg)
0 5 10 15 20 25 30
1978 1988 1998 2008 2018
Year
Capture-based
Farm-based

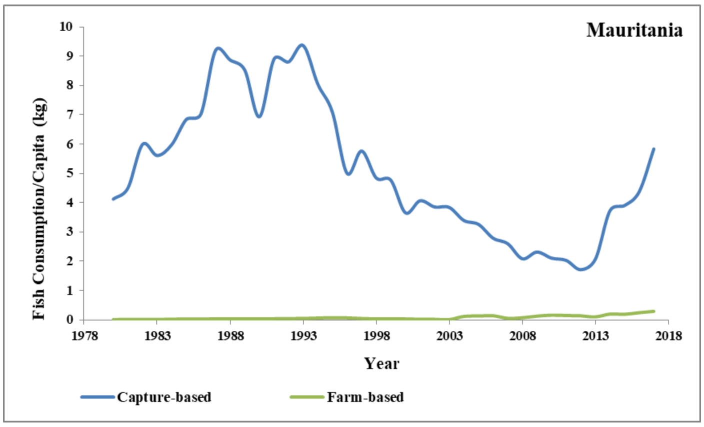
Mauritania
Fish Consumption/Capita (kg)
0 1 2 3 4 5 6 7 8 9 10
1978 1983 1988 1993 1998 2003 2008 2013 2018
Year
Capture-based
Farm-based

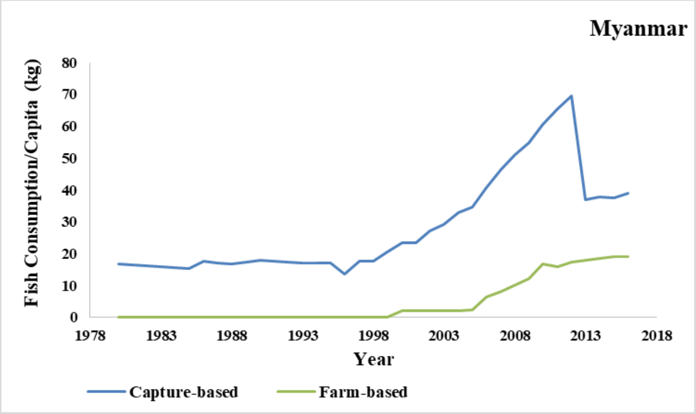
Myanmar
Fish Consumption/Capita (kg)
0 10 20 30 40 50 60 70 80
1978 1983 1988 1993 1998 2003 2008 2013 2018
Year
Capture-based
Farm-based

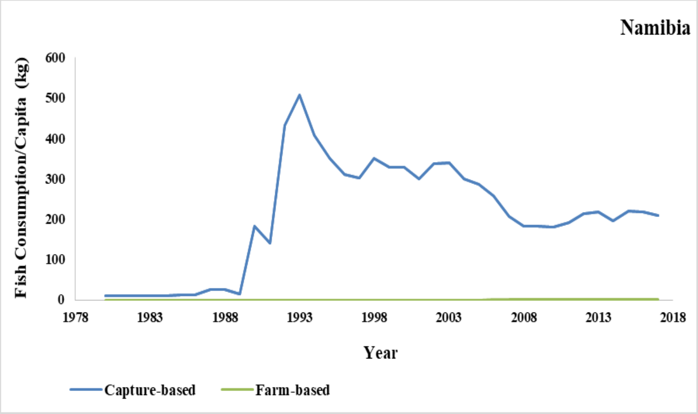
Namibia
Fish Consumption/Capita (kg)
0 100 200 300 400 500 600
1978 1983 1988 1993 1998 2003 2008 2013 2018
Year
Capture-based
Farm-based

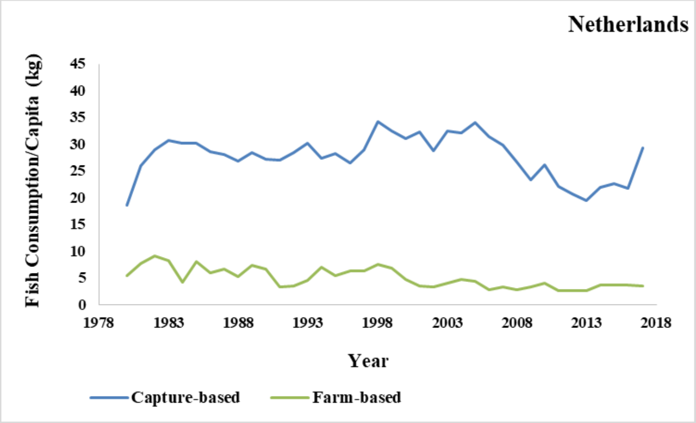
Netherlands
Fish Consumption/Capita (kg)
0 5 10 15 20 25 30 35 40 45
1978 1983 1988 1993 1998 2003 2008 2013 2018
Year
Capture-based
Farm-based

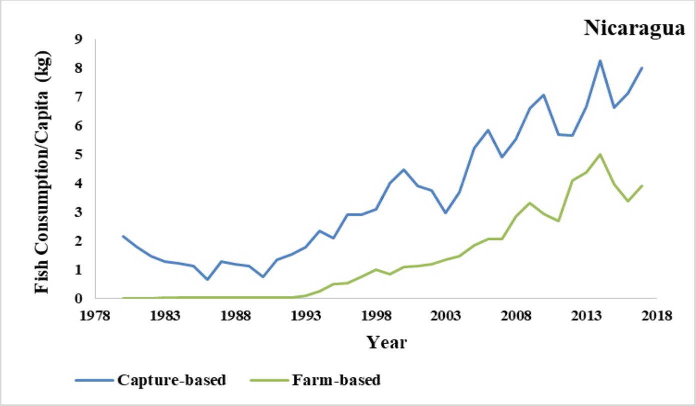
Nicaragua
Fish Consumption/Capita (kg)
0 1 2 3 4 5 6 7 8 9
1978 1983 1988 1993 1998 2003 2008 2013 2018
Year
Capture-based
Farm-based

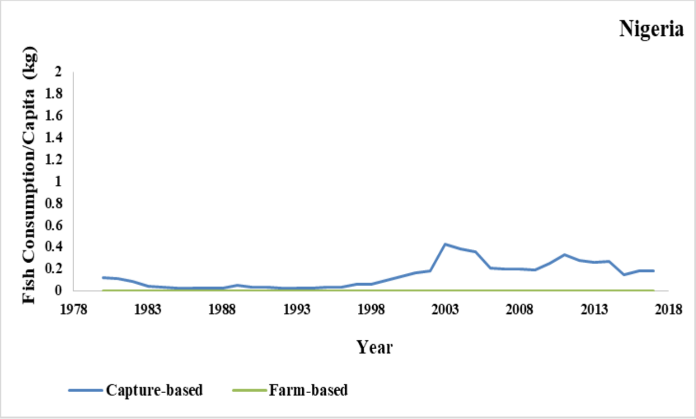
Nigeria
Fish Consumption/Capita (kg)
0 0.2 0.4 0.6 0.8 1 1.2 1.4 1.6 1.8 2
1978 1983 1988 1993 1998 2003 2008 2013 2018
Year
Capture-based
Farm-based

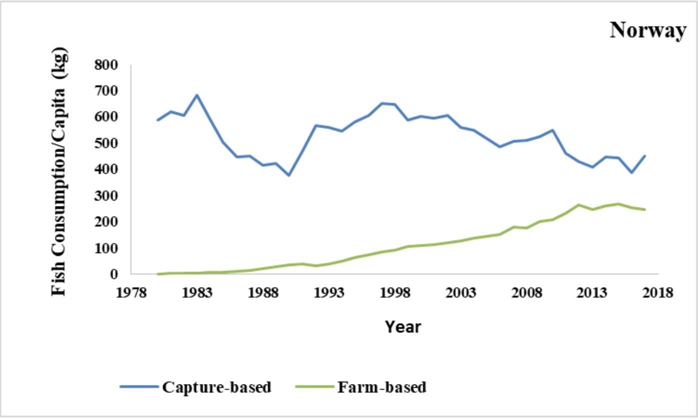
Norway
Fish Consumption/Capita (kg)
0 100 200 300 400 500 600 700 800
1978 1983 1988 1993 1998 2003 2008 2013 2018
Year
Capture-based
Farm-based

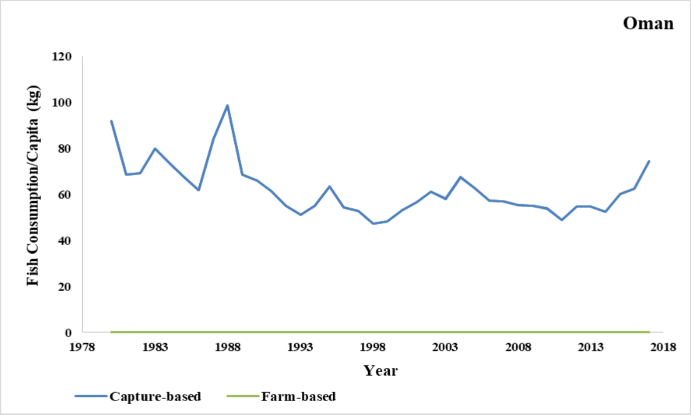
Oman
Fish Consumption/Capita (kg)
Year
Capture-based
Farm-based

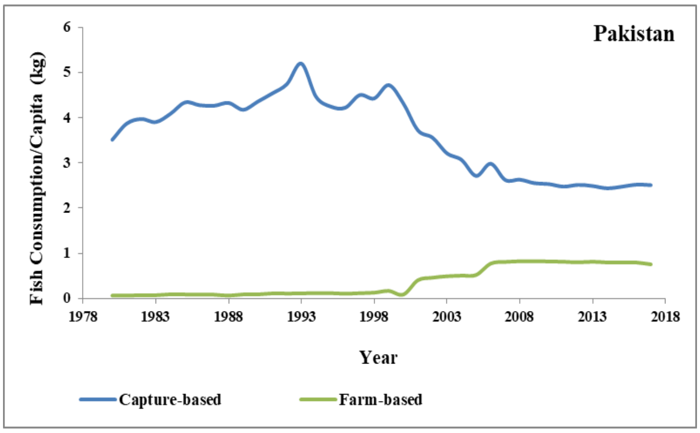
Pakistan
Fish Consumption/Capita (kg)
Year
Capture-based
Farm-based

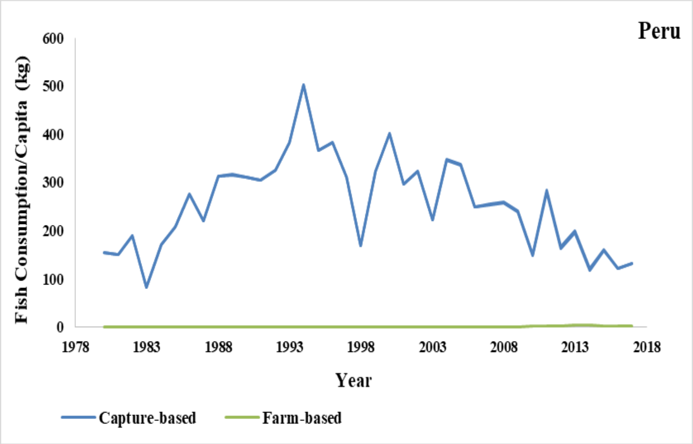
Peru
Fish Consumption/Capita (kg)
Year
Capture-based
Farm-based

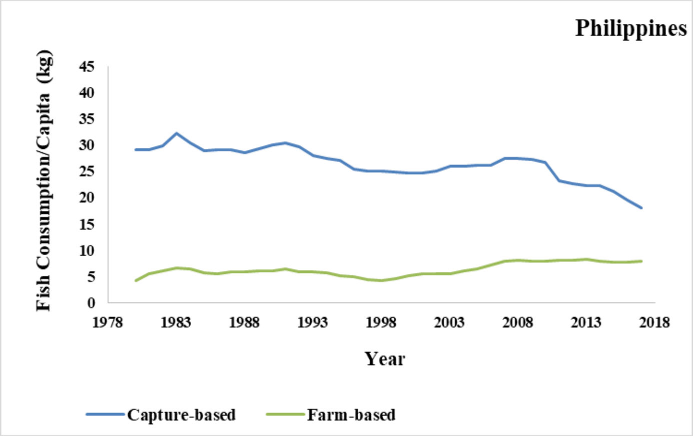
Philippines
Fish Consumption/Capita (kg)
Year
Capture-based
Farm-based

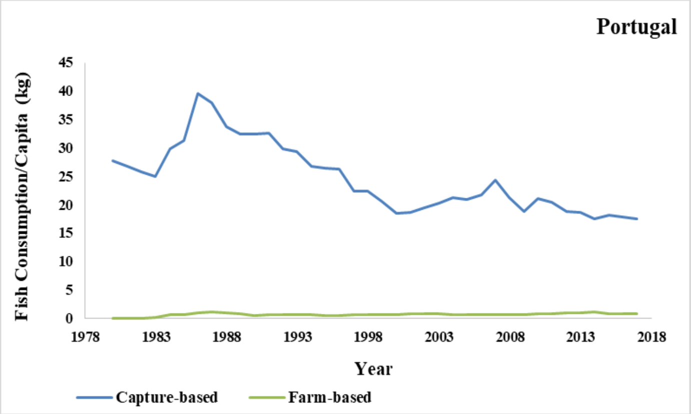
Portugal
Fish Consumption/Capita (kg)
Year
Capture-based
Farm-based

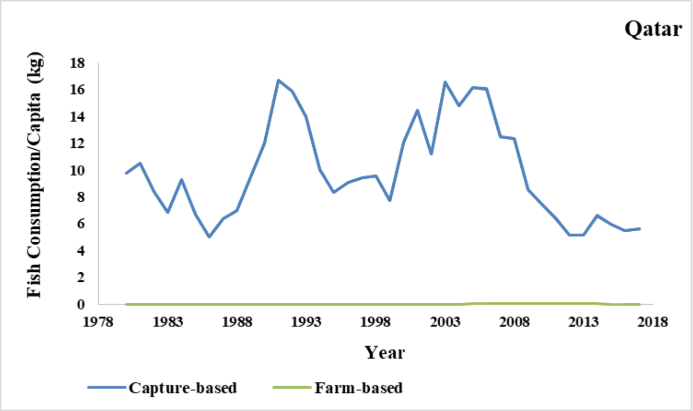
Qatar
Fish Consumption/Capita (kg)
Year
Capture-based
Farm-based

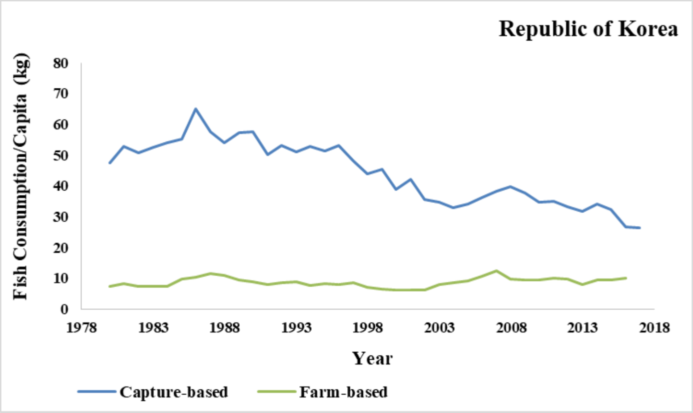
Republic of Korea
Fish Consumption/Capita (kg)
Year
Capture-based
Farm-based

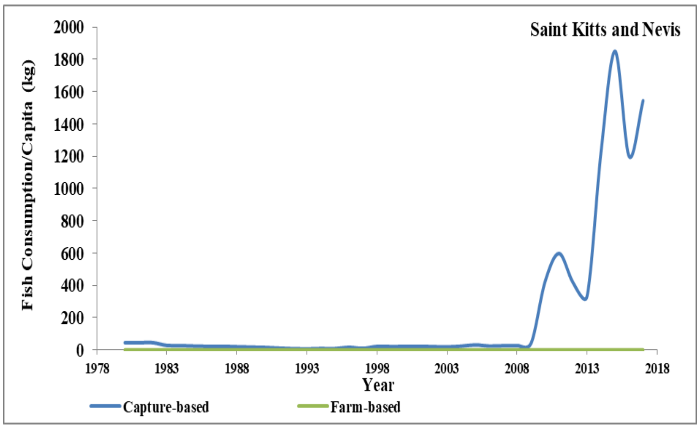
Saint Kitts and Nevis
Fish Consumption/Capita (kg)
Year
Capture-based
Farm-based

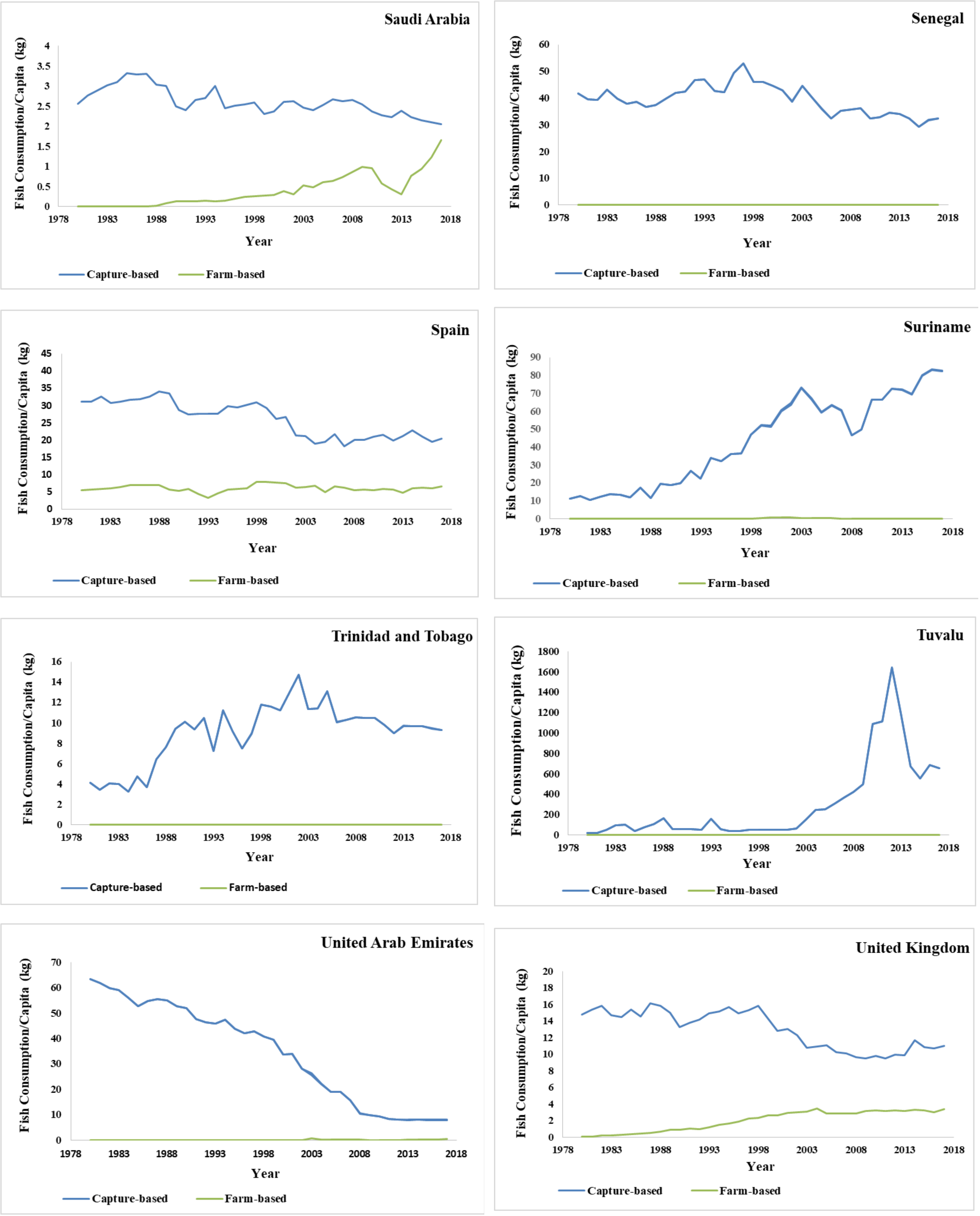

Figure 30: Trends of capture-based and farmed-based per capita fish consumption in the 64 countries investigated over the period of 1980-2017.

Indicator 1.5: Migration, Displacement and Sea-Level Rise

Population exposure to global mean sea level rise

Methods

This is the first year this indicator has been included in a Lancet Countdown report. It represents a direct measure of population exposure to Global Mean Sea Level Rise (GMSLR). By using a bathtub model, this indicator overlays future GMSLR (of 1m and 5m) with coastal elevation value grid-cells to delineate areas of potential inundation and current global population distribution grid-cells to delineate populations living in areas that will be exposed to absolute global mean sea level rise.

In the first step, the Coastal Digital Elevation Model (CoastalDEM) dataset was used to categorise inundated grid-cells under two scenarios of global mean sea level rise (GMSLR): i.e. 1m and 5m of GMSLR. In the second step, the gridded population dataset (LandScan) was overlaid on the grid-cells identified in step one to estimate population exposure values. These grid-cells are then matched with country boundaries using Global Administrative Areas (GADM) 3.6 Data Set. Finally, grid-cell level data was aggregated to country level (population numbers exposed to GMSLR; proportion of population exposed to GMSLR).

Data

1. GMSLR data was taken from estimated global mean increases in sea-levels.[75]
2. Elevation data was taken from Coastal Digital Elevation Model (CoastalDEM).[76]
3. Population distribution was taken from LandScan.[77]
4. Global Administrative Areas (GADM) version 3.6.[78]

Caveats

Between 1902 and 2015, the global mean sea level increased by 0.12–0.21m.[79] Relative to 1986–2005, additional GMSLR of 0.43-0.84m is projected by 2100 (0.29-1.10m, likely range),[79] although it depends particularly on the rate of Greenland and Antarctic ice sheet melting.[80]

Estimates of population exposure to GMSLR vary according to the input datasets, timeframes and geographic scales, the parameters that are set for about emissions and socioeconomic scenarios, and methods of analysis;[81-83] results should be regarded within the context of the methods and datasets used. As such underlying errors and uncertainties in the input datasets (GMSLR, elevation, and population) are limitations of this analysis.

The datasets employed for this indicator are global, reputable and widely used in analyses of exposure to SLR. CoastalDEM (3-arc second; 90m) is a new global coastal digital elevation model that has been adjusted to reduce SRTM error.[84] LandScan disaggregates census data within administrative boundaries based on weightings derived from land cover data, proximity to roads, slope, and populated areas.[83]

Population exposure to SLR is not a proxy indicator for population displacement due to SLR. First, migration decisions are shaped by more than environmental factors.[85,86] Second, SLR could generate ‘trapped’ populations, people who desire to move away but do not have the necessary resources to escape sites of risk,[87,88] and some will prefer to stay put for social, cultural and political reasons including attachment to place. Third, other climate impacts and demographic factors contribute migration into low-lying coastal sites.[81,89-91] Fourth, relocation and migration can be prevented or forestalled through other adaptive strategies. Nonetheless, human mobility/migration is an emerging and likely response in the absence of other adaptation pathways.

For populations exposed to SLR, corresponding health outcomes result from the direct and indirect effects of sea level change and adaptive opportunities and constraints, including migration as a potential adaptive response.[92,93] For those who reside or are ‘trapped’ in low-lying vulnerable sites, SLR-related health impacts may emerge from changes in water and food security, disease ecology, flooding and saltwater intrusion, and the psychosocial impacts of disrupted livelihoods.[86,94] Several case studies identify health risks and opportunities of

managed retreat among populations affected by SLR and coastal change, including for mental health, food security, water supply, sanitation, infectious diseases, injury, and health care access.[92,95]

Future form of the indicator

In future forms of this indicator economic activity (as measured by GDP) could be included to consider the economic activity/status of places that are exposed to various levels of GMSLR; this factor will shape adaptive responses in sites of vulnerability. For example, managed retreat might be cost-effective in some coastal sites, while protection might be pursued in sites with dense populations and assets.[96,97] As newer and higher spatial resolution and more precise datasets become available, methods will be updated accordingly to produce robust estimates of population exposure to GMSLR.

Additional analysis

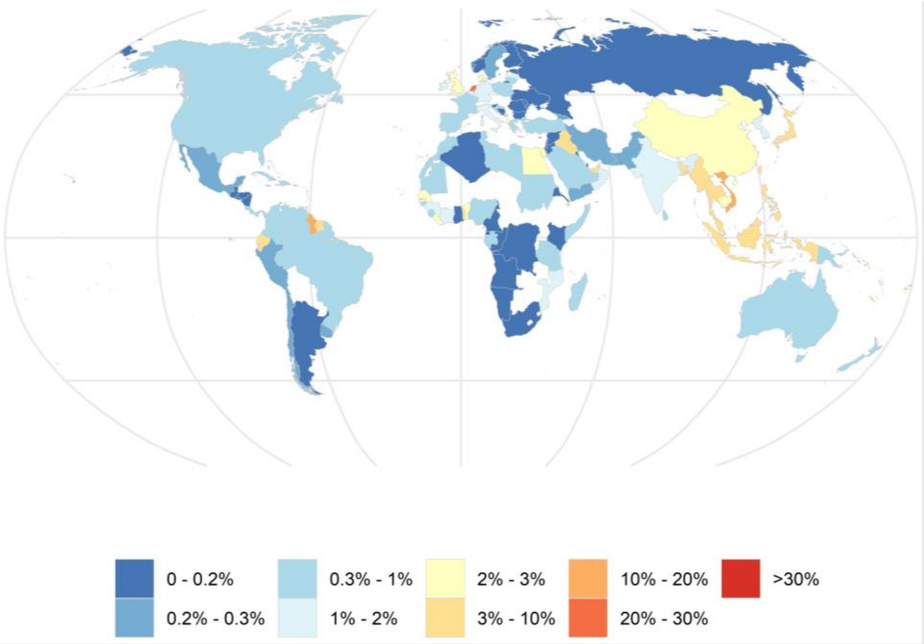

Figure 31: Proportion of national population exposed to 1m GMSLR.

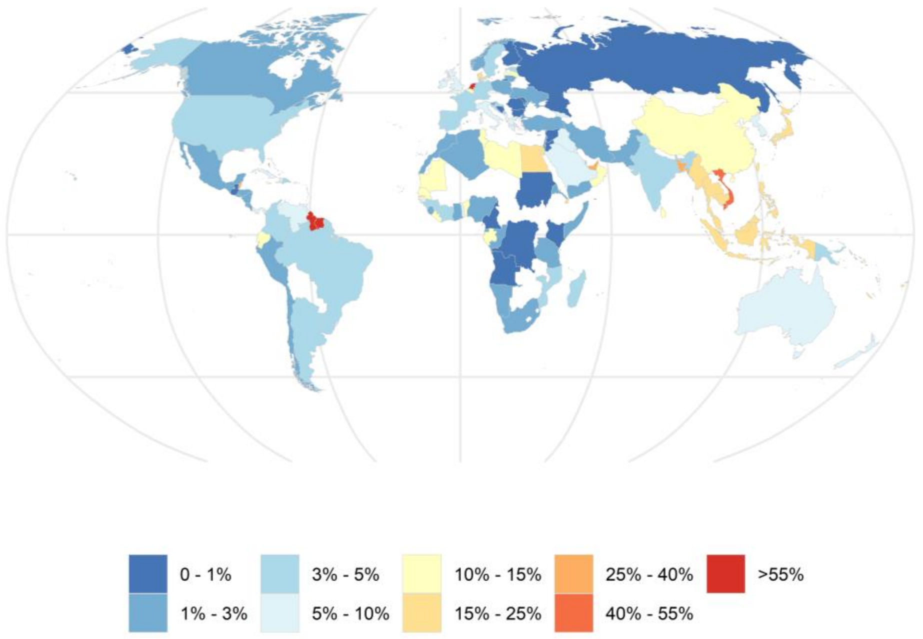

Figure 32: Proportion of national population exposed to 5m GMSLR.

National-level policies on climate change, migration, displacement, (im)mobility and relocation

Methods

This is the first year this indicator has been included in a Lancet Countdown report.

This component of this indicator on national policies reports:

1a. The number of currently valid national-level policies including legislation for migrants, migration, displacement, displaced people, relocation, and relocated people specifically related to climate change (not climate or disasters), including immobility (trapped populations/non-migration/non-displacement);

1b. The number of such policies mentioning health or well-being along with a qualitative analysis of how health and/or well-being are/is mentioned;

2a. The number of countries with at least one such policy;

2b. The number of such countries whose policies mention health or well-being along with a qualitative discussion of how health or well-being is mentioned.

“Country” refers sovereign state or autonomous non-sovereign territory (not just a sub-national jurisdiction). Multi-lateral, inter-governmental, and international policies are specifically excluded. Explicit mentions of “climate change” and “health” or “well-being” must be present, not implied definitions or references to wider contexts which might (or might not) encompass these points, e.g. ”climate”, “climate disasters”, “humanitarian”, and “environment”.

The method for identifying national-level policies is:

1. A systematic review, using the keywords which define the indicator.

2. Crowd-sourcing and expert queries (e.g. IOM).[98]

Because this search can never know what might have been missed, the numbers reported for this indicator represent minimum counts. Each policy included is also categorised by:

1. (a) Migration/mobility/displacement/relocation from a location, (b) migration/mobility/displacement/relocation to a location, and (c) immobility/trapped populations.
2. (a) Domestic migration/mobility/displacement/relocation and (b) international migration/mobility/displacement/relocation.

(All immobility, by definition, is domestic.)

A given policy might be counted in more than one category for 1abc and for 2ab. Some policies do not have an end date and some do, with both included. Policies which are now out-of-date are retained in a separate list as well as a list of policies considered but not included in this indicator.

Data

Country	Policy	Title	Website or other source
Australia		National Climate Resilience and Adaptation Strategy	https://www.environment.gov.au/system/files/resources/3b44e21e-2a78-4809-87c7-a1386e350c29/files/national-climate-resilience-and-adaptation-strategy.pdf
Austria		The Austrian Strategy for Adaptation to Climate Change. Part 1: Context	https://www4.unfccc.int/sites/NAPC/Documents%20NAP/The%20Austrian%20Strategy%20for%20Adaptation%20to%20Climate%20Change.pdf
Bangladesh		Third National Communication of Bangladesh to the United Nations Framework Convention on Climate Change	https://unfccc.int/sites/default/files/resource/TNC%20Report%20(Low%20Resolution)%2003_01_2019.pdf
Bangladesh		National Strategy on the Management of Disaster and Climate Induced Internal Displacement	https://www.preventionweb.net/files/46732_nsmdciidfinalversion21sept2015withc.pdf
Belgium		Belgium National Climate Change Adaptation Strategy	https://www.climat.be/files/4214/9880/5755/NAP_EN.pdf
Brazil	NAP	National Adaptation Plan to Climate Change — General Strategy<br><br>National Adaptation Plan to Climate Change — Sectoral and Thematic Strategies	https://www4.unfccc.int/sites/NAPC/Documents/Parties/Brazil%20NAP%20English.pdf
Bulgaria		Third National Action Plan on Climate Change - For the Period 2013-2020	https://www.preventionweb.net/files/58313_thirdnationalactionplanfortheperiod.pdf
Burkina Faso	NAP	Burkina Faso National Climate Change Adaptation Plan (NAP)	https://www4.unfccc.int/sites/NAPC/Documents/Parties/PNA_Version_version%20finale%5bTransmission%5d.pdf
Cameroon	NAP	Plan National d'Adaptation aux Changements Climatiques du Cameroun	https://www4.unfccc.int/sites/NAPC/Documents/Parties/PNACC_Cameroun_VF_Valid%c3%a9e_24062015%20-%20FINAL.pdf
Chad	INDC/NDC	Intended Nationally Determined Contribution (INDC) for the Republic of Chad	https://publications.iom.int/system/files/indcs_and_ndcs.pdf and https://www4.unfccc.int/sites/NDCStaging/Pages/All.aspx and https://www4.unfccc.int/sites/submissions/indc/Submission%20Pages/submissions.aspx
Chile	NAP	Plan Nacional de Adaptación al Cambio Climático<br><br>Plan de Adaptación al Cambio Climático del Sector Silvoagropecuario<br><br>Plan de Adaptación al Cambio Climático en Biodiversidad	https://www4.unfccc.int/sites/NAPC/Documents/Parties/Chile%20NAP%20including%20sectoral%20plans%20Spanish.pdf

		Plan de Adaptación al Cambio Climático para Pesca y Acuicultura	
Comoros	INDC/NDC	Contributions Prévues Déterminées au niveau National de l'Union des Comores	https://publications.iom.int/system/files/indcs_and_ndcs.pdf and https://www4.unfccc.int/sites/NDCStaging/Pages/All.aspx and https://www4.unfccc.int/sites/submissions/indc/Submission%20Pages/submissions.aspx
Egypt	INDC/NDC	Egyptian Intended Nationally Determined Contribution	https://publications.iom.int/system/files/indcs_and_ndcs.pdf and https://www4.unfccc.int/sites/NDCStaging/Pages/All.aspx and https://www4.unfccc.int/sites/submissions/indc/Submission%20Pages/submissions.aspx
Fiji		Planned Relocation Guidelines: A framework to undertake climate change related relocation	https://cop23.com.fj/wp-content/uploads/2018/12/CC-PRG-BOOKLET-22-1.pdf
Germany		German Strategy for Adaptation to Climate Change	https://www.bmu.de/fileadmin/bmu-import/files/english/pdf/application/pdf/das_gesamt_en_bf.pdf
Ghana		Ghana National Climate Change Policy	https://www.un-page.org/files/public/ghanaclimatechangepolicy.pdf
Ghana		National Migration Policy for Ghana	http://www.migratingoutofpoverty.org/files/file.php?name=national-migration-policy-for-ghana.pdf&site=354
Haiti	INDC/NDC	Contribution Prévue Déterminée au niveau National	https://publications.iom.int/system/files/indcs_and_ndcs.pdf and https://www4.unfccc.int/sites/NDCStaging/Pages/All.aspx and https://www4.unfccc.int/sites/submissions/indc/Submission%20Pages/submissions.aspx
Haiti		Politique Migratoire d'Haiti 2015-2030	https://www.academia.edu/16745864/Migration_Policy_of_Haiti_2015-2030_Politique_migratoire_dHa%C3%AFti_2015-2030_
Ireland		National Adaptation Framework - Building Resilience to Climate Change	https://www.dccae.gov.ie/documents/National%20Adaptation%20Framework.pdf
Kenya		Kenya National Adaptation Plan 2015-2030	https://www4.unfccc.int/sites/NAPC/Documents%20NAP/Kenya_NAP_Final.pdf
Kiribati	INDC/NDC	Republic of Kiribati Intended Nationally Determined Contribution	https://publications.iom.int/system/files/indcs_and_ndcs.pdf and https://www4.unfccc.int/sites/NDCStaging/Pages/All.aspx and https://www4.unfccc.int/sites/submissions/indc/Submission%20Pages/submissions.aspx
Kiribati		Kiribati National Framework for Climate Change and Climate Change Adaptation	http://www.president.gov.ki/wp-content/uploads/2014/08/National-Framework-for-Climate-Change-Climate-Change-Adaptation.pdf
Kiribati		Kiribati National Labour Migration Policy	https://www.unescap.org/sites/default/files/Kiribati%20National%20Labour%20Migration%20Policy.pdf
Mali		Programme d'Action National d'Adaptation aux Changements Climatiques	https://www.uncdf.org/article/4754/local-mali-programme-daction-national-dadaptation-aux-changements-climatiques
Mauritius	INDC/NDC	Intended Nationally Determined Contribution for the Republic of Mauritius	https://publications.iom.int/system/files/indcs_and_ndcs.pdf and https://www4.unfccc.int/sites/NDCStaging/Pages/All.aspx and https://www4.unfccc.int/sites/submissions/indc/Submission%20Pages/submissions.aspx
Myanmar	INDC/NDC	Myanmar's Intended Nationally Determined Contribution - INDC	https://publications.iom.int/system/files/indcs_and_ndcs.pdf and https://www4.unfccc.int/sites/NDCStaging/Pages/All.aspx and https://www4.unfccc.int/sites/submissions/indc/Submission%20Pages/submissions.aspx

Nigeria		National Migration Policy 2015	https://publications.iom.int/system/files/pdf/national_migration_policy_2015.pdf
Papua New Guinea	INDC/NDC	Intended Nationally Determined Contribution (INDC) Under the United Nations Framework Convention on Climate Change	https://publications.iom.int/system/files/indcs_and_ndcs.pdf and https://www4.unfccc.int/sites/NDCStaging/Pages/All.aspx and https://www4.unfccc.int/sites/submissions/indc/Submission%20Pages/submissions.aspx
Philippines		National Climate Change Action Plan	http://extwprlegs1.fao.org/docs/pdf/phi152934.pdf
Poland		Polish National Strategy for Adaptation to Climate Change (NAS 2020) - With the perspective by 2030	https://klimada.mos.gov.pl/wp-content/uploads/2014/12/ENG_SPA2020_final.pdf
Rwanda	INDC/NDC	Intended Nationally Determined Contribution (INDC) for the Republic of Rwanda	https://publications.iom.int/system/files/indcs_and_ndcs.pdf and https://www4.unfccc.int/sites/NDCStaging/Pages/All.aspx and https://www4.unfccc.int/sites/submissions/indc/Submission%20Pages/submissions.aspx
Solomon Islands	NAPA	National Adaptation Programmes of Action	https://unfccc.int/resource/docs/napa/slb01.pdf
Somalia	INDC/NDC	Somalia's Intended Nationally Determined Contributions (INDCs)	https://publications.iom.int/system/files/indcs_and_ndcs.pdf and https://www4.unfccc.int/sites/NDCStaging/Pages/All.aspx and https://www4.unfccc.int/sites/submissions/indc/Submission%20Pages/submissions.aspx
South Sudan	INDC/NDC	Intended Nationally Determined Contribution (Draft)	https://publications.iom.int/system/files/indcs_and_ndcs.pdf and https://www4.unfccc.int/sites/NDCStaging/Pages/All.aspx and https://www4.unfccc.int/sites/submissions/indc/Submission%20Pages/submissions.aspx
Sri Lanka	NAP	National Adaptation Plan for Climate Change Impacts in Sri Lanka: 2016-2025	https://www4.unfccc.int/sites/NAPC/Documents/Parties/SLU-NAP-May-2018.pdf
St. Lucia	NAP	Saint Lucia's National Adaptation Plan (NAP) 2018–2028	https://www4.unfccc.int/sites/NAPC/Documents/Parties/SLU-NAP-May-2018.pdf
Sudan	NAP	National Adaptation Plan	https://www4.unfccc.int/sites/NAPC/Documents%20NAP/National%20Reports/Sudan%20NAP.pdf
Suriname	INDC/NDC	Intended Nationally Determined Contribution Under UNFCCC	https://publications.iom.int/system/files/indcs_and_ndcs.pdf and https://www4.unfccc.int/sites/NDCStaging/Pages/All.aspx and https://www4.unfccc.int/sites/submissions/indc/Submission%20Pages/submissions.aspx
Vanuatu		National Policy on Climate Change and Disaster-Induced Displacement	https://ndmo.gov.vu/imhttps://ndmo.gov.vu/images/download/Vanuatu-National-Policy-on-Climate-Change-and-Disaster-Induced-Displacement-2018-published.pdfages/download/Vanuatu-National-Policy-on-Climate-Change-and-Disaster-Induced-Displacement-2018-published.pdf

Caveats

As documented in previous Lancet Countdown reports[1,7,99] and supporting publications,[87,88,100-103] the main problems with using migration or displacement as a climate change and health indicator are:

1. Attributing movement or immobility to climate change or climate change impacts is not straightforward.
2. Attributing health outcomes to movement or immobility is not straightforward.

Selecting policies, and in particular national policies, does not cover all possibilities, but it serves as an indicator. As well, it is an indicator of how national governments perceive the climate change / (im)mobility / health links, without making a statement on the actual links, which the literature explains is exceptionally difficult. This approach to the indicator also means that misattributions are easily filtered out, such as reporting

migration and health links to disasters or climate, both of which are different from links to climate change. Using ‘climate change’ synonymously with ‘climate’, ‘climate-related disasters’, and/or ‘disasters’, is a common mistake in many policies reviewed as well as in the academic literature.

The main caveat is that most of the data is confined to documents in English, with a few other languages on occasion. The advantage is that policies which are not available in English have typically been discussed in English publications, including blogs and news reports, suggesting that much relevant material has been captured. Nonetheless, the numbers reported can only be taken as the minimum, as in ‘at least so many’ policies match the criteria stated. One minor caveat is that the number of countries sometimes changes year-to-year, providing a different baseline. These changes are rarely more than 1-2 countries per year out of a sample of around 200. Substantial changes to the numbers of countries will be reported, if this occurs.

The indicator design helps in overcoming these caveats by reporting that the counts provided must be only minimum numbers. Through publicity, publication, crowd sourcing, and expert connections, this limitation will be overcome because people will provide examples of what it has been missed. As an indicator, it is important to accept that the numbers are not comprehensive but provide only minimum numbers as a lower-bound baseline.

Section 2: Adaptation, Planning, and Resilience for Health

2.1: Adaptation Planning and Assessment

Indicator 2.1.1: National Adaptation Plans for Health

Methods

The collection of data for this exercise included a voluntary national survey, the WHO Health and Climate Change Survey (2018)[104] that was sent to all WHO member states and was completed by ministry of health focal points. Of the 194 WHO member states, 101 participated in the survey, providing representation from all 6 WHO regions, World Bank Group-defined income categories, and a diverse range of threats and vulnerabilities to the health effects of climate change.

Survey participation has grown substantially from the 40 Member States that completed the 2015 WHO Health and Climate Change Survey.

The survey is conducted every two years, as such, data in this year's report is a further analysis of the data used in the 2019 report of the Lancet Countdown.[1]

Validation of the 2018 country reported data was undertaken in multiple steps. First, survey responses were reviewed for missing information or inconsistencies with follow-up questions directed to survey respondents. A summary of responses was shared with WHO regional focal points for review and comments. Source documents including national health strategies and plans, and scientific assessments of health vulnerabilities and assessments were collected. A desktop review was conducted to compare with survey results with follow-up to survey respondents to seek clarification or additional documentation. In the case of vulnerability and adaptation assessments, findings were also cross referenced with existing external publications.[105] Finally, partial results were reviewed by key national health and climate stakeholders and ministry of health officials as part of the development and review of the WHO UNFCCC health and climate change country profiles.

Further information on the WHO Health and Climate Change Survey, its methodology and the WHO UNFCCC Health and Climate Change Country Profile Initiative can be found at https://www.who.int/globalchange/resources/countries/en/

Data

1. 2018 WHO Health and Climate Change Survey.[104]

Caveats

The survey sample is not a representative sample of all countries as this survey was voluntary, however, the inclusion of 101 countries in this survey compared with 40 in the 2015 survey demonstrates a large increase in coverage. Additionally, the WHO is running a climate change and health special initiative in Small Island Developing States and there are 26 small island developing countries and territories represented within the total number of respondents.

Future form of the indicator

The WHO Health and Climate Change Survey will be conducted biennially and will continue to be the primary source of data to track this indicator. Next year, new survey data will be available for this indicator.

The future evolution of this indicator will explore the monitoring and review of the existing strategies/plans and progress on level of implementation of strategies/plans. With more countries initiating the national adaptation plan (NAP) process, alignment of the health component with the overall NAP will also be more closely

monitored. Interim information regarding the specific content of national strategies/plans, as explored in this qualitative analysis, may be re-assessed in the future.

Indicator 2.1.2: National Assessments of Climate Change Impacts, Vulnerability, and Adaptation for Health

Methods

Similar to the methods provided for Indicator 2.1.1, national assessments of vulnerability, impacts and adaptation for health (health V&As) were monitored through the 2018 WHO Health and Climate Change Survey.

The collection of data for this exercise included a voluntary national survey, the WHO Health and Climate Change Survey (2018)[104] that was sent to all WHO member states and was completed by ministry of health focal points. Of the 194 WHO member states, 101 participated in the survey, providing representation from all 6 WHO regions, World Bank Group-defined income categories, and a diverse range of threats and vulnerabilities to the health effects of climate change.

Survey participation has grown substantially from the 40 Member States that completed the 2015 WHO Health and Climate Change Survey.

The survey is conducted every two years, as such, data in this year's report is a further analysis of the data used in the 2019 report of the Lancet Countdown.[1]

Validation of the 2018 country reported data was undertaken in multiple steps. First, survey responses were reviewed for missing information or inconsistencies with follow-up questions directed to survey respondents. A summary of responses was shared with WHO regional focal points for review and comments. Source documents including national health strategies and plans, and scientific assessments of health vulnerabilities and assessments were collected. A desktop review was conducted to compare with survey results with follow-up to survey respondents to seek clarification or additional documentation. In the case of vulnerability and adaptation assessments, findings were also cross referenced with existing external publications.[1] Finally, partial results were reviewed by key national health and climate stakeholders and ministry of health officials as part of the development and review of the WHO UNFCCC health and climate change country profiles.

Further information on the WHO Health and Climate Change Survey, its methodology and the WHO UNFCCC Health and Climate Change Profile Initiative can be found at https://www.who.int/globalchange/resources/countries/en/

Data

1. 2018 WHO Health and Climate Change Survey.[104]

Caveats

The survey sample is not a representative sample of all countries as this survey was voluntary, however, the inclusion of 101 countries in this survey compared with 40 in the 2015 survey demonstrates a large increase in coverage. Additionally, the WHO is running a climate change and health special initiative in Small Island Developing States and there are 26 small island developing countries and territories represented within the total number of respondents.

Future form of the indicator

The WHO Health and Climate Change Survey will be conducted biennially and will continue to be the primary source of data to track this indicator. Next year, new survey data will be available for this indicator. The future evolution of this indicator will explore the coverage and comprehensive of the assessments, such as the use of qualitative and/or quantitative data and the use of future projections of risks of climate-sensitive diseases.

Indicator 2.1.3: City-Level Climate Change Risk Assessments

Methods

The CDP serves as an official reporting platform for the Compact of Mayors. It administrates, collects and analyses a global survey of city based environmental and climate change data on an annual basis.

In 2019, 814 cities participated in the survey, with 789 reporting publicly, that included questions on emissions, adaptation assessments and plans. This represents a 33% increase in cities responding to the CDP survey.

Respondents to the surveys to describe the magnitude of the impact of climate-based hazards (extremely serious, serious, less serious) and identify three critical assets or services that may be most impacted. Based on this data two indicators can be developed.

The first is a global cities-based indicator of government areas that have undertaken a climate change risk or vulnerability assessment.

The second is global cities-based indicator of the perceived vulnerability of public health assets and service to climate change.

Data

1. 2019 CDP Annual Cities Survey.[106]

Caveats

This is a sample survey and cities are under no obligation to respond. As such the survey may suffer from selection bias. The majority of responding cities are also from High Income Countries (69%). As such, the results are not representative.

Future form of the indicator

The CDP collect this data annually and it is foreseen that the data collection will continue to 2030. In subsequent annual surveys, additional questions related specifically to health risks will be included. They will be piloted and tested in the 2020 survey.

Additional analysis

Table 3: Cities that responded to the 2019 CDP survey by World Bank income group

Income group	Freq.	Percent
High income	394	48.4%

Upper middle income	319	39.2%
Lower middle income	78	9.6%
Low income	23	2.8%
**Total Cities**	**814**	

Table 4: Cities by CPD Region that have undertaken a climate change risk or vulnerability assessment at the local government area.

	**Africa**	**East Asia**	**Europe**	**Latin America**	**North America**	**Middle East**	**South Asia & Oceania**	**South & West Asia**
Yes	31	15	116	137	127	2	51	12
No	1	1	4	6	3	N/A	N/A	N/A
In Progress	8	1	26	50	21	1	5	2
Intend Future	6	N/A	16	49	31	1	9	1
Don't Know	3	N/A	4	36	9	2	2	N/A
**Total**	**49**	**17**	**166**	**278**	**191**	**6**	**67**	**15**

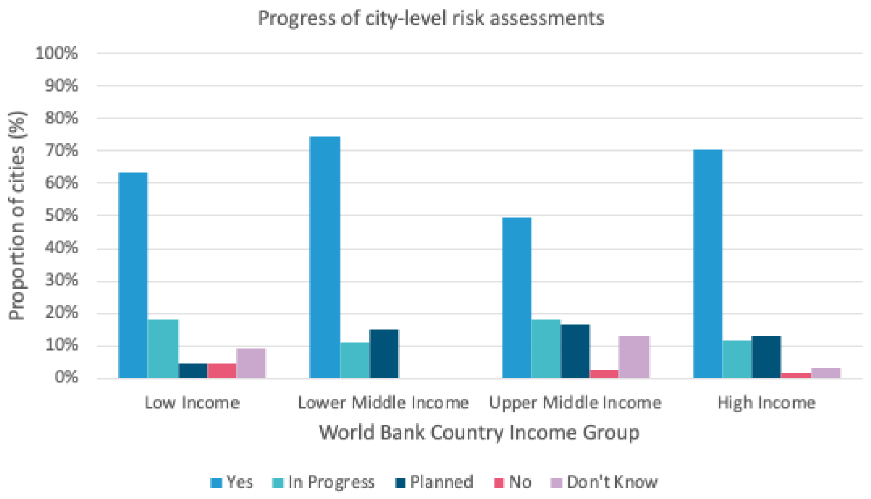

Figure 33: Proportion of cities that have conducted climate change risk assessments, by World Bank income group.

Indicator 2.2: Climate Information Services for Health

Methods

The number of World Meteorological Organization (WMO) national member states (NMS) whose Meteorological and Hydrological services are providing climate services to the health sector is calculated based on self-reported information provided by member states to the World Meteorological Organization (WMO) through the Country Profile Database Integrated questionnaire. The questionnaire is one of the main sources of information to the WMO Country Profile data base and is open all year round for WMO members to update their profile information.

Reported data reflects answers to Question number 7.6 of this questionnaire: "Please indicate which user communities/sectors your NMS provides with climate products/information and estimate the extent to which these products are used to improve decisions". "Human Health" is one of multiple sectors which can be chosen.

Data

1. World Meteorological Organization Country Profile database.[107]

Caveats

The current data source from WMO only considers climate services provided by NMS. It is unclear the degree to which other providers, such as academic institutions and research projects, private sector products, products from other Ministries, or regional and global products and services are being used, in proportion to services made available by NMS.

The open questionnaire can be updated at any time by WMO members, therefore the figures here reported may change over the year. As each country may update their profile information at different moments in time, snapshots do not reflect progress for any given year but rather information provided until a certain date.

The current questionnaire does not record the number of WMO members that do not provide climate services to the health sector.

The questionnaire captures information on the provision of climate services, the status of service provision to the health sector (divided in 5 categories) and the type of services provided (divided in 5 categories as well). However, only the provision and status of climate service has been reported here due to uncertainties over the quality of the data on the type of services provided. Questions do not capture the source or quality of the service and only one of the answer options covers the utility of the climate services. They do not capture whether data originates from national meteorological observations or is resulting from regional or global products. They do not capture the potential use of all-sector forecasts or outlooks which are accessed and used by the health sector.

The WMO and WHO have some differences in their individual Member States. Responses collected from WMO Member States, were reclassified according to WHO Region. WMO members that are not individual WHO members were excluded from the analyses and include Macao and Hong Kong (reported as China), Curaçao, French Polynesia, and St. Maartens. The following WHO Members are not members of WMO (and therefore representative data is not available): Andorra, Equatorial Guinea, Marshall Islands, Nauru, Palau, San Marino.

Future form of the indicator

In 2019, WMO began implementation of new survey instruments to provide greater insight on the status of climate service provision for the health sector and the type of service provided. Other complementary WMO surveys capturing specific product types, user satisfaction, and application areas, may be publicly available in the future to inform future editions of this indicator.

The WHO Health and Climate Change Country Survey now contains indicators on the inclusion of meteorological information in integrated risk monitoring and early warning systems for climate-sensitive diseases. This information may be used to improve this indicator in future publications.

Additional analysis

Table 5: Provision of climate services from national meteorological and hydrological services by sector.

**Sector**	**Number of countries providing climate services**
Government	106
Agriculture	99
Local authorities	96
Water resources	95
Scientific	91
Energy	87
**Human Health**	**86**
Emergency planning and response	86
Transport	81
Environmental protection	76
Building	76
Aviation	75
Commercial	73
Tourism	71
Finance and insurance	71
Forestry	67
Fisheries	59
Recreation, sport	59
Maritime transport	54

Responding countries provided additional information on how well the climatic services are integrated in the health sector. 66 of the 86 respondents indicated that climate services were highly engaged or in the process of being scoped for further engagement with the health sector.

Full list of countries providing climate services to the health sector: Angola, Antigua and Barbuda, Argentina, Armenia, Australia, Austria, Barbados, Belgium, Bhutan, Bolivarian Republic of Venezuela, Bosnia and Herzegovina, Brazil, Brunei Darussalam, Cameroon, Canada, Chad, Chile, China, Colombia, Cook Islands, Cote d'Ivoire, Croatia, Cyprus, Djibouti, Dominica, Ecuador, Egypt, El Salvador, Fiji, Finland, France, Georgia, Germany, Guinea Bissau, Hungary, Iceland, India, Indonesia, Iraq, Ireland, Jamaica, Japan, Kazakhstan, Kenya, Kuwait, Latvia, Lesotho, Madagascar, Malawi, Malaysia, Maldives, Mali, Mexico, Morocco, Mozambique, Myanmar, Namibia, Nepal, Niger, Nigeria, Niue, North Macedonia, Peru, Philippines, Qatar, Republic of

Korea, Russian Federation, Sao Tome and Principe, Saudi Arabia, Senegal, Serbia, Singapore, Slovenia, Solomon Islands, South Africa, Spain, Sri Lanka, Sudan, Thailand, Trinidad and Tobago, Turkey, Ukraine, United Kingdom, United Republic of Tanzania, United States of America, Vanuatu, Zimbabwe.

2.3: Adaptation Delivery and Implementation

Indicator 2.3.1: Detection, Preparedness and Response to Health Emergencies

Methods

This indicator takes data from the International Health Regulations (IHR (2005)) State Party Self- Assessment Annual Reporting Tool (SPAR).

Under the IHR (2005) all States Parties are required to have or to develop minimum core public health capacities to implement the IHR (2005) effectively. IHR (2005) also states that all States Parties should report to the World Health Assembly annually on the implementation of IHR (2005). In order to facilitate this process, WHO developed an IHR Monitoring questionnaire, interpreting the Core Capacity Requirements in Annex 1 of IHR (2005) into 20 indicators for 13 capacities. Since 2010, this self-reporting IHR monitoring questionnaire is sent annually to National IHR Focal Points (NFPs) for data collection. It contains a checklist of 20 indicators specifically developed for monitoring the development and implementation of 13 IHR capacities. The method of estimation calculates the proportion/percentage of attributes (a set of specific elements or functions which reflect the level of performance or achievement of a specific indicator) reported to be in place in a country.

The core capacities to implement the International Health Regulations (2005) have been established by a technical group of experts, as those capacities required to detect, assess, notify and report events, and to respond to public health risks and emergencies of national and international concern. To assess the development and strengthening of core capacities, a set of components are measured for each of the core capacities, by considering a set of one to three indicators that measure the status and progress in developing and strengthening the IHR core capacities. Each indicator is assessed by using a group of specific elements referred to as ‘attributes’ that represents a complex set of activities or elements required to carry out this component. The annual questionnaire has been conducted since 2010 with a response rate of 72% in 2012, 66% in 2016 and 85% in 2017, and 100% of countries reporting at least once since 2010. Annual reporting results are complemented by after action reviews, exercises, and joint external evaluation (JEE).

At the beginning of 2018, in compliance with the recommendations of the IHR Review Committee on Second Extensions for Establishing National Public Health Capacities and on IHR Implementation , and following formal global consultations with States Parties held in 2015, 2016, and 2017, and 2018, the WHO Secretariat replaced the IHR Monitoring questionnaire by the “IHR State Party Self-assessment Annual Reporting (SPAR) Tool”. This has strong implication for the future of this indicator: preparedness and response capacities have now been merged into one capacity called “C8: National health emergency framework”; one capacity relevant to climate adaptation and resilience has been added (“C9: Health services provision”); and capacity grading has been introduced, which requires countries to grade their capacity indicators in progressive levels from 0 to 5 as opposed to the previous “Yes/No/Not know” answers options. C8 contains three components. A full breakdown of the 0-5 scale for each of the three components is provided in the 2019 Lancet Countdown report appendix.[1]

Data

1. International Health Regulations (2005) Annual Reporting. Data is available through the Global Health Observatory Data Repository for 2010-2017,[108] and through the SPAR interactive for 2019

Caveats

There are some limitations to considering these capacities as proxies of health- system adaptive capacity and system resilience. Most importantly, IHR monitoring questionnaires responses are self-reported. Secondly, the countries that report IHR implementation annually differ from year to year within these regional aggregate scores. Thirdly, IHR Core Capacity Requirements are not specific to climate change, and hence whilst they provide a proxy baseline, they do not directly measure a country's adaptive capacity in relation to climate driven risk changes. Fourthly, these findings capture potential capacity – not action. Finally, the quality of surveillance for early detection and warning is not shown and neither is the impact of that surveillance on public health. Response systems have been inadequate in numerous public health emergencies and thus the presence of such plans is not a proxy for their effectiveness. Nonetheless, these four capacities provide a useful starting point to consider the potential adaptive capacity of health systems globally.

Additional analysis

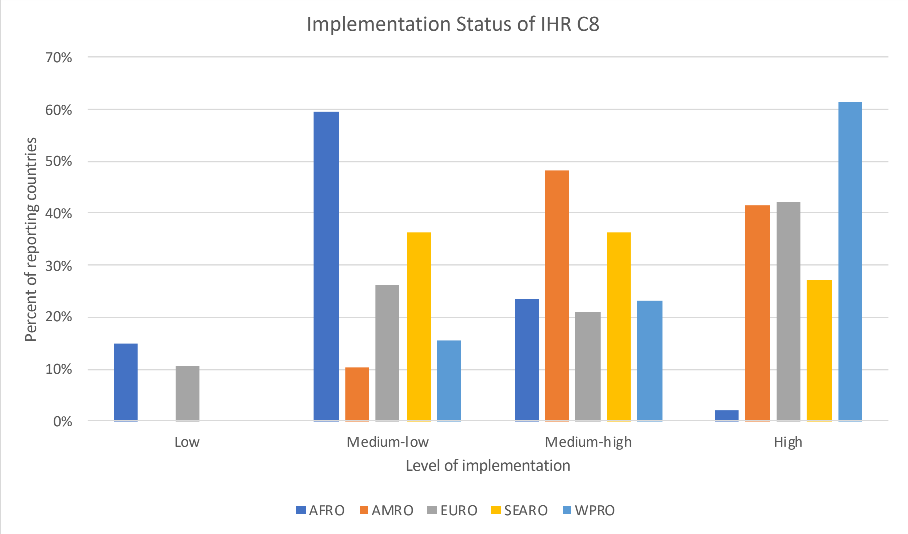

Figure 34: Level of implementation of the IHR C8 capacity in 2019, by WHO region

Reporting countries in 2019: Afghanistan, Albania, Algeria, Andorra, Angola, Argentina, Armenia, Australia, Austria, Azerbaijan, Bahamas, Bahrain, Bangladesh, Belgium, Benin, Bhutan, Bosnia and Herzegovina, Botswana, Brazil, Burkina Faso, Burundi, Cabo Verde, Cambodia, Cameroon, Canada, Central African Republic, Chad, Chile, China, Colombia, Comoros, Congo, Costa Rica, Cote d'Ivoire, Croatia, Cuba, Cyprus, Czech Republic, Democratic People's Republic of Korea, Democratic Republic of the Congo, Denmark, Djibouti, Dominica, Dominican Republic, Ecuador, Egypt, El Salvador, Equatorial Guinea, Eritrea, Eswatini, Ethiopia, France, Gabon, Gambia, Georgia, Germany, Ghana, Greece, Guatemala, Guinea, Guinea-Bissau, Guyana, Honduras, Hungary, Iceland, India, Indonesia, Iraq, Ireland, Israel, Jamaica, Japan, Jordan, Kazakhstan, Kenya, Kuwait, Kyrgyzstan, Lao People's Democratic Republic, Latvia, Lebanon, Lesotho, Liberia, Libya, Liechtenstein, Lithuania, Luxembourg, Madagascar, Malawi, Maldives, Mali, Malta, Mauritania, Mauritius, Mexico, Micronesia, Monaco, Mongolia, Morocco, Mozambique, Myanmar, Namibia, Nepal, Netherlands, Nicaragua, Niger, Nigeria, Niue, North Macedonia, Norway, Oman, Pakistan, Palau, Panama, Paraguay, Peru, Philippines, Poland, Portugal, Qatar, Republic of Korea, Republic of Moldova, Romania, Russian Federation, Rwanda, Saint Kitts and Nevis, Saint Lucia, Saint Vincent and the Grenada, Sao Tome and Principe, Saudi Arabia, Senegal, Serbia, Seychelles, Sierra Leone, Singapore, Slovakia, Slovenia, South Africa, South Sudan, Spain, Sri Lanka, Sudan, Suriname, Sweden, Switzerland, Syrian Arab Republic, Tajikistan, Tanzania, Thailand, Timor-Leste, Togo, Trinidad and Tobago, Tunisia, Turkey, Turkmenistan, Uganda, Ukraine, United Arab Emirates, United Kingdom, United States of America, Uruguay, Uzbekistan, Venezuela, Vietnam, Yemen, Zambia, Zimbabwe.

Indicator 2.3.2: Air Conditioning Benefits and Harms

Methods

A meta-analysis published in 2007 by Bouchama et al. found having home air conditioning to be the strongest protective factor against heatwave-related mortality (pooled relative risk [RR] = 0.23; 95% confidence interval: 0.1, 0.6; based on six studies) and having visited other air-conditioned places also to be highly protective (pooled RR = 0.34; 95% confidence interval: 0.2, 0.5; based on five studies).[109] Thus, residential air conditioning is of special interest with regard to protection against heatwave-related mortality.

The prevented fraction is the percent reduction in an adverse health outcome due to a preventive exposure, compared with the scenario of complete absence of the exposure.[110] The prevented fraction is determined by two factors: 1) the RR of the adverse health outcome in exposed persons compared with unexposed persons; and 2) the prevalence of the exposure. The prevented fraction increases with decreasing relative risk and with increasing prevalence of exposure. The formula for prevented fraction is simply:

$$P_e(1 - RR)$$

Where P_e is the prevalence of the exposure and RR is the relative risk of the adverse health outcome in exposed persons compared with unexposed persons.

For the air conditioning indicator, the prevented fraction is the percent reduction in heatwave-related deaths due to a given proportion of the population (P_{ac}) having household air conditioning, compared with a scenario of complete absence of household air conditioning. Thus, the prevented fraction is simply:

$$P_{ac}(1 - RR_{ac})$$

Where RR_{ac} is the relative risk of death during a heatwave or hot weather among persons who have household air conditioning compared with persons who do not have household air conditioning.

As intuitively expected, according to this formula, the higher the protection against heatwave-related mortality conferred by household air conditioning (i.e., the lower the relative risk of heatwave-related mortality in persons living in a household with air conditioning versus persons living in a household without air conditioning), the greater the prevented fraction; and the higher the proportion of the population with access to household air conditioning, the greater the prevented fraction.

To estimate prevented fraction, P_{ac} was assumed to be the same as the proportion of households with air conditioning (kindly provided by the International Energy Agency, IEA). For the 2019 Lancet Countdown report, to estimate prevented fraction a RR_{ac} of 0.23 was used, taken from the 2007 meta-analysis.[109] However, for the 2020 Lancet Countdown report, this RR_{ac} was updated in a new meta-analysis. First, literature was searched for non-ecologic, analytical epidemiologic studies that examined the relationship between availability of household air conditioning and heatwave-related mortality, and 12 articles were identified, six of which were included in the 2007 meta-analysis.

Second, the 12 articles were reviewed and three were eliminated:

1) a grey-literature report on the 2003 French heatwave included in the 2007 meta-analysis that was subsequently published in a peer reviewed journal (the peer-reviewed publication was included in the updated meta-analysis);

2) a study included in the 2007 meta-analysis in which the air conditioning exposure was measured as hours per day of air conditioning use (as opposed to simply having air conditioning in the house versus not having air conditioning in the house, as measured by the included studies); and

3) a study that presented results separately for central air conditioning versus no air conditioning and room air conditioning versus no air conditioning, with no way to combine the results into any air conditioning (central or room) versus no air conditioning.

Third, a random-effects meta-analysis was conducted that included the nine remaining studies[111-119] and the pooled RR_{ac} was calculated to be 0.24 (95% confidence interval: 0.15, 0.39) which was then used to calculate

the prevented fraction. This pooled RR_{ac} does not meaningfully differ from the pooled RR_{ac} of 0.23 from the 2007 meta-analysis that was used in the 2019 Lancet Countdown report.[1]

Thus, the formula for prevented fraction is:

$$P_{ac}(1 – RR_{ac}) = P_{ac}(1 – 0.24) = P_{ac}(0.76)$$

The prevented fraction could range from 0 for a country or region with no household air conditioning (i.e., $P_{ac} = 0$) to 76% for a country or region in which every household has air conditioning (i.e., $P_{ac} = 1.0$). A low prevented fraction does not necessarily translate into a high absolute number of heatwave-related deaths because in a given country or region the number of heatwave-related deaths that would occur in the complete absence of household air conditioning may be low.

Data

1. The IEA kindly provided data for 2000-2018, including revisions based on improved IEA analyses of the 2000-2016 data provided for the 2019 Lancet Countdown report. This data included the proportion of households with air conditioning (used for the prevented fraction calculation) and CO_2 emissions due to air conditioning (megatons) for the entire world and for major countries/regions. Data were provided for 11 individual countries (USA, Canada, China, India, Japan, Korea, Indonesia, Mexico, Brazil, Russia, and South Africa), and 6 regions (Europe, Caspian, Middle East, North Africa, Other Africa, and Rest of World), defined as follows:
    a. Europe: Albania, Austria, Belarus, Belgium, Bosnia and Herzegovina, Bulgaria, Croatia, Cyprus, Czech Republic, Denmark, Estonia, Finland, Former Yugoslav Republic of Macedonia, France, Germany, Gibraltar, Greece, Holy See, Hungary, Iceland, Ireland, Israel, Italy, Kosovo, Latvia, Lithuania, Luxembourg, Malta, Moldova, Monaco, Montenegro, Netherlands, Norway, Poland, Portugal, Romania, San Marino, Serbia, Slovak Republic, Slovenia, Spain, Sweden, Switzerland, Turkey, Ukraine, United Kingdom
    b. Caspian: Armenia, Azerbaijan, Georgia, Kazakhstan, Kyrgyzstan, Tajikistan, Turkmenistan, Uzbekistan
    c. Middle East: Bahrain, Iran, Iraq, Jordan, Kuwait, Lebanon, Oman, Qatar, Saudi Arabia, Syria, United Arab Emirates, Yemen
    d. North Africa: Algeria, Egypt, Libya, Morocco, Tunisia
    e. Other Africa: Angola, Benin, Botswana, Burkina Faso, Burundi, Cameroon, Cape Verde, Central African Republic, Chad, Comoros, Congo, Côte d'Ivoire, Dem. Republic of the Congo, Djibouti, Equatorial Guinea, Eritrea, Eswatini, Ethiopia, Gabon, Gambia, Ghana, Guinea, Guinea-Bissau, Kenya, Lesotho, Liberia, Madagascar, Malawi, Mali, Mauritania, Mauritius, Mozambique, Namibia, Niger, Nigeria, Réunion, Rwanda, Sao Tome and Principe, Senegal, Seychelles, Sierra Leone, Somalia, South Sudan, Sudan, Togo, Uganda, Tanzania, Zambia, Zimbabwe
    f. Rest of world: Afghanistan, Antigua and Barbuda, Argentina, Aruba, Australia, Bahamas, Bangladesh, Barbados, Belize, Bermuda, Bhutan, Bolivia, British Virgin Islands, Brunei Darussalam, Cambodia, Cayman Islands, Chile, Macau (China), Taiwan (China), Colombia, Cook Islands, Costa Rica, Cuba, Curaçao, Dem. People's Republic of Korea, Dominica, Dominican Republic, Ecuador, El Salvador, Falkland Islands (Malvinas), Fiji, French Guiana, French Polynesia, Grenada, Guadeloupe, Guatemala, Guyana, Haiti, Honduras, Jamaica, Kiribati, Lao People's Democratic Republic, Malaysia, Maldives, Martinique, Mongolia, Montserrat, Myanmar, Nepal, Netherlands Antilles, New Caledonia, New Zealand, Nicaragua, Pakistan, Palau, Panama, Papua New Guinea, Paraguay, Peru, Philippines, Pitcairn, Saint Kitts and Nevis, Saint Lucia, Saint Pierre and Miquelon, Saint Vincent and the Grenadines, Samoa, Singapore, Sint Maarten (Dutch part), Solomon Islands, Sri Lanka, Suriname, Thailand, Timor-Leste, Tonga, Trinidad and Tobago, Turks and Caicos Islands, Uruguay, Vanuatu, Venezuela, Vietnam

Caveats

For the prevented fraction calculation, an RR_{ac} of 0.24 was assumed for heatwave-related death for persons living in a household with air conditioning versus persons living in a household without air conditioning, based on a meta-analysis that included nine studies: four from the USA; two from France; one from Italy, one from Greece, and one from Australia. This RR_{ac} may differ in other parts of the world. Furthermore, the proportion of households with air conditioning was used to estimate the proportion of the population having household air conditioning. The estimate did not take into account the size of households with air conditioning versus those without air conditioning or the vulnerability to heat stress of persons living in households with air conditioning versus those without air conditioning. In addition, the presence of air conditioning in a household does not guarantee the use of air conditioning in that household. Finally, data limitations prevented the estimation of the absolute number of heatwave-related deaths prevented by air conditioning.

Future form of the indicator

As new studies become available, the meta-analysis of the relationship between having household air conditioning and heatwave-related (or, more generally, heat-related) mortality will be updated. If there are sufficient studies, morbidity will also be examined. The indicator may be updated each year as new data become available on air conditioning use. City-level case studies to estimate absolute number of lives saved from air conditioning versus premature deaths from exposure to $PM_{2.5}$ due to air conditioning may also be performed. Additionally, national building codes, minimum energy performance standards and labelling rules for air conditioners, and progress on implementing the Kigali Amendment may be tracked in the future.

Additional analysis

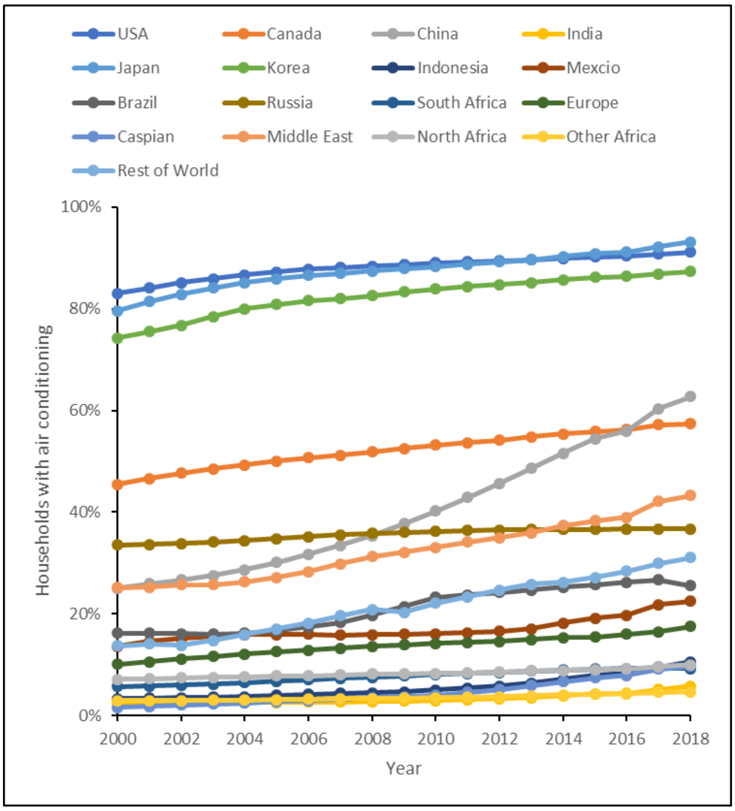

Figure 35: Percent of households with air conditioning, by selected countries/regions.

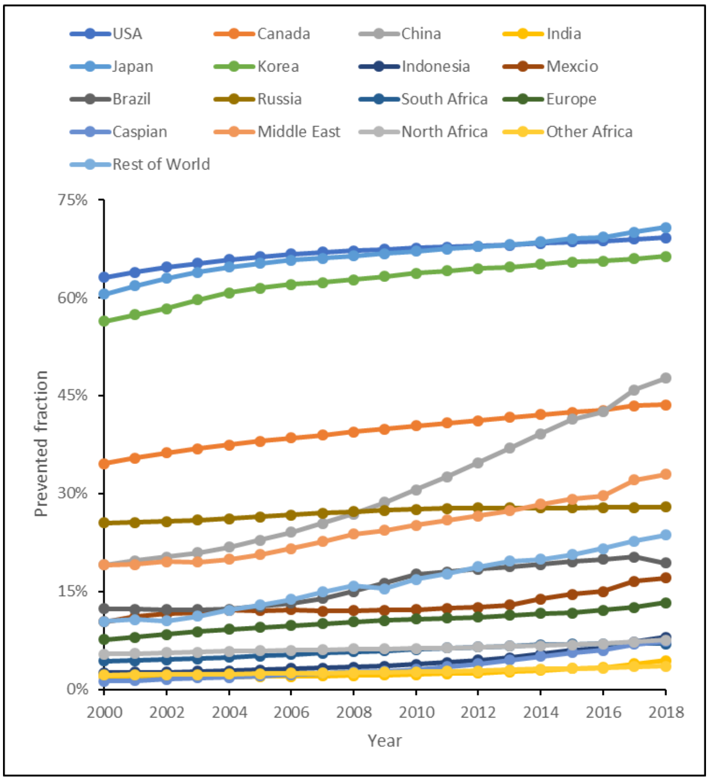

Figure 36: Prevented fraction of heatwave-related mortality due to air conditioning by selected countries/regions.

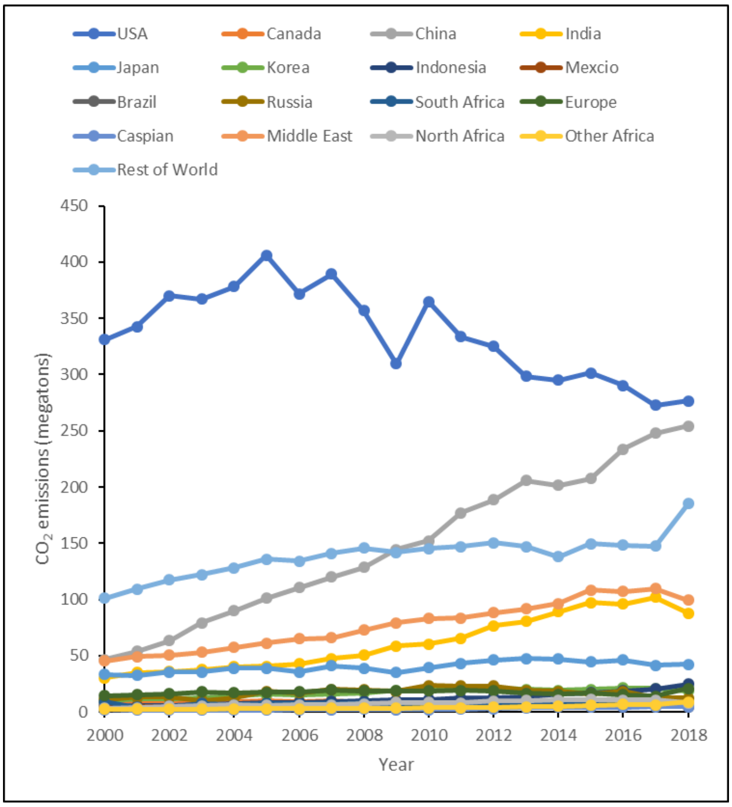

Figure 37: CO_2 emissions from air conditioning by selected countries/regions

Indicator 2.3.3: Urban Green Space

Methods

Urban areas were obtained from the Global Human Settlement program of the European Commission, which uses remote sensing and demographic data to define more than 10,000 urban centres worldwide.[120] 468 urban centres larger than one million inhabitants were selected for this indicator.

Data on population size per urban centre were collected from the Center for International Earth Science Information Network - CIESIN - Columbia University, which models the distribution of human population (counts and densities) on a continuous global raster surface with an output resolution of 30 arc-seconds (approximately 1 km at the equator).[121]

Green space magnitude was estimated using the Normalized Difference Vegetation Index (NDVI), the most commonly used satellite image–based vegetation index. Chlorophyll in plants absorbs red light (with wavelengths of 0.635–0.700 μm) for use in photosynthesis, whereas leaves reflect near-infrared light (0.7–1.1 μm). NDVI calculates the ratio of the difference between reflectance of the near-infrared wavelengths (which is high for green plants) and reflectance of red reflectance (which is low for plants) to the sum of these two measures. NDVI ranges from –1.0 to 1.0, with larger values indicating higher levels of vegetative density.[122] Publicly available data from the Moderate resolution Imaging Spectroradiometer (MODIS) from NASA's Terra satellite was used.[123] MODIS provides images every 16 days at a 250-m resolution since 1999.[124] MODIS data was downloaded quarterly (to account for every season) for 2019.

A seasonally time-varying measure was created, based on the NDVI for a representative month in each season (January, April, July, and October). Four exposure metrics were calculated for each urban centre: Peak NDVI (maximum NDVI across all seasons); four-season average NDVI; population weighted average based on peak NDVI; and population weighted average based on four-season NDVI (accounts for population size per raster). The population-weighted NDVI was estimated by multiplying each NDVI value (peak or four-season average) by the population size within the same raster, summing up over the weighted values, and dividing by the sum of the weights.

Google Earth Engine was used to estimate the four different exposure metrics described above: peak NDVI, four-season average NDVI, and both population weighted averages NDVI (based on peak and four-season average) for each urban centre.

Levels of greenness were categorised as follows:

Level of greeness	NDVI
Exceptionally low	<0.19
Very low	0.20-0.29
Low	0.30-0.39
Moderate	0.40-0.49
High	0.50-0.59
Very high	0.60-0.69
Exceptionally high	>0.70

Data

1. Urban areas were obtained from the Global Human Settlement program of the European Commission.[120]
2. Population size per urban centre were collected from the Center for International Earth Science Information Network - CIESIN - Columbia University. [121]
3. Satellite data from moderate resolution Imaging Spectroradiometer (MODIS) from NASA's Terra satellite.[123]

Caveats

Some limitations of this analysis must be noted. First, although satellite-based measures of vegetation have been used extensively to measure greenness, NDVI does not correspond directly to the quality of greenness (curated

park vs vacant lot), the type of green space (park vs forest), the type of vegetation (trees vs flowers), nor does it identify access to the green space (public vs private). Nevertheless, a validation study demonstrated that NDVI performs adequately when compared with environmental psychologists' evaluations of green spaces.[125] Moreover, the authors have previously reviewed the literature on greenness and health and found consistent and strong evidence of associations for higher greenness measured by NDVI, with improvements in birth weights and physical activity, lower mortality rates, as well as lower levels of depression.[126-128] In addition, NDVI is a publicly available commonly used metric that is gathered consistently across the globe over time, which will enable comparisons across locations and between studies. In addition, the number of urban centres included in this analysis was restricted to those with a population higher than one million. These urban centres are defined by the Global Human Settlement Program, based on clusters of contiguous 1km^2 grid cells of at least 1,500 inhabitants or at least 50% built-up surface share per km^2.[129] Using this definition, some of the urban centres of >1 million inhabitants consist of clusters of smaller urban centres and thus not all 467 urban centres captured represent cities of over 1 million inhabitants. . Future versions of this indicator will aim to improve its capture of cities around the globe and will examine trends over time.

Future form of the indicator

This indicator will be expanded to include a larger number of global cities. It will also aim to estimate the proportion of each city that is green space, in addition to the average greenness of a city. Additional analysis will be done around historic trends.

Additional analysis

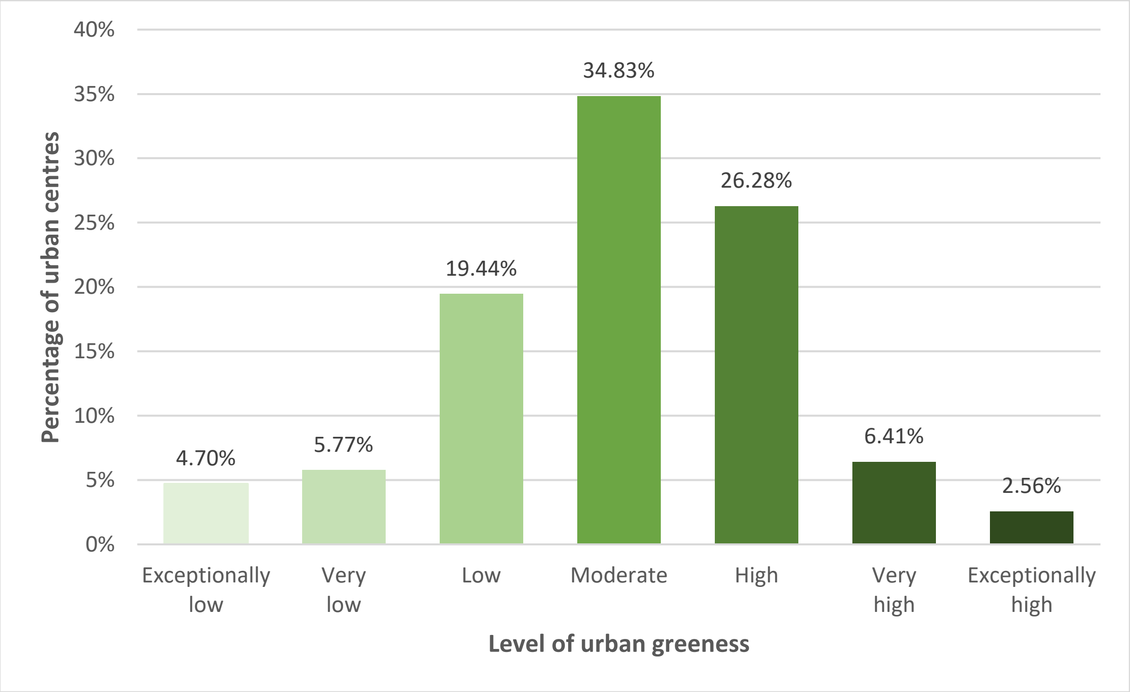

Figure 38: Distribution of global urban centres by level of greenness.

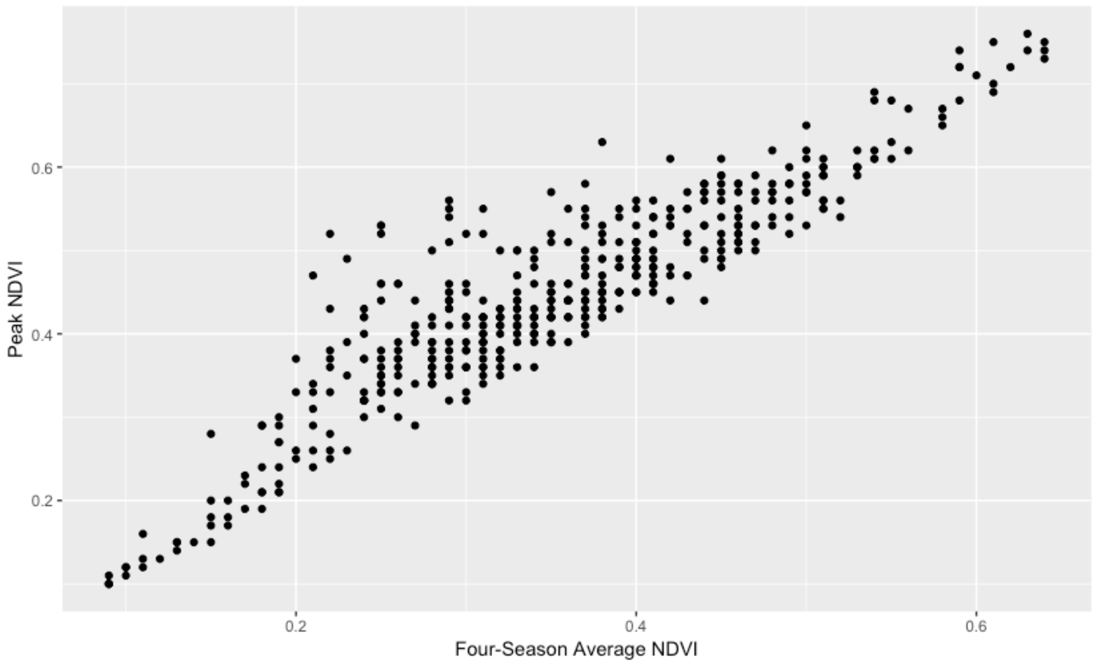

Figure 39: Scatterplot of Peak NDVI versus annual mean NVDI.

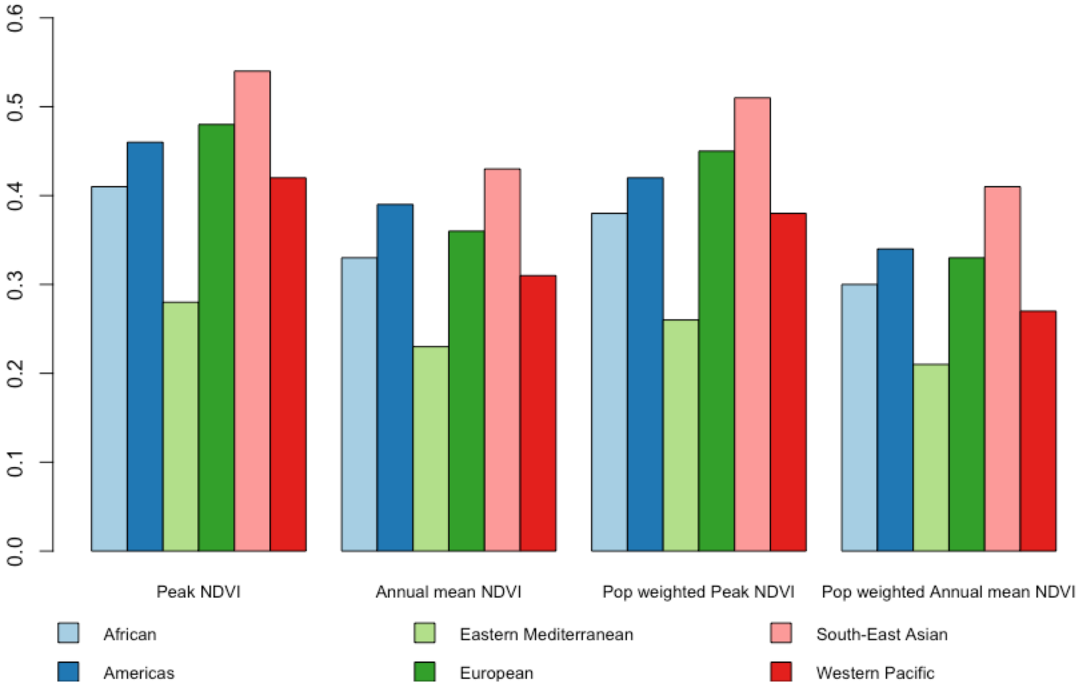

Figure 40: Urban Green Space by WHO Region.

Indicator 2.4: Spending on Adaptation for Health and Health-Related Activities

Methods

The methodology for obtaining the data for this indicator remains the same, two significant changes have been made to the analysis. To present a more cohesive full report, the data for this indicator was converted to USD. Additionally, the definition for health-related spending in non-health sectors was expanded.

The 'Adaptation and Resilience to Climate Change' dataset is the same, annually updated, data source that was used in the 2017-2019 Lancet Countdown reports.[1,7,99] It measures spending on economic activities related to adaptation and resilience to climate change. It was developed by the data research firm kMatrix in partnership with numerous stakeholders.[130] It includes the key adaptation measure identified by the IPCC. This classification of adaptation activities was originally developed through attempts by the UK Department for Environment, Food and Rural Affairs to measure adaptation in 2009/2010. The definition of adaptation activities was extended through collaboration with the Greater London Authority in 2014 and updated through a project with Climate-KIC in 2017. This added several new industrial sectors as well as significantly expanding the activities under health and healthcare.

The methodology used for data acquisition and analysis is based on a system called as 'profiling', which was originally developed at Harvard Business School to track and analyse technical and industrial change.[131] This is the basis for building taxonomies of economic activities and value chains, which can then be populated with estimates of key economic metrics like sales value and employment by triangulating transactional and operational business data to estimate economic values. This methodology is particularly valuable in areas where government statistics and standard industry classifications are not available. When measuring an industry or sector, the new taxonomy is populated from the bottom up, searching for evidence for the ideal definition and including only economic activities where sufficient evidence is available.

For each transaction listed in the adaptation economy data, a minimum of seven separate sources must independently record the transaction for it to be confirmed and included in the database. Triangulating data from multiple sources permits large volumes of unsorted, fragmented data of different types from different sources to be processed to arrive at more accurate estimates of transactional value that would not be possible using a single source. Data is collected from a broad range of sources, which are tracked in time and periodically subjected to both manual and automatised quality checks, where they are scrutinised and updated as necessary. Accessing and analysing multiple types of data is also key to identifying the 'purpose' behind an economic activity, which is key for accurately assigning economic activities to the adaptation dataset. Developing the new definition of adaptation and resilience to climate change involved the top-down taxonomy of the entire 'make and mend' economy, and then adaptation and resilience in all forms. Then these categories were filtered to isolate economic activities that can be strictly identified as being relevant to adaptation and resilience to climate change. The taxonomy of A&RCC is drawn from 11 sectors of the economy at-large: Agriculture & Forestry, Built Environment, Disaster Preparedness, Energy, Health/Health Care, ICT, Natural Environment, Professional Services, Transport, Waste, and Water.[132]

There are a number of activities across different sectors that are 'health-related' in the adaptation and resilience to climate change dataset, outside of the strictly defined healthcare sector. This indicator quantifies spending related to health adaptation in two categories – 1) all spend in health and healthcare sectors; 2) 'health-related' spend in other sectors.

For the 2020 Lancet Countdown report, the definition of health-related spending was developed in consultation with experts in climate change adaptation and health. Health-related spending activities in non-health sectors were identified based on the following definition:

Health-related adaptation spend outside of the health sector is spend that occurs:

In the following sectors: agriculture & forestry, built environment, disaster preparedness, energy, transportation, waste, or water sectors; AND

Directly impacts one or more basic determinant of health: food, water, air or shelter. These correspond closely with "physiological needs" in Maslow's Hierarchy of Needs

Further, spending activities classified as health-related must have an obvious and intuitive relationship to health. A broad definition of shelter is adopted, referring to social interconnectedness, domestic and public dwellings.

Geographical Coverage:

The A&RCC dataset has global coverage for 226 countries and territories. Data has been reported for a subset of countries and territories for whom adaptation spending data, regional and income classifications, and population estimates are available. This year's indicator covers 191 countries and territories with data reported in the A&RCC dataset, and that are assigned a region in the WHO regional classification and an income group in the World Bank income group classification. Per capita values are based on 182 countries with population estimates from the IMF World Economic Outlook October 2019 update.[132]

Data

1. Adaptation and Resilience to Climate Change dataset from kMatrix Ltd, in partnership with University College London.[130]

Caveats

Economic activity or transactions are only measured where there is an economic 'footprint', i.e. where there is transactional/financial data available to be measured. Therefore, public sector spending without an economic 'footprint' (government spending on salaries, for example), cannot be measured. It also not possible to directly identify what percentage of measured spending is public versus private. Values are not currently adjusted for inflation. Values of sales generated are not directly comparable with values derived from national statistics.

The reference period is the financial years 2015/16 to 2018/19. Further historical data could be available in the future.

Additional analysis

At the country level, annual growth in estimated spend varies between 11.3% (Latvia) and 20.56% (United Kingdom) for health adaptation, and between 6.8% (Gambia) and 11.04% (United Kingdom) for health-related adaptation spending. From 2015/16 to 2018/19, health adaptation spending has increased by 35.56%, and health-related adaptation by 19.78%.

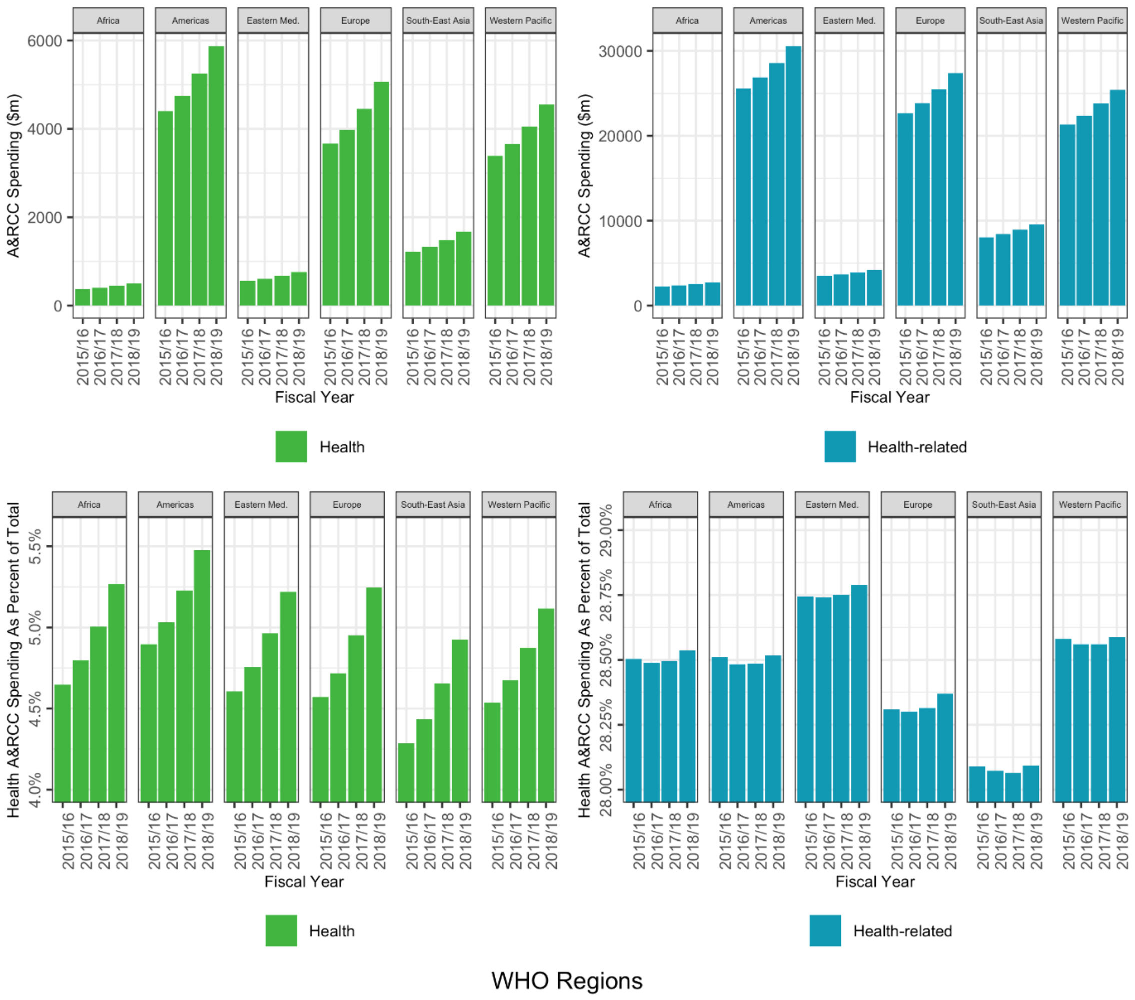

Figure 41: Spending on adaptation for health and health-related activities by WHO region.

Section 3: Mitigation Actions and Health Co-Benefits

3.1: Energy System and Health

Indicator 3.1.1: Carbon Intensity of the Energy System

Methods

This indicator contains two components:

Carbon intensity of the energy system, both at global and regional scales, (1972-2017), in tCO_2/TJ; and

Global CO_2 emissions from energy combustion by fuel, in $GtCO_2$ (1972-2018). Global emissions without fuel breakdown are also provided for 2019.

Technical definition is the tonnes of CO_2 emitted for each unit (TJ) of primary energy supplied.

The rationale for the indicator choice is that carbon intensity of the energy system will provide information on the level of fossil fuel use, which has associated air pollution impacts. Higher intensity values indicate a more fossil dominated system, and one that is likely to have a higher coal share. As countries pursue climate mitigation goals, the carbon intensity is likely to reduce with benefits for air pollution.

The indicator is calculated based on total CO_2 emissions from fossil fuel combustion divided by Total Primary Energy Supply (TPES). TPES reflects the total amount of primary energy used in a specific country, accounting for the flow of energy imports and exports.

The data is available for most countries of the world, for the period 1971-2016.

Data

1. This indicator is based on based on the IEA dataset, CO_2 Emissions From Fuel Combustion: CO_2 Indicators, accessed via the UK data service,[133] and supplemented with additional data for 2018, 2019 and 2020.[134-136]

Caveats

The indicator does not provide information on the share of different fossil fuels, their use in different sectors, and the absolute levels of usage. These are all important elements in understanding the air pollution emissions, and their impacts. Therefore, additional indicators (3.1.2 & 3.1.3) provide additional complimentary information.

Future form of the indicator

This indicator will need to be updated to provide the data for the most recent years, which have seen important shifts in the use of fossil fuels, particularly coal.

Indicator 3.1.2: Coal Phase-Out

Methods

Two indicators are used here:

1. Total primary coal supply by region / country (in exajoules, EJ); and
2. Share of electricity generation from coal (% of total generation from coal) and also global generation from coal (in TWh).

These indicators are important to enable tracking of changes in coal consumption at a regional and country level. Due to the level of coal used for power generation, a second indicator tracks the contribution to electricity generation from coal power plants in selected countries. As countries pursue climate mitigation goals, the use of coal is likely to reduce with resulting benefits for air pollution.

The indicator on primary energy coal supply is an aggregation of all coal types used across all sectors (from the IEA energy balances). The data are available for most countries of the world, for the period 1978-2018.

The indicator on the share of electricity generation from coal is estimated based on electricity generated from coal plant as a percentage of total electricity generated. Regional data are available from 1990-2017, with global share estimated for 2018; pre-1990 data are not used due to incomplete time series.

Countries or regions with large levels of coal use (as a share of generation, or in absolute terms), have been selected to show in the figures.

Data

1. This indicator is based on the extended energy balances from the International Energy Agency. The specific dataset is called World Extended Energy Balances (for 2019), and is sourced via the UK data service.[137]

Caveats

These indicators provide a proxy for air quality emissions associated with the combustion of coal. Further work is required to convert coal use by sector and type into emissions of different air quality pollutants.

Future form of the indicator

As per 3.1.1, this indicator will need to be updated to provide the data for the most recent years, which have seen important shifts in the use of coal.

Additional Analysis

Figure 42 presents TPES of coal, which increased in 2018 by 1.2% overall, driven by a slight increase in use in China, which accounts for 52% of total global coal consumption, and elsewhere in Asia, such as in India (5.5%), Indonesia (9.6%) and Vietnam (18%) and represent an additional 13% of the global total. There has also been a large relative growth in Russia (5.7%) Turkey (8%) and Ukraine (12.3%). Yet, there is an emerging pattern that coal use continues to drop among major users, such as the USA (-4.2%), Japan (-1.2%), Germany (-6%) and Australia (-3.3%).

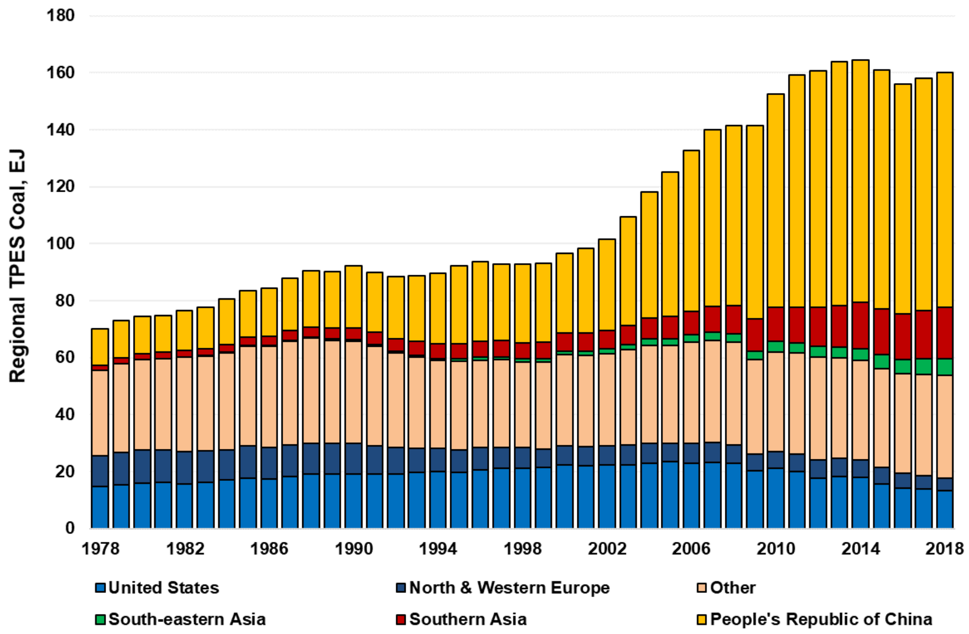

Figure 42: TPES of coal in selected countries and regions, 1978-2018. Regional primary energy supply of coal is shown in bars of Exajoules.

Indicator 3.1.3: Zero-Carbon Emission Electricity

Methods

Two indicators are used here, and presented in two ways:

1. Total low carbon electricity generation, in absolute terms (TWh) and as a % share of total electricity generated (to include nuclear, and all renewables); and
2. Total renewable generation (wind and solar), in TWh, and as a % share of total electricity generated.

The increase in the use of low carbon and renewable energy for electricity generation will push other fossil fuels, such as coal, out of the mix over time, resulting in an improvement in air quality, with benefits to health.

The renewables (wind and solar) indicator has been used to allow for the tracking of rapidly emergent renewable technologies. For both indicators, generation, rather than capacity, has been chosen as a metric as the electricity generated from these technologies is what actually displaces fossil-based generation. Countries with large levels of low carbon generation (as shares, or in absolute terms), or with higher fossil dependency, have been selected.

The data is again taken from the IEA extended energy balances.[138] The absolute level indicators are total gross electricity generated aggregated from the relevant technology types. The share indicators are estimated as the low carbon or renewable generation as a % of total generation.

The data are available for most countries of the world, for the period 1971-2017. Only the period from 1990 has been used, due to data gaps for selected countries prior to 1990.

Data

1. This indicator is based on the extended energy balances from the International Energy Agency. The specific dataset is called World Extended Energy Balances, and is sourced via the UK data service (http://stats.ukdataservice.ac.uk/).[138]

Caveats

This indicator set does not provide information on the air pollutant emissions displaced due to the increasing share of RE generation.

Future form of the indicator

This set should be developed to include an indicator to assess the direct impact on air quality emissions from additional low carbon generation, one approach being to compare the emission intensity of the current system with a counterfactual case, which does not have the additional share of RE generation.

Additional analysis

With the power sector accounting for 38% of total energy-related CO_2 emissions, the importance of renewables for displacing fossil fuels is crucial. In 2016, low carbon electricity globally accounted for 32% of total global electricity, with continued gains in China (see main report). As costs continue to fall, solar generation continues to grow at remarkable rates of around 30% but still only accounts for 2% of total generation.

The types of generation levels from renewables across 1.5°C compliant scenarios are shown in Figure 43. It highlights that generation from new renewables (solar, wind, geothermal, ocean) need to increase by 9.7% per annum, to a level in 2050 that is larger than the total global generation today. Since 1990, the annual growth rate

for these renewables was over 14%. To maintain the momentum in renewable generation growth, there is a need to ensure that all new generation growth is provided for by non-fossil fuel sources, with strong supply side policies to prevent investment in coal and gas.

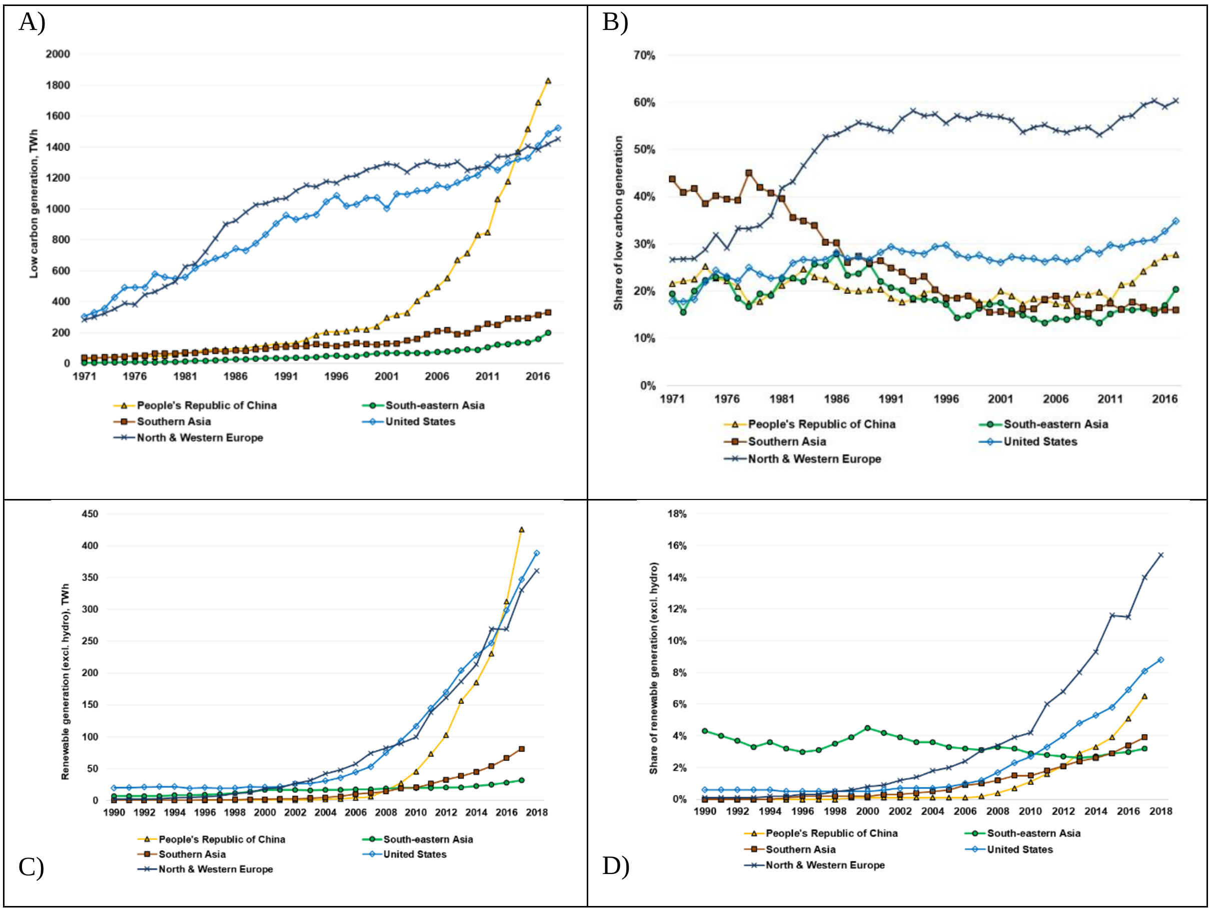

Figure 43: Renewable and low-carbon emission electricity generation. A) Electricity generated from zero carbon sources, TWh; B) Share of electricity generated from zero carbon sources; C) Electricity generated from renewable sources (excl. hydro), TWh; D) Share of electricity generated from renewable sources (excluding hydro).

Indicator 3.2: Clean Household Energy

Methods

The 2020 report presents a combination of data from both the Sustainable Development Goal 7 and fuel consumption in the residential sector produced by the International Energy Agency (IEA).

Access to clean energy is defined by the IEA (2019) as:

"a household having reliable and affordable access to both clean cooking facilities and to electricity, which is enough to supply a basic bundle of energy services initially, and then an increasing level of electricity over time to reach the regional average".[139]

Within SDG 7.1.2 (proportion of population with primary reliance on clean fuels and technology) "Clean" fuels are defined by emission rate targets and specific fuel recommendations included in the WHO guidelines for indoor air quality: household fuel combustion.[140]

This indicator is modelled with household survey data compiled by WHO. Estimates of primary cooking energy for the total, urban and rural population for a given year are obtained separately using a multilevel model done at the country level.[141] The latest data for this indicator was provided by the WHO.[142]

The use of energy in the residential sector is drawn from the IEA extended global residential modelling produced in the World Energy Outlook from the 'World Extended Energy Balances' 2019 edition, which covers all countries or major regions in the world.[137] The values are measured in PJ and cover all fuels consumed within the residential sector final energy demand.

Data from the IEA World Energy Balances on Total Final Consumption of all fuel sources in the Residential sector is taken to calculate the use of 'clean' energy in the residential sector. 'Clean' energy use in this indicator is defined as having no emissions at the point of use for the consumed energy. These 'clean' energy sources are electricity, solar photovoltaic electricity, solar thermal and geothermal heat.

Other measures of 'clean' fuels such as those looking at low-emission levels which would include liquified natural gas (LPG) and natural gas, were not used in this analysis for the reason that they additionally directly emit GHG emissions at point of use.

The specific IEA variables were combined in the following way:

Low-emission fuels consumed of total demand = "*Electricity*" + "*Natural Gas*"

Zero emission energy consumed = "*Geothermal*" + "*Solar/wind/other*" + "*Electricity*"

Solid biofuels consumed = "*Biofuels and waste*"

Official reporting of SDG 7 relies on the primary fuel and technology used for cooking in the home alone as a proxy indicator for measuring "access" to clean household energy. The data used in this 2020 report focus more on the amount of 'healthy' energy consumed at the household level. Estimating the actual energy used in the household serves as a measure of action to achieving the intent of SDG 7.1.2. The data is summarized for regions across the globe.[138]

A simple box model was used to estimate indoor domestic air pollution exposures from indoor cooking and infiltrating outdoor air pollution.

GAINS data for 2018 informed both outdoor (ambient air pollution and fraction from domestic sources) and indoor (national levels of clean or solid fuel use for cooking, stove types, and their $PM_{2.5}$ emission factors) sources of air pollution.

The infiltration of outdoor and removal of indoor air pollution within the box was estimated using typical dwelling air change rates and required extract ventilation, obtained by reviewing previous studies, accounting for differences across formal and informal housing and climate regions of the modelled countries. The estimate housing modification of air pollution exposure was then taken to be the time and population-weighted indoor exposure, with the counterfactual being ambient levels minus the domestic fraction.

Attributable premature mortality was estimated by calculating population attributable fractions for each country by applying GBD integrated exposure response (IER) functions for long-term $PM_{2.5}$ exposure to GBD estimates of annual deaths (2017) for five causes of mortality (chronic obstructive pulmonary disease, ischaemic heart disease, lower respiratory infections, stroke, lung cancer). The estimates were converted to mortality per million population using data from the UN's World Population Prospects 2017 Revision.

Data

1. Healthy fuels for cooking were provided by the WHO.[142]
2. The additional energy usage and access is based on data from the IEA World Energy Balances 2020.[138]
3. Demographic data from the United Nation World Population Prospects (UN WPP).[143]
4. IER functions and mortality rates were taken from the GBD Project.[144]

Caveats

The data from the IEA on residential energy flows and energy access provide an indication of both the access to electricity and the proportion of the different types of energy used within the residential sector. These provide an important picture on how access and use might be interacting.

Future form of the indicator

This indicator provides a representation of the fuel mix used by households for different demands (heating, cooling, cooking, hot water, lighting and other plug loads) for the mix of income groupings at the country level.

The indicator estimating household air pollution exposure and premature mortality per country is under development and will be presented as a standalone indicator in the 2021 report.

Indicator 3.3: Premature Mortality from Ambient Air Pollution by Sector

Methods

This indicator quantifies contributions of individual source sectors to ambient $PM_{2.5}$ exposure and its health impacts. Contributions from different fuels, in particular coal, have been highlighted across all sectors.

Estimates of sectoral source contributions to annual mean exposure to ambient $PM_{2.5}$ were calculated using the GAINS model,[145] which combines bottom-up emission calculations with atmospheric chemistry and dispersion coefficients.

Energy statistics are taken from the IEA World Energy Statistics and the IEA World Energy Outlook 2019,[134] merged with GAINS information on application of emission control technologies and their emission factors.

Atmospheric transfer coefficients are based on full year simulations with the EMEP Chemistry Transport Model[146] at 0.1°×0.1° resolution using meteorology of 2015. Calculations for Europe are described in detail by Kiesewetter et al. (2015)[147] and calculations for the rest of the world are described by Amann et al., (2020).[148] Calculated ambient $PM_{2.5}$ concentrations have been validated against in-situ observations from the latest version of the WHO's Urban Ambient Air Pollution Database (2018 update),[149] and other sources where available (e.g. Chinese statistical yearbook).

Premature deaths from total ambient $PM_{2.5}$ for regions other than Europe are calculated using the methodology of the WHO (2016) assessment on the burden of disease from ambient air pollution,[150] which relies on disease specific integrated exposure response relationships (IERs) developed within the Global Burden of Disease 2013 study.[151] Disease and age specific baseline mortality rates are taken from the GBD Results database.[152] The shares of different diseases to age-specific total deaths taken from UN World Population Prospects (2017 update);[143] for 2018, the statistics were interpolated linearly between 2015 and 2020. For Europe, this indicator follows the WHO Europe methodology and apply Exposure-response relationships for all-cause mortality among population over 30 years of age as reported under the REVIHAAP assessment (WHO, 2013).[153] Details are described in Kiesewetter et al. (2015).[147]

Attribution of estimated premature deaths from AAP to polluting sectors was done proportional to the contributions of individual sectors to population-weighted mean $PM_{2.5}$ in each country.

To demonstrate the relevance of demographic changes compared to emission changes, two variants of the indicator were calculated. The benchmark indicator includes the differences in mortality year. To show the changes of $PM_{2.5}$ concentrations only, the indicator figure also includes a variant calculated with constant mortality year 2015. This allows a better comparison with Lancet Countdown 2019 results, where the population year was fixed to 2015.

For technical reasons, there are three deviations in the aggregation of countries versus the WHO regions:

- Sudan and Somalia are included in the 'African Region' here, but belong to WHO Eastern Mediterranean Region.
- Algeria is included in the 'Eastern Mediterranean' here, but belongs to WHO African Region.

Data

1. Energy statistics are taken from the IEA World Energy Statistics (for 2015) and the World Energy Outlook 2019 and combined with information from the GAINS model database on application rates of emission control technologies and their emission factors for all precursor pollutants of $PM_{2.5}$.
2. Exposure-response relationships for all-cause mortality among populations in Europe over 30 years are taken from the REVIHAAP assessment.[153]
3. Calculations for premature deaths from total ambient $PM_{2.5}$ for other regions follow the methodology of the WHO global assessment on the burden of disease from ambient air pollution.[150]
4. Total age-specific deaths in individual years are taken from the UN World Population prospects 2017.[143]
5. Contributions of individual diseases to age-specific deaths are taken from the Global Burden of Disease 2013 assessment (for year 2010)[151]

Caveats

The indicator relies on model calculations which are inherently uncertain. The resolution of approximately 7 to 10 km is deemed appropriate for urban background levels of $PM_{2.5}$ but may underestimate exposure in case of strong local $PM_{2.5}$ increments. The meteorology year is fixed to 2015.

Uncertainty in the shape of integrated exposure-response relationships (IERs) make the quantification of health burden inherently uncertain.

Different dose-response relationships are used for Europe (REVIHAAP, recommended by WHO-Europe) and Asia (WHO-Global).

The non-linearity of the IERs used for non-European countries complicates the translation between the mortality burden attributed to an individual source, which is calculated proportional to the source contribution to ambient $PM_{2.5}$, and the effect of mitigating this source. While a reduction of emissions would lead to a (roughly) proportional reduction of ambient $PM_{2.5}$, this would not necessarily result in a proportional reduction of the health burden. In highly polluted environments, the health benefits of a marginal reduction of emissions would be disproportionately smaller than the relative change in concentrations.

Future form of the indicator

Other health indicators than premature deaths should be included for a more complete assessment of the health burden, particularly Years of Life Lost (YLLs) and Years Lived with Disability (YLDs).

An ideal indicator would provide a marker of benefits for air quality and/or health that are directly attributable to climate change mitigation action, which requires scenario analysis.

Indicator 3.4: Sustainable and Healthy Transport

Methods

Fuel use data (by fuel type) from the IEA datasets are divided by corresponding population statistics from the World Bank.

Data

1. Fuel use data is from the IEA (2019) Global EV Outlook 2019: Scaling up the transition to electric mobility.[154]

Caveats

This indicator captures change in total fuel use and type of fuel use for transport, but it does not capture shifts in modes of transport used. In particular, it does not capture walking and cycling for short trips, which can yield substantial health benefits through increased physical activity.[155]

Future form of the indicator

An ideal fuel use indicator would capture the direct health impacts of the use of transport fuels, with country- and urban-level specificity within the global coverage. In turn, the co-benefits of transitioning to less-polluting fuels would be quantified directly in terms of reduced exposures to air pollution and their corresponding health impact.

To more fully capture sustainable uptake a future indicator could collate information on the proportion of total distance travelled by different modes of transport based on comprehensive local survey data. Other data on sustainable travel infrastructure, for instance the presence of cycle schemes, would also be useful.

3.5: Food, Agriculture, and Health

Indicator 3.5.1: Emissions from Agricultural Production and Consumption

Methods

For livestock:

The following livestock are included

Ruminant	Non Ruminant
Cattle, dairy<br><br>(FAO Item Code 960)	Chicken, broilers<br><br>(FAO Item Code 1053)
Cattle, non-dairy<br><br>(FAO Item Code 961)	Chicken, layers<br><br>(FAO Item Code 1052)
Buffaloes<br><br>(FAO Item Code 946)	Swine, market<br><br>(FAO Item Code 1049)
Goats<br><br>(FAO Item Code 1016)	Swine, breeding<br><br>(FAO Item Code 1079)
Sheep<br><br>(FAO Item Code 976)	

Emissions from enteric fermentation, manure management and manure left on pasture are obtained from Herrero et al.[156] his information is presented in tonne carbon dioxide equivalent (CO2e) per tropical livestock unit (tlu), which is converted to livestock head using the table below. The emissions per head were multiplied by the number of animals per country obtained from the FAO database to calculate the total emissions per livestock type per country.

	Head per tlu
Bovine (Buffalo, Cattle (dairy), Cattle (non-dairy)	1.43
Small Ruminants (Goats, Sheep)	10
Poultry (Chicken)	100
Swine	5

The emissions per head are divided into world regions (as in the GLOBIOM model) and, for ruminants, livestock system. To convert to country values, a weighted average of the livestock numbers in all regions is taken.

To obtain the emissions from grazing, the fertilizer applied to grassland from Chang et al.[157] is used.

For Crops:

The emissions from fertilizer (synthetic and manure applied), rice cultivation and cultivation from organic land for maize, rice, wheat, soybean and other crops for the year 2000 are obtained from Carlson et al.,[158] which use IPCC methodology and a non-linear N2O emission model.

Data from the FAO or emissions from fertilizer (synthetic and manure), rice cultivation and cultivation from organic land was obtained from 2000-2017.[159] The rate of increase/decrease for the years 2001-2017 in relation to 2000 are calculated. This rate is then applied to the data derived from Carlson et al. to obtain values from 2000-2017.[158]

The GHG emissions associated with agrciultural commodity consumption uses FAO production and trade data to estimate the total GHG emissions footprint associated with each of comoodoties condisdered in a given country. This method is used by Dalin et al.[160] for tracing water consumption in global food networks but is adapted here to calculated GHG footprint. The basic equation the indiciator follows is

Consumption = production + imports - exports

All crop-related GHG emissions (including feed crops) are considered in the crop accounts, and the animal GHG emissions only have the enteric fermentation, manure management and grassland-related emissions (in line with indicator 3.5.1).

FAO production and trade data are used in the following manner. For a given commodity the national production values in tonnes are converted into CO_2e values using the carbon intensity values supplied by indicator 3.5.1 GHG production estimates (via Carlson et al.) associated with producing that tonnage of the commodity. Next, secondary commodities are converted in primary equivalent values by multiplying the trade tonnage by the value derived from Dalin et al.[160] For example, the primary equivalences for wheat products are as follows:

Bran, wheat	1.01
Bread	0.88
Bulgur	1.05
Cereals, breakfast	1.18
Flour, wheat	1.01
Macaroni	1.01
Pastry	0.88
Wafers	0.88
Wheat	1.00

These values are then converted into GHG emissions equivalent, based on the carbon intensity. For a given year, the trade balances are corrected to take into account that a given commodity may have been produced in one country, processed in another and finally imported into a third, using an algorithm developed by Kastner et al 2011.[161]

Data

1. National annual production of animal products items (tonnes) – FAOSTAT.[159]
2. National annual trade (country-country) of animal products items (tonnes) – FAOSTAT.[159]
3. Correspondence of items across item lists with different grouping – FAOSTAT.[159]
4. GHG emissions intensity per country of animal products – provided by LC 3.7 GHG production estimates (via Herrero et al. 2013)[156] Definitions: Animal types: bovine cattle (beef and buffalo), sheep and goat ruminants, pigs, poultry (chicken, ducks, geese and turkeys).
5. National annual production of crops (tonnes) – FAOSTAT.[159]
6. National annual trade (country-country) of crop products (tonnes) – FAOSTAT.[159]
7. Carbon intensity of animal products for each country– provided by Carlson et al. (2017).[158]

Caveats

For livestock, data on stock numbers has been abstracted from FAO database, however, some data is missing for some years, most notably Somalia (missing data 2000-2011) for non-dairy cattle. Data on grazing emissions from small islands is also missing.

The emission factors differ from FAO numbers:

- For livestock, this is due to calculation of emissions of enteric fermentation, manure management and manure left on pasture at Globiom region (n=29) and livestock system (n=8) level whereas the FAO use subcontinental (n=9) and climatic level (n=3).[159]

- For crops, this is due to the FAO assuming slightly higher synthetic N application, greater manure N inputs, and a linear emissions factor of 1%, in contrast to a mean of 0.77% used by the non-linear model of Carlson et al. (2017).[158]

Agricultural consumption emissions estimates are derived directly from FAO trade values, as described above. Therefore, these values differ from the production estimates, which are based on extrapolating year 2000 figures. On average across all years, the estimate of total emissions due to consumption are 2.25% above production values, and do not differ by more than 10% in any given year. The sole exception to this is the estimates of the differences between production and consumption by WHO region shown in the figure in the main text. For this figure the production values are derived directly from FAO values.

Future form of the Indicator

In future years, consumption and production emissions will both be estimated by using FAO trade values. Efforts will also be made to estimate waste emissions.

Additional analysis

Total global crop and livestock consumption emissions are given in Figure 44 and Figure 46 below.

The 2018 report of the Lancet Countdown emphasised the complexity of developing a standard indicator for sustainable diets.[7] It acknowledged the limited dietary data availability and the context-dependent nature of what diets can and should be considered sustainable in different high and low-income countries. The EAT-Lancet commission report, published in early 2019, further advanced the debate on defining sustainable and healthy diets and global targets for their achievement.[162] The EAT-Lancet commission proposed an integrated framework based on considerations of acceptable limits to food system influence on six environmental processes (climate change, land system change, freshwater use, biodiversity loss, and interference with global nitrogen and phosphorous cycles) and evidence on dietary health impacts. They elaborated on the disproportionately negative environmental effects of ruminant meat production and the negative health implications of red meat consumption in high-income populations.

The overall emissions from livestock has increased by 16% from 2000 to 2017. Enteric fermentation (68%) has the highest contribution to total livestock emissions, followed by manure management (17%), manure left on pasture (14%) and grassland fertilizer (1%). The majority of the temporal increase in emissions is attributed to manure left on pasture, enteric fermentation and manure management which have increased by 19%, 16% and 13% respectively from 2000 to 2017, whereas the emissions from grassland fertilizer has only increased by 2%.

As ruminants undergo enteric fermentation they have the highest emissions of all livestock (93% of total). This is split between non-dairy cattle (67%), followed by dairy cattle (13%), goats and sheep (12%) and buffalo (9%). Emissions from non-ruminants are divided between pigs (5%) and poultry (1%). The largest increase in emissions from 2000 to 2017 was poultry (58%), followed by dairy cattle (30%), small ruminant (26%), buffalo (23%), non-dairy (11%) and pigs (10%).

The overall emissions from crops has increased by 12% from 2000 to 2017. Fertilizer (24%) has the lowest contribution to total crop emissions, followed by cultivation of organic soils (28%) and rice cultivation (48%). The majority of the temporal increase in emissions is attributed to emissions from fertilizer which has increased by 29% from 2000 to 2017, whereas the emissions from rice and organic soil cultivation have only increased by 7% and 10% respectively.

As rice produces methane in addition to fertilizer application, it has the highest emissions of all crops (53% of total), followed by wheat (7%), maize (5%) and soybean (1%). The largest increase in emissions from 2000 to 2017 is attributed to wheat and maize (19%), followed by soybean (13%) whereas emissions from rice have only increased by 8%. The majority of the increases are due to fertilizer emissions which have increased by between 25 and 36% where emissions from cultivation of organic soils have only increased by between 0 and 2% for the named crops.

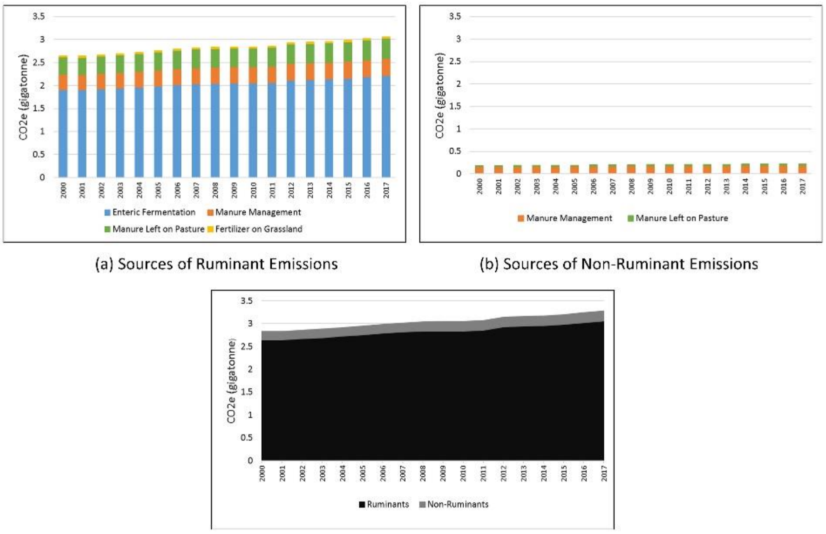

(a) Sources of Ruminant Emissions

(b) Sources of Non-Ruminant Emissions

(c) Total Livestock Emissions

Figure 44: GHG emissions from livestock. a) Sources of total ruminant emissions; b) Sources of total non ruminant emissions; c) Total livestock emissions.

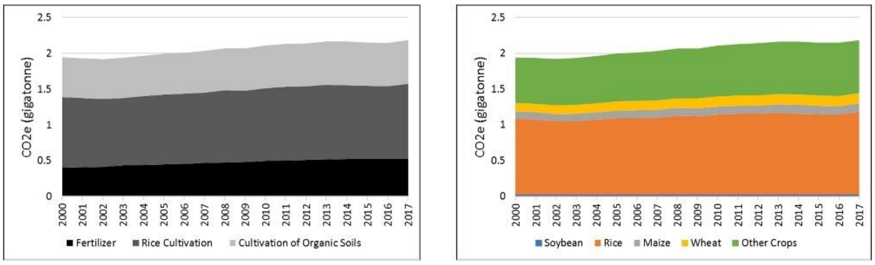

(a) Total Emissions of Crops Divided by Emissions Source

(b) Total Emissions of Crops Divided by Crop Type

Figure 45: GHG emissions from crops. a) Total emissions of crops by emissions source. b) Total emissions of crops by crop type.

For crops, main crops comprise wheat rice and maize, and each incorporate as many secondary commodities as were available. Other crops comprise barley, beans (dry and green), broad beans and horse beans, cassava, chickpeas, cotton, groundnuts, millet, mustard, oats, peas (dry and green), potatoes, rapeseed, rye, sesame seed, sorghum, soybeans, sweet potatoes, oil palm fruit, sugarcane and sugar beet, yams.

For livestock, all categories also include secondary products (such as cheese in the case of milk) where data was available. Cattle products comprise beef meat and milk and buffalo meat and milk. Sheep and goat products comprise meat and milk. Poultry products comprise meat and eggs of chickens, geese, ducks and turkeys. Pig products include secondary processed commodities such as ham and bacon.

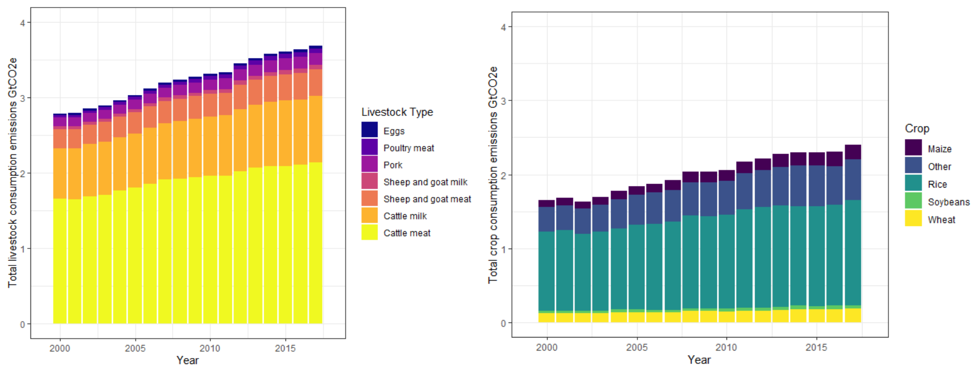

Figure 46: Total global agricultural consumption emissions from livestock and crops 2000-2017.

Indicator 3.5.2: Diet and Health Co-Benefits

Methods

Baseline consumption data

Baseline food consumption was estimated by adopting estimates of food availability from the FAO's food balance sheets, and adjusting those for the amount of food wasted at the point of consumption.[74,163,164] An alternative would have been to rely on a set of consumption estimates that have been based on a variety of data sources, including dietary surveys, household budget and expenditure surveys, and food availability data.[165,166] However, neither the exact combination of these data sources, nor the estimation model used to derive the data have been made publicly available. For some individual countries, using dietary surveys would also have been an alternative. However, underreporting is a persistent problem in dietary survey,[167,168] and regional differences in survey methods would have meant that results would not be comparable between countries. In contrast to dietary surveys, waste-adjusted food-availability estimates indicate levels of energy intake per region that reflect differences in the prevalence of overweight and obesity across regions.[169]

Food balance sheets report on the amount of food that is available for human consumption.[163] They reflect the quantities reaching the consumer, but do not include waste from both edible and inedible parts of the food commodity occurring in the household. As such, the amount of food actually consumed may be lower than the quantity shown in the food balance sheet, depending on the degree of losses of edible food in the household, e.g. during storage, in preparation and cooking, as plate-waste, or quantities fed to domestic animals and pets, or thrown away.

The waste-accounting methodology developed by the FAO was followed to account for the amount of food wasted at the household level that was not accounted for in food availability estimates.[164] Table 6 provides and overview of the parameters used in the calculation.

Table 6: Percentage of food wasted during consumption (cns), and percentage of processed utilisation (pctprcd). The percentage of fresh utilisation is calculated as 1-pctprcd. Conversion factors to edible portions of foods are provided below the table

Food group	Item	Region						
		Europe	USA, Canada, Oceania	Indus-trialized Asia	Sub-Saharan Africa	North Africa, West and Central Asia	South and Southeast Asia	Latin America
cereals	wp(cns)	25	27	20	1	12	3	10
roots and tuber	pct_{prcd}	73	73	15	50	19	10	80
	wp(cns)	17	30	10	2	6	3	4
	wp(cns_{prcd})	12	12	12	1	3	5	2
oilseeds and pulses	cns	4	4	4	1	2	1	2
fruits and vegetables	pct_{prcd}	60	60	4	1	50	5	50
	wp(cns)	19	28	15	5	12	7	10
	wp(cns_{prcd})	15	10	8	1	1	1	1
milk and dairy	wp(cns)	7	15	5	0.1	2	1	4
eggs	wp(cns)	8	15	5	1	12	2	4
meat	wp(cns)	11	11	8	2	8	4	6
fish and seafood	pct_{prcd}	*40% for low-income countries, and 96% for all others.*						
	wp(cns)	11	33	8	2	4	2	4
	wp(cns_{prcd})	10	10	7	1	2	1	2

Conversion factors: maize, millet, sorghum: 0.69; wheat, rye, other grains: 0.78; rice: 1; roots: 0.74 (0.9 for industrial processing); nuts and seeds: 0.79; oils: 1; vegetables: 0.8 (0.75 for industrial processing); fruits: 0.8 (0.75 for industrial processing); beef: 0.715; lamb: 0.71; pork: 0.68; poultry: 0.71; other meat: 0.7; milk and dairy: 1; fish and seafood: 0.5; other crops: 0.78

For each commodity and region, food consumption was estimated by multiplying food availability data with conversion factors (*cf*) that represent the amount of edible food (e.g. after peeling) and with the percentage of food wasted during consumption (*1-wp(cns)*). For roots and tubers, fruits and vegetables, and fish and seafood, the differences in wastage between the proportion that is utilised fresh (pct_{frsh}) and the proportion that utilised in processed form (pct_{prcd}) were also accounted for. The equation used for each food commodity and region was:

$$Consumption = Availability \cdot \frac{pct_{frsh}}{100} \cdot cf_{frsh} \cdot \left(1 - \frac{wp(cns_{frsh})}{100}\right)$$

$$+ Availability \cdot \frac{pct_{prcd}}{100} \cdot cf_{prcd} \cdot \left(1 - \frac{wp(cns_{prcd})}{100}\right)$$

Comparative risk assessment

The mortality and disease burden attributable to dietary and weight-related risk factors was estimated by calculating population impact fractions (PIFs) which represent the proportions of disease cases that would be avoided when the risk exposure was changed from a baseline situation to a counterfactual situation. For calculating PIFs, the general formula was used:[170-172]

$$PIF = \frac{\int RR(x)P(x)dx - \int RR(x)P'(x)dx}{\int RR(x)P(x)dx}$$

Where $RR(x)$ is the relative risk of disease for risk factor level x, $P(x)$ is the number of people in the population with risk factor level x in the baseline scenario, and $P'(x)$ is the number of people in the population with risk factor level x in the counterfactual scenario. Changes in relative risks were assumed to follow a dose-response relationship,[171] and that PIFs combine multiplicatively, i.e. $PIF = 1 - \prod_i(1 - PIF_i)$ where the i's denote independent risk factors.[171,173]

The number of avoided deaths due to the change in risk exposure of risk *i*, $\Delta deaths_i$, was calculated by multiplying the associated PIF by disease-specific death rates, *DR*, and by the number of people alive within a population, *P*:

$$\Delta deaths_i(r,a,d) = PIF_i(r,d) \cdot DR(r,a,d) \cdot P(r,a)$$

Where PIFs are differentiated by region *r* and disease/cause of death *d*; the death rates are differentiated by region, age group *a*, and disease; the population groups are differentiated by region and age group; and the change in the number of deaths is differentiated by region, age group and disease.

Publicly available data sources were used to parameterise the comparative risk analysis. Mortality and population data were adopted from the Global Burden of Disease project.[174] Baseline data on the weight distribution in each country were adopted from a pooled analysis of population-based measurements undertaken by the NCD Risk Factor Collaboration.[169]

The relative risk estimates that relate the risk factors to the disease endpoints were adopted from meta-analyses of prospective cohort studies for dietary weight-related risks.[175-182] In line with the meta-analyses, non-linear dose-response relationships for fruits and vegetables, nuts and seeds, and fish, were included and it was assumed linear dose-response relationships for the remaining risk factors. As the analysis was primarily focused on mortality from chronic diseases, adults aged 20 year or older were included, and the relative-risk estimates were adjusted for attenuation with age based on a pooled analysis of cohort studies focussed on metabolic risk factors,[183] in line with other assessments.[151,184]

Table 7: Relative risk parameters (mean and low and high values of 95% confidence intervals) for dietary risks and weight-related risks

Food group	Endpoint	Unit	RR mean	RR low	RR high	Reference
Red meat	CHD	100 g/d	1.15	1.08	1.23	Bechthold et al (2019)
	Stroke	100 g/d	1.12	1.06	1.17	Bechthold et al (2019)
	Colorectal cancer	100 g/d	1.12	1.06	1.19	Schwingshackl et al (2018)
	Type 2 diabetes	100 g/d	1.17	1.08	1.26	Schwingshackl et al (2017)
Fish	CHD	15 g/d	0.94	0.90	0.98	Zheng et al (2012)
Fruits	CHD	100 g/d	0.95	0.92	0.99	Aune et al (2017)
	Stroke	100 g/d	0.77	0.70	0.84	Aune et al (2017)
	Cancer	100 g/d	0.94	0.91	0.97	Aune et al (2017)
Vegetables	CHD	100 g/d	0.84	0.80	0.88	Aune et al (2017)
	Cancer	100 g/d	0.93	0.91	0.95	Aune et al (2017)
Legumes	CHD	57 g/d	0.86	0.78	0.94	Afshin et al (2014)
Nuts	CHD	28 g/d	0.71	0.63	0.80	Aune et al (2016)
	Cancer	28 g/d	0.85	0.76	0.94	Aune et al (2016)
	Type 2 diabetes	28 g/d	0.61	0.43	0.88	Aune et al (2016)
Underweight	CHD	15<BMI<18.5	1.17	1.09	1.24	Global BMI Collab (2016)
	Stroke	15<BMI<18.5	1.37	1.23	1.53	Global BMI Collab (2016)
	Cancer	15<BMI<18.5	1.10	1.05	1.16	Global BMI Collab (2016)
	Respiratory disease	15<BMI<18.5	2.73	2.31	3.23	Global BMI Collab (2016)
Overweight	CHD	25<BMI<30	1.34	1.32	1.35	Global BMI Collab (2016)
	Stroke	25<BMI<30	1.11	1.09	1.14	Global BMI Collab (2016)
	Cancer	25<BMI<30	1.10	1.09	1.12	Global BMI Collab (2016)
	Respiratory disease	25<BMI<30	0.90	0.87	0.94	Global BMI Collab (2016)
	Type 2 diabetes	25<BMI<30	1.88	1.56	2.11	Prosp Studies Collab (2009)
Obesity (grade 1)	CHD	30<BMI<35	2.02	1.91	2.13	Global BMI Collab (2016)
	Stroke	30<BMI<35	1.46	1.39	1.54	Global BMI Collab (2016)
	Cancer	30<BMI<35	1.31	1.28	1.34	Global BMI Collab (2016)
	Respiratory disease	30<BMI<35	1.16	1.08	1.24	Global BMI Collab (2016)
	Type 2 diabetes	30<BMI<35	3.53	2.43	4.45	Prosp Studies Collab (2009)
Obesity (grade 2)	CHD	30<BMI<35	2.81	2.63	3.01	Global BMI Collab (2016)
	Stroke	30<BMI<35	2.11	1.93	2.30	Global BMI Collab (2016)
	Cancer	30<BMI<35	1.57	1.50	1.63	Global BMI Collab (2016)
	Respiratory disease	30<BMI<35	1.79	1.60	1.99	Global BMI Collab (2016)
	Type 2 diabetes	30<BMI<35	6.64	3.80	9.39	Prosp Studies Collab (2009)
Obesity (grade 3)	CHD	30<BMI<35	3.81	3.47	4.17	Global BMI Collab (2016)
	Stroke	30<BMI<35	2.33	2.05	2.65	Global BMI Collab (2016)
	Cancer	30<BMI<35	1.96	1.83	2.09	Global BMI Collab (2016)
	Respiratory disease	30<BMI<35	2.85	2.43	3.34	Global BMI Collab (2016)
	Type 2 diabetes	30<BMI<35	12.49	5.92	19.82	Prosp Studies Collab (2009)

Table 8 provides an overview of the relative-risk parameters used. For the counterfactual scenario, minimal risk exposure levels (TMRELs) were defined as follows: 300 g/d for fruits, 500 g/d for vegetables, 100 g/d for legumes, 20 g/d for nuts and seeds, 50 g/d for fish, 0 g/d for red meat, and no underweight, overweight, or obesity. The TMRELs are in line with those defined by the Nutrition and Chronic Diseases Expert Group (NutriCoDE),[184] with the exception that a higher value for vegetables was used, and zero as minimal risk exposure for red meat was used, in each case based on a more comprehensive meta-analyses.[176]

Table 8: Overview of existing ratings on the certainty of evidence for a statistically significant association between a risk factor and a disease endpoint. The ratings include those of the Nutrition and Chronic Diseases Expert Group (NutriCoDE),[184] the World Cancer Research Fund,[185] and NutriGrade.[179-181] The ratings relate to the risk-disease associations in general, and not to the specific relative-risk factor used for those associations in this analysis.

Food group	Endpoint	Association	Certainty of evidence
Fruits	CHD	reduction	NutriCoDE: probable or convincing;<br>NutriGrade: moderate quality of meta-evidence
	Stroke	reduction	NutriCoDE: probable or convincing<br>NutriGrade: moderate quality of meta-evidence
	Cancer	reduction	WCRF: strong evidence (probable) for some cancers<br>NutriGrade: moderate quality of meta-evidence for colorectal cancer
Vegetables	CHD	reduction	NutriCoDE: probable or convincing<br>NutriGrade: moderate quality of meta-evidence
	Cancer	reduction	WCRF: strong evidence (probable) for non-starchy vegetables and some cancers<br>NutriGrade: moderate quality of meta-evidence for colorectal cancer
Legumes	CHD	reduction	NutriCoDE: probable or convincing<br>NutriGrade: moderate quality of meta-evidence
Nuts and seeds	CHD	reduction	NutriCoDE: probable or convincing<br>NutriGrade: moderate quality of meta-evidence
Fish	CHD	reduction	NutriCoDE: probable or convincing<br>NutriGrade: moderate quality of meta-evidence
Red meat	CHD	increase	NutriGrade: moderate quality of meta-evidence
	Stroke	increase	NutriGrade: moderate quality of meta-evidence
	Cancer	increase	WCRF: strong evidence (probable) for colorectal cancer<br>NutriGrade: moderate quality of meta-evidence for colorectal cancer
	Type-2 diabetes	increase	NutriCoDE: probable or convincing<br>NutriGrade: high quality of meta-evidence

NutriCoDE: Nutrition and Chronic Diseases Expert Group
NutriGrade: Grading of Recommendations Assessment, Development, and Evaluation (GRADE) tailored to nutrition research
WCRF: World Cancer Research Fund

The selection of risk-disease associations used in the health analysis was supported by available criteria used to judge the certainty of evidence, such as the Bradford-Hill criteria used by the Nutrition and Chronic Diseases Expert Group (NutriCoDE),[184] the World-Cancer-Research-Fund criteria used by the Global Burden of Disease project,[71] as well as NutriGrade.[186] The latter was used in a series of meta-analyses that graded the quality of evidence of a set of risk-disease associations (Table 8).[179-181] The quality of evidence for most of the risk-disease associations included in the analysis was graded as moderate to high, but it was graded as low to very low for two of the three disease associations for nuts.[179-181] However, a more detailed meta-analysis was used for those associations than the one that was graded.[177] All available risk-disease associations that were graded as having moderate quality of evidence and showed statistically significant results in the meta-analyses that included NutriGrade assessments were not included, because for some associations more detailed meta-analyses (with more sensitivity analyses) were available that indicated potential confounding with other major dietary risks. Such sensitivity analyses were not presented in the meta-analyses that included NutriGrade assessments, but they are important for health assessments that evaluate changes in multiple risk factors.

For the different diet scenarios, uncertainty intervals were calculated, associated with changes in mortality based on standard methods of error propagation and the confidence intervals of the relative risk parameters. For the error propagation, the error distribution of the relative risks was approximated by a normal distribution and used that side of deviations from the mean which was largest. This method leads to conservative and potentially larger uncertainty intervals as probabilistic methods, such as Monte Carlo sampling, but it has significant computational advantages, and is justified for the magnitude of errors dealt with here (<50%) (see e.g. IPCC Uncertainty Guidelines).

Data

Type	Coverage	Source
Exposure data:		
Food consumption data	Country-level	Food availability data adjusted for food waste at the household level.[74,164] Estimates of energy intake were in line with trends in body weight across countries.[169]
Weight estimates	Country-level	Baseline data from pooled analysis of measurement studies with global coverage.[169]
Health analysis:		
Relative risk estimates	General	Adopted from meta-analysis of prospective cohort studies. The certainty of evidence for the risk-disease associations were rated as moderate to high by NutriGrade.[179-181]
Mortality and population data	Country-level	Adopted from the Global Burden of Disease project by country and age group.[144]

Caveats

In the comparative risk assessment, relative risk factors that are subject to the caveats common in nutritional epidemiology were used, including small effect sizes and potential measurement error of dietary exposure, such as over and underreporting and infrequent assessment.[187] For these calculations, it was assumed that the risk-disease relationships describe causal associations, an assumption supported by the existence of statistically significant dose-response relationships in meta-analyses, the existence of plausible biological pathways, and supporting evidence from experiments, e.g. on intermediate risk factors.[175-177,179-182,184,188-190] However, residual confounding with unaccounted risk factors cannot be ruled out in epidemiological studies. Additional aspects rarely considered in meta-analyses are the importance of substitution between food groups that are associated with risks, and the time lag between dietary exposure and disease.

To address potential confounding, risk-disease associations that became non-significant in fully adjusted models were omitted, in particular those related to milk intake,[191,192] but potential confounding might also exists for the association between increased fish intake and reduced CHD risk.[193-195] The quality of evidence in meta-analyses that covered the same risk-disease associations as used here was graded with NutriGrade as moderate or high for all risk-disease pairs included in the analysis (Table 8).[179-181] In addition, the Nutrition and Chronic Diseases Expert Group and the World Cancer Research Fund graded the evidence for a causal association of ten of the 12 risk-disease associations included in the analysis as probable or convincing,[184,185] The relative health ranking of leading risk factors found in our analysis was similar to existing rankings that relied on different relative-risk parameters and exposure data.[71,196]

A proxy of food consumption that was derived from estimates of food availability that were adjusted for the amount of food wasted at the point of consumption was used as exposure data.[163,164] An alternative would have been to rely on a set of consumption estimates that has been based on a variety of data sources, including dietary surveys, household budget and expenditure surveys, and food availability data.[165,166] However, neither the exact combination of these data sources, nor the estimation model used to derive the data have been made publicly available. For some individual countries, using dietary surveys would also have been an alternative. However,

underreporting is a persistent problem in dietary survey,[167,168] and regional differences in survey methods would have meant that our results would not be comparable between countries. In contrast to dietary surveys, waste-adjusted food-availability estimates indicate levels of energy intake per region that reflect differences in the prevalence of overweight and obesity across regions.[169]

Future form of the Indicator

Disease-specific mortality rates are changing in every region. This indicator will be improved to more accurately capture the changing effect of diet on mortality by normalising by the disease-specific mortalities to which it is attributed.

Additional analysis

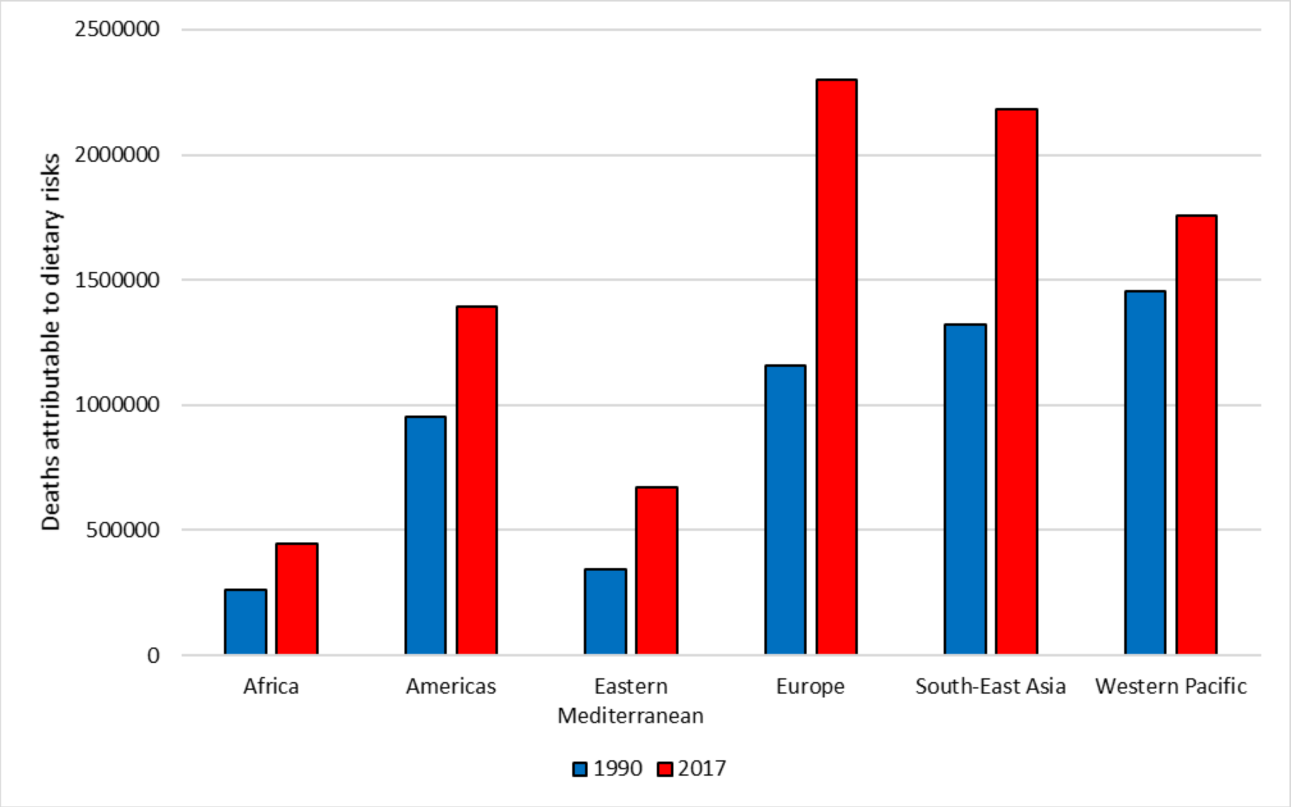

Figure 47: Deaths attributable to dietary risk factors in 1990 and 2017 by WHO region.

Indicator 3.6: Mitigation in the Healthcare Sector

Methods

This indicator is in the form of healthcare-associated GHG emissions per capita per year, including direct emissions from healthcare facilities as well as emissions from the consumption of goods and services supplied by other sectors. Results are calculated by assigning aggregate national health expenditures from WHO to final demand for 'Health and Social Work' sectors in the MRIO model. Environmental satellite accounts including GHG emissions accompany each MRIO model. Consumption-based GHG emissions are then calculated using the standard Leontief inverse technique.[197]

Results for years after the MRIO model year are achieved through deflation of healthcare expenditure data. Both WIOD and EXIOBASE MRIO models were run for this analysis and results compared; WIOD results are shown as they reflect the most recent environmental satellite accounts.[198,199] WIOD tables are in US dollars, while EXIOBASE tables are in euros. For expenditure years after the model baseline, WHO expenditure data in nominal US dollars expenditures are converted to nominal national currencies using market exchange rates, deflated in national currencies to baseline year using consumer price indices from the World Bank, and converted to baseline model year euros currency (dollars or euros) using market exchange rates.[200,201]

The Lancet Countdown reported healthcare sector GHG emissions for the first time in 2019.[1] In that report, global healthcare emissions were found to contribute approximately 4.6% of global emissions, with large disparities in per capita emissions of more than 40x across the countries studied. Independent research by Pichler et al. on CO_2 emissions (excluding other GHGs) associated with health care in OECD countries (excluding Chile) as well as India and China found a contribution of 4.4% in 2014, while an NGO effort covering all GHG emissions estimated 4.4% in 2014.[202,203] The Pichler et al. work considered temporal trends and introduced adjustments into the emissions satellite accounts of the MRIO model EORA to reflect shifts in major GHG emissions sources that occurred between the baseline model year and when each healthcare expenditure occurred. Based on this suggestion, the Lancet Countdown modelling approach has been updated in the same way, using the PRIMAP database of national GHG emissions to adjust emissions by sector relative to the baseline year.[204]

Figure 48 shows the effect of this methodological update for the example of Spain. Adjusting the EE MRIO tables to account for changes in carbon emissions includes decarbonization of Spain's energy system (domestic, in light blue) and those of Spain's trading partners (imported, in light red). In the case of Spain, these adjustments lower 2016 emissions by approximately 11%.

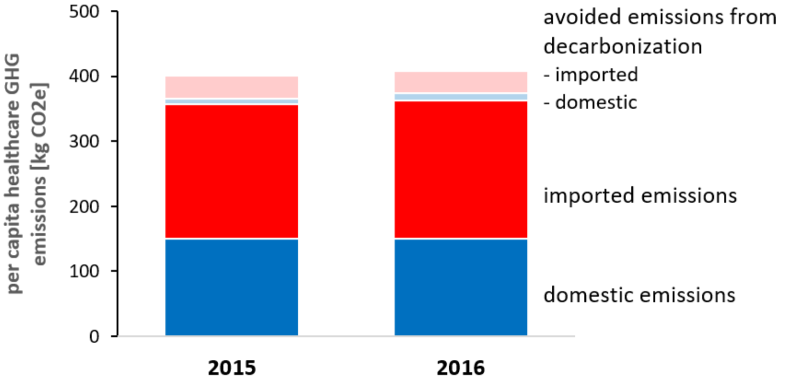

Figure 48: Effect of modelling updates to per capital healthcare GHG emissions for Spain, 2015-2016.

Data

1. Environmentally extended multi-region input-output tables: WIOD 2013 release with environmental accounts, latest model year 2011, latest emissions account year 2009, air emissions include CO_2, CH_4, N_2O, NO_x, SO_x, CO, NMVOC, and NH_3;
2. Per capita health expenditure data is from the World Health Organization's Global Health Expenditure Database; the latest reporting year is 2017.[205] Population data is also from the WHO.[201]
3. Market exchange rates are from UN Statistics Division.[206]
4. Consumer price indices are from the World Bank.[200]
5. HAQ Index values for 2015 are from the Global Burden of Disease project, as published in The Lancet.[207]

Caveats

As only total health expenditure data are available from WHO, all expenditures are assigned to Final Demand, with no separation for investment.

MRIO models are built from aggregated top-down statistical data. Results do not reflect individual health care systems' power purchase agreements for renewable energy or any offsetting activities. Results do not include direct emissions of waste anaesthetic gases from clinical operations nor emissions from metered dose inhalers, as these are not currently reported consistently in national emissions inventories.

Future form of the indicator

This indicator could be expressed in future years by sectoral contributions, in order to isolate the contribution of specific healthcare supplies and activities such as pharmaceutical manufacturing.

Additional analysis

Based on the updated values, global healthcare contributed 4.6% of global GHG emissions (excluding land use change) in 2017, with an increase of 6.1% in absolute emissions over the prior year. This annual increase was nearly twice the rate of the 2015-2016 increase; nearly 65% of the annual increase was due to China, mostly driven by increases in healthcare expenditures.

Over the past decade, per capita healthcare GHG emissions have increased in approximately three-quarters of the world's 40 largest economies, particularly in China where healthcare emissions have more than tripled. However, among ~150 smaller economies, per capita emissions have decreased by more than 7% on average.

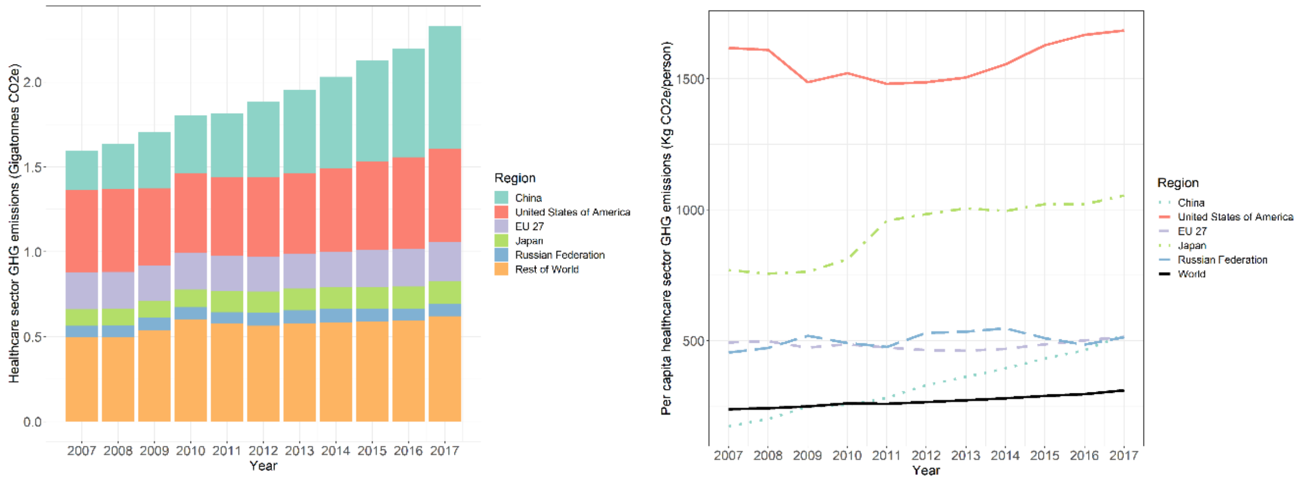

Figure 49: Total and per capita GHG emissions from the healthcare sector of the highest emitters.

Section 4: Economics and Finance

4.1: Health and Economic Costs of Climate Change and Benefits from Mitigation

Indicator 4.1.1: Economic Losses due to Climate-Related Extreme Events

Methods

The Swiss Re Institute provided the data for this indicator. The Swiss Re Institute sigma catastrophe database is an international commercial database recording both natural and man-made disasters from 1970 and has over 12,000 entries.

The term 'natural catastrophe' refers to an event caused by natural forces. Such an event generally results in a large number of individual losses involving many insurance policies. The scale of the losses resulting from a catastrophe depends not only on the severity of the natural forces concerned, but also on man-made factors, such as building design or the efficiency of disaster control in the afflicted region.

Natural catastrophes are categorised as follows:

Category	Peril Group	Peril
	Earthquake	Earthquake
		Tsunami
		Volcano eruption
	Weather-related	Storm
		Flood
		Hail
		Cold, frost
		Drought, bush fires, heat waves
		Other natural catastrophes

For this indicator, we present data for 'weather-related' events only.

Total (insured and uninsured) economic losses reported by Swiss Re are all the financial losses directly attributable to a major event, i.e. damage to buildings, infrastructure, vehicles etc. This also includes losses due to business interruption as a direct consequence of the property damage. Insured losses are gross of any reinsurance, be it provided by commercial or government schemes. Total loss figures do not include indirect financial losses – i.e. loss of earnings by suppliers due to disabled businesses, estimated shortfalls in GDP and non-economic losses, such as loss of reputation or impaired quality of life. Insured losses refer to all insured losses except liability. To calculated uninsured losses, insured losses are subtracted from total losses.

Data are collected from a variety of sources, both internal and external. These include professional insured claims aggregators as well as insurance associations. Among the sources are also official government data, when available. Economic loss data can be estimated on the basis of Swiss Re proprietary catastrophe risk models. Also, if insured loss data are available, economic loss data are estimated on the basis of the local insurance penetration and other event-specific information (such as damages to public infrastructure, number of buildings damaged or destroyed etc.).

Minimum threshold apply to inclusion in the database. At least one of the following must apply, for events recorded in 2019 (with economic values changing each year following changes to US CPI):

- **Insured losses (claims):** USD 52.7 million
- **Economic losses**: USD 105.4 million
- **Casualties**: Dead or missing: 20 ;Injured: 50; Homeless: 2000

Loss values are presented in US$, or if initially expressed in local currency, converted to US$ using year-end exchange rates and are then adjusted for inflation to give current (US$2019) values. The GDP data is taken from

Oxford Economics. Further information on the methodology of the sigma explorer database can be found here: https://www.sigma-explorer.com/documentation/Methodology_sigma-explorer.com.pdf

Once data was received from Swiss Re, economic losses (insured and uninsured) were divided by annual GDP values (in US$ 2019) for each income grouping, also provided by Swiss Re.

Data

1. Swiss Re Institute sigma catastrophe database.[208,209]

Caveats

Only events with measurable economic losses above the threshold levels are included. Each natural catastrophe event recorded is assigned a direct economic loss, and where applicable, an insured loss. Where available, data is taken from official institutions, but where not, estimates are calculated. The process for estimation depends on what data is available. For example, if loss estimates from insurance market data is available, this data may be combined with data on insurance penetration and other event-specific information to estimate total economic losses. If only low-quality information is available, such as a description of the number of homes damaged or destroyed, assumptions on value and costs are made

Additional analysis

Table 9: Insured and uninsured losses from climate-related extreme events 2010-2019.

		Number of Events	Insured Losses/$1000 GDP	Uninsured Losses/$1000 GDP
2010	Low Income	20	$0.02	$0.52
	Lower-Middle	60	$0.01	$1.62
	Upper-Middle	67	$0.11	$6.03
	High Income	117	$0.70	$0.69
2011	Low Income	14	$0.00	$0.75
	Lower-Middle	55	$0.01	$0.83
	Upper-Middle	48	$0.94	$2.76
	High Income	87	$1.13	$0.90
2012	Low Income	29	$0.85	$3.25
	Lower-Middle	48	$0.00	$0.99
	Upper-Middle	56	$0.06	$1.32
	High Income	95	$1.38	$1.38
2013	Low Income	13	$0.01	$0.16
	Lower-Middle	47	$0.27	$3.78
	Upper-Middle	55	$0.13	$1.64
	High Income	112	$0.68	$0.64
2014	Low Income	14	$0.00	$0.30
	Lower-Middle	40	$0.19	$3.09
	Upper-Middle	69	$0.11	$1.57
	High Income	115	$0.54	$0.39
2015	Low Income	18	$0.00	$1.36
	Lower-Middle	55	$0.24	$1.34
	Upper-Middle	62	$0.05	$1.02
	High Income	109	$0.55	$0.45
2016	Low Income	19	$0.29	$5.41
	Lower-Middle	48	$0.12	$1.74
	Upper-Middle	53	$0.08	$2.24
	High Income	106	$0.75	$0.65

2017	Low Income	16	$0.00	$2.61
	Lower-Middle	50	$0.02	$1.30
	Upper-Middle	51	$0.12	$1.11
	High Income	142	$2.68	$2.95
2018	Low Income	20	$0.01	$0.68
	Lower-Middle	49	$0.07	$1.09
	Upper-Middle	33	$0.04	$0.73
	High Income	121	$1.43	$0.93
2019	Low Income	27	$0.31	$6.90
	Lower-Middle	53	$0.09	$2.49
	Upper-Middle	29	$0.05	$1.09
	High Income	127	$0.89	$0.60

Indicator 4.1.2: Costs of Heat-Related Mortality

Methods

This indicator employs an average value of statistical life (VSL) for OECD countries, derived from studies into the average amount of money an individual is willing to pay to reduce the risk of death,[210] to monetise heat-related mortality (data for which is provided by Indicator 1.1.2). The same ratio between VSL and GNI-per-capita is assumed for each country as for the OECD for the years 2000-2018. 169 countries spanning six World Health Organization (WHO) regions around the world were included in the estimation. Population and GNI per capita data is taken from the World Bank.[211,212]

The average VSL applicable for the OECD countries (VSL_{OECD}) was estimated at USD 3.83 million in 2015, and OECD average income (Y_{OECD}) for 2015 was $40,002 ($2015). The assumption is shown in Eq. (1), where Y denotes the gross national income (GNI) per capita, i denotes the country, t denotes time.

$$\frac{VSL_{it}}{Y_{it}} = \frac{VSL_{OECD}}{Y_{OECD}} \qquad (1)$$

In order to calculate the monetised value of mortality relative to per-capita GNI (R), Eq. (2) was applied, in which mortality (M) is multiplied by the fixed VSL-to-GNI per capita-ratio produced by Eq. (1).

$$R_{it} = \frac{VSL_{it} * M_{it}}{Y_{it}} = \frac{VSL_{OECD}}{Y_{OECD}} * M_{it} \qquad (2)$$

In order to calculate the monetised value of mortality as a proportion of GNI (V), Eq. (3) was applied, where mortality (M) as a proportion of total population (P) is multiplied by the fixed VSL-to-average-income-ratio in OECD countries.

$$V_{it} = \frac{VSL_{it} * M_{it}}{GNI_{it}} = \frac{VSL_{it} * M_{it}}{Y_{it} * P_{it}} = \frac{VSL_{OECD}}{Y_{OECD}} * \frac{M_{it}}{P_{it}} \qquad (3)$$

GNI, rather than GDP, is used in this indicator as estimates of VSL are related to income, rather than production. However, using GDP per capita in these calculations would lead to little difference in results, as GDP and GNI per capita values for the OECD are very similar.

Country-level results are aggregated according to WHO regions. Due to data limitations, some countries are not included:, Eritrea, Andorra, Antigua and Barbuda, Bahrain, Barbados, Cook Islands, Dominica, Grenada, Kiribati, Maldives, Malta, Marshall Islands, Micronesia, Monaco, Montenegro, Nauru, Niue, Palau, Saint Kitts and Nevis, Saint Lucia, San Marino, Seychelles, Singapore, South Sudan, Tonga, Tuvalu. The population of these countries accounts for 0.3% of the total global population.

Data

1. Heat-related mortality data, as described in Indicator 1.1.3.
2. VSL estimate taken from the OECD Mortality Risk Valuation in Environment, Health and Transport Policies.[213]
3. Population and GNI per capita data is taken from the World Bank.[211,212]

Caveats

There are three principal caveats to this indicator. First, VSL values rely on estimates of 'willingness to pay' by individuals. The results of these studies highly depend on the survey design and characteristics of the individuals surveyed. Second, because studies examining country- or region-specific VSLs are rare, a simplified, non-specific method of assuming implicit VSL values for all non-OECD countries is employed. Third, and linked to the above, the calculation method employed assumes that the average individual's willingness to pay to reduce the risk of death is linked to the GNI per capita of the country in which they find themselves.

Future form of the indicator

In future reports, Values of Life Year Lost (VLY) rather than VSL will be used, in order to take into account the age distribution of heat-related mortality.

Additional analysis

Table 10: Monetised value of heat-related mortality by WHO region.

		Europe	Western Pacific	South-East Asia	Americas	Eastern Mediterranean	Africa
2000	the value relative to per-capita GNI (million)	5.43	4.29	1.56	1.43	0.72	0.52
	the value as a proportion of GNI	0.63%	0.26%	0.10%	0.17%	0.15%	0.08%
2001	the value relative to per-capita GNI (million)	6.05	4.35	1.55	1.68	0.68	0.47
	the value as a proportion of GNI	0.70%	0.26%	0.10%	0.20%	0.14%	0.07%
2002	the value relative to per-capita GNI (million)	6.12	4.49	2.39	2.20	0.85	0.61
	the value as a proportion of GNI	0.71%	0.27%	0.15%	0.26%	0.17%	0.09%
2003	the value relative to per-capita GNI (million)	8.35	3.95	2.61	1.76	0.78	0.74

	the value as a proportion of GNI	0.96%	0.23%	0.16%	0.20%	0.15%	0.10%
2004	the value relative to per-capita GNI (million)	5.06	4.43	2.31	1.22	0.72	0.59
	the value as a proportion of GNI	0.58%	0.26%	0.14%	0.14%	0.14%	0.08%
2005	the value relative to per-capita GNI (million)	6.00	5.00	2.93	2.28	0.69	0.80
	the value as a proportion of GNI	0.68%	0.29%	0.17%	0.26%	0.13%	0.11%
2006	the value relative to per-capita GNI (million)	7.44	4.92	2.11	2.06	0.85	0.66
	the value as a proportion of GNI	0.85%	0.28%	0.12%	0.23%	0.16%	0.09%
2007	the value relative to per-capita GNI (million)	7.03	4.70	2.57	2.22	0.76	0.75
	the value as a proportion of GNI	0.80%	0.27%	0.15%	0.25%	0.14%	0.10%
2008	the value relative to per-capita GNI (million)	6.12	4.01	2.11	1.86	0.79	0.71
	the value as a proportion of GNI	0.69%	0.23%	0.12%	0.20%	0.14%	0.09%
2009	the value relative to per-capita GNI (million)	6.08	4.67	3.57	1.85	0.97	0.81
	the value as a proportion of GNI	0.68%	0.26%	0.20%	0.20%	0.17%	0.10%
2010	the value relative to per-capita GNI (million)	8.23	5.89	4.63	2.73	1.09	0.94
	the value as a proportion of GNI	0.92%	0.33%	0.26%	0.29%	0.18%	0.11%
2011	the value relative to per-capita GNI (million)	6.64	5.38	1.91	2.82	0.95	0.81
	the value as a proportion of GNI	0.74%	0.30%	0.10%	0.30%	0.16%	0.09%
2012	the value relative to per-capita GNI (million)	8.03	5.02	4.09	3.06	1.06	0.86
	the value as a proportion of GNI	0.89%	0.28%	0.22%	0.32%	0.17%	0.10%
2013	the value relative to per-capita GNI (million)	6.99	6.82	3.04	2.38	0.90	0.95
	the value as a proportion of GNI	0.77%	0.38%	0.16%	0.25%	0.14%	0.10%
2014	the value relative to per-capita GNI (million)	6.41	4.98	4.13	2.28	1.00	0.78
	the value as a proportion of GNI	0.71%	0.27%	0.22%	0.24%	0.15%	0.08%
2015	the value relative to per-capita GNI (million)	8.27	5.11	3.40	2.94	1.10	1.10
	the value as a proportion of GNI	0.91%	0.28%	0.18%	0.30%	0.17%	0.11%
2016	the value relative to per-capita GNI (million)	8.06	6.77	4.51	3.46	1.28	1.30

	the value as a proportion of GNI	0.88%	0.37%	0.23%	0.35%	0.19%	0.13%
2017	the value relative to per-capita GNI (million)	7.81	7.49	4.07	2.91	1.40	1.11
	the value as a proportion of GNI	0.85%	0.40%	0.21%	0.29%	0.21%	0.11%
2018	the value relative to per-capita GNI (million)	10.61	8.13	3.78	3.26	1.26	0.98
	the value as a proportion of GNI	1.15%	0.43%	0.19%	0.33%	0.18%	0.09%

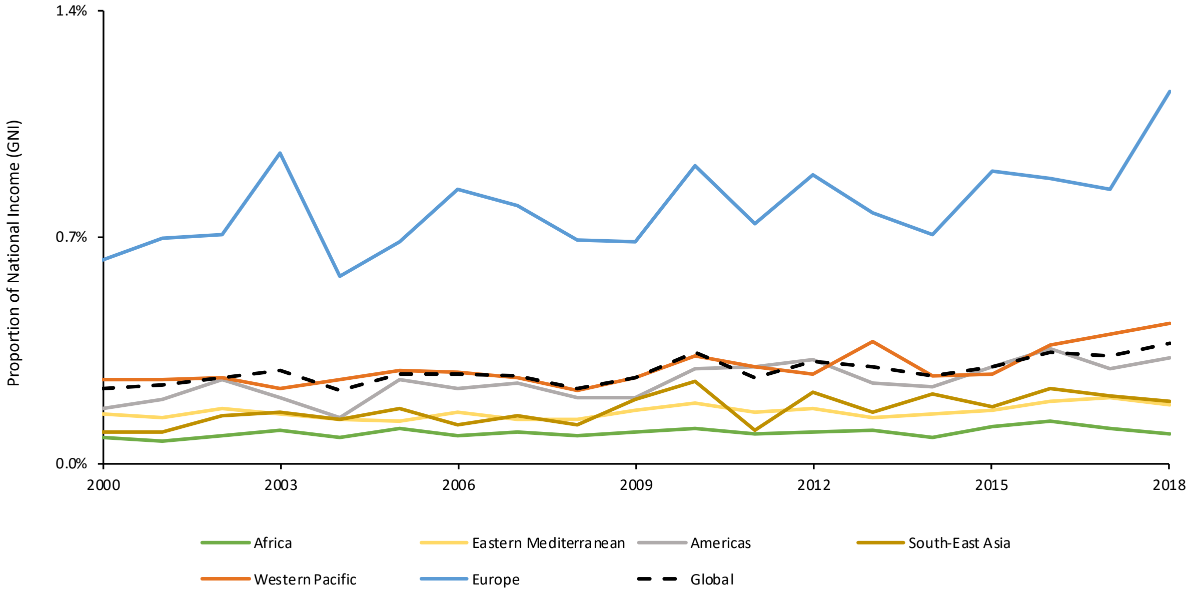

Figure 50: Monetised value of heat-related mortality as the proportion of GNI in a given region.

Indicator 4.1.3: Loss of Earnings from Heat-Related Reduction in Labour Capacity

Methods

Indicator 1.1.3 provides data on heat-related labour capacity loss, in terms of lost work hours. This data has been partially disaggregated for use in this indicator. Lost work hours have been provided for 25 countries, across four sectors (services, manufacturing, construction and agriculture) and for four years (1995, 2003, 2008, 2015). In order to calculate potential loss of earnings from this labour capacity loss, it was necessary to compile a dataset of average earnings per hour for each of these countries, sectors and years.

Earnings and income statistics were compiled from the ILOSTAT databases held by the International Labour Organisation (ILO).[20] The datasets used were those under the 'Earning and Labour Income' category.

Table 11 summarises the number of data points available under each of the potentially relevant datasets under the 'Earning and Labour Income' category for the 25 countries, with the search limited to the time range 1995-2019.

Table 11: Summary of data points available for 25 countries within ILO datasets potentially relevant to earning and labour income.

Earnings and labour income	IND	CHN	USA	JPN	RUS	DEU	IRN	KOR	CAN	IDN	MEX	BRA
Mean nominal monthly earnings of employees by sex and economic activity -- Harmonized series: Annual	36	816	249	2232	861	1821	63	2889	1143	4248	2709	5166
Mean nominal monthly earnings of employees by sex and economic activity: Annual	12	294	112	801	343	831	21	1018	515	1778	1053	2055
Mean nominal monthly earnings of employees by sex and economic activity: Quarterly	0	0	0	0	0	627	0	59	0	1784	8123	3360
Mean nominal monthly earnings of employees by sex and economic activity: Monthly	0	0	0	1222	272	0	0	552	5004	0	0	0
Mean nominal monthly earnings of employees by sex and occupation -- Harmonized series: Annual	0	0	0	0	432	369	0	783	594	1887	915	2328
Mean nominal monthly earnings of employees by sex and occupation: Annu al	0	0	0	0	159	123	0	300	216	933	607	1146
Mean nominal hourly earnings of employees by sex and occupation: Annu al	0	0	48	0	60	123	0	270	0	51	96	54
Mean nominal hourly earnings of employees by sex and occupation -- Harmonized series: Annual	0	0	120	0	180	369	0	783	0	0	180	162
Average monthly earnings of prime-	0	0	0	0	0	0	0	0	0	0	66	66

age employees by sex, household type and presence of children: Annual												
Average monthly earnings of prime-age employees by sex, household type and rural / urban areas: Annual	0	0	0	0	0	0	0	0	0	0	54	0
**TOTAL PER COUNTRY**	**48**	**1110**	**529**	**4255**	**2307**	**4263**	**84**	**6654**	**7472**	**10681**	**13803**	**14337**

**Earnings and labour income (continued)**	**CRI**	**EGY**	**PHL**	**PAK**	**ZAF**	**ESP**	**KHM**	**VNM**	**BGD**	**NGA**	**THA**	**COL**	**ITA**
Mean nominal monthly earnings of employees by sex and economic activity -- Harmonized series: Annual	3420	3749	3491	1581	1464	1857	2634	2964	465	120	1755	2919	571
Mean nominal monthly earnings of employees by sex and economic activity: Annual	1346	1412	1391	732	556	751	1054	1224	173	72	719	1214	204
Mean nominal monthly earnings of employees by sex and economic activity: Quarterl y	0	3977	5597	1464	886	0	0	3624	407	0	4307	3623	0
Mean nominal monthly earnings of employees by sex and economic activity: Monthly	0	0	0	0	0	0	0	0	0	0	0	3990	0
Mean nominal monthly earnings of employees by sex and occupation -- Harmonized series: Annual	889	900	1296	912	1458	1077	1176	1166	204	198	678	144	348
Mean nominal monthly earnings of employees by sex and occupation: Ann ual	423	399	666	520	630	392	574	594	87	102	324	174	116
Mean nominal hourly earnings of employees by sex and occupation: Ann ual	318	30	0	129	278	258	0	78	0	0	21	279	116
Mean nominal hourly earnings of employees by sex and occupation -- Harmonized series: Annual	342	0	0	297	0	774	0	198	0	0	63	45	348

Average monthly earnings of prime-age employees by sex, household type and presence of children: Annual	0	0	0	0	0	0	65	66	0	0	0	66	0
Average monthly earnings of prime-age employees by sex, household type and rural / urban areas: Annual	0	0	0	0	0	0	52	54	0	0	0	54	0
**TOTAL**	**6738**	**10467**	**12441**	**5635**	**5272**	**5109**	**5555**	**9968**	**1336**	**492**	**7867**	**12508**	**1703**

The variable for each of these datasets is 'earnings'. ILO state that 'statistics of earnings presented in ILOSTAT refer, to the extent possible, to employees' gross remuneration, i.e. the total before any deductions are made by the employer in respect of taxes, contributions of employees to social security and pension schemes, life insurance premiums, union dues and other obligations of employees. Earnings include direct wages and salaries, remuneration for time not worked (excluding severance and termination pay), bonuses and gratuities, and housing and family allowances paid by the employer directly to the employee'.[214]

The above table shows that the data coverage is variable, with the coverage of some countries apparently quite comprehensive, of others relatively sparse. There is also variation in the coverage of the different indicators. The table also shows that while there is some data on hourly earnings, the data on earnings per month are generally more comprehensive.

The process for assembling a combined dataset for hourly earnings for the 25 countries, across four sectors (services, manufacturing, construction and agriculture) and for each year (1995, 2003, 2008 and 2015) therefore required some gap filling and some assumptions in place of data being available in all cases in exactly the format required.

Table 12 below summarises the results of this process, providing hourly earnings for each country, sector and year, in real USD (2018). Following the table a summary is given of the main steps that needed to be taken to integrate the various ILO datasets into this single integrated table of data for the required countries, sectors and years.

Table 12: Average hourly earnings by sector for 2512 countries and selected years, in real US 2018$. Source: various datasets available from ILOSTAT.[20]

		Year	Services	Manufacturing	Agriculture	Construction
**India**	**IND**	1995	0.355	0.355	0.355	0.355
		2003	0.183	0.183	0.183	0.183
		2008	0.842	0.560	0.397	0.583
		2015	1.051	0.835	0.503	0.774
**China**	**CHN**	1995	0.600	0.491	0.334	0.549
		2003	1.573	0.992	0.553	0.911
		2008	3.940	1.954	1.046	1.738
		2015	5.610	4.528	2.615	4.002
**United States of America**	**USA**	1995	16.712	21.199	13.548	24.182
		2003	18.969	21.839	13.548	25.552
		2008	32.424	25.722	12.946	26.407

		2015	32.373	27.272	13.423	28.365
**Japan**	**JPN**	1995	29.506	28.201	32.196	32.196
		2003	24.096	20.156	21.543	21.543
		2008	20.286	19.115	18.407	18.407
		2015	14.833	14.847	16.575	16.575
**Russian Federation**	**RUS**	1995	2.721	2.210	1.008	2.374
		2003	2.721	2.210	1.008	2.374
		2008	5.609	4.349	2.296	5.033
		2015	3.765	3.203	1.980	3.007
**Germany**	**DEU**	1995	23.318	27.374	14.639	25.895
		2003	23.318	23.300	11.735	21.169
		2008	30.149	33.466	12.418	26.571
		2015	25.064	29.680	17.629	21.919
**Islamic Republic of Iran**	**IRN**	1995	4.735	1.575	4.735	4.735
		2003	4.735	4.735	4.735	4.735
		2008	4.735	4.735	4.735	4.735
		2015	4.735	4.735	4.735	4.735
**Republic of Korea**	**KOR**	1995	16.474	13.864	8.023	17.075
		2003	14.221	13.347	8.023	13.347
		2008	16.813	16.856	8.023	17.570
		2015	17.354	12.466	9.763	8.783
**Canada**	**CAN**	1995	14.978	21.395	20.941	22.509
		2003	14.301	20.396	20.632	20.652
		2008	18.861	25.887	25.571	27.721
		2015	18.128	20.525	14.171	23.550
**Indonesia**	**IDN**	1995	0.815	0.641	0.422	0.664
		2003	0.815	0.641	0.422	0.664
		2008	0.883	0.660	0.567	0.775
		2015	0.923	0.758	0.437	0.771
**Mexico**	**MEX**	1995	1.784	1.770	1.064	1.351
		2003	3.607	2.306	1.573	1.749
		2008	3.215	2.859	1.606	2.911
		2015	2.645	1.900	1.385	1.351
**Brazil**	**BRA**	1995	7.010	6.544	2.798	4.324
		2003	1.890	1.772	0.673	1.205
		2008	3.884	3.681	1.794	2.957
		2015	3.462	3.547	1.873	2.799
**Costa Rica**	**CRI**	1995	3.62	2.88	1.93	2.76
		2003	3.54	3.03	1.83	2.41
		2008	8.04	7.03	1.33	5.31
		2015	6.12	5.96	3.08	4.12
**Egypt**	**EGY**	1995	1.19	1.02	0.72	1.11
		2003	1.16	0.87	0.54	1.06

		2008	0.08	0.10	0.11	0.14
		2015	1.42	1.42	1.21	1.52
**Philippines**	**PHL**	1995	2.62	2.46	1.45	1.83
		2003	2.18	1.62	0.91	1.17
		2008	3.04	2.37	1.40	2.09
		2015	0.02	0.01	0.02	0.01
**Pakistan**	**PAK**	1995	0.89	0.89	0.89	0.89
		2003	0.54	0.54	0.54	0.54
		2008	0.84	0.58	0.40	0.58
		2015	1.05	0.84	0.50	0.77
**South Africa**	**ZAF**	1995	10.11	7.82	0.75	4.32
		2003	3.34	2.71	0.75	1.53
		2008	7.07	6.72	1.78	5.13
		2015	1.99	1.87	1.06	1.44
**Spain**	**ESP**	1995	18.87	16.69	7.02	13.74
		2003	17.61	17.76	7.02	13.97
		2008	16.44	19.48	11.49	16.08
		2015	11.83	14.92	8.14	12.48
**Cambodia**	**KHM**	1995	0.37	0.47	0.25	0.27
		2003	0.48	0.40	0.21	0.43
		2008	0.88	0.48	0.31	0.56
		2015	1.09	0.99	0.63	1.07
**Vietnam**	**VNM**	1995	0.73	0.56	0.45	0.56
		2003	0.73	0.56	0.45	0.56
		2008	0.73	0.56	0.45	0.56
		2015	1.55	1.47	0.87	1.25
**Bangladesh**	**BGD**	1995	0.36	0.36	0.36	0.36
		2003	0.18	0.18	0.18	0.18
		2008	0.84	0.56	0.40	0.58
		2015	1.37	0.95	0.73	0.89
**Nigeria**	**NGA**	1995	21.75	1.67	1.37	2.22
		2003	21.75	1.67	1.37	2.22
		2008	25.28	0.97	1.37	2.22
		2015	21.75	1.67	1.37	2.22
**Thailand**	**THA**	1995	2.38	1.83	1.08	1.48
		2003	2.38	1.83	1.08	1.48

		2008	2.38	1.83	1.08	1.48
		2015	2.01	1.91	0.75	1.31
**Colombia**	**COL**	1995	2.86	2.36	2.85	3.09
		2003	1.77	1.21	0.63	1.01
		2008	3.31	2.93	1.70	2.34
		2015	2.44	2.26	1.41	1.99
**Italy**	**ITA**	1995	16.36	15.27	10.43	15.41
		2003	22.67	10.81	10.43	10.43
		2008	25.00	16.37	14.87	14.92
		2015	23.27	15.49	13.65	14.04
**Mean per sector across 25 countries**			8.312	7.089	4.852	6.825

Currency standardisation

All money values are expressed in real US 2018$. Where ILO data were reported in local currency units, these were first converted to nominal US dollars using the official exchange rate annual data provided by the World Bank World Development Indicators.[215] Nominal USD values were corrected to USD (2018) values using the USD consumer price index (CPI) provided by the World Bank's World Development Indicators.

Identifying relevant sectors

Across the various ILO datasets listed in the table above, which needed to be combined in order to achieve sufficient data coverage, there are different conventions for aggregating employment sectors. Conventions also change between the different reporting years. Wherever possible, earnings for aggregated sectors with titles closely corresponding to 'agriculture', 'construction', 'manufacturing' and 'services' – were selected. However, in some cases such titles were not precise or included other activities – for example agriculture was sometimes combined with 'hunting', 'fishing' or 'forestry'. Under one classification system there is a group called 'elementary occupations', which is defined as including construction, manufacturing and agricultural workers; hence this group was sometimes used for one or more of these three sectors in the absence of more specific data, or it was combined with another partially relevant group and the mean value calculated. The strategy of combining multiple, potentially relevant sectors, with the mean value calculated, was also frequently required in the case of 'services'. In most cases an amalgamated 'services' category was not provided, hence the figure for services was calculated from the mean of the relevant employment sectors or activities that were listed for that year. Examples of sub-sectors selected to contribute to the mean for the services category included: 'wholesale and retail trade'; 'accommodation and food service activities'; 'financial and insurance activities'; 'real estate activities'; 'arts, entertainment and recreation'; 'other service activities'; etc. The specific list of sub-sectors or activities from which the services mean value was calculated, varied between country and year, depending on data availability.

In some countries and sectors the exact required year (1995, 2003, 2008, 2015) was not available, but a close year (the year before or after) was. In such cases the closest year was used, with the appropriate year CPI deflator figure to ensure correct uprating to USD (2018).

Data gaps or discrepancies

Even after combining all available datasets there were some countries with gaps in data for particular sectors or years, or data that appeared to be erroneous. In general the approach was to fill gaps with values taken from the nearest and most comparable sector and year available in that country. This was done after all available data had been converted into real 2018 US dollars, or using the appropriate deflator value for the actual year of the data, to avoid issues of loss or gain of value if a gap was filled by taking data from a different year.

Countries with major problems with data gaps were:

- ***India***: Data was only available for the manufacturing sector, and only between the years 1995 and 2006. The manufacturing values for the available years of 1995 and 2003 (real 2018 values) were copied to the other sectors for these years. This is likely to have resulted in an underestimate for services, though agriculture and construction would have been closer. The 2008 manufacturing value is based on the latest available 2006 data. For the remaining sectors in 2008 the Pakistan values were used. Pakistan values were also used for all sectors in 2015. Comparing the GDP per capita of Pakistan and India shows that in 2008 the values were very close (both on a PPP and official exchange rate basis), and the available manufacturing wage figure for India also very close to that found for Pakistan, giving some basis for the resort to using Pakistan's values in the other sectors. By 2015 the countries' GDPs per capita were still close, although India's was slightly higher. On this basis, using Pakistan's wage values as proxies for India's was also deemed acceptable, although this may have resulted in a slight underestimate for India.
- ***Iran***: Data only available for manufacturing, up to 2001. This most recent value, after uprating, was copied to all other sectors and years with missing data.
- ***Bangladesh***: Data is available in all sectors but only from 2016. 2015 adopts 2016 values. Earlier years are copied from India, which itself has copied some values from Pakistan (as described above). Bangladesh's GDP per capita has been similar to India and Pakistan over the period, but has consistently been the lowest of the three. Hence borrowing from these countries may have resulted in a slight overestimate of wage values for Bangladesh in the years before 2015.
- ***Nigeria***: Only values from years 2011 and 2013 are available. 2015 adopts 2013 values in all sectors. 2008 adopts 2011 values for services and manufacturing; however 2011 values for agriculture and construction were suspected erroneous, so 2013 values were used. 1995 and 2003 simply adopt 2013 values (appropriately uprated).
- ***Thailand***: No data earlier than 2011. 1995, 2003 and 2008 adopt 2011 values, appropriately uprated, in all sectors.

Countries with relatively minor problems with data gaps were:

- ***Japan***: No data in any year for agriculture. Values copied from construction sector in relevant years.
- ***Germany***: No relevant data on services before 2003. 1995 adopts 2003 value.
- ***Republic of Korea***: No data for agriculture before 2009 – this value (appropriately uprated) used for previous years for this sector
- ***USA*****:** No data for agriculture 1995 – 1995 adopts 2003 value
- ***Costa Rica:*** Construction and agriculture 2015 values are from 2014. All 2003 values are from 2004
- ***Pakistan:*** For the year 1995 the only sector available is manufacturing – this value is copied to the other sectors for this year. 2003 is represented by 2004 data; 2008 by 2009 data
- ***South Africa:*** No data for agriculture before 2000, and 2000 value appeared highly anomalous – hence 1995 agriculture value adopts 2003 value (appropriately uprated). No data for agriculture in 2008, 2007 value is adopted
- ***Spain:*** Agriculture 1995 and 2003 both adopt the 2002 value
- ***Cambodia:*** For the year 1995 all sectors adopt 1999 values; for 2003 all sectors adopt 2001 values
- ***Vietnam:*** All sectors in years 1995, 2003 and 2008 adopt 2007 values

Countries with some suspected erroneous data points were:

- ***Indonesia***: Data for 1995 across all sectors appeared to be erroneous – in the most extreme case the wage for agriculture was ten times higher in 1995 than in 2003, and this was not corrected for by the available exchange rate data. In the absence of an explanation, Indonesia's 1995 data was assumed to be erroneous and 2003 values adopted instead.
- ***Russian Federation***: The 1995 exchange rate for the Russian Federation did not appear to correct extremely high values for earnings in nominal LCUs, with a result that the figures appeared erroneous. Here again 2003 values are adopted for all sectors.
- ***Egypt***: All sector values in 2008 appear surprisingly low compared to other years – however these values have been maintained
- ***Philippines***: All sector values in 2015 appear surprisingly low compared to other years – however these values have been maintained
- ***Nigeria***: an extreme difference was observed in all years between services and the other sectors, with services wages 10 to 20 times higher than those in other sectors. However, this was consistent across all years and so not corrected.
- ***Colombia***: 2008 data was extremely low relative to other years suspected to be erroneous, or not sufficiently corrected for by exchange rates. 2008 adopts 2010 data for all sectors, which is more consistent with other years

Converting from monthly to hourly earnings

As noted, wherever possible data was selected from datasets that reported in terms of earnings per hour, however the majority of data points could only be covered by data reported in monthly terms, with some expressed as per week. In order to convert from monthly to weekly earnings, data points were divided by 4.33, following ILO convention. In order to convert from weekly to hourly earnings, a standard assumption of 40 hours per week was used. This is a simplification, as the actual number of hours worked per week could vary substantially between countries and sectors. It is possible that hours worked per week may be available in other ILOSTAT databases, however, in its description of the earnings and labour cost indicators, ILO notes that in order to derive hourly earnings, 'monthly earnings are divided by 4.33 weeks and then by actual weekly hours worked for each gender, *if available*' (emphasis added). This implies that where hourly earnings are not already provided in ILOSTAT databases, this is in part because data on hours worked per week are not available.

Producing estimates for total potential loss of income

Once the earnings dataset was complete it was multiplied by the data on potential work hours lost by country and sector, provided by Indicator 1.1.3, to provide absolute values in US$ billion (2018). To present these values as a proportion of GDP, national GDP values were taken from the International Monetary Fund's World Economic Outlook Database,[216] and were uprated to US$ (2018) using the US dollar GDP deflator from the World Bank World Development Indicators dataset.[215]

Data

1. Data to estimate labour capacity lost as described in Indicator 1.1.3.
2. ILOSTAT databases held by the International Labour Organisation (ILO).[20] The datasets used were those under the 'Earning and Labour Income' category.
3. Exchange rate annual data provided by the World Bank World Development Indicators.[215]

Caveats

As described in the Method, producing a complete dataset on hourly earnings by country and sector across multiple years, required some simplifying assumptions to be made in order to fill gaps in data. Such key assumptions and caveats are:

- The use of different combinations of ILOSTAT databases, rather than one single dataset – there are risks of inconsistencies, for example associated with different classifications and reporting methods
- Some data has been copied from the closest available year and / or most similar sector in the same country, either in order to fill data gaps, or because some data appeared to be erroneous.
- Some datapoints were derived from the averaging of the data for a number of different sub-sectors or employment activities that could be considered part of the broader sector in question. This approach was most frequently applied for the services category, but not exclusively. The changing reporting conventions between datasets and years meant that it was not possible to keep the sub-sectors and employment activities entirely consistent between different countries and years, when sector earnings needed to be aggregated in this way.
- The conversion of monthly data to hourly was carried out on the basis of a standard assumption of 4.33 weeks per month, and 40 hours per week.

All of these issues mean that caution should be exercised when examining results for any particular country. It is suggested that the results should not be used to draw specific inferences for any particular country, but rather to consider global trends and the types of countries and sectors that could be more affected by heat-related loss of income, than others. In addition, it must be emphasised that the results produced are the *potential* loss of earnings, rather than actual, and the bearer of the costs could vary between countries and sectors. In some instances workers may be able to claim sick pay, in which case the loss would potentially be borne by the employer through paying for non-productive time. In other instances, no arrangements for sick pay may be in place, in which case it would be the worker who would bear the cost through a direct loss of earnings due to inability to work.

Future form of the indicator

Options to develop the indicator in future Lancet Countdown reports could include exploring other sources of data to supplement the ILO databases and develop a more robust and complete dataset for the existing set of 25 countries, considering annual change in labour capacity loss, and adding more countries to the dataset, depending on data availability.

Additional analysis

Table 13 and Table 14, below, present the full results by country. First, Table 13 presents the potential annual earnings lost in US2018 dollars, for each of the years and countries studied. Second, Table 14 presents the potential earnings losses expressed as percentage shares of GDP per country. Following these tables, Figure 53 below presents the resulting earnings losses by sector and year as percentage shares of GDP per country averaged across World Bank Income Groups. Losses in upper-middle and lower-middle income countries increase as a share of GDP through the time periods, with this trend more noticeable in the lower-middle income countries. In 1995, 2003 and 2008 losses as a share of GDP are highest in low income countries, but are on a declining trend through the time periods. This is not due to a reduction in the actual earnings losses, which generally increase through the time periods in most countries. Rather, it is due to rapid increases in GDP, from a low starting base, in low income countries, so that even increasing losses are smaller as shares of GDP. It is also important to note that by 2015 there are no countries in the sample of 25 that remain in the low income category, as all countries' economies have grown to the extent that they are lower-middle income or above**.**

Table 13: Potential loss of earnings, billions of 2018 US$.

	Year	Services	Manufacturing	Agriculture (shade)	Construction (shade)	Agriculture (sun)	Construction (sun)
IND	1995	1.71	2.26	21.15	1.28	39.34	2.37
	2003	1.07	1.31	12.12	1.05	22.59	1.96
	2008	4.85	4.03	23.38	5.28	44.44	10.03
	2015	11.40	8.79	33.90	12.94	61.49	23.47
CHN	1995	1.24	3.40	8.18	0.93	16.08	1.83
	2003	5.57	7.39	11.81	2.39	22.85	4.62
	2008	12.62	10.81	14.22	5.32	29.49	11.04
	2015	26.04	20.82	25.05	17.20	50.29	34.53
USA	1995	10.13	8.74	1.97	8.31	4.72	19.86
	2003	9.10	5.86	1.03	8.62	2.59	21.76
	2008	14.78	5.83	0.85	8.76	2.18	22.52
	2015	23.40	8.64	1.37	11.85	3.32	28.76
JPN	1995	8.62	9.56	5.22	9.44	11.17	20.19
	2003	3.89	3.32	1.81	3.72	3.88	7.99
	2008	5.14	4.28	1.94	3.89	4.24	8.49
	2015	3.98	3.05	1.43	3.14	3.07	6.74
RUS	1995	0.024	0.029	0.024	0.033	0.077	0.104
	2003	0.020	0.024	0.018	0.024	0.056	0.074
	2008	0.103	0.085	0.060	0.117	0.172	0.333
	2015	0.059	0.045	0.035	0.059	0.108	0.185
DEU	1995	0.084	0.196	0.039	0.203	0.132	0.687
	2003	0.157	0.250	0.037	0.198	0.119	0.640
	2008	0.055	0.105	0.011	0.087	0.039	0.310
	2015	0.287	0.385	0.041	0.251	0.121	0.731
IRN	1995	0.23	0.08	0.63	0.25	1.55	0.62
	2003	0.29	0.34	0.79	0.37	1.90	0.88
	2008	0.39	0.37	0.81	0.52	1.91	1.23
	2015	0.57	0.49	0.90	0.69	2.04	1.57
KOR	1995	0.69	0.88	0.54	0.91	1.15	1.95
	2003	0.31	0.35	0.23	0.36	0.54	0.83
	2008	1.00	0.92	0.39	0.92	0.88	2.07
	2015	1.02	0.64	0.32	0.39	0.73	0.89
CAN	1995	0.08	0.09	0.06	0.08	0.17	0.24
	2003	0.04	0.05	0.03	0.06	0.09	0.20
	2008	0.05	0.05	0.03	0.09	0.09	0.29
	2015	0.08	0.05	0.02	0.12	0.06	0.38
IDN	1995	0.79	0.71	3.30	0.55	7.05	1.19
	2003	0.82	0.75	3.75	0.56	8.26	1.22
	2008	1.00	0.77	4.59	0.76	10.15	1.68
	2015	1.67	1.37	3.79	1.36	8.30	2.99
MEX	1995	0.33	0.29	0.51	0.14	1.20	0.34
	2003	0.89	0.51	0.62	0.28	1.53	0.70
	2008	0.86	0.63	0.54	0.62	1.29	1.51

	2015	1.06	0.59	0.67	0.38	1.54	0.88
BRA	1995	3.51	2.60	5.04	1.82	12.05	4.34
	2003	1.18	0.91	1.11	0.62	2.61	1.47
	2008	2.54	2.07	2.62	1.85	6.29	4.44
	2015	4.03	2.59	2.30	2.73	5.36	6.38
CRI	1995	0.02	0.02	0.03	0.01	0.08	0.03
	2003	0.02	0.02	0.03	0.01	0.07	0.04
	2008	0.05	0.03	0.01	0.04	0.04	0.09
	2015	0.06	0.03	0.05	0.03	0.11	0.08
EGY	1995	0.09	0.09	0.29	0.08	0.82	0.24
	2003	0.13	0.07	0.26	0.12	0.72	0.35
	2008	0.01	0.01	0.08	0.03	0.20	0.08
	2015	0.34	0.23	0.88	0.52	2.17	1.28
PHL	1995	0.91	0.70	3.60	0.50	7.77	1.08
	2003	1.22	0.60	2.48	0.48	5.27	1.02
	2008	2.10	0.91	4.33	0.98	8.96	2.03
	2015	0.02	0.01	0.05	0.01	0.11	0.02
PAK	1995	0.90	0.63	4.44	0.68	7.82	1.20
	2003	0.75	0.66	3.14	0.45	5.51	0.79
	2008	1.36	0.82	3.02	0.62	5.37	1.11
	2015	2.00	1.66	4.13	1.16	7.36	2.07
ZAF	1995	0.09	0.07	0.02	0.04	0.08	0.13
	2003	0.05	0.03	0.02	0.02	0.06	0.06
	2008	0.07	0.06	0.02	0.07	0.06	0.26
	2015	0.04	0.02	0.01	0.03	0.05	0.11
ESP	1995	0.20	0.22	0.10	0.21	0.32	0.67
	2003	0.46	0.48	0.14	0.60	0.44	1.86
	2008	0.21	0.23	0.09	0.39	0.32	1.34
	2015	0.36	0.28	0.12	0.26	0.36	0.81
KHM	1995	0.02	0.01	0.37	0.01	0.74	0.01
	2003	0.04	0.05	0.39	0.02	0.75	0.04
	2008	0.08	0.04	0.57	0.03	1.11	0.06
	2015	0.38	0.38	0.68	0.33	1.27	0.62
VNM	1995	0.24	0.24	3.02	0.10	5.83	0.19
	2003	0.42	0.42	3.29	0.30	6.32	0.57
	2008	0.43	0.48	2.61	0.37	5.04	0.72
	2015	1.92	2.39	6.85	1.50	12.70	2.77
BGD	1995	0.31	0.32	4.31	0.11	7.24	0.19
	2003	0.25	0.20	1.82	0.12	3.10	0.21
	2008	1.18	0.73	3.74	0.43	6.46	0.75
	2015	2.52	1.87	7.16	1.15	11.82	1.89
NGA	1995	6.41	0.33	4.76	0.15	10.18	0.32
	2003	9.29	0.41	6.45	0.23	13.84	0.50
	2008	16.18	0.29	5.44	0.38	11.69	0.82
	2015	26.06	0.97	6.95	0.94	14.31	1.93
THA	1995	1.27	1.38	5.61	0.84	10.91	1.64

	2003	1.99	1.83	5.86	0.84	11.32	1.62
	2008	1.85	1.56	5.18	0.89	10.07	1.74
	2015	2.90	2.97	3.87	1.25	7.22	2.33
COL	1995	0.26	0.17	0.76	0.16	1.65	0.36
	2003	0.28	0.14	0.22	0.07	0.46	0.16
	2008	0.50	0.31	0.49	0.19	1.01	0.39
	2015	0.84	0.42	0.64	0.34	1.25	0.66
ITA	1995	0.18	0.29	0.14	0.24	0.43	0.74
	2003	0.94	0.60	0.28	0.49	0.78	1.37
	2008	0.47	0.39	0.17	0.39	0.52	1.19
	2015	1.07	0.68	0.27	0.49	0.74	1.32

Table 14: Potential loss of earnings lost as % of GDP

	Year	Services	Manufacturing	Agriculture (shade)	Construction (shade)	Agriculture (sun)	Construction (sun)
IND	1995	0.30	0.40	3.75	0.23	6.97	0.42
	2003	0.13	0.16	1.46	0.13	2.73	0.24
	2008	0.34	0.28	1.63	0.37	3.10	0.70
	2015	0.51	0.40	1.53	0.58	2.77	1.06
CHN	1995	0.11	0.30	0.72	0.08	1.42	0.16
	2003	0.25	0.33	0.53	0.11	1.02	0.21
	2008	0.23	0.20	0.26	0.10	0.55	0.20
	2015	0.22	0.18	0.21	0.15	0.42	0.29
USA	1995	0.09	0.07	0.02	0.07	0.04	0.17
	2003	0.06	0.04	0.01	0.06	0.02	0.14
	2008	0.09	0.03	0.00	0.05	0.01	0.13
	2015	0.12	0.04	0.01	0.06	0.02	0.15
JPN	1995	0.10	0.11	0.06	0.11	0.13	0.24
	2003	0.07	0.06	0.03	0.06	0.07	0.13
	2008	0.09	0.07	0.03	0.07	0.07	0.14
	2015	0.09	0.07	0.03	0.07	0.07	0.15
RUS	1995	0.00	0.01	0.00	0.01	0.01	0.02
	2003	0.00	0.00	0.00	0.00	0.01	0.01
	2008	0.00	0.00	0.00	0.01	0.01	0.02
	2015	0.00	0.00	0.00	0.00	0.01	0.01
DEU	1995	0.00	0.00	0.00	0.01	0.00	0.02
	2003	0.00	0.01	0.00	0.01	0.00	0.02
	2008	0.00	0.00	0.00	0.00	0.00	0.01
	2015	0.01	0.01	0.00	0.01	0.00	0.02
IRN	1995	0.13	0.04	0.35	0.14	0.87	0.35
	2003	0.14	0.16	0.39	0.18	0.93	0.43
	2008	0.08	0.08	0.17	0.11	0.40	0.26
	2015	0.14	0.12	0.23	0.17	0.52	0.40
KOR	1995	0.08	0.10	0.06	0.11	0.13	0.23

	2003	0.03	0.04	0.03	0.04	0.06	0.09
	2008	0.09	0.08	0.03	0.08	0.07	0.18
	2015	0.07	0.04	0.02	0.03	0.05	0.06
CAN	1995	0.01	0.01	0.01	0.01	0.02	0.03
	2003	0.00	0.00	0.00	0.00	0.01	0.02
	2008	0.00	0.00	0.00	0.00	0.00	0.02
	2015	0.00	0.00	0.00	0.01	0.00	0.02
IDN	1995	0.21	0.19	0.88	0.15	1.88	0.32
	2003	0.24	0.22	1.10	0.16	2.42	0.36
	2008	0.15	0.12	0.70	0.12	1.55	0.26
	2015	0.18	0.15	0.42	0.15	0.91	0.33
MEX	1995	0.06	0.05	0.09	0.03	0.22	0.06
	2003	0.09	0.05	0.06	0.03	0.16	0.07
	2008	0.07	0.05	0.04	0.05	0.10	0.12
	2015	0.09	0.05	0.05	0.03	0.12	0.07
BRA	1995	0.29	0.22	0.42	0.15	1.00	0.36
	2003	0.16	0.12	0.15	0.08	0.35	0.20
	2008	0.13	0.10	0.13	0.09	0.32	0.22
	2015	0.21	0.14	0.12	0.14	0.28	0.34
CRI	1995	0.09	0.09	0.17	0.07	0.44	0.18
	2003	0.10	0.07	0.11	0.06	0.28	0.16
	2008	0.14	0.09	0.04	0.10	0.10	0.26
	2015	0.10	0.06	0.08	0.06	0.20	0.14
EGY	1995	0.10	0.09	0.30	0.09	0.85	0.24
	2003	0.12	0.06	0.23	0.11	0.63	0.31
	2008	0.01	0.01	0.04	0.01	0.10	0.04
	2015	0.10	0.07	0.25	0.15	0.62	0.37
PHL	1995	0.72	0.55	2.85	0.39	6.15	0.85
	2003	1.09	0.53	2.21	0.43	4.69	0.91
	2008	1.03	0.45	2.12	0.48	4.40	1.00
	2015	0.01	0.00	0.02	0.00	0.04	0.01
PAK	1995	0.74	0.52	3.63	0.56	6.40	0.99
	2003	0.63	0.55	2.61	0.38	4.59	0.66
	2008	0.68	0.41	1.51	0.31	2.68	0.55
	2015	0.70	0.58	1.45	0.41	2.58	0.73
ZAF	1995	0.04	0.03	0.01	0.01	0.03	0.06
	2003	0.02	0.01	0.01	0.01	0.02	0.03
	2008	0.02	0.02	0.00	0.02	0.02	0.08
	2015	0.01	0.01	0.00	0.01	0.02	0.03
ESP	1995	0.02	0.02	0.01	0.02	0.03	0.07
	2003	0.04	0.04	0.01	0.05	0.04	0.15
	2008	0.01	0.01	0.00	0.02	0.02	0.07
	2015	0.03	0.02	0.01	0.02	0.03	0.06
KHM	1995	0.38	0.26	7.08	0.12	14.02	0.23
	2003	0.72	0.78	6.20	0.30	11.97	0.59
	2008	0.66	0.35	4.70	0.24	9.16	0.47

	2015	1.99	1.98	3.58	1.75	6.65	3.25
VNM	1995	0.75	0.74	9.42	0.30	18.22	0.58
	2003	0.78	0.79	6.20	0.56	11.93	1.08
	2008	0.37	0.41	2.26	0.32	4.37	0.63
	2015	0.95	1.19	3.39	0.74	6.29	1.37
BGD	1995	0.44	0.46	6.10	0.16	10.24	0.26
	2003	0.30	0.24	2.15	0.14	3.66	0.25
	2008	1.04	0.64	3.29	0.38	5.67	0.66
	2015	1.15	0.85	3.26	0.52	5.38	0.86
NGA	1995	3.15	0.16	2.34	0.07	5.00	0.16
	2003	6.74	0.30	4.68	0.17	10.04	0.36
	2008	4.18	0.07	1.40	0.10	3.02	0.21
	2015	5.00	0.19	1.33	0.18	2.75	0.37
THA	1995	0.49	0.53	2.15	0.32	4.19	0.63
	2003	0.98	0.90	2.87	0.41	5.55	0.79
	2008	0.54	0.46	1.51	0.26	2.95	0.51
	2015	0.68	0.70	0.91	0.30	1.70	0.55
COL	1995	0.15	0.10	0.45	0.10	0.97	0.21
	2003	0.22	0.11	0.17	0.06	0.37	0.13
	2008	0.18	0.11	0.17	0.07	0.36	0.14
	2015	0.27	0.14	0.21	0.11	0.40	0.21
ITA	1995	0.01	0.02	0.01	0.01	0.02	0.04
	2003	0.04	0.03	0.01	0.02	0.04	0.07
	2008	0.02	0.01	0.01	0.01	0.02	0.04
	2015	0.06	0.04	0.01	0.03	0.04	0.07

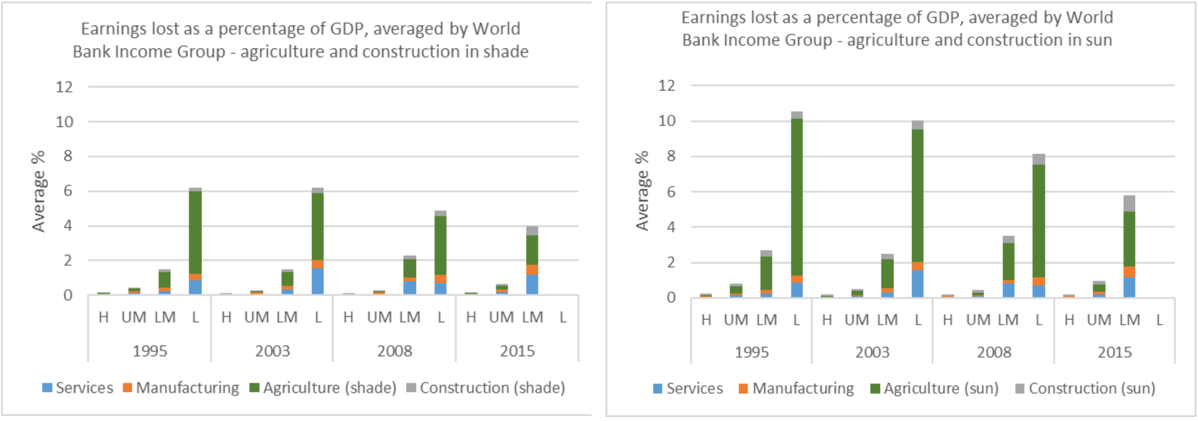

A **B**

Figure 51: Earnings lost as a percentage of GDP, averaged by World Bank Income Group. H = High income; UM = Upper middle income; LM = Lower middle income; L = Low income. Includes data from from 25 countries. Panel A assumes all agriculture and construction activities undertaken in shade; Panel B assumes all agriculture and construction activities undertaken in the sun. By 2015 none of the 25 countries tracked remained in the low-income group.

Indicator 4.1.4: Costs of the Health Impacts of Air Pollution

Methods

This indicator is based on estimates of total Years of Life Lost (YLL) in each member state of the European Union, resulting from $PM_{2.5}$ exposure to emissions from anthropogenic sources, assuming constant levels of emissions and subsequent population exposure to 2115, integrated across the lifetime of the population present in 2015.

The calculations are performed by the GAINS integrated assessment model (see Indicator 3.3 and Kiesewetter et al (2015)[147] for a full description of the model and how YLLs are estimated). Key inputs and assumptions are the following:

- YLLs are calculated based on the loss of life expectancy from all-cause mortality from ambient $PM_{2.5}$ exposure resulting from anthropogenic sources, using exposure-response relationships following the WHO Europe methodology,[153] with population cohort exposure kept constant across lifetimes
- Calculations are based on the population structure present in 2010, using data extracted from UN life tables. However, 2015 population numbers are used to calculate total YLLs from the calculated reduction in life expectancies.
- Increased health risk from $PM_{2.5}$ exposure occurs once population cohorts reach 30 years old with younger cohorts only included once they reach this age, (maximum age = 100). Health consequences for people born after 2015 are not considered.
- Energy production and consumption statistics are taken from the IEA Energy statistics are taken from the IEA World Energy Outlook 2018 (for 2015)[217] and 2019 (for 2018),[134] merged with GAINS information on application of emission control technologies and their emission factors.

Total YLLs in each country and year are then multiplied by an estimated 'Value of a Life Year' (VLY), which is taken to be €50,000 for all countries, for all population cohorts, following the lower bound estimate suggested by Part III of the 2009 European Commission Impact Assessment Guidelines.[218] Average annual values are then calculated by dividing the product of this calculation by 100 (the assumed upper age limit for someone born in 2015). Values given in Euros are converted to US$ using the 2019 official exchange rate given in World Bank World Development Indicators.[215]

Data

1. Energy production and consumption statistics are taken from the IEA Energy statistics are taken from the IEA World Energy Outlook 2018 (for 2015)[217] and 2019 (for 2018).[134]
2. VLY estimate is taken from the European Commission Impact Assessment Guidelines.[218]

Caveats

See indicator 3.3, for caveats related to the calculation of reduced life expectancy.

There is relatively little literature attempting to estimate a VLY, with such literature that does exist largely focusing on European countries. The value employed by this indicator (€50,000) is the lower bound estimate suggested for use by the 2009 European Commission Impact Assessment Guidelines, with the upper value set at €100,000. As such, it is possible that the values presented by this indicator are conservative, however given the relative lack of evidence and complexity in producing estimates for VLYs, it is difficult to draw such a conclusion with confidence.

Future form of the indicator

In future Lancet Countdown reports this indicator will be expanded to estimate the value of YLLs of additional countries and regions.

Additional analysis

Table 15 presents average annual economic value of YLLs, if pollution were to be held at the levels experienced in the years indicated, across the remaining lifetime of the population alive in those years. Results for 2015 have altered slightly from those presented in 2019 report, due to updated energy production and consumption statistics. Results for both years have been converted to US$ using the 2019 official exchange rate.

Table 15: Average annual economic value of YLL for each EU country, 2015 and 2018.

Country Code	Country name	2015 Pollution Levels	2018 Pollution Levels
AUT	Austria	$1.70 billion	$1.62 billion
BEL	Belgium	$3.47 billion	$3.24 billion
BGR	Bulgaria	$1.86 billion	$1.76 billion
CYP	Cyprus	$0.22 billion	$0.21 billion
CZE	Czechia	$2.78 billion	$2.59 billion
DEU	Germany	$18.02 billion	$16.92 billion
DNK	Denmark	$0.96 billion	$0.87 billion
ESP	Spain	$9.49 billion	$8.99 billion
EST	Estonia	$0.19 billion	$0.18 billion
FIN	Finland	$0.70 billion	$0.67 billion
FRA	France	$14.02 billion	$13.09 billion
GBR	United Kingdom	$10.83 billion	$9.70 billion
GRC	Greece	$3.33 billion	$3.07 billion
HRV	Croatia	$1.21 billion	$1.08 billion
HUN	Hungary	$3.18 billion	$2.96 billion
IRL	Ireland	$0.48 billion	$0.45 billion
ITA	Italy	$20.19 billion	$18.71 billion
LTU	Lithuania	$0.70 billion	$0.66 billion
LUX	Luxembourg	$0.13 billion	$0.13 billion
LVA	Latvia	$0.40 billion	$0.38 billion
MLT	Malta	$0.07 billion	$0.06 billion
NLD	Netherlands	$3.93 billion	$3.61 billion
POL	Poland	$14.72 billion	$13.58 billion
PRT	Portugal	$1.81 billion	$1.71 billion
ROU	Romania	$7.00 billion	$6.52 billion
SVK	Slovakia	$1.57 billion	$1.45 billion
SVN	Slovenia	$0.55 billion	$0.53 billion
SWE	Sweden	$0.97 billion	$0.91 billion
**TOTAL**		**$124.47** billion	**$115.67** billion

4.2: The Economics of the Transition to Zero-Carbon Economies

Indicator 4.2.1: Investment in New Coal Capacity

Methods

The data for investment in new coal-fired power plants is sourced from the IEA World Energy Investment.

'Investment' is defined as ongoing capital spending on assets. For investment in new coal-fired power plants this investment is spread out evenly from the year in which a new plant or upgrade of an existing one begins its construction to the year in which it becomes operational. This definition applies to 2017 data onwards, and differs from the definition previously employed by the IEA, in which investment was defined as overnight capital expenditure.

Data reported in previous Lancet Countdown reports for global investment may have been updated with improved data. As investment in new coal capacity in South Africa was zero in 2006, a low positive value was entered to allow an index for future years to be calculated. Actual data cannot be reported for confidentiality reasons.

Data

1. IEA World Energy Investment 2020.[219]

Caveats

Other areas of expenditure, including operation and maintenance, research and development, financing costs, mergers and acquisitions or public markets transactions, are not included. Investment estimates are derived from IEA data for energy demand, supply and trade, and estimates of unit capacity costs, For more information, see IEA (2020).[219]

Additional analysis

Table 16: Annual investment in new coal-fired power capacity 2006-2019 (an index score 100 corresponds to 2006 levels)

	2006	2007	2008	2009	2010	2011	2012	2013	2014	2015	2016	2017	2018	2019
**Global**	100	106	111	118	124	126	123	115	106	96	88	80	77	72
**China**	100	95	91	84	79	78	78	78	75	66	54	44	35	26
**United States**	100	121	130	120	89	57	29	7	2	0	0	0	0	5
**EU**	100	128	148	172	216	248	248	220	196	152	96	72	76	76
**India**	100	185	254	376	466	436	371	290	231	203	188	180	188	190
**Japan**	100	93	113	133	113	100	107	80	67	107	140	147	147	127
**Korea**	100	89	56	28	31	89	144	142	131	117	97	50	25	22
**Russia**	100	167	200	233	367	500	500	467	400	467	500	600	800	933
**Southeast Asia**	100	85	122	181	285	385	367	348	326	296	315	348	363	352
**South Africa**	100	500	1000	1000	1000	1000	1000	1000	1000	900	1100	1300	1200	1200
**Brazil**	100	150	250	300	300	250	200	100	0	50	50	50	50	50

Indicator 4.2.2: Investments in Zero-Carbon Energy and Energy Efficiency

Methods

The data for this indicator is sourced from the annual IEA World Energy Investment publication.[219] Five categories of investment are defined:

Hydropower – Investment in small, large and pumped-hydropower.

Renewables & Nuclear – investment in all (non-hydro) renewable and nuclear electricity generation, and renewable transport and heating (including biofuels and solar thermal heating)

Energy Efficiency – See below

Electricity Networks & Storage – investment in electricity transmission and distribution infrastructure, and battery storage (excludes pumped-hydro).

Fossil Fuels – including oil, gas and coal, upstream mining, drilling and pipeline infrastructure, and coal, gas and oil power and other fossil fuel-based energy generation capacity.

For most sectors, 'investment' is defined as ongoing capital spending on assets. For some sectors, such as power generation, this investment is spread out evenly from the year in which a new plant or upgrade of an existing one begins its construction to the year in which it becomes operational. For other sources, such as upstream oil and gas and liquefied natural gas (LNG) projects, investment reflects the capital spending incurred over time as production from a new source ramps up or to maintain output from an existing asset. This definition and differs from the definition previously employed by the IEA before 2019, in which investment was defined as overnight capital expenditure.

Data

1. IEA World Energy Investment 2020.[219]

Caveats

Other areas of expenditure, including operation and maintenance, research and development, financing costs, mergers and acquisitions or public markets transactions, are not included. Investment estimates are derived from IEA data for energy demand, supply and trade, and estimates of unit capacity costs, For more information, see IEA (2020).[219]

Additional analysis

Values presented below are in US$2019, billion.

Table 17: Energy investments 2014-2019.

	**2014**	**2015**	**2016**	**2017**	**2018**	**2019**
Fossil Fuels	1,391	1,150	972	978	975	976
Hydropower	55	56	52	51	53	52
Other Renewables & Nuclear	273	290	301	303	296	306
Electricity Networks & Storage	282	298	309	301	299	277
Energy Efficiency	355	239	265	251	252	249
**Total**	2,356	2,032	1,899	1,883	1,876	1,860

Indicator 4.2.3: Employment in Low-Carbon and High-Carbon Industries

Methods

The data for this indicator is sourced from IRENA (renewables)[220] and IBISWorld (fossil fuel extraction).[221,222] Renewable industries included are:

- Hydropower;
- Solar heating/cooling;
- Solar photovoltaic;
- Wind energy;
- Bioenergy;
- Other technologies.

Bioenergy includes liquid biofuels, soil biomass and biogas. ‘Other technologies’ includes geothermal energy, ground-based heat pumps, concentrated solar power, municipal and industrial waste, and ocean energy. Fossil fuel extraction values include direct employment, whereas renewable energy jobs include direct and indirect employment (e.g. equipment manufacturing), except for large hydropower (direct employment only).

Due to an improvement in data collection and estimation methodology, employment values reported for fossil fuel extraction are in some years substantially higher than those reported in the 2018 Lancet Countdown report.[7] Similarly, an improvement to the methodology for estimating hydropower has altered historic values for Hydropower (previously called ‘large’ hydropower), and Other Technologies (which previously included small hydropower). Since 2018, ‘Other Technologies’ includes employment related to ground-based heat pumps.

Data

1. Data for employment in renewables from IRENA.[220] Data is available through to 2019.
2. Data for employment in fossil fuel extraction from IBISWorld: oil and gas exploration and production; and coal mining.[221,222] Data is available through to 2019.

Caveats

Fossil fuel extraction values include direct employment, whereas renewable energy jobs include direct and indirect employment (e.g. equipment manufacturing).

Additional analysis

Table 18: Employment in renewable energy and fossil fuel extraction industries.

	Million Jobs							
	2012	2013	2014	2015	2016	2017	2018	2019
Hydropower	1.66	2.21	2.04	2.16	2.06	1.99	2.05	1.96
Other Technologies	0.22	.023	0.19	0.2	0.24	0.16	0.18	0.13
Solar Heating/Cooling	0.89	0.5	0.76	0.94	0.83	0.81	0.8	0.82
Wind Energy	0.75	0.83	1.03	1.08	1.16	1.15	1.16	1.17
Bioenergy	2.4	2.5	2.99	2.88	2.74	3.06	3.18	3.58
Solar Photovoltaic	1.36	2.27	2.49	2.77	3.09	3.37	3.61	3.76
Fossil Fuel Extraction	13.94	12.24	12.49	12.40	12.37	12.42	13.05	12.66

Bioenergy includes liquid biofuels, solid biomass and biogas. ‘Other technologies’ includes geothermal energy, ground-based heat pumps (in EU countries), concentrated solar power, municipal and industrial waste, and ocean energy. Fossil fuel extraction values include direct employment, whereas renewable energy jobs include

direct and indirect employment (e.g. equipment manufacturing), except for hydropower (direct employment only).

Fossil fuel extraction data slightly updated for all years.

Indicator 4.2.4: Funds Divested from Fossil Fuels

Methods

The data for this indicator is collected and provided by 350.org.[223] They represent the total assets (or assets under management, AUM) for institutions that have publicly committed to divest (for which data is available), with non-US$ values converted using the market exchange rate when the commitment was made, and thus do not directly represent the actual sums divested from fossil fuel companies. A company is committed to 'divestment' if it falls into any of the following five categories:

- 'Fossil Free' - An institution or corporation that does not have any investments (direct ownership, shares, commingled mutual funds containing shares, corporate bonds) in fossil fuel companies (coal, oil, natural gas) and committed to avoid any fossil fuel investments in the future

- 'Full' - An institution or corporation that made a binding commitment to divest (direct ownership, shares, commingled mutual funds containing shares, corporate bonds) from any fossil fuel company (coal, oil, natural gas).

- 'Partial' - An institution or corporation that made a binding commitment to divest across asset classes from some fossil fuel companies (coal, oil, natural gas), or to divest from all fossil fuel companies (coal, oil, natural gas), but only in specific asset classes (e.g. direct investments, domestic equity).

- 'Coal and Tar Sands' - An institution or corporation that made a binding commitment to divest (direct ownership, shares, commingled mutual funds containing shares, corporate bonds) from any coal and tar sands companies.

- 'Coal only' - An institution or corporation that made a binding commitment to divest (direct ownership, shares, commingled mutual funds containing shares, corporate bonds) from any coal companies.

Eight organisations that were originally recorded as non-healthcare institutions have been considered as such for the purpose of this indicator (London School of Hygiene and Tropical Medicine, The Royal College of General Practitioners, New Zealand Nurses Organisation, HESTA, HCF, Berliner Ärzteversorgung, Doctors for the Environment Australia, and the Royal College of Emergency Medicine). Divestment commitments by the American Medical Association, which divested in 2018, was not included in the data provided by 350.org, and was added separately.

Data

1. 350.org Divestment Commitments dataset.[223]

Caveats

Data on the number of institutions that have divested and the value of their assets is dependent on institutions reporting this information to 350.org.

Additional analysis

The cumulative value of divestment (both global total and for healthcare institutions) is presented below (Table 19). Organisations that have divested but for which no date of divestment has been recorded (a total of $504.42 million) are recorded in a separate column, with the total assumed to begin in 2008.

Table 19: Cumulative fossil fuel divestment.

	**US$ billion (current prices)**		
	**Global**	**Global (including data with no divestment date)**	**Healthcare Institutions**
2008	$ 0.00	$ 504.42	$ -
2009	$ 0.00	$ 504.42	$ -
2010	$ 0.00	$ 504.42	$ -
2011	$ 0.09	$ 504.51	$ -
2012	$ 2.11	$ 506.53	$ -
2013	$ 16.13	$ 520.54	$ -
2014	$ 303.46	$ 807.87	$ 27.82
2015	$ 2,997.82	$ 3,502.24	$ 27.94
2016	$ 4,079.53	$ 4,583.95	$ 30.34
2017	$ 5,366.86	$ 5,871.27	$ 41.04
2018	$ 7,502.16	$ 8,006.57	$ 41.90
2019	$ 11,513.63	$ 12,018.05	$ 41.92

Due to confidentiality issues, the full dataset is not available for publication. However, interested readers may visit the 350.org website for further information.

Indicator 4.2.5: Net Value of Fossil Fuel Subsidies and Carbon Prices

Methods

Fossil Fuel Subsidies

Data for fossil fuel subsidies was taken from two sources. The IEA provides data on fossil fuel consumption subsidies for 42 countries, calculated using its 'price gap' approach – the difference between the end-user prices paid for fossil fuels in the country, and reference prices that account for the full cost of supply.[224] However, the countries provided in this list are mainly non-OECD. The OECD itself provides estimates of fossil fuel subsidies within the OECD countries, plus Argentina, Brazil, China, Colombia, India, Indonesia, Russia and South Africa - a total of 43 countries.[225] The OECD's estimates are derived from a bottom-up inventory of subsidy mechanisms within each country, and include production and consumption support, infrastructure investments, incentives and R&D. It divides the type of support into three broad categories: Consumer Support Estimate (CSE), Producer Support Estimate (PSE) and General Services Support Estimate (GSE).

Combining the IEA and OECD datasets allows a coverage of 75 countries (after accounting for overlaps, and the removal of two countries – Taipei and Libya – due to inconsistencies in their representation across other datasets used by this indicator). These countries accounted for 92% of global CO_2 emissions in 2016.[224]

The OECD describes an approach for combining these two datasets, and reconciling different estimates for the countries covered by both.[226] This involves selecting line items in the OECD inventory that correspond to the price-gap definition of subsidies that is the basis of the IEA data – i.e. measures that bring about reduced consumer prices: 'conceptually, an OECD estimate derived from individual measures that capture transfers to consumers from producers and taxpayers should match the IEA price-gap estimates' (p.22-3).[226]

The OECD suggests that the price gap approach may not capture ‘all the transfers generated by other policies that also confer benefits to consumers, such as direct budgetary transfers to consumers or reduced excise taxes, or policies that provide support to the production of fossil fuels without directly affecting end-user prices’ (p. 21), also adding that ‘IEA data do not capture support to producers of fossil fuels…’ (p. 22). As such it is suggested that the OECD inventory ‘casts a wider net and thus complements the IEA data on fossil fuel subsidies’ (p. 21). The implication of this comparison is that in the few cases of countries whose subsidies have been calculated by both OECD and IEA, the OECD estimate would be expected to be the larger of the two.[226]

A comparison of the small number of countries that have been covered by both datasets enables some assessment of the differences between the approaches, and what, if anything, can be done to adjust the OECD dataset to make it more consistent with the IEA approach. OECD notes that ‘finding the price-gap equivalent necessitates identifying which of the measures in the *Inventory* translate into reduced consumer prices’.[226] One way of identifying such measures could be to use the category ‘Consumer Price Estimate’. However, this equivalence is not completely straightforward, as some producer support measures could feed through to lower consumer prices, depending on the context – for example the structure of the producing company and whether it would pass through the effect of the support in lower prices, and whether or not the producer was a dominant supplier to its domestic market.

The OECD lists a number of reasons why, even after attempting to make adjustments to the OECD *Inventory* accounts, discrepancies between the two datasets may remain. These include that some measures affecting fuel prices may not have been included in the *Inventory*; measurement errors; differences in definitions of what constitutes support; and ‘reporting or time lags for fuel price pass-through’ (p. 23).[226] To resolve any remaining discrepancies between IEA and OECD estimates for overlapping countries, the OECD proposes a rule of thumb based on summing the estimates for the years 2010 to 2015, and selecting the larger of the two estimates for consumer price support for the combined dataset.

As part of the preparatory analysis for this indicator, the OECD’s rule of thumb was followed for the countries which are included in both sets, in order to select which of the two estimates should be used for each of these countries – according to the rule of thumb the larger of the two estimates over the cumulative period should be selected – as well as to investigate the differences between the datasets more broadly. Reflecting the availability of new data, the cumulative period analysed was expanded to 2010-2017. The results are shown in Figure 52 below.

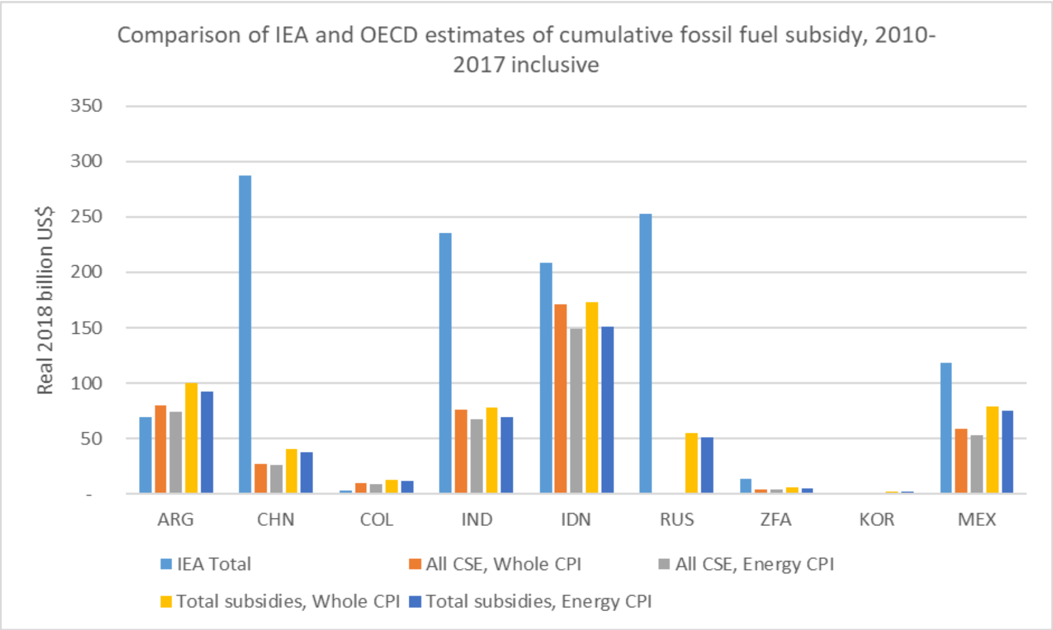

Figure 52: Comparison of the IEA ‘price gap’ subsidy estimate with estimates based on the OECD Inventory, for CSE-only subsidies, and for total subsidies. Prices are given in real 2018 billion US$, with both OECD estimates uprated by two different deflators, whole CPI and energy only CPI. The nine countries are those covered by both datasets.

For each of the nine overlapping countries, Figure 52 above compares the IEA total ‘price gap’ subsidy estimate, with the sum of the OECD’s CSE category only, as well as with the total listed OECD Inventory subsidies (CSE, PSE and GSE). Each of these OECD totals is shown converted to 2018 dollars using a slightly different deflator index, one based on the standard CPI, and another on the energy only components of the CPI. The IEA data is reported in real 2018 US$ only.

Figure 52 shows that in the case of six of the nine overlapping countries - China, India, Indonesia, Russia, South Africa and Mexico – the cumulative estimate of the IEA is greater – in several cases substantially so. This is an interesting result, insofar as it is contrary to the expectation of the OECD approach as casting ‘a wider net’ than the price gap approach. In any case, following the OECD ‘rule of thumb’, this indicator accordingly uses the IEA data for these six countries.

A further observation from this Figure, that informs the approach taken for this indicator, is that in general the selection from the OECD inventory of items in the CSE is not effective in rendering the inventory total closer to that given by the IEA’s price gap approach. This is only the case for Argentina and Colombia, where the CSE totals are slightly closer to the IEA estimate than the total Inventory subsidies. However, in each other case the total OECD estimate is closer to the IEA than the CSE estimates only. This is particularly noticeable in the case of Korea, where no subsidies are recorded as CSE, and in Russia, where the CSEs are very low compared to the Producer Support Estimates, and a great deal lower than the IEA total. It is possible that policies and measures directed at producers could affect the fuel price experienced by consumers, if as a result of the policies producers sell into domestic markets at lower prices. It seems that in several of the cases illustrated in Figure 52, such an effect may be taking place.

These observations in these limited cases of countries that are covered by both datasets, do not strongly support the approach of selecting only consumer focussed measures from the OECD Inventory as a means of rendering it more consistent with the IEA price gap approach. Rather it seems that in most cases the datasets would be more aligned by selecting the country totals, including PSE, CSE and GSE categories, from the OECD Inventory. As such this was the approach followed for this indicator for all of the countries covered only by the OECD Inventory, as well as the three overlapping countries – Argentina, Colombia and Korea – for which the OECD Inventory cumulative 2010-2017 total is larger than the IEA total. The remaining countries use the IEA data.

The resulting combined subsidy dataset is summarised in Table 20 below, which also indicates whether the estimate is based on IEA or OECD data.

Table 20: Table: Fossil fuel subsidies in 75 countries, 2016 and 2017.

			Subsidies (Real 2018 billion US$)	
**Dataset**	**Country name**	**ISO ALPHA-3 code**	**2016**	**2017**
OECD	Australia	AUS	1.133	0.963
OECD	Austria	AUT	0.818	0.877
OECD	Belgium	BEL	0.224	0.268
OECD	Canada	CAN	0.005	0.007
OECD	Chile	CHL	0.002	0.002
OECD	Czechia	CZE	0.067	0.000
OECD	Denmark	DNK	0.192	0.240
OECD	Estonia	EST	0.045	0.047
OECD	Finland	FIN	0.001	0.001
OECD	France	FRA	0.084	0.000
OECD	Germany	DEU	0.266	0.266
OECD	Greece	GRC	1.214	1.059
OECD	Hungary	HUN	0.259	0.275

OECD	Iceland	ISL	0.000	0.000
OECD	Ireland	IRL	0.376	0.384
OECD	Israel	ISR	0.133	0.141
OECD	Italy	ITA	1.344	0.645
OECD	Japan	JPN	2.437	1.863
OECD	Republic of Korea	KOR	0.254	0.265
OECD	Latvia	LVA	0.136	0.153
OECD	Lithuania	LTU	0.099	0.103
OECD	Luxembourg	LUX	0.002	0.002
IEA	Mexico	MEX	10.832	11.749
OECD	Netherlands	NLD	0.177	0.173
OECD	New Zealand	NZL	0.043	0.041
OECD	Norway	NOR	1.111	0.984
OECD	Poland	POL	1.004	0.589
OECD	Portugal	PRT	0.284	0.451
OECD	Slovakia	SVK	0.111	0.111
OECD	Slovenia	SVN	0.054	0.053
OECD	Spain	ESP	1.296	1.374
OECD	Sweden	SWE	2.105	2.067
OECD	Switzerland	CHE	2.595	2.491
OECD	Turkey	TUR	0.792	0.633
OECD	United Kingdom	GBR	13.868	12.432
OECD	United States of America	USA	2.438	2.424
OECD	Argentina	ARG	15.148	7.937
OECD	Brazil	BRA	1.308	1.623
IEA	China	CHN	43.734	40.047
OECD	Colombia	COL	1.032	1.317
IEA	India	IND	15.039	14.492
IEA	Indonesia	IDN	18.278	18.836
IEA	Russian Federation	RUS	33.368	21.250
IEA	South Africa	ZAF	6.014	5.324
IEA	Algeria	DZA	7.594	10.009
IEA	Angola	AGO	0.530	0.223
IEA	Azerbaijan	AZE	1.500	2.054
IEA	Bahrain	BHR	1.243	1.423
IEA	Bangladesh	BGD	1.091	1.404
IEA	Bolivia	BOL	0.678	0.881
IEA	Brunei Darussalam	BRN	0.104	0.181
IEA	Ecuador	ECU	1.464	2.372
IEA	Egypt	EGY	7.922	19.425
IEA	El Salvador	SLV	0.258	0.367
IEA	Gabon	GAB	0.142	0.131
IEA	Ghana	GHA	0.029	0.115
IEA	Iraq	IRQ	5.687	7.680
IEA	Islamic Republic of Iran	IRN	31.179	48.662
IEA	Kazakhstan	KAZ	4.863	5.434
IEA	Kuwait	KWT	6.892	6.894
IEA	Malaysia	MYS	1.553	2.085
IEA	Nigeria	NGA	0.054	0.962

IEA	Oman	OMN	0.118	0.128
IEA	Pakistan	PAK	1.707	3.471
IEA	Qatar	QAT	1.326	1.650
IEA	Saudi Arabia	SAU	38.947	44.605
IEA	Sri Lanka	LKA	0.075	0.195
IEA	Thailand	THA	0.551	0.864
IEA	Trinidad and Tobago	TTO	0.610	0.663
IEA	Turkmenistan	TKM	3.862	4.098
IEA	Ukraine	UKR	2.460	2.131
IEA	United Arab Emirates	ARE	8.168	8.422
IEA	Uzbekistan	UZB	1.668	3.498
IEA	Bolivarian Republic of Venezuela	VEN	7.472	15.459
IEA	Vietnam	VNM	0.107	0.471
	**TOTAL**		**319.580**	**349.914**

According to this data, total fossil fuel subsidies were $320bn in 2016. This figure is consistent with the OECD's estimates for subsidies based on their combination of both datasets, for 2014 and 2015, at $551bn and $373bn, respectively.[226]

Carbon prices and revenues

Information on carbon prices and carbon pricing revenues was sourced from the World Bank Carbon Pricing Dashboard.[227] Table 21 shows the data on revenues from carbon pricing instruments provided by this source. Revenues were allocated to the nation state within which the instrument operated, as also shown in Table 21. Shares of the EU ETS revenues were allocated to any of the 75 countries included in this indicator that also were participants in the EU ETS, on the basis of their share of the emissions of all EU ETS states, calculated using IEA CO_2 emissions data.[133] This was considered an acceptable simplification given that for the period 2013-2020 88% of allowances are allocated for auction to participating states in proportion to their emissions.[228]

Table 21: Carbon pricing revenues by instrument in 2016 and 2017

Name of the initiative	Instrument Type	Jurisdiction Covered	Nation state	2016 (billion US$ nominal)	2017 (billion US$ nominal)
Alberta CCIR	ETS	Alberta	Canada	155.15	72.86
Alberta carbon tax	Carbon tax	Alberta	Canada	0.00	714.62
Australia ERF Safeguard Mechanism	ETS	Australia	Australia	0.00	0.00
BC GGIRCA	ETS	British Columbia	Canada	0.00	0.00
BC carbon tax	Carbon tax	British Columbia	Canada	910.97	965.94
Beijing pilot ETS	ETS	Beijing	China	0.00	0.00
California CaT	ETS	California	United States	901.10	2025.72
Chile carbon tax	Carbon tax	Chile	Chile	0.00	167.91
Chongqing pilot ETS	ETS	Chongqing	China	0.00	0.00
Colombia carbon tax	Carbon tax	Colombia	Colombia	0.00	171.53
Denmark carbon tax	Carbon tax	Denmark	Denmark	521.91	621.45
EU ETS	ETS	EU, Norway, Iceland, Liechtenstein	EU ETS	4056.02	6849.57

Estonia carbon tax	Carbon tax	Estonia	Estonia	2.21	2.40
Finland carbon tax	Carbon tax	Finland	Finland	1436.51	1660.59
France carbon tax	Carbon tax	France	France	4062.59	6742.03
Fujian pilot ETS	ETS	Fujian	China	0.51	0.00
Guangdong pilot ETS	ETS	Guangdong (except Shenzhen)	China	1.56	2.51
Hubei pilot ETS	ETS	Hubei	China	0.00	0.00
Iceland carbon tax	Carbon tax	Iceland	Iceland	31.56	37.24
Ireland carbon tax	Carbon tax	Ireland	Ireland	459.71	520.72
Japan carbon tax	Carbon tax	Japan	Japan	2340.92	2486.73
Kazakhstan ETS	ETS	Kazakhstan	Kazakhstan	0.00	0.00
Korea ETS	ETS	Korea, Republic of	South Korea	2.30	0.00
Latvia carbon tax	Carbon tax	Latvia	Latvia	6.35	9.74
Liechtenstein carbon tax	Carbon tax	Liechtenstein	Liechtenstein	6.40	6.66
Mexico carbon tax	Carbon tax	Mexico	Mexico	440.41	624.45
New Zealand ETS	ETS	New Zealand	New Zealand	0.00	0.02
Norway carbon tax	Carbon tax	Norway	Norway	1408.21	1633.41
Ontario CaT	ETS	Ontario	Canada	0.00	1491.23
Poland carbon tax	Carbon tax	Poland	Poland	1.14	1.33
Portugal carbon tax	Carbon tax	Portugal	Portugal	143.49	170.89
Quebec CaT	ETS	Quebec	Canada	336.06	477.63
RGGI	ETS	RGGI	United States	266.47	198.38
Saitama ETS	ETS	Saitama	Japan	0.00	0.00
Shanghai pilot ETS	ETS	Shanghai	China	0.00	0.25
Shenzhen pilot ETS	ETS	Shenzhen	China	0.00	0.00
Slovenia carbon tax	Carbon tax	Slovenia	Slovenia	144.01	175.68
Spain carbon tax	Carbon tax	Spain	Spain	101.56	148.78
Sweden carbon tax	Carbon tax	Sweden	Sweden	2702.10	2846.39
Switzerland ETS	ETS	Switzerland	Switzerland	4.01	4.94
Switzerland carbon tax	Carbon tax	Switzerland	Switzerland	1061.94	1173.01
Tianjin pilot ETS	ETS	Tianjin	China	0.00	0.00
Tokyo CaT	ETS	Tokyo	Japan	0.00	0.00
UK carbon price floor	Carbon tax	United Kingdom	United Kingdom	1168.88	1241.22
Ukraine carbon tax	Carbon tax	Ukraine	Ukraine	3.24	3.71
Washington CAR	ETS	Washington	United States	0.00	0.00
**TOTAL**				**22,677.30**	**33,249.54**

Net carbon price and revenue calculations

In reality at present, both carbon prices and fossil subsidies are typically applied to individual sectors or fuels, and do not cover the entire economy. Within different particular jurisdictions the sectors covered by subsidies and carbon prices are often not identical. As such the only way of producing a consistent indicator across multiple countries was to average out both subsidies and prices across the CO_2 emissions of the whole economy, resulting in net average economy-wide carbon prices and revenues. Each country's total fossil fuel subsidies, and total carbon price revenues, were divided by the relevant total CO_2 emissions for each year, using data from the IEA[133] resulting in a subsidy per tonne of CO_2, and a carbon price per tonne of CO_2. The first was subtracted from the second to provide the net carbon price. The total subsidies were also subtracted from the total revenues of each country and year, in order to calculate net revenue, which may also be expressed as a proportion of national expenditure on health, using current annual (i.e. not including capital) health expenditure data from the World

Health Organization's Global Health Expenditure Database.[229] All data points for each country are presented under 'additional analysis', below.

Currency standardisation

All money values are expressed in real US$ (2018). The OECD Inventory presents data in nominal local currency units. These units were first converted to nominal US dollars using the official exchange rate annual data provided by the World Bank World Development Indicators,[215] and then corrected to real 2018 values, using GDP deflator for the US dollar, from the same source. The IEA subsidy data is given in real US$ 2018. The World Bank carbon pricing revenue data is given in nominal US dollars, so again the World Bank's US GDP deflator was applied. The WHO health expenditure data is given in Real US$ 2017, which are again uprated to US$ 2018.

Data

1. Fossil fuel subsidies data from the IEA and OECD.[224,225]
2. Carbon pricing data from the World Bank Carbon Pricing Dashboard.[227]

Caveats

The principal caveat is that the indicator is strongly dependent on the reliability of the main datasets from the IEA, OECD and World Bank. It is possible that data on individual countries may not be fully comprehensive due to reporting errors, lack of information or other issues, as indeed is acknowledged by OECD.[226] The indicator should be considered as a way of illustrating global trends, and caution should be exercised in attempting to draw out specific conclusions relating to individual countries covered by the indicator.

The indicator replaces two indicators from previous Lancet Countdown reports, in which subsidies and carbon prices were dealt with as separate indicators. The nature of indicators that draw on multiple datasets is that the most recent year on which they can report is defined by the most recent year that is common to all datasets used. In this case that year was 2017; whereas in last year's Lancet Countdown report the separate carbon pricing indicator was able to report to 2018.

The economy-wide net carbon price was derived by dividing fossil fuel subsidies and carbon pricing revenues by total CO_2 emissions. This fits well with the subsidies, as these are for fossil fuels, the principal source of CO_2. However, some of the carbon pricing instruments from which the revenue was assessed are not only for fossil fuel combustion but apply to other sectors and non-CO_2 gases. There was therefore an argument for using total GHGs as the denominator for deriving the net price. However, the problem here is data. The most recent estimates for country-level greenhouse gas emissions provided by the Emissions Database for Global Atmospheric Research EDGAR extends only to 2015.[230]

Additionally, at present, both carbon prices and fossil subsidies are typically applied to individual sectors or fuels, and do not cover the entire economy. Within different jurisdictions, the sectors covered by subsidies and carbon prices are often not identical. As such the only way of producing a consistent indicator across multiple countries was to average out both subsidies and prices across the CO_2 emissions of the whole economy, resulting in net average economy-wide carbon prices and revenues.

Additional analysis

Table 22 below presents the net carbon price, net carbon revenue and net carbon revenue as a percentage of current health expenditure, for 2016 and 2017 for each of the 75 countries considered. This data is the basis of the aggregated figures presented in the main text.

Table 22: Net carbon price, net carbon revenue, and net carbon revenue as a percentage of current health expenditure for 75 countries, 2016 and 2017.

	Net carbon price (Real 2018 US$ / tonne CO2)		Net Carbon Revenue (Real 2018 billion US$)		Net carbon revenue as % of current health expenditure	
Country name	2016	2017	2016	2017	2016	2017
Australia	-2.968	-2.504	-1.133	-0.963	-0.874	-0.721
Austria	-11.882	-11.360	-0.737	-0.737	-1.699	-1.660
Belgium	-1.129	-0.803	-0.104	-0.073	-0.202	-0.138
Canada	2.661	6.949	1.459	3.806	0.829	2.134
Chile	-0.020	1.974	-0.002	0.170	-0.007	0.665
Czechia	0.641	2.161	0.065	0.220	0.428	1.374
Denmark	11.857	14.835	0.397	0.464	1.180	1.358
Estonia	-1.424	-0.606	-0.022	-0.010	-1.302	-0.552
Finland	34.510	42.067	1.557	1.792	6.561	7.531
France	15.086	24.722	4.551	7.568	1.530	2.525
Germany	0.945	1.791	0.694	1.287	0.168	0.303
Greece	-17.938	-14.584	-1.131	-0.922	-6.666	-5.511
Hungary	-4.619	-3.835	-0.202	-0.176	-2.061	-1.783
Iceland	17.264	19.713	0.036	0.043	1.863	2.051
Ireland	4.122	6.347	0.152	0.227	0.650	0.930
Israel	-2.115	-2.214	-0.133	-0.141	-0.520	-0.527
Italy	-2.820	0.155	-0.918	0.050	-0.527	0.028
Japan	0.006	0.604	0.007	0.684	0.001	0.126
Republic of Korea	-0.426	-0.441	-0.251	-0.265	-0.225	-0.222
Latvia	-17.721	-19.297	-0.120	-0.129	-6.501	-6.936
Lithuania	-7.885	-7.322	-0.085	-0.079	-2.727	-2.517
Luxembourg	1.035	1.893	0.009	0.016	0.256	0.467
Mexico	-23.248	-24.909	-10.373	-11.109	-15.889	-16.973
Netherlands	0.185	1.051	0.029	0.164	0.034	0.190
New Zealand	-1.401	-1.269	-0.043	-0.041	-0.239	-0.216
Norway	11.494	21.989	0.406	0.764	0.961	1.788
Poland	-2.115	0.240	-0.620	0.073	-1.844	0.208
Portugal	-1.589	-3.273	-0.074	-0.166	-0.374	-0.824
Slovakia	-2.364	-1.279	-0.071	-0.041	-1.059	-0.624
Slovenia	8.416	11.632	0.114	0.156	2.849	3.839
Spain	-3.704	-2.659	-0.879	-0.674	-0.750	-0.564
Sweden	20.130	24.709	0.765	0.930	1.297	1.539
Switzerland	-39.159	-34.579	-1.482	-1.284	-1.772	-1.495
Turkey	-2.339	-1.672	-0.792	-0.633	-2.262	-1.721
United Kingdom	-32.642	-28.951	-12.161	-10.386	-4.725	-3.990
United States of America	-0.252	-0.031	-1.219	-0.146	-0.036	-0.004
Argentina	-79.514	-43.285	-15.148	-7.937	-31.313	-13.214
Brazil	-3.124	-3.796	-1.308	-1.623	-0.682	-0.815
China	-4.825	-4.325	-43.732	-40.045	-7.584	-6.292
Colombia	-11.767	-15.159	-1.032	-1.141	-4.541	-4.945
India	-7.309	-6.705	-15.039	-14.492	-17.071	-15.246
Indonesia	-40.236	-37.946	-18.278	-18.836	-59.193	-60.585
Russian Federation	-22.090	-13.827	-33.368	-21.250	-39.840	-24.590

South Africa	-14.365	-12.626	-6.014	-5.324	-21.073	-18.357
Algeria	-59.493	-76.700	-7.594	-10.009	-67.933	-91.483
Angola	-24.704	-12.351	-0.530	-0.223	-15.595	-6.374
Azerbaijan	-47.761	-66.687	-1.500	-2.054	-53.995	-73.989
Bahrain	-41.925	-47.732	-1.243	-1.423	-73.151	-82.573
Bangladesh	-14.953	-17.934	-1.091	-1.404	-18.862	-22.936
Bolivia	-33.702	-40.236	-0.678	-0.881	-26.647	-35.334
Brunei Darussalam	-16.266	-26.979	-0.104	-0.181	-33.379	-61.400
Ecuador	-41.377	-69.144	-1.464	-2.372	-16.914	-26.883
Egypt	-38.812	-92.844	-7.922	-19.425	-76.989	-183.784
El Salvador	-40.472	-63.948	-0.258	-0.367	-13.389	-19.874
Gabon	-42.256	-38.759	-0.142	-0.131	-30.075	-30.772
Ghana	-2.264	-8.341	-0.029	-0.115	-1.518	-5.830
Iraq	-40.523	-54.908	-5.687	-7.680	-86.467	-93.144
Islamic Republic of Iran	-56.239	-85.804	-31.179	-48.662	-79.971	-123.096
Kazakhstan	-19.076	-21.246	-4.863	-5.434	-88.332	-104.203
Kuwait	-73.964	-77.096	-6.892	-6.894	-136.717	-106.403
Malaysia	-7.184	-9.879	-1.553	-2.085	-13.628	-17.189
Nigeria	-0.643	-11.186	-0.054	-0.962	-0.391	-6.654
Oman	-1.872	-1.956	-0.118	-0.128	-3.731	-4.591
Pakistan	-10.398	-18.921	-1.707	-3.471	-20.295	-38.568
Qatar	-16.772	-20.598	-1.326	-1.650	-25.840	-37.012
Saudi Arabia	-73.918	-83.815	-38.947	-44.605	-93.916	-107.559
Sri Lanka	-3.570	-8.437	-0.075	-0.195	-2.213	-5.715
Thailand	-2.256	-3.537	-0.551	-0.864	-3.265	-4.945
Trinidad and Tobago	-34.930	-36.817	-0.610	-0.663	-38.491	-42.054
Turkmenistan	-55.994	-59.395	-3.862	-4.098	-159.853	-152.208
Ukraine	-12.433	-12.417	-2.457	-2.127	-32.454	-26.468
United Arab Emirates	-42.527	-42.856	-8.168	-8.422	-61.725	-64.450
Uzbekistan	-20.508	-43.106	-1.668	-3.498	-57.106	-108.296
Bolivarian Republic of Venezuela	-58.666	-135.943	-7.472	-15.459	-129.473	-500.849
Viet Nam	-0.555	-2.461	-0.107	-0.471	-0.885	-3.712

Section 5: Public and Political Engagement

Indicator 5.1: Media Coverage of Health and Climate Change

Indicator 5.1.1: Global Coverage of Health and Climate Change

Methods

Intersecting trends in coverage of climate change and health were identified in 61 selected newspaper sources from January 2007 through December 2019. The 61 sources are located across 36 countries, in four languages, and spanning the six World Health Organization (WHO) regions: African Region, Region of the Americas, South-East Asia Region, European Region, Eastern Mediterranean Region, and Western Pacific Region. These sources were monitored through Nexis Uni, Proquest and Factiva databases accessed via the University of Colorado libraries.

The 2020 report of the Lancet Countdown uses a revised search strategy within these three databases, to increase the precision of the indicator; that is, to reduce the number of 'false positives', while retaining the maximum number of 'true positives'. This was done by retaining those terms that a) produced relevant data, and b) had a low degree of polysemy (i.e. words that have fewer meanings *or* words used in fewer disciplines/domains). Testing for interaction *between* terms also enabled fewer terms to be used (for example, it was found that the term 'morbidity' would usually pull in the term 'mortality', when related to humans).

The terms were translated once the strategy had been finalised with certain terms presenting difficulties in translation. The English terms 'hay-fever' and 'West Nile', for example, correlated with more than one term in Spanish and Portuguese and the decision was made to include all relevant terms in the respective search strategies.

For the final strategy, search functions were compared across databases to ensure consistency, as different databases utilise different search filter operators. The searches were conducted with the following key words in English, Spanish, Portuguese and German respectively:

English:

(climate change OR global warming) AND (health OR illness OR epidemiolog* OR malnutrition OR morbidity OR fatalit* OR diarrh* OR malaria OR chikungunya OR west nile OR dengue OR hay-fever OR zika)

German:

(Klimawandel OR Globale Erwärmung) AND (Gesundheit OR Krankheit OR Epidemiolog* OR Mangelernährung OR Morbidität OR Sterblich* OR Durchfall* OR Malaria OR Chikungunya OR West-Nil-Virus OR Dengue-Fieber OR Heuschnupfen OR Zika)

Portuguese:

(mudanças climáticas OR aquecimento global) AND (saúde OR doença OR epidemiologi* OR desnutrição OR morbilidade OR fatalidade* OR diarr* OR malária OR chikungunya OR nilo do oeste OR vírus do nilo OR dengue OR febre dos fenos OR rinite alérgica OR zika)

Spanish:

(cambio climático OR calentamiento global) AND (salud OR enfermedad* OR epidemiología OR epidemiólog* OR desnutrición OR malnutrición OR morbosidad OR muert* OR diarrea* OR malaria OR paludismo OR chikungunya OR nilo del oeste OR nilo occidental OR virus del nilo OR dengue OR fiebre del heno OR rinitis alérgica OR zika)

The signal of the search strategies above was found to be strong enough (over 80% relevance in a systematically randomised sample of 500) to allow a more parsimonious approach to this indicator, requiring no screening of articles during the extraction of the data.

Results were obtained from the databases by entering the relevant search strategy along with the relevant date. Counting occurred month by month and the number of returns for each source was recorded on a Microsoft Excel spreadsheet. Primary counting took place for each source along with a secondary independent count of a systematically randomised 20% sample by another researcher. Tertiary counts were undertaken where any mismatch occurred between primary and secondary counts. All counts were agreed by the whole research team.

Using the Excel spreadsheet constructed through the phases of counting, the data was organised in numerous ways for a better understanding of the patterns in coverage. These included by WHO region, by World Bank Income and Lending Group, and by individual source. The average scores for each month (and aggregated into annual averages) were used as an adjustment for the number of sources selected per region.

Data

1. Three databases were used: Nexis Uni; Proquest; and Factiva databases accessed via the University of Colorado libraries. The 61 newspaper sources are located across 36 countries, in four languages, and spanning the six World Health Organization (WHO) regions.

Caveats

Compared to 62 sources in previous iterations of the Lancet Countdown report, only 61 were used this year with The Globe and Mail now unavailable through Nexis Uni. This will be investigated for the 2021 report with the hope of retaining access to the Globe and Mail.

Remaining concerns about the degree to which the databases return hits of duplicate articles were not found to be warranted (i.e. are not actually the same article reproduced elsewhere but rather are simply two entries in the database for a single article). During the counting phase, a series of tests were undertaken to determine the consistency of numbers across counts. These highlighted small differences in a number of cases, but not enough to change the larger trends in the data. After investigation, these were attributed to the internal functioning of the database. For the 2021 Lancet Countdown report, these will be investigated further.

In developing the search strategy, it was found that a significant portion of articles may mention both climate change and health but do not deeply engage with them as integrated issues. For example, an article could cover an election candidate's political priorities, including a discussion of the health sector and a candidate's response to climate change. However, including this coverage remains important as it brings both sets of issues – health and climate change- onto the public agenda and into public awareness.

Future form of the indicator

In the 2021 report of the Lancet Countdown, this indicator will look to include more sources with a greater distribution across the selected regions, particularly in the World Bank's Low Income and Lending group for which there are currently no sources.

Additional analysis

Geographical distribution of newspaper coverage

Table 23 demonstrates the percentage change in health and climate change coverage in each WHO region from 2007 to 2019. By separating The Americas into component regions, it is clear that North American sources contribute most to its +68.8% percentage change, with all other regions of The Americas observing a decrease in average coverage. The Eastern Mediterranean and the African region, though lowest in absolute terms, see the largest increase in average coverage between 2007 and 2019. Western Pacific starts with the highest absolute numbers in 2007 but sees a percentage decrease of 8.3% by 2019.

*Table 23: percentage change for annual coverage average of health and climate change by WHO region (*The Americas are also broken into component regions)*

Region	2007 annual average	2019 annual average	% change
The Americas	99.8	168	+68.8%
North America*	156.6	389.7	+148.9%
Central/South America and the Caribbean*	60	49.2	-17.9%
African region	40	22.3	-44.2%
South East Asian region	50.5	139.9	+177%
European region	123.2	205.1	+66.5%
Eastern Mediterranean	6.5	51.3	+689.7%
Western Pacific	178.1	163.3	-8.3%

Figure 53 shows the average health and climate change coverage regionalised using WHO regions. The pattern largely follows the overall total trends, with peaks in 2009 (Copenhagen COP) and 2015 (Paris COP) and a dip that flattens out between these. Despite this, important differences between regions are observable. Four regions (The Western Pacific, the European Region, The Americas, and South East Asia) make up the bulk of the coverage. All regions observe an increase in coverage from 2018 to 2019, but steeper increases are seen in the Western Pacific (+114%), the European Region (+128%), The Americas (+93%). Gentler increases are seen in the South East Asian region (+39%), the African region (+22%) and the Eastern Mediterranean region (+20%).

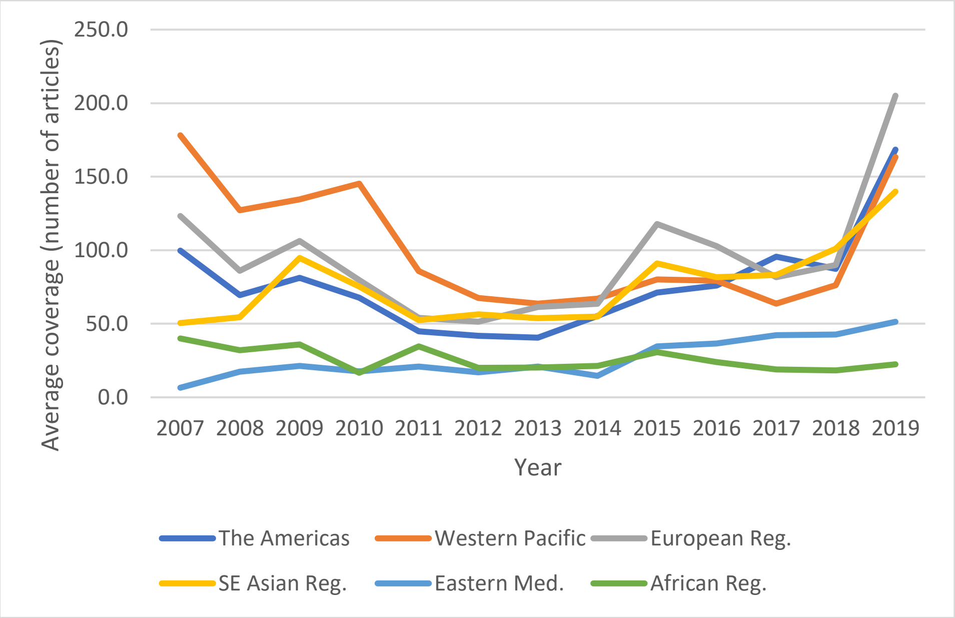

Figure 53: Average annual media coverage by WHO region.

Adjusted for the number of sources in each region, the Western Pacific has the greatest proportion of coverage (26% of total), driven by greater attention in the earlier years of monitoring (2007-2012). This is followed by the European Region (23%), whose coverage has increased in later years of monitoring (2018-2019 in particular). The South East Asian Region and the Region of the Americas followed closely behind (both at 19% of total coverage). The Eastern Mediterranean and the African Region have the lowest proportion of total coverage at 7% and 6%, respectively.

Fluctuations in monthly proportion of coverage can be seen in Figure 54. Each region has its largest proportion of coverage at different times between 2007 and 2019: The Americas is at 30.4% in October 2019; the European Region is at 34.3% in May 2015; the Western Pacific is at 47.5% in November 2007; the South East Asian region is at 32.5% in July 2016; the Eastern Mediterranean Region is at 14.7% in November 2011; and the African Region is at 19.9% in September 2011.

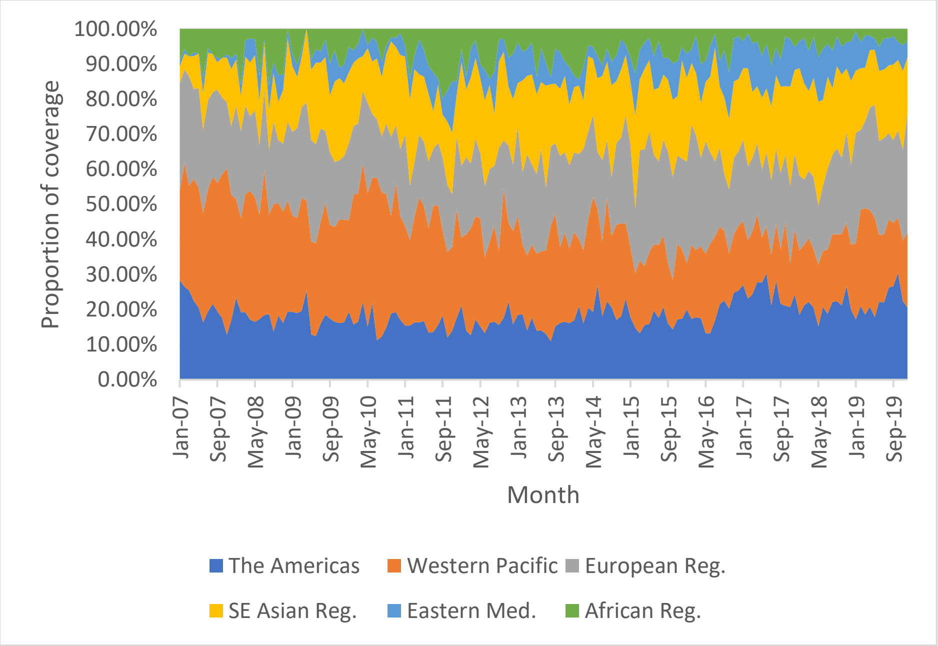

Figure 54: Proportion of average monthly media coverage by WHO region, 2007 to 2019.

Indicator 5.1.2: Coverage of Health and Climate Change in the People's Daily

Methods

For the 2020 report of the Lancet Countdown, the methodology was updated for Indicator 5.1.2 from "searching the keywords on the website and then trawling" to "first trawling all articles and then searching the keywords in the text". The filtration process was also improved by adding score and keywords ratio as filtration criteria. The reasons are as follows:

Firstly, as it is not clear how the search engine of People's Daily website works, the new method improves the transparency of the searching process.

Secondly, the previous method had overestimated the number of articles that fit the search criteria. For example, when different keywords of 'climate change' in the search engine were searched, the website provided duplicate results. The new method will recognise every article that contains the keywords, and make sure every article will only be counted once. This will therefore result in different article numbers than has been documented in previous iterations of the report, due to greater accuracy.

Thirdly, the new method not only improved the degree of accuracy, but also saved more time. The original website with the search engine (http://data.people.com.cn/rmrb/20191231/1) set an anti-trawling system to prevent trawling while searching the keywords. For skipping the system, it took several days to finish searching and trawling. The new method trawls all the articles from the website without the search engine (http://paper.people.com.cn/rmrb/html/2019-12/31/nbs.D110000renmrb_01.htm), so that the analysis could be performed with improved accuracy and less time.

Fourth, the previous use of LDA (Latent Dirichlet Allocation) does not work very well in this case. LDA is known as the method to synthesise keywords in the natural linguistics. It could recognize the keywords of articles automatically. In the analysis for 2019 data, LDA was first used to allocate the topics of all the articles which contain both the "Climate Change" and "Health" keywords. However, the keywords shown after the allocation is not as close to the topics (especially the health one) as expected. Therefore, it was not possible to simply rely on machine learning to filter keywords. The new method uses LDA for a different group of articles. These articles are the "true" articles in 2008-2018 which we manually confirmed their relevance with both climate change and health. The keywords shown in this round of LDA are more relevant to both of the topics and can help to distinguish the supplemental keywords.

The detailed steps of the new method used in 2020 is shown as below:

Step 1: Trawling all the articles from 2008 to 2019

Trawling all articles that were published in "People's Daily" from 2008 to 2019 (http://paper.people.com.cn/rmrb/html/2019-12/31/nbs.D110000renmrb_01.htm)

Step 2 Searching for "Climate Change" topic articles

Using the new method to search articles that contain the keywords in the topic of "Climate Change". The keywords are presented in the first column of Table A5.2. The keywords in the first column of Table 1 are identical to last year's keywords, to ensure comparability with other paper media in the world. The results are shown in Figure 55.

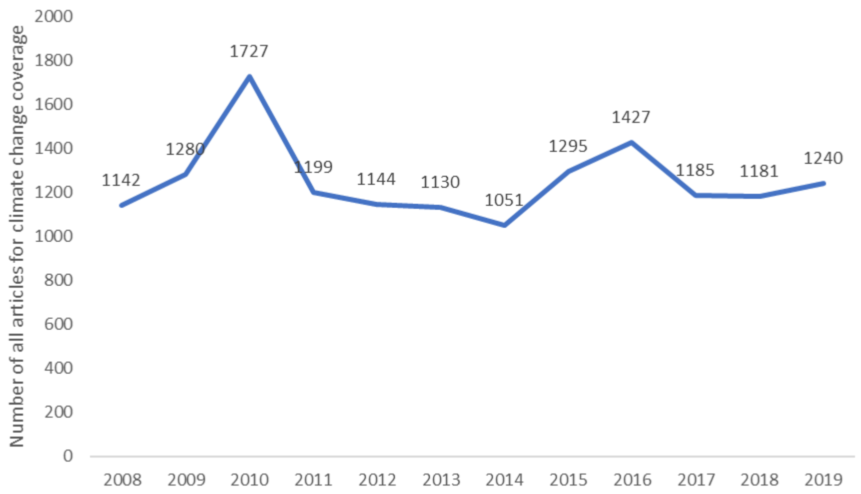

Figure 55: Number of articles identified in People's Daily by searching the keywords from topic Climate Change

Step 3.1 Removing ambiguous keywords from the original keyword list, and adding sub-level keywords and supplementary keywords

First, ambiguous keywords from the original keyword list were removed – pollution. In China, air pollution and health is a hot topic of much discussion. Including "pollution" as a "climate change" keyword would "dilute" results for climate change and health articles. Therefore, "haze", "air pollution" and "atmospheric pollution" have been set as sub-level keywords. It means talking about air pollution and health ONLY is not about climate change and health issue. The pre-requisite for an air pollution-health article to be considered "true" is that it also talks about climate change issues at the same time.

Then, supplemental keywords from the 74 articles were identified based on last year's search. These 74 articles are considered to be the ones that fit the topic "Climate Change and Health". Firstly, "word segmentation" was performed and "stop words" were removed to all the 74 articles as the pre-treatment step for LDA. After these two processes, the sentences in the articles are segmented into words and phrases. Then, the articles were analysed by LDA so that the program could identify the keywords that appears in the 74 articles in both "climate change" and "health" topics. Additionally, a manual screening was completed to ensure important keywords related to "Climate Change" and "Health" (even if they are not high-frequency words) are not missed. Together with the original keyword list (with "pollution" erased), there is a new list of keywords of climate change and health, displayed in the second, third and fifth column of Table 24. Important new keywords include high temperature, heat wave, storm, flood, wildfire, interdecadal, ocean acidification, dengue, food, mask, survive.

The reason for identifying supplementary keywords is that the identification of the topic in an article by humans and by machines is different. Humans are able to identify the real topic of an article by reading it, but a machine can only think of the high-frequency words as the theme or topic of the article. So, the keywords selected by humans could be an important supplement for a machine to better identify the target articles.

Step 3.2 Adding mis-hit keywords

This year mis-hit keywords have been added during the search, listed in column 6 of Table 24. The aim is to increase the accuracy of machine filtration. Before adding mis-hit keywords, several articles that talk about "healthy development" of the economy and "coral death" are identified by machine as "true". So, adding these mis-hit keywords help to identify keywords.

Step 4 Identifying articles that have both climate change and health keywords (first-round search)

Based on the new keyword, sub-level and mis-hit keyword list in column 2 and 3 and 5 and 6 (Table 24), a first-round filtration, which aims to identify articles that have both climate change and health keywords were completed. The results are the basis for the second-round search in step 5.

Step 5 Machine filtration on the results from step 4 by score and ratio (second-round search)

The articles obtained from step 4 were first scored based on the times of appearance of the keywords shown in the articles. For example, if the keywords of climate change and health have appeared 12 times in one article, then the score for this article is 12. If the keyword found is one of the "mis-hit words", the appearance will not be counted as one score. At the same time, the ratio of times of appearance of the keywords to the total number of characters in the article (short for "the ratio" thereafter) is also calculated. When the score and the ratio of one article are both higher than the manually-set thresholds, the article will be considered as relevant articles for health and climate change. Via this step, the numbers of relevant articles are illustrated by the grey line in Figure 56.

Step 6: Manual screening of the results after machine filtration

The sixth step was manually screening the filtered articles. If the manual screening confirmed that the topic is Health and Climate Change, it is retained. The orange line in Figure 56 shows the number of articles that passed the manual screening.

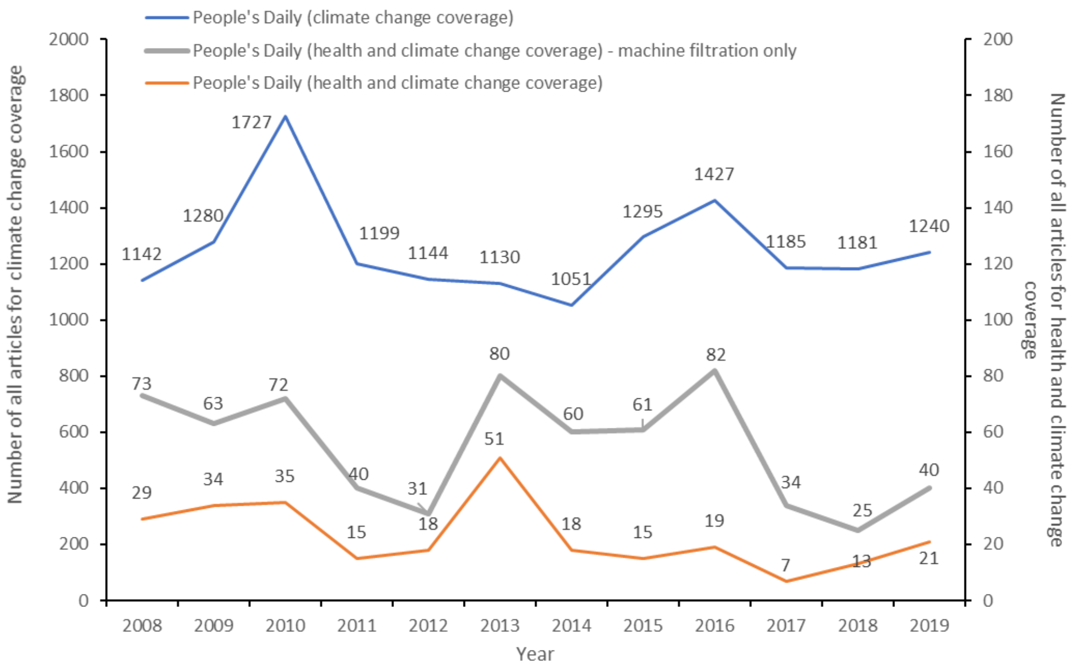

Figure 56: Number of articles for climate change and health coverage 2008-2019. Numbers of all articles for climate change only (blue line), for both health and climate change after machine filtration only (grey line), and for both health and climate change after machine filtration and manual screening (orange line).

Parameter setting:

In the new method, the important parameters and their values are shown as below:

1. LDA parameters in step 3.1 -- Number of topics and Number of keywords under each topic. The number of topics is set to be 3 and the number of keywords under each topic is set to be 20.

When doing LDA analysis to the 74 articles, different settings of the parameters were tried. The result was the most focused when topic number was set as 3. Also, while the number of keywords were set as 20, the result covered as many as useful keywords.

2. Filtration parameters in step 5 – the thresholds of scores and ratios. The threshold of score for each article is set to be ten, meaning the times of appearance of the keywords from both climate change and health in one

article should be no less than 10. The threshold of ratio for each article is set to be no less than 1%, meaning in every 100 characters in the article, there should be no less than 1 keyword.

If the two thresholds are set too low, it would increase the workload of manual screening and increase the “false rate” of machine filtration. And if the two thresholds are set too high, it would possibly exclude the “true” articles. So after several trial tests, the thresholds for score and the ratio is better set as no less than 10 and 1% respectively. Under these settings, all 74 articles that are considered “true” in the past 2008-2018 analysis can be “found” by the machine filtration; and the workload of manual screening is also acceptable (less than 100 articles for each year).

Table 24: Chinese keywords for the search in People’s Daily.

原始气候变化关键词	新气候变化关键词	气候变化二级关键词	健康关键词	新健康关键词	剔除词
**气候变化**	气候变化	霾	疟疾	疟疾	口蹄疫
**全球变暖**	全球变暖	空气污染	腹泻	腹泻	黑烂病
**温室**	温室	大气污染	感染	感染	珊瑚死亡
**极端天气**	极端天气		肺炎	肺炎	沙虫死亡
**全球环境变化**	全球环境变化		流行病	流行病	高温加热
**低碳**	低碳		公共卫生	公共卫生	低碳水
**可再生能源**	可再生能源		卫生	卫生	健康发展
**碳排放**	碳排放		发病	发病	生态健康
**二氧化碳排放**	二氧化碳排放		营养	营养	河流健康
**气候污染**	气候污染		精神障碍	精神障碍	生态环境健康
**气候**	气候			发育	
**全球升温**	全球升温			传染	
**再生能源**	再生能源			疾患	
**CO2排放**	CO2排放			症	
**污染**	污染			瘟疫	
	极端气候			流感	
	高温			流行感冒	
	变暖			治疗	
	排放			保健	
	环境变化			健康	
	升温			死亡	
	全球温升			精神疾病	
	热浪			精神病	
	暴雨			登革热	
	气温			饥饿	
	洪水			粮食	

	洪灾			有害	
	气候反常			皮肤病	
	野火			风湿	
	山火			呼吸系统疾病	
	雪灾			人类健康	
	低温			人体健康	
	年代际			身体健康	
	冰雪			心脏病	
	可持续发展			糖尿病	
	海洋酸化			疾病	
	静稳			热死	
	温室气体			口罩	
				防护	

Table 25: English translation of the Chinese keywords.

Original Keywords of "Climate Change"	New keywords of "Climate Change"	Sub- level keywords of "Climate Change"	Keywords of "Health"	New keywords of "Health"	Removal words
Climate change	Climate change	Haze	Malaria	Malaria	Aftosa
**Global warming**	Global warming	Air pollution	Diarrhea	Diarrhea	Black shank
**Greenhouse**	Greenhouse	Atmospheric Pollution	Infected	Infected	Coral death
**Extreme weather**	Extreme weather		Pneumonia	Pneumonia	Sandworm death
**Global environment change**	Global environment change		Epidemic	Epidemic	Heating to higher temperature
**Low carbon**	Low carbon		Public health	Public health	Low carbohydrate
**Carbon dioxide emissions**	Carbon dioxide emissions		Hygiene	Hygiene	Healthy development
**Renewable energy**	Renewable energy		Disease outbreak	Disease outbreak	Ecological health
**Carbon Production**	Carbon Production		Nutrition	Nutrition	River health
**Air pollution**	Air pollution		Mental disorders	Mental disorders	Eco-environmental health
**Climate**	Climate			Growth	
**Global warming**	Global warming			Infection	
**Renewable energy**	Renewable energy			Affection	
**CO_2 emissions**	CO_2 emissions			Symptom	
**Pollution**	Pollution			Epidemic	
	Extreme weather			Flu	
	High temperature			Influenza	
	Warming			Treatment	
	Emission			Health care	

	Environmental change			Health	
	Warming			Death	
	Global warming			Mental disease	
	Heat wave			Mental illness	
	Rainstorm			Dengue	
	Temperature			Hunger	
	Flood			Food	
	Flood			Harmful	
	Abnormal weather			Skin disease	
	Wildfire			Rheumatism	
	Mountain fire			Respiratory diseases	
	Snowstorm			Human health	
	Low temperature			Body health	
	Interdecadal			Heart disease	
	Ice and snow			Diabetes	
	Sustainable development			Illnesses	
	Ocean acidification			Heat death	
	Stagnant			Mask	
	Greenhouse gas			Protection	
				Survive	

Data

1. All the articles from 2008 to the present published on People's Daily (from the official website of People's Daily).

Additional analysis

Table 26: Titles of the articles covering health and climate change in the People's Daily in 2019.

**文章名字**	**Title of the article**
极端天气肆虐欧洲多国	Extreme weather in Europe
全球气候治理，需要行动的合力	Global climate governance requires need cooperation work
3个州进入紧急状态 受影响人口超过1.4亿 极寒天气肆虐美国中西部地区	The emergency has been declared in three states because the extremely cold climate, which has affected 140 billion people in American Midwest area
非洲国家积极寻求粮食安全出路	African countries seek for food security actively
强热带气旋“伊代”带来暴风、强降雨天气和洪涝灾害，受灾人数超过300万中国救援队在莫桑比克展开医疗救助行动	Chinese rescue team carried out medical assistance operations in Mozambique because more than 3 million people have been affected by the strong tropical cyclone 'Idai', which brought storms, heavy rain and floods.
世界气象组织报告显示全球变暖加剧	WMO says the global warming has intensified
非洲多国粮食安全问题依然严峻	There are still many problems about food security in Africa countries
今年南方入汛提前，北方局部地区有可能发生夏旱防汛与抗旱，都不可大意	This year South China might enter flood season earlier, and part area in North China will have drought. The government should take actions towards flood and drought seriously
中国正与世界分享农业实践成功经验	China is sharing her successful experience in agriculture sector with the world
6月份最后一周有7个国家最高温突破45摄氏度欧洲大陆遭遇罕见热浪袭击	For the last week in June, 7 countries' temperature has broken 45°C. the European continent is suffering from a rare heatwave

印度遭受连续高温炙烤	The continuous high temperature in India
特大洪灾肆虐南亚三国	The super flood in three countries in South Asia
东南亚全力应对登革热疫情	Southeast Asia sparing no effort to address the dengue fever
联合国报告显示，2018年世界饥饿人口达8.216亿全球实现“零饥饿”目标面临挑战	The United Nations reported that 8.216 billion people are in hunger in 2018. It is a challenge for the global goals
中国为解决全球发展不平等带来曙光	China brings hope for solving the unbalanced development problem in the world
今年汛期全国平均气温偏高十月西南等地降水依然较多	The average temperature this year is higher during the flood season, and southwest area rainfall still high
中非合作助力非洲早日实现“零饥饿”	China and African government cooperate to help Africa get out from hunger
中法生物多样性保护和气候变化（2019年11月6日，北京）	The biodiversity protection and climate change between France and China (Nov 6th 2019, Beijing)
报告显示气候变化已开始损害儿童健康	A report shows that climate change is damaging children's health
法国多地空气污染治理不达标	Many area’s air pollution governance doesn’t reach the standard in France
澳大利亚山火持续肆虐	The wildfire in Australia still continues

Table 27: Titles of the articles covering health and climate change in the People’s Daily 2008-2018.

年份	文章名字	Title
2008	天气预报与气候预测怎样更准确　北京奥运气象服务怎样更精细	How to make weather forecast and climate forecast more accurate(the climate service during Beijing Olympic games how to be more careful)
	全球变暖也会有寒冬	The global warming still has a cold winter although
	极端天气的警示	The warning from the extreme weather
	瑞典　大雪灾有教训　认真对待　不可轻敌	A lesson from the huge snow disaster in Sweden ---treat with it seriously,don’t look down it
	我国正在经历一场历史罕见低温雨雪冰冻灾害	Our country is suffering a low temperature sleety frozen disaster which is infrequent in our history
	印尼想方设法抗暴雨	Indonesia is casting about resisting the heavy rain
	一些国家继续对我国部分地区遭冰雪灾害表示慰问　联合国环境署与人居署赞赏中国	Some countries are still consolatory to our part areas which suffered from the snow disaster.United nations environment programme and United Nations Habitat praise China
	万众一心融冰　众志成城化雪——四川抗击冰雪灾害纪实	All the people in one mind is melting ice,and the unity in strength is melting snow.-----The record about people resist snow disaster in Szechwan province
	温暖融化冰雪——甘肃抗击低温冰雪灾害纪实	The warm can melt the snow---The record about people resist low temperature snow disaster in Gansu province
	18万白衣天使奋战在抗击雨雪冰冻灾害的前沿　冻不住的生命线	One hundred and eighteen thousand ‘white angels’ (doctors and nurses) are fighting in front of the frozen disaster’s front line,which can’t freeze the lives
	在提高粮食产量上做文章——关于世界粮食价格上涨的对话（上）	An article about how to advance the food’s output---a conversation about the world food’s advancing price
	美国地球政策研究所所长建议　用非常手段应对全球粮食危机　希拉克呼吁重视粮食	The director of the American Earth policy institute suggests that :we should use a extremity approach to answer the global food crisis,and Chirac government appeals to think much of food

	广西西林　山洪灾害造成10人死亡　汛期提前，洪涝及地质灾害随时可能发生　自	The Flash flood due to 10 people's death in Guangxi Xilin,the flood season comes earlier which makes it is possible to happen flood and geological disaster
	联合国粮农组织总干事呼吁　各国采取紧急措施大力发展农业　亚行行长主张提高农	The united nations food and agriculture organization general director appeals that:every country should take urgent measure in developing agriculture,President of ADB protest develop agriculture
	国家防总紧急启动防汛Ⅲ级应急响应　防御南方暴雨洪灾	Nation defense start flood control level Ⅲ emergency response urgently to recover the rainstorm and flood in south
	珠江将发生流域性洪水广西部分江河发生特大洪水	Peal river area will happen a catchment flood and Guangxi part will have a huge flood
	今年全国洪涝受灾4300万人	In our country 4.3 billion people went through the flood this year
	外交部举行吹风会　介绍胡锦涛主席即将出席八国集团同有关国家领导人对话会议情况	The ministry of foreign affairs hold a meeting which is called'leaking meeting' to introduce the meeting situation ,which chairman Hu Jintao will attend the group of eight and communicate with concerned countries' leaders
	湖北恩施　暴雨已造成八人死亡两人失踪	In Enshi -Hubei the rainstorm has caused 8 people dead and 2 people disappeared
	今日"小暑"　我国大部地区进入炎热季节　沪赣皖等发布高温橙色预警信号	Today is traditional 'slight heat',and most areas have in a hot season, Shanghai Jiangxi Anhui and other province put out the high temperature orange warn signal
	国家气候中心解析上半年我国天气气候特点　气象灾害重　极端事件多	National climate center analysis the characteristics about weather and climate in our country,the result is that climate disaster is serious and the extreme events are more than before
	经济大国能源安全和气候变化领导人会议在日本举行　胡锦涛出席并发表重要讲话	Chairman Hu Jintao attend the meeting hold in Japan which is called 'Economic power country's leader about energy security and climate change and give an important speech
	适应气候变化是现实的选择——专访中国气象局气候变化中心主任罗勇	Accommodate the climate change is a realist choice--interview the china meteorological administration climate change center's director Luo Yong
	滁河发生历史第二位大洪水　回良玉要求确保防汛安全	The Chun River happened the second serious flood in history,Hui Liangyu require to ensure the flood control safety
	为了美丽的家园	To our beautiful homeland
	潘基文呼吁应对粮食安全面临的挑战	Pan Jiwen appeals to meet the food safety challenge
	中国应对气候变化的政策与行动	China's policy and action tackles climate changing
	十九县遭受强降雨雪袭击　西藏全力以赴抗击雪灾	Nineteen country seat suffered in the rainstorm attraction, Tibet will go all out to fight with snow disaster
	云南发生特大滑坡泥石流灾害　22人死亡　42人失踪	A severe landslide disaster in Yunan caused 22 death and 42 missing
2009	流行病蔓延与全球变暖（大千絮语）	The epidemic spread and global warming

	不能指望"天帮忙"只能依靠"人努力"	Rely on "working" instead of "praying"
	改善生态环境保障粮食安全	Improve the zoology environment to protect the food safety
	以人为本保护大气	Based on the human, protect the atmosphere
	积极应对全球气候变暖	Confront the global warming problem actively
	同舟共济关爱地球（人民论坛）	People in the same condition help each other to care about the earth
	清洁空气绿色发展（科技大观）	The clean air about the green development (technology opinion)
	携手保障世界粮食安全（国际论坛）	Hand to hand to protect the world food safety(International forum)
	研究报告预示减排政策转变？	The research forecasts that the emission reducing policy will change
	中国正着手制定长期应对气候变化国家方案	China is preparing the country plan about the climate change
	人畜共患病缘何频发	Why Zoonosis is frequently spreading?
	热浪连袭，各地拉响警报（热点解读）	The heat wave occurs continuously, each place sounds alarm（hot spot interpretation)
	印度酷暑难当	The extreme heat in India
	非洲不想让农业拖发展后腿	The Africa avoids the agriculture hinder the development
	地球"发烧"气候异常（热点解读对话）	The earth has a fever which cause the climate unusual(hot spot interpretation dialog)
	高温立法如何趋利避害（关注高温立法）	High temperature legislation how to profit -seeking to avoid harm
	强降雨使重庆严重受灾	The rainstorm makes Chongqing have suffered damage seriously
	太平洋岛国正遭受气候变化严重威胁	The Pacific island countries are threatened suffering the climate change seriously

	非洲要求公平应对气候变化（国际论坛）	The Africa requires to confront the climate changing fairly
	警惕甲感"第二波"（热点解读）	Pay attention to a sense comes again(hot spot interpretation)
	水灾殃及西非多国	The flood brings disaster to the west Africa countries
	土耳其洪水造成多人死亡	The flood in turkey effect many people dead
	"流泪"的冰川（环球走笔）	The glaciers are crying(Globe Walking pen)
	中国将发挥更加积极建设性作用	China will make a more positive effect in contribution
	联合国气候变化峰会在纽约举行	The United Nations climate change summit will hold in New York
	目标·基础·关键	Target,basic and point
	携手应对气候变化挑战	Confront the climate challenge hand by hand
	南非城市闲地种庄稼	The free city's idly in South Africa is used to seed crops
	十亿饥饿人口的警示	The warning of ten billion people in hunger
	让人人享有生殖健康	Let everybody enjoy the reproductive health
	防灾预案不能"没想到"（人民时评）	The disaster prevention plan should everything be ready(people's review)
	全球目光投向哥本哈根（国际视点）	The global eyes turns to Copenhagen (internationals point)
	增加投入协同应对（经济透视）	Increase investment and together confront(economic perspective)
	假如极端天气突袭哥本哈根（漫笔）	What if the extreme weather attack Copenhagen harden(informal essay)
2010	启动国家四级救灾应急响应	Initiate national level 4 disaster relief emergency response
	新疆——直升机救援雪崩受困牧民	Sinking—use helicopter to rescue the herdsmen who were snowbound

	天气走极端，防灾保安全（民生聚焦）	The extreme weather needs disaster prevention and safety (people's livelihood focus)
	8000顶救灾帐篷急运新疆灾区	8 thousands disaster relief tents are transported to Sinkiang's disaster area
	服务经济社会发展保障人民安全福祉	Serving economic and social development ensure people's security
	暴雨大风袭击我国南方部分省份	Part of provinces are attacked by the rainstorm and strong wind in south
	强降雨致部分地区受灾	The rainstorm induced some area suffering damage
	大旱之后遇大涝地质灾害要当心（热点解读）	We should be careful when we meet the geological disaster after the drought(hot spot interpretation)
	进村义诊紧急消毒	Doctors are volunteering to do emergent disinfection in countryside,
	直升机救起洪水围困群众	The helicopter rescue the people who were surrounded by the flood
	国家紧急启动三级救灾应急响应	Initiate national level 3 disaster relief emergency response
	南方9省份约1533万人受灾	There are about 1.533 billion people are stricken in 9 provinces in South
	"危难时刻，他们的名字叫'共产党员'"	Commie appears in the crisis
	全国自然灾害直接经济损失2113.9亿元	The country directly had an economic losts about $ 211.39 billions due to the natural disaster
	渝鄂湘赣等地灾情严重	The situation of a disaster in Chongqing Hubei Hunan Jiangxi and other places is very serious
	高温关怀离不开法制保障（人民时评）	High temperature care can't leave the legal protection(people's review)
	强降雨致10省市1563万人受灾(热点解读·对话)	The rainstorm brings 1.563 million people on suffering disaster
	长江上游形成大洪水（热点解读）	There is a big deluge upper the Yangtze River(hot spot interpretation)

	量力而行有序参观	Do according to your abilities and visit orderly
	汛情仍严峻抗洪不能松（热点解读）	The flood situation is still severe,we can’t take a breath during fighting
	北方高温将持续到月末	The high temperature in north will continue until the end of the month
	长江汉江洪水总体可控(热点解读)	The flood in the Yangtze River and the Han river generally can control(hot spot interpretation)
	洪水肆虐巴基斯坦	The Pakistan was ravaged by the flood
	上海将持续晴热高温——世博园多措并举迎”烤”验	Shanghai will continue the high temperature and sunny days—-the expo park will meet a test
	高温停工莫成一纸空文(民生观)	We are not allowed to make the high temperature shutdown no sense(livelihood)
	中国第二批人道主义救灾物资运抵巴基斯坦	The second batch of humanitarian relief materials from China is arrived in Pakistan
	以高度负责态度应对全球气候变化努力实现”十一五”节能减排目标	Highly responsible to confront the global climate change,and are try our best to realize the “ten one five” target of energy conversation
	中国将继续向巴基斯坦提供支持和帮助	China will still provide support and help to Pakistan
	海南遭遇特大暴雨（热点解读）	Hainan suffered a super rainstorm(hot spot interpretation)
	尾气成城市空气主要污染源（经济聚焦）	The exhaust gas has become the major source of pollution(economic focus)
	健康亚运健康广州	Healthy Asian games and healthy Guangzhou
	“十一五”能耗降20%可望实现（热点解读）	The 11th Five-year can realize the reduce consumption about 20 percent (hot spot interpretation ）
	联合国气候变化坎昆会议（第一现场）	The united nations conference on climate change Cankun(first sense)
	量化减排成坎昆难解之题	Quantitative emission reduction in Cankun is a difficult problem

	寒潮暴雪为何频频来袭？(热点解读)	Why the cold-air outbreak and blizzard come frequently?(hot spot interpretation)
2011	洪水过后，望谟高考正常举行（热点解读）	After the flood,the college entrance examination in Wangmo will hold normally(hot spot interpretation)
	南方雨势加强范围扩大	The heavy rain in south will be extended
	20多条中小河流水位超警（热点解读）	The water level of more than 20 rivers are over warning (hot spot interpretation)
	南方多省遭遇暴雨洪涝灾情	Many provinces in south have suffered the rainstorm and flood disasters
	应对热带风暴"米雷"	Confront the tropical storm "mile"
	连续十一天发布高温预警	Issue high temperature warning for 11 consecutive days
	领个高温补贴有多难(政策解读)	How difficult to receive high temperature subsidy!(policy analyzing)
	川陕豫1229万人受灾（热点解读）	In Sichuan Shaanxi and Henan there are 12.29 million people are in disaster(hot spot interpretation)
	曼谷严阵以待防洪魔	Bangkok will embattled to the flood
	泰国洪灾开始缓解	The flood in Thailand starts remission
	我国拟将PM2.5纳入常规空气质量评价	Our country prepare to put PM2.5 into the air quality assessment
	增强适应气候变化能力保障可持续发展	Strengthen the ability about accommodating the climate change to ensure the sustainable development
	中国应对气候变化的政策与行动（2011）	China's policies and actions about confronting the climate change (2011)
	贫穷的人们在等待德班的决定	The poor people are waiting for Durban's decision
	城市雾霾从哪里来	Where is the urban smog from?
2012	非洲萨赫勒地区遭遇严重粮荒	The Sahel in Africa suffered a serious food shortage

	大风为何袭扰江浙沪	Why the wind attacked Jiangsu Zhejiang and Shanghai ?
	适应气候变化提高防灾减灾能力	Accommodate the climate changes and advance the ability in disaster prevention and mitigation capabilities
	暴雨连袭南方迎战（热点解读）	The rainstorm attack suddenly and in south people are prepare for the fight(hot spot interpretation)
	砸不垮的脊梁	The back can't be destroyed
	粮食安全关乎非洲发展前景	The food security decides the prospects in Africa
	国家防总启动防汛应急响应	The national defence starts flood control emergency response
	暴雨将袭华北和东北等地	The rainstorm will attack north-east and north area in China
	陕西境内黄河现二十三年最大洪峰	The yellow river in Shaanxi province has appeared the largest flood peak in 23 years
	防汛进入紧要关头	The flood control has turn into an emergency moment
	"我们一定能战胜洪灾"	We certainly can defeat the flood disaster
	"火炉"城市越来越多（关注·炎热天气）	There are more and more cities called 'stove'(focus on hot weather)
	患难之中的真情	True love in trouble
	香港治理空气污染目标未能实现	The target of air pollution abatement failed in Hongkong
	"1＞4"防灾投资能让救灾省钱	'One is better than four' ,which the disaster prevention investment can save money
	直接经济损失超5亿元	The economic losses straightly exceeded more than $500 million
	柴油车是PM2.5的排放大户（读数·发现经济运行的轨迹）	The PM2.5 exhaust emission mostly is from diesel vehicles(count•finding track about economic operation)
	创新管理，让减排做到更优（点睛）	Innovation management lets the emission reduction better(finishing touch)

2013	南方冬季湿冷易致多种疾病	Wet and cold in winter in south easily cause many disease
	中国输非洲抗疟药不是假药（求证·探寻喧哗背后的真相·"抗疟假药"调查（上））	The antimalarial which China transported to Africa is not fake(check•find out the truth behind the crowd•investment of fake antimalarial)
	雾霾来袭，如何突围？（深阅读·当日新闻的背后）	How to break out when the smog comes(deep reading• behind the news of the day)
	我们的空气怎么了（深阅读·当日新闻的背后）	What happen to the air(deep reading•behind the news of the day)
	雾霾继续最高级别预警(热点解读)	Smog is still the highest level warning(hot spot interpretation)
	雾霾天，口罩怎么选？(服务台)	How to choose the respirator when it is smoggy weather?(informational desk)
	温室气体浓度创12年来新高	The gas concentration, which in greenhouse, is the highest during recent 12 years
	再现蓝天不能只靠"应急"（热点解读）	Reappear the blue sky doesn't depend on the emergency response (hot spot interpretation)
	大气严重污染时叫停部分机动车	Part of cars are called themselves quits when the air pollution serious
	环保部回应雾霾防治（热点解读·对话）	The response about prevention and cure from the Ministry of environmental protection(hot spot interpretation •dialog)
	成霾预警分级指标（热点解读）	Smog warning classification index(hot spot interpretation)
	治理雾霾，需要告别"口头环保"（人民时评）	In order to Control smog,we need leave the verbal environmental protection(people's review)
	上月中东部雾霾频袭历史少见	Last month the smog attacked Middle East frequently,which is rare in history
	六大重污染行业排放设限	The emissions are limited in six industries,which are the major pollution
	雾霾发难，油品难逃其责(深阅读)	The oil things are responsible for the smoggy weather's coming(deep reading)

	中东部地区再迎雾霾天	The Middle East will have smoggy weather soon
	共同努力防治雾霾（代表委员问部长）	Try our best to protect the smog together(representative asked minister)
	护佑民生冷暖共促和谐发展	Ensure the change of temperature about livelihood to promote harmonious development
	中国农业创新助力发展（国际论坛）	China's agricultural innovation help the development (international forum)
	阿根廷遭遇特大洪水（第一现场）	The huge flood in Argentina (first scene)
	将健康影响纳入环境影响评价（专家视角）	The healthy is concluded in the environmental impact assessment(expert perspective)
	H7N9病毒"北上"证据尚不足（热点解读）	The evidence about H7N9 has spread to north is not enough(hot spot interpretation)
	北京出现高温雾霾天气	High temperature and smog weather in Beijing
	"天生丽质"也不能盲目乐观（绿色焦点）	We still can't be blind optimism although we have the natural beauty(green perspective)
	今夏最大范围高温来临	The high temperature will come,which is a maximal range this summer
	"温比亚"登陆，粤琼桂全力防御	Wibiya landed,so Guangdong Hainan and Guangxi vehemently defense it
	柴油车新标能否有利好空气？（绿色焦点·关注空气质量①）	Whether the new standard of diesel vehicle good for the air?(green perspective •attention to the air quality I)
	今年最强降雨袭北方	The strongest rainstorm will attack north this year
	强降雨致多地受灾	The rainstorm makes many places suffer disaster
	炎炎夏季好睡眠（小贴士）	Sleep well in hot summer(tips)
	高温难退，"烤"验持续(热点解读)	High temperature is hard to retreat,and the trial is continued (hot spot interpretation)

	8月份部分地区可能更热（热点解读）	In some areas weather will be hotter in July (hot spot interpretation)
	面对高温，如何应急（政策聚焦）	How to meet an emergency when we confront the high temperature (policy focus)
	防暑降温措施落地了吗(政策聚焦)	Has it put out the measures of heat and cold control?(policy focus)
	如何预防热射病（民生服务窗）	How to prevent the fever?(livelihood service window)
	近年降水为何"北多南少"（绿色焦点）	Why the amount of precipitation in north is more than south in recent years(green focus)
	如何预防中暑（链接）	How to prevent heatstroke (interlinkage)
	专家提醒高温作业应事先体检（信息服务台）	Expert remind that people need a prior medical examination before high temperature operation(information desk)
	做好高温天气医疗卫生服务	We need a good health service when the hot weather
	高温天气如何防疾病（民生服务窗）	How to prevent the illness in hot weather?(livelihood service window)
	多地汛情严峻	The flood is serious in many places
	今年气候尚属正常	The weather in this year is temporary normal
	洪水盘踞东北高温渐离江南	The flood is entrenched in northeast,and the high temperature gradually leave regions south of the Yangtze River
	农业创造财富和就业机会（国际论坛）	Agriculture creates the opportunities of wealth and employment(international forum)
	汕头普宁内涝严重20万群众转移安置	The 200 thousand people are relocated because the serious water logging in Shantou and Puning
	松花江流域洪水将持续（热点解读）	The flood will continue in Songhua river(Hot spot interpretation)
	当地回应称，不存在任何隐瞒（热点解读）	The local government replied that there are no concealment(hot spot interpretation)

	陕西"杀人蜂"为何肆虐	Why the bees in Shaanxi ,which can kill people, are such wild
	澳大利亚热议环境治理困境（国际视点）	Australian government ardently discusses the environmental governance dilemma (international viewpoint)
	雾霾对生殖能力影响不大	Smog has little effect in fertility
	挥发性有机物污染，危害不小（绿色焦点·关注大气污染防治②）	Volatilization of organic pollution's harmful isn't small(green focus•care about the air pollution control)
2014	空气质量预报如何更准确(绿色家园·关注大气污染防治③)	How to make the air quality forecast more accurate?(green home•care about the air pollution control)
	治雾霾，谁和谁在博弈？（民生新起点）	In order to control smog who we are fighting to ?(A new starting point for people's livelihood)
	接种一次疫苗不能终身免疫（服务窗）	Vaccination can't be lifetime immunity (service window)
	全面落实国家适应气候变化战略	Fully implement the national strategy which confront the climate change
	多地遭暴雨袭击	Many places suffered the rainstorm
	13日南方将迎新一轮强降雨	The south will greet a new round of heavy rain in May 13th
	抗灾答案，书写始于暴雨之前（评论员观察）	The answer of the flood control is written before the rainstorm's beginning(commentator observation)
	气候在变化风险有多大(绿色家园)	The risk is growing because of the climate changing(green home)
	大气污染防治法执法检查启动	The law enforcement inspection starts,which is about the air pollution control
	保持凉爽防中暑	Keep cool to prevent from heat stoke
	最近天空比较蓝	The skies are blue in recent days
	最近天空比较蓝	The skies are blue in recent days
	冰川在哭泣	The glacier is crying

	遏制全球变暖行动刻不容缓	The action about preventing global warming can’t wait
	防控登革热广州大灭蚊(热点解读)	Guangzhou control mosquitoes to control the dengue(hot spot interpretation)
	气候灾变问题很遥远吗（生态论苑）	Is the climate catastrophe problem far?(ecological forum)
	科学认知气候变化高度重视气候安全	Cognize the climate change scientifically,and highly valued climate security
	极端气象灾害威胁国家安全（绿色家园）	The extreme weather disasters threaten national security (green home)
2015	去年全国灾情总体偏轻	The disaster last year was lighter
	去年大气污染扩散气象条件较差	The atmosphere pollution and diffusion weather conditions were worse last year
	科学认知气候关注气候安全	Cognize climate scientifically and care about climate security
	源头精细管控按尾气排放限行（他山之石）	Source carefully control limit line,which according to the exhaust emission (the Stone of Other Mountains)
	今夏要防"南涝北旱"（深阅读）	We need prevent the flood in south and the drought in north(deep•reading）
	南方强降雨造成48人死亡失踪	The rainstorm in south conduced that 48 people died and disappeared
	今年不会出现"史上最强"厄尔尼诺	‘This year will not appear the strongest El Nino in history
	"防"字当头应对极端天气	Confront the extreme weather we need protection first
	维护气候安全保障生态文明	Maintain the climate security to ensure the ecological civilization
	厄尔尼诺袭来极端天气增多（绿色焦点·气象防灾减灾④）	El Nino’s attacking lead to the increase of extreme weather (green focus•weather control)
	该河段水中未检出氰化物	No cyanide detected d in the river water
	今年洪灾死亡人数历史同期最少	The number of the dead people ,which is because the flood,is the least in history

	生态环境科技发展新趋势（审时度势）	Eco-environmental technology develops a new tend((consider the situation)
	气候变化可能威胁社会发展和全球健康成果	The climate change may threaten social development and global health outcomes
	青藏高原气候变化：变暖变湿	Climate change on the Tibetan plateau:warm and wet
2016	中国启动对非紧急粮食援助	China launches food emergency aid to Africa
	百年最强厄尔尼诺形成（绿色家园）	The strongest El Nino in recent hundred years has formed(green home)
	强化行动以应对气候安全挑战（专家视角）	Strengthen the actions in order to confront the challenges about climate security (expert perspective)
	莫让极端天气酿成极端灾害（人民时评）	Don’t let the extreme weather cause the extreme disasters(people’s review)
	中央气象台暴雨预警升级为黄色	The rainstorm warning in Central Weather Station upgrade to yellow
	三峡腾出库容确保长江度汛	Three gorges vacated the reservoir to help the Yangtze River pass the flood season
	我国已全面进入主汛期（在国新办新闻发布会上）	Our country is in main flood season(press conference in the State Council Information Office)
	长江中下游迎来最强降雨	Middle and lower Yangtze River will have the strongest rainfall
	今年降水较常年多二成	The precipitation this year is higher than before
	长江干堤支堤险情均有效控制（关注南方汛情·动态）	The danger of the Yangtze River main embankment has been controlled(pay attention to the flood situation in south• trends)
	“绿色合力”也是防灾举措（生态论苑）	‘Green together’ is also a protection about disaster control (ecological forum)
	洪灾疾控有锦囊（信息服务台）	Tips for the flood control(information desk)
	河北洪灾已致130人死亡	The flood in Hebei has caused 130 people died

	高温天气防暑防肠炎（信息服务台）	Prevent sunstroke and anti-enteritis in hot weather(information desk)
	推进全球气候治理，让《巴黎协定》尽早生效（聚焦G20杭州峰会·成果展望）	Advance the management of global climate to become the Paris Agreement effective early (focus on G20 Hangzhou Summit•result outlook)
	全国爱粮节粮宣传周启动	The national food awareness week was lunched
	非洲空气污染呈加重态势	The air pollution in Africa is aggravating the situation
	雾霾来了，新德里喘不过气	The smog's coming makes New Delhi lose it's breath
	辽宁：将严肃处理瞒报责任人	Liaoning:we will deal with the principal seriously who concealed
2017	该给地球降温了（绿色家园）	It is time to cool the earth(green home)
	中俄探索中西医结合治寒带病	China and Russia integrated Chinese and western medical in order to explore the treatment of cold disease
	水位虽有减退形势依然严峻	The water level although letdown,but the situation is still severer
	部分地区汛情不容大意	The flood in some places should be careful
	一带一路拓展中非农业合作	The belt and road initiative expand the cooperation between China and Africa
	今夏为啥这么热（绿色焦点）	Why this summer is so hot? (green focus)
	让清洁美丽世界为文明添彩（钟声·推动构建人类命运共同体⑦）	Let the beautiful cleaning world add colour to civilization(ring•promote to contribute the community of human destiny)
2018	气候变化影响人类健康	Climate change influences human health
	去年我国冬季气温为历史同期最高	The winter temperature in our country last year was the highest,which is in the same period in history
	我们没有星球B（域外听风）	There isn't a planet B for us to live(extraterrestrial listening)

	极端天气趋多，如何有效应对？（绿色焦点）	How to confront effectively of the extreme weather’s increasing?(green focus)
	空气质量堪忧，欧盟下“最后通牒”	Worried about the air quality,European union put out the final notice
	遭遇强降雨多地齐防汛	Many places get a flood control because the rainstorm suffering
	汛情紧要时防灾松不得（美丽中国·热点）	Flood control can’t be slack at a crucial time(beauty China • hotspot)
	”七下八上”关键期防汛抗洪不松懈（全力开展防汛救灾）	“Seven down eight up” Flood control can’t be slack at a crucial time (nationwide flood control and disaster relief)
	欧洲多地遭遇极端高温天气	Many places in Europe suffered the extreme high temperature weather
	减少极端天气要靠”人努力”(生态论苑)	To reduce the extreme weather depends on people’s effort(ecological forum)
	印度多地遭遇暴雨和洪灾	Many places suffered the rainstorm and flood in India
	应对气候变暖还需持续攻坚（绿色焦点）	We need to continue to attack for the climate warming (green Focus)
	救灾乏力折射美国体制弊端（深度观察）	Weak relief reflects the disadvantages of American system(in-depth observation)

Indicator 5.1.3: Content of Coverage in US and Indian Newspapers

Methods

This indicator complements the tracking of media engagement by focusing on the *content* of media coverage of health and climate change, enabling better understanding about what is being reported as well as the levels of coverage.

Media sources and timeframe

The focus was on the elite media in two countries representing very different contexts. Two English-language newspapers from India and two from the US were examined. The media sources considered are the *Hindustan Times* (HT), *Times of India* (TOI), *Washington Post* (WP), and *New York Times* (NYT).

As in the 2019 Lancet Countdown report, the focus of analysis was narrowed for articles in two time periods during 2019. First, the time period July to September (inclusive) was considered for both the Indian and US sources. This time period is used as it covers a period of events that are linked to extreme weather in both regions; wildfires in the US and monsoon flooding in India. This enables consideration of media reporting in light of these events, and the ways in which links may be made through them to climate change and health. Second, reporting during November to December 2019 is considered. This time period covers the lead up to and hosting of the Conference of the Parties (COP) talks. In addition, this covers the time period during which findings from the Lancet Countdown report itself has been reported in the media.

Search terms

Media articles were obtained in conjunction with researchers developing indicators for section 5.1.1 (trends in media coverage). Search terms developed by this team of researchers, that were designed to return articles at the intersection of health and climate change, were used. For identification of articles in the Indian media (HT and TOI) we used the Factiva database. For identification of articles in the U.S. media (WP and NYT) we used the Nexis database.

Articles in which appeared a minimum of one key search term from both (a) health, and (b) climate change were identified:

Health terms

- **Health**
- **Illness**
- **Epidemiolog***
- **Malnutrition**
- **Morbidity**
- **Fatalit***
- **Diarrh***
- **Malaria**
- **Chikungunya**
- **West Nile**
- **Dengue**
- **Hay-fever**
- **Zika**

Climate change terms

- Climate change
- Global warming

Pre-screening of articles

The initial search string returned 1,073 articles across the four media sources. The articles across the five months and four media sources were pre-screened in order to ensure that only those making meaningful connections between health and climate change were retained for further analysis.

The procedure used to select articles was as follows:

a. An article must make a meaningful connection between health and climate change. This can be made explicitly, or implied through the narrative used, but health topics and climate change aspects must be clearly linked to be included.
b. Articles are retained where any reference is made to health and climate change that meets criterion (a). This may include long articles where only passing reference is made to the link, as well as articles where the focus is more substantial.
c. Where reference to air pollution is made, it is not deemed to meet the criterion (a) unless an explicit or implicit link is made to health. For example, an article that covers the need for coal-fired plants to close in order to meet climate change targets and reduce air pollution, is not retained unless a link is also made to the health impacts of either air pollution or climate change. It is not enough simply to reference air pollution in the context of climate change for this to be deemed reference to 'health'.

Coder 1 read all articles in order to screen for false positive articles (those returned by the search string but in which no meaningful connection was made between health and climate change, as above) and in order to remove duplicates. Following screening, 209 articles were retained for coding, corresponding to the following totals per newspaper: HT (42); TOI (39); NYT (83); WP (45).

Application of coding framework

For the 2019 *Lancet Countdown* report a coding framework designed to align with the indicators used elsewhere in the report (particularly Working Group 1 and Working Group 2) was developed. This framework was refined iteratively and applied for the 2019 report. The same coding framework was applied for the 2020 report. Having coded all articles, for the 2020 report the three 'air pollution' sub-categories, and the three 'adaptation' categories were combined for final reporting, to reflect the fact that relatively few articles were coded within these.

The final framework incorporated the following codes/themes:

1. Health impacts of climate change, specifically:
    - Generic/ non-specific health impacts
    - Heatwaves and temperature increase
    - Precipitation extremes and storms
    - Wildfires
    - Disease (vector-borne, lifestyle)
    - Food security/ malnutrition
    - Population displacement
    - Mental health and illness
    - Other impacts

2. Benefits of addressing climate change and health together (co-benefits/co-hazards and common causes), specifically:
    - Air pollution (non-specific; relating to transport or energy generation; other co-hazards of air pollution)
    - Food/ diet and agriculture
    - Other co-benefits, common causes and co-hazards

3. Adaptation (Generic or non-specific adaptation; longer-term planning; emergency responses; other reference to adaptation)

4. Other/miscellaneous
    - Activism/protest (including health sector activism on climate change)
    - Lancet Countdown reference (direct reference to the programme or report)
    - Other

Data

1. Newspaper articles in *Hindustan Times*, *Times of India*, *New York Times*, *Washington Post*. Articles analysed during time period July to September, and November to December. The data used is the full

text of media articles. This cannot be made publicly available due to copyright restrictions, however the full search strings applied, together with the databases used, are detailed above.

Caveats

The content analysis is able to provide a broad picture of how health and climate change are being reported in the target news sources and time points. The selected newspapers cannot be taken to be representative of reporting across the two countries (U.S. and India) or the WHO regions in which they are located, given that different media sources are known to have widely diverging positions on climate change. The coding framework used is intended to identify themes in reporting at the intersection of health and climate change; it is not intended to provide insights into the more general ways in which climate change and/or health are reported in news media.

The articles returned are necessarily those in which there was found to be a conjunction of a pre-selected health term and climate change term. The exact search terms used are likely to have influenced the types of articles obtained. The search strings have been amended and rationalised since the previous report, in part to reduce the extent of false positive results returned. This has the consequence that the results provided in the 2020 report of the Lancet Countdown are not directly comparable to the 2019 report, although broad patterns in terms of prevalence of themes are nonetheless found to be similar.

Future form of the indicator

Analyses of the content of coverage will form part of the working group's future programme of work. The potential exists to track patterns of reporting over time in relation to content of coverage, depending on resources available. It is anticipated that media reporting of the coronavirus is likely to affect the content analysis of the 2021 report of the Lancet Countdown; for example, in consideration of the consequences for emissions of social and economic restrictions linked to attempts to contain and delay spread of the virus. This will require detailed consideration in the analysis carried out for the 2021 report.

Additional analysis

Illustrative Extracts from the Data

The following extracts from articles give an impression of the themes identified through analysis; they are sub-headed by theme.

Health impacts of climate change

"The average number of premature heat-related deaths in Britain, now about 2,000 a year, is expected to triple to more than 7,000 by the 2050s unless action is taken, the Committee on Climate Change, an independent advisory group, has said."
[*As Extreme Heat Broils Europe, Officials Search for Responses*. Elian Peltier, *New York Times*, 27 July.]

"Climate change won't just change how we live, it will radically remake how we die. Along with cancer and heart disease, now we can expect to perish during crop failures, extreme heat events, catastrophic storms and tropical disease pandemics, and suffer increases in asthma and other lung ailments."
[*A searing look at the end of life - and Earth.* Philip Kennicott, *Washington Post*, 29 September.]

"…few countries are likely to suffer from the health effects of climate change as much as India."
[*Kids hit worst by climate change: Lancet*. Sushmi Dey, *Times of India*, 15 November 2019.]

"Hot weather can cause a spike in the number of babies being born early, a phenomenon that may harm infant health and is likely to get worse as temperatures climb due to climate change, scientists said."
[*Hot weather linked to rise in early childbirth*. Agence France-Presse, *Hindustan Times*, 3 December.]

Co-benefits, co-hazards and common causes

“Access to affordable and reliable energy is fundamental to reducing poverty [and] improving health… More energy to improve lives, but with fewer emissions to help address climate change - is what we call the dual challenge.”
[*A road map to transforming India’s energy.* Dev Sanyal, *Hindustan Times*, 29 August.]

“Videos, quizzes and interactive exercises help you understand how climate change affects health, how humans might adapt to physical changes on Earth, and how you can take care of your own health while helping to sustain the planet.”
[*A free online course reveals the scary reality of global warming and the human health dangers.* Erin Blakemore, *Washington Post*, September 3.]

“Vegan burgers… are a response to rising consumer concern about the healthiness of red meat and to criticism that cattle farming is bad for the climate.”
[*Can a Company Be Virtuous and Profitable?* Jack Ewing, *New York Times*, November 17.]

“Cycling is also good for planet earth because it is a zero-emission mode of transport. Cycling also helps to fight climate change, as the carbon footprint during cycling is almost nil…”
[*Central University Punjab faculty member on solo cycling tour in Odisha*, Neel Kamal, *Times of India*, 28 December]

Adaptation

“With summer temperatures sizzling, officials in Montgomery County are considering what would be a first-in-the-region law mandating air conditioning in all rental properties… The state Department of Health received more than 450 complaints of heat-related illnesses from July 2 to July 8… "Our policy needs to catch up with the reality of climate change," [council member] Hucker said.”
[*Bill seeks to require AC in Montgomery rentals.* Rebecca Tan, *Washington Post*, 17 July.]

“Encouraging hikers to avoid the worst heat is part of a "Take a Hike. Do it Right" campaign that began in 2015 to reduce, among other incidents, heat-related rescues and deaths on the city's 200 miles of hiking trails… Last year, heat caused or contributed to the deaths of 182 people in Maricopa County, which includes Phoenix. Preliminary figures suggest the toll this year will be similar, if not higher, according to the health department… The increase is due to global climate change and to the urban heat island effect.”
[*As Phoenix Heats Up, The Night Comes Alive*. Marguerite Holloway, *New York Times*, 19 August.]

“One of India's nationally determined contributions (NDCs) under the Paris Agreement is "to better adapt to climate change by enhancing investments in development programmes in sectors vulnerable to climate change, particularly agriculture, water resources, Himalayan region, coastal regions, health, and disaster management".
[*Experts say India's climate adaptation fund inadequate*. Jayashree Nandi, *Hindustan Times*, 23 September.]

““It is our goal to take this model to the public to train them to adapt to climate change issues as preparedness programme.” Exposure to environmental pollutants for a long period will result in life threatening diseases, which increases health and economic burden to human society.”
[*Bharathidasan Univ gets funding for research project in humanities*. Sampath Kumar, *Times of India*, 21 December.]

Other/miscellaneous (including activism)

““Our first strike witnessed a crowd of some 50-odd people and this is the third such gathering. The rising number of people shows us their willingness to talk about climate change”, says Nimisha Agarwal from the NGO Jhatkaa.org… “We need to understand what air quality is and its impact on our health.””
[*City’s Eco-warriors protest for a better tomorrow*. Joyeeta Chakravorty, *Times of India*, 22 September.]

“Apart from students and scientists, organizers say, health-care workers - specifically, nurses - are anticipated to have the strongest presence at major climate rallies being held nationwide over the next few days.”

[*Nurses emerge as critical voice in activism over climate change*. Rebecca Tan, *Washington Post*, 23 September.]

“Dozens of nurses, clad in white coats and signs pinned to them that said, 'Climate change makes us sick,' also were arrested. They said they came from across the country to make a stand in the nation's capital.”
[*After another climate change protest, Jane Fonda might turn 82 in city jail.* Marissa J. Lang, *Washington Post*, December 21.]

Prevalence of themes

The figures below illustrate the relative prevalence of thematic types across newspaper articles. Thematic types were coded non-exclusively (an article could be coded multiple times) and as such percentages in Figure 57 and Figure 58 sum to over 100% for each newspaper. Figure 59 and Figure 60 show the relative prevalence of sub-categories within the two prominent themes, ‘impacts’ and ‘cobenefits/co-hazards’; here percentages sum to 100% corresponding to the proportion of codes assigned to each sub-category within the theme.

Figure 57 provides a breakdown of the proportions of newspaper articles in which principal themes were identified.

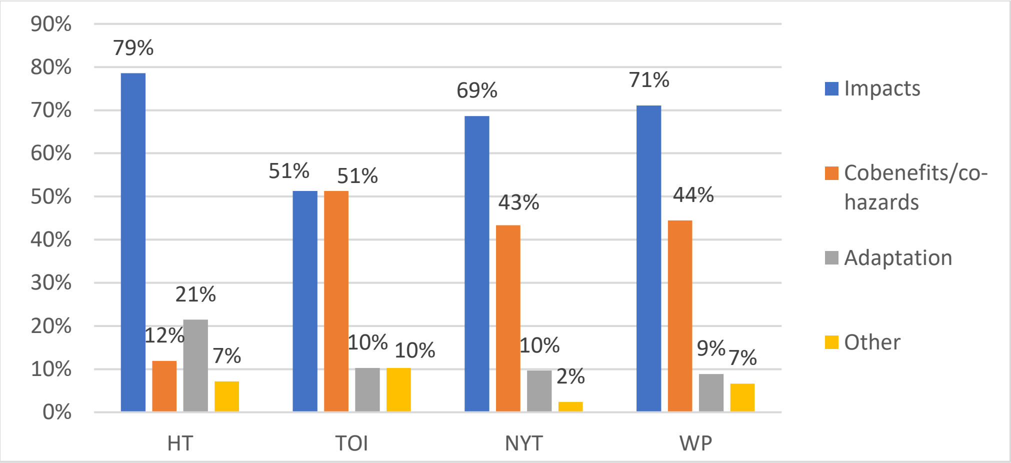

Figure 57: Proportion of newspaper articles where themes were identified, by newspaper. Note: HT = Hindustan Times, TOI = Times of India, NYT = New York Times, WP = Washington Post.

Figure 58 provides a similar breakdown of the proportions of articles in which principal themes were identified, shown by country.

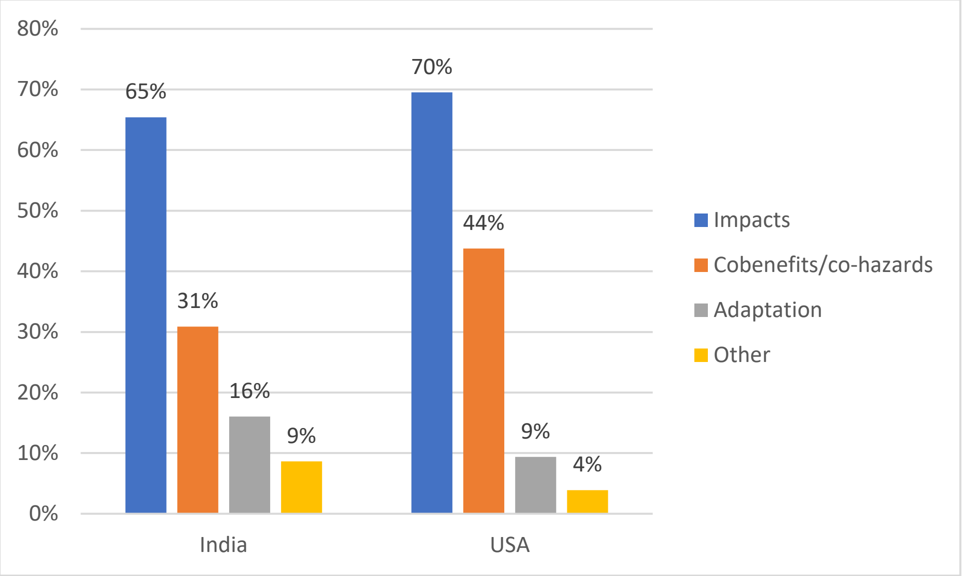

Figure 58: Proportion of newspaper articles where themes were identified, by country.

Figure 59 shows the proportion of codes identified within the impacts theme according to the sub-theme identified.

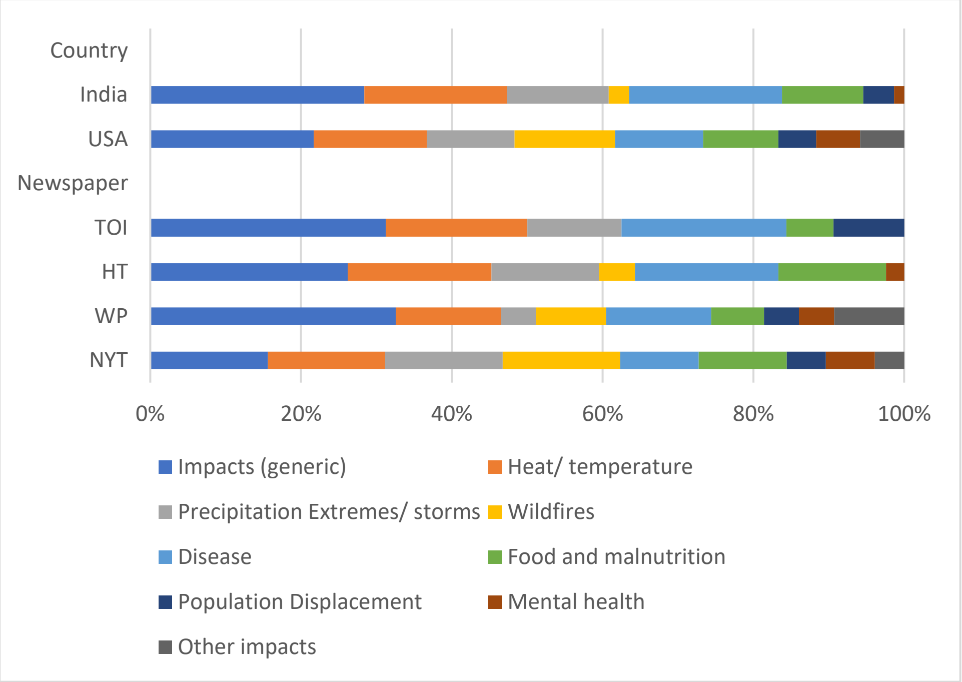

Figure 59: Proportion of sub-themes of 'Impacts' in newspaper articles. Note: HT = Hindustan Times, TOI = Times of India, NYT = New York Times, WP = Washington Post.

Figure 60 shows the proportion of codes identified within the cobenefits/co-hazards/common causes theme according to the sub-theme identified.

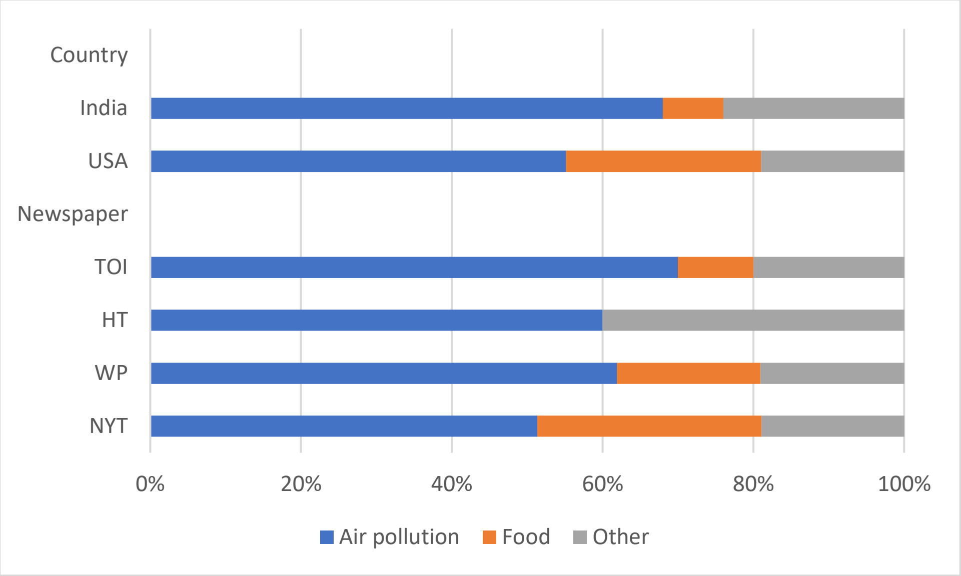

Figure 60: Proportion of sub-themes of 'cobenefits/co-hazards/common causes' in newspaper articles. Note: HT = Hindustan Times, TOI = Times of India, NYT = New York Times, WP = Washington Post.

Indicator 5.2: Individual Engagement in Health and Climate Change

Methods

This indicator provides an individual-level indicator of public engagement. It tracks engagement with climate change and health through people's usage of the online encyclopedia Wikipedia. Over the years, Wikipedia has grown to be a major and trusted source of information that has outpaced traditional encyclopedias in terms of reach, coverage, and comprehensiveness.[231] It is regularly listed among the ten most-visited websites worldwide.[232] The English edition covers more than six million articles and over 130,000 active editors. People around the world use it to engage in topics they are interested in. Fortunately, the traffic that goes to Wikipedia – and even that which goes to individual articles of the encyclopedia – can be analyzed over time because the Wikimedia foundation makes these statistics available to everyone for free. This makes it a global indicator of what people pay attention to on a daily basis. What is more – and of particular relevance in the context of this report -, the platform's health content makes it one of the most frequently used resources for information on health on the internet.[233]

To investigate to what extent people do not only pay attention to climate change and human health in isolation, but also to the connection between both, *clickstream statistics* from the English Wikipedia were drawn upon. *Clickstream* refers to a dataset provided by the Wikimedia foundation.[234] It reports "streams of clicks", or in other words: how people get to a Wikipedia article and what links they click on. This is reported on a monthly basis and in pairs of resources, the first being where the visit came from, the second which page was visited. This provides an indicator of monthly-level global attention towards one issue (if both articles are representative of the same issue) or two issues (if articles come from different domains, such as climate change and health). By looking at climate change – health articles pairs, an indicator is generated of attention towards climate change consequences for human health over time.

The approach to using clickstream data as an indicator of public engagement in climate change and health is based on the following premises: (1) The Wikipedia platform is a globally used source for information on a multitude of topics.[235] (2) Citizens use the platform to inform themselves about topics they are interested in. (3) By tracking engagement with Wikipedia articles that are related to climate change as well as with articles on health, it is possible to identify public engagement with the relationship between both topics.

The following behavioural patterns are relevant for the validity of the measure as a proxy for public engagement with climate change and health:

A person is generally interested in the nexus between climate change and public health and informs her/himself about the topic online by, e.g., reading the Wikipedia article on *Effects of global warming on human health* (https://en.wikipedia.org/wiki/Effects_of_global_warming_on_human_health).

A person is interested in climate change and the consumption of information about the topic then sparks interest in its consequences for human health. For instance, the person reads the article on *Global warming* (https://en.wikipedia.org/wiki/Global_warming) and then turns to the article on *Malnutrition* (https://en.wikipedia.org/wiki/Malnutrition).

A person is interested in a certain aspect of human health or consequences of climate change with an immediate impact on human health, and then turns its attention to climate change issues. For instance, the person reads the article on *Malaria* (https://en.wikipedia.org/wiki/Malaria) and then turns to the article on *Global warming* (https://en.wikipedia.org/wiki/Global_warming).

Indicator construction

In order to use the Wikipedia viewership statistics as a proxy for public engagement with climate change and health, it is key to select articles that are representative of these topics. To generate the populations of articles related to climate change on the one hand and health on the other, a semi-automated approach was implemented. Based on an initial set of keywords, a search was undertaken for related articles using the internal Wikipedia search.

Table 28: Initial keywords used for searching climate change and health Wikipedia articles.

Climate Change	Health
climate change	epidemy
warming	disease
ipcc	malaria
greenhouse	diarrhoea
	infection
	sars
	measles
	pneumonia
	epidemic
	pandemic
	public health
	health care
	healthcare
	epidemiology
	mortality
	morbidity
	nutrition
	illness
	infectious
	ncd
	non-communicable disease
	noncommunicable disease
	communicable disease
	air pollution
	nutrition
	malnutrition
	mental disorder
	stunting

For each search using one of the keywords, the first 100 results were extracted and those which led to an article with a minimum word count of 300 were identified, ensuring that the articles that were chosen as seed articles had been given a certain degree of attention by Wikipedia editors, therefore being more likely to link to other relevant articles.

Next, a screening was conducted of the articles collected via the Wikipedia search for categories, which are used on the Wikipedia to categorize pages in a meaningful way (e.g., using categories such as *Climate change* or *Effects of global warming*). Those categories were then themselves screened for relevant articles. All additional articles were once more filtered such that those with a title matching one of the initial keywords was chosen. For the health-related articles, it was necessary to exclude several articles manually that turned out to be irrelevant for these purposes. Health topics are covered extensively on the Wikipedia, but topics that can, in principle, be related to climate change, were priotitised. In addition, the fact that the Wikipedia page on the effects of global warming on human health offers a variety of links to further health-related articles was exploited and treated as a curated list of relevant health articles for which the links were subsequently also added to the list.[236] All in all, 551 articles related to climate change and 857 articles related to health were identified that were seen as being representative for either of the issues. The complete list of articles is listed under *Additional Information*.

For the clickstream analysis, the set of articles was extended by also taking “second-level pages” into account, that is pages that are linked to in the set of 551 climate change or 857 health articles and that are also somewhat related to climate change or health. Sometimes, people might not directly jump from one of the major articles on climate change to another one on health, but travel through an intermediary page (e.g., a possible individual stream of clicks could be: *Climate change* → *Human impact on the environment* → *Respiratory disease*). The clickstream data only permits identification of click volume for pairs of articles, but by extending the network, it is also possible to capture clickstreams involving relevant pages that are linked in the original set of articles.

After taking these additional articles into account, 1837 articles related to climate change and 6902 articles related to health were identified.

Technically, the fact that the population of health articles is far larger than the population of climate change articles does not invalidate this measurement strategy. It seems plausible that there are much more articles on health-related than on climate change-related topics because the health field is so much broader (which is one reason why the health articles cluster in the network plot is not especially dense – some health topics are very far apart from each other, although both could be covering health issues that are affected by climate change). But this should not directly affect the metrics. Even if there are many more health than climate change articles, it could still be that health topics are mentioned (and clicked on) much more often in climate change articles than the other way around. To sum up, what is key in this analysis is not that one or the other topic is more extensively covered on the platform, but the co-visit patterns. Closely related to this issue, co-visit data has not been normalized using the number of links between two topics as the baseline. An increase in the number of links could reflect increased attention in the editor population towards that topic, representing another approximation of public engagement in climate change and public health (though maybe somewhat less representative of the global population as the editor population is much smaller, different on many characteristics, and editing activities can sometimes be highly idiosyncratic and driven by highly productive individuals). If both attention and the number of possible links increases as a consequence of heightened public engagement, normalizing co-visit patterns to the number of links would obscure substantive dynamics. That being said, dynamics in the link structure may be explored more thoroughly in the future.

Data

Publicly available data from the Wikimedia foundation is drawn upon. Data from all platforms is considered, i.e. accesses to the Wikipedia via desktop machines, mobile browsers, and mobile apps.

The clickstream data were downloaded from the Wikimedia Dumps (https://dumps.wikimedia.org/other/clickstream/). Spider traffic (i.e. traffic generated by automated bots crawling the platform) is excluded. Referrer-resource pairs (i.e. the pairs of the article of origin and the target article) that had less than 10 clicks are removed in the original dataset, so slight underreporting of the actual clickstream traffic is expected. However, it is not expected that this will add any systematic bias to these indicators, in particular since interest is mainly in changes of engagement over time.

Clickstream data is available from November 2017 onwards. Data from 2018 and 2019 is the focus for this report. The analyses are limited to the English Wikipedia.

The benefits of the Wikipedia usage metadata for the purpose of tracking public engagement in climate change and health are that these data (a) are globally available, (b) cover the time period of interest, (c) are collectible at virtually no cost, and, most importantly, (e) have high face validity to measure engagement in this very specific topic. Reading articles on Wikipedia is motivated by attention towards a particular issue. Individuals invest time to inform themselves about a topic, which is one manifestation of engagement. Aggregate reading behaviour can therefore be seen as an a priori valid approximation of public issue engagement.

Caveats

All clickstream information is only available at the aggregate level. It is not possible to link the data to information about individuals who visited the platform. Also, the data are not geo-referenced, so it is not possible to infer where page visits came from. Although the English Wikipedia is predominantly used in English-speaking countries (according to the Wikimedia Traffic Analysis Report, about 40% of the traffic on the English Wikipedia comes from the United States), it is a globally popular resource.[235] It makes up for 50% of the global traffic to all Wikipedia language editions. Therefore, it can be seen as a global indicator of public attention that is somewhat biased towards attention from countries such as the United States, United Kingdom, India, Canada, and Australia. Extending the analyses to other language editions will help to remedy this bias and uncover potential geographic engagement heterogeneity in the future.

More generally, the measure represents an online proxy for an offline phenomenon. In addition, it is sensitive towards the selection of articles used to capture engagement. The global popularity of the platform, which consistently ranks among the ten most visited websites worldwide, speaks in favour of its usefulness for this application. However, more direct indicators of public engagement, such as survey-based measures, might provide a useful supplement and source for validation in the future.

While the data are available for free, access to future data depends on the Wikimedia API. There is no indication of Wikimedia restricting access in the future. Instead, Wikimedia has invested in data quality and making access more robust and convenient.

Future form of the indicator

Beyond the 2020 report of the Lancet Countdown, analyses of individual-level engagement are being undertaken using pageview data from Wikimedia. In time, it is envisaged that this indicator may draw on both clickstream and pageview data.

Various steps are planned that will help increase the precision, scope, and value of this indicator for next year's report.

First, efforts will be made to increase the number of articles used. With an ever-growing Wikipedia, more relevant articles might become available. This requires a joint automated and human classification effort to ensure that the coverage of relevant articles (true positives) is as large as possible and the number of irrelevant articles (false positives) in the sample minimal.

Second, there are plans to extend the data collection and analysis efforts to other language editions (both for the pageviews and the clickstream data). This would make it possible to track more fine-grained trends at the regional level. It is likely that there is heterogeneity in public engagement in climate change and health, as different regions of the world are currently affected by health consequences of climate change to a varying degree. Studying engagement in different language versions of the Wikipedia could at least partly pick up this heterogeneity.

Third, future work will seek to enrich the analyses with related event data. It is plausible to assume and could already be partly shown that public engagement is sensitive towards events, such as extreme weather events or epidemics, but also political and scientific activity such as the UN Climate Change Conferences or the publication of IPCC reports and protests such as the School strike for climate.

Fourth, complementary data will be explored to track and validate public attention, such as survey, experimental, and other online data.

Additional analysis

List of English Wikipedia articles used to track public engagement in climate change:

1998 United Nations Climate Change Conference, 2001 United Nations Climate Change Conference, 2002 United Nations Climate Change Conference, 2003 United Nations Climate Change Conference, 2004 United Nations Climate Change Conference, 2005 United Nations Climate Change Conference, 2006 United Nations Climate Change Conference, 2007 United Nations Climate Change Conference, 2008 United Nations Climate Change Conference, 2009 United Nations Climate Change Conference, 2010 United Nations Climate Change Conference, 2011 United Nations Climate Change Conference, 2012 United Nations Climate Change Conference, 2013 United Nations Climate Change Conference, 2014 People's Climate March, 2014 UN Climate Summit, 2014 United Nations Climate Change Conference, 2015 United Nations Climate Change Conference, 2016 United Nations Climate Change Conference, 2017 People's Climate March, 2017 United Nations Climate Change Conference, 2018 United Nations Climate Change Conference, 2019 UN Climate Action Summit, 2019 United Nations Climate Change Conference, 2020 United Nations Climate Change Conference, 4 Degrees and Beyond International Climate Conference, A Green New Deal, Abrupt climate change, Academy of Climate Change Education and Research, Action for Climate Empowerment, Adaptation to climate change in Jordan,

Adaptation to global warming in Australia, Advisory Group on Greenhouse Gases, Alice, the Zeta Cat and Climate Change, Antarctic Cold Reversal, Antarctic sea ice, Antarctica cooling controversy, APEC Climate Center, Arctic geoengineering, Arctic ice pack, Arctic methane emissions, Arctic Ocean Conference, Arctic policy of Barack Obama, Arctic resources race, Arctic sea ice decline, ArcticNet, Asilomar International Conference on Climate Intervention Technologies, Atmospheric carbon cycle, Attorney General of Virginia's climate science investigation, Attribution of recent climate change, Australian Greenhouse Office, Australian Youth Climate Coalition, Aviation and climate change, Avoiding Dangerous Climate Change (2005 conference), Bali Declaration by Climate Scientists, Bangladesh Climate Change Resilience Fund, Bangladesh Climate Change Trust, Bio-energy with carbon capture and storage, Bjerknes Centre for Climate Research, Book:Global warming, Book:Global warming denial, Breakthrough - National Centre for Climate Restoration, Business action on climate change, C40 Cities Climate Leadership Group, California Climate Action Registry, California Climate Credit, California Climate Executive Orders, Campaign against Climate Change, Canadian Youth Climate Coalition, Carbon accounting, Carbon audit regime, Carbon capture and storage in Australia, Carbon capture and utilization, Carbon Clear, Carbon credit, Carbon cycle, Carbon dioxide, Carbon dioxide equivalent, Carbon dioxide flooding, Carbon dioxide in Earth's atmosphere, Carbon Dioxide Information Analysis Center, Carbon dioxide removal, Carbon Disclosure Project, Carbon emission label, Carbon Emission Reduction Target, Carbon emission trading, Carbon farming, Carbon fee and dividend, Carbon fixation, Carbon footprint, Carbon governance in England, Carbon leakage, Carbon literacy, Carbon lock-in, Carbon monitoring, Carbon neutrality, Carbon Neutrality Coalition, Carbon offset, Carbon Pollution Reduction Scheme, Carbon pricing in Australia, Carbon pricing in Canada, Carbon process management, Carbon profiling, Carbon project, Carbon sequestration, Carbon Sequestration Leadership Forum, Carbon shifting, Carbon Solutions Global, Carbon tax, Carbon tetrachloride, Carbon tetrafluoride, Carbon Tracker, Carbon Trade Watch, Carbon War Room, Carbon-neutral fuel, CarbonFix Standard, Carboniferous rainforest collapse, CarboNZero programme, Carl Smith (climate activist), CCS and climate change mitigation, Center for Climate Systems Research, Center for the Study of Carbon Dioxide and Global Change, Centre for Climate Change Economics and Policy, Centre for International Climate and Environmental Research, Chicago Climate Action Plan, Chicago Climate Exchange, China Carbon Forum, Chlorofluorocarbon, Cities for Climate Protection program, Citizens' Climate Lobby, City of Oakland Energy and Climate Action Plan, Civil Society Coalition on Climate Change, Climate Action Network, Climate Action Plan, Climate Alliance, Climate and Clean Air Coalition to Reduce Short-Lived Climate Pollutants, Climate and energy, Climate Audit, Climate bond, Climate Capitalism, Climate Central, Climate change (general concept), Climate Change (Scotland) Act 2009, Climate Change Accountability Act (Bill C-224), Climate change acronyms, Climate Change Act 2008, Climate change adaptation, Climate change adaptation in Bangladesh, Climate change adaptation in Greenland, Climate change adaptation strategies on the German coast, Climate Change Agreement, Climate change and agriculture, Climate change and ecosystems, Climate Change and Emissions Management Amendment Act, Climate change and gender, Climate change and invasive species, Climate change and potatoes, Climate change and poverty, Climate Change and Sustainable Energy Act 2006, Climate change art, Climate Change Authority, Climate change denial, Climate Change Denial Disorder, Climate Change Denial: Heads in the Sand, Climate change denialism, Climate change education, Climate change feedback, Climate change hoax, Climate change in Alabama, Climate change in Alaska, Climate change in Arizona, Climate change in Arkansas, Climate change in Australia, Climate change in Bangladesh, Climate change in California, Climate change in Canada, Climate change in China, Climate change in Colorado, Climate change in Connecticut, Climate change in Finland, Climate change in Florida, Climate change in Georgia (U.S. state), Climate change in Grenada, Climate change in Guatemala, Climate change in Honduras, Climate change in Idaho, Climate change in India, Climate change in Indonesia, Climate change in Iowa, Climate change in Iraq, Climate change in Japan, Climate change in Kansas, Climate change in Kentucky, Climate change in Louisiana, Climate change in Maine, Climate change in Massachusetts, Climate change in Mexico, Climate change in Missouri, Climate change in Montana, Climate change in Nebraska, Climate change in Nevada, Climate change in New Jersey, Climate change in New York (state), Climate change in New York City, Climate change in New Zealand, Climate change in North Carolina, Climate change in Ohio, Climate change in Pakistan, Climate change in Russia, Climate change in Saskatchewan, Climate change in South Carolina, Climate change in South Korea, Climate change in Sweden, Climate change in Tennessee, Climate change in Texas, Climate change in the Arctic, Climate change in the Caribbean, Climate change in the United Kingdom, Climate change in the United States, Climate change in Turkey, Climate change in Tuvalu, Climate change in Vietnam, Climate change in Virginia, Climate change in Washington, Climate change in West Virginia, Climate change in Wyoming, Climate Change Levy, Climate change mitigation, Climate change mitigation scenarios, Climate change opinion by country, Climate Change Performance Index, Climate change policy of California, Climate change policy of the George W. Bush administration, Climate change policy of the United States, Climate Change Research Centre, Climate Change Response (Emissions Trading) Amendment Act 2008, Climate Change Response (Zero Carbon) Amendment Bill, Climate Change Response Act 2002, Climate change scenario,

Climate change skepticism and denial, Climate Change TV, Climate change, industry and society, Climate Change: Global Risks, Challenges and Decisions, Climate Commission, Climate Council, Climate crisis, Climate debt, Climate Denial Crock of the Week, Climate Disclosure Standards Board, Climate emergency declaration, Climate engineering, Climate ethics, Climate fiction, Climate footprint, Climate gap, Climate governance, Climate Hawks Vote, Climate Hustle, Climate inertia, Climate Institute of Australia, Climate Investment Funds, Climate justice, Climate Justice Action, Climate Justice Now!, Climate Law and Governance Initiative, Climate legislation, Climate movement, Climate Policy (journal), Climate resilience, Climate restoration, Climate risk, Climate risk management, Climate Rush, Climate Science Rapid Response Team, Climate security, Climate Solutions Road Tour, Climate spiral, Climate system, Climate Vulnerability Monitor, Climate Vulnerable Forum, Climate Week NYC, Climate-Alliance Germany, Climate-friendly gardening, Climate-smart agriculture, Climatic Research Unit, Climatic Research Unit documents, Climatic Research Unit email controversy, Cloud formation and climate change, Co-benefits of climate change mitigation, CO2 Coalition, CO2 fertilization effect, CO2 is Green, Committee on Climate Change, Committee on Climate Change Science and Technology Integration, Conservatory (greenhouse), Cool It: The Skeptical Environmentalist's Guide to Global Warming, Copenhagen Climate Challenge, Criticism of the IPCC Fourth Assessment Report, David Parker (climatologist), Debate over China's economic responsibilities for climate change mitigation, Decarbonisation measures in proposed UK electricity market reform, Deep Decarbonization Pathways Project, Deforestation and climate change, Delta 3 greenhouse, Department of Communications, Climate Action and Environment, Department of Energy and Climate Change, Description of the Medieval Warm Period and Little Ice Age in IPCC reports, Directorate-General for Climate Action, Drawdown (climate), Durban Industry Climate Change Partnership Project, East Asia Climate Partnership, Economic impacts of climate change, Economics of climate change mitigation, Economics of global warming, Economists' Statement on Climate Change, Effects of climate change on island nations, Effects of climate change on plant biodiversity, Effects of climate change on terrestrial animals, Effects of climate change on wine production, Effects of global warming, Effects of global warming on human health, Effects of global warming on humans, Effects of global warming on marine mammals, Effects of global warming on oceans, Effects of global warming on South Asia, Effects of global warming on Sri Lanka, Effects of global warming on the United Arab Emirates, Euro-Mediterranean Center on Climate Change, European Assembly for Climate Justice, European Climate Change Programme, European Climate Exchange, European Climate Forum, European Climate Foundation, Evangelical Climate Initiative, Extinction risk from global warming, ExxonMobil climate change controversy, Fisheries and climate change, Fluorocarbon, Fourth National Climate Assessment, Freedom of Information requests to the Climatic Research Unit, G8 Climate Change Roundtable, Garnaut Climate Change Review, Geostationary Carbon Cycle Observatory, German Climate Action Plan 2050, German Climate Consortium, Glacial earthquake, Glacial survival hypothesis, Global Atmosphere Watch, Global Carbon Project, Global Centre of Excellence on Climate Adaptation, Global Climate Action Summit, Global Climate Coalition, Global Climate Network, Global climate regime, Global Historical Climatology Network, Global Roundtable on Climate Change, Global warming, Global warming conspiracy theory, Global warming controversy, Global warming game, Global warming hiatus, Global warming in Antarctica, Global warming in Belgium, Global warming in Luxembourg, Global warming in Norway, Global warming in popular culture, Global Warming Policy Foundation, Global Warming Pollution Reduction Act of 2007, Global warming potential, Global Warming Solutions Act of 2006, Global Warming: The Signs and The Science, Global Warming: What You Need to Know, Glossary of climate change, Gold Standard (carbon offset standard), Great March for Climate Action, Green Climate Fund, Green New Deal, Green Zone Community Climate Action, Greenhouse, Greenhouse debt, Greenhouse Development Rights, Greenhouse effect, Greenhouse gas, Greenhouse gas accounting, Greenhouse gas emissions accounting, Greenhouse gas emissions by Australia, Greenhouse gas emissions by China, Greenhouse gas emissions by India, Greenhouse gas emissions by Russia, Greenhouse gas emissions by the United Kingdom, Greenhouse gas emissions by the United States, Greenhouse gas emissions by Turkey, Greenhouse gas emissions in Kentucky, Greenhouse gas footprint, Greenhouse gas inventory, Greenhouse gas monitoring, Greenhouse Gas Pollution Pricing Act, Greenhouse Gases Observing Satellite, Greenhouse Mafia, Hadley Centre for Climate Prediction and Research, High Level Advisory Group on Climate Financing, Historical impacts of climate change, How Global Warming Works, Human Rights and Climate Change, Index of climate change articles, Indian Network on Climate Change Assessment, Indian Youth Climate Network, Individual action on climate change, Individual and political action on climate change, InsideClimate News, Integrated Carbon Observation System, Intergovernmental Panel on Climate Change, Interim Climate Change Committee, International Conference on Climate Change, International Journal of Greenhouse Gas Control, IPCC Fifth Assessment Report, IPCC First Assessment Report, IPCC Fourth Assessment Report, IPCC list of greenhouse gases, IPCC Second Assessment Report, IPCC Summary for Policymakers, IPCC supplementary report, 1992, IPCC Third Assessment Report, Journal for Geoclimatic Studies, Laboratoire des sciences du climat et de l'environnement, Land surface effects on climate, Last Glacial Maximum, Life-cycle greenhouse-gas emissions of energy sources, List of authors of Climate Change 2007: The

Physical Science Basis, List of climate change books, List of climate change initiatives, List of climate engineering topics, List of climate scientists, List of countries by carbon dioxide emissions, List of countries by carbon dioxide emissions per capita, List of countries by greenhouse gas emissions, List of countries by greenhouse gas emissions per capita, List of European power companies by carbon intensity, List of ministers of climate change, List of school climate strikes, List of U.S. states and territories by carbon dioxide emissions, London Climate Change Agency, Long-term effects of global warming, Low-carbon diet, Low-carbon economy, Mayors National Climate Action Agenda, Media coverage of global warming, Midwestern Greenhouse Gas Reduction Accord, Minister for Climate Change (New Zealand), Ministry of Climate and Energy (Denmark), Ministry of Climate and Environment (Norway), Ministry of Economic Affairs and Climate Policy, Ministry of Energy, Science, Technology, Environment and Climate Change (Malaysia), Ministry of Environment, Forest and Climate Change, Mitigation of global warming in Australia, Mycorrhizae and changing climate, National Climate Assessment, National Climatic Data Center, National Oceanic and Atmospheric Administration Climate and Societal Interactions Program, New England Governors and Eastern Canadian Premiers Climate Change Action Plan 2001, New South Wales Greenhouse Gas Abatement Scheme, North American Carbon Program, NZ Climate Party, Ocean acidification in the Arctic Ocean, Orbiting Carbon Observatory, Orbiting Carbon Observatory 2, Orbiting Carbon Observatory 3, Ozone, Paleoclimatology, Pan-African Media Alliance on Climate Change, Pastoral Greenhouse Gas Research Consortium, People's Climate Movement, Perfluorocarbon tracer, Peter Thorne (climatologist), Phil Jones (climatologist), Physical impacts of climate change, Physical properties of greenhouse gases, Political economy of climate change, Politics of global warming, Portal:Global warming, Premier's Climate Change Council, Presbyterian Church (U.S.A.) Carbon Neutral Resolution, Presidential Climate Action Plan, Program on Energy Efficiency in Artisanal Brick Kilns in Latin America to Mitigate Climate Change, Public opinion on global warming, Pyrogenic carbon capture and storage, Regional climate change initiatives in the United States, Regional effects of global warming, Regional Greenhouse Gas Initiative, Regulation of greenhouse gases under the Clean Air Act, Renewable Energy Sources and Climate Change Mitigation, Ringed seals and climate change, Royal Greenhouses of Laeken, Running on Climate, San Francisco Climate Action Plan, School strike for climate, Scientific consensus on climate change, Scorcher: The Dirty Politics of Climate Change, Sea level rise, Seawater greenhouse, September 2019 climate strikes, Soft climate change denial, Soil carbon feedback, South Pacific Sea Level and Climate Monitoring Project, Space mirror (climate engineering), Special Report on Climate Change and Land, Special Report on the Ocean and Cryosphere in a Changing Climate, Stop Climate Chaos, Stop Climate Chaos Scotland, Stratospheric Particle Injection for Climate Engineering, Table of historic and prehistoric climate indicators, Talk:Climate change (general concept), Template:Climate change in Canada, Template:Climate-change-stub, Template:United Nations climate change conferences, The Carbon Principles, The Climate Corporation, The Climate Group, The Climate Mobilization, The Climate Monologues, The Climate Reality Project, The Climate Registry, The Doubt Machine: Inside the Koch Brothers' War on Climate Science, The Great Derangement: Climate Change and the Unthinkable, The Greenhouse Conspiracy, Tianjin Climate Exchange, Total equivalent warming impact, Transarctica, Tropical cyclones and climate change, U.S. Climate Action Partnership, U.S. Climate Change Technology Program, UK Youth Climate Coalition, United Kingdom Climate Change Programme, United Nations Climate Change conference, United Nations Special Envoy on Climate Change, United States Climate Alliance, United States federal register of greenhouse gas emissions, United States House Select Committee on Energy Independence and Global Warming, United States House Select Committee on the Climate Crisis, Valleyfield greenhouse, Vatican Climate Forest, Warming stripes, Western Climate Initiative, Weyburn-Midale Carbon Dioxide Project, White House Office of Energy and Climate Change Policy, World Climate Change Conference, Moscow, World Climate Conference, World People's Conference on Climate Change, World Wide Views on Global Warming, Wuppertal Institute for Climate, Environment and Energy, Zero Carbon World.

List of English Wikipedia articles used to track public engagement in health:

1793 Philadelphia yellow fever epidemic, 1837 Great Plains smallpox epidemic, 1847 North American typhus epidemic, 1916 New York City polio epidemic, 1968 flu pandemic, 1974 smallpox epidemic in India, 1983 West Bank fainting epidemic, 1998 Winter Olympics flu epidemic, 2009 flu pandemic, 2009 flu pandemic by country, 2009 flu pandemic in India, 2009 flu pandemic timeline, 2013 Swansea measles epidemic, 2019 Kuala Koh measles outbreak, 2019 New York measles outbreak, 2019 Pacific Northwest measles outbreak, 2019 Philippines measles outbreak, 2019 Samoa measles outbreak, 2019 Tonga measles outbreak, Academy of Nutrition and Dietetics, Acute eosinophilic pneumonia, Adenovirus infection, Adult-onset Still's disease, Advances in Nutrition, Affordable Medicines Facility-malaria, Africa Fighting Malaria, African Malaria Network Trust, African Nutrition Leadership Programme, Against Malaria Foundation, Agency for Toxic Substances and Disease Registry, Air pollution, Air pollution and traffic congestion in Tehran, Air pollution

forecasting, Air pollution in Hong Kong, Air pollution in Macau, Air pollution sensor, Airborne disease, Airport malaria, Alabama Department of Public Health, Alan Howard (nutritionist), Alexander disease, All India Institute of Hygiene and Public Health, Alveolar hydatid disease, Alzheimer Disease and Associated Disorders, Alzheimer's disease biomarkers, Alzheimer's Disease Cooperative Study, Alzheimer's disease in the media, Alzheimer's Disease Neuroimaging Initiative, Amazon Malaria Initiative, America's Health Care Crisis Solved, American Association of Public Health Dentistry, American Association of Public Health Physicians, American College of Epidemiology, American Journal of Epidemiology, American Public Health Association, American Society for Nutrition, American Society for Parenteral and Enteral Nutrition, Anaerobic infection, Andersen healthcare utilization model, Animal nutrition, Animal nutritionist, Annals of Epidemiology, Annual Review of Nutrition, Anthroponotic disease, Anti-AQP4 disease, Anti-IgLON5 disease, Antidiarrhoeal, Antimalarial medication, Apparent infection rate, Applied Physiology, Nutrition, and Metabolism, Asia Pacific Leaders Malaria Alliance, Asia Pacific Malaria Elimination Network, Aspiration pneumonia, Association for Nutrition, Association of Medical Microbiology and Infectious Disease Canada, Association of Public Health Laboratories, Ateneo School of Medicine and Public Health, Atypical pneumonia, Australian Measles Control Campaign, Autoimmune disease, Autoimmune disease in women, Autoimmune inner ear disease, Autosomal dominant polycystic kidney disease, Autosomal recessive polycystic kidney disease, Awards and decorations of the Public Health Service, Bacterial pneumonia, Balwadi Nutrition Programme, Bangladesh National Nutrition Council, Batten disease, Baumol's cost disease, Behavior change (public health), Belgian Health Care Knowledge Centre, BENTA disease, Bills of mortality, Binswanger's disease, Biochemistry of Alzheimer's disease, Biologically based mental illness, Biomarker epidemiology, Biphasic disease, Blackheart (plant disease), Blood-borne disease, Blount's disease, Bluetongue disease, Bombay plague epidemic, British Journal of Nutrition, Bronchopneumonia, Caerphilly Heart Disease Study, Calcium pyrophosphate dihydrate crystal deposition disease, California Center for Public Health Advocacy, California Department of Health Care Services, California Department of Public Health, Canadian Institute of Public Health Inspectors, Canadian Public Health Association, Canadian Society for Epidemiology and Biostatistics, Canavan disease, Cancer Epidemiology (journal), Cancer Epidemiology, Biomarkers & Prevention, Canine vector-borne disease, Capitation (healthcare), Cardiovascular disease, Caribbean Public Health Agency, Caroli disease, Carolinas HealthCare System Blue Ridge Morganton, Carrion's disease, Cat-scratch disease, Catheter-associated urinary tract infection, Causes of mental disorders, Cavitary pneumonia, Center for Infectious Disease Research, Center for Infectious Disease Research and Policy, Center for Public Health Preparedness, Centers for Disease Control (Taiwan), Centers for Disease Control and Prevention, Centers for Disease Control and Prevention timeline, Centre for History in Public Health, London School of Hygiene and Tropical Medicine, Chagas disease, Chelates in animal nutrition, Chicago 1885 cholera epidemic myth, Chicago Department of Public Health, Child Health and Nutrition Research Initiative, Child mortality, Childhood chronic illness, Children's right to adequate nutrition in New Zealand, Chinese Classification of Mental Disorders, Cholera outbreaks and pandemics, Chronic diseases, Chronic illness, Chronic Lyme disease, Cinematography in healthcare, Classification of mental disorders, Classification of pneumonia, Clinical epidemiology, Clinical Epidemiology (journal), Clinical nutrition, Clinton health care plan of 1993, Clostridioides difficile infection, CNS demyelinating autoimmune diseases, Coalition for Epidemic Preparedness Innovations, Cocoliztli epidemics, Cognitive epidemiology, Coinfection, Cold agglutinin disease, Colorado Department of Health Care Policy and Financing, Colorado Department of Public Health and Environment, Commission on the Accreditation of Healthcare Management Education, Common disease-common variant, Communicable diseases, Community Dentistry and Oral Epidemiology, Community-acquired pneumonia, Comorbidity, Comparison of the healthcare systems in Canada and the United States, Compartmental models in epidemiology, Compression of morbidity, Computational epidemiology, Conflict epidemiology, Congenital cytomegalovirus infection, Congenital malaria, Contagious bovine pleuropneumonia, Contagious disease, Convention on Long-Range Transboundary Air Pollution, Corn stunt disease, Council on Education for Public Health, Critical illness insurance, Critical Reviews in Food Science and Nutrition, Crohn's disease, Cryptic infection, Cryptogenic organizing pneumonia, CUNY Graduate School of Public Health & Health Policy, Cytomegaloviral disease, Cytomegalovirus infection, Dalla Lana School of Public Health, Degenerative disease, Dementia and Alzheimer's disease in Australia, Depression of Alzheimer disease, Desquamative interstitial pneumonia, Developmental disorder, Diagnosis of malaria, Diagnostic and Statistical Manual of Mental Disorders, Diarrheal diseases, Disease, Disease burden, Disease cluster, Disease Control Priorities Project, Disease diffusion mapping, Disease management (health), Disease resistance, Disease surveillance, Disease X, Diseases, Diseases of abnormal polymerization, Diseases of despair, Diseases of poverty, Doctor of Public Health, Dole Nutrition Institute, Drugs for Neglected Diseases Initiative, Dukes' disease, Dust pneumonia, E-epidemiology, Early-onset Alzheimer's disease, Ebola virus disease, Ebola virus disease in Mali, Ebola virus disease in Spain, Ebola virus disease in the United Kingdom, Ebola virus disease treatment research, Ebola virus epidemic in Guinea, Ebola virus epidemic in Liberia, Ebola virus epidemic in Sierra Leone, Economic epidemiology, Ehrlichiosis ewingii infection, EMBRACE Healthcare Reform Plan, Emerging infectious disease,

Emerging Infectious Diseases (journal), Emerging Themes in Epidemiology, Endemic (epidemiology), Endogenous infection, Environmental disease, Environmental epidemiology, Eosinophilic pneumonia, Ephialtes (illness), Epidemic, Epidemic curve, Epidemic Intelligence Service, Epidemic models on lattices, Epidemic polyarthritis, Epidemic typhus, Epidemiology, Epidemiology (journal), Epidemiology and Infection, Epidemiology and Psychiatric Sciences, Epidemiology data for low-linear energy transfer radiation, Epidemiology in Country Practice, Epidemiology of asthma, Epidemiology of attention deficit hyperactive disorder, Epidemiology of bed bugs, Epidemiology of binge drinking, Epidemiology of breast cancer, Epidemiology of cancer, Epidemiology of chikungunya, Epidemiology of child psychiatric disorders, Epidemiology of childhood obesity, Epidemiology of depression, Epidemiology of diabetes, Epidemiology of malnutrition, Epidemiology of measles, Epidemiology of metabolic syndrome, Epidemiology of plague, Epidemiology of pneumonia, Epidemiology of schizophrenia, Epidemiology of syphilis, Eradication of infectious diseases, Escape Fire: The Fight to Rescue American Healthcare, Essence (Electronic Surveillance System for the Early Notification of Community-based Epidemics), European Centre for Disease Prevention and Control, European Journal of Clinical Nutrition, European Journal of Epidemiology, European Journal of Nutrition, European Parliament Committee on the Environment, Public Health and Food Safety, European Programme for Intervention Epidemiology Training, European Prospective Investigation into Cancer and Nutrition, European Public Health Alliance, European Public Health Association, European Society for Clinical Nutrition and Metabolism, European Society for Paediatric Infectious Diseases, European Society of Clinical Microbiology and Infectious Diseases, European Working Group for Legionella Infections, Evolution of Infectious Disease, Evolutionary epidemiology, Experimental epidemiology, Fair Share Health Care Act, Febrile infection-related epilepsy syndrome, Federation of European Nutrition Societies, Field Epidemiology Training Program, Fifth disease, Fire breather's pneumonia, First Nations nutrition experiments, Focal infection theory, Focus of infection, Food & Nutrition Research, Food and Nutrition Bulletin, Food pyramid (nutrition), Foodborne illness, Foot-and-mouth disease, Free-market healthcare, Fungal pneumonia, Gastrointestinal disease, Genetic epidemiology, Genetic Epidemiology (journal), Geospatial Measurements of Air Pollution, Germ theory of disease, GIS and public health, Global Acute Malnutrition, Global Alliance for Improved Nutrition, Global Burden of Disease Study, Global Coalition Against Pneumonia, Global Infectious Disease Epidemiology Network, Global Malaria Action Plan, Global Network for Neglected Tropical Diseases, Global Public Health Intelligence Network, Global Research Collaboration for Infectious Disease Preparedness, Globalization and disease, Gram-negative bacterial infection, Graves' disease, Groningen epidemic, Group B streptococcal infection, Hanoi School of Public Health, Health care access among Dalits in India, Health Care Card, Health Care Compact, Health care efficiency measures, Health care finance in the United States, Health Care for Women International, Health care fraud, Health care in Argentina, Health care in Australia, Health Care in Canada Survey, Health care in Colombia, Health care in Cyprus, Health care in France, Health care in Karachi, Health care in Mozambique, Health care in New Zealand, Health care in Poland, Health care in Saudi Arabia, Health care in Spain, Health care in Sweden, Health care in the Philippines, Health care in the United Kingdom, Health care in the United States, Health care in Turkey, Health care in Venezuela, Health care prices in the United States, Health care ratings, Health care rationing, Health care reforms proposed during the Obama administration, Health care system in Japan, Health care system of the elderly in Germany, Health care systems by country, Health care time and motion study, Healthcare availability for undocumented immigrants in the United States, Healthcare in Afghanistan, Healthcare in Albania, Healthcare in Algeria, Healthcare in Austria, Healthcare in Azerbaijan, Healthcare in Bahrain, Healthcare in Belgium, Healthcare in Brazil, Healthcare in Canada, Healthcare in China, Healthcare in Croatia, Healthcare in Cuba, Healthcare in Denmark, Healthcare in Egypt, Healthcare in Estonia, Healthcare in Ethiopia, Healthcare in Finland, Healthcare in Georgia (country), Healthcare in Germany, Healthcare in Ghana, Healthcare in Greece, Healthcare in Hungary, Healthcare in Iceland, Healthcare in India, Healthcare in Indonesia, Healthcare in Iran, Healthcare in Iraq, Healthcare in Israel, Healthcare in Italy, Healthcare in Kenya, Healthcare in Kuwait, Healthcare in Luxembourg, Healthcare in Madagascar, Healthcare in Malawi, Healthcare in Malaysia, Healthcare in Malta, Healthcare in Mexico, Healthcare in Moldova, Healthcare in Nicaragua, Healthcare in Nigeria, Healthcare in Norway, Healthcare in Pakistan, Healthcare in Panama, Healthcare in Peru, Healthcare in Portugal, Healthcare in Qatar, Healthcare in Romania, Healthcare in Russia, Healthcare in Rwanda, Healthcare in Saint Helena, Healthcare in San Marino, Healthcare in Senegal, Healthcare in Serbia, Healthcare in Sierra Leone, Healthcare in Singapore, Healthcare in Slovakia, Healthcare in Slovenia, Healthcare in South Africa, Healthcare in South Korea, Healthcare in Switzerland, Healthcare in Taiwan, Healthcare in Tanzania, Healthcare in Thailand, Healthcare in the Czech Republic, Healthcare in the Isle of Man, Healthcare in the Netherlands, Healthcare in the Republic of Ireland, Healthcare in the State of Palestine, Healthcare in the United Arab Emirates, Healthcare in Tristan da Cunha, Healthcare in Uganda, Healthcare in Ukraine, Healthcare in Zambia, Healthcare rationing in the United States, Healthcare reform debate in the United States, Healthcare reform in China, Healthcare reform in the United States, Healthcare shortage area, Healthcare Spending Account, Healthcare transport, Healthcare UK, HealthCare Volunteer,

HealthCare.gov, History of emerging infectious diseases, History of health care reform in the United States, History of malaria, History of mental disorders, History of USDA nutrition guides, Holozoic nutrition, Home health care software, Homosexuality as a disease, Hookworm infection, Hospital-acquired infection, Hospital-acquired pneumonia, How to Have Sex in an Epidemic, Human genetic resistance to malaria, Human nutrition, Human papillomavirus infection, Hypertensive disease of pregnancy, ICAN: Infant, Child, & Adolescent Nutrition, Idiopathic disease, Idiopathic interstitial pneumonia, Idiopathic multicentric Castleman disease, Idiopathic pneumonia syndrome, IgG4-related disease, Illinois Department of Healthcare and Family Services, Illinois Department of Public Health, Illness, Imagine No Malaria, Immigrant health care in the United States, Indian Public Health Association, Indiana University School of Public Health-Bloomington, Indoor air pollution in developing nations, Inequality in disease, Infant mortality, Infant nutrition, Infection, Infection control, Infection Control Society of Pakistan, Infection rate, Infections associated with diseases, Infectious causes of cancer, Infectious coryza in chickens, Infectious disease (athletes), Infectious disease (medical specialty), Infectious Disease (Notification) Act 1889, Infectious Disease Pharmacokinetics Laboratory, Infectious Disease Research Institute, Infectious diseases, Infectious Diseases Institute, Infectious Diseases Society of America, Inflammatory bowel disease, Inflammatory demyelinating diseases of the central nervous system, Influenza pandemic, Integrated disease surveillance program, Integrated Management of Childhood Illness, Intermountain Healthcare, International Association of National Public Health Institutes, International Conference on Emerging Infectious Diseases, International Journal of Behavioral Nutrition and Physical Activity, International Journal of Epidemiology, International Journal of Sport Nutrition and Exercise Metabolism, International Lyme and Associated Diseases Society, International Partnership on Avian and Pandemic Influenza, International Society for Environmental Epidemiology, International Society for Infectious Diseases in Obstetrics and Gynaecology, International Society for Pharmacoepidemiology, International Statistical Classification of Diseases and Related Health Problems, International Union of Air Pollution Prevention and Environmental Protection Associations, International Union of Nutritional Sciences, Intestinal infectious diseases, Iron Triangle of Health Care, Isolation (health care), Jembrana disease, Jennifer McMahon (nutritionist), Jiann-Ping Hsu College of Public Health, Journal of Alzheimer's Disease, Journal of Clinical Epidemiology, Journal of Epidemiology, Journal of Epidemiology and Biostatistics, Journal of Epidemiology and Community Health, Journal of Exposure Science and Environmental Epidemiology, Journal of Human Nutrition and Dietetics, Journal of Nutrition, Journal of Nutritional Biochemistry, Journal of Parenteral and Enteral Nutrition, Journal of the Academy of Nutrition and Dietetics, Jurosomatic illness, Kawasaki disease, Krabbe disease, Kyasanur Forest disease, Landscape epidemiology, Legionnaires' disease, Leveraging Agriculture for Improving Nutrition and Health, Lipid pneumonia, List of autoimmune diseases, List of diseases eliminated from the United States, List of epidemics, List of foodborne illness outbreaks, List of foodborne illness outbreaks by death toll, List of infections of the central nervous system, List of infectious diseases, List of infectious diseases causing flu-like syndrome, List of Legionnaires' disease outbreaks, List of medical professionals who died during the SARS outbreak, List of mental disorders, List of national public health agencies, List of pneumonia deaths, List of sexually transmitted infections by prevalence, List of types of malnutrition, Liverpool Neurological Infectious Diseases Course, Lobar pneumonia, Localized disease, London Declaration on Neglected Tropical Diseases, Los Angeles County Department of Public Health, Lower respiratory tract infection, Lung disease, Lyme disease, Lyme disease microbiology, Lymphocytic interstitial pneumonia, Lysosomal storage disease, Madras motor neuron disease, Makerere University School of Public Health, Malaria, Malaria and the Caribbean, Malaria antigen detection tests, Malaria Atlas Project, Malaria Consortium, Malaria Control Project, Malaria culture, Malaria Day in the Americas, Malaria Eradication Scientific Alliance, Malaria Journal, Malaria No More, Malaria No More UK, Malaria Policy Advisory Committee, Malaria prophylaxis, Malaria vaccine, Malarial nephropathy, MalariaWorld, Malaysian Journal of Nutrition, Malnutrition, Malnutrition in India, Malnutrition in Kerala, Malnutrition in Peru, Malnutrition in South Africa, Malnutrition in Zimbabwe, Management of Crohn's disease, Managerial epidemiology, Marburg virus disease, Mass psychogenic illness, Massachusetts Department of Public Health, Massachusetts smallpox epidemic, Maternal mortality ratio, Mayaro virus disease, Measles, Measles & Rubella Initiative, Measles hemagglutinin, Measles morbillivirus, Measles resurgence in the United States, Measles vaccine, Medical students' disease, Medicines for Malaria Venture, Mekong Basin Disease Surveillance, Melanie's Marvelous Measles, Meningococcal disease, Mental disorder, Mental disorders and gender, Mental disorders in film, Mental illness, Mental illness portrayed in media, Michael Colgan (nutritionist), Micronutrient malnutrition, Mitochondrial disease, Mixed connective tissue disease, Mobile source air pollution, Modern Healthcare, Molecular epidemiology, Molecular Nutrition & Food Research, Morbidity and Mortality Weekly Report, Mortality Medical Data System, Mosquito-borne disease, Mosquito-malaria theory, Motor neuron disease, Motor Neurone Disease Association, Muesli belt malnutrition, Multiple complex developmental disorder, Multisystem developmental disorder, Music therapy for Alzheimer's disease, Mycobacterium avium-intracellulare infection, Mycoplasma hominis infection, Mycoplasma pneumonia, National Air Pollution Symposium, National Association for Public Health Policy, National Center for Disease Control and Public

Health (Georgia), National Center for Immunization and Respiratory Diseases, National Center for Infectious Diseases, National Comorbidity Survey, National Emerging Infectious Diseases Laboratories, National Foundation for Infectious Diseases, National Health and Nutrition Examination Survey, National Hotel disease, National Institute for Communicable Diseases, National Institute of Malaria Research, National Malaria Eradication Program, National Perinatal Epidemiology Unit, National School of Public Health (Spain), Native American disease and epidemics, Neglected tropical disease research and development, Neglected tropical diseases, Neonatal infection, Neurodevelopmental disorder, Neuroepidemiology (journal), NewYork-Presbyterian Healthcare System, NINCDS-ADRDA Alzheimer's Criteria, Noma (disease), Non-alcoholic fatty liver disease, Non-communicable disease, Non-communicable diseases, Non-specific interstitial pneumonia, Norwegian Institute of Public Health, Notifiable disease, Notifiable diseases in Sweden, Notifiable diseases in the United Kingdom, Nutrition, Nutrition (journal), Nutrition and Cancer, Nutrition and Education International, Nutrition and Health, Nutrition Foundation of the Philippines, Nutrition in Clinical Practice, Nutrition Journal, Nutrition Reviews, Nutrition transition, Nutritional Neuroscience (journal), NutritionDay, Nutritionist, Occult pneumonia, Occupational exposure to Lyme disease, Opportunistic infection, Outline of air pollution dispersion, Overwhelming post-splenectomy infection, Oxford Brookes Centre for Nutrition and Health, Paediatric and Perinatal Epidemiology, Paget's disease of bone, Pandemic, Pandemic Preparedness and Response Act, Pandemic severity index, Papaya Bunchy Top Disease, Parasitic disease, Parasitic pneumonia, Pay for performance (healthcare), Pelvic inflammatory disease, Pervasive developmental disorder, Pervasive developmental disorder not otherwise specified, Peyronie's disease, Pick's disease, Pinta (disease), Plague (disease), Plague City: SARS in Toronto, Plant nutrition, Plum Island Animal Disease Center, Pneumococcal infection, Pneumococcal pneumonia, Pneumocystis pneumonia, Pneumonia, Pneumonia (non-human), Pneumonia jacket, Pneumonia severity index, Pogosta disease, Postorgasmic illness syndrome, Prebiotic (nutrition), Pregnancy-associated malaria, President's Malaria Initiative, Prevalence of mental disorders, Preventing Chronic Disease, Prime Healthcare Services, Professional degrees of public health, Professional Further Education in Clinical Pharmacy and Public Health, Progressive disease, Psychiatric epidemiology, Psychogenic disease, Public health, Public Health Advisor, Public Health Agency of Canada, Public Health Agency of Sweden, Public health emergency (United States), Public Health Emergency of International Concern, Public Health England, Public Health Foundation of India, Public health genomics, Public Health Information Network, Public health insurance option, Public health intervention, Public health laboratory, Public Health Nutrition, Public health observatory, Public health problems in the Aral Sea region, Public Health Service Act, Public health surveillance, Public health system in India, Publicly funded health care, Rare disease, Real-time outbreak and disease surveillance, Refugee health care in Canada, Reproductive health care for incarcerated women in the United States, Reproductive system disease, Respiratory disease, Respiratory diseases, Respiratory tract infection, Responses to the West African Ebola virus epidemic, Rheumatoid disease of the spine, Ron Rivera (public health), Royal Commission on the Future of Health Care in Canada, Rural health care in Australia, San Francisco Department of Public Health, Saskatchewan Disease Control Laboratory, School health and nutrition services, Second plague pandemic, Sentara Healthcare, Serratia infection, Services for mental disorders, Shona Holmes health care incident, Single-payer healthcare, Skin and skin structure infection, Skin infection, Smallpox epidemic, Social Psychiatry and Psychiatric Epidemiology, Sociality and disease transmission, Society of Infectious Diseases Pharmacists, South African Malaria Initiative, South Texas Center for Emerging Infectious Diseases, Southern tick-associated rash illness, Spatial and Spatio-temporal Epidemiology, Specific replant disease, Stateville Penitentiary Malaria Study, Strengthening the reporting of observational studies in epidemiology, Streptococcus pneumoniae, Subclinical infection, Suicide epidemic, Superinfection, Surgical Infections, Susceptibility and severity of infections in pregnancy, Sweating sickness epidemics, Systemic disease, Target Malaria, Template:Acari-borne diseases, Template:Ebola virus disease epidemic, Template:Eradication of infectious disease, Template:Gram-positive actinobacteria diseases, Template:Infectious disease, Template:Infectious-disease-stub, Template:Pervasive developmental disorders, Template:Plant nutrition, Template:Vertically transmitted infection, Tenet Healthcare, The Global Fund to Fight AIDS, Tuberculosis and Malaria, The Journal of Nutrition, Health and Aging, Theiler's disease, Third plague pandemic, Tick-borne disease, Tick-Borne Disease Alliance, Timeline of Alzheimer's disease, Timeline of healthcare in China, Timeline of healthcare in Cuba, Timeline of healthcare in Egypt, Timeline of healthcare in Ethiopia, Timeline of healthcare in France, Timeline of healthcare in Germany, Timeline of healthcare in India, Timeline of healthcare in Italy, Timeline of healthcare in Japan, Timeline of healthcare in Kenya, Timeline of healthcare in Nigeria, Timeline of healthcare in Russia, Timeline of healthcare in South Africa, Timeline of healthcare in the Democratic Republic of the Congo, Timeline of healthcare in the United Kingdom, Timeline of malaria, Timeline of measles, Top dying disease, Traditional Healthcare, Tropical disease, U.S.-Mexico Border Infectious Disease Surveillance Project, UCSC Malaria Genome Browser, Undernutrition, United Kingdom aid efforts for the 2014 Ebola epidemic in West Africa, United States Public Health Service, Universal Declaration on the Eradication of Hunger and Malnutrition, University of Zambia School of Public Health, Usual interstitial pneumonia, Vaccine-preventable diseases,

Value-based health care, Vapours (disease), Vector (epidemiology), Venereal Disease Research Laboratory test, Ventilator-associated pneumonia, Vermont health care reform, Vertically transmitted infection, Very early onset inflammatory bowel disease, Veterinary public health, Viral pneumonia, Virgin soil epidemic, Waterborne Disease and Outbreak Reporting System, Waterborne diseases, Weather and climate effects on Lyme disease exposure, West African Ebola virus epidemic timeline, West African Ebola virus epidemic timeline of reported cases and deaths, Western African Ebola virus epidemic, WHO disease staging system for HIV infection and disease, WHO Disease Staging System for HIV Infection and Disease in Adults and Adolescents, WHO Disease Staging System for HIV Infection and Disease in Children, Wildlife trafficking and emerging zoonotic diseases, Wilson's disease, Wilt disease, Working Environment (Air Pollution, Noise and Vibration) Convention, 1977, World Malaria Day, World Pneumonia Day.

Complementing the analysis presented in the 2020 report of the Lancet Countdown, Figure 61 and Figure 62 provide the standalone network plots for the climate change and the health-related articles, respectively. Figure 63 presents the aggregate monthly health and climate change co-clicks on Wikipedia articles.

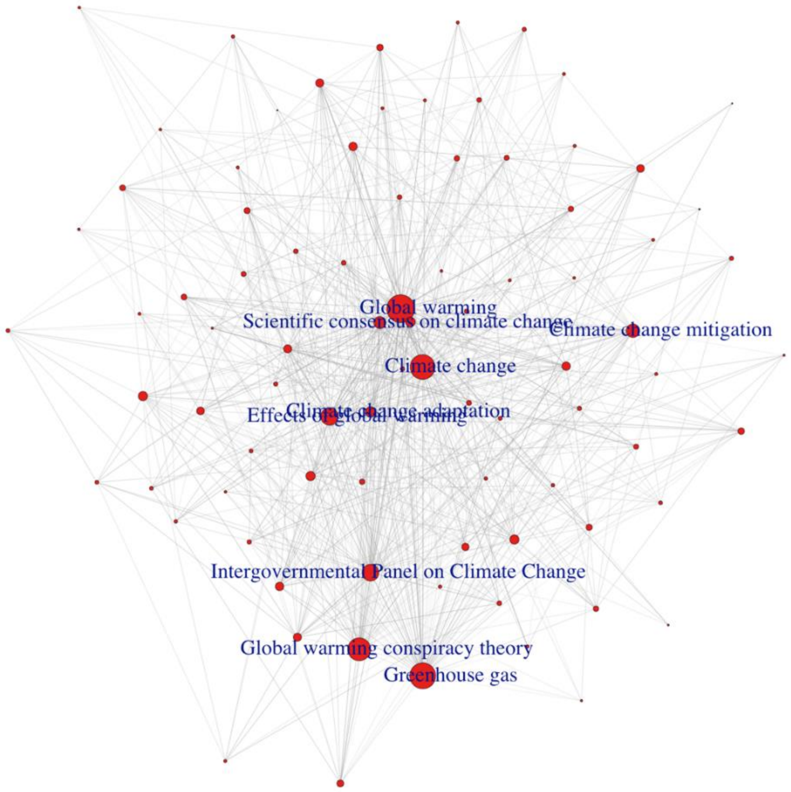

Figure 61: Connectivity graph of Wikipedia articles on climate change. Popularity of articles displayed by node size. Edges represent co-visits in the 2019 clickstream data.

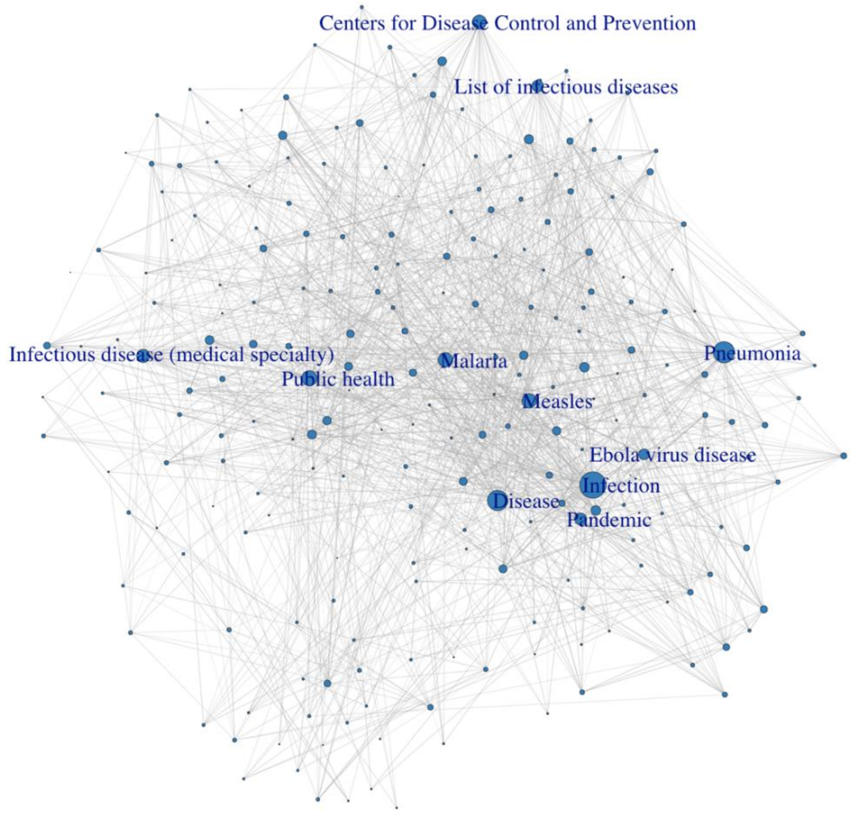

Figure 62: Connectivity graph of Wikipedia articles on health. Popularity of articles displayed by node size. Edges represent co-visits in the 2019 clickstream data.

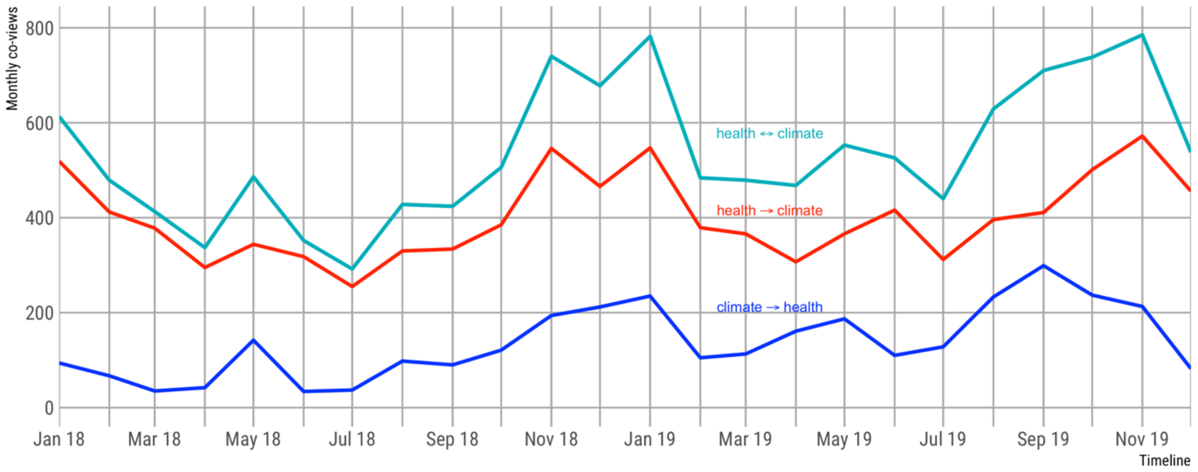

Figure 63: Aggregate monthly co-clicks on articles related to human health and climate change by Wikipedia users, 2018-2019.

Indicator 5.3: Coverage of Health and Climate Change in Scientific Journals

Methods

The inclusion of climate-related terms and their co-occurrence with health terms in scientific publications was tracked using a bibliometric search in both Ovid Medline (including Medline In-Process & Other Non-Indexed Citations for those citations not indexed) and Ovid Embase databases.

The Ovid Embase and Ovid Medline databases were selected due to their coverage of health, medical and biomedical sciences, with content that is predominantly journal articles. Ovid Medline contains 25 million citations from 5600 journals, while Ovid Embase is bigger with 32 million citations from 8,500 journals. Where Medline is predominantly health and biomedicine, Embase has a greater pharmaceutical focus, all of which are relevant to health and climate change. Both databases are updated online daily and can thus provide the annual data (with a 31 December cut-off each year) needed for the indicator. These databases also function through the sophisticated Ovid interface and allow access to the comprehensive indexing systems and thesaurus of Medical Subject Headings (MeSH) for Medline and Emtree for Embase.

Also considered for use were Science Direct and the Web of Science suite of databases, but, with broad subject coverage, these would not enable the necessary search precision.

By screening the retrieved articles between 2007 and 2019, those articles that contained both health and climate change terms in their title or abstract, but do not make any meaningful link between them, were excluded. A meaningful link here means some association between climate change and an aspect of health. This link may be the focus of the article or tangential to it. As an example, climate change may be mentioned at the end of an abstract, where it is noted the health topic that is the focus of the article (e.g. dengue fever distribution) is expected to worsen or change under climate change scenarios.

Data were extracted using search filters that function via Boolean operators (AND, OR, NOT) (see below for final search strategies). For purposes of consistency and efficiency of analysis, the majority of each search filter is designed to produce results with the search terms in either the title or abstract. Indeed, indexing is also likely to be poorly assigned or inconsistently assigned to references. The search filter is designed to retrieve all relevant results (high sensitivity) while keeping irrelevant results, and therefore effort on the part of the researchers, to a minimum (high precision).

To identify articles where associations are made between climate change and health, the filter was split into two facets, one for climate change and one for health. Terms that made up the filter were derived using both subjective and objective methods. Subjective methods included utilising terms already known by the research team, as well as those appearing in previous iterations of the Lancet Countdown. Objective methods included the use of online word frequency software (Writewords). Articles looking at health and climate change were run through this software, which organises the words or phrases in order of frequency, allowing relevant terms to be extracted.

Though this process was iterative, the climate change facet was undertaken first, as this was considered to likely consist of fewer terms and be comparatively less complex. All terms were tested independently and alongside other terms: that is, each was input into the OVID databases, from which samples of 100 were drawn and screened for relevance. Terms with high relevance were either piloted or adapted, to be tested alongside other terms and to restrict inclusion to records referring to human health. With different indexing systems, these were then translated between the databases. In addition, terms to ascertain results for editorials, comment sections, and letters were used to compare the volume of these against journal articles.

Estimates of sensitivity for the strategies were established by running the climate change facet through the Ovid interface alone, without the health facet. Samples of 1000 were then extracted and screened for relevant articles. The number of relevant articles found that were also found by the whole search strategy were divided by the number of total relevant articles found, giving an estimate of sensitivity in percentage form. For this indicator, the 90% sensitivity threshold required for systematic reviews was used (Beynon, 2013).

With an acceptable estimate of sensitivity (>90%), results of the search strategies were downloaded into Endnote and into two separate libraries: one for Medline (and Medline In-Process & Other Non-Indexed Citations), the other for Embase. Duplicates were removed from the individual libraries, before the libraries were merged and duplicates, shared across the library, were removed. The remaining records were screened for

inclusion based on the inclusion and exclusion criteria outlined above for articles making a meaningful link between health and climate change. Results were screened twice by the same researcher. In addition, another researcher screened a 10% sample to ensure the criteria were met. The step-wise process of the selection of articles can be seen in Figure 64.

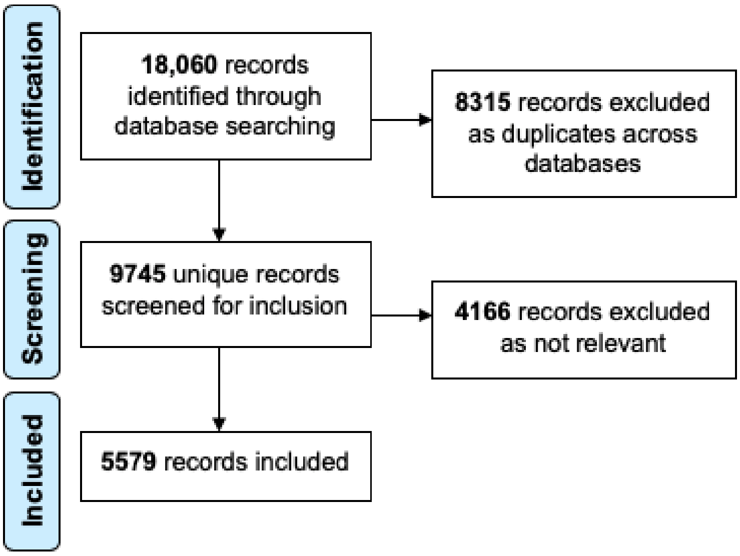

Figure 64: PRISMA flow diagram showing steps of selection process.

Numbers indicate the article count retained at each step of the process. With the applied search terms more than 18,000 scientific articles on health and climate change were identified for the period of 2007-2019. After the screening process, only 30.8% (n=5579) were retained and found to be relevant.

Following screening, precision was established by calculating the number of relevant records retrieved, divided by the total number of records retrieved. The development of the search strategy was repeated, and all of the necessary stages leading up to this point, until precision was established at over 50% for each database.

With an acceptable level of precision established for each database, the data were coded and organised in Endnote in a number of ways. To ascertain how articles were distributed geographically, they were organised by region (based on the institutional location of first author). Following this step, the articles were arranged into both World Health Organization regions and World Bank Income and Lending Groups, to give different visualisations of the distribution of scientific engagement across the world.

The data was also organised according to *type of manuscript*. Primary studies, developing new research, were separated from research-related articles (i.e. those discussing the implications or direction of research, such as editorials, commentaries and letters, and those comparing research within and between fields, such as reviews). Systematic reviews were also separated. This enabled an appreciation of the volume of new research (including primary research and systematic reviews) as compared to the volume of discussion around that research.

<u>Search terms</u>

**Medline**		**Medline (In-Process & Other Non-Indexed Citations)**		**Embase**	
**1**	carbon footprint*.ti,ab.	**1**	(climat* adj3 chang*).ti,ab.	**1**	(climat* adj3 chang*).ti,ab.
**2**	carbon footprint/	**2**	climate variability.ti,ab.	**2**	Climate Change/
**3**	(climat* adj3 chang*).ti,ab..	**3**	(climat* adj3 warming).ti,ab.	**3**	Greenhouse Effect/
**4**	climat* cris?s.ti,ab.	**4**	global warming.ti,ab.	**4**	greenhouse gas*.ti,ab.

**5**	climat* variability.ti,ab.	**5**	greenhouse effect*.ti,ab.	**5**	global warming.ti,ab.
**6**	climat* warming.ti,ab.	**6**	green house effect*.ti,ab.	**6**	Carbon Footprint/
**7**	exp Climate Change/	**7**	greenhouse gas*.ti,ab.	**7**	Greenhouse Gas/
**8**	GHG*.ti,ab.	**8**	(greenhouse adj2 emission*).ti,ab.	**8**	(greenhouse adj2 emission*).ti,ab.
**9**	global warming.ti,ab.	**9**	climat* model*.ti,ab.	**9**	(climat* adj3 warming).ti,ab.
**10**	greenhouse effect*.ti,ab.	**10**	climat* scenario*.ti,ab.	**10**	GHG*.ti,ab.
**11**	greenhouse effect/	**11**	green house emission*.ti,ab.	**11**	climat* model*.ti,ab.
**12**	greenhouse emission*.ti,ab.	**12**	GHG*.ti,ab.	**12**	climat* variability.ti,ab.
**13**	greenhouse gas*.ti,ab.	**13**	carbon footprint*.ti,ab.	**13**	carbon footprint*.ti,ab.
**14**	Greenhouse Gases/	**14**	climate induced.ti,ab.	**14**	climat* scenario*.ti,ab.
**15**	climate induced.ti,ab.	**15**	climat* cris?s.ti,ab.	**15**	greenhouse effect*.ti,ab.
**16**	climat* scenario*.ti,ab.	**16**	health.ti.	**16**	climate induced.ti,ab.
**17**	climat* model*.ti,ab.	**17**	disease*.ti.	**17**	climat* cris?s.ti,ab.
**18**	exp Health/	**18**	infectious.ti.	**18**	Ep.fs.
**19**	Global Health/	**19**	mortality.ti.	**19**	exp Malignant neoplasm/
**20**	health status/	**20**	healthy.ti.	**20**	exp skin disease/
**21**	health status disparities/	**21**	mental.ti.	**21**	exp lung disease/
**22**	exp disease/	**22**	malaria.ti.	**22**	diabetes mellitus/
**23**	exp virus diseases/	**23**	dengue.ti.	**23**	Disease association/
**24**	exp viruses/ and human*.ab.	**24**	respiratory.ti.	**24**	Western blotting/
**25**	exp Communicable Diseases/	**25**	infection*.ti.	**25**	etiology/
**26**	Infection/	**26**	wellbeing.ti.	**26**	immunology/
**27**	aedes/	**27**	well being.ti.	**27**	Infection/
**28**	water/ps	**28**	outbreak*.ti.	**28**	Death/
**29**	allergens/	**29**	zika.ti.	**29**	Cardiovascular disease/
**30**	exp Disease Outbreaks/	**30**	undernutrition.ti.	**30**	Fever/
**31**	exp Mortality/	**31**	influenza.ti.	**31**	health/
**32**	mo.fs.	**32**	hospitali?ation*.ti.	**32**	Mental disease/
**33**	exp Malaria/	**33**	epidemic.ti.	**33**	Epidemiology/
**34**	exp disease transmission, infectious/	**34**	ecohealth.ti.	**34**	Cerebrovascular accident/
**35**	exp Neoplasms/	**35**	ebola.ti.	**35**	hospital admission/
**36**	exp Heat Stress Disorders/	**36**	death.ti.	**36**	anemia/

**37**	exp Fever/	**37**	kills.ti.	**37**	Chronic disease/
**38**	exp Metabolic Diseases/	**38**	cholera.ti.	**38**	public health/
**39**	exp Death/	**39**	foodborne.ti.	**39**	cancer risk/
**40**	exp Skin/re	**40**	epidemics.ti.	**40**	Virus infection/
**41**	exp Environmental Illness/	**41**	endemic.ti.	**41**	kidney failure/
**42**	Community-Acquired Infections/	**42**	pandemic.ti.	**42**	Mental health/
**43**	exp Mental Disorders/	**43**	syndrome.ti.	**43**	Neurologic disease/
**44**	Environmental Exposure/ae	**44**	asthma.ti.	**44**	Health status/
**45**	nutrition disorders/	**45**	illness*.ti.	**45**	exp Birth weight/
**46**	child nutrition disorders/	**46**	morbidity.ti.	**46**	Human immunodeficiency virus/
**47**	exp Rickettsiaceae/	**47**	cancer.ti.	**47**	exp zoonosis/
**48**	exp infant nutrition disorders/	**48**	malnutrition.ti.	**48**	prophylaxis/
**49**	exp malnutrition/	**49**	mental health.ti.	**49**	Disease transmission/
**50**	exp wasting syndrome/	**50**	mental disorder*.ti.	**50**	Gastrointestinal disease/
**51**	exp encephalitis/	**51**	(global adj2 nutrition*).ti.	**51**	Infection risk/
**52**	salmonella infections/	**52**	(population adj2 nutrition*).ti.	**52**	Mental stress/
**53**	Helminthiasis/	**53**	(security adj2 nutrition*).ti.	**53**	antivirus agent/
**54**	food contamination/	**54**	(insecurity adj2 nutrition*).ti.	**54**	exp allergen/
**55**	zoonoses/	**55**	(global adj2 food adj2 (supply or production)).ti.	**55**	Childhood disease/
**56**	Noncommunicable Diseases/	**56**	(security adj2 food).ti.	**56**	immunogenicity/
**57**	health.ti.	**57**	(insecurity adj2 food).ti.	**57**	malnutrition/
**58**	disease*.ti.	**58**	lyme disease.ti.	**58**	Pregnancy outcome/
**59**	infectious.ti.	**59**	Chikungunya.ti.	**59**	exp *malaria/
**60**	mortality.ti.	**60**	Hantavirus.ti.	**60**	Health hazard/
**61**	healthy.ti.	**61**	West Nile disease.ti.	**61**	Life expectancy/
**62**	mental.ti.	**62**	west nile fever.ti.	**62**	Child development/
**63**	mental.ti.	**63**	global disease*.ab.	**63**	dermatology/
**64**	malaria.ti.	**64**	global health.ab.	**64**	hygiene/
**65**	malaria.ti.	**65**	well being.ab.	**65**	virus detection/
**66**	dengue.ti.	**66**	wellbeing.ab.	**66**	genotoxicity/
**67**	respiratory.ti.	**67**	human health.ab.	**67**	Allergic rhinitis/
**68**	infection*.ti.	**68**	vector borne disease*.ab.	**68**	women's health/

**69**	wellbeing.ti.	**69**	health implication*.ab.	**69**	exp leishmania/
**70**	well being.ti.	**70**	public health.ab.	**70**	encephalitis/
**71**	outbreak*.ti.	**71**	health consequence*.ab.	**71**	Child health/
**72**	zika.ti.	**72**	mental health.ab.	**72**	Communicable disease/
**73**	undernutrition.ti.	**73**	reproductive health.ab.	**73**	virus vector/
**74**	influenza.ti.	**74**	health adaptation.ab.	**74**	infant mortality/
**75**	hospitali?ation.ti.	**75**	(mortality adj2 morbidity).ab.	**75**	Health disparity/
**76**	epidemic.ti.	**76**	infectious disease*.ab.	**76**	Psychological well being/
**77**	ecohealth.ti.	**77**	health outcomes.ab.	**77**	Reproductive health/
**78**	ebola.ti.	**78**	health vulnerability.ab.	**78**	Tropical medicine/
**79**	death.ti.	**79**	(health adj2 impact*).ab.	**79**	Vulnerable population/
**80**	kills.ti.	**80**	(health adj2 threat*).ab.	**80**	Allergic disease/
**81**	cholera.ti.	**81**	(burden adj2 disease*).ab.	**81**	Maternal welfare/
**82**	foodborne.ti.	**82**	(population adj2 health).ab.	**82**	Toxoplasma gondii/
**83**	epidemics.ti.	**83**	(health adj2 effect*).ab.	**83**	Disease burden/
**84**	endemic.ti.	**84**	(health adj2 risk*).ab.	**84**	Childhood mortality/
**85**	pandemic.ti.	**85**	(health adj2 benefit*).ab.	**85**	Dengue virus/
**86**	syndrome.ti.	**86**	(health adj2 co-benefit*).ab.	**86**	Infectious agent/
**87**	asthma.ti.	**87**	mental disorder*.ab.	**87**	respiratory tract allergy/
**88**	illness*.ti.	**88**	Noncommunicable Disease*.ab.	**88**	enterovirus/
**89**	morbidity.ti.	**89**	malaria.ab.	**89**	anopheles/
**90**	cancer.ti.	**90**	syndrome.ab.	**90**	pollen allergy/
**91**	malnutrition.ti.	**91**	(tree or trees or soil).ti.	**91**	campylobacter/
**92**	mental health*.ti.	**92**	(people or human* or public health or men or women or children or patients or students).af.	**92**	exp Heat injury/
**93**	(global adj2 nutrition*).ti.	**93**	(editorial or letter or comment).pt.	**93**	Global health/
**94**	(population adj2 nutrition*).ti.	**94**	or/1-15	**94**	Non communicable disease/
**95**	(security adj2 nutrition*).ti.	**95**	or/16-90	**95**	norovirus/
**96**	(insecurity adj2 nutrition*).ti.	**96**	94 and 95	**96**	Ebola hemorrhagic/
**97**	(global adj2 food adj2 (supply or production)).ti.	**97**	96 not 91	**97**	Health impact assessment/
**98**	(security adj2 food).ti.	**98**	97 and 92	**98**	Yellow fever/
**99**	(insecurity adj2 food).ti.	**99**	limit 98 to yr="2007 - 2019"	**99**	leptospira/
**100**	Chikungunya.ti.	**100**	limit 99 to abstracts	**100**	chikungunya/

**101**	Hantavirus.ti.	**101**	100 not 93	**101**	Arbovirus/
**102**	West Nile virus.ti.			**102**	tick-borne disease/
**103**	west nile fever.ti.			**103**	Food insecurity/
**104**	global disease*.ab.			**104**	Premature mortality/
**105**	global health.ab.			**105**	Trihalomethanes/
**106**	well being.ab.			**106**	population health/
**107**	wellbeing.ab.			**107**	Japanese encephalitis/
**108**	human health.ab.			**108**	Crimean-Congo hemorrhagic fever/
**109**	vector borne disease*.ab.			**109**	urban health/
**110**	health implication*.ab.			**110**	disease*.ti.
**111**	public health.ab.			**111**	cancer.ti.
**112**	health consequence*.ab.			**112**	health.ti.
**113**	mental health.ab.			**113**	infection*.ti.
**114**	reproductive health.ab.			**114**	mortality.ti.
**115**	health adaptation.ab.			**115**	respiratory.ti.
**116**	(mortality adj2 morbidity).ab.			**116**	death.ti.
**117**	infectious disease*.ab.			**117**	healthy.ti.
**118**	syndrome.ab.			**118**	mental.ti.
**119**	health outcomes.ab.			**119**	asthma.ti.
**120**	health vulnerability.ab.			**120**	influenza.ti.
**121**	(health adj2 impact*).ab.			**121**	illness*.ti.
**122**	(health adj2 threat*).ab.			**122**	malaria.ti.
**123**	(burden adj2 disease*).ab.			**123**	infectious.ti.
**124**	(population adj2 health).ab.			**124**	outbreak*.ti.
**125**	(health adj2 effect*).ab.			**125**	hospitali?ation*.ti.
**126**	(health adj2 risk*).ab.			**126**	epidemic.ti.

**127**	(health adj2 benefit).ab.			**127**	dengue.ti.
**128**	(health adj2 co-benefit*).ab.			**128**	endemic.ti.
**129**	mental disorder*.ab.			**129**	well being.ti.
**130**	Noncommunicable Disease*.ab.			**130**	pandemic.ti.
**131**	malaria.ab.			**131**	cholera.ti.
**132**	mycotoxins/ not food contamination/			**132**	ebola.ti.
**133**	respiratory tract diseases/			**133**	zika.ti.
**134**	Aspergillus/			**134**	west nile virus.ti.
**135**	Candida/			**135**	epidemics.ti.
**136**	exp candida/			**136**	wellbeing.ti.
**137**	exp aspergillus/			**137**	Hantavirus.ti.
**138**	Disease Susceptibility/			**138**	(insecurity adj2 food).ti.
**139**	encephalitis/			**139**	kills.ti.
**140**	HIV infections/			**140**	(global adj2 food adj2 (supply or production)).ti.
**141**	bacterial infection/			**141**	flavivirus.ti.
**142**	or/1-17			**142**	(global adj2 nutrition*).ti.
**143**	or/18-131			**143**	(security adj2 nutrition*).ti.
**144**	or/18-141			**144**	ecohealth.ti.
**145**	(tree or trees).ti.			**145**	(security adj2 food).ti.
**146**	soil.ti.			**146**	(mortality adj2 morbidity).ab.
**147**	exp animals/ not humans.sh.			**147**	public health.ab.
**148**	142 and 143			**148**	mental health.ab.
**149**	142 and 144			**149**	infectious disease*.ab.
**150**	148 not 145			**150**	well being.ab.
**151**	150 not 146			**151**	malaria.ab.
**152**	151 not 147			**152**	health outcomes.ab.
**153**	149 not 145			**153**	(health adj2 effect*).ab.
**154**	153 not 146			**154**	human health.ab.
**155**	154 not 147			**155**	mental disorder*.ab.
**156**	155 NOT 152			**156**	(burden adj2 disease*).ab.
**157**	limit 152 to yr="2007 - Current"			**157**	(health adj2 impact*).ab.

**158**	limit 155 to yr="2007 - Current"			**158**	wellbeing.ab.
**159**	(editorial or letter or comment).pt.			**159**	global health.ab.
**160**	157 not 159			**160**	gastroenteritis.ab.
**161**	158 not 159			**161**	(population adj2 health).ab.
				**162**	reproductive health.ab.
				**163**	(health adj2 threat*).ab.
				**164**	health consequence*.ab.
				**165**	health implication*.ab.
				**166**	flavivirus.ab.
				**167**	aeroallergens.ab.
				**168**	vector borne disease*.ab.
				**169**	(health adj2 co-benefit*).ab.
				**170**	health adaptation.ab.
				**171**	or/1-17
				**172**	or/18-170
				**173**	(tree or trees).ti.
				**174**	soil.ti.
				**175**	(exp animal/ or nonhuman/) not exp human/
				**176**	or/172-174
				**177**	171 and 172
				**178**	177 not 176
				**179**	limit 178 to yr="2007 -2019"
				**180**	limit 179 to abstracts

Data

The bibliometric search worked with specific inclusion and exclusion criteria that were applied to capture only the most relevant literature. This includes peer-reviewed scientific articles on health and climate change in English, with no direct restriction to country or population applied. All peer-reviewed articles reporting the findings of original qualitative and quantitative studies will be included, together with reviews, editorials, viewpoints, letters or comments; those in the latter category (reviews, editorials, viewpoints, letters, comments) will be filtered for analysis. This practice – of including reviews, editorials, viewpoints and comments – was followed in the 2017 and 2018 Lancet Countdown reports and provides an indication of scientific engagement outside of peer review (in analyses presented in these earlier Lancet Countdown reports, it was noted that apparent increases in engagement can reflect increases in comments and editorials rather than in original science).

Caveats

The methodology provided here enables a quantitative appraisal of the research question. The quality of the data and the specifics of its content are not assessed by the indicator team. However, with the outputs all published in peer-reviewed journals, there is a de facto check on quality. For this reason, the indicator does not cover grey literature.

Future form of the indicator

There is scope to formulate add-ons to the indicator, for example focusing on trends in scientific coverage of particular climate-sensitive health outcomes and/or regions.

A sensitivity analysis will be used for the 2021 analysis to determine appropriate classification criteria when coding the final data. The search terms will also be reviewed following the report, with attention given to whether the data can be further classified into general scientific topics.

There is also potential to think in more depth about scientific literature in other languages.

Additional analysis

Proportion of coverage in relation to Embase total publications

Set against a backdrop of annually increasing publications in both databases, the shift in scientific engagement, more generally, can be approximated by the number of articles in a scientific database as a whole compared to those for climate change and health and climate change. Figure 65 demonstrates that both health and climate change and climate change are increasing in proportion of scientific interest, though climate change has a steeper curve and therefore a greater rate of increase. After a drop in proportion in 2008, it is not until 2011 that climate change begins to increase again. Despite one year of decrease in proportion, in 2017, this picks up again in 2018. 2019 sees the highest proportion for both climate change (0.61%) and health and climate change (0.052%).

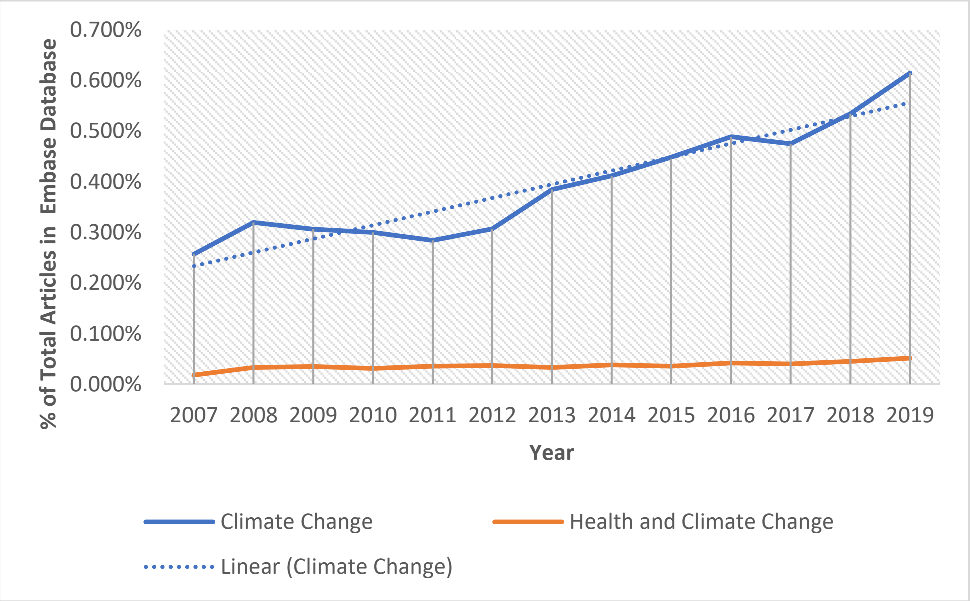

Figure 65: Proportion of climate change and health and climate change articles in relation to the entire number of scientific articles in the Embase database.

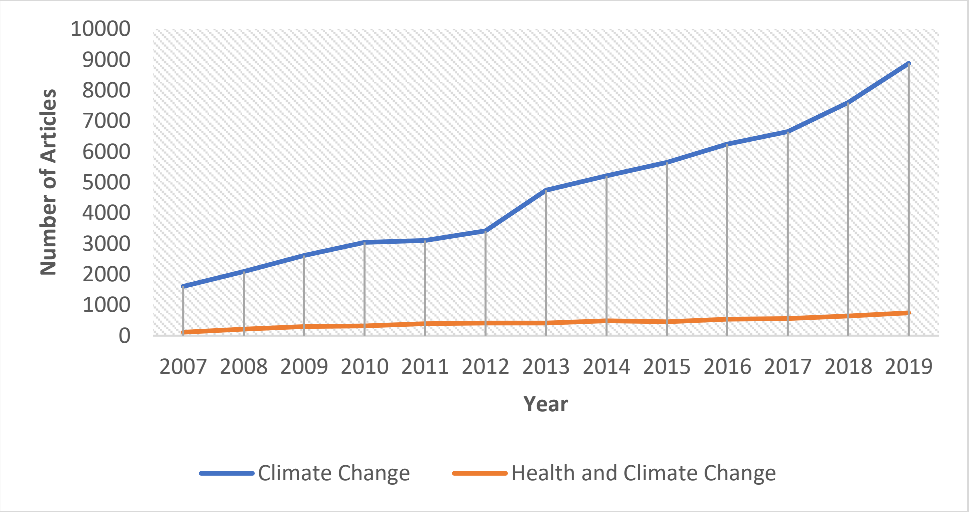

Figure 66: Absolute numbers of scientific papers on climate change and health and climate change, 2007-2019.

Figure 67 shows the countries contributing most (≥20 publications) to scientific engagement between 2007 and 2019. The lower-limit of 20 in this graph demonstrates the concentration of scientific engagement in particular countries.

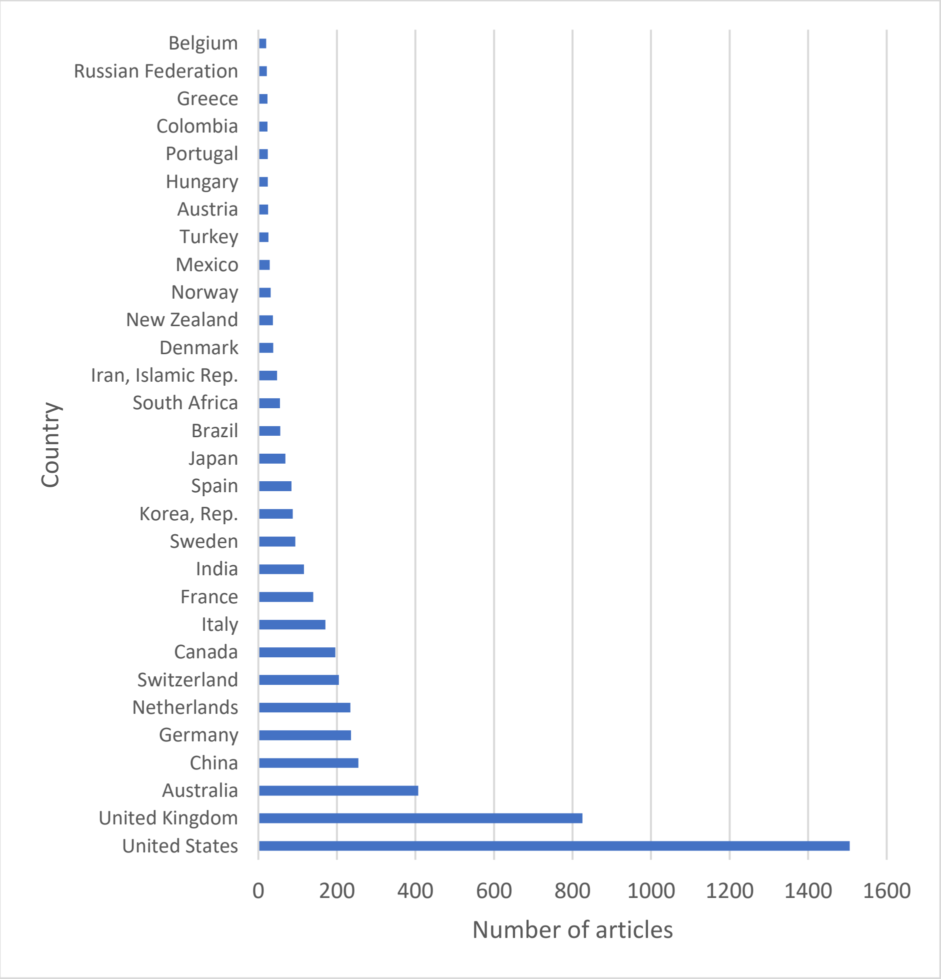

Figure 67: Countries with ≥20 articles on health and climate change in the period between 2007 and 2019.

Geographical Coverage

Arranged into WHO regions (Figure 68), the majority of publications come from the European Region, the Western Pacific, and the Region of the Americas. Increased engagement in 2019 can be seen across the European Region (+24%), The Americas (+26%), and the South East Asian Region (+41%), while decreases are observed in the African Region (-31%), the Eastern Mediterranean (-38%), and the Western Pacific (-5%).

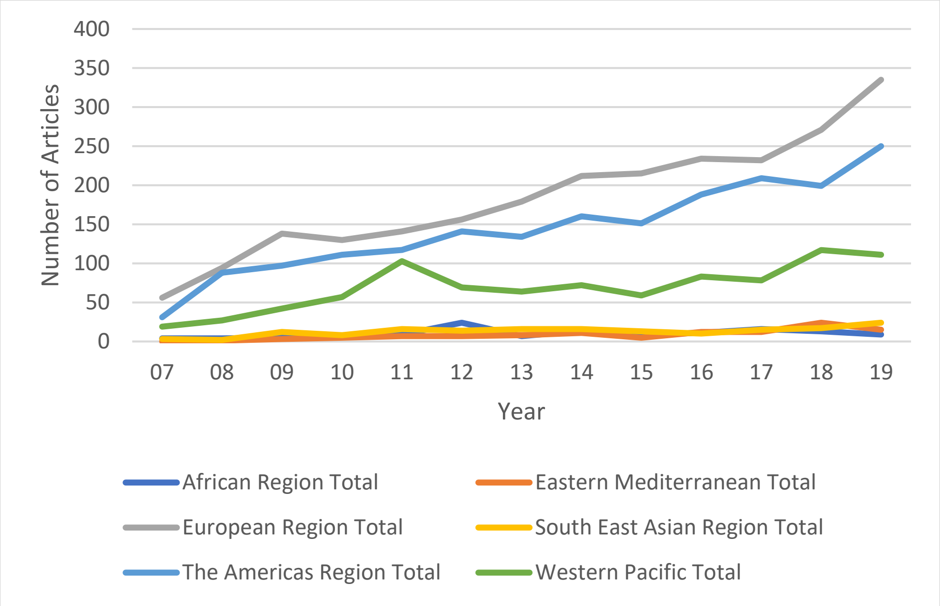

Figure 68: Total number of scientific publications on health and climate change between 2007 and 2019 by WHO Region.

Indicator 5.4: Government Engagement in Health and Climate Change

Indicator 5.4.1: Engagement in Health and Climate Change in the United Nations General Assembly

Methods

In order to produce the measure of high-level political engagement with climate change and health in the UN General Assembly, a new dataset of UN General Debate statements is used, which is discussed below. This approach to using UNGD statements to produce the indicators is based on the application of natural language processing to the corpus of UNGD statements. References to key search terms linked to (a) health, and (b) climate change are identified:

Health terms	Climate change terms
• **malaria**<br>• **diarrhoea**<br>• **infection**<br>• **disease**<br>• **diseases**<br>• **sars**<br>• **measles**<br>• **pneumonia**<br>• **epidemic**<br>• **epidemics**<br>• **pandemic**<br>• **pandemics**<br>• **epidemiology**<br>• **healthcare**<br>• **health**<br>• **mortality**<br>• **morbidity**<br>• **nutrition**<br>• **illness**<br>• **illnesses**<br>• **ncd**<br>• **ncds**<br>• **air pollution**<br>• **nutrition**<br>• **malnutrition**<br>• **malnourishment**<br>• **mental disorder**<br>• **mental disorders**<br>• **stunting**	• climate change<br>• changing climate<br>• climate emergency<br>• climate action<br>• climate crisis<br>• climate decay<br>• global warming<br>• green house<br>• temperature<br>• extreme weather<br>• global environmental change<br>• climate variability<br>• greenhouse<br>• greenhouse-gas<br>• low carbon<br>• ghge<br>• ghges<br>• renewable energy<br>• carbon emission<br>• carbon emissions<br>• carbon dioxide<br>• carbon-dioxide<br>• co2 emission<br>• co2 emissions<br>• climate pollutant<br>• climate pollutants<br>• decarbonization<br>• decarbonisation<br>• carbon neutral<br>• carbon-neutral<br>• carbon neutrality<br>• climate neutrality<br>• net-zero<br>• net zero

These key terms have been updated from previous years to reflect the changing terminology used to discuss climate change. In order to produce an indicator of engagement with the intersection of climate change and health, an examination was undertaken to determine whether any of the climate change related terms appeared immediately before or after any health terms in the GD statements. This was based on a search of the 25 words before and after a reference to a health-related term. The choice of 25-word window context corresponds to approximately half a paragraph of text. Given that UNGD statements are highly structured and methodically

developed by governments over prolonged periods of time, it is assumed that half a paragraph of text around public health terms captures a sufficiently narrow context. The number of climate change term references in these contexts are then identified and counted to produce the measure of engagement with the link between health and climate change. A robustness analysis was conducted by varying the size of the context (5, 10, and 50 words). This substantively produced the same trends over time. A sample of the references produced by the search was also further examined as an additional check to ensure that the references identified reflect engagement with the health impacts of climate change.

Data

To produce this indicator, a new and updated dataset of GD statements was drawn upon: *the United Nations General Debate corpus*, in which the annual GD statements have been pre-processed and prepared for the application of natural language processing to the official English versions of the statements.[237] The dataset contains all of the country speeches made in the UN General Debate between 1970 and 2019. Table 29 presents summary of the data by year:

Table 29: Summary information for UN General Debate Corpus.

Year	General Debate statements	Total sentences	Total words
1970	70	11841	304174
1971	116	19892	508523
1972	125	21208	541018
1973	120	21452	536411
1974	129	22051	568613
1975	126	21379	534339
1976	134	23827	599970
1977	140	24822	605742
1978	141	25267	625320
1979	144	26501	651959
1980	149	27223	657546
1981	145	26097	633635
1982	147	23438	638098
1983	149	26780	640595
1984	150	27982	660387
1985	137	19265	592655
1986	149	19041	577509
1987	152	18346	563016
1988	154	18604	569472
1989	153	19444	574342
1990	156	17893	522192
1991	162	18553	538349
1992	167	18594	543126
1993	175	20165	587437
1994	178	19946	580525
1995	172	17872	536740
1996	181	18058	522695

1997	176	17709	514492
1998	181	18888	514854
1999	181	18541	531291
2000	178	16262	464312
2001	189	14753	414681
2002	188	13985	380481
2003	189	14737	399396
2004	192	14904	405290
2005	185	13016	353065
2006	193	14647	390476
2007	191	14585	387883
2008	192	14298	384880
2009	193	16038	423395
2010	189	14438	391946
2011	194	16295	429974
2012	195	16842	444517
2013	193	16398	440893
2014	194	15865	421945
2015	193	16134	436361
2016	194	16001	420148
2017	196	16814	439621
2018	196	16987	455195
2019	195	17537	466108
Total	8288	941215	25325592

The data was pre-processed for analysis by removing punctuation, symbols, numbers, stopwords, and URLs. In addition, all tokens were normalised (lowercased). All pre-processing and analysis was carried out in R using the "quanteda" package.[238]

Caveats

The search for climate change terms in the context of public health references is a proxy for the semantic linkage between the two sets of terms in GD statements. This approach produces a scalable and reproducible measure with a high degree of reliability that does not involve human judgement or subjective biases. However, there may be examples of governments referring to climate change and health but not the direct linkages between the two, which are included in the count; and there may be examples of governments discussing the health impacts of climate change in their UNGD statements, which are not included because the distance between the mention of the climate change term and the health term exceeds 25 words. Based on an analysis of a sample of the speeches and references, such cases are relatively rare and do not have a significant bearing on the indicator or the trends uncovered.

It is also worth noting that the analysis here is based on a narrow range of search terms, which excludes reference to many of indirect links between climate change and health. A number of GD statements in this time period refer to such indirect connections, such as the effects of climate change on water and agriculture – however, these are not included here. Therefore, the results present a somewhat conservative estimate of high-level political engagement with the intersection of climate change and health. Future work in this area will consider engagement with these indirect links.

Future form of the indicator

In the future, a closer examination of references to indirect links between climate change and health is planned. For example, what are the main ways in which governments view climate change impacting on health? Consideration will be given as to whether this changes over time based on awareness of the multiple ways in which climate change and health are connected. Some of the references to the indirect links between climate change and health made in UNGD statements have been highlighted in the main report.

Additional analysis

Figure 69 presents the total proportion of countries referring to climate change, health, and the intersation between the two at the UN General debate and additional findings and breakdowns are also presented here. Figure 70 below presents the proportion of countries that engage with the intersection of climate change and health by WHO region. It is worth noting that the relatively higher level of political engagement by countries in the Western Pacific is especially driven by the Small Island Developing States (SIDS) in this region. It also worth noting that North America WHO region contains only two countries, USA and Canada. As Canada made a reference to the health impacts of climate change (the US statement made no reference to climate change), the North America region has 50 per cent of countries engaged with the climate change-health links.

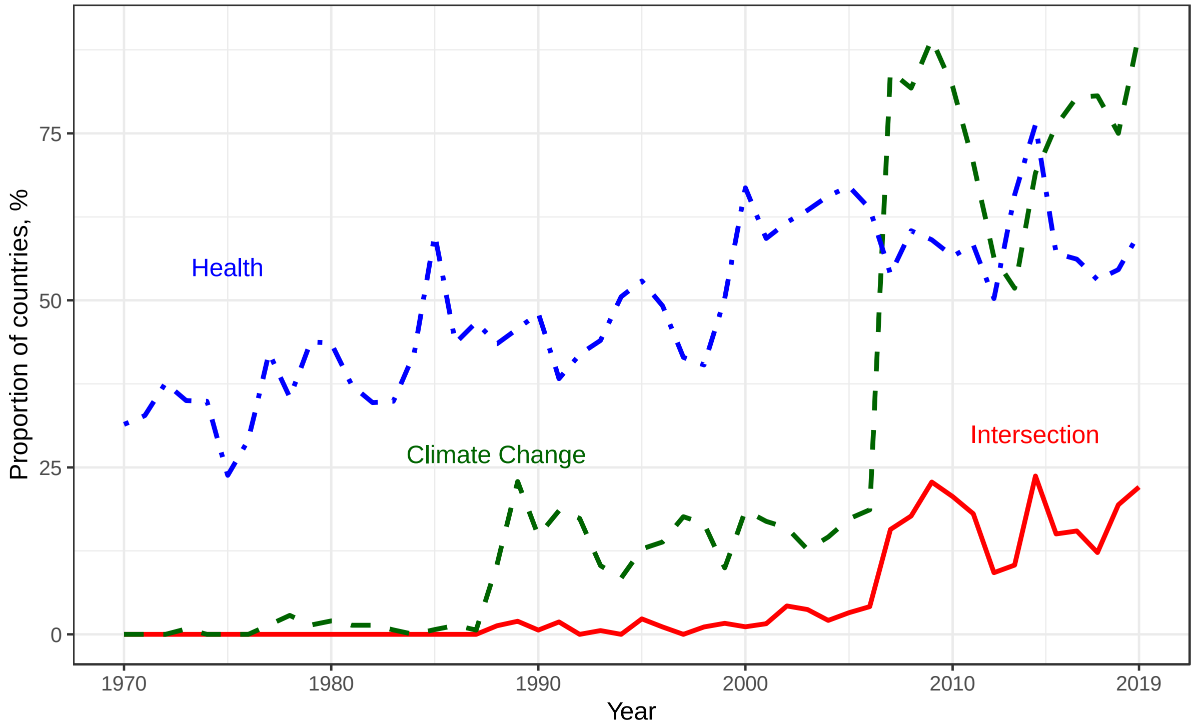

Figure 69: Proportion of countries referring to climate change, health, and the intersection between the two, UN General Debate, 1970-2019.

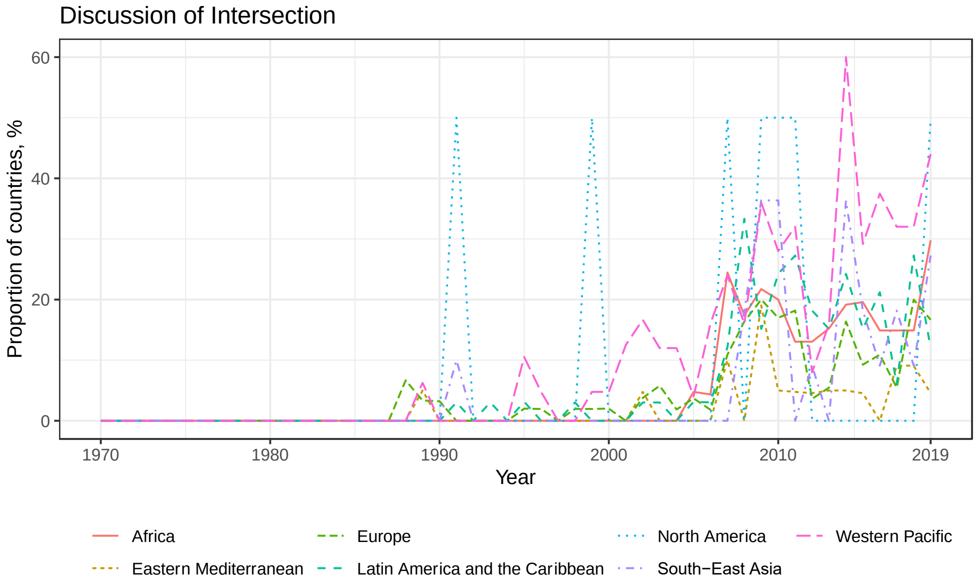

Figure 70: Proportion of countries referring to intersection of health and climate change by region, 1970-2019.

Figure 71 below presents the total number of references to the health impacts of climate change in GD statements between 1970 and 2019. The Figure demonstrates a very similar trend to when the proportion of countries is considered; with both approaches spikes are observed in 2009-10, 2014, and 2019. Figure 71 shows that the total references to climate change was higher in 2019 than in any previous year.

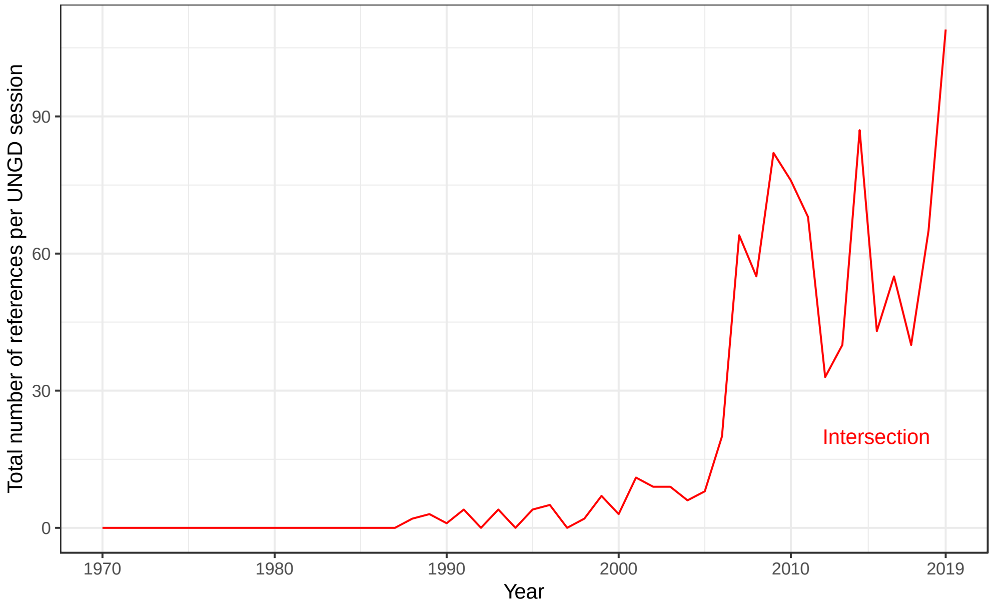

Figure 71: Total number of references to intersection, 1970-2019.

Figure 72, below, presents the total number of references to the climate change-health link between 1970 and 2019 by WHO region. The Figure shows that the most references tend to be made by countries in the Western Pacific. Countries in Africa, Latin America and the Caribbean, and Europe are the most engaged after the Western Pacific countries. In general, the Figure suggests that there is lower engagement among countries in the Eastern Mediterranean, North America, and South-East Asia.

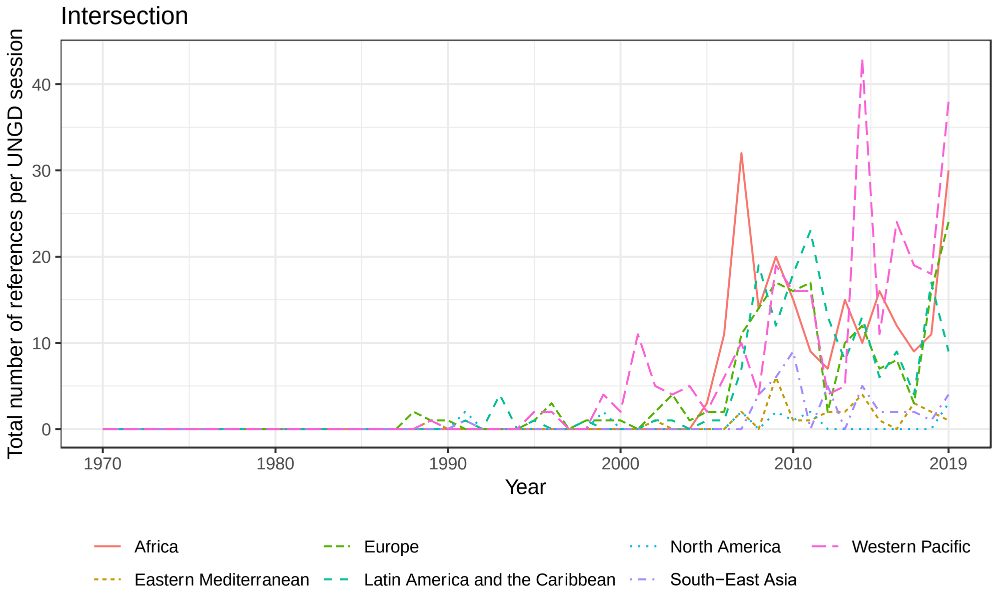

Figure 72: Total number of references to intersection by region, 1970-2019.

In addition to grouping countries by WHO region, different types of countries are also considered in terms of their potential importance and role in addressing issues related to climate change. This is provided in Figure 73. As noted in previous years' reports, the SIDS have driven much of the engagement with the health impacts of climate change, as well as climate change more generally, in the UN General Assembly. As such, a SIDS grouping is included. Arguably the three most important countries/unions in addressing climate change are USA, China, and the EU. This is both in terms of their carbon dioxide emissions and their power within the international system. This grouping is referred to as Tier 1 countries in Figure 73. Finally, an additional grouping of countries that are also important in terms of their CO2 emissions, their influence in international politics, and their potential impact on addressing climate change are also considered. This grouping, referred to as Tier 2 countries, includes: Poland, Australia, South Africa, Brazil, India, France, Germany, and Indonesia.

Figure 73 shows the proportion of countries that engage with the intersection of climate change and health based on these country groupings. Figure 74 shows the total number of references to the climate change-health intersection according to these groupings. Both figures demonstrate the higher level of engagement with the climate change-health linkages by SIDS than by Tier 1 or Tier 2 countries. However, it is worth noting that Figure 73 shows that a growing number of Tier 2 countries are engaging with the climate change-health intersection.

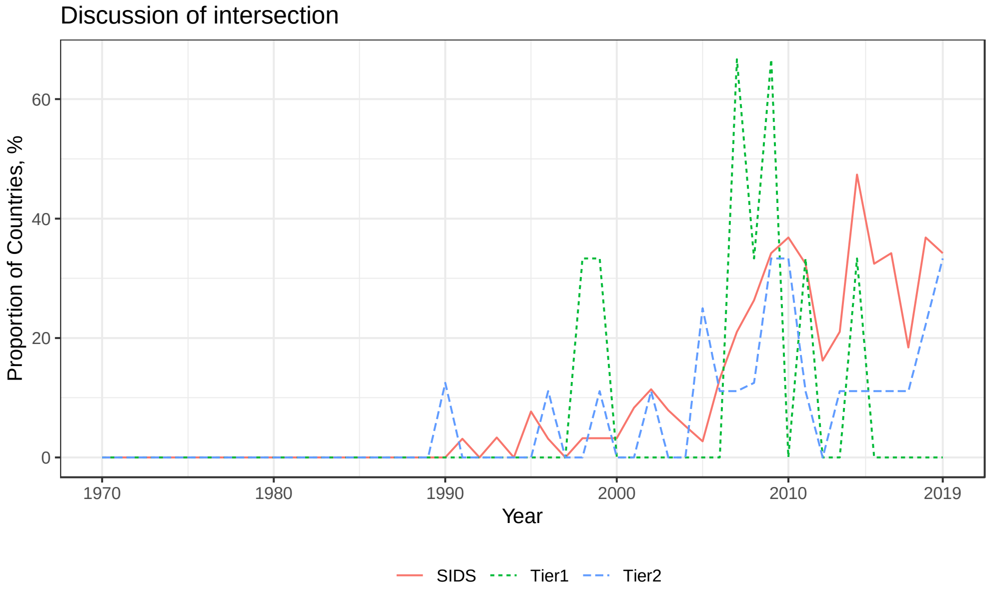

Figure 73: Proportion of countries referring to intersection of health and climate change by country grouping, 1970-2019.

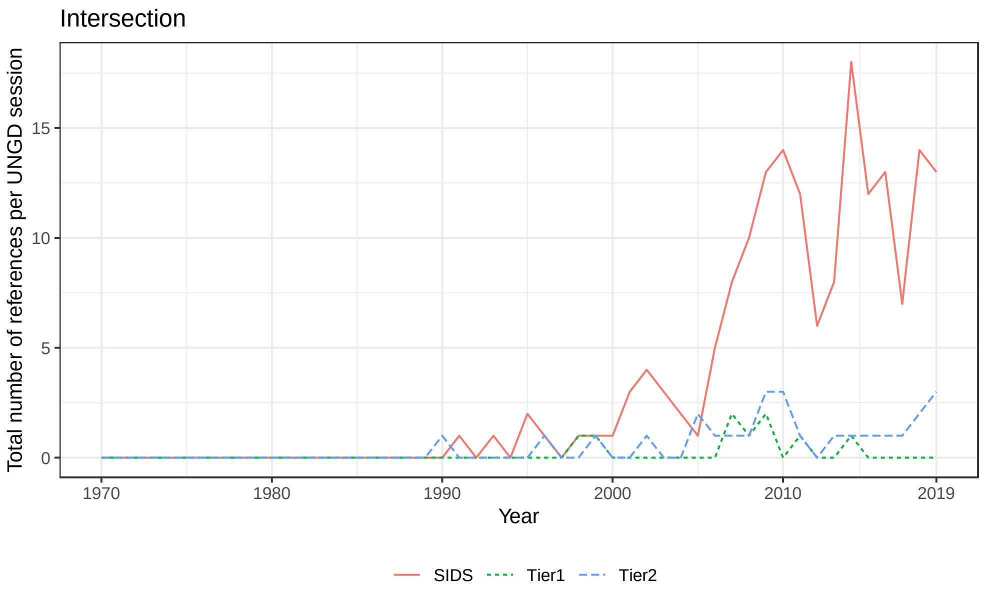

Figure 74: Total number of references to intersection by country grouping, 1970-2019.

Figure 75 below shows the level of political engagement with climate change and health separately, rather than engagement with the intersection of climate change and health. This is measured by the references to the key search terms associated with climate change and health in General Debate speeches. Figure 76 shows the proportion of countries that refer to public health in their GD statements between 1970 and 2019, while Figure 77 shows the proportion of countries that make a reference to climate change during this period. The figures show that in general there is higher levels of engagement with climate change than health. Figure 75 and Figure 77 also show a sharp increase in engagement with climate change in the General Debate around 2006, followed by a decline in 2009 after the COP 15 in Copenhagen that year. However, there has been an increase in engagement with climate change in recent years around the Paris Agreement with a huge increase in references to climate change in 2019. Engagement with health has in comparison been lower. However, there has broadly been increasing engagement with public health during this time period, and a sharp increase in 2000 with the launch of the Millennium Development Goals (MDGs). There is also an increase in the salience of global health

from 2012 onwards, which coincides with the transition from the MDGs to the Sustainable Development Goals (SDGs).

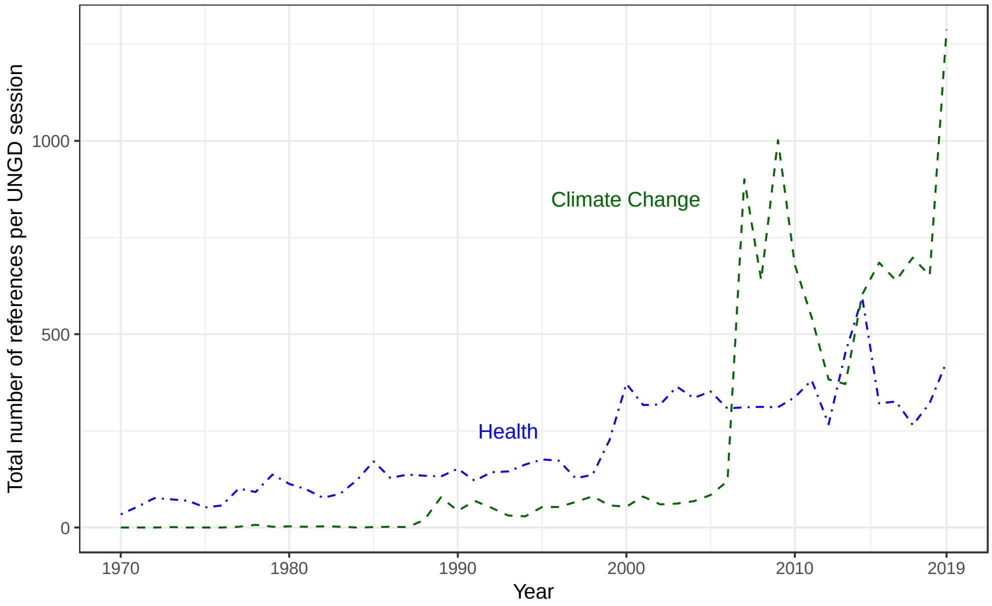

Figure 75: Total number of references to public health and climate change, 1970-2019.

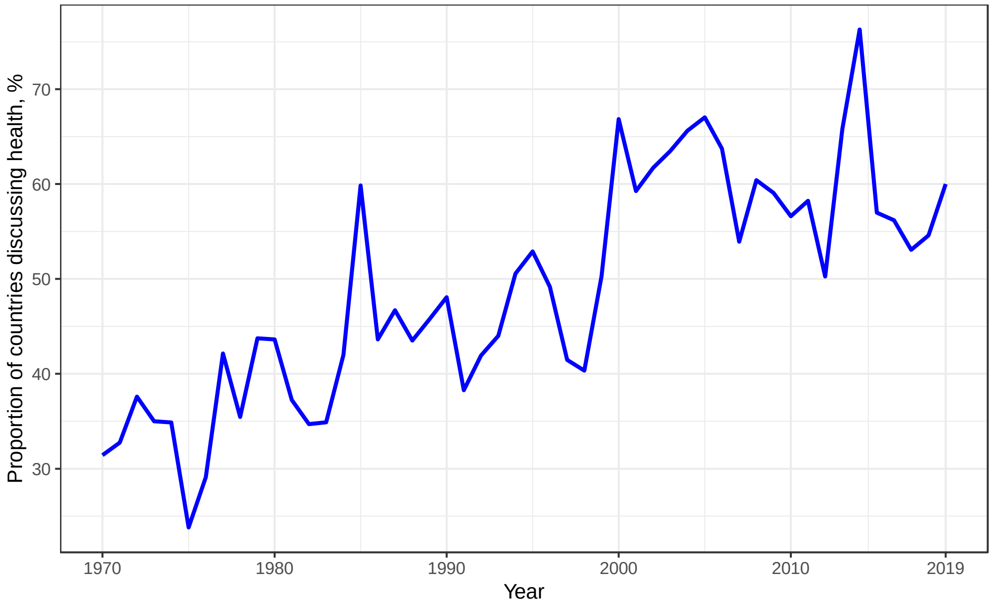

Figure 76: Proportion of countries referring to public health, 1970-2019.

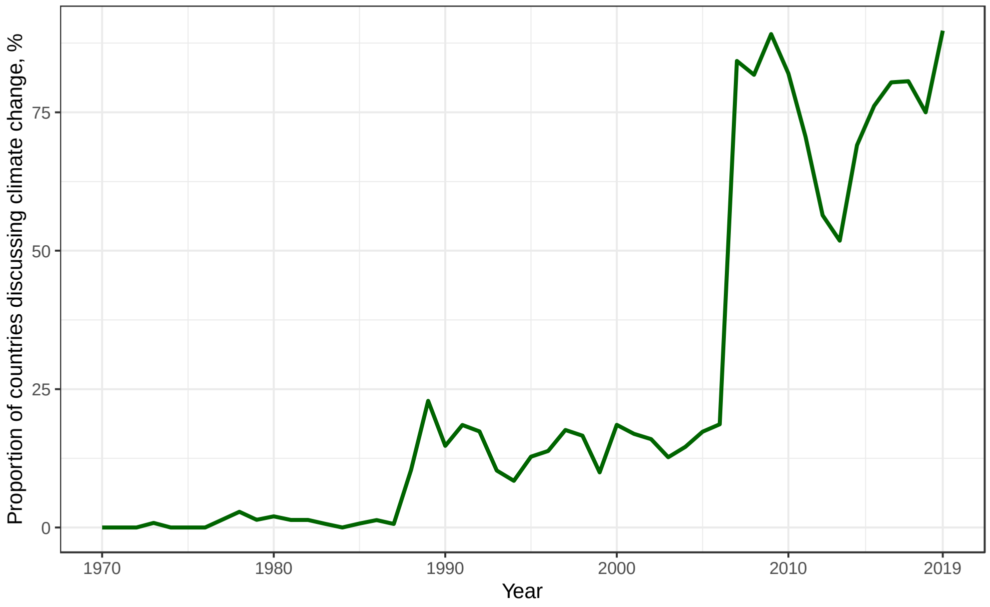

Figure 77: Proportion of countries referring to climate change, 1970-2019.

Figure 78 below presents a world map, which shows the countries that refer to the intersection of climate change and health in their 2019 UNGD statements, and the number of individual references they make. The map shows the relatively low level of engagement with the climate change-health relationship around the world in 2019. However, due to their size, the SIDS do not show up on the map. As noted previously, the SIDS tend to be highly represented among nations engaging with the health-climate change links.

Figure 79 and Figure 80 present world maps, which show the countries that refer to public health and climate change respectively in their 2019 UNGD statements, as well as indicating the number of references made by each country. The figures demonstrate that while there is relatively low engagement with the intersection of health and climate change, there is considerable engagement with the issues of climate change and health separately. Figure 79 and Figure 80 show that as well as a much larger share of countries around the world discussing climate change and health in their GD statements compared to those discussing the intersection, there is also much deeper engagement with these two areas individually, in that countries tend to make a number of references to climate change and health in their GD statements.

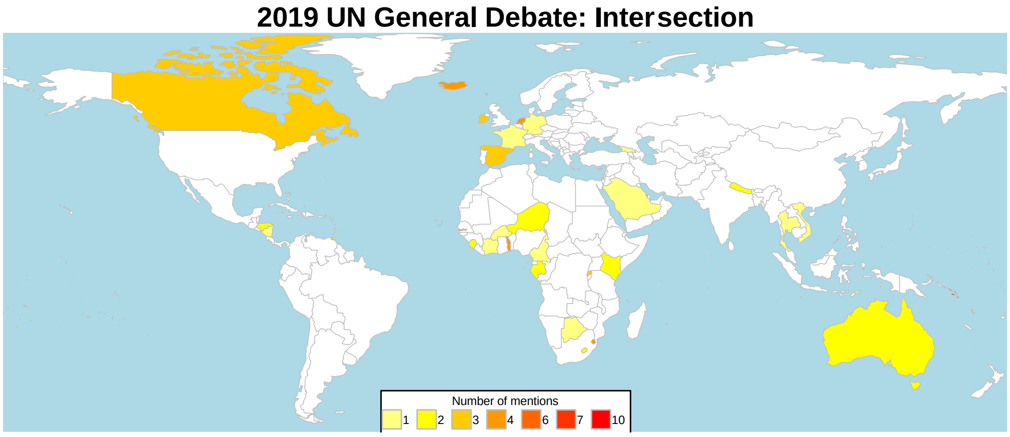

Figure 78: World map showing references to intersection of climate change and health, 2019.

2019 UN General Debate: Health

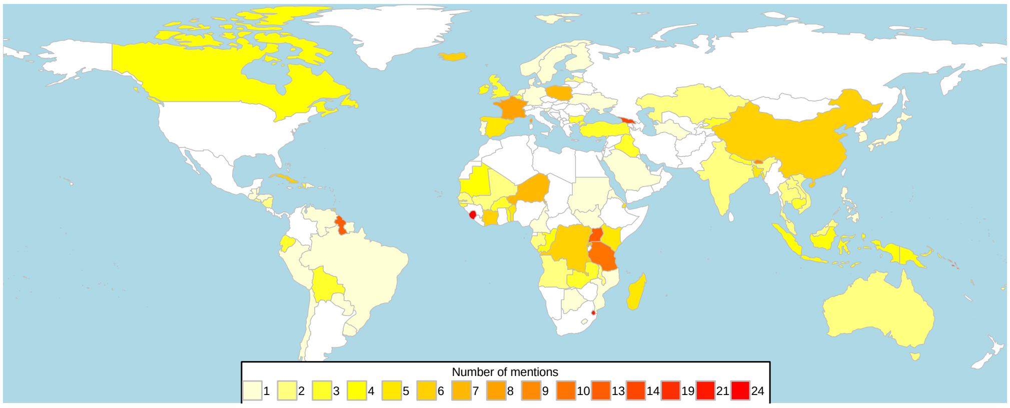

Figure 79: World map showing references to public health, 2019.

2019 UN General Debate: Climate Chang e

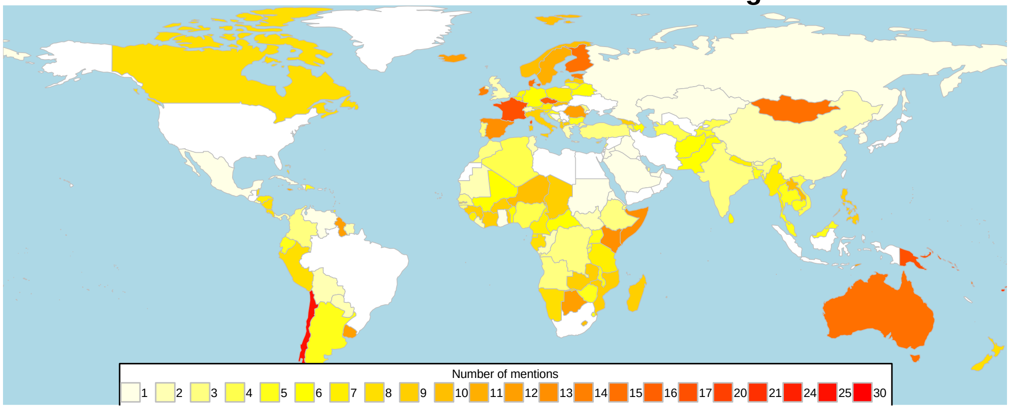

Figure 80: World map showing references to climate change, 2019.

The figures below show engagement with climate change, health, and the intersection of climate change and health over 1970-2019 for selected countries.

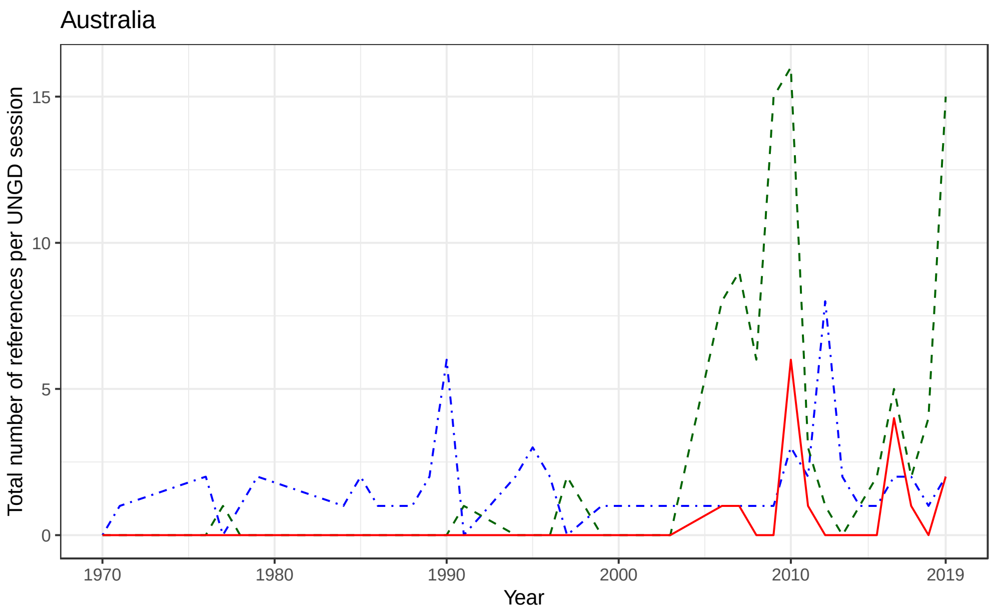

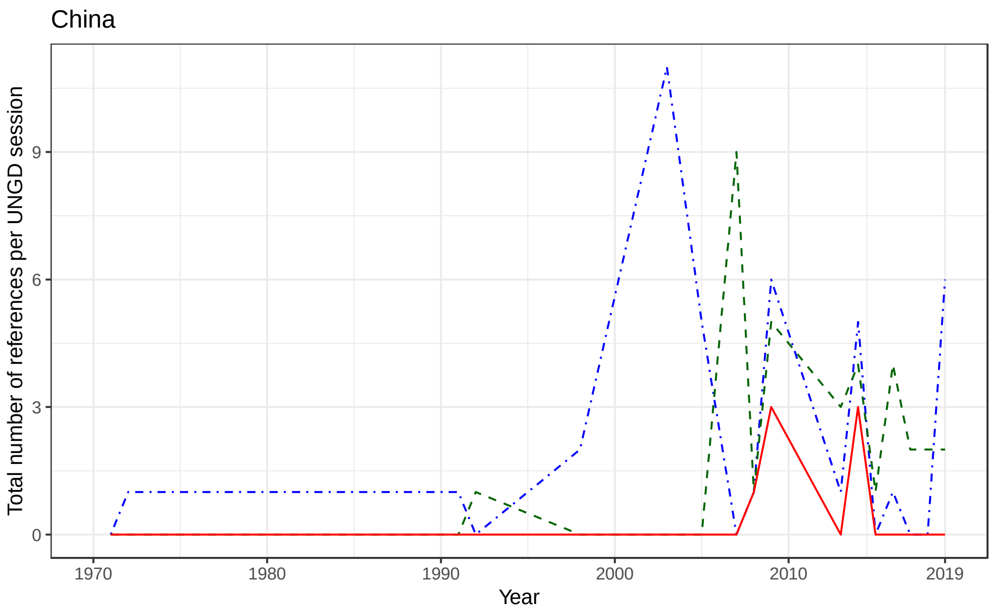

Germany

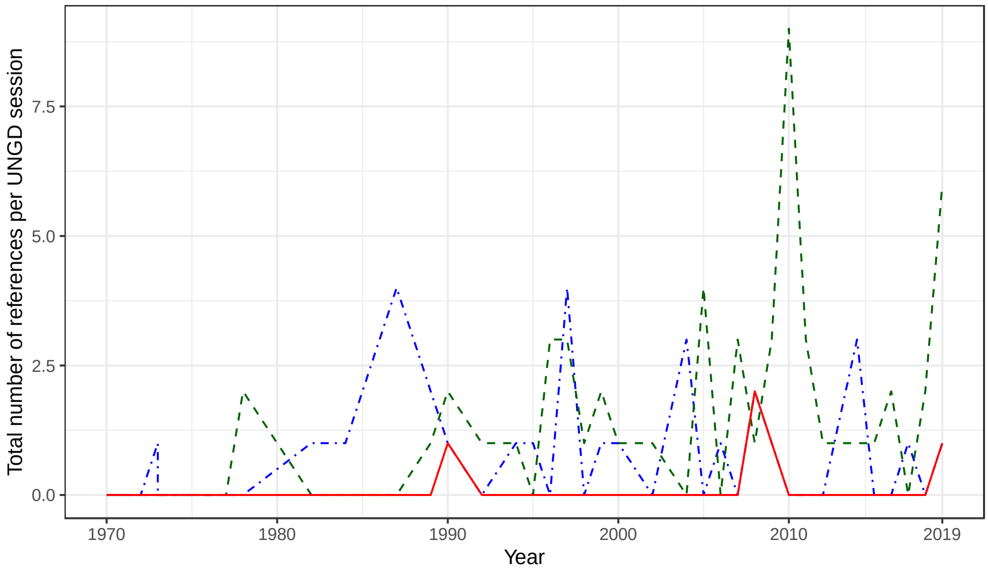

Total number of references per UNGD session
0.0
2.5
5.0
7.5
1970
1980
1990
2000
2010
2019
Year

EU

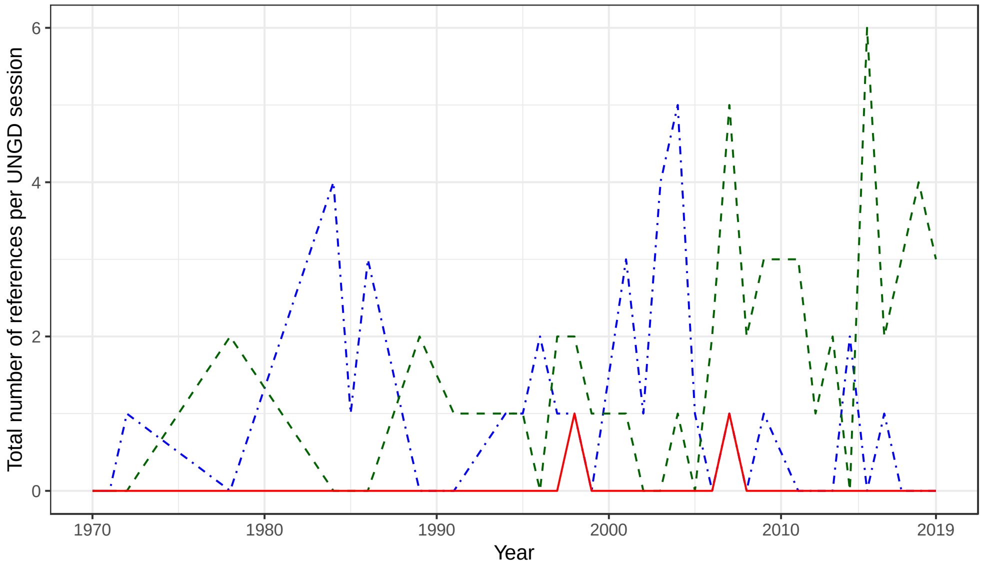

Total number of references per UNGD session
0
2
4
6
1970
1980
1990
2000
2010
2019
Year

France

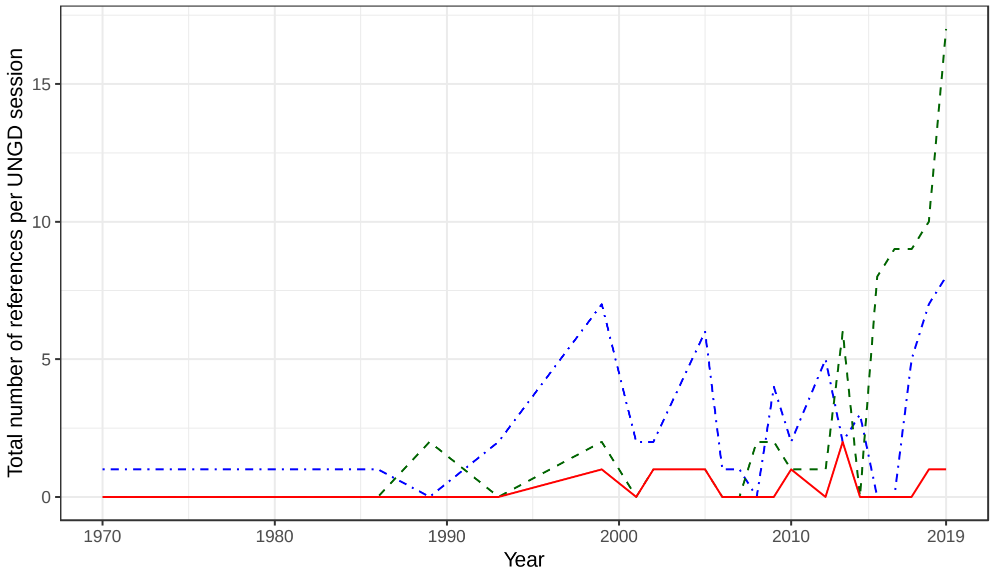
Total number of references per UNGD session
15
10
5
0
1970
1980
1990
2000
2010
2019
Year

UK

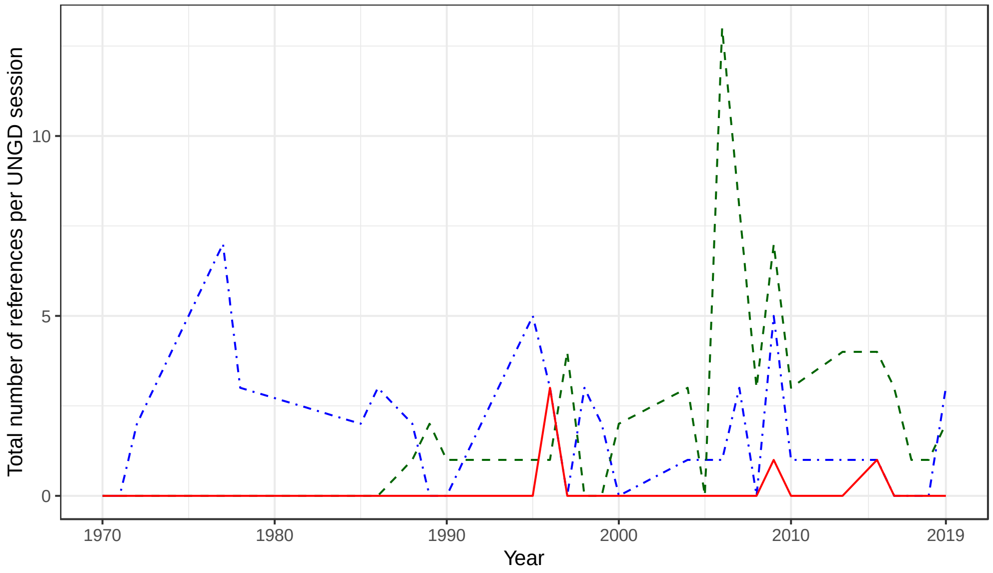
Total number of references per UNGD session
10
5
0
1970
1980
1990
2000
2010
2019
Year

India

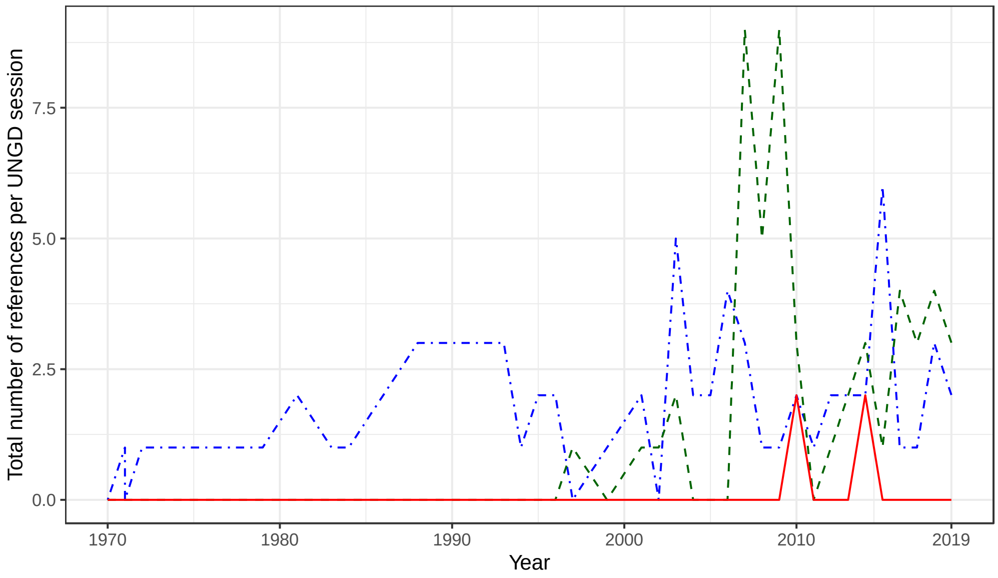

Total number of references per UNGD session
0.0
2.5
5.0
7.5
1970
1980
1990
2000
2010
2019
Year

USA

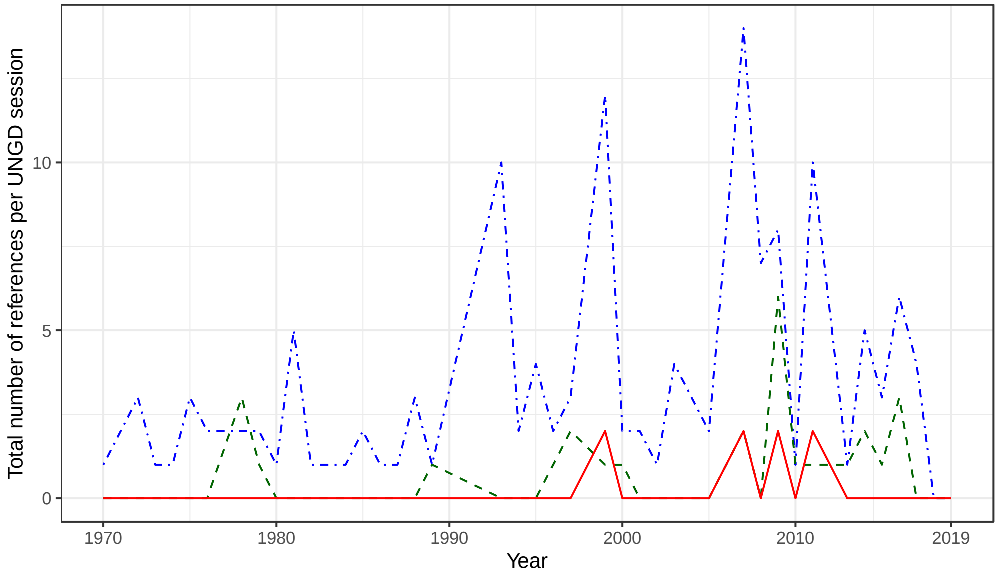

Total number of references per UNGD session
0
5
10
1970
1980
1990
2000
2010
2019
Year

Indicator 5.4.2: Engagement in Health and Climate Change in the Nationally Determined Contributions

Methods

Under the Paris Agreement, the Nationally Determined Contributions (NDCs) for each Party to the Agreement are communicated through the NDC registry. As a measure of engagement across climate change and health, and in particular of governments' appreciation of the health risks of climate change, all available first NDCs (as of March 31st 2020) were analysed with respect to their inclusion of both health-related terms and health-exposure terms. Though effective as a standalone analysis, this will also be compared in the 2021 Lancet Countdown report with the second iteration of NDCs to determine any change in governmental engagement with health in relation to climate change.

Each NDC file was uploaded to Nvivo. A number of NDCs were in either French or Spanish and were therefore translated using Google Translate. While each document covered broadly the same material (all had mitigation strategies, most had adaptation sections, coverage of national circumstances, and an account of fairness and ambition within the NDC), most were different in presentation, making extraction of text difficult in some instances. For example, some NDCs appeared to be screenshots of pages, which meant GoogleDocs was used for its Optical Character Recognition capacity, converting image into text.

The two categories used in analysis (health-related terms and exposure terms) were developed iteratively, with text extracted from the NDCs if it fell into one of the two categories. Health-related terms were those referring to health directly, denoting either its presence (e.g. health, well-being) or its absence (e.g. death, illness, disease). Any term mentioning 'health' (e.g. health centre, health surveillance) was also included, though false positives were excluded (e.g. ecosystem health, ocean health).

Exposure terms consisted of conditions, events or phenomena with an impact on health. For example, malnutrition is a health outcome and thus a health-related term, food security/insecurity is an exposure term. All *potential* exposure terms were included. Those related to other fields (such as agriculture, forestry or water resources, for example) and those not related to climate change (such as those within general accounts of national circumstances, which are often referred to earlier on in the NDC) were discounted later.

All terms were agreed upon by both researchers working on the indicator. Variations of all terms occurred within the NDC and an indicative list can be found below.

Relevant text was extracted and organised within a Microsoft Excel spreadsheet. Text was extracted in sentence form if containing a health-related terms and/or an exposure term. Sentences were understood as capital letter to full-stop, or semi-colon in a list or table. In this way, both sentences *and* the number of words within them could be counted (using Excel formulae) and included in the analysis. In order to ascertain the number of words and sentences within each NDC document, online freely available software was utilised (http://textfixer.com). This counts words pasted into a search box, including the number of sentences from full-stop to full-stop. The proportion of each document given to health-related terms and health-exposures (in words and in sentences) could therefore be measured.

With Excel organised in an appropriate way, extracted text was categorised. This categorisation process was both automatic (using Excel formulae) and manual. Though not all data extracted were used in the final analysis, extraction was based on:

- The number of words in the extracted text (divided by the total number of words in the text for a proportion)
- The page on which the text was located
- The section in which the text was located, if possible
- The percentage of the NDC taken up in text by the extract
- Whether or not the text pertained to health, and how many health terms used in that text
- Whether or not the text pertained to a health exposure, and how many health exposure terms used in that text
- Whether or not the text pertained to both health and a health exposure

- If an exposure term, the context in which this exposure was used (as 52% of potential exposure terms were used in a context not immediately related to health, such for their impact on agriculture or water resources)

The sum or average of these (depending upon relevance) was provided for each NDC, allowing analysis of the data as whole. Individual NDCs were also aggregated into regional data using World Health Organisation regions and World Bank Income and Lending Groups. Other geographical groupings, for comparative purposes, included Small Island Developing States (SIDS) and Least Developed Countries (LDCs).

Indicative list of key terms

Health-related terms	Exposure terms
Health	Flood
Illness	Drought
Disease	Pollution
Death	Water security
Loss of life	Food security
Malnutrition	Heat waves
Malaria	Cold waves
Dengue	Cyclones

Data

1. 158 first round NDCs were retrieved from the UNFCCC NDC Registry (interim),[239] representing 185 nations in total (the European Union NDC represents 28 nations)

NDCs retrieved from UNFCCC registry (n=185):

1 European Union First NDC (not included, Latvia's used instead.)

2 Afghanistan First NDC

3 Albania First NDC

4 Algeria First NDC

5 Andorra First NDC

6 Antigua and Barbuda First NDC

7 Argentina First NDC

8 Armenia First NDC

9 Australia First NDC

10 Azerbaijan First NDC

11 Bahamas First NDC

12 Bahrain First NDC

13 Bangladesh First NDC

14 Barbados First NDC

15 Belarus First NDC

16 Belize First NDC

17 Benin First NDC

18 Bhutan First NDC

19 Bolivia (Plurinational State of) First NDC

20 Bosnia and Herzegovina First NDC

21 Botswana First NDC

22 Brazil First NDC

23 Burkina Faso First NDC

24 Burundi First NDC

25 Cabo Verde First NDC

26 Cambodia First NDC

27 Cameroon First NDC

28 Canada First NDC

29 Central African Republic First NDC

30 Chad First NDC

31 Chile First NDC

32 China First NDC

33 Colombia First NDC

34 Comoros First NDC

35 Congo First NDC

36 Cook Islands First NDC

37 Costa Rica First NDC

38 Cuba First NDC

39 Côte d'Ivoire First NDC

40 Democratic People's Republic of Korea First NDC

41 Democratic Republic of the Congo First NDC

42 Djibouti First NDC

43 Dominica First NDC

44 Dominican Republic First NDC

45 Ecuador First NDC

46 Egypt First NDC

47 El Salvador First NDC

48 Equatorial Guinea First NDC

49 Eritrea First NDC

50 Eswatini First NDC

51 Ethiopia First NDC

52 Fiji First NDC

53 Gabon First NDC

54 Gambia First NDC

55 Georgia First NDC

56 Ghana First NDC

57 Grenada First NDC

58 Guatemala First NDC

59 Guinea First NDC

60 Guinea Bissau First NDC

61 Guyana First NDC

62 Haiti First NDC

63 Honduras First NDC

64 Iceland First NDC

65 India First NDC

66 Indonesia First NDC

67 Israel First NDC

68 Jamaica First NDC

69 Japan First NDC

70 Jordan First NDC

71 Kazakhstan First NDC

72 Kenya First NDC

73 Kiribati First NDC

74 Kuwait First NDC

75 Kyrgyzstan First NDC

76 Lao People's Democratic Republic First NDC

77 Lebanon First NDC

78 Lesotho First NDC

79 Liberia First NDC

80 Liechtenstein First NDC

81 Madagascar First NDC

82 Malawi First NDC

83 Malaysia First NDC

84 Maldives First NDC

85 Mali First NDC

86 Marshall Islands First NDC (**updated**)

87 Mauritania First NDC

88 Mauritius First NDC

89 Mexico First NDC

90 Micronesia First NDC

91 Monaco First NDC

92 Mongolia First NDC

93 Montenegro First NDC

94 Morocco First NDC

95 Mozambique First NDC

96 Myanmar First NDC

97 Namibia First NDC

98 Nauru First NDC

99 Nepal First NDC

100 New Zealand First NDC

101 Nicaragua First NDC

102 Niger First NDC

103 Nigeria First NDC

104 Niue First NDC

105 Oman First NDC

106 Pakistan First NDC

107 Palau First NDC

108 Panama First NDC

109 Papua New Guinea First NDC

110 Paraguay First NDC

111 Peru First NDC

112 Qatar First NDC

113 Republic of Korea First NDC

114 Republic of Moldova First NDC

115 Rwanda First NDC

116 Saint Kitts and Nevis First NDC

117 Saint Lucia First NDC

118 Samoa First NDC

119 San Marino First NDC

120 Sao Tome and Principe First NDC

121 Saudi Arabia First NDC

122 Serbia First NDC

123 Seychelles First NDC

124 Sierra Leone First NDC

125 Singapore First NDC

126 Solomon Islands First NDC

127 Somalia First NDC

128 South Africa First NDC

129 Sri Lanka First NDC

130 St Vincent and the Grenadines First NDC

131 State of Palestine First NDC

132 Sudan First NDC

133 Suriname First NDC

134 Switzerland First NDC

135 Syrian Arabic Republic First NDC

136 Tajikistan First NDC

137 Thailand First NDC

138 The Republic of North Macedonia First NDC

139 The United Republic of Tanzania First NDC

140 Timor-Leste First NDC

141 Togo First NDC

142 Tonga First NDC

143 Trinidad and Tobago First NDC

144 Tunisia First NDC

145 Turkmenistan First NDC

146 Tuvalu First NDC

147 Uganda First NDC

148 Ukraine First NDC

149 United Arab Emirates First NDC

150 United States of America First NDC

151 Uruguay First NDC

152 Uzbekistan First NDC

153 Vanuatu First NDC

154 Venezuela First NDC

155 Viet Nam First NDC

156 Zambia First NDC

157 Zimbabwe First NDC

158 Norway First NDC

159 Austria First NDC (represented by Latvia NDC)

160 Belgium First NDC (represented by Latvia NDC)

161 Bulgaria First NDC (represented by Latvia NDC)

162 Cyprus First NDC (represented by Latvia NDC)

163 Croatia First NDC (represented by Latvia NDC)

164 Czechia First NDC (represented by Latvia NDC)

165 Denmark First NDC (represented by Latvia NDC)

166 Estonia First NDC (represented by Latvia NDC)

167 Finland First NDC (represented by Latvia NDC)

168 France First NDC (represented by Latvia NDC)

169 Germany First NDC (represented by Latvia NDC)

170 Greece First NDC (represented by Latvia NDC)

171 Hungary First NDC (represented by Latvia NDC)

172 Ireland First NDC (represented by Latvia NDC)

173 Italy First NDC (represented by Latvia NDC)

174 Latvia First NDC

175 Lithuania First NDC (represented by Latvia NDC)

176 Luxembourg First NDC (represented by Latvia NDC)

177 Malta First NDC (represented by Latvia NDC)

178 Netherlands First NDC (represented by Latvia NDC)

179 Poland First NDC (represented by Latvia NDC)

180 Portugal First NDC (represented by Latvia NDC)

181 Romania First NDC (represented by Latvia NDC)

182 Slovakia First NDC (represented by Latvia NDC)

183 Slovenia First NDC (represented by Latvia NDC)

184 Spain First NDC (represented by Latvia NDC)

185 Sweden First NDC (represented by Latvia NDC)

186 UK First NDC (represented by Latvia NDC)

Caveats

There may be cases within the NDCs where the discussion of health and climate change is split over two or more sentences, and where key identifiers for either the health-related category or exposure category are only implied. The researchers found that this was a rare occurrence that would not affect larger trends in the data.

Future form of the indicator

This indicator uses the data from all available first NDCs held on the UNFCCC NDC Registry.[239] The 2021 Lancet Countdown report will compare this to the second NDCs (enhanced for COP26) held on the UNFCCC registry.

Additional analysis

Arranging the NDC data according to World Bank Income and Lending Groups, it is clear that the higher the income group, the lower the average coverage of health, whether or not the High Income Group has the European Union weighted as one NDC or 27 (Figure 81). Almost 97% of Low Income country NDCs mentioned health, compared to just under 37% of High Income country NDCs.

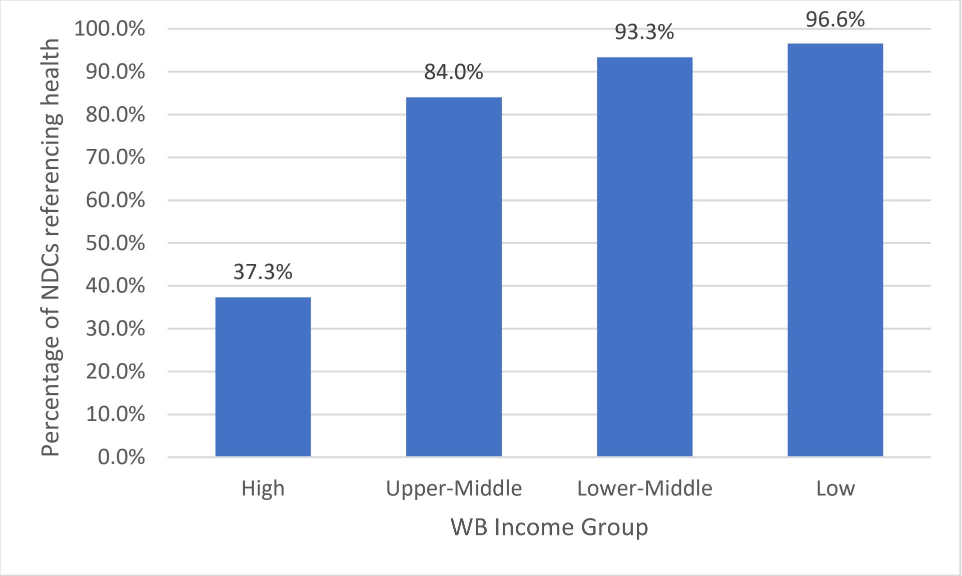

*Figure 81: Reference to health in the NDCs by WB Income and Lending Group (*High Income Group is weighted to take account of the single NDC representing 27 EU countries; treating the EU as one country would increase the group proportion to 65.6%).*

Health-related terms

Across the NDCs, 'health' and its derivatives are the most commonly used health-related term, appearing in 615 sentences (2.1% of all sentences). This is followed by 'disease' and its derivatives, which appears in 163 sentences across the NDCs (0.6% of all sentences). The next highest is a specific disease, 'malaria', employed in 40 sentences (0.14% of all sentences). Figure 82 demonstrates the most frequent health-related terms (derivatives of health, disease, malnutrition, and death) per WHO region (adjusted for number of countries per region). This demonstrates that particular health-related issues are more prominent in certain places. For example, while 'health' is the highest across all regions, 'disease' has a greater relative significance in the South East Asian and African regions. Though with low absolute numbers, 'malnutrition' has highest frequency in the African region.

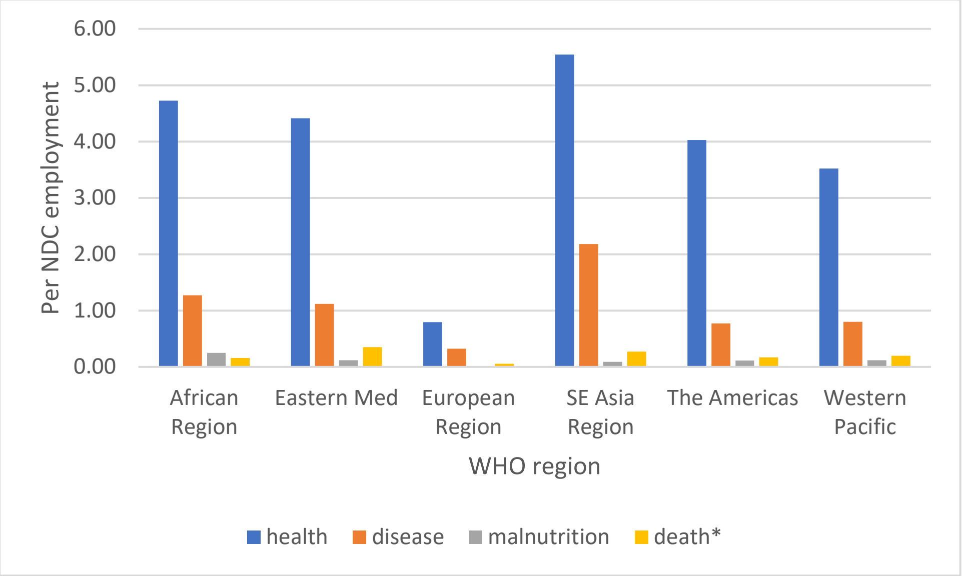

*Figure 82: Average employment of health-related terms in NDCs across WHO regions. (*While all terms have derivatives (e.g. 'healthcare' and 'healthy' for 'health'), death here also counts for 'loss of life', 'casualties' and 'lives lost').*

The most frequent *specific* health-related terms, and their regional distribution, can be seen in Figure 83. While both 'malaria' and 'dengue' are clearly of more concern across the South East Asian region, 'diarrhea' is not. On average, 'Malaria' is also the most frequently employed across the African, Eastern Mediterranean, and Western Pacific regions. 'Diarrhea' is most frequently referred to in the African and Western Pacific regions.

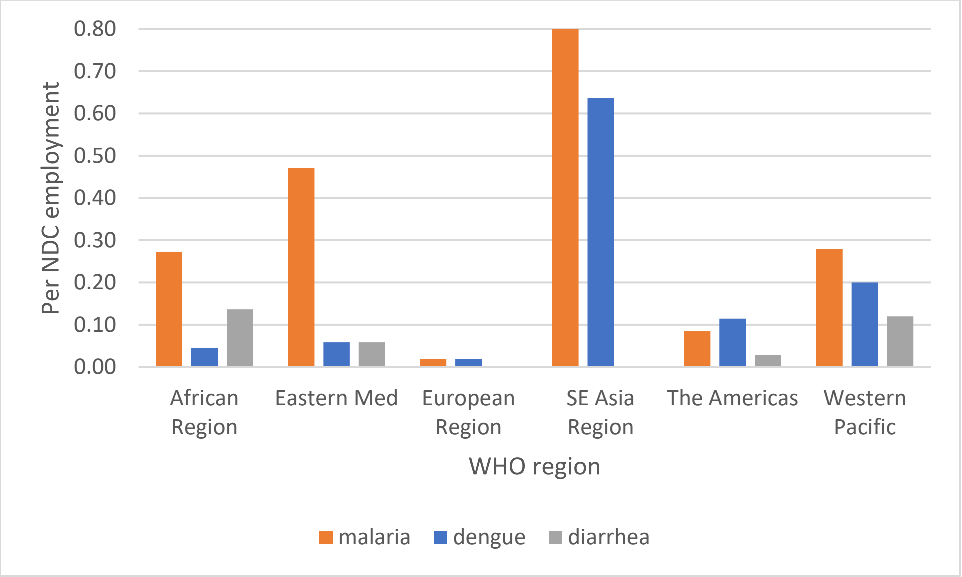

Figure 83: Average employment of specific health-related terms in NDCs across WHO regions.

Health-exposure terms

The most frequent health-exposure term that intersects with health-related terms relates to food security or food insecurity, followed by flood, drought, heatwave. Figure 84 through Figure 87 show the regional distribution of employment of these terms within the NDCs (including WHO regions along with the SIDS and the LDCs). Though low in absolute numbers, these demonstrate differences between regions regarding type or manner of health-exposure.

Figure 84 shows a reasonable distribution of the term 'flood' (and its derivatives) across regions, with the European region lowest (0.04 sentences per NDC). Both the Least Developed Countries (LDC) (0.43) and the Small Island Developing States (0.36) demonstrate a particular concern for floods. South East Asia (0.36) shows the greatest concern of all WHO regions, followed closely by The Americas (0.34), the Western Pacific (0.32), and the African Region (0.30).

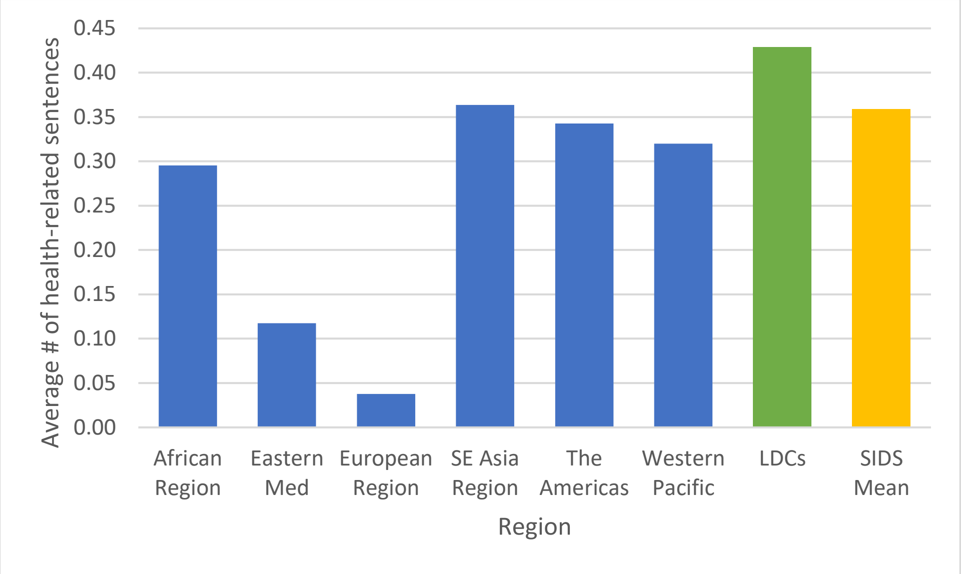

Figure 84: Average number of sentences per NDC with the health exposure 'flood' and a health-related term by region (WHO regions, including SIDS and LDCs).

Droughts (Figure 85) appear to be of particular concern for the South East Asian (0.36) region with a notable difference from other WHO regions. The LDCs (0.29), compared to other regions, are also higher in their concern for drought.

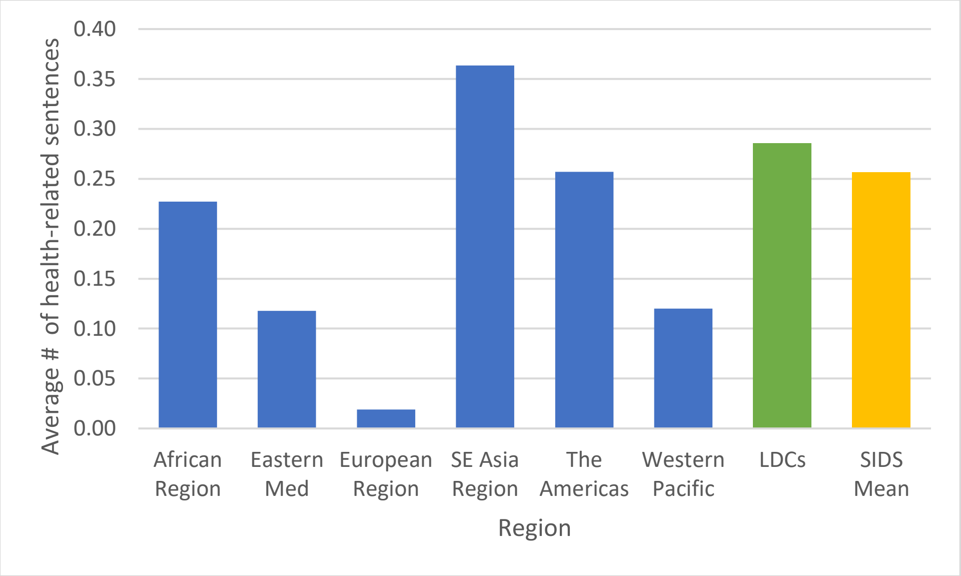

Figure 85: Figure A5.3180. Average number of sentences per NDC with the health exposure 'drought' and a health-related term by region (WHO regions, including SIDS and LDCs).

As with drought, both the South East Asian WHO (0.82) region and the LDCs (0.55) are highest in average number of sentences containing food security or insecurity and a health-related term (Figure 86). The European region is again, lowest with 0.04 sentences per NDC.

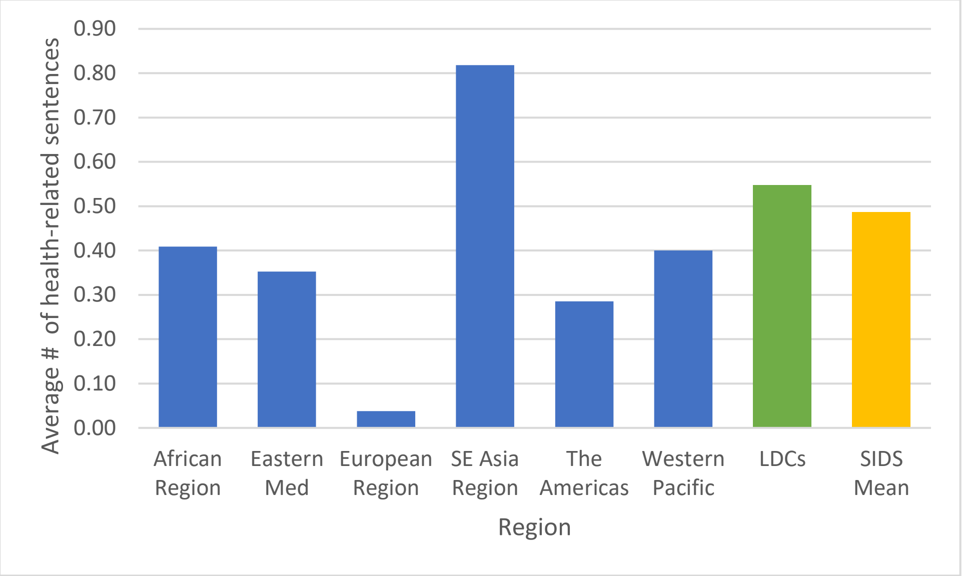

Figure 86: Average number of sentences per NDC with the health exposure 'food (in)security' and a health-related term by region (WHO regions, including SIDS and LDCs).

The Eastern Mediterranean region is the most concerned with the health exposure 'heat wave' (Figure 87), with 0.24 sentences per NDC containing the term along with a health-related term. The South East Asian region follows at 0.09 sentences per NDC, with the European Region at 0.06.

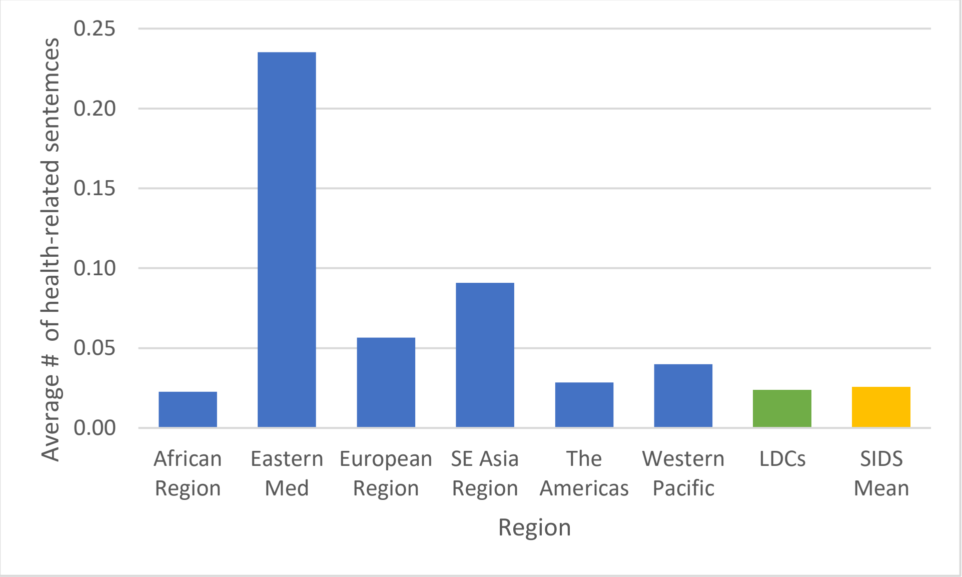

Figure 87: Average number of sentences per NDC with the health exposure 'heatwave' and a health-related term by region (WHO regions, including SIDS and LDCs).

Indicator 5.5: Corporate Engagement in Health and Climate Change in the Healthcare Sector

Methods

In order to produce the measure of engagement with climate change and health in healthcare companies' UN Global Compact Communication on Progress (UNGCCOP) reports, the publicly available COP reports were used. A total of 48,783 reports were downloaded from UNGCCOP. The reports are available for companies based in 129 countries.

UNGCCOP reports are submitted in 30 different languages. For the development of this indicator, only on the reports available in English (20,775) were examined, or around 43 per cent of the total number of UNGCCOP reports. A number of the English language files were corrupt or could not be converted into plain text format for analysis.

There are only single UNGCCOP report submissions before 2011, thus the sample of UNGCCOP reports is limited to the period 2011-2019. These documents were pre-processed and prepared for the application of natural language processing by converting the reports to plain text format; removing punctuation and numbers; removing stopwords; regularising (lowercasing); and stemming. All pre-processing and analysis was carried out in R using the "quanteda" package.[238]

The approach to using the UNGCCOP reports to produce the indicators is based on identifying references to key search terms linked to (a) health, and (b) climate change:

Health terms

- **malaria**
- **diarrhoea**
- **infection**
- **disease**
- **diseases**
- **sars**
- **measles**
- **pneumonia**
- **epidemic**
- **epidemics**
- **pandemic**
- **pandemics**
- **epidemiology**
- **healthcare**
- **health**
- **mortality**
- **morbidity**
- **nutrition**
- **illness**
- **illnesses**
- **ncd**
- **ncds**
- **air pollution**
- **nutrition**
- **malnutrition**
- **malnourishment**
- **mental disorder**
- **mental disorders**
- **stunting**

Climate change terms

- climate change
- changing climate
- climate emergency
- climate action
- climate crisis
- climate decay
- global warming
- green house
- temperature
- extreme weather
- global environmental change
- climate variability
- greenhouse
- greenhouse-gas
- low carbon
- ghge
- ghges
- renewable energy
- carbon emission
- carbon emissions
- carbon dioxide
- carbon-dioxide
- co2 emission
- co2 emissions
- climate pollutant
- climate pollutants
- decarbonization
- decarbonisation
- carbon neutral
- carbon-neutral

- carbon neutrality
- climate neutrality
- net-zero
- net zero

These key terms have been updated from previous years to reflect the changing terminology used to discuss climate change. In order to produce an indicator of engagement with the intersection of climate change and health, an examination of whether any of the climate change related terms appeared immediately before or after any public health terms in the UNGCCOP reports was undertaken. This was based on a search of the 25 words before and after a reference to a public health related term.

Data

1. UN Global Compact Communication on Progress reports.[240]

The distribution of available English-language reports over time is presented in Table 30:

Table 30: English -language UNGCCOP reports by year.

Year	Number of reports
2011	1268
2012	1767
2013	2129
2014	2258
2015	2450
2016	2650
2017	2662
2018	2661
2019	2930

Caveats

As noted above, only UNGCCOP reports that were submitted in English have been considered. This means a little under half of all available reports have been analysed.

This analysis here is based on a narrow range of search terms, which excludes reference to many of indirect links between climate change and health. Reports may also discuss indirect connections, such as the effect of climate change on agriculture, however, these are not included here. Therefore, the results present a somewhat conservative estimate of high corporate engagement with the intersection of climate change and health. Future work in this area will consider engagement with these indirect links, as well as providing additional forms of analysis.

Future form of the indicator

In the future, efforts will be made to increase the number of reports analysed by translating key search terms into several other key languages, and incorporating reports submitted in languages other than English into the sample. Translation of key terms has been implemented in WG5 into Spanish, Portuguese, and German. Plans are underway to expand the analysis using these translations for the 2021 Lancet Countdown report.

Additional analysis

Some additional findings and breakdowns are presented in this section. Figure 88 presents the total number of references to climate change, health, and the intersection of climate change and health across for the UNGCCOP

reports in the healthcare sector. Despite the increase in the proportion of companies engaging with the climate change-health linkages, the overall number of references remains fairly low and consistent throughout the time period.

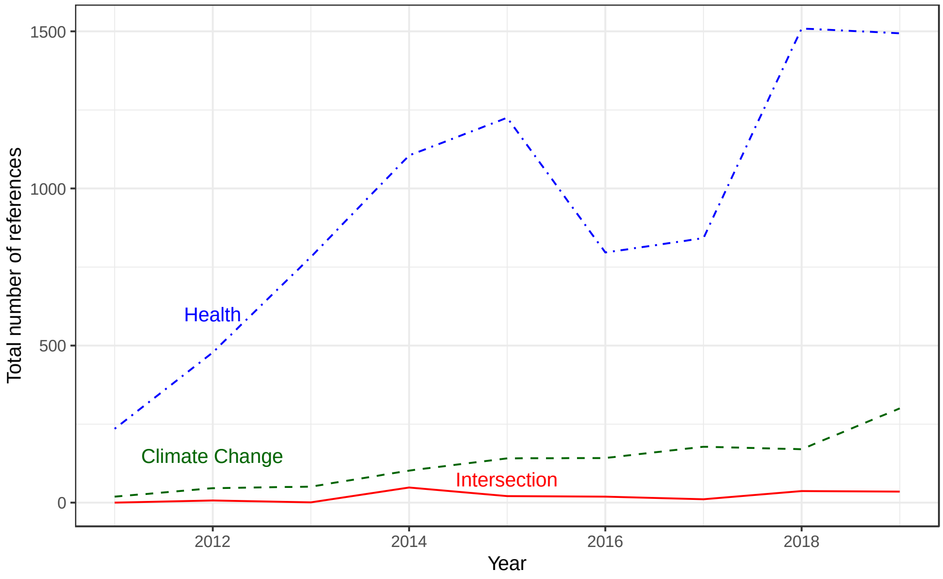

Figure 88: Total references to climate change, health, and the intersection of climate change and health in healthcare sector, 2011-2019.

In Figure 89, below, the total references with the intersection of climate change and health to better show any trends occurring in engagement are presented.

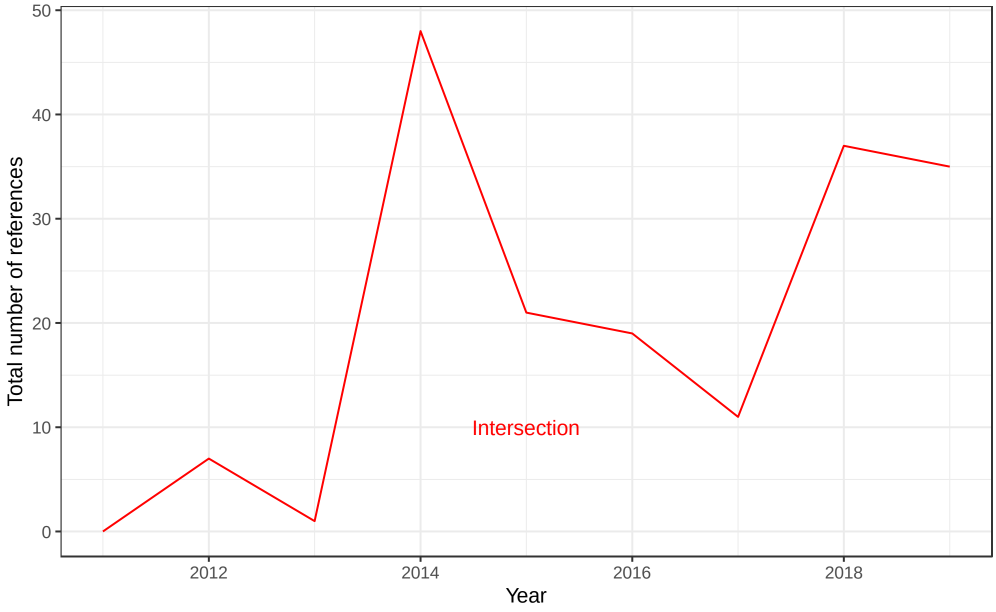

Figure 89: Total references to the intersection of climate change and health in healthcare sector, 2011-2019.

Figure 90 shows the average number of references to climate change, health, and the intersection in UNGCCOP reports from healthcare corporations. The figure again demonstrates the relatively low level of engagement with the health impacts of climate change in healthcare sector UNGCCOP reports.

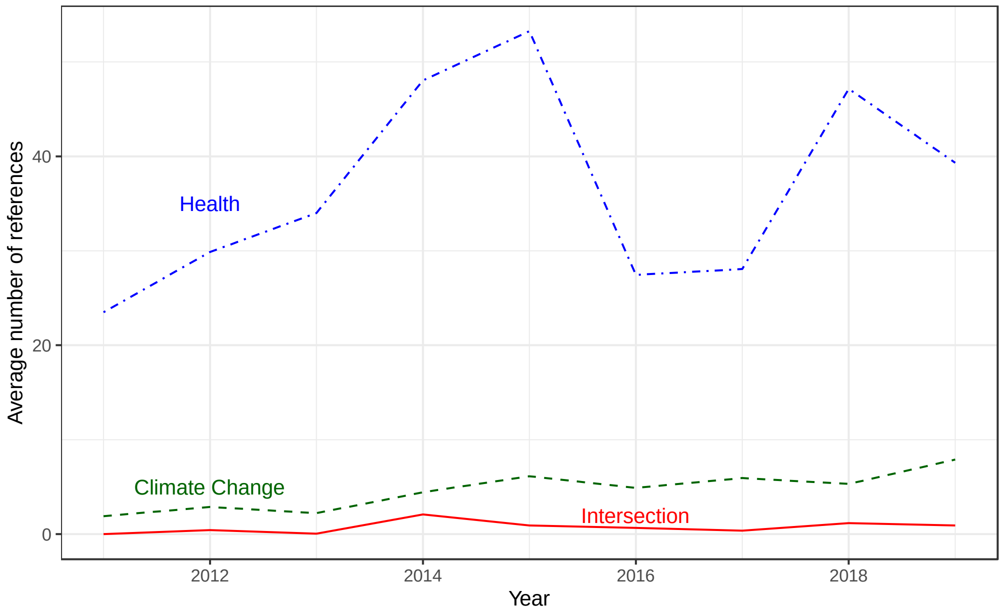

Figure 90: Average references to climate change, health, and the intersection of climate change and health in the healthcare sector COP reports, 2011-2019.

Engagement with climate change and health in the healthcare sector UNGCCOP reports by WHO region is also considered. Figure 91 shows the total number of references to the climate change-health intersection based on which of the WHO regions a healthcare company is based on, and Figure 92 shows the proportion of healthcare companies based in the different WHO regions that refer to the health impacts of climate change in their annual UNGCCOP report. These figures show that the highest proportion of UNGCCOP reports engaging with the climate change-health intersection in recent years has come from corporations based in Europe, which has significantly more engagement with the climate change-health relationship than other regions. The lowest engagement comes from corporations based in the Eastern Mediterranean region.

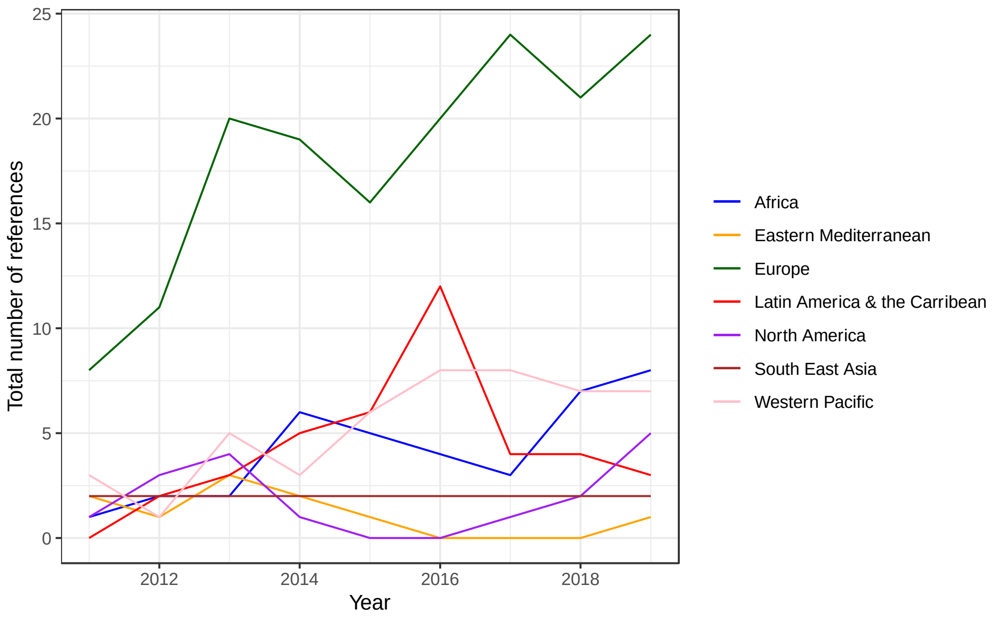

Figure 91: Total references with the intersection of climate change and health by WHO region, 2011-2019.

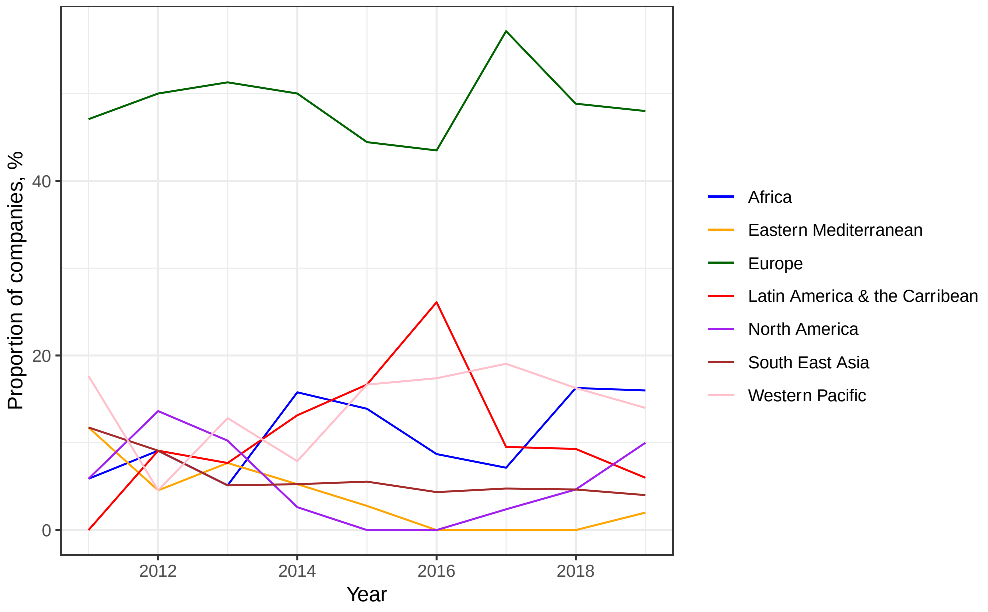

Figure 92: Proportion of companies referring to intersection of health and climate change by WHO region, 2011-2019.

Engagement across different sectors is also considered. Table 31 shows the total number of references to climate change, health, and the intersection across the different sectors in 2019, while Table 32 shows the proportion of corporations in each sector that engage with climate change, health, and the climate change-health intersection in 2019. Figure 93 presents the proportion of corporations engaging with the climate change-health relations in each sector in 2019 in the form of bar graphs.

Table 31: Total proportion of corporations in each sector engaging with the intersection of climate change and health in 2019.

	**Health**	**Climate change**	**Intersection**
**Aerospace & Defense**	20	16	3
**Alternative Energy**	15	14	11
**Automobiles & Parts**	54	41	16
**Banks**	54	46	13
**Beverages**	34	31	12
**Chemicals**	70	68	38
**Construction & Materials**	94	88	38
**Diversified**	50	45	11
**Electricity**	32	35	20
**Electronic & Electrical Equ...**	54	39	14
**Equity Investment Instruments**	7	6	2
**Financial Services**	126	125	42
**Fixed Line Telecommunications**	10	8	1
**Food & Drug Retailers**	2	2	2
**Food Producers**	67	55	24
**Forestry & Paper**	18	13	7
**Gas, Water & Multiutilities**	16	14	8
**General Industrials**	135	112	41
**General Retailers**	49	41	12

**Health Care Equipment & Ser...**	38	27	12
**Household Goods & Home Cons...**	26	20	7
**Industrial Engineering**	32	27	10
**Industrial Goods & Services**	0	0	0
**Industrial Metals & Mining**	26	25	10
**Industrial Transportation**	39	33	14
**Leisure Goods**	11	12	4
**Life Insurance**	7	7	3
**Media**	44	35	10
**Mining**	17	17	8
**Mobile Telecommunications**	25	20	11
**Nonequity Investment Instru...**	0	0	0
**Nonlife Insurance**	13	10	3
**Oil & Gas Producers**	33	32	24
**Oil Equipment, Services & D...**	23	17	6
**Personal Goods**	50	38	15
**Pharmaceuticals & Biotechno...**	53	38	24
**Real Estate**	1	1	0
**Real Estate Investment & Se...**	42	36	19
**Real Estate Investment Trusts**	8	8	4
**Retail**	1	1	1
**Software & Computer Services**	87	57	13
**Support Services**	191	147	30
**Technology Hardware & Equip...**	45	35	13
**Travel & Leisure**	51	48	12

Table 32: Total proportion of corporations in each sector engaging with the intersection of climate change and health in 2019.

	**Health**	**Climate change**	**Intersection**
**Aerospace & Defense**	54.0540541	43.2432432	8.10810811
**Alternative Energy**	57.6923077	53.8461538	42.3076923
**Automobiles & Parts**	70.1298701	53.2467532	20.7792208
**Banks**	65.060241	55.4216867	15.6626506
**Beverages**	62.962963	57.4074074	22.2222222
**Chemicals**	62.5	60.7142857	33.9285714
**Construction & Materials**	60.2564103	56.4102564	24.3589744
**Diversified**	52.6315789	47.3684211	11.5789474
**Electricity**	71.1111111	77.7777778	44.4444444
**Electronic & Electrical Equ...**	53.4653465	38.6138614	13.8613861
**Equity Investment Instruments**	53.8461538	46.1538462	15.3846154
**Financial Services**	55.7522124	55.3097345	18.5840708
**Fixed Line Telecommunications**	55.5555556	44.4444444	5.55555556
**Food & Drug Retailers**	33.3333333	33.3333333	33.3333333
**Food Producers**	67.6767677	55.5555556	24.2424242
**Forestry & Paper**	69.2307692	50	26.9230769

**Gas, Water & Multiutilities**	76.1904762	66.6666667	38.0952381
**General Industrials**	58.6956522	48.6956522	17.826087
**General Retailers**	53.2608696	44.5652174	13.0434783
**Health Care Equipment & Ser...**	76	54	24
**Household Goods & Home Cons...**	72.2222222	55.5555556	19.4444444
**Industrial Engineering**	49.2307692	41.5384615	15.3846154
**Industrial Goods & Services**	0	0	0
**Industrial Metals & Mining**	59.0909091	56.8181818	22.7272727
**Industrial Transportation**	56.5217391	47.826087	20.2898551
**Leisure Goods**	64.7058824	70.5882353	23.5294118
**Life Insurance**	58.3333333	58.3333333	25
**Media**	61.1111111	48.6111111	13.8888889
**Mining**	70.8333333	70.8333333	33.3333333
**Mobile Telecommunications**	78.125	62.5	34.375
**Nonequity Investment Instru...**	0	0	0
**Nonlife Insurance**	65	50	15
**Oil & Gas Producers**	56.8965517	55.1724138	41.3793103
**Oil Equipment, Services & D...**	60.5263158	44.7368421	15.7894737
**Personal Goods**	67.5675676	51.3513514	20.2702703
**Pharmaceuticals & Biotechno...**	72.6027397	52.0547945	32.8767123
**Real Estate**	100	100	0
**Real Estate Investment & Se...**	65.625	56.25	29.6875
**Real Estate Investment Trusts**	88.8888889	88.8888889	44.4444444
**Retail**	100	100	0
**Software & Computer Services**	58	38	10
**Support Services**	56.5088757	43.4911243	8.87573964
**Technology Hardware & Equip...**	56.25	43.75	16.25
**Travel & Leisure**	63.75	60	15

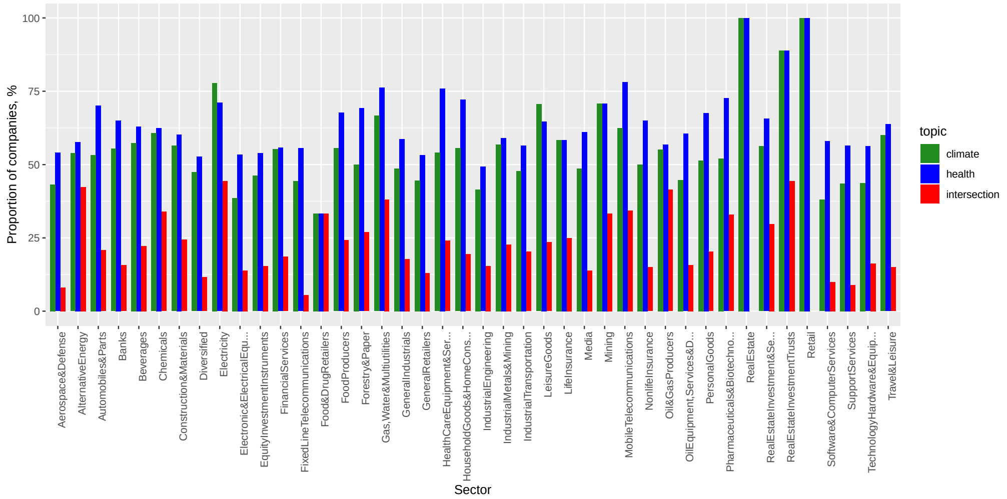

Figure 93: Proportion of corporations referring to climate change, health, and the intersection of climate change and health by sector in 2019.

As discussed in the main report, the highest level of engagement with the intersection of climate change and health in 2019 can be seen in the alternative energy, chemicals, electricity, mobile telecommunications, oil and

gas producers, and real estate investment sectors. In contrast, surprisingly lower levels of engagement are observed in the healthcare sector.

Additional information is also presented here on engagement with the climate change-health intersection in the healthcare sector, which is the focus in the main report. In addition to the total number of references to, and total proportion of reports that refer to, the climate change and health, the Tables below display the total number of references to each of the keywords related to health (Table 33) and climate change (Table 34) in the UNGCCOP reports of corporations in the health care sector for 2019.

Table 33: Total references to public health-related keywords in healthcare sector UNGCCOP reports in 2019.

**Keywords**	**Count**
health	4819
healthcare	2315
disease	317
diseases	313
infection	149
malaria	123
illness	87
nutrition	87
mortality	68
illnesses	63
ncds	33
pandemic	18
air_pollution	16
morbidity	11
malnutrition	10
epidemics	6
ncd	6
epidemic	5
measles	5
pandemics	5
stunting	4
malnourishment	2
sars	2
epidemiology	1
pneumonia	1

Table 34: Total references to climate change-related keywords in healthcare sector UNGCCOP reports in 2019.

**Keywords**	**Count**
greenhouse	394
climate_change	286
co2_emissions	148
renewable_energy	99
temperature	54
carbon_emissions	43
carbon_dioxide	24
low_carbon	23
global_warming	17
carbon_emission	13
carbon_neutral	11
climate_action	9
co2_emission	9
extreme_weather	8
carbon_neutrality	4
climate_neutrality	3
green_house	3
carbon-neutral	1

In addition to looking at companies by WHO region, companies from different types of countries are also considered in terms of their potential importance and role in addressing issues related to climate change. This is provided in Figure 90. As noted in previous years' reports, SIDS have driven much of the engagement with the health impacts of climate change, as well as climate change more generally, in the UN General Assembly. As such, a SIDS grouping is included. Arguably the three most important countries/unions in addressing climate change are USA, China, and the EU. This grouping is referred to as Tier 1 countries in Figure 90. Finally, an additional grouping of countries that are also important in terms of their CO2 emissions, their influence in international politics, and their potential impact on addressing climate change is also considered. This grouping, referred to as Tier 2 countries includes: Poland, Australia, South Africa, Brazil, India, France, Germany, and Indonesia. Hence, companies in the healthcare sector are examined based on the type of country in which they are based in Figure 94 (total references) and Figure 95 (proportion of companies). The results show that the highest engagement with the climate change-health relationship tends to come from healthcare companies based in Tier 2 countries.

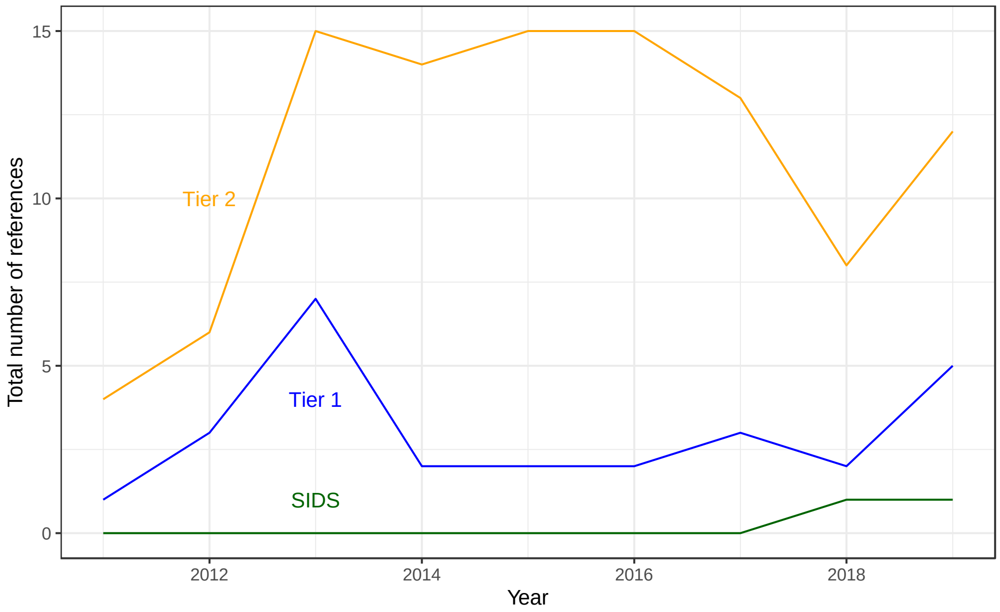

Figure 94: Total references to the climate change-health intersection in the healthcare sector by SIDS, Tier 1 countries, and Tier 2 countries, 2011-2019.

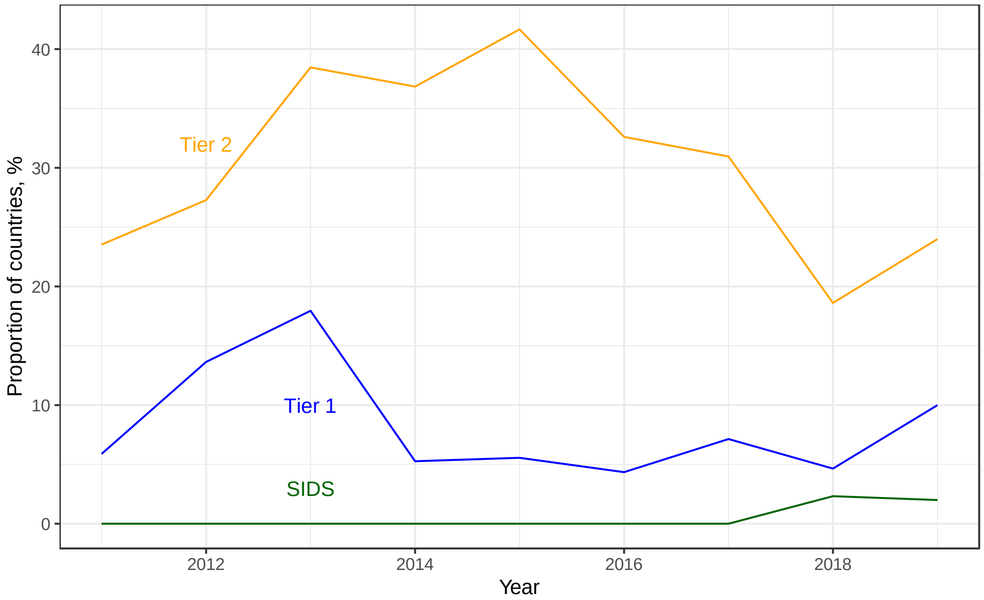

Figure 95: Proportion of corporations in the healthcare sector referring to the climate change-health intersection in the healthcare sector by SIDS, Tier 1 countries, and Tier 2 countries, 2011-2019.

References

1. Watts N, Amann M, Arnell N, et al. The 2019 report of The *Lancet* Countdown on health and climate change: ensuring that the health of a child born today is not defined by a changing climate. *The Lancet* 2019; **394**(10211): 1836-78.
2. Copernicus Climate Change Service (C3S). ERA5 hourly data on single levels from 1979 to present. Available at https://doi.org/10.24381/cds.adbb2d47. 2020.
3. Hersbach H, Bell B, Berrisford P, et al. Global reanalysis: goodbye ERA-Interim, hello ERA5. *ECMWF Newsl* 2019; **159**: 17-24.
4. NASA Socioeconomic Data and Applications Center (SEDAC) Gridded Population of the World (GPWv4). Available at https://beta.sedac.ciesin.columbia.edu/data/collection/gpw-v4. 2020.
5. The Inter-Sectoral Impact Model Intercomparison Project (ISIMP). Input data set: Historical, gridded population. Available at https://www.isimip.org/gettingstarted/input-data-bias-correction/details/31/. 2020.
6. Australian Bureau of Meteorology. Annual climate statement 2019. Accessed in April 2020 at http://www.bom.gov.au/climate/current/annual/aus/. 2019.
7. Watts N, Amann M, Arnell N, et al. The 2018 report of the Lancet Countdown on health and climate change: shaping the health of nations for centuries to come. *The Lancet* 2018; **392**(10163): 2479-514.
8. Global Burden of Disease Collaborative Network. Global Burden of Disease Study 2017 (GBD 2017) Population Estimates 1950-2017: Seattle, United States:Institute for Health Metrics and Evaluation (IHME), 2018.
9. NHS England, Public Health England. Reducing the use of natural resources in health and social care. London: NHS England, 2018.
10. NHS England. Greener NHS campaign to tackle climate 'health emergency'. 2020. https://www.england.nhs.uk/2020/01/greener-nhs-campaign-to-tackle-climate-health-emergency/ (accessed 26 April 2020).
11. World Health Organisation. IHR core capacity data (http://gamapserver.who.int/gho/interactive_charts/ihr/monitoring/atlas2.html?).
12. United Nations DESA/Population Division. 2018 Revision of the World Urbanization Prospects. 2018.
13. United Nations. 2019 Revision of World Population Prospects. Available at https://population.un.org/wpp/. 2020.
14. Agarwal V. Indian Heat Wave Breaks Record for Highest Temperature. 2016. https://blogs.wsj.com/indiarealtime/2016/05/20/indian-heat-wave-breaks-record-for-highest-temperature/ (accessed 5 May 2020).
15. NASA. Heat Wave Hits Thailand, India. 2016. https://earthobservatory.nasa.gov/images/87981/heat-wave-hits-thailand-india (accessed 5 May 2020).
16. Honda Y, Kondo M, McGregor G, et al. Heat-related mortality risk model for climate change impact projection. *Environ Health Prev Med* 2014; **19**(1): 56-63.
17. Institute for Health Metrics and Evaluation (IHME) Global Health Data Exchange (GHDx). Global Burden of Disease. Available at http://ghdx.healthdata.org/gbd-results-tool. 2020.
18. Kjellstrom T, Freyberg C, Lemke B, Otto M, Briggs D. Estimating population heat exposure and impacts on working people in conjunction with climate change. *International Journal of Biometeorology* 2018; **62**(3): 291-306.
19. CIESIN. Gridded population of the world (GWP). New York, NY, USA: Columbia University; 2017.
20. ILO. ILOSTAT database. Geneva, Switzerland: International Labour Organization; 2020.
21. NASA EarthData. Active Fire Data. Available at https://earthdata.nasa.gov/earth-observation-data/near-real-time/firms/active-fire-data. 2020.
22. Copernicus Climate Change Service (C3S). Fire danger indices historical data from the Copernicus Emergency Management Service. Available at https://doi.org/10.24381/cds.0e89c522. 2020.

23. Black C, Tesfaigzi Y, Bassein JA, Miller LA. Wildfire smoke exposure and human health: Significant gaps in research for a growing public health issue. *Environmental toxicology and pharmacology* 2017; **55**: 186-95.
24. Beguería S, Vicente-Serrano SM, Reig F, Latorre B. Standardized precipitation evapotranspiration index (SPEI) revisited: parameter fitting, evapotranspiration models, tools, datasets and drought monitoring. *International Journal of Climatology* 2014; **34**(10): 3001-23.
25. MeteoSwiss. 2020. https://www.meteoswiss.admin.ch/home/climate/swiss-climate-in-detail/climate-indicators/drought-indices/spi-and-spei.html (accessed 23 May 2020).
26. Copernicus Climate Change Service (C3S). ERA5 monthly averaged data on single levels from 1979 to present. Available at https://doi.org/10.24381/cds.f17050d7. 2020.
27. Global SPEI database. 2020.
28. Loecke TD, Burgin AJ, Riveros-Iregui DA, et al. Weather whiplash in agricultural regions drives deterioration of water quality. *Biogeochemistry* 2017; **133**(1): 7-15.
29. Centre for Research on the Epidemiology of Disasters. EM-DAT The International Disaster Database. Available at https://emdat.be/. 2020.
30. World Health Organization. Global Health Observatory. Available at https://apps.who.int/nha/database/Select/Indicators/en. 2020.
31. Herring SC, Christidis N, Hoell A, Hoerling MP, Stott PA. Explaining Extreme Events of 2017 from a Climate Perspective. *Bulletin of the American Meteorological Society* 2019; **100**(1): S1-S117.
32. Herring SC, Christidis N, Hoell A, Hoerling MP, Stott PA. Explaining Extreme Events of 2018 from a Climate Perspective. *Bulletin of the American Meteorological Society* 2020; **101**(1): S1-S128.
33. Herring SC, Christidis N, Hoell A, Kossin JP, III CJS, Stott PA. Explaining Extreme Events of 2016 from a Climate Perspective. *Bulletin of the American Meteorological Society* 2018; **99**(1): S1-S157.
34. Herring SC, Hoell A, Hoerling MP, Kossin JP, III CJS, Stott PA. Explaining Extreme Events of 2015 from a Climate Perspective. *Bulletin of the American Meteorological Society* 2016; **97**(12): S1-S145.
35. World Weather Attribution. World Weather Attribution. 2020. https://www.worldweatherattribution.org/ (accessed 10 May 2020).
36. Lindgren E, Andersson Y, Suk JE, Sudre B, Semenza JC. Monitoring EU emerging infectious disease risk due to climate change. *Science* 2012; **336**(6080): 418-9.
37. Stanaway JD, Shepard DS, Undurraga EA, et al. The global burden of dengue: an analysis from the Global Burden of Disease Study 2013. *The Lancet Infectious Diseases* 2016; **16**(6): 712-23.
38. Hales S, de Wet N, Maindonald J, Woodward A. Potential effect of population and climate changes on global distribution of dengue fever: an empirical model. *The Lancet* 2002; **360**(9336): 830-4.
39. Rocklöv J, Tozan Y. Climate change and the rising infectiousness of dengue. *Emerging Topics in Life Sciences* 2019; **3**(2): 133-42.
40. Liu-Helmersson J, Quam M, Wilder-Smith A, et al. Climate change and Aedes vectors: 21st century projections for dengue transmission in Europe. *EBioMedicine* 2016; **7**: 267-77.
41. Harris I, Osborn TJ, Jones P, Lister D. Version 4 of the CRU TS monthly high-resolution gridded multivariate climate dataset. *Scientific Data* 2020; **7**(1): 109.
42. Kraemer MU, Sinka ME, Duda KA, et al. The global distribution of the arbovirus vectors Aedes aegypti and Ae. albopictus. *Elife* 2015; **4**: e08347.
43. Liu-Helmersson J, Stenlund H, Wilder-Smith A, Rocklöv J. Vectorial capacity of Aedes aegypti: effects of temperature and implications for global dengue epidemic potential. *PloS one* 2014; **9**(3): e89783.
44. Grover-Kopec EK, Blumenthal MB, Ceccato P, Dinku T, Omumbo JA, Connor SJ. Web-based climate information resources for malaria control in Africa. *Malaria journal* 2006; **5**(1): 38.
45. Lyon B, Dinku T, Raman A, Thomson MC. Temperature suitability for malaria climbing the Ethiopian Highlands. *Environmental Research Letters* 2017; **12**(6): 064015.
46. Koninklijk Nederlands Meteorologisch Instituut. KNMI Climate Explorer. Available at https://climexp.knmi.nl/. 2020.

47. University of Washington Joint Institute for the Study of the Atmosphere and Ocean (JISAO). Elevation data. Available at http://research.jisao.washington.edu/data_sets/elevation/. 2020.
48. New M, Lister D, Hulme M, Makin I. A high-resolution data set of surface climate over global land areas. *Climate research* 2002; **21**(1): 1-25.
49. Feachem RG, Chen I, Akbari O, et al. Malaria eradication within a generation: ambitious, achievable, and necessary. *The Lancet* 2019; **394**(10203): 1056-112.
50. Gething PW, Van Boeckel TP, Smith DL, et al. Modelling the global constraints of temperature on transmission of Plasmodium falciparum and P. vivax. *Parasites & vectors* 2011; **4**(1): 92.
51. Snow RW, Sartorius B, Kyalo D, et al. The prevalence of Plasmodium falciparum in sub-Saharan Africa since 1900. *Nature* 2017; **550**(7677): 515-8.
52. Jacobs JM, Rhodes M, Brown CW, et al. Modeling and forecasting the distribution of Vibrio vulnificus in Chesapeake Bay. *J Appl Microbiol* 2014; **117**(5): 1312-27.
53. McLaughlin JB, DePaola A, Bopp CA, et al. Outbreak of Vibrio parahaemolyticus gastroenteritis associated with Alaskan oysters. *N Engl J Med* 2005; **353**(14): 1463-70.
54. Martinez-Urtaza J, van Aerle R, Abanto M, et al. Genomic Variation and Evolution of Vibrio parahaemolyticus ST36 over the Course of a Transcontinental Epidemic Expansion. *mBio* 2017; **8**(6).
55. Muhling BA, Gaitán CF, Stock CA, Saba VS, Tommasi D, Dixon KW. Potential Salinity and Temperature Futures for the Chesapeake Bay Using a Statistical Downscaling Spatial Disaggregation Framework. *Estuaries and Coasts* 2017; **41**(2): 349-72.
56. Parveen S, Hettiarachchi KA, Bowers JC, et al. Seasonal distribution of total and pathogenic Vibrio parahaemolyticus in Chesapeake Bay oysters and waters. *International journal of food microbiology* 2008; **128**(2): 354-61.
57. NOAA Earth System Research Laboratory. Optimum Interpolation 1/4 Degree Daily Sea Surface Temperature Analysis version 2. Available at https://www.ncdc.noaa.gov/oisst. 2020.
58. Copernicus Marine Environment Monitoring Service. Mercator Ocean Reanalysis. Available at http://marine.copernicus.eu/. 2020.
59. World Health Organisation. IHR Core capacity Monitoring Framework: Checklist and Indicators for Monitoring Progress in the Development of IHR Core Capacities in States Parties. 2013.
60. Semenza JC, Sewe MO, Lindgren E, et al. Systemic Resilience to Cross-border Infectious Disease Threat Events in Europe. *Transboundary and emerging diseases* 2019.
61. Jia P, Lu L, Chen X, et al. A climate-driven mechanistic population model of Aedes albopictus with diapause. *Parasites & Vectors* 2016; **9**(1): 175.
62. Gourdji SM, Sibley AM, Lobell DB. Global crop exposure to critical high temperatures in the reproductive period: historical trends and future projections. *Environmental Research Letters* 2013; **8**(2): 024041.
63. Lobell DB, Banziger M, Magorokosho C, Vivek B. Nonlinear heat effects on African maize as evidenced by historical yield trials. *Nature Climate Change* 2011; **1**(1): 42-5.
64. Challinor AJ, Koehler AK, Ramirez-Villegas J, Whitfield S, Das B. Current warming will reduce yields unless maize breeding and seed systems adapt immediately. *Nature Climate Change* 2016; **6**(10): 954-+.
65. Lobell DB, Schlenker W, Costa-Roberts J. Climate trends and global crop production since 1980. *Science* 2011; **333**(6042): 616-20.
66. Aslam MA, Ahmed M, Stöckle CO, Higgins SS, Hayat R. Can growing degree days and photoperiod predict spring wheat phenology? *Frontiers in Environmental Science* 2017; **5**: 57.
67. Portmann FT, Siebert S, Döll P. MIRCA2000—Global monthly irrigated and rainfed crop areas around the year 2000: A new high-resolution data set for agricultural and hydrological modeling. *Global biogeochemical cycles* 2010; **24**(1).
68. Sacks WJ, Deryng D, Foley JA, Ramankutty N. Crop planting dates: an analysis of global patterns. *Global Ecology and Biogeography* 2010; **19**(5): 607-20.
69. FAO. The State of World Fisheries and Aquaculture 2018 - Meeting the sustainable development goals. Rome: Food and Agriculture Organization of the United Nations, 2018.
70. Hoegh-Guldberg O, Cai R, Poloczanska E, Brewer P, Sundby S, Hilmi K. The Ocean In: Barros VR, Field CB, Dokken DJ, Mastrandrea MD, Mach KJ, Bilir TE, et al., editors. Climate

Change 2014: Impacts, Adaptation, and Vulnerability Part B: Regional Aspects Contribution of Working Group II to the Fifth Assessment Report of the Intergovernmental Panel of Climate Change. Cambridge, United Kingdom and New York, NY. USA: Cambridge University Press; 2014.
71. GBD 2017 Diet Collaborators, Afshin A, Sur PJ, et al. Health effects of dietary risks in 195 countries, 1990-2017: a systematic analysis for the Global Burden of Disease Study 2017. *Lancet (London, England)* 2019; **0**(0).
72. NASA Earth Observations (NEO). Sea surface temperature (1 month—AQUA/MODIS). Available at https://neo.sci.gsfc.nasa.gov/view.php?datasetId=MYD28M. 2020.
73. National Oceanic and Atmospheric Administration (NOAA). Coral Reef Watch Version 3.1 Daily Global 5-km Satellite Coral Bleaching Degree Heating Week Product. 2020.
74. FAO. New Food Balance Sheets. 2020. http://www.fao.org/faostat/en/#data/FBS (accessed 19 February 2020).
75. Kopp RE, DeConto RM, Bader DA, et al. Evolving Understanding of Antarctic Ice-Sheet Physics and Ambiguity in Probabilistic Sea-Level Projections. *Earth's Future* 2017; **5**(12): 1217-33.
76. Kulp SA, Strauss BH. CoastalDEM: a global coastal digital elevation model improved from SRTM using a neural network. *Remote sensing of environment* 2018; **206**: 231-9.
77. Bright EA, Rose AN, Urban ML, McKee J. LandScan 2017 High-Resolution Global Population Data Set: Oak Ridge National Lab.(ORNL), Oak Ridge, TN (United States), 2018.
78. Global Administrative Areas (GADM) version 3.6. Available at http://www.gadm.org/. 2020.
79. Pörtner H, Roberts D, Masson-Delmotte V, et al. IPCC Special Report on the Ocean and Cryosphere in a Changing Climate. *IPCC Intergovernmental Panel on Climate Change: Geneva, Switzerland* 2019.
80. Thomas MA, Lin T. Illustrative Analysis of Probabilistic Sea Level Rise Hazard. *Journal of Climate* 2020; **33**(4): 1523-34.
81. Hinkel J, Lincke D, Vafeidis AT, et al. Coastal flood damage and adaptation costs under 21st century sea-level rise. *Proceedings of the National Academy of Sciences* 2014; **111**(9): 3292-7.
82. Lichter M, Vafeidis AT, Nicholls RJ, Kaiser G. Exploring data-related uncertainties in analyses of land area and population in the "Low-Elevation Coastal Zone"(LECZ). *Journal of Coastal Research* 2011; **27**(4): 757-68.
83. Mondal P, Tatem AJ. Uncertainties in measuring populations potentially impacted by sea level rise and coastal flooding. *PLoS One* 2012; **7**(10).
84. Kulp SA, Strauss BH. New elevation data triple estimates of global vulnerability to sea-level rise and coastal flooding. *Nature communications* 2019; **10**(1): 1-12.
85. Black R, Adger WN, Arnell NW, Dercon S, Geddes A, Thomas D. The effect of environmental change on human migration. *Global environmental change* 2011; **21**: S3-S11.
86. Hauer ME, Fussell E, Mueller V, et al. Sea-level rise and human migration. *Nature Reviews Earth & Environment* 2019: 1-12.
87. Ayeb-Karlsson S, Kniveton D, Cannon T. Trapped in the prison of the mind: Notions of climate-induced (im)mobility decision-making and wellbeing from an urban informal settlement in Bangladesh. *Palgrave Communications* 2020: forthcoming.
88. Ayeb-Karlsson S, Smith CD, Kniveton D. A discursive review of the textual use of 'trapped'in environmental migration studies: The conceptual birth and troubled teenage years of trapped populations. *Ambio* 2018; **47**(5): 557-73.
89. Kummu M, De Moel H, Salvucci G, Viviroli D, Ward PJ, Varis O. Over the hills and further away from coast: global geospatial patterns of human and environment over the 20th–21st centuries. *Environmental Research Letters* 2016; **11**(3): 034010.
90. McGranahan G, Balk D, Anderson B. The rising tide: assessing the risks of climate change and human settlements in low elevation coastal zones. *Environment and urbanization* 2007; **19**(1): 17-37.
91. Neumann B, Vafeidis AT, Zimmermann J, Nicholls RJ. Future coastal population growth and exposure to sea-level rise and coastal flooding-a global assessment. *PloS one* 2015; **10**(3): e0118571.
92. Dannenberg AL, Frumkin H, Hess JJ, Ebi KL. Managed retreat as a strategy for climate change adaptation in small communities: public health implications. *Climatic change* 2019; **153**(1-2): 1-14.

93. Schütte S, Gemenne F, Zaman M, Flahault A, Depoux A. Connecting planetary health, climate change, and migration. *The Lancet Planetary Health* 2018; **2**(2): e58-e9.
94. Luber G, Knowlton K, Balbus J, et al. Human health. *Climate change impacts in the United States: the third National Climate Assessment* 2014: 220-56.
95. McMichael C, Katonivualiku M, Powell T. Planned relocation and everyday agency in low-lying coastal villages in Fiji. *The Geographical Journal* 2019; **185**(3): 325-37.
96. Diaz DB. Estimating global damages from sea level rise with the Coastal Impact and Adaptation Model (CIAM). *Climatic change* 2016; **137**(1-2): 143-56.
97. Jevrejeva S, Jackson L, Grinsted A, Lincke D, Marzeion B. Flood damage costs under the sea level rise with warming of 1.5 C and 2 C. *Environmental Research Letters* 2018; **13**(7): 074014.
98. International Organization for Migration. Implementation of the Workplan of the Task Force on Displacement under the Warsaw International Mechanism for Loss and Damage, United Nations Framework Convention on Climate Change (UNFCCC), Pillar I: Policy/Practice – National/Subnational Activity I.1: Mapping Human Mobility and Climate Change in Relevant National Policies and Institutional Frameworks 2018. https://environmentalmigration.iom.int/iom-pdd-task-force-displacement-stakeholder-meeting.
99. Watts N, Amann M, Ayeb-Karlsson S, et al. The Lancet Countdown on health and climate change: from 25 years of inaction to a global transformation for public health. *The Lancet* 2017.
100. Abubakar I, Aldridge RW, Devakumar D, et al. The UCL–Lancet Commission on Migration and Health: the health of a world on the move. *The Lancet* 2018; **392**(10164): 2606-54.
101. Baldwin A. Premediation and white affect: climate change and migration in critical perspective. *Transactions of the Institute of British Geographers* 2016; **41**(1): 78-90.
102. Kelman I. Difficult decisions: migration from small island developing states under climate change. *Earth's Future* 2015; **3**(4): 133-42.
103. Kelman I. Islandness within climate change narratives of small island developing states (SIDS). *Island Studies Journal* 2018; **13**(1): 149-66.
104. WHO. WHO Health and Climate Change Survey Report: Tracking Global Progress. Geneva, Switzerland: World Health Organization, 2019.
105. Berry P, Enright PM, Shumake-Guillemot J, Villalobos Prats E, Campbell-Lendrum D. Assessing health vulnerabilities and adaptation to climate change: A review of international progress. *International Journal of Environmental research and public health* 2018; **15**(12): 2626.
106. CDP. Annual Cities Survey Data. In: CDP, editor. London, UK; 2020.
107. WMO. Country Profile Database. 2019.
108. WHO. Global Health Observatory Data Repository. 2019.
109. Bouchama A, Dehbi M, Mohamed G, Matthies F, Shoukri M, Menne B. Prognostic factors in heat wave related deaths: a meta-analysis. *Archives of internal medicine* 2007; **167**(20): 2170-6.
110. Miettinen OS. Proportion of disease caused or prevented by a given exposure, trait or intervention. *American journal of epidemiology* 1974; **99**(5): 325-32.
111. Ciancio B, Di Renzi M, Binkin N, et al. Fattori di rischio per la mortalita durante un'ondata di calore a Bari, estate 2005. *Bollettino Epidemiologico Nazionale* 2007; **63**: 113-25.
112. Lorente C, Serazin C, Salines G, et al. Etude des facteurs de décès des personnes âgées résidant en établissement durant la vague de chaleur d'août 2003: Institut de Veille Sanitaire (InVS), 2005.
113. Kaiser R, Rubin CH, Henderson AK, et al. Heat-related death and mental illness during the 1999 Cincinnati heat wave. *The American journal of forensic medicine and pathology* 2001; **22**(3): 303-7.
114. Marmor M. Heat wave mortality in nursing homes. *Environmental Research* 1978; **17**(1): 102-15.
115. Naughton MP, Henderson A, Mirabelli MC, et al. Heat-related mortality during a 1999 heat wave in Chicago. *American journal of preventive medicine* 2002; **22**(4): 221-7.
116. Semenza JC, Rubin CH, Falter KH, et al. Heat-related deaths during the July 1995 heat wave in Chicago. *New England journal of medicine* 1996; **335**(2): 84-90.
117. Zhang Y, Nitschke M, Krackowizer A, et al. Risk factors for deaths during the 2009 heat wave in Adelaide, Australia: a matched case-control study. *Int J Biometeorol* 2017; **61**(1): 35-47.

118. Theocharis G, Tansarli G, Mavros M, Spiropoulos T, Barbas S, Falagas M. Association between use of air-conditioning or fan and survival of elderly febrile patients: a prospective study. *European journal of clinical microbiology & infectious diseases* 2013; **32**(9): 1143-7.
119. Vandentorren S, Bretin P, Zeghnoun A, et al. August 2003 heat wave in France: risk factors for death of elderly people living at home. *The European Journal of Public Health* 2006; **16**(6): 583-91.
120. European Commission. Urban Centre Database GHS-UCDB R2019A. 2020 (accessed 5 May 2020).
121. Center for International Earth Science Information Network - CIESIN - Columbia University. Gridded Population of the World, Version 4 (GPWv4): Population Density, Revision 11. In: (SEDAC) NSDaAC, editor. Palisades, NY; 2018.
122. Kriegler FL, Malila WA, Nalekpa RF, Richardson W. Preprocessing Transformations and Their Effects on Multispectral Recognition. *Proceedings of the Sixth International Symposium on Remote Sensing of Environment* 1969; **II**: 97.
123. NASA LP DAAC. MOD13Q1.006 Terra Vegetation Indices 16-Day Global 250m.
124. Carroll M, DiMiceli C, Sohlberg R, Townshend J. MODIS Normalized Difference Vegetation Index. In: Facility GLC, editor. College Park, MD.
125. Rhew IC, Vander Stoep A, Kearney A, Smith NL, Dunbar MD. Validation of the Normalized Difference Vegetation Index as a Measure of Neighborhood Greenness. *Annals of Epidemiology* 2011; **21**(12): 946-52.
126. Fong KC, Hart JE, James P. A Review of Epidemiologic Studies on Greenness and Health: Updated Literature Through 2017. *Current Environmental Health Reports* 2018; **5**(1): 77-87.
127. Fong KC, Kloog I, Coull BA, et al. Residential Greenness and Birthweight in the State of Massachusetts, USA. *International Journal of Environmental Research and Public Health* 2018; **15**(6).
128. James P, Banay R, Hart J, Laden F. A Review of the Health Benefits of Greenness. *Current Epidemiology Reports* 2015; **2**(2): 131-42.
129. Florczyk AJ, Melchiorri M, Corbane C, et al. Description of the GHS Urban Centre Database 2015. Brussels, Belgium: European Commission - DG Joint Research Centre, 2019.
130. kMatrix Ltd. Adaptation and Resilience to Climate Change dataset. 2020.
131. Jaikumar R. Postindustrial Manufacturing. Harvard Business Review. 1986.
132. Georgeson L, Maslin M, Poessinouw M. The global green economy: a review of concepts, definitions, measurement methodologies and their interactions. *Geo: Geography and Environment* 2017; **4**(1): e00036.
133. IEA. CO2 Emissions from Fuel Combustion (2019 edition). UK Data Service; 2019.
134. IEA. World Energy Outlook 2019. Paris: IEA, 2019.
135. IEA. IEA Statistical Report. Paris: IEA, 2020.
136. IEA. Global Energy Review 2020. Paris, France: International Energy Agency, 2020.
137. IEA. World Extended Energy Balances (2019 edition). UK Data Service; 2020.
138. IEA. World Extended Energy Balances. UK Data Service; 2020.
139. IEA. Methodology. Defining energy access. 2019. https://www.iea.org/energyaccess/methodology/ (accessed 6 June 2019).
140. WHO. Indicator 7.1.2: Proportion of population with primary reliance on clean fuels and technology. 19 July 2016 2016. https://unstats.un.org/sdgs/metadata/files/Metadata-07-01-02.pdf (accessed 8 June 2019).
141. Bonjour S, Adair-Rohani H, Wolf J, et al. Solid fuel use for household cooking: country and regional estimates for 1980–2010. *Environmental Health Perspectives* 2013; **121**(7): 784-90.
142. WHO. Household Energy Database. Geneva: World Health Organization; 2020.
143. UNDESA. World Population Prospects: The 2017 revision. New York, NY, USA: United Nations Department of Economic and Social Affairs; 2017.
144. Roth GA, Abate D, Abate KH, et al. Global, regional, and national age-sex-specific mortality for 282 causes of death in 195 countries and territories, 1980–2017: a systematic analysis for the Global Burden of Disease Study 2017. *The Lancet* 2018; **392**(10159): 1736-88.

145. Amann M, Bertok I, Borken-Kleefeld J, et al. Cost-effective control of air quality and greenhouse gases in Europe: Modeling and policy applications. *Environmental Modelling & Software* 2011; **26**(12): 1489-501.
146. Simpson D, Benedictow A, Berge H, et al. The EMEP MSC-W chemical transport model–technical description. *Atmospheric Chemistry and Physics* 2012; **12**(16): 7825-65.
147. Kiesewetter G, Borken-Kleefeld J, Schöpp W, et al. Modelling street level PM 10 concentrations across Europe: source apportionment and possible futures. *Atmospheric Chemistry and Physics* 2015; **15**(3): 1539-53.
148. Amann M, Kiesewetter G, Schöpp W, et al. Reducing global air pollution: the scope for further policy interventions. *Philosophical Transactions of the Royal Society A* 2020; **20190331**.
149. WHO. WHO Global Urban Ambient Air Pollution Database (update 2018). Geneva, Switzerland: World Health Organization; 2018.
150. WHO. Ambient Air Pollution: A global assessment of exposure and burden of disease. Geneva, Switzerland: World Health Organization, 2016.
151. Forouzanfar MH, Alexander L, Anderson HR, et al. Global, regional, and national comparative risk assessment of 79 behavioural, environmental and occupational, and metabolic risks or clusters of risks in 188 countries, 1990–2013: a systematic analysis for the Global Burden of Disease Study 2013. *The Lancet* 2015; **386**(10010): 2287-323.
152. IHME. GBD Results Query Tool. http://ghdx.healthdata.org/gbd-results-tool (accessed May 2016).
153. WHO European Centre for Environment and Health. Review of evidence on health aspects of air pollution - REVIHAAP Project. Copenhagen, Denmark: WHO Regional Office for Europe, 2013.
154. IEA. Global EV Outlook 2019: Scaling up the transistion to electric mobility. Paris, France: International Energy Agency; 2019.
155. Woodcock J, Givoni M, Morgan AS. Health impact modelling of active travel visions for England and Wales using an Integrated Transport and Health Impact Modelling Tool (ITHIM). *PLoS One* 2013; **8**(1): e51462.
156. Herrero M, Havlík P, Valin H, et al. Biomass use, production, feed efficiencies, and greenhouse gas emissions from global livestock systems. *Proceedings of the National Academy of Sciences* 2013; **110**(52): 20888-93.
157. Chang J, Ciais P, Herrero M, et al. Combining livestock production information in a process-based vegetation model to reconstruct the history of grassland management. *Biogeosciences* 2016; **13**(12): 3757-76.
158. Carlson KM, Gerber JS, Mueller ND, et al. Greenhouse gas emissions intensity of global croplands. *Nature Climate Change* 2017; **7**(1): 63-8.
159. FAO. FAOSTAT. 2020.
160. Dalin C, Wada Y, Kastner T, Puma MJ. Groundwater depletion embedded in international food trade. *Nature* 2017; **543**: 700–4.
161. Kastner T, Kastner M, Nonhebel S. Tracing distant environmental impacts of agricultural products from a consumer perspective. *Ecological Economics* 2011; **70**(6): 1032–40.
162. Willett W, Rockstrom J, Loken B, et al. Food in the Anthropocene: the EAT-Lancet Commission on healthy diets from sustainable food systems. *The Lancet* 2019; **393**(10170): 447-92.
163. FAO. Food balance sheets: a handbook. Rome, Italy: Food and Agriculture Organization of the United Nations; 2001.
164. Gustavsson J, Cederberg C, Sonesson U, Van Otterdijk R, Meybeck A. Global food losses and food waste: extent, causes and prevention. Rome, Italy: Food and Agriculture Organization of the United Nations, 2011.
165. Del Gobbo LC, Khatibzadeh S, Imamura F, et al. Assessing global dietary habits: a comparison of national estimates from the FAO and the Global Dietary Database. *The American journal of clinical nutrition* 2015; **101**(5): 1038-46.
166. Micha R, Khatibzadeh S, Shi P, Andrews KG, Engell RE, Mozaffarian D. Global, regional and national consumption of major food groups in 1990 and 2010: a systematic analysis including 266 country-specific nutrition surveys worldwide. *BMJ open* 2015; **5**(9): e008705.

167. Freedman LS, Commins JM, Moler JE, et al. Pooled results from 5 validation studies of dietary self-report instruments using recovery biomarkers for energy and protein intake. *American journal of epidemiology* 2014; **180**(2): 172-88.
168. Rennie KL, Coward A, Jebb SA. Estimating under-reporting of energy intake in dietary surveys using an individualised method. *British Journal of Nutrition* 2007; **97**(6): 1169-76.
169. NCD Risk Factor Collaboration. Trends in adult body-mass index in 200 countries from 1975 to 2014: a pooled analysis of 1698 population-based measurement studies with 19· 2 million participants. *The Lancet* 2016; **387**(10026): 1377-96.
170. GBD Risk Factors Collaborators. Global, regional, and national comparative risk assessment of 79 behavioural, environmental and occupational, and metabolic risks or clusters of risks in 188 countries, 1990–2013: a systematic analysis for the Global Burden of Disease Study 2013. *Lancet (London, England)* 2015; **386**(10010): 2287.
171. Lim SS, Vos T, Flaxman AD, et al. A comparative risk assessment of burden of disease and injury attributable to 67 risk factors and risk factor clusters in 21 regions, 1990–2010: a systematic analysis for the Global Burden of Disease Study 2010. *The Lancet* 2012; **380**(9859): 2224-60.
172. Murray CJL, Ezzati M, Lopez AD, Rodgers A, Vander Hoorn S. Comparative quantification of health risks: conceptual framework and methodological issues. *Population Health Metrics* 2003; **1**(1): 1-.
173. Murray CJL, Ezzati M, Flaxman AD, et al. GBD 2010: design, definitions, and metrics. *Lancet* 2012; **380**(9859): 2063-6.
174. Lozano R, Naghavi M, Foreman K, et al. Global and regional mortality from 235 causes of death for 20 age groups in 1990 and 2010: a systematic analysis for the Global Burden of Disease Study 2010. *The Lancet* 2012; **380**(9859): 2095-128.
175. Afshin A, Micha R, Khatibzadeh S, Mozaffarian D. Consumption of nuts and legumes and risk of incident ischemic heart disease, stroke, and diabetes: a systematic review and meta-analysis. *The American Journal of Clinical Nutrition* 2014; **100**(1): 278-88.
176. Aune D, Giovannucci E, Boffetta P, et al. Fruit and vegetable intake and the risk of cardiovascular disease, total cancer and all-cause mortality–a systematic review and dose-response meta-analysis of prospective studies. *International Journal of Epidemiology* 2016.
177. Aune D, Keum N, Giovannucci E, et al. Nut consumption and risk of cardiovascular disease, total cancer, all-cause and cause-specific mortality: a systematic review and dose-response meta-analysis of prospective studies. *BMC medicine* 2016; **14**(1): 207-.
178. Aune D, Keum N, Giovannucci E, et al. Whole grain consumption and risk of cardiovascular disease, cancer, and all cause and cause specific mortality: systematic review and dose-response meta-analysis of prospective studies. *BMJ (Clinical research ed)* 2016; **353**: i2716-i.
179. Bechthold A, Boeing H, Schwedhelm C, et al. Food groups and risk of coronary heart disease, stroke and heart failure: A systematic review and dose-response meta-analysis of prospective studies. *Critical Reviews in Food Science and Nutrition* 2019; **59**(7): 1071-90.
180. Schwingshackl L, Hoffmann G, Lampousi AM, et al. Food groups and risk of type 2 diabetes mellitus: a systematic review and meta-analysis of prospective studies. *European Journal of Epidemiology* 2017; **32**(5): 363-75.
181. Schwingshackl L, Schwedhelm C, Hoffmann G, et al. Food groups and risk of colorectal cancer. *International Journal of Cancer* 2018; **142**(9): 1748-58.
182. Zheng J, Huang T, Yu Y, Hu X, Yang B, Li D. Fish consumption and CHD mortality: an updated meta-analysis of seventeen cohort studies. *Public Health Nutrition* 2012; **15**(4): 725-37.
183. Singh GM, Danaei G, Farzadfar F, et al. The Age-Specific Quantitative Effects of Metabolic Risk Factors on Cardiovascular Diseases and Diabetes: A Pooled Analysis. *PLOS ONE* 2013; **8**(7): e65174-e.
184. Micha R, Shulkin ML, Peñalvo JL, et al. Etiologic effects and optimal intakes of foods and nutrients for risk of cardiovascular diseases and diabetes: Systematic reviews and meta-analyses from the Nutrition and Chronic Diseases Expert Group (NutriCoDE). *PLOS ONE* 2017; **12**(4): e0175149-e.
185. World Cancer Research Fund, American Institute for Cancer Research. Diet, Nutrition, Physical Activity and Cancer: A Global Perspective. Continuous Update Project Expert Report. London, UK: World Cancer Research Fund International, 2018.

186. Schwingshackl L, Knüppel S, Schwedhelm C, et al. Perspective: NutriGrade: A Scoring System to Assess and Judge the Meta-Evidence of Randomized Controlled Trials and Cohort Studies in Nutrition Research. *Advances in Nutrition: An International Review Journal* 2016; **7**(6): 994-1004.
187. Satija A, Yu E, Willett WC, Hu FB. Understanding nutritional epidemiology and its role in policy. *Advances in nutrition* 2015; **6**(1): 5-18.
188. Global Bmi Mortality Collaboration ED, Di Angelantonio E, Bhupathiraju S, et al. Body-mass index and all-cause mortality: individual-participant-data meta-analysis of 239 prospective studies in four continents. *Lancet (London, England)* 2016; **388**(10046): 776-86.
189. Prospective Studies Collaboration. Body-mass index and cause-specific mortality in 900 000 adults: collaborative analyses of 57 prospective studies. *The Lancet* 2009; **373**(9669): 1083-96.
190. Schwingshackl L, Hoffmann G, Iqbal K, Schwedhelm C, Boeing H. Food groups and intermediate disease markers: a systematic review and network meta-analysis of randomized trials. *The American journal of clinical nutrition* 2018; **108**(3): 576-86.
191. Aune D, Lau R, Chan DSM, et al. Dairy products and colorectal cancer risk: a systematic review and meta-analysis of cohort studies. *Annals of Oncology: Official Journal of the European Society for Medical Oncology* 2012; **23**(1): 37-45.
192. Aune D, Navarro Rosenblatt DA, Chan DSM, et al. Dairy products, calcium, and prostate cancer risk: a systematic review and meta-analysis of cohort studies. *The American Journal of Clinical Nutrition* 2015; **101**(1): 87-117.
193. Jayedi A, Shab-Bidar S, Eimeri S, Djafarian K. Fish consumption and risk of all-cause and cardiovascular mortality: a dose–response meta-analysis of prospective observational studies. *Public health nutrition* 2018; **21**(7): 1297-306.
194. Xun P, Qin B, Song Y, et al. Fish consumption and risk of stroke and its subtypes: accumulative evidence from a meta-analysis of prospective cohort studies. *European Journal of Clinical Nutrition* 2012; **66**(11): 1199-207.
195. Zhao LG, Sun JW, Yang Y, Ma X, Wang YY, Xiang YB. Fish consumption and all-cause mortality: a meta-analysis of cohort studies. *European Journal of Clinical Nutrition* 2016; **70**(2): 155-61.
196. Schwingshackl L, Knüppel S, Michels N, et al. Intake of 12 food groups and disability-adjusted life years from coronary heart disease, stroke, type 2 diabetes, and colorectal cancer in 16 European countries. *European journal of epidemiology* 2019; **34**(8): 765-75.
197. Miller RE, Blair PD. Input-output analysis: foundations and extensions. Cambridge, UK: Cambridge University Press; 2009.
198. Dietzenbacher E, Los B, Stehrer R, Timmer M, De Vries G. The construction of world input–output tables in the WIOD project. *Economic Systems Research* 2013; **25**(1): 71-98.
199. Stadler K, Wood R, Bulavskaya T, et al. EXIOBASE 3: Developing a time series of detailed environmentally extended multi-regional input-output tables. *Journal of Industrial Ecology* 2018; **22**(3): 502-15.
200. WBG. Consumer price index (2010 = 100). 2020. https://data.worldbank.org/indicator/FP.CPI.TOTL?end=2017&locations=US&start=2000.
201. WHO. Global Health Expenditure Database: Indicators and data. Geneva, Switzerland: World Health Organization; 2019.
202. Pichler P-P, Jaccard IS, Weisz U, Weisz H. International comparison of health care carbon footprints. *Environmental Research Letters* 2019; **14**(6): 064004.
203. Health Care Without Harm. Health Care's Climate Footprint: Health Care Without Harm, 2019.
204. Gütschow J, Jeffery L, Gieseke R, Günther A. The PRIMAP-hist national historical emissions time series (1850-2017). v2.1. 2019. https://doi.org/10.5880/pik.2019.018.
205. WHO. Current health expenditure by financing schemes, in Global Health Expenditure Database. In: Organization WH, editor.; 2020.
206. UNSD. Basic Data Selection. United Nations Statistics Division; 2019.
207. Fullman N, Yearwood J, Abay SM, et al. Measuring performance on the Healthcare Access and Quality Index for 195 countries and territories and selected subnational locations: a systematic analysis from the Global Burden of Disease Study 2016. *The Lancet* 2018; **391**(10136): 2236-71.
208. Swiss Re Institute. Sigma explorer. Zurich, Switzerland: Swiss Re; 2020.

209. Munich Re. NatCatSERVICE. 2019.
210. OECD. Executive Summary. Mortality Risk Valuation in Environment, Health and Transport Policies. Paris, France: OECD; 2012.
211. WBG. Population, total. Washington, DC, USA: World Bank Group; 2019.
212. WBG. GNI (current US$). Washington, DC, USA: World Bank Group; 2020.
213. OECD. Mortality Risk Valuation in Environment, Health and Transport Policies. OECD Publishing; 2012.
214. ILO. Indicator description: earnings and labour cost 2020.
215. WBG. World Development Indicators. Washington, DC, USA: World Bank Group, 2020.
216. IMF. World Economic Outlook Database 2019. https://www.imf.org/external/pubs/ft/weo/2019/01/weodata/index.aspx (accessed 25 April 2020).
217. IEA. World Energy Outlook 2018. Paris, France: International Energy Agency, 2018.
218. European Commission. Part III: Annexes to Impact Assessment Guidelines. Brussels, Belgium: European Commission, 2009.
219. IEA. World Energy Investment 2020. In: IEA, editor. Paris, France; 2020.
220. IRENA. Renewable Energy and Jobs: Annual Review 2020. Abu Dhabi, United Arab Emirates: International Renewable Energy Agency, 2020.
221. IBISWorld. IBISWorld Industry Report: Global Coal Mining. Los Angeles, CA: IBISWorld, 2020.
222. IBISWorld. IBISWorld Industry Report: Global Oil & Gas Exploration & Production. Los Angeles, CA: IBISWorld, 2020.
223. 350.org. Divestment Commitments. 2020. https://gofossilfree.org/divestment/commitments/ (accessed 14 April 2019).
224. IEA. Fossil fuel subsidies. 2019. https://www.iea.org/weo/energysubsidies/ (accessed 25th November 2019).
225. OECD. OECD inventory of support measures for fossil fuels. 2019. https://stats.oecd.org/Index.aspx?DataSetCode=FFS_AUS (accessed 25th November 2019).
226. OECD. OECD Companion to the Inventory of Support Measures for Fossil Fuels 2018. Paris, France: OECD Publishing; 2018.
227. WBG. Carbon Pricing Dashboard. 2019. https://carbonpricingdashboard.worldbank.org/ (accessed 25th November 2019).
228. European Commission. Auctioning. 2020. https://ec.europa.eu/clima/policies/ets/auctioning_en (accessed 25 April 2020).
229. WHO. Global Health Expenditure Database. Geneva, Switzerland: World Health Organization; 2019.
230. Crippa M, Oreggioni G, Guizzardi D, et al. Fossil CO2 and GHG emissions of all world countries. *Luxemburg: Publication Office of the European Union* 2019.
231. Giles J. Internet encyclopaedias go head to head. Nature Publishing Group; 2005.
232. Alexa. The top 500 sites on the Web. 2018. https://www.alexa.com/topsites.
233. Smith DA. Situating Wikipedia as a health information resource in various contexts: A scoping review. *PloS one* 2020; **15**(2): e0228786.
234. Wikimedia Statistics. https://stats.wikimedia.org/v2/#/all-projects (accessed April 5, 2020.
235. Wikimedia Statistics. Wikimedia Traffic Analysis Report - Page Views Per Wikipedia Language - Breakdown. 2018. https://stats.wikimedia.org/wikimedia/squids/SquidReportPageViewsPerLanguageBreakdown.htm.
236. Wikipedia. Effects of global warming on human health. 2020. https://en.wikipedia.org/wiki/Effects_of_global_warming_on_human_health.
237. Baturo A, Dasandi N, Mikhaylov SJ. Understanding state preferences with text as data: Introducing the UN General Debate corpus. *Research & Politics* 2017; **4**(2): 2053168017712821.
238. Benoit K. quanteda: Quantitative Analysis of Textual Data. R package version 1.2.3. 2018. http://quanteda.io.
239. UNFCCC. NDC Registry (interim). 2020. https://www4.unfccc.int/sites/NDCStaging/Pages/All.aspx (accessed 31 March 2020).
240. UN Global Compact. Communication on Progress. 2020. https://www.unglobalcompact.org/ (accessed 31 March 2020).
